# Palladium-catalyzed asymmetric allenylic alkylation: construction of multiple chiral thiochromanone derivatives†

**DOI:** 10.1039/d3sc01060k

**Published:** 2023-04-25

**Authors:** Li-Xia Liu, Yu-Qing Bai, Xiang Li, Chang-Bin Yu, Yong-Gui Zhou

**Affiliations:** a State Key Laboratory of Catalysis, Dalian Institute of Chemical Physics, Chinese Academy of Sciences Dalian 116023 China cbyu@dicp.ac.cn ygzhou@dicp.ac.cn; b University of Chinese Academy of Sciences Beijing 100049 China

## Abstract

The development of a new strategy for the construction of chiral cyclic sulfide-containing multiple stereogenic centers is highly desirable. Herein, by the combination of base-promoted retro-sulfa-Michael addition and palladium-catalyzed asymmetric allenylic alkylation, the streamlined synthesis of chiral thiochromanones containing two central chiralities (including a quaternary stereogenic center) and an axial chirality (allene unit) was successfully realized with up to 98% yield, 49.0 : 1 dr and >99% ee.

## Abstract 2

The development of a new strategy for the construction of chiral cyclic sulfide-containing multiple stereogenic centers is highly desirable.

## Graphical abstract

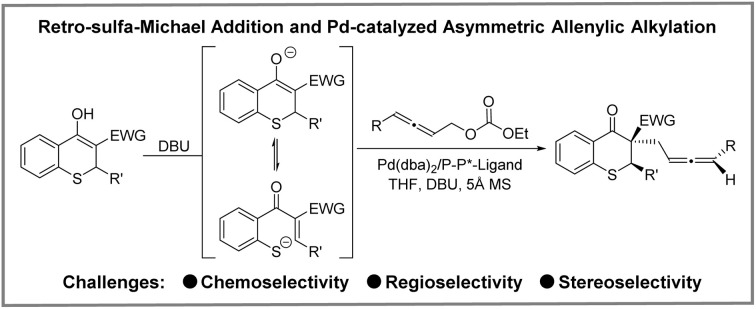

## Introduction

**Scheme 1:**
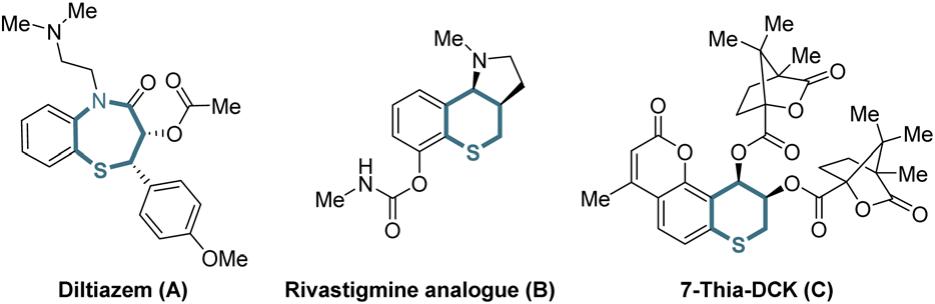
Selected bioactive cyclic sulfide derivatives.

Organic sulfur compounds play important roles in pharmaceuticals, natural products and other fields, in which molecules containing chiral cyclic sulfide skeletons generally have special biological activity.^1^ For example, diltiazem (A),^2*a*^ a calcium channel blocking agent, is used for treating supraventricular tachycardia, a rhythm disturbance of the heart. Additionally, the rivastigmine analogue (B)^2*b*^ is synthesized as an acetylcholine-esterase (AChE) inhibitor for the treatment of Alzheimer's disease, and 7-thia-DCK (C)^2*c*^ is an anti-AIDS agent (Scheme 1). Therefore, developing simple and efficient methods for the synthesis of chiral cyclic sulfide derivatives has become an intriguing area. Among the developed methods, organocatalytic sulfa-Michael-initiated cascade reactions are particularly appealing, which construct chiral cyclic sulfides containing one or more stereocenters.^3^ In contrast, metal-catalyzed asymmetric reactions^4^ are underexplored owing to the poisoning of metal catalysts by sulfur,^5^ especially sulfur anions. Considering thiochromanes are important components of cyclic sulfide derivatives,^2*b*,*c*,6^ it is vital to build chiral thiochromane derivatives containing multiple stereogenic centers.

**Scheme 2:**
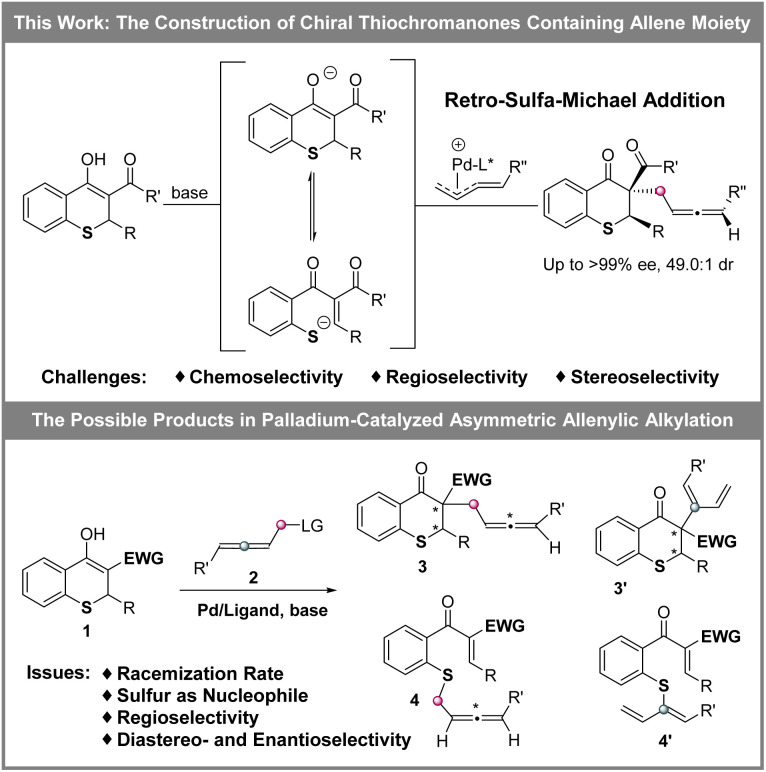
Construction of multiple chiral thiochromanone derivatives.

The allene moiety is present widely in natural products and bioactive compounds,^7^ and is an extremely versatile functional group in organic synthesis as well.^8^ Recently, Pd-catalyzed asymmetric allenylic alkylations^9^ have been developed as an efficient approach to construct chiral allenes. Previously, metal-catalyzed asymmetric reactions to build multiple chiral compounds, utilizing retro-oxa-Michael addition to simultaneously racemize two stereocenters, have been developed.^10^ However, these asymmetric reactions involving a retro-sulfa-Michael addition process were less developed, in which only organocatalytic examples were reported.^3*d*,*h*,11^ Combining palladium-catalyzed asymmetric allenylic alkylation and retro-sulfa-Michael addition to construct multiple chiral thiochromanone derivatives, some issues would need to be considered. (1) The rate of the racemization process *via* retro-sulfa-Michael addition should be faster than the rate of the next allenylic alkylation. (2) The sulfur is also nucleophilic,^12^ which would compete with the carbon nucleophile, leading to different chemoselective products. (3) Regioselective products would be observed with an electrophilic π-allylpalladium intermediate (allene partner).^13^ (4) The precise control of multiple chiralities including axial and central chirality is also a challenge. Hence, multiple possible products might be produced (Scheme 2). In this article, we reported the synthesis of enantioenriched multiple substituted thiochromanone derivatives containing two central chiralities and an axial chirality through the combination of retro-sulfa-Michael addition and palladium-catalyzed allenylic alkylation under basic conditions with up to 49.0 : 1 dr and >99% ee.

## Results and discussion

The regio- and chemoselectivity of asymmetric allenylic alkylation might be ascribed to the electron-withdrawing group (EWG) in the thiochromanone derivatives. Thus, we initially explored the effect of electron-withdrawing groups on the reaction (Table 1). Just as we speculated, different electron-withdrawing groups led to regio- and chemoselective isomers on the allenylic alkylation. When the nitro group was used as the EWG, the chemoselective isomer 4aa with sulfur as a nucleophile was observed (entry 1). Then, the desired product 3ba with carbon as a nucleophile was obtained when the acetyl group was introduced as the EWG instead of the nitro group with low diastereoselectivity (entry 2). It is delightful that 14.7 : 1 dr was gained using the methoxycarbonyl as the EWG (entry 3).

**Table 1:** Effect of the electron-withdrawing group (EWG)^a^

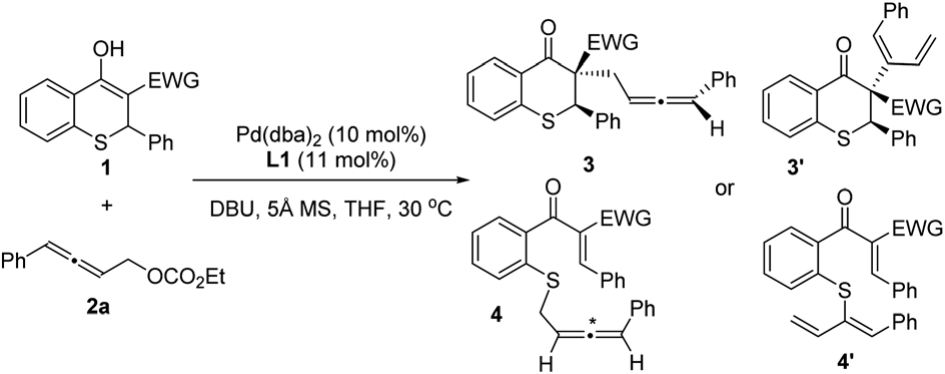						
Entry	EWG	Product	Yield^b^ (%)	d*r*^b^ (%)	ee^c^ (%)	
					Major	Minor
1	NO_2_ (1a)	4aa	74	—	84	—
2	C(O)Me (1b)	3ba	94	3.5 : 1	70	90
3	CO_2_Me (1c)	3ca	94	14.7 : 1	98	31
4	C(O)N(CH_2_)_5_ (1d)	3da	12	5.0 : 1	96	5
		4'da	84	—	—	—

a Reaction conditions: 1 (0.1 mmol), 2a (1.5 equiv.), Pd(dba)_2_ (10 mol%), L1 (11 mol%), DBU (1.2 equiv.), THF (1.0 mL), 5 Å MS (50 mg), 30 °C, 10–24 h.

b Yield and diastereomeric ratio were measured by analysis of ^1^H NMR spectra using 1,3,5-trimethoxybenzene as the internal standard.

c Determined by HPLC.

In addition, using amido as the EWG, the regio- and chemoselective isomer 4′da was discovered as the main product when nucleophilic 1d reacted with π-allylpalladium species. The yield of the desired product 3da was only 12% with 5.0 : 1 dr and 96% ee for the major diastereoisomer (entry 4). Besides, using 1,3-bis(diphenylphosphino)propane as the ligand, trace *rac*-3′da was observed, in which different isomers might be obtained owing to the effect of ligand. On the whole, methoxycarbonyl should be the optimal electron-withdrawing group, with which the allenylic alkylation could proceed smoothly to deliver the desired major isomer.

Afterwards, the optimization of the asymmetric allenylic alkylation was examined using thiochromanone 1c and allenylic carbonates 2 as substrates. First, different allenylic carbonates were screened (Table 2, entries 1–3) and ethyl (4-phenylbuta-2,3-dien-1-yl) carbonate 2a might be better (entry 1). We next investigated the effect of solvent on this alkylation reaction (entries 4–6). The reactions performed smoothly in a variety of solvents, offering good stereoselectivities and reactivities. Then, tetrahydrofuran was chosen as the optimal solvent. Next, organic and inorganic bases were investigated, and 1,8-diazabicyclo-[5.4.0]undec-7-ene (DBU) still performed better (entries 7–9).

**Table 2:** Condition optimization^a^

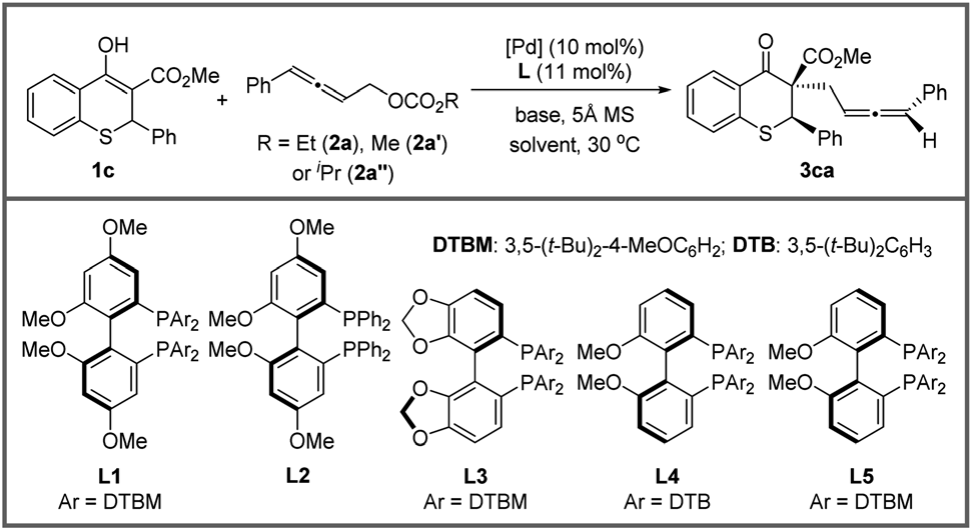						
Entry	Solvent	Base/L	[Pd]	Yield^b^ (%)	dr^b^	ee^c^ (%)
						Major/minor
1	THF	DBU/L1	Pd(dba)_2_	94	14.7 : 1	98/31
2^d^	THF	DBU/L1	Pd(dba)_2_	>95	12.7 : 1	97/59
3^e^	THF	DBU/L1	Pd(dba)_2_	>95	12.7 : 1	97/30
4	MeCN	DBU/L1	Pd(dba)_2_	91	14.2 : 1	98/42
5	DMF	DBU/L1	Pd(dba)_2_	90	14.0 : 1	97/11
6	Toluene	DBU/L1	Pd(dba)_2_	>95	11.4 : 1	96/69
7	THF	Et_3_N/L1	Pd(dba)_2_	92	5.1 : 1	90/83
8	THF	TMG/L1	Pd(dba)_2_	>95	8.7 : 1	95/84
9	THF	KO^*t*^Bu/L1	Pd(dba)_2_	>95	11.0 : 1	97/55
10	THF	DBU/L2	Pd(dba)_2_	>95	4.0 : 1	79/31
11	THF	DBU/L3	Pd(dba)_2_	>95	9.8 : 1	98/77
12	THF	DBU/L4	Pd(dba)_2_	>95	18.6 : 1	95/30
13	THF	DBU/L5	Pd(dba)_2_	>95	15.0 : 1	98/65
14	THF	DBU/L4	Pd_2_(dba)_3_	>95	15.3 : 1	98/54
15	THF	DBU/L4	[Pd(C_3_H_5_)Cl]_2_	86	13.3 : 1	98/16
16	THF	DBU/L4	Pd(OAc)_2_	>95	15.0 : 1	98/9
17^f^	THF	DBU/L4	Pd(dba)_2_	>95	31.0 : 1	98/11
18^g^	THF	DBU/L4	Pd(dba)_2_	92^h^	32.3 : 1	99/52

a Reaction conditions: 1c (0.1 mmol), 2a (1.5 equiv.), [Pd] (10 mol%), L (11 mol%), base (1.2 equiv.), solvent (1.0 mL), 5 Å MS (50 mg), 30 °C, 10–72 h.

b Yield and diastereomeric ratio were measured by analysis of ^1^H NMR spectra using 1,3,5-trimethoxybenzene as the internal standard.

c Determined by chiral HPLC.

d 2a′ instead of 2a.

e 2a′′ instead of 2a.

f 0 °C instead of 30 °C.

g −20 °C instead of 30 °C.

h Isolated yield for the reaction at the 0.2 mmol scale, 72 h.

A chiral ligand, as we all know, is the key to stereoselectivity in the asymmetric reaction. Different axially chiral bisphosphine ligands with large steric hindrance were screened in the asymmetric allenylic alkylation (entries 11–13), and the large steric hindrance was necessary (entries 1 and 10). Surprisingly, the reaction delivered the desired product in 18.6 : 1 dr with L4. Subsequently, different catalyst precursors were examined with L4, including Pd_2_(dba)_3_, [Pd(C_3_H_5_)Cl]_2_ and Pd(OAc)_2_, with which all reactions resulted in lower diastereoselectivities (entries 14–16). Lastly, to further improve the diastereoselectivity and enantioselectivity, the effect of temperature was tested (entries 17–18). When the temperature decreased, higher stereoselectivity could be achieved under −20 °C. Finally, optimized conditions were established in entry 18 of Table 2.

**Scheme 3:**
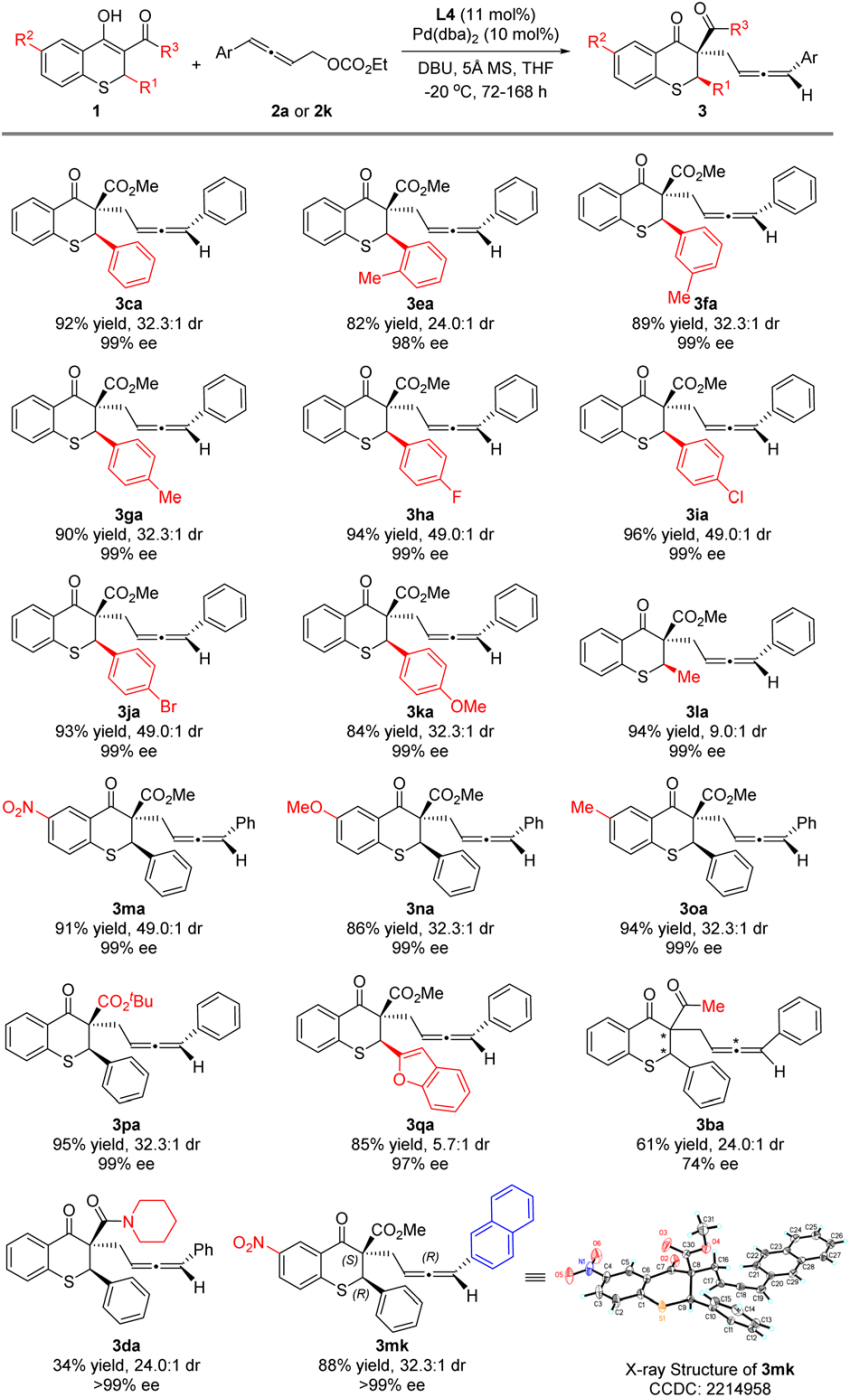
Substrate scope for the thiochromanone derivatives 1.

Under the optimized reaction conditions, thiochromanone derivatives 1b–1p were reacted with 2a to explore the generality of the substrates (Scheme 3). Fortunately, thiochromanone derivatives with various aryl substituted R^1^ could react smoothly in this palladium-catalyzed asymmetric allenylic alkylation to furnish the desirable products in good yields (82–96%), diastereoselectivities (24.0:1–49.0 : 1) and enantioselectivities (98–99%), which displayed valuable functional group tolerance in aromatic rings including methyl, fluoro, chloro, bromo and methoxyl. Among them, the substrate with an electron-rich substituent is relatively more active than that with an electron-deficient substituent (for details about reaction time, please see the ESI†). The reaction was slightly sensitive to the steric bulk of R^1^. When *o*-tolyl (1e) was introduced, the reactivity, diastereoselectivity and enantioselectivity of this alkylation were a little lower. Notably, the halogens in the *para*-position of the aromatic ring (1h–1j) could slimly improve the diastereoselectivity to 49.0 : 1. However, alkyl (methyl) had a negative effect on the diastereoselectivity (1l), which was 9.0 : 1. Furthermore, various R^2^ groups were well tolerated, such as the strong electron-withdrawing nitro group (1m), electron-donating methoxy group (1n) and methyl group (1o). Increasing the steric hindrance of the R^3^ group from methoxyl to *tert*-butoxyl, the reaction delivered the desired product 3pa in 95% yield, 32.3 : 1 dr and 99% ee for the major diastereoisomer. We also investigated the thiochromanone derivative bearing the heterocyclic aromatic substituted group (2-benzofuranyl) and found that it gave the corresponding product 3qa in 85% yield, albeit in lower diastereoselectivity (5.7 : 1 dr) and 97% ee for the major diastereoisomer. When 1b with acetyl instead of methoxycarbonyl reacted with 2a, the strong impact of ligands was observed after screening a series of chiral ligands, and (1*R*,1′*R*,2*S*,2′*S*)-DuanPhos was selected as the optimal ligand, with which the reaction delivered 3ba at 30 °C in 61% yield, 24.0 : 1 dr and 74% ee for the major diastereoisomer. When the reaction temperature was decreased to 0 °C, the reaction could not perform. Besides, thiochromanone 1d with the amide group reacted with 2a under the optimized conditions to deliver the desired product 3da with low 34% yield, in which there was the side product 4′da with 48% yield and a little recovered 1d. To assign the absolute configuration, 3mk was synthesized and its absolute configuration was assigned as (2*R*,3*S*,*R*_a_) by X-ray diffraction analysis (for details, please see the ESI†).

**Scheme 4:**
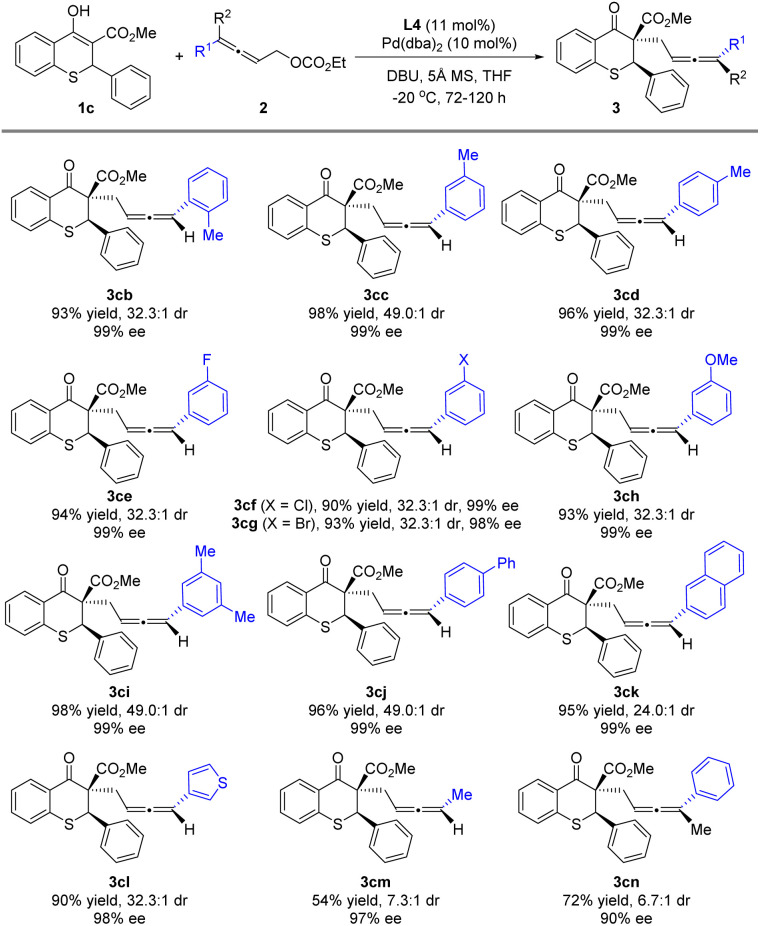
Substrate scope for the allenylic carbonates 2

Reactions of 1c with various allenylic carbonates 2b–2m were next investigated (Scheme 4). The methyl substituent at the *ortho*, *meta* or *para*-position on the benzene ring of the allenylic carbonates (2b–2d) almost did not affect the stereoselectivity of the reaction, and better diastereoselectivity was obtained with the *meta*-position substituent. Subsequently, when the fluoro, chloro, bromo or methoxy group was introduced at the *meta*-position, the alkylation reaction underwent smoothly to deliver 3ce, 3cf, 3cg or 3ch in satisfactory yield, excellent ee and dr. Similarly, the reaction was conducted with 3,5-dimethylphenyl or 4-phenylphenyl as the R, furnishing the product 3ci or 3cj in high yield and stereoselectivity. Moreover, allenylic carbonates bearing aromatic substituents, such as naphthyl (2k) and thienyl (2l), were also suitable substrates. The alkyl substituted allenylic carbonate 2m also produced the product with a 97% ee value, albeit in 54% yield and 7.3 : 1 dr. In addition, when the trisubstituted allene partner (2n) was used, the alkylation reaction underwent smoothly to deliver product 3cn in 72% yield with 6.7 : 1 dr and 90% ee for the major diastereoisomer at 30 °C, while no reaction was observed at 0 °C.

**Scheme 5:**
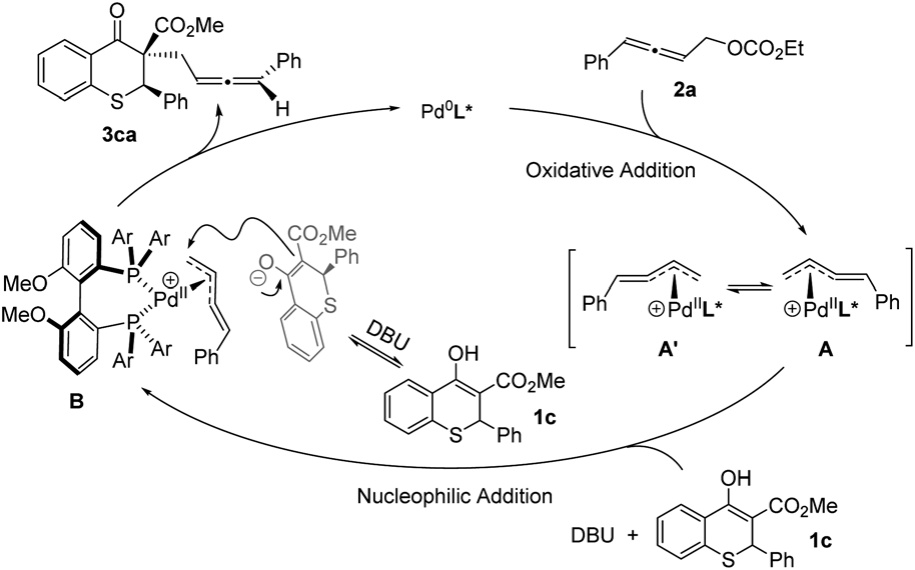
Plausible mechanism.

Based on the above experimental results and the putative mechanism on palladium-catalyzed allenylic alkylation,^9*a*,*e*,*k*^ a plausible mechanism was proposed in Scheme 5. First, oxidative addition of racemic allene 2a formed the π-allylpalladium complexes A and A′, which were in rapid equilibrium. Subsequently, the nucleophile would attack from the back side of the terminal carbon atom to give the chiral product 3ca, and regenerate the active Pd(0) species.

**Scheme 6:**
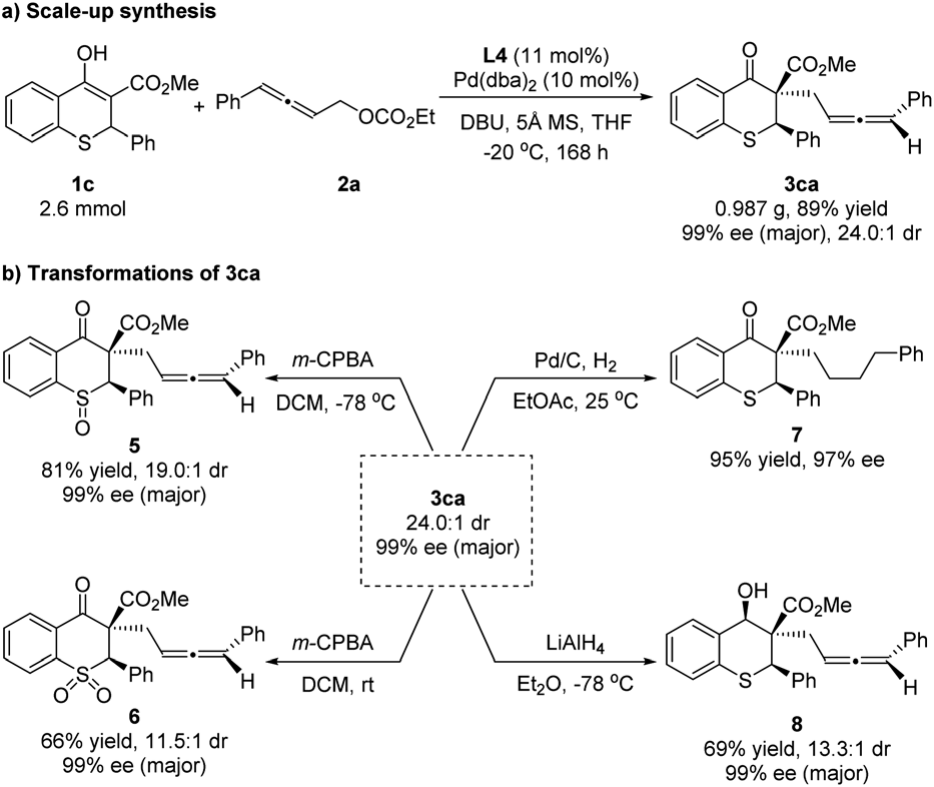
Scale-up synthesis and transformations of 3ca.

To illustrate the practicality of this asymmetric allenylic alkylation reaction, a scale-up synthesis of 3ca was carried out (Scheme 6a). The product was isolated in 89% yield with 24.0 : 1 dr and 99% ee for the major diastereoisomer under the standard conditions without loss of activity and enantioselectivity. Next, the elaboration of the product was proceeded. The chiral 3ca could be converted to the corresponding chiral sulfoxide 5 or sulfone 6 using 3-chloroperoxybenzoic acid as the oxidant at different temperatures. Furthermore, the reductions of 3ca were concentrated on carbonyl and allenyl groups, respectively. The allenyl group could be easily hydrogenated with 10% Pd/C, affording the single reductive isomer 7 in 95% yield and 97% ee, showing that the diastereoselectivity of 3ca was ascribed to the allene unit. A selective reduction of the carbonyl group of 3ca with lithium aluminum hydride (LiAlH_4_) at −78 °C proceeded smoothly, providing the chiral alcohol 8 in 69% yield, 13.3 : 1 dr and 99% ee for the major diastereoisomer (Scheme 6b). The relative configuration of the hydroxyl and phenyl in compound 8 was assigned as *cis*-8 by the NOE spectrum (for details, please see the ESI†).

## Conclusions

In summary, we have realized the synthesis of enantioenriched multiple substituted thiochromanone derivatives containing two central chiralities and an axial chirality (allene unit) based on the combination of retro-sulfa-Michael addition and palladium-catalyzed asymmetric allenylic alkylation under basic conditions, overcoming the challenges of chemo-, regio- and stereoselectivities. The reaction showed great functional group tolerance, and a broad range of highly enantioenriched products could be conveniently prepared with up to 98% yield, 49.0 : 1 dr and >99% ee. Further investigations utilizing the racemization strategy through retro-hetero-Michael addition on other reactions are being actively pursued in our laboratory.

## Data availability

Experimental data has been uploaded as part of the ESI.†

## Author contributions

L.-X. L. performed the experiments and prepared the ESI.† Y.-Q. B. and X. L. checked the data. Prof. C.-B. Y. and Y.-G. Z. conceived and directed the project. L.-X. L. and C.-B. Y. prepared the draft and Y.-G. Z. revised the manuscript.

## Conflicts of interest

There are no conflicts to declare.

## Supplementary Material

SC-014-D3SC01060K-s001

SC-014-D3SC01060K-s002
