# Supplementary material for: Palladium-catalyzed asymmetric allenylic alkylation: construction of multiple chiral thiochromanone derivatives
Source: Chem Sci. 2023 Apr 25;14(20):5477–82. doi: 10.1039/d3sc01060k (PMC10208048; doi:10.1039/d3sc01060k)
Supplement: SC-014-D3SC01060K-s001 [file SC-014-D3SC01060K-s001.pdf]

Electronic Supplementary Material (ESI) for Chemical Science.
This journal is © The Royal Society of Chemistry 2023

Supporting Information

Palladium-Catalyzed Asymmetric Allenylic Alkylation: Construction of Multiple Chiral Thiochromanone Derivatives

Li-Xia Liu, Yu-Qing Bai, Xiang Li, Chang-Bin Yu and Yong-Gui Zhou**
State Key Laboratory of Catalysis, Dalian Institute of Chemical Physics, Chinese Academy of Sciences, Dalian 116023, P. R. China; University of Chinese Academy of Sciences, Beijing 100049, P. R. China

Email: cbyu@dicp.ac.cn; ygzhou@dicp.ac.cn

Table of Contents

1. **General and Materials..S1**
2. **Synthesis of Substrates..S1-10**
3. **Palladium-Catalyzed Asymmetric Allenylic Alkylation..S11-21**
4. **Scale-up Synthesis..S22**
5. **Product Elaborations..S23-26**
6. **Determination of Absolute Configuration..S27-28**
7. **References..S29**
8. **Copy of NMR and HPLC Spectra..S30-206**

1. General and Materials

General: All reactions were carried out under an atmosphere of nitrogen using the standard Schlenk techniques, unless otherwise noted. Solvents were treated prior to use according to the standard methods. ^{1}H NMR, ^{13}C NMR and ^{19}F NMR spectra were recorded at room temperature in $CDCl_3$ on 400 MHz and 700 MHz instrument with TMS as internal standard. Enantiomeric excess was determined by HPLC analysis, using chiral column described below in detail. Optical rotations were measured by polarimeter. Flash column chromatography was performed on silica gel (200-300 mesh). The heat source for all heating reactions is the oil bath. High-resolution mass spectrometry (HRMS) was measured on an electrospray ionization (ESI) apparatus using the time-of-flight (TOF) mass spectrometry. All reactions were monitored by TLC analysis.

Materials: Commercially available reagents and solvents were used throughout without further purification.

2. Synthesis of Substrates

2.1. The Synthesis of Thiochromanone Derivatives

The thiochromanone derivatives **1a-c** and **1e-1p** were synthesized from (substituted) 2-mercaptobenzaldehydes and *α*,*β*-unsaturated ester through two steps according to the known procedure,[1] all of which are the new compounds. Firstly, the known 2-mercaptobenzaldehydes **S1** underwent the organocatalytic domino sulfa-Michael-aldol reactions with *α*,*β*-unsaturated ester **S2** to provide the intermediate alcohols. Then, the desired thiochromanone derivatives **1** could be synthesized through the oxidation with PCC.

Besides, thiochromanone derivative with amide group **1d** was synthesized through exchange from the thiochromanone **1c** and piperidine according to the known procedure.[2]

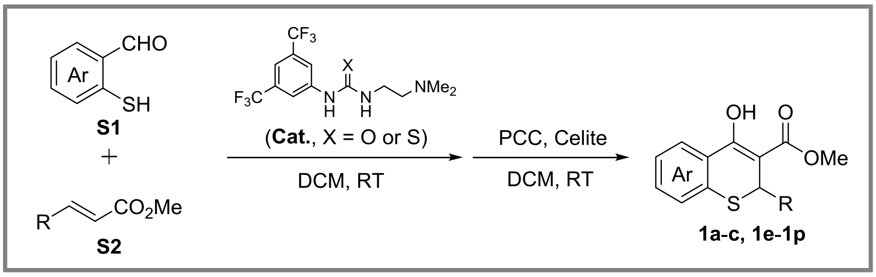

General Procedure: To a solution of (substituted) 2-mercaptobenzaldehydes **S1** (7.0 mmol) in dichloromethane (60 mL) was added organocatalyst (1.4 mmol, 20 mol%) and *α*,*β*-unsaturated esters **S2** (9.1 mmol) sequentially under nitrogen atmosphere. The reaction mixture was stirred at room temperature for about 48-120 h. The reaction was complete as monitored by TLC. Then the reaction mixture was concentrated under the reduced pressure to obtain the crude residue. The residue was purified by flash chromatography on silica gel using hexanes/ethyl acetate (20/1-10/1) as eluent to give the intermediate alcohol compounds.

To a solution of pyridinium chlorochromate (PCC, 2.5 equiv.) in dichloromethane (0.1 M) was added celite (equal in quality to PCC) and the obtained alcohol compounds at ambient temperature. TLC analysis indicated completion of the reactions after about 5-8 h. After filtration through celite, the volatiles were removed under the reduced pressure. The residue was purified by flash chromatography on silica gel using hexanes/ethyl acetate (50/1) as eluent to give the desirable thiochromanone derivatives **1**.

3-Nitro-2-phenylthiochroman-4-one (1a):

0.243 g, 30% yield (two steps), orange solid, mp 173-174 ^oC, new compound, R_f = 0.05 (hexanes/ethyl acetate 3/1), keto. ^1H NMR (400 MHz, $CDCl_3$) δ 8.18 (d, *J* = 7.6 Hz, 1H), 7.56-7.45 (m, 3H), 7.42-7.34 (m, 3H), 7.34-7.26 (m, 2H), 6.13 (d, *J* = 13.1 Hz, 1H), 5.27 (d, *J* = 13.1 Hz, 1H). ^{13}C NMR (100 MHz, $CDCl_3$) δ 184.0, 140.6, 135.2, 133.0, 130.7, 129.9, 129.4, 128.9, 128.0, 126.9, 126.2, 95.1, 48.3. HRMS Calculated for $C_{15}H_{12}NO_3S$ $[M+H]^+$ 286.0532, found: 286.0533.

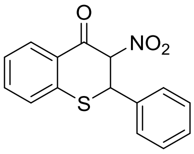

1-(4-Hydroxy-2-phenyl-2*H*-thiochromen-3-yl)ethan-1-one (1b):

0.324 g, 41% yield (two steps), yellow viscous liquid, new compound, R_f = 0.79 (hexanes/ethyl acetate 3/1), enol. ^1H NMR (400 MHz, $CDCl_3$) δ 8.05 (dd, *J* = 7.8, 1.5 Hz, 1H), 7.29-7.25 (m, 1H), 7.25-7.15 (m, 6H), 7.15-7.08 (m, 1H), 4.99 (s, 1H), 2.18 (s, 3H). ^{13}C NMR (100 MHz, $CDCl_3$) δ 198.2, 173.9, 142.0, 134.5, 132.3, 129.3, 128.7, 128.0, 127.7, 127.5, 126.8, 125.9, 106.0, 42.0, 25.2. HRMS Calculated for $C_{17}H_{15}O_2S$ $[M+H]^+$ 283.0787, found: 283.0788.

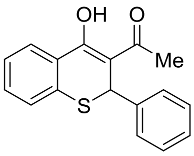

Methyl 4-hydroxy-2-phenyl-2*H*-thiochromene-3-carboxylate (1c):

1.126 g, 39% yield (two steps), pale yellow viscous liquid, new compound, R_f = 0.47 (hexanes/ethyl acetate 20/1), enol/keto = 14.3/1. The enol isomer: ^1H NMR (400 MHz, $CDCl_3$) δ 13.04 (s, 1H), 8.01-7.92 (m, 1H), 7.25-7.12 (m, 8H), 5.12 (s, 1H), 3.77 (s, 3H). ^{13}C NMR (100 MHz, $CDCl_3$) δ 171.8, 166.1, 142.7, 134.2, 131.4, 128.5, 127.8, 127.4, 126.6, 126.5, 125.6, 96.9, 52.3, 39.6. HRMS Calculated for $C_{17}H_{15}O_3S$ $[M+H]^+$ 299.0736, found: 299.0742.

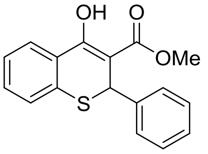

Methyl 4-hydroxy-2-(*o*-tolyl)-2*H*-thiochromene-3-carboxylate (1e):

0.701 g, 32% yield (two steps), pale yellow solid, mp 115-116 ^oC, new compound, R_f = 0.62 (hexanes/ethyl acetate 20/1), enol/keto = 12.5/1. The enol isomer: ^1H NMR (400 MHz, $CDCl_3$) δ 13.08 (s, 1H), 8.06-7.95 (m, 1H), 7.24-7.19 (m, 2H), 7.17-7.14 (m, 1H), 7.12-7.04 (m, 2H), 6.98-6.89 (m, 2H), 5.32 (s, 1H), 3.71 (s, 3H), 2.49 (s, 3H). ^{13}C NMR (100 MHz, $CDCl_3$) δ 171.8, 166.6, 139.8, 134.3, 133.9, 131.4, 130.8, 128.6, 128.1, 127.4, 126.5, 126.2, 126.1, 125.7, 96.5, 52.4, 35.7, 19.7. HRMS Calculated for $C_{18}H_{17}O_3S$ $[M+H]^+$ 313.0893, found: 313.0890.

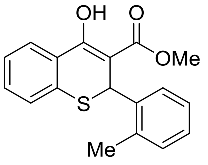

Methyl 4-hydroxy-2-(*m*-tolyl)-2*H*-thiochromene-3-carboxylate (1f):

0.575 g, 26% yield (two steps), pale yellow viscous liquid, new compound, R_f = 0.62 (hexanes/ethyl acetate 20/1), enol/keto = 14.3/1. The enol isomer: ^1H NMR (400 MHz, $CDCl_3$) δ 13.05 (s, 1H), 8.03-7.87 (m, 1H), 7.25-7.14 (m, 3H), 7.11-6.94 (m, 4H), 5.09 (s, 1H), 3.75 (s, 3H), 2.24 (s, 3H). ^{13}C NMR (100 MHz, $CDCl_3$) δ 171.9, 166.1, 142.6, 138.1, 134.2, 131.4, 128.5, 128.3, 128.3, 127.8, 127.4, 126.5, 125.6, 123.7, 96.8, 52.3, 39.6, 21.5. HRMS Calculated for $C_{18}H_{17}O_3S$ $[M+H]^+$ 313.0893, found: 313.0889.

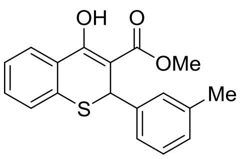

Methyl 4-hydroxy-2-(*p*-tolyl)-2*H*-thiochromene-3-carboxylate (1g):

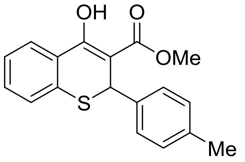

0.362 g, 17% yield (two steps), pale yellow viscous liquid, new compound, R_f = 0.62 (hexanes/ ethyl acetate 20/1), enol/keto = 14.3/1. The enol isomer: ^{1}H NMR (400 MHz, $CDCl_3$) δ 13.03 (s, 1H), 8.01-7.92 (m, 1H), 7.24-7.14 (m, 3H), 7.08 (d, *J* = 8.1 Hz, 2H), 6.99 (d, *J* = 8.0 Hz, 2H), 5.10 (s, 1H), 3.76 (s, 3H), 2.24 (s, 3H). ^{13}C NMR (100 MHz, $CDCl_3$) δ 171.9, 166.0, 139.8, 137.2, 134.2, 131.4, 129.2, 128.5, 127.8, 126.5, 126.5, 125.6, 97.0, 52.3, 39.4, 21.1. HRMS Calculated for $C_{18}H_{17}O_3S$ $[M+H]^+$ 313.0893, found: 313.0891.

Methyl 2-(4-fluorophenyl)-4-hydroxy-2*H*-thiochromene-3-carboxylate (1h):

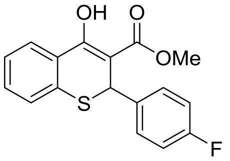

0.582 g, 26% yield (two steps), pale yellow viscous liquid, new compound, R_f = 0.50 (hexanes/ ethyl acetate 20/1), enol/keto = 20.0/1. The enol isomer: ^{1}H NMR (400 MHz, $CDCl_3$) δ 13.02 (s, 1H), 8.00-7.93 (m, 1H), 7.30-7.14 (m, 5H), 6.90-6.83 (m, 2H), 5.10 (s, 1H), 3.77 (s, 3H). ^{13}C NMR (100 MHz, $CDCl_3$) δ 171.7, 166.1, 162.1 (d, $^1J_{F\text{-}C}$ = 246.0 Hz), 138.5 (d, $^4J_{F\text{-}C}$ = 3.0 Hz), 133.9, 131.5, 128.4, 128.2 (d, $^3J_{F\text{-}C}$ = 8.3 Hz), 127.9, 126.5, 125.8, 115.3 (d, $^2J_{F\text{-}C}$ = 21.7 Hz), 97.0, 52.4, 39.0. ^{19}F NMR (376 MHz, $CDCl_3$) δ -115.11. HRMS Calculated for $C_{17}H_{14}FO_3S$ $[M+H]^+$ 317.0642, found: 317.0643.

Methyl 2-(4-chlorophenyl)-4-hydroxy-2*H*-thiochromene-3-carboxylate (1i):

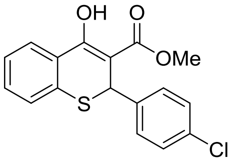

0.895 g, 38% yield (two steps), pale yellow viscous liquid, new compound, R_f = 0.50 (hexanes/ ethyl acetate 20/1), enol/keto = 14.3/1. The enol isomer: ^{1}H NMR (400 MHz, $CDCl_3$) δ 13.02 (s, 1H), 7.98-7.93 (m, 1H), 7.29-7.20 (m, 2H), 7.18-7.09 (m, 5H), 5.08 (s, 1H), 3.77 (s, 3H). ^{13}C NMR (100 MHz, $CDCl_3$) δ 171.6, 166.2, 141.2, 133.7, 133.2, 131.6, 128.6, 128.3, 128.0, 127.9, 126.6, 125.8, 96.7, 52.4, 39.0. HRMS Calculated for $C_{17}H_{14}ClO_3S$ $[M+H]^+$ 333.0347 (^{35}Cl) and 335.0321 (^{37}Cl), found: 333.0350 (^{35}Cl) and 335.0324 (^{37}Cl).

Methyl 2-(4-bromophenyl)-4-hydroxy-2*H*-thiochromene-3-carboxylate (1j):

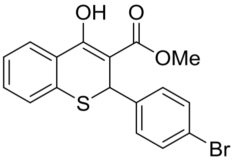

1.272 g, 48% yield (two steps), pale yellow viscous liquid, new compound, R_f = 0.50 (hexanes/ ethyl acetate 20/1), enol/keto = 25.0/1. The enol isomer: ^{1}H NMR (400 MHz, $CDCl_3$) δ 13.02 (s, 1H), 8.02-7.87 (m, 1H), 7.32-7.14 (m, 5H), 7.12-6.99 (m, 2H), 5.06 (s, 1H), 3.77 (s, 3H). ^{13}C NMR (100 MHz, $CDCl_3$) δ 171.6, 166.2, 141.7, 133.7, 131.6, 131.6, 128.4, 128.3, 127.9, 126.6, 125.9, 121.4, 96.6, 52.4, 39.1. HRMS Calculated for $C_{17}H_{14}BrO_3S$ $[M+H]^+$ 376.9842 (^{79}Br) and 378.9822 (^{81}Br), found: 376.9844 (^{79}Br) and 378.9832 (^{81}Br).

Methyl 4-hydroxy-2-(4-methoxyphenyl)-2*H*-thiochromene-3-carboxylate (1k):

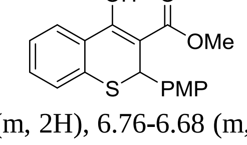

0.555 g, 34% yield (two steps, 1.0 equivalent amount of organocatalyst was used), light yellow liquid, new compound, R_f = 0.42 (hexanes/ethyl acetate 20/1), enol/keto = 11.1/1. The enol isomer: ^{1}H NMR (400 MHz, $CDCl_3$) δ 13.01 (s, 1H), 7.96 (dd, *J* = 7.7, 1.5 Hz, 1H), 7.26-7.16 (m, 3H), 7.14-7.07 (m, 2H), 6.76-6.68 (m,

2H), 5.09 (s, 1H), 3.77 (s, 3H), 3.71 (s, 3H). ^{13}C NMR (100 MHz, $CDCl_3$) δ 171.9, 165.9, 158.9, 134.9, 134.2, 131.4, 128.5, 127.9, 127.7, 126.4, 125.6, 113.8, 97.2, 55.2, 52.3, 39.1. HRMS Calculated for $C_{18}H_{16}NaO_4S$ $[M+Na]^+$ 351.0662, found: 351.0665.

Methyl 4-hydroxy-2-methyl-2*H*-thiochromene-3-carboxylate (1l):

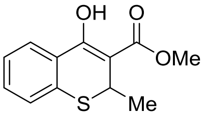

0.488 g, 42% yield (two steps), pale yellow viscous liquid, new compound, R_f = 0.67 (hexanes/ ethyl acetate 20/1), enol/keto = 11.1/1. The enol isomer: ^{1}H NMR (400 MHz, $CDCl_3$) δ 12.67 (s, 1H), 7.95-7.81 (m, 1H), 7.34-7.26 (m, 2H), 7.24-7.19 (m, 1H), 4.02 (q, *J* = 6.9 Hz, 1H), 3.86 (s, 3H), 1.35 (d, *J* = 6.9 Hz, 3H). ^{13}C NMR (100 MHz, $CDCl_3$) δ 171.6, 164.5, 134.6, 131.1, 128.2, 128.0, 126.5, 125.4, 100.0, 52.1, 31.9, 23.1. HRMS Calculated for $C_{12}H_{16}NO_3S$ $[M+NH_4]^+$ 254.0845, found: 254.0846.

Methyl 4-hydroxy-6-nitro-2-phenyl-2*H*-thiochromene-3-carboxylate (1m):

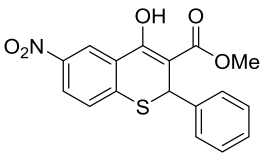

0.296 g, 12% yield (two steps), pale yellow solid, mp 182-183 ^{o}C, new compound, R_f = 0.34 (hexanes/ethyl acetate 20/1), enol. ^{1}H NMR (400 MHz, $CDCl_3$) δ 13.04 (s, 1H), 8.82 (d, *J* = 2.5 Hz, 1H), 8.08 (dd, *J* = 8.7, 2.5 Hz, 1H), 7.30 (d, *J* = 8.7 Hz, 1H), 7.25-7.08 (m, 5H), 5.25 (s, 1H), 3.80 (s, 3H). ^{13}C NMR (100 MHz, $CDCl_3$) δ 171.3, 163.8, 145.7, 143.3, 141.8, 128.8, 128.7, 128.2, 128.0, 126.5, 125.4, 121.7, 97.6, 52.7, 40.1. HRMS Calculated for $C_{17}H_{14}NO_5S$ $[M+H]^+$ 344.0587, found: 344.0589.

Methyl 4-hydroxy-6-methoxy-2-phenyl-2*H*-thiochromene-3-carboxylate (1n):

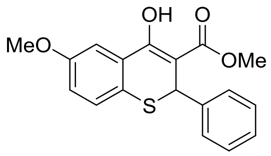

0.092 g, 9% yield (two steps), pale yellow viscous liquid, new compound, R_f = 0.54 (hexanes/ ethyl acetate 20/1), enol/keto = 12.5/1. The enol isomer: ^{1}H NMR (400 MHz, $CDCl_3$) δ 13.07 (s, 1H), 7.51 (d, *J* = 2.8 Hz, 1H), 7.23-7.10 (m, 5H), 7.06 (d, *J* = 8.6 Hz, 1H), 6.84 (dd, *J* = 8.6, 2.8 Hz, 1H), 5.09 (s, 1H), 3.82 (s, 3H), 3.76 (s, 3H). ^{13}C NMR (100 MHz, $CDCl_3$) δ 171.9, 166.1, 157.9, 142.6, 129.5, 128.9, 128.4, 127.4, 126.7, 124.8, 118.7, 110.9, 97.5, 55.5, 52.3, 39.6. HRMS Calculated for $C_{18}H_{16}NaO_4S$ $[M+Na]^+$ 351.0662, found: 351.0657.

Methyl 4-hydroxy-6-methyl-2-phenyl-2*H*-thiochromene-3-carboxylate (1o):

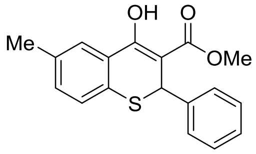

0.994 g, 40% yield (two steps), pale yellow solid, mp 143-144 ^{o}C, new compound, R_f = 0.59 (hexanes/ethyl acetate 20/1), enol/keto = 11.1/1. The enol isomer: ^{1}H NMR (400 MHz, $CDCl_3$) δ 13.05 (s, 1H), 7.78 (s, 1H), 7.23-7.10 (m, 5H), 7.10-7.01 (m, 2H), 5.10 (s, 1H), 3.76 (s, 3H), 2.34 (s, 3H). ^{13}C NMR (100 MHz, $CDCl_3$) δ 171.9, 166.3, 142.8, 135.5, 132.4, 130.6, 128.4, 128.3, 127.7, 127.4, 126.9, 126.6, 97.0, 52.3, 39.6, 21.1. HRMS Calculated for $C_{18}H_{17}O_3S$ $[M+H]^+$ 313.0893, found: 313.0892.

***tert*-Butyl 4-hydroxy-2-phenyl-2*H*-thiochromene-3-carboxylate (1p):**

0.250 g, 8% yield (two steps), pale yellow viscous liquid, new compound, R_f = 0.62 (hexanes/ ethyl acetate 20/1), enol/keto = 2.5/1. The enol and keto mixture: ^{1}H NMR (400 MHz, $CDCl_3$) δ 13.24 (s, 1H, enol), 8.15 (dd, *J* = 8.0, 1.2 Hz, 1H, keto), 7.95 (dd, *J* = 7.7, 1.5 Hz, 1H, enol),

7.45-7.16 (m, 16H, enol and keto), 5.05 (s, 1H, enol), 4.93 (d, *J* = 12.6 Hz, 1H, keto), 4.19 (d, *J* = 12.6 Hz, 1H, keto), 1.42 (s, 9H, enol), 1.23 (s, 9H, keto). ^{13}C NMR (100 MHz, $CDCl_3$) δ 190.9, 171.3, 166.9, 165.3, 143.5, 141.5, 136.5, 134.0, 133.9, 131.1, 129.9, 129.7, 128.8, 128.8, 128.7, 128.4, 128.3, 127.6, 127.3, 126.9, 126.4, 126.3, 125.5, 125.4, 98.6, 82.6, 82.2, 62.6, 47.6, 40.2, 28.1, 27.6. HRMS Calculated for $C_{20}H_{21}O_3S$ $[M+H]^+$ 341.1206, found: 341.1203.

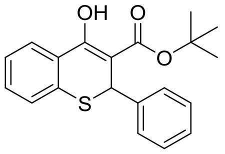

Methyl 2-(benzofuran-2-yl)-4-hydroxy-2*H*-thiochromene-3-carboxylate (1q):
0.732 g, 23% yield (two steps), yellow solid, mp 146-147 ^{o}C, new compound, R_f = 0.51 (hexanes/ ethyl acetate 20/1), enol. ^{1}H NMR (400 MHz, $CDCl_3$) δ 13.03 (s, 1H), 7.98 (dd, *J* = 7.3, 1.8 Hz, 1H), 7.38 (dd, *J* = 18.4, 8.0 Hz, 2H), 7.27-7.17 (m, 4H), 7.14-7.09 (m, 1H), 6.30 (s, 1H), 5.31 (s, 1H), 3.80 (s, 3H). ^{13}C NMR (100 MHz, $CDCl_3$) δ 171.5, 166.5, 156.5, 155.1, 133.9, 131.6, 128.2, 128.1, 128.0, 126.8, 125.9, 124.3, 122.7, 120.8, 111.3, 104.8, 94.8, 52.4, 34.4. HRMS Calculated for $C_{19}H_{14}NaO_4S$ $[M+Na]^+$ 361.0505, found: 361.0499.

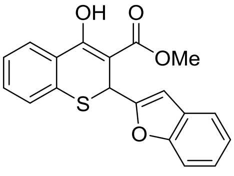

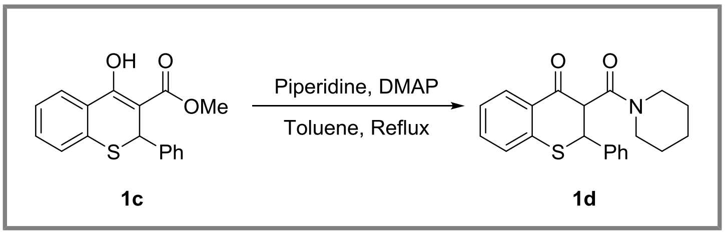

To a solution of thiochromanone derivative **1c** (2.5 mmol, 0.746 g) in toluene (25 mL) was added the piperidine (5.0 mmol, 0.426 g) and 4-dimethylaminopyridine (DMAP) (1.0 mmol, 0.122 g) sequentially. The mixture was refluxed for 24 hours. After cooling to room temperature, the reaction mixture was concentrated under reduced pressure to afford the crude residue. The residue was purified by flash chromatography on silica gel using hexanes/ethyl acetate/dichloromethane (20/1/1-3/1/1) as eluent to give the desirable thiochromanone derivative with amide **1d**.

2-Phenyl-3-(piperidine-1-carbonyl)thiochroman-4-one (1d):
0.335 g, 38% yield, white solid, mp 202-203 ^{o}C, new compound, R_f = 0.43 (hexanes/ethyl acetate 3/1), keto. ^{1}H NMR (400 MHz, $CDCl_3$) δ 8.12 (dd, *J* = 8.0, 1.3 Hz, 1H), 7.51-7.40 (m, 3H), 7.38-7.26 (m, 4H), 7.25-7.18 (m, 1H), 5.27 (d, *J* = 12.5 Hz, 1H), 4.54 (d, *J* = 12.5 Hz, 1H), 3.71-3.55 (m, 1H), 3.53-3.41 (m, 1H), 3.39-3.30 (m, 1H), 3.29-3.11 (m, 1H), 1.65-1.37 (m, 4H), 1.27-1.04 (m, 2H). ^{13}C NMR (100 MHz, $CDCl_3$) δ 192.0, 166.0, 141.5, 137.3, 133.9, 130.5, 129.9, 128.8, 128.5, 128.1, 127.2, 125.5, 58.5, 48.5, 47.5, 43.1, 26.3, 25.5, 24.5. HRMS Calculated for $C_{21}H_{22}NO_2S$ $[M+H]^+$ 352.1366, found: 352.1367.

2.2. Synthesis of Allenylic Carbonates

The allenylic carbonates **2a-2m** could be conveniently synthesized from the commercially available propargyl alcohol and aldehydes through two steps according to the known procedure,[3,4] all of which are new compounds. Firstly, the propargyl alcohol underwent copper-catalyzed reactions with aldehydes to provide the intermediate allenylic alcohols.[3] Then, the allenylic carbonates **2** were prepared from the above allenylic alcohol intermediates according to the known literature procedure with slight modification.[4] In addition, allenylic carbonate **2n**, a new compound, could be synthesized through two steps according to the known procedure.[4,5] Firstly, the propargylic dioxolanone **S1** underwent copper-catalyzed S_N2' substitution with Grignard reagent to provide the intermediate α-hydroxyallene.[5] Then, the allenylic carbonate **2n** was prepared from the above α-hydroxyallene intermediate according to the known literature procedure with slight modification.[4]

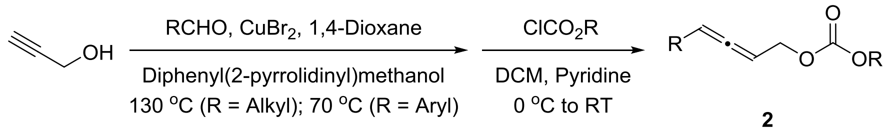

General Procedure: To a dried Schlenk flask were sequentially added copper dibromide (1.787 g, 8.0 mmol), diphenyl(2-pyrrolidinyl)methanol (5.067 g, 20 mmol), 1,4-dioxane (40 mL), aldehydes (30 mmol), and propargyl alcohol (1.682 g, 30 mmol) under the nitrogen atmosphere. The mixture was stirred in an oil bath preheated at 70 °C or 130 °C for 12-20 h. After cooling to room temperature, the resulting mixture was diluted with diethyl ether (60 mL), which was washed with an aqueous solution of hydrochloric acid (3.0 M, 60 mL). After separation, the aqueous layer was extracted with diethyl ether (60 mL×3). The combined organic layer was dried over anhydrous sodium sulfate, filtered and concentrated under the reduced pressure. The residue was purified by flash chromatography on silica gel using hexanes/ethyl acetate (20/1-5/1) as eluent to afford the intermediate allenylic alcohols.

A solution of the above allenylic alcohols (9.7 mmol) and pyridine (2.34 mL, 29.1 mmol) in dichloromethane (25 mL) was cooled to 0 ℃. The ethyl chloroformate (1.85 mL, 19.4 mmol) (or isopropyl chloroformate) was added dropwise over a period of 5 minutes. The reaction mixture was allowed to warm to room temperature. When the reaction was completed as monitored by TLC, the reaction mixture was acidified with the hydrogen chloride aqueous solution (3.0 M) to pH 5-6 and extracted three times with dichloromethane. The combined organic layer was dried over anhydrous sodium sulfate, filtered and concentrated under the reduced pressure. The residue was purified by flash chroma- tography on silica gel using hexanes/ethyl acetate (50/1) as eluent to give the desirable allenylic carbonates **2**.

Ethyl (4-phenylbuta-2,3-dien-1-yl) carbonate (2a):

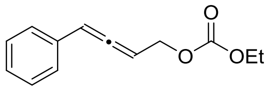

0.184 g, 24% yield (two steps), yellow liquid, new compound, R_f = 0.49 (hexanes/ethyl acetate 10/1). ^{1}H NMR (400 MHz, $CDCl_3$) δ 7.33-7.28 (m, 4H), 7.25-7.14 (m, 1H), 6.37-6.28 (m, 1H), 5.84-5.68 (m, 1H), 4.78-4.67 (m, 2H), 4.20 (q, *J* = 7.1 Hz, 2H), 1.29 (t, *J* = 7.1 Hz, 3H). ^{13}C NMR (100 MHz, $CDCl_3$) δ 206.7, 155.0, 133.2, 128.7, 127.4, 127.1, 96.7, 90.8, 65.1, 64.2, 14.3. HRMS Calculated for

$C_{13}H_{18}NO_3$ $[M+NH_4]^+$ 236.1281, found: 236.1280.

Isopropyl (4-phenylbuta-2,3-dien-1-yl) carbonate (2a″):

1.120 g, 45% yield (two steps), yellow liquid, new compound, R_f = 0.56 (hexanes/ethyl acetate 20/1). ^{1}H NMR (400 MHz, $CDCl_3$) δ 7.33-7.27 (m, 4H), 7.25-7.19 (m, 1H), 6.37-6.26 (m, 1H), 5.83-5.64 (m, 1H), 4.92-4.83 (m, 1H), 4.76-4.66 (m, 2H), 1.30 (d, *J* = 6.3 Hz, 3H), 1.26 (d, *J* = 6.3 Hz, 3H). ^{13}C NMR (100 MHz, $CDCl_3$) δ 206.6, 154.5, 133.2, 128.7, 127.4, 127.1, 96.7, 90.9, 72.2, 64.9, 21.8, 21.7. HRMS Calculated for $C_{14}H_{16}NaO_3$ $[M+Na]^+$ 255.0992, found: 255.0993.

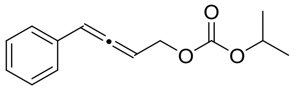

Ethyl (4-(*o*-tolyl)buta-2,3-dien-1-yl) carbonate (2b):

0.191 g, 4% yield (two steps), yellow liquid, new compound, R_f = 0.53 (hexanes/ethyl acetate 20/1). ^{1}H NMR (400 MHz, $CDCl_3$) δ 7.39-7.32 (m 1H), 7.18-7.10 (m, 3H), 6.58-6.43 (m, 1H), 5.72 (q, *J* = 6.7 Hz, 1H), 4.77-4.69 (m, 2H), 4.21 (q, *J* = 7.1 Hz, 2H), 2.36 (s, 3H), 1.30 (t, *J* = 7.1 Hz, 3H). ^{13}C NMR (100 MHz, $CDCl_3$) δ 207.4, 155.0, 135.3, 131.4, 130.5, 127.6, 127.4, 126.2, 94.0, 89.8, 65.4, 64.2, 19.9, 14.3. HRMS Calculated for $C_{14}H_{17}O_3$ $[M+H]^+$ 233.1172, found: 233.1174.

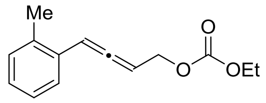

Ethyl (4-(*m*-tolyl)buta-2,3-dien-1-yl) carbonate (2c):

1.212 g, 26% yield (two steps), yellow liquid, new compound, R_f = 0.53 (hexanes/ethyl acetate 20/1). ^{1}H NMR (400 MHz, $CDCl_3$) δ 7.20 (t, *J* = 7.5 Hz, 1H), 7.13-7.07 (m, 2H), 7.04 (d, *J* = 7.5 Hz, 1H), 6.33-6.25 (m, 1H), 5.80-5.69 (m, 1H), 4.77-4.68 (m, 2H), 4.20 (q, *J* = 7.1 Hz, 2H), 2.33 (s, 3H), 1.30 (t, *J* = 7.1 Hz, 3H). ^{13}C NMR (100 MHz, $CDCl_3$) δ 206.7, 155.0, 138.3, 133.0, 128.6, 128.3, 127.7, 124.2, 96.7, 90.6, 65.3, 64.2, 21.4, 14.3. HRMS Calculated for $C_{14}H_{16}NaO_3$ $[M+Na]^+$ 255.0992, found: 255.0994.

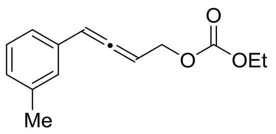

Ethyl (4-(*p*-tolyl)buta-2,3-dien-1-yl) carbonate (2d):

1.571 g, 34% yield (two steps), yellow liquid, new compound, R_f = 0.53 (hexanes/ethyl acetate 20/1). ^{1}H NMR (400 MHz, $CDCl_3$) δ 7.19 (d, *J* = 8.0 Hz, 2H), 7.12 (d, *J* = 8.0 Hz, 2H), 6.35-6.21 (m, 1H), 5.84-5.63 (m, 1H), 4.77-4.67 (m, 2H), 4.20 (q, *J* = 7.1 Hz, 2H), 2.33 (s, 3H), 1.30 (t, *J* = 7.1 Hz, 3H). ^{13}C NMR (100 MHz, $CDCl_3$) δ 206.5, 155.0, 137.3, 130.1, 129.4, 127.0, 96.5, 90.6, 65.3, 64.2, 21.3, 14.3. HRMS Calculated for $C_{14}H_{16}KO_3$ $[M+K]^+$ 271.0731, found: 271.0732.

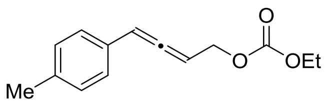

Ethyl (4-(3-fluorophenyl)buta-2,3-dien-1-yl) carbonate (2e):

1.775 g, 38% yield (two steps), yellow liquid, new compound, R_f = 0.44 (hexanes/ethyl acetate 20/1). ^{1}H NMR (400 MHz, $CDCl_3$) δ 7.29-7.26 (m, 1H), 7.06 (d, *J* = 7.7 Hz, 1H), 7.00 (d, *J* = 9.9 Hz, 1H), 6.96-6.86 (m, 1H), 6.36-6.22 (m, 1H), 5.90-5.64 (m, 1H), 4.78-4.68 (m, 2H), 4.20 (q, *J* = 7.1 Hz, 2H), 1.30 (t, *J* = 7.1 Hz, 3H). ^{13}C NMR (100 MHz, $CDCl_3$) δ 206.8, 163.1 (d, $^1J_{F\text{-}C}$ = 243.9 Hz), 154.9, 135.7 (d, $^3J_{F\text{-}C}$ = 7.9 Hz), 130.1 (d, $^3J_{F\text{-}C}$ = 8.3 Hz), 122.8 (d, $^4J_{F\text{-}C}$ = 2.7 Hz), 114.3 (d, $^2J_{F\text{-}C}$ = 21.4 Hz), 113.6 (d, $^2J_{F\text{-}C}$ = 22.12 Hz), 96.1 (d, $^4J_{F\text{-}C}$ = 2.6 Hz), 91.2, 64.8, 64.3, 14.3. ^{19}F NMR

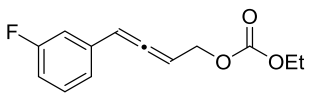

(376 MHz, $CDCl_3$) δ -113.32. The HRMS Calculated for $C_{13}H_{14}FO_3$ $[M+H]^+$ 237.0921, found: 237.0922.

4-(3-Chlorophenyl)buta-2,3-dien-1-yl ethyl carbonate (2f):

2.096 g, 42% yield (two steps), yellow liquid, new compound, R_f = 0.39 (hexanes/ethyl acetate 20/1).

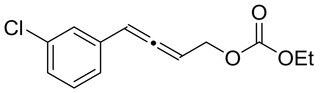

^1H NMR (400 MHz, $CDCl_3$) δ 7.28 (s, 1H), 7.26-7.14 (m, 3H), 6.31-6.23 (m, 1H), 5.87-5.73 (m, 1H), 4.77-4.69 (m, 2H), 4.21 (q, J = 7.1 Hz, 2H), 1.30 (t, J = 7.1 Hz, 3H). ^{13}C NMR (100 MHz, $CDCl_3$) δ 206.8, 154.9, 135.3, 134.6, 129.9, 127.4, 126.9, 125.2, 95.9, 91.3, 64.7, 64.3, 14.3. HRMS Calculated for $C_{13}H_{17}ClNO_3$ $[M+NH_4]^+$ 270.0891 (^{35}Cl) and 272.0866 (^{37}Cl), found: 270.0889 (^{35}Cl) and 272.0856 (^{37}Cl).

4-(3-Bromophenyl)buta-2,3-dien-1-yl ethyl carbonate (2g):

2.252 g, 38% yield (two steps), yellow liquid, new compound, R_f = 0.38 (hexanes/ethyl acetate 20/1).

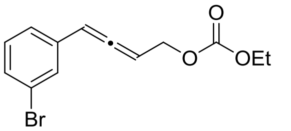

^1H NMR (400 MHz, $CDCl_3$) δ 7.43 (s, 1H), 7.38-7.30 (m, 1H), 7.23-7.13 (m, 2H), 6.37-6.15 (m, 1H), 5.88-5.69 (m, 1H), 4.77-4.68 (m, 2H), 4.21 (q, J = 7.1 Hz, 2H), 1.30 (t, J = 7.1 Hz, 3H). ^{13}C NMR (100 MHz, $CDCl_3$) δ 206.7, 154.9, 135.5, 130.3, 130.1, 129.8, 125.7, 122.8, 95.8, 91.4, 64.7, 64.3, 14.3. HRMS Calculated for $C_{13}H_{17}BrNO_3$ $[M+NH_4]^+$ 314.0386 (^{79}Br) and 316.0367 (^{81}Br), found: 314.0392 (^{79}Br) and 316.0374 (^{81}Br).

Ethyl (4-(3-methoxyphenyl)buta-2,3-dien-1-yl) carbonate (2h):

1.390 g, 28% yield (two steps), yellow liquid, new compound, R_f = 0.34 (hexanes/ethyl acetate 20/1).

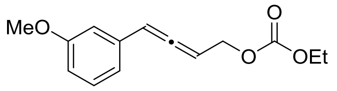

^1H NMR (400 MHz, $CDCl_3$) δ 7.22 (t, J = 7.9 Hz, 1H), 6.92-6.825 (m, 2H), 6.78 (dd, J = 8.2, 2.3 Hz, 1H), 6.33-6.22 (m, 1H), 5.83-5.65 (m, 1H), 4.77-4.68 (m, 2H), 4.19 (q, J = 7.1 Hz, 2H), 3.81 (s, 3H), 1.29 (t, J = 7.1 Hz, 3H). ^{13}C NMR (100 MHz, $CDCl_3$) δ 206.7, 159.9, 154.9, 134.6, 129.6, 119.7, 113.3, 112.2, 96.7, 90.9, 65.1, 64.2, 55.2, 14.3. HRMS Calculated for $C_{14}H_{17}O_4$ $[M+H]^+$ 249.1121, found: 249.1119.

4-(3,5-Dimethylphenyl)buta-2,3-dien-1-yl ethyl carbonate (2i):

2.200 g, 45% yield (two steps), pale yellow liquid, new compound, R_f = 0.45 (hexanes/ethyl acetate 20/1).

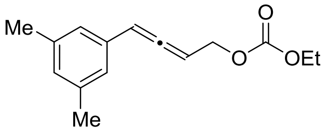

^1H NMR (400 MHz, $CDCl_3$) δ 6.91 (s, 2H), 6.86 (s, 1H), 6.27-6.20 (m, 1H), 5.83-5.62 (m, 1H), 4.75-4.68 (m, 2H), 4.24-4.16 (m, 2H), 2.29 (s, 6H), 1.30 (t, J = 7.1 Hz, 3H). ^{13}C NMR (100 MHz, $CDCl_3$) δ 206.7, 155.0, 138.2, 132.9, 129.2, 124.9, 96.7, 90.5, 65.3, 64.2, 21.2, 14.3. HRMS Calculated for $C_{15}H_{19}O_3$ $[M+H]^+$ 247.1329, found: 247.1328.

4-([1,1'-Biphenyl]-4-yl)buta-2,3-dien-1-yl ethyl carbonate (2j):

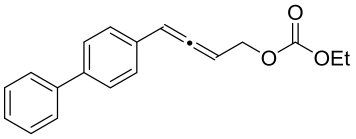

2.123 g, 36% yield (two steps), yellow liquid, new compound, R_f = 0.32 (hexanes/ethyl acetate 20/1). ^1H NMR (400 MHz, $CDCl_3$) δ 7.60-7.53 (m, 4H), 7.45-7.41 (m, 2H), 7.38-7.31 (m, 3H), 6.38-6.33 (m, 1H), 5.86-5.69 (m, 1H), 4.79-4.69 (m, 2H),

4.24-4.17 (m, 2H), 1.30 (t, *J* = 7.1 Hz, 3H). ^{13}C NMR (100 MHz, $CDCl_3$) δ 206.9, 155.0, 140.7, 140.3, 132.2, 128.8, 127.5, 127.4, 127.4, 127.0, 96.4, 90.9, 65.1, 64.2, 14.3. HRMS Calculated for $C_{19}H_{19}O_3$ $[M+H]^+$ 295.1329, found: 295.1327.

Ethyl (4-(naphthalen-2-yl)buta-2,3-dien-1-yl) carbonate (2k):

1.834 g, 34% yield (two steps), pale yellow liquid, new compound, R_f = 0.57 (hexanes/ethyl acetate 20/1). ^{1}H NMR (400 MHz, $CDCl_3$) δ 7.82-7.73 (m, 3H), 7.65 (s, 1H), 7.48-7.38 (m, 3H), 6.57-6.42 (m, 1H), 5.94-5.67 (m, 1H), 4.81-4.71 (m, 2H), 4.25-4.14 (m, 2H), 1.28 (t, *J* = 7.1 Hz, 3H). ^{13}C NMR (100 MHz, $CDCl_3$) δ 207.2, 155.0, 133.6, 132.9, 130.7, 128.4, 127.8, 127.8, 126.4, 126.1, 125.9, 124.7, 97.1, 91.1, 65.2, 64.2, 14.3. HRMS Calculated for $C_{17}H_{20}NO_3$ $[M+NH_4]^+$ 286.1438, found: 286.1437.

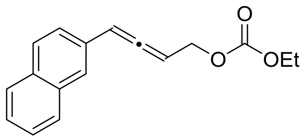

Ethyl (4-(thiophen-3-yl)buta-2,3-dien-1-yl) carbonate (2l):

1.726 g, 38% yield (two steps), yellow liquid, new compound, R_f = 0.62 (hexanes/ethyl acetate 20/1). ^{1}H NMR (400 MHz, $CDCl_3$) δ 7.27-7.25 (m, 1H), 7.11 (d, *J* = 2.8 Hz, 1H), 7.09-7.02 (m, 1H), 6.45-6.33 (m, 1H), 5.77-5.59 (m, 1H), 4.74-4.65 (m, 2H), 4.19 (q, *J* = 7.1 Hz, 2H), 1.29 (t, *J* = 7.1 Hz, 3H). ^{13}C NMR (100 MHz, $CDCl_3$) δ 207.0, 154.9, 134.3, 126.4, 126.1, 121.7, 91.3, 90.0, 65.1, 64.2, 14.3. HRMS Calculated for $C_{11}H_{12}NaO_3S$ $[M+Na]^+$ 247.0399, found: 247.0401.

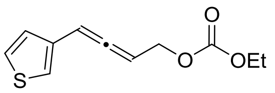

Ethyl penta-2,3-dien-1-yl carbonate (2m):

0.408 g, 13% yield (two steps), colorless liquid, new compound, R_f = 0.58 (hexanes/ethyl acetate 20/1). ^{1}H NMR (400 MHz, $CDCl_3$) δ 5.41-5.10 (m, 2H), 4.63-4.56 (m, 2H), 4.20 (q, *J* = 7.1 Hz, 2H), 1.68 (dd, *J* = 5.7, 4.5 Hz, 3H), 1.31 (t, *J* = 7.1 Hz, 3H). ^{13}C NMR (100 MHz, $CDCl_3$) δ 206.5, 155.0, 87.7, 85.9, 66.0, 64.0, 14.3, 13.8. HRMS Calculated for $C_8H_{13}O_3$ $[M+H]^+$ 157.0859, found: 157.0859.

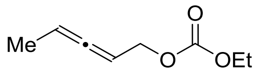

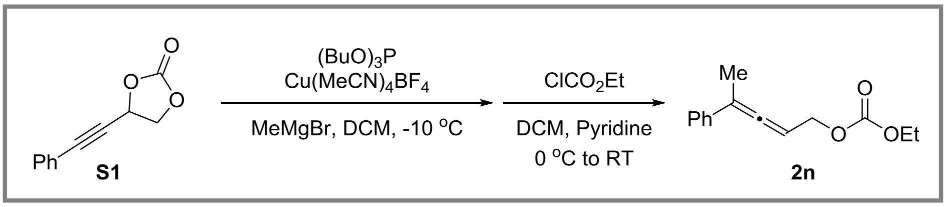

To a dried Schlenk flask were sequentially added catalyst $Cu(MeCN)_4BF_4$ (0.189 g, 0.6 mmol), (*n*-BuO)$_3$P (0.32 mL, 1.2 mmol) and dichloromethane (10 mL) under the nitrogen atmosphere. The mixture was stirred at room temperature for 1 h. After cooling to -10 °C, the methylmagnesium bromide (12 mL, 1.0 M in tetrahydrofuran, 12 mmol) was added and the reaction was stirred for 30 min at -10 °C. Then, a solution of the dioxolanone **S1** (1.129 g, 6 mmol) in dichloromethane (5.0 mL) was added and the reaction mixture was stirred at -10 °C for 22 h. Afterwards, the reaction mixture was quenched with ammonium chloride saturated solution. After separation, the aqueous layer was extracted with diethyl ether (20 mL×3). The combined organic layer was dried over anhydrous sodium sulfate, filtered and concentrated under the reduced pressure. The residue was purified by flash chromatography on silica gel using hexanes/ethyl

acetate (20/1-10/1) as eluent to afford the intermediate α-hydroxyallene.

A solution of the above α-hydroxyallene (0.343 g, 2.1 mmol) and pyridine (0.51 mL, 6.3 mmol) in dichloromethane (10 mL) was cooled to 0 ℃. The ethyl chloroformate (0.40 mL, 4.2 mmol) was added dropwise over a period of 5 minutes. The reaction mixture was allowed to warm to room temperature. When the reaction was completed as monitored by TLC, the reaction mixture was acidified with the hydrogen chloride aqueous solution (3.0 M) to pH 5-6 and extracted three times with dichloromethane. The combined organic layer was dried over anhydrous sodium sulfate, filtered and concentrated under the reduced pressure. The residue was purified by flash chromatography on silica gel using hexanes/ethyl acetate (50/1) as eluent to give the desirable allenylic carbonate **2n**.

Ethyl (4-phenylpenta-2,3-dien-1-yl) carbonate (2n):
0.451 g, 33% yield (two steps), pale yellow liquid, new compound, R_f = 0.67 (hexanes/ethyl acetate 20/1). ^{1}H NMR (400 MHz, $CDCl_3$) δ 7.42-7.37 (m, 2H), 7.35-7.29 (m, 2H), 7.25-7.19 (m, 1H), 5.73-5.57 (m, 1H), 4.70 (d, *J* = 6.8 Hz, 2H), 4.20 (q, *J* = 7.1 Hz, 2H), 2.12 (d, *J* = 2.8 Hz, 3H), 1.30 (t, *J* = 7.1 Hz, 3H). ^{13}C NMR (100 MHz, $CDCl_3$) δ 205.8, 155.0, 136.0, 128.4, 127.1, 125.9, 102.9, 88.7, 65.7, 64.1, 16.8, 14.3. HRMS Calculated for $C_{14}H_{20}NO_3$ $[M+NH_4]^+$ 250.1438, found: 250.1443.

---

3. Palladium-Catalyzed Asymmetric Allenylic Alkylation

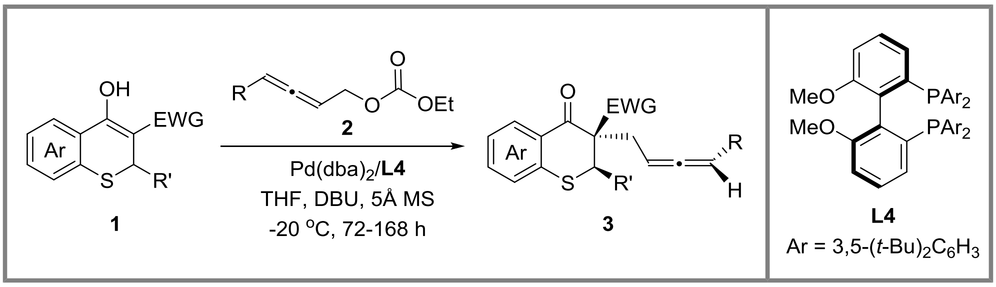

The metal precursor Pd(dba)$_2$ (0.02 mmol, 11.5 mg, 10 mol%), ligand (*R*)-DTB-BIPHEP (**L4**) (0.022 mmol, 22.7 mg, 11 mol%) and tetrahydrofuran (1.5 mL) were placed in a dried Schlenk tube under nitrogen atmosphere. The mixture was stirred at 30 ^{o}C for 30 min. Then the mixture was cooled to -20 ^{o}C. Thereafter, the thiochromanone derivatives **1** (0.2 mmol), 5Å MS (50.0 mg) and 1,8-diazabicyclo[5,4,0]undec-7-ene (DBU) (35.9 μL, 0.24 mmol) were added and the reaction was stirred at -20 ^{o}C for 10 minutes. Sequentially, the allenylic carbonates **2** (0.3 mmol) and tetrahydrofuran (0.5 mL) were added slowly. The mixture was stirred at -20 ^{o}C for 72-168 h. After the completion of the reaction, the volatiles were directly removed under the reduced pressure. The residue was quickly purified by column chromatography on silica gel (hexanes/ethyl acetate/dichloromethane 100/1/2-100/3/6) to give the desirable allenylic alkylation products **3**.

The racemates were prepared by running reactions with an achiral 1,3-bis(diphenylphosphino)-propane ligand at 30 ^{o}C. It was worth noting that the products **3** were sensitive to water or alcohol, which would lead to partial decrease of dr value.

(-)-Methyl 4-oxo-2-phenyl-3-(4-phenylbuta-2,3-dien-1-yl)thiochromane-3-carboxylate (3ca): The reaction was conducted at -20 ^{o}C for 72 h. 78.5 mg, 92% yield, pale yellow viscous liquid, new compound, R$_f$ = 0.28 (hexanes/ethyl acetate 20/1), 32.3:1 dr, 99% ee (major diastereoisomer), $[\alpha]^{20}_D$ = -209.67 (*c* 0.63, CHCl$_3$). The major diastereoisomer: ^{1}H NMR (400 MHz, CDCl$_3$) δ 8.17 (d, *J* = 7.9 Hz, 1H), 7.43-7.31 (m, 6H), 7.27-7.22 (m, 4H), 7.20-7.10 (m, 3H), 6.22-5.96 (m, 1H), 5.66-5.43 (m, 1H), 4.98 (s, 1H), 3.65 (s, 3H), 3.22-3.02 (m, 1H), 2.73-2.51 (m, 1H). ^{13}C NMR (100 MHz, CDCl$_3$) δ 207.4, 192.4, 169.7, 141.5, 135.6, 133.9, 133.2, 130.8, 130.5, 129.2, 128.9, 128.6, 128.5, 127.3, 127.1, 126.8, 125.5, 95.5, 89.9, 62.7, 52.4, 51.1, 32.5. HPLC: Chiralpak IC column, 254 nm, 30 ^{o}C, *n*-Hexane/*i*-PrOH = 95/5, flow = 1.0 mL/min, retention time 15.0 min (minor) and 16.0 min (major). HRMS Calculated for the $C_{27}H_{23}O_3S$ [M+H]$^+$ 427.1362, found: 427.1372.

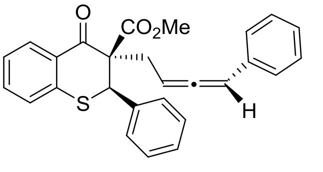

The mixtrue with low 2.3:1 dr: ^{1}H NMR (400 MHz, CDCl$_3$) δ 8.17 (d, *J* = 7.9 Hz, 1H, major), 7.88 (d, *J* = 7.9 Hz, 1H, minor), 7.40-7.12 (m, 26H, major + minor), 6.16-6.01 (m, 2H, major + minor), 5.62-5.45 (m, 2H, major + minor), 5.09 (s, 1H, minor), 4.98 (s, 1H, major), 3.65 (s, 6H, major + minor), 3.18-3.04 (m, 2H, major + minor), 2.67-2.53 (m, 2H, major + minor). ^{13}C NMR (100 MHz, CDCl$_3$) δ 207.4, 207.2, 192.4, 192.2, 169.7, 169.7, 141.5, 141.0, 135.7, 135.6, 133.9, 133.8, 133.2, 133.0, 130.8, 130.8, 130.5, 130.4, 129.2, 129.0, 128.9, 128.8, 128.6, 128.5, 128.5, 128.4, 127.3, 127.2, 127.1, 127.0, 126.9, 126.8, 125.5, 125.4, 95.9, 95.5, 90.0,

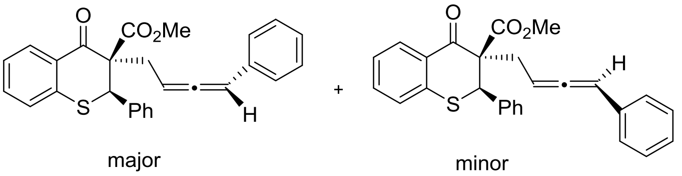

major minor

89.9, 62.9, 62.7, 52.4, 52.3, 51.1, 50.9, 32.5, 32.3.

(-)-Methyl 4-oxo-3-(4-phenylbuta-2,3-dien-1-yl)-2-(*o*-tolyl)thiochromane-3-carboxylate (3ea): The reaction was conducted at -20 °C for 120 h. 71.9 mg, 82% yield, pale yellow viscous liquid, new compound, R_f = 0.36 (hexanes/ethyl acetate 20/1), 24.0:1 dr, 98% ee (major diastereoisomer),

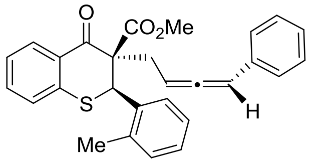

$[\alpha]^{20}_{D}$ = -25.30 (*c* 0.83, $CHCl_3$). The major diastereoisomer: ^{1}H NMR (400 MHz, $CDCl_3$) δ 8.20 (dd, *J* = 8.0, 1.3 Hz, 1H), 7.52 (d, *J* = 7.7 Hz, 1H), 7.42-7.34 (m, 1H), 7.29-7.09 (m, 10H), 6.00-5.85 (m, 1H), 5.65-5.43 (m, 1H), 5.04 (s, 1H), 3.62 (s, 3H), 3.15-3.00 (m, 1H), 2.84-2.69 (m, 1H), 2.41 (s, 3H). ^{13}C NMR (100 MHz, $CDCl_3$) δ 207.0, 192.1, 170.1, 141.3, 136.4, 134.9, 134.1, 133.2, 130.9, 130.8, 130.5, 128.5, 128.5, 128.1, 127.1, 127.0, 126.9, 126.4, 125.4, 94.9, 89.9, 62.0, 52.5, 47.0, 32.8, 20.3. HPLC: Chiralpak ID column, 254 nm, 30 °C, *n*-Hexane/*i*-PrOH = 97/3, flow = 1.0 mL/min, retention time 13.9 min (major) and 16.6 min. HRMS Calculated for $C_{28}H_{24}NaO_3S$ $[M+Na]^+$ 463.1338, found: 463.1341.

(-)-Methyl 4-oxo-3-(4-phenylbuta-2,3-dien-1-yl)-2-(*m*-tolyl)thiochromane-3-carboxylate(3fa): The reaction was conducted at -20 °C for 120 h. 78.1 mg, 89% yield, pale yellow viscous liquid, new compound, R_f = 0.36 (hexanes/ethyl acetate 20/1), 32.3:1 dr, 99% ee (major diastereoisomer),

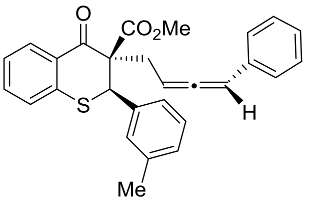

$[\alpha]^{20}_{D}$ = -241.32 (*c* 0.90, $CHCl_3$). The major diastereoisomer: ^{1}H NMR (400 MHz, $CDCl_3$) δ 8.24-8.10 (m, 1H), 7.42-7.37 (m, 1H), 7.27-7.13 (m, 10H), 6.19-6.00 (m, 1H), 5.60-5.41 (m, 1H), 4.97 (s, 1H), 3.65 (s, 3H), 3.22-3.03 (m, 1H), 2.69-2.52 (m, 1H), 2.33 (s, 3H). ^{13}C NMR (100 MHz, $CDCl_3$) δ 207.5, 192.5, 169.7, 141.7, 138.1, 135.3, 134.0, 133.2, 130.8, 130.5, 130.1, 129.7, 128.6, 128.4, 127.3, 127.1, 126.8, 126.1, 125.5, 95.5, 90.0, 62.7, 52.3, 51.1, 32.4, 21.5. HPLC: Chiralcel OD-3 column, 254 nm, 30 °C, *n*-Hexane/*i*-PrOH = 95/5, flow = 0.8 mL/min, retention time 10.2 min and 23.6 min (major). HRMS Calculated for $C_{28}H_{24}NaO_3S$ $[M+Na]^+$ 463.1338, found: 463.1342.

(-)-Methyl 4-oxo-3-(4-phenylbuta-2,3-dien-1-yl)-2-(*p*-tolyl)thiochromane-3-carboxylate(3ga): The reaction was conducted at -20 °C for 120 h. 79.3 mg, 90% yield, pale yellow viscous liquid, new compound, R_f = 0.36 (hexanes/ethyl acetate 20/1), 32.3:1 dr, 99% ee (major diastereoisomer),

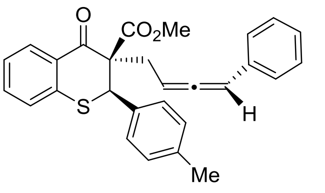

$[\alpha]^{20}_{D}$ = -226.76 (*c* 0.93, $CHCl_3$). The major diastereoisomer: ^{1}H NMR (400 MHz, $CDCl_3$) δ 8.31-8.04 (m, 1H), 7.43-7.37 (m, 1H), 7.27-7.22 (m, 6H), 7.20-7.11 (m, 5H), 6.21-6.00 (m, 1H), 5.61-5.41 (m, 1H), 4.96 (s, 1H), 3.65 (s, 3H), 3.16-3.03 (m, 1H), 2.64-2.55 (m, 1H), 2.34 (s, 3H). ^{13}C NMR (100 MHz, $CDCl_3$) δ 207.4, 192.6, 169.8, 141.7, 138.8, 134.0, 133.2, 132.4, 130.9, 130.5, 129.2, 129.0, 128.6, 127.3, 127.0, 126.9, 125.5, 95.5, 89.9, 62.8, 52.4, 50.9, 32.4, 21.2. HPLC: Chiralpak IA, 254 nm, 30 °C, *n*-Hexane/*i*-PrOH = 95/5, flow = 1.0 mL/ min, retention time 13.6 min and 17.4 min (major). HRMS Calculated for $C_{28}H_{24}NaO_3S$ $[M+Na]^+$ 463.1338, found: 463.1340.

(-)-Methyl 2-(4-fluorophenyl)-4-oxo-3-(4-phenylbuta-2,3-dien-1-yl)thiochromane-3-carboxylate (3ha): The reaction was conducted at -20 °C for 120 hours. 83.4 mg, 94% yield, pale yellow

viscous liquid, new compound, R_f = 0.40 (hexanes/ethyl acetate 20/1), 49.0:1 dr, 99% ee (major diastereoisomer), $[\alpha]^{20}_D$ = -215.33 (*c* 0.73, $CHCl_3$). The major diastereoisomer: ^{1}H NMR (400 MHz, $CDCl_3$) δ 8.17 (dd, *J* = 8.0, 1.2 Hz, 1H), 7.44-7.34 (m, 3H), 7.27-7.22 (m, 4H), 7.20-7.12 (m, 3H), 7.07-6.96 (m, 2H), 6.18-5.97 (m, 1H), 5.63-5.41 (m, 1H), 4.95 (s, 1H), 3.65 (s, 3H), 3.17- 3.04 (m, 1H), 2.63-2.50 (m, 1H). ^{13}C NMR (100 MHz, $CDCl_3$) δ 207.4, 192.1, 169.7, 162.8 (d, $^1J_{F\text{-}C}$ = 248.6 Hz), 141.2, 133.8, 133.3, 131.4 (d, $^4J_{F\text{-}C}$ = 3.4 Hz), 131.0, 131.0, 130.8, 130.5, 128.6, 127.2 (d, $^3J_{F\text{-}C}$ = 8.9 Hz), 126.8, 125.7, 115.5 (d, $^2J_{F\text{-}C}$ = 21.6 Hz), 95.6, 89.8, 62.6, 52.4, 50.3, 32.4. ^{19}F NMR (376 MHz, $CDCl_3$) δ -112.38. HPLC: Chiralcel OD-H column, 254 nm, 30 °C, *n*-Hexane/*i*-PrOH = 95/5, flow = 1.0 mL/min, retention time 9.6 min and 15.7 min (major). HRMS Calculated for $C_{27}H_{21}FNaO_3S$ $[M+Na]^+$ 467.1088, found: 467.1087.

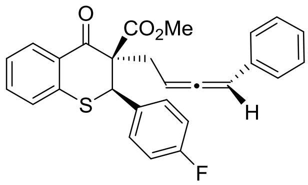

(-)-Methyl 2-(4-chlorophenyl)-4-oxo-3-(4-phenylbuta-2,3-dien-1-yl)thiochromane-3-carboxylate (3ia): The reaction was conducted at -20 °C for 120 hours. 88.3 mg, 96% yield, pale yellow viscous liquid, new compound, R_f = 0.40 (hexanes/ethyl acetate 20/1), 49.0:1 dr, 99% ee (major diastereoisomer), $[\alpha]^{20}_D$ = -200.57 (*c* 1.03, $CHCl_3$). The major diastereoisomer: ^{1}H NMR (400 MHz, $CDCl_3$) δ 8.17 (d, *J* = 7.9 Hz, 1H), 7.44-7.38 (m, 1H), 7.35-7.28 (m, 4H), 7.27-7.21 (m, 4H), 7.19-7.13 (m, 3H), 6.19-5.99 (m, 1H), 5.60-5.40 (m, 1H), 4.93 (s, 1H), 3.64 (s, 3H), 3.17-3.04 (m, 1H), 2.64-2.53 (m, 1H). ^{13}C NMR (100 MHz, $CDCl_3$) δ 207.4, 192.0, 169.6, 141.0, 134.8, 134.1, 133.8, 133.4, 130.8, 130.6, 130.5, 128.7, 128.6, 127.3, 127.2, 126.9, 125.7, 95.6, 89.8, 62.5, 52.5, 50.5, 32.5. HPLC: Chiralcel OD-H column, 254 nm, 30 °C, *n*-Hexane/*i*-PrOH = 95/5, flow = 1.0 mL/min, retention time 10.2 min and 16.5 min (major). HRMS Calculated for $C_{27}H_{21}ClNaO_3S$ $[M+Na]^+$ 483.0792 (^{35}Cl) and 485.0773 (^{37}Cl), found: 483.0793 (^{35}Cl) and 485.0792 (^{37}Cl).

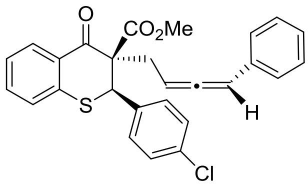

(-)-Methyl 2-(4-bromophenyl)-4-oxo-3-(4-phenylbuta-2,3-dien-1-yl)thiochromane-3-carboxylate (3ja): The reaction was conducted at -20 °C for 120 hours. 94.5 mg, 93% yield, pale yellow viscous liquid, new compound, R_f = 0.40 (hexanes/ethyl acetate 20/1), 49.0:1 dr, 99% ee (major diastereoisomer), $[\alpha]^{20}_D$ = -186.71 (*c* 1.10, $CHCl_3$). The major diastereoisomer: ^{1}H NMR (400 MHz, $CDCl_3$) δ 8.17 (dd, *J* = 8.0, 1.2 Hz, 1H), 7.51-7.35 (m, 3H), 7.28-7.21 (m, 6H), 7.19-7.13 (m, 3H), 6.14-6.00 (m, 1H), 5.57-5.44 (m, 1H), 4.91 (s, 1H), 3.64 (s, 3H), 3.17-3.04 (m, 1H), 2.62-2.52 (m, 1H). ^{13}C NMR (100 MHz, $CDCl_3$) δ 207.4, 192.0, 169.6, 141.0, 134.6, 133.8, 133.4, 131.7, 130.9, 130.7, 130.5, 128.6, 127.3, 127.2, 126.9, 125.7, 123.0, 95.6, 89.8, 62.5, 52.5, 50.5, 32.5. HPLC: Chiralcel OD-H column, 254 nm, 30 °C, *n*-Hexane/*i*-PrOH = 95/5, flow = 1.0 mL/min, retention time 11.1 min (minor) and 17.2 min (major). HRMS Calculated for $C_{27}H_{21}BrNaO_3S$ $[M+Na]^+$ 527.0287 (^{79}Br) and 529.0270 (^{81}Br), found: 527.0292 (^{79}Br) and 529.0275 (^{81}Br).

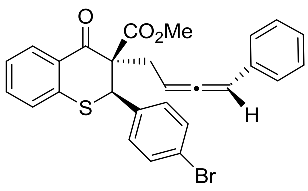

(-)-Methyl 2-(4-methoxyphenyl)-4-oxo-3-(4-phenylbuta-2,3-dien-1-yl)thiochromane-3-carboxylate (3ka): The reaction was conducted at -20 °C for 72 h. 76.9 mg, 84% yield, pale yellow viscous liquid, new compound, R_f = 0.23 (hexanes/ethyl acetate 20/1), 32.3:1 dr, 99% ee (major

diastereoisomer), $[\alpha]^{20}_{D}$ = -208.12 (*c* 1.07, $CHCl_3$). The major diastereoisomer: ^{1}H NMR (400 MHz, $CDCl_3$) δ 8.21-8.09 (m, 1H), 7.43-7.34 (m, 1H), 7.33-7.27 (m, 2H), 7.26-7.20 (m, 4H),

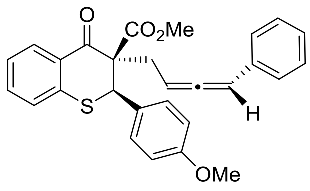

7.19-7.13 (m, 3H), 6.89-6.81 (m, 2H), 6.15-6.00 (m, 1H), 5.59-5.43 (m, 1H), 4.93 (s, 1H), 3.79 (s, 3H), 3.65 (s, 3H), 3.15-3.01 (m, 1H), 2.65-2.52 (m, 1H). ^{13}C NMR (100 MHz, $CDCl_3$) δ 207.4, 192.6, 169.8, 159.8, 141.8, 134.0, 133.2, 130.8, 130.5, 130.4, 128.6, 127.4, 127.2, 127.0, 126.9, 125.5, 113.8, 95.4, 89.9, 62.8, 55.3, 52.4, 50.5, 32.4. HPLC: Chiralcel OD-H column, 254 nm, 30 ^{o}C, *n*-Hexane/*i*-PrOH = 94/6, flow = 1.0 mL/ min, retention time 13.3 min (minor) and 26.0 min (major). HRMS Calculated for $C_{28}H_{24}NaO_4S$ $[M+Na]^+$ 479.1288, found: 479.1291.

(-)-Methyl 2-methyl-4-oxo-3-(4-phenylbuta-2,3-dien-1-yl)thiochromane-3-carboxylate (3la): The reaction was conducted at -20 ^{o}C for 72 h. 68.2 mg, 94% yield, pale yellow viscous liquid, new compound, R_f = 0.45 (hexanes/ethyl acetate 20/1), 9.0:1 dr, 99% ee (major diastereoisomer),

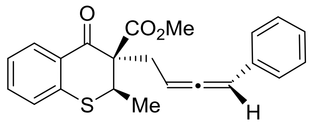

$[\alpha]^{20}_{D}$ = -87.47 (*c* 0.95, $CHCl_3$). The major diastereoisomer: ^{1}H NMR (400 MHz, $CDCl_3$) δ 8.16 (d, *J* = 7.6 Hz, 1H), 7.41-7.34 (m, 1H), 7.30-7.14 (m, 7H), 6.29-5.97 (m, 1H), 5.65-5.35 (m, 1H), 3.84 (q, *J* = 6.9 Hz, 1H), 3.63 (s, 3H), 3.39-3.24 (m, 1H), 2.92-2.77 (m, 1H), 1.61 (d, *J* = 7.0 Hz, 3H). ^{13}C NMR (100 MHz, $CDCl_3$) δ 207.1, 191.7, 170.2, 141.3, 134.0, 133.0, 130.7, 130.4, 128.6, 127.2, 127.1, 126.9, 125.3, 95.0, 89.3, 61.7, 52.4, 41.8, 31.1, 15.6. HPLC: Chiralpak IC column, 254 nm, 30 ^{o}C, *n*-Hexane/*i*-PrOH = 99/1, flow = 1.0 mL/min, retention time 19.7 min (major) and 22.0 min. HRMS Calculated for $C_{22}H_{21}O_3S$ $[M+H]^+$ 365.1206, found: 365.1209.

(-)-Methyl 6-nitro-4-oxo-2-phenyl-3-(4-phenylbuta-2,3-dien-1-yl)thiochromane-3-carboxyla-te (3ma): The reaction was conducted at -20 ^{o}C for 120 h. 86.1 mg, 91% yield, pale yellow viscous liquid, new compound, R_f = 0.18 (hexanes/ethyl acetate 20/1), 49.0:1 dr, 99% ee (major

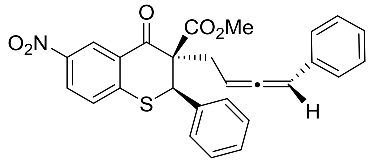

diastereoisomer), $[\alpha]^{20}_{D}$ = -207.17 (*c* 1.10, $CHCl_3$). The major diastereoisomer: ^{1}H NMR (400 MHz, $CDCl_3$) δ 8.94 (d, *J* = 2.5 Hz, 1H), 8.16 (dd, *J* = 8.7, 2.6 Hz, 1H), 7.40-7.32 (m, 6H), 7.24-7.19 (m, 2H), 7.18-7.08 (m, 3H), 6.15-5.99 (m, 1H), 5.62-5.48 (m, 1H), 5.07 (s, 1H), 3.66 (s, 3H), 3.22-3.07 (m, 1H), 2.63-2.52 (m, 1H). ^{13}C NMR (100 MHz, $CDCl_3$) δ 207.5, 190.5, 168.9, 149.7, 145.6, 134.2, 133.6, 130.9, 129.4, 129.2, 128.8, 128.6, 128.1, 127.2, 126.7, 126.7, 125.7, 96.2, 89.3, 62.1, 52.6, 51.0, 32.0. HPLC: Chiralpak IB column, 254 nm, 30 ^{o}C, *n*-Hexane/*i*-PrOH = 85/15, flow = 1.0 mL/min, retention time 11.4 min and 18.6 min (major). HRMS Calculated for $C_{27}H_{22}NO_5S$ $[M+H]^+$ 472.1213, found: 472.1218.

(-)-Methyl 6-methoxy-4-oxo-2-phenyl-3-(4-phenylbuta-2,3-dien-1-yl)thiochromane-3-carbo-xylate (3na): The reaction was conducted at -20 ^{o}C for 120 h. 78.8 mg, 86% yield, yellow viscous liquid, new compound, R_f = 0.21 (hexanes/ethyl acetate 20/1), 32.3:1 dr, 99% ee (major diastereoisomer), $[\alpha]^{20}_{D}$ = -234.07 (*c* 0.93, $CHCl_3$). The major diastereoisomer: ^{1}H NMR (400 MHz, $CDCl_3$) δ 7.65 (d, *J* = 2.9 Hz, 1H), 7.39-7.29 (m, 5H), 7.25- 7.21 (m, 2H), 7.19-7.09 (m, 4H), 7.05-6.98 (m, 1H), 6.15-6.04 (m, 1H), 5.59-5.43 (m, 1H), 4.96 (s, 1H), 3.84 (s, 3H), 3.65 (s, 3H), 3.19-3.06 (m, 1H), 2.66-2.54 (m, 1H). ^{13}C NMR (100 MHz, $CDCl_3$) δ 207.4, 192.5, 169.8,

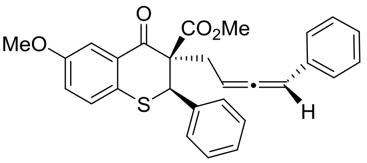

157.8, 135.7, 133.9, 133.0, 131.6, 129.1, 128.8, 128.6, 128.6, 128.5, 127.1, 126.9, 122.3, 112.6, 95.5, 89.9, 62.8, 55.6, 52.4, 51.5, 32.5. HPLC: Chiral- pak AD-3, 254 nm, 30 ^{o}C, *n*-Hexane/*i*-PrOH = 90/10, flow = 0.8 mL/min, retention time 18.2 min and 19.4 min (major). HRMS Calculated for $C_{28}H_{25}O_4S$ $[M+ H]^+$ 457.1468, found: 457.1466.

(-)-Methyl 6-methyl-4-oxo-2-phenyl-3-(4-phenylbuta-2,3-dien-1-yl)thiochromane-3-carboxy-late (3oa): The reaction was conducted at -20 ^{o}C for 120 hours. 82.9 mg, 94% yield, pale yellow viscous liquid, new compound, R_f = 0.31 (hexanes/ethyl acetate 20/1), 32.3:1 dr, 99% ee (major diastereoisomer), $[\alpha]^{20}_{D}$ = -225.21 (*c* 0.88, $CHCl_3$). The major diastereoisomer: ^{1}H NMR (400 MHz, $CDCl_3$) δ 8.00-7.94 (m, 1H), 7.39-7.30 (m, 5H), 7.26-7.21 (m, 3H), 7.20-7.11 (m, 4H), 6.15-6.02 (m, 1H), 5.59-5.45 (m, 1H), 4.96 (s, 1H), 3.64 (s, 3H), 3.17-3.06 (m, 1H), 2.64-2.55 (m, 1H), 2.36 (s, 3H). ^{13}C NMR (100 MHz, $CDCl_3$) δ 207.4, 192.7, 169.8, 138.2, 135.7, 135.5, 134.4, 134.0, 130.6, 129.1, 128.8, 128.5, 128.5, 127.2, 127.0, 126.9, 95.4 89.9, 62.8, 52.3, 51.3, 32.5, 21.0. HPLC: Chiralpak AD-H column, 254 nm, 30 ^{o}C, *n*-Hexane/*i*-PrOH = 96/4, flow = 1.0 mL/min, retention time 22.0 min and 25.0 min (major). HRMS Calculated for $C_{28}H_{25}O_3S$ $[M+H]^+$ 441.1519, found: 441.1516.

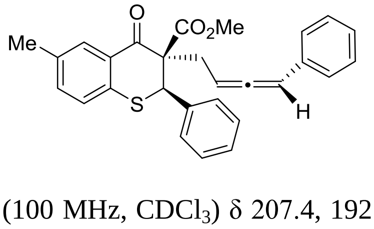

(-)-*t*-Butyl 4-oxo-2-phenyl-3-(4-phenylbuta-2,3-dien-1-yl)thiochromane-3-carboxylate (3pa): The reaction was conducted at -20 ^{o}C for 120 h. 88.7 mg, 95% yield, pale yellow viscous liquid, new compound, R_f = 0.40 (hexanes/ethyl acetate 20/1), 32.3:1 dr, 99% ee (major diastereoisomer), $[\alpha]^{20}_{D}$ = -182.51 (*c* 1.03, $CHCl_3$). The major diastereoisomer: ^{1}H NMR (400 MHz, $CDCl_3$) δ 8.18 (dd, *J* = 8.2, 1.5 Hz, 1H), 7.50-7.43 (m, 2H), 7.40-7.31 (m, 4H), 7.26-7.13 (m, 7H), 6.17-6.01 (m, 1H), 5.56-5.41 (m, 1H), 4.92 (s, 1H), 3.19-3.03 (m, 1H), 2.62-2.41 (m, 1H), 1.32 (s, 9H). ^{13}C NMR (100 MHz, $CDCl_3$) δ 207.4, 192.4, 168.1, 141.7, 135.5, 134.2, 132.8, 131.2, 130.3, 129.6, 128.8, 128.5, 128.4, 126.9, 126.9, 126.8, 125.2, 95.2, 90.1, 83.2, 62.2, 51.3, 32.0, 27.7. HPLC: Chiralpak AD-3 column, 254 nm, 30 ^{o}C, *n*-Hexane/*i*-PrOH = 96/4, flow = 0.8 mL/min, the retention time 11.2 min and 13.5 min (major). HRMS Calculated for $C_{30}H_{28}NaO_3S$ $[M+Na]^+$ 491.1651, found: 491.1656.

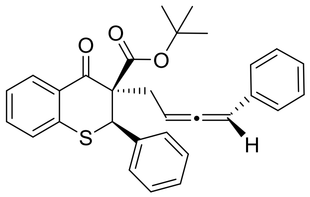

(+)-Methyl 2-(benzofuran-2-yl)-4-oxo-3-(-4-phenylbuta-2,3-dien-1-yl)thiochromane-3-carbo-xylate (3qa): The reaction was conducted at -20 ^{o}C for 72 h. 79.1 mg, 85% yield, pale yellow viscous liquid, new compound, R_f = 0.32 (hexanes/ethyl acetate 20/1), 5.7:1 dr, 97% ee (major diastereoisomer), $[\alpha]^{20}_{D}$ = +33.29 (*c* 0.82, $CHCl_3$). The major dias-tereoisomer: ^{1}H NMR (400 MHz, $CDCl_3$) δ 8.21 (dd, *J* = 8.0, 1.1 Hz, 1H), 7.53-7.47 (m, 1H), 7.43-7.37 (m, 2H), 7.28-7.16 (m, 9H), 6.75 (s, 1H), 6.14-6.01 (m, 1H), 5.65-5.52 (m, 1H), 5.14 (s, 1H), 3.65 (s, 3H), 3.28-3.16 (m, 1H), 2.97-2.85 (m, 1H). ^{13}C NMR (100 MHz, $CDCl_3$) δ 207.7, 190.9, 169.5, 154.3, 152.6, 139.9, 133.8, 133.5, 130.5, 130.4, 128.6, 128.0, 127.3, 127.1, 126.9, 125.8, 124.7, 123.0, 121.3, 111.2, 106.1, 95.3, 89.2, 61.3, 52.7, 44.6, 32.9. HPLC: Chiralpak AD-H column, 254 nm, 30 ^{o}C, *n*-Hexane/*i*-PrOH = 93/7, flow = 1.0 mL/min, the

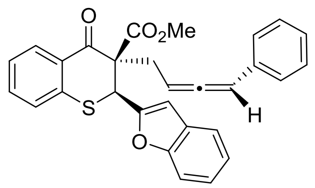

retention time 19.8 min and 22.3 min (major). HRMS Calculated for $C_{29}H_{23}O_4S$ $[M+H]^+$ 467.1312, found: 467.1320.

(+)-3-Acetyl-2-phenyl-3-(4-phenylbuta-2,3-dien-1-yl)thiochroman-4-one (3ba):
The reaction was conducted at 30 °C for 48 hours with commercially available (1*R*,1'*R*,2*S*,2'*S*)-DuanPhos as the chiral ligand. 49.8 mg, 61% yield, pale yellow viscous liquid, new compound, R_f = 0.35 (hexanes/ethyl acetate 20/1), 24.0:1 dr, 74% ee (major isomer), $[\alpha]^{20}_D$ = +0.69 (*c* 0.58, $CHCl_3$). The major diastereoisomer: ^1H NMR (400 MHz, $CDCl_3$) δ 8.18 (dd, *J* = 8.0, 1.2 Hz, 1H), 7.46- 7.41 (m, 1H), 7.30-7.23 (m, 9H), 7.21-7.14 (m, 3H), 6.18-6.03 (m, 1H), 5.48-5.37 (m, 1H), 4.86 (s, 1H), 3.24-3.13 (m, 1H), 2.80-2.70 (m, 1H), 2.11 (s, 3H). ^{13}C NMR (100 MHz, $CDCl_3$) δ 207.4, 205.2, 194.4, 140.8, 137.1, 133.9, 133.8, 130.9, 130.2, 128.8, 128.7, 128.6, 128.5, 127.4, 127.1, 126.8, 125.7, 95.5, 89.6, 67.1, 51.2, 33.7, 31.5. HPLC: Chiralcel OD-H column, 254 nm, 30 °C, *n*-Hexane/*i*-PrOH = 95/5, flow = 1.0 mL/min, retention time 9.4 min and 11.5 min (major). HRMS Calculated for $C_{27}H_{23}O_2S$ $[M+H]^+$ 411.1413, found: 411.1415. Notably, the absolute and relative configurations of this allenylic alkylation product were not further assigned.

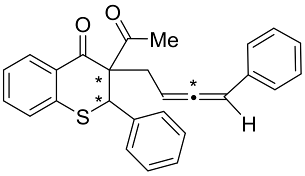

2-Phenyl-3-(4-phenylbuta-2,3-dien-1-yl)-3-(piperidine-1-carbonyl)thiochroman-4-one (3da):
The reaction was conducted at -20 °C for 168 h and the *rac*-**3da** was prepared with *rac*-BINAP at -20 °C. 32.3 mg, 34% yield, pale yellow viscous liquid, new compound, R_f = 0.52 (hexanes/ethyl acetate 5/1), 24.0:1 dr, >99% ee (major diastereoisomer), $[\alpha]^{20}_D$ = -49.90 (*c* 1.08, $CHCl_3$). The major diastereoisomer: ^1H NMR (400 MHz, $CDCl_3$) δ 8.09 (dd, *J* = 7.9, 1.2 Hz, 1H), 7.51-7.43 (m, 1H), 7.37-7.33 (m, 1H), 7.33-7.28 (m, 3H), 7.27-7.21 (m, 7H), 7.19-7.14 (m, 1H), 6.22-6.02 (m, 1H), 5.71-5.55 (m, 1H), 4.87 (s, 1H), 4.04-3.14 (m, 2H), 3.14-2.13 (m, 4H), 1.50-1.37 (m, 4H), 1.37-1.10 (m, 2H). ^{13}C NMR (100 MHz, $CDCl_3$) δ 207.7, 192.6, 167.3, 140.5, 137.9, 134.3, 133.7, 131.8, 130.1, 129.1, 128.6, 128.5, 128.3, 127.8, 126.9, 126.9, 126.1, 94.6, 91.0, 63.0, 52.1, 38.5, 24.9, 24.3. HPLC: Chiralpak IB column, 254 nm, 30 °C, *n*-Hexane/*i*-PrOH = 94/6, flow = 1.0 mL/min, retention time 13.1 min and 20.6 min (major). HRMS Calculated for $C_{31}H_{30}NO_2S$ $[M+H]^+$ 480.1992, found: 480.2000.

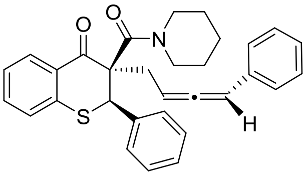

(-)-Methyl (2*R*,3*S*)-3-((*R*)-4-(naphthalen-2-yl)buta-2,3-dien-1-yl)-6-nitro-4-oxo-2-phenylthiochromane-3-carboxylate (3mk): The reaction was conducted at -20 °C for 168 h. 91.3 mg, 88% yield, pale yellow solid, mp 197-198 °C, new compound, R_f = 0.12 (hexanes/ethyl acetate 20/1), 32.3:1 dr, >99% ee (major diastereoisomer), $[\alpha]^{20}_D$ = -223.76 (*c* 1.14, $CHCl_3$). The major diaste- reoisomer: ^1H NMR (400 MHz, $CDCl_3$) δ 8.92 (d, *J* = 2.5 Hz, 1H), 8.03 (dd, *J* = 8.7, 2.5 Hz, 1H), 7.75-7.65 (m, 3H), 7.47 (s, 1H), 7.45-7.35 (m, 7H), 7.31 (dd, *J* = 8.5, 1.4 Hz, 1H), 7.24-7.21 (m, 1H), 6.33-6.18 (m, 1H), 5.70-5.55 (m, 1H), 5.11 (s, 1H), 3.65 (s, 3H), 3.28-3.11 (m, 1H), 2.66-2.50 (m, 1H). ^{13}C NMR (100 MHz, $CDCl_3$) δ 208.1, 190.5, 168.9, 149.6, 145.5, 134.2, 133.5, 132.6, 131.1, 130.8, 129.5, 129.2, 128.8, 128.3, 128.1, 127.7, 127.7, 126.5, 126.4, 125.9, 125.7, 125.6, 124.5, 96.6, 89.6, 62.2, 52.7, 51.0, 32.0. HPLC: Chiralpak IB column, 254 nm, 30 °C,

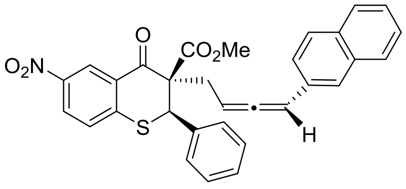

n-Hexane/*i*-PrOH = 80/20, flow = 1.0 mL/min, retention time 12.7 min (minor) and 22.5 min (major). HRMS Calculated for $C_{31}H_{24}NO_5S$ $[M+H]^+$ 522.1370, found: 522.1377.

Methyl 4-oxo-2-phenyl-3-(4-(*o*-tolyl)buta-2,3-dien-1-yl)thiochromane-3-carboxylate (3cb): The reaction was conducted at -20 °C for 72 h. 81.7 mg, 93% yield, pale yellow viscous liquid, new compound, R_f = 0.43 (hexanes/ethyl acetate 20/1), 32.3:1 dr, 99% ee (major diastereoisomer),

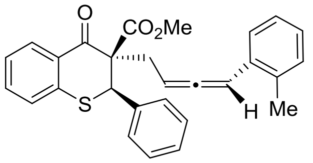

$[\alpha]^{20}_D$ = -193.32 (*c* 0.96, $CHCl_3$). The major diastereoisomer: ^1H NMR (400 MHz, $CDCl_3$) δ 8.17 (dd, *J* = 8.2, 1.4 Hz, 1H), 7.48-7.21 (m, 9H), 7.20-7.03 (m, 3H), 6.47-6.18 (m, 1H), 5.61-5.38 (m, 1H), 4.99 (s, 1H), 3.64 (s, 3H), 3.23-3.01 (m, 1H), 2.68-2.53 (m, 1H), 2.24 (s, 3H). ^{13}C NMR (100 MHz, $CDCl_3$) δ 208.0, 192.4, 169.7, 141.6, 135.6, 135.0, 133.3, 132.2, 130.8, 130.5, 130.4, 129.2, 128.9, 128.5, 127.5, 127.3, 127.0, 126.1, 125.6, 92.8, 88.9, 62.7, 52.4, 51.2, 32.5, 19.8. HPLC: Chiralcel OD-H column, 254 nm, 30 °C, *n*-Hexane/*i*-PrOH = 95/5, flow = 1.0 mL/min, retention time 10.1 min and 25.1 min (major). HRMS Calculated for $C_{28}H_{24}NaO_3S$ $[M+Na]^+$ 463.1338, found: 463.1342.

(-)-Methyl -oxo-2-phenyl-3-(4-(*m*-tolyl)buta-2,3-dien-1-yl)thiochromane-3-carboxylate(3cc): The reaction was conducted at -20 °C for 72 h. 86.1 mg, 98% yield, pale yellow viscous liquid, new compound, R_f = 0.43 (hexanes/ethyl acetate 20/1), 49.0:1 dr, 99% ee (major diastereoisomer),

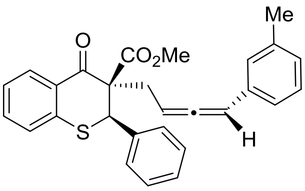

$[\alpha]^{20}_D$ = -213.13 (*c* 0.70, $CHCl_3$). The major diastereoisomer: ^1H NMR (400 MHz, $CDCl_3$) δ 8.23-8.11 (m, 1H), 7.44-7.31 (m, 6H), 7.26-7.22 (m, 2H), 7.16-7.10 (m, 1H), 7.01-6.95 (m, 3H), 6.16-5.97 (m, 1H), 5.59-5.42 (m, 1H), 4.99 (s, 1H), 3.65 (s, 3H), 3.19-3.07 (m, 1H), 2.65-2.55 (m, 1H), 2.28 (s, 3H). ^{13}C NMR (100 MHz, $CDCl_3$) δ 207.4, 192.4, 169.7, 141.5, 138.1, 135.6, 133.8, 133.2, 130.9, 130.5, 129.1, 128.9, 128.5, 128.5, 127.9, 127.5, 127.3, 125.5, 124.0, 95.6, 89.7, 62.7, 52.3, 51.1, 32.5, 21.3. HPLC: Chiralcel OD-H column, 254 nm, 30 °C, *n*-Hexane/*i*-PrOH = 95/5, flow = 1.0 mL/min, retention time 9.7 min and 21.8 min (major). HRMS Calculated for $C_{28}H_{24}NaO_3S$ $[M+Na]^+$ 463.1338, found: 463.1341.

(-)-Methyl 4-oxo-2-phenyl-3-(4-(*p*-tolyl)buta-2,3-dien-1-yl)thiochromane-3-carboxylate (3cd): The reaction was conducted at -20 °C for 72 h. 84.5 mg, 96% yield, pale yellow viscous liquid, new compound, R_f = 0.43 (hexanes/ethyl acetate 20/1), 32.3:1 dr, 99% ee (major diastereoisomer),

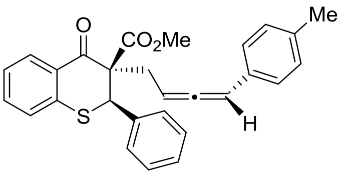

$[\alpha]^{20}_D$ = -193.85 (*c* 0.57, $CHCl_3$). The major diastereoisomer: ^1H NMR (400 MHz, $CDCl_3$) δ 8.22-8.11 (m, 1H), 7.43-7.31 (m, 6H), 7.26-7.22 (m, 2H), 7.12-7.01 (m, 4H), 6.19-5.94 (m, 1H), 5.59-5.40 (m, 1H), 4.99 (s, 1H), 3.65 (s, 3H), 3.19-3.04 (m, 1H), 2.66-2.54 (m, 1H), 2.30 (s, 3H). ^{13}C NMR (100 MHz, $CDCl_3$) δ 207.2, 192.5, 169.7, 141.5, 136.8, 135.6, 133.2, 130.9, 130.9, 130.5, 129.3, 129.1, 128.9, 128.5, 127.3, 126.7, 125.5, 95.4, 89.7, 62.7, 52.3, 51.1, 32.6, 21.2. HPLC: Chiralcel OD-H column, 254 nm, 30 °C, *n*-Hexane/*i*-PrOH = 95/5, flow = 1.0 mL/min, retention time 9.6 min and 18.8 min (major). HRMS Calculated for $C_{28}H_{24}NaO_3S$ $[M+Na]^+$ 463.1338, found: 463.1342.

(-)-Methyl 3-(4-(3-fluorophenyl)buta-2,3-dien-1-yl)-4-oxo-2-phenylthiochromane-3-carboxylate (3ce): The reaction was conducted at -20 °C for 120 hours. 83.7 mg, 94% yield, pale yellow viscous liquid, new compound, R_f = 0.38 (hexanes/ethyl acetate 20/1), 32.3:1 dr, 99% ee (major diastereoisomer), $[\alpha]^{20}_{D}$ = -179.28 (*c* 0.85, $CHCl_3$). The major diastereoisomer: ^1H NMR (400 MHz, $CDCl_3$) δ 8.20-8.13 (m, 1H), 7.43-7.32 (m, 6H), 7.27-7.17 (m, 3H), 6.98-6.92 (m, 1H), 6.91-6.80 (m, 2H), 6.16-5.95 (m, 1H), 5.69-5.48 (m, 1H), 4.94 (s, 1H), 3.65 (s, 3H), 3.20-3.02 (m, 1H), 2.69-2.47 (m, 1H). ^{13}C NMR (100 MHz, $CDCl_3$) δ 207.6, 192.3, 169.6, 163.1 (d, $^1J_{F\text{-}C}$ = 245.2 Hz), 141.5, 136.5 (d, $^3J_{F\text{-}C}$ = 7.7 Hz), 135.5, 133.3, 130.8, 130.5, 129.9 (d, $^3J_{F\text{-}C}$ = 8.3 Hz), 129.2, 128.9, 128.5, 127.2, 125.6, 122.6 (d, $^4J_{F\text{-}C}$ = 2.7 Hz), 113.9 (d, $^2J_{F\text{-}C}$ = 21.3 Hz), 113.4 (d, $^2J_{F\text{-}C}$ = 22.4 Hz), 94.8 (d, $^4J_{F\text{-}C}$ = 2.5 Hz), 90.4, 62.6, 52.4, 51.3, 32.4. ^{19}F NMR (376 MHz, $CDCl_3$) δ -113.49. HPLC: Chiralcel OD-3 column, 254 nm, 30 °C, *n*-Hexane/*i*-PrOH = 95/5, flow = 0.8 mL/min, retention time 12.3 min and 35.0 min (major). HRMS Calculated for $C_{27}H_{21}FNaO_3S$ $[M+Na]^+$ 467.1088, found: 467.1084.

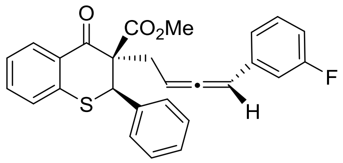

(-)-Methyl 3-(4-(3-chlorophenyl)buta-2,3-dien-1-yl)-4-oxo-2-phenylthiochromane-3-carboxylate (3cf): The reaction was conducted at -20 °C for 120 hours. 83.1 mg, 90% yield, pale yellow viscous liquid, new compound, R_f = 0.38 (hexanes/ethyl acetate 20/1), 32.3:1 dr, 99% ee (major diastereoisomer), $[\alpha]^{20}_{D}$ = -188.92 (*c* 0.84, $CHCl_3$). The major diastereoisomer: ^1H NMR (400 MHz, $CDCl_3$) δ 8.21-8.14 (m, 1H), 7.44- 7.32 (m, 6H), 7.27-7.23 (m, 2H), 7.20-7.10 (m, 3H), 7.08-7.01 (m, 1H), 6.16-5.92 (m, 1H), 5.67-5.51 (m, 1H), 4.93 (s, 1H), 3.65 (s, 3H), 3.17-3.06 (m, 1H), 2.65-2.55 (m, 1H). ^{13}C NMR (100 MHz, $CDCl_3$) δ 207.6, 192.3, 169.6, 141.5, 136.1, 135.5, 134.5, 133.3, 130.8, 130.5, 129.7, 129.2, 129.0, 128.5, 127.2, 127.0, 126.7, 125.6, 125.0, 94.6, 90.4, 62.6, 52.4, 51.3, 32.4. HPLC: Chiralcel OD-3 column, 254 nm, 30 °C, *n*-Hexane/*i*-PrOH = 95/5, flow = 0.8 mL/min, retention time 12.7 min (minor) and 37.3 min (major). HRMS Calculated for $C_{27}H_{22}ClO_3S$ $[M+H]^+$ 461.0973 (^{35}Cl) and 463.0953 (^{37}Cl), found: 461.0969 (^{35}Cl) and 463.0938 (^{37}Cl).

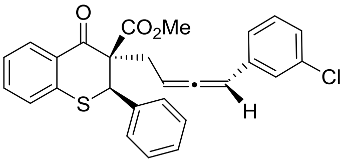

(-)-Methyl 3-(4-(3-bromophenyl)buta-2,3-dien-1-yl)-4-oxo-2-phenylthiochromane-3-carboxylate (3cg): The reaction was conducted at -20 °C for 120 hours. 94.4 mg, 93% yield, pale yellow viscous liquid, new compound, R_f = 0.38 (hexanes/ethyl acetate 20/1), 32.3:1 dr, 98% ee (major diastereoisomer), $[\alpha]^{20}_{D}$ = -173.55 (*c* 0.87, $CHCl_3$). The major diastereoisomer: ^1H NMR (400 MHz, $CDCl_3$) δ 8.18 (dd, *J* = 7.8, 1.0 Hz, 1H), 7.43-7.23 (m, 10H), 7.15-7.05 (m, 2H), 6.15-5.93 (m, 1H), 5.73-5.50 (m, 1H), 4.93 (s, 1H), 3.65 (s, 3H), 3.23-3.01 (m, 1H), 2.72-2.48 (m, 1H). ^{13}C NMR (100 MHz, $CDCl_3$) δ 207.6, 192.3, 169.6, 141.5, 136.3, 135.5, 133.3, 130.8, 130.5, 130.0, 129.9, 129.6, 129.2, 129.0, 128.6, 127.3, 125.6, 125.4, 122.8, 94.5, 90.5, 62.6, 52.4, 51.3, 32.4. HPLC: Chiralcel OD-3 column, 254 nm, 30 °C, *n*-Hexane/*i*-Pr-OH = 95/5, flow = 0.8 mL/min, retention time 13.1 min and 39.9 min (major). HRMS Calculated for $C_{27}H_{22}BrO_3S$ $[M+H]^+$ 505.0468 (^{79}Br) and 507.0450 (^{81}Br), found: 505.0471 (^{79}Br) and 507.0454 (^{81}Br).

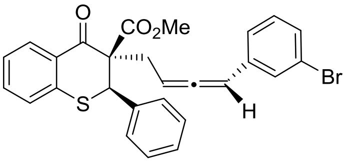

(-)-Methyl 3-(4-(3-methoxyphenyl)buta-2,3-dien-1-yl)-4-oxo-2-phenylthiochromane-3-carboxylate (3ch): The reaction was conducted at -20 °C for 72 hours. 84.6 mg, 93% yield, pale yellow viscous liquid, new compound, R_f = 0.20 (hexanes/ethyl acetate 20/1), 32.3:1 dr, 99% ee (major diastereoisomer), $[\alpha]^{20}_D$ = -192.34 (*c* 1.02, $CHCl_3$). The major diastereoisomer: ^1H NMR (400 MHz, $CDCl_3$) δ 8.27-8.04 (m, 1H), 7.42-7.29 (m, 6H), 7.25-7.21 (m, 2H), 7.17-7.12 (m, 1H), 6.80-6.67 (m, 3H), 6.11-5.99 (m, 1H), 5.61-5.42 (m, 1H), 4.98 (s, 1H), 3.76 (s, 3H), 3.64 (s, 3H), 3.17- 3.05 (m, 1H), 2.65-2.54 (m, 1H). ^{13}C NMR (100 MHz, $CDCl_3$) δ 207.4, 192.4, 169.7, 159.9, 141.5, 135.6, 135.4, 133.2, 130.8, 130.5, 129.5, 129.2, 128.9, 128.5, 127.3, 125.5, 119.5, 113.1, 111.9, 95.6, 90.0, 62.7, 55.2, 52.3, 51.2, 32.4. HPLC: Chiralcel OD-3 column, 254 nm, 30 °C, *n*-Hexane/*i*-PrOH = 94/6, flow = 0.8 mL/ min, retention time 14.7 min and 32.0 min (major). HRMS Calculated for $C_{28}H_{25}O_4S$ $[M+H]^+$ 457.1468, found: 457.1473.

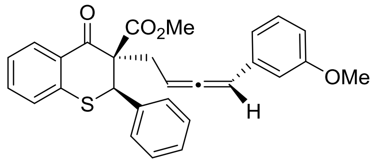

(-)-Methyl 3-(4-(3,5-dimethylphenyl)buta-2,3-dien-1-yl)-4-oxo-2-phenylthiochromane-3-carboxylate (3ci): The reaction was conducted at -20 °C for 120 h. 89.1 mg, 98% yield, pale yellow viscous liquid, new compound, R_f = 0.33 (hexanes/ethyl acetate 20/1), 49.0:1 dr, 99% ee (major diastereoisomer), $[\alpha]^{20}_D$ = -195.93 (*c* 0.74, $CHCl_3$). The major diastereoisomer: ^1H NMR (400 MHz, $CDCl_3$) δ 8.25-8.08 (m, 1H), 7.43-7.29 (m, 6H), 7.26-7.22 (m, 2H), 6.87-6.72 (m, 3H), 6.13-5.92 (m, 1H), 5.57-5.39 (m, 1H), 5.00 (s, 1H), 3.65 (s, 3H), 3.25-3.06 (m, 1H), 2.66-2.53 (m, 1H), 2.24 (s, 6H). ^{13}C NMR (100 MHz, $CDCl_3$) δ 207.5, 192.4, 169.8, 141.5, 138.0, 135.6, 133.7, 133.2, 130.9, 130.5, 129.1, 128.8, 128.5, 127.3, 125.5, 124.7, 95.7, 89.5, 62.7, 52.3, 51.0, 32.5, 21.2. HPLC: Chiralcel OD-H column, 254 nm, 30 °C, *n*-Hexane/*i*-PrOH = 96/4, flow = 1.0 mL/min, retention time 9.3 min and 20.1 min (major). HRMS Calculated for $C_{29}H_{27}O_3S$ $[M+H]^+$ 455.1675, found: 455.1682.

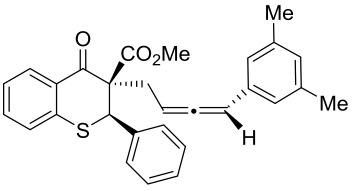

(-)-Methyl 3-(4-([1,1'-biphenyl]-4-yl)buta-2,3-dien-1-yl)-4-oxo-2-phenylthiochromane-3-carboxylate (3cj): The reaction was conducted at -20 °C for 120 h. 96.2 mg, 96% yield, pale yellow viscous liquid, new compound, R_f = 0.31 (hexanes/ethyl acetate 20/1), 49.0:1 dr, 99% ee (major diastereoisomer), $[\alpha]^{20}_D$ = -186.99 (*c* 1.10, $CHCl_3$). The major diastereoisomer: ^1H NMR (400 MHz, $CDCl_3$) δ 8.22-8.12 (m, 1H), 7.58-7.53 (m, 2H), 7.50-7.45 (m, 2H), 7.43-7.30 (m, 9H), 7.26-7.22 (m, 4H), 6.22-6.04 (m, 1H), 5.66-5.48 (m, 1H), 5.00 (s, 1H), 3.65 (s, 3H), 3.21-3.03 (m, 1H), 2.69-2.56 (m, 1H). ^{13}C NMR (100 MHz, $CDCl_3$) δ 207.6, 192.4, 169.7, 141.5, 140.8, 139.9, 135.6, 133.3, 133.0, 130.8, 130.5, 129.2, 128.9, 128.8, 128.5, 127.3, 126.9, 125.6, 95.2, 90.0, 62.7, 52.4, 51.2, 32.5. HPLC: Chiralpak ID + IC column, 254 nm, 30 °C, *n*-Hexane/*i*-PrOH = 94/6, flow = 1.0 mL/min, retention time 43.1 min (major) and 47.4 min (minor). HRMS Calculated for $C_{33}H_{27}O_3S$ $[M+H]^+$ 503.1675, found: 503.1673.

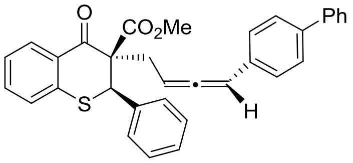

(-)-Methyl 3-(4-(naphthalen-2-yl)buta-2,3-dien-1-yl)-4-oxo-2-phenylthiochromane-3-carboxylate (3ck): The reaction was conducted at -20 °C for 120 h. 90.4 mg, 95% yield, pale yellow viscous liquid, new compound, R_f = 0.30 (hexanes/ethyl acetate 20/1), 24.0:1 dr, 99% ee (major diastereoisomer), $[\alpha]^{20}_D$ = -160.81 (*c* 1.10, $CHCl_3$). The major diastereoisomer:

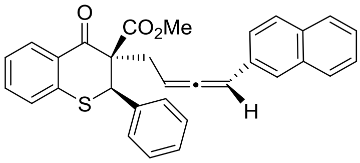

^1H NMR (400 MHz, $CDCl_3$) δ 8.18 (dd, *J* = 7.9, 1.1 Hz, 1H), 7.76-7.67 (m, 3H), 7.55-7.52 (m, 1H), 7.45-7.31 (m, 9H), 7.24-7.19 (m, 2H), 6.35-6.16 (m, 1H), 5.69-5.50 (m, 1H), 5.01 (s, 1H), 3.64 (s, 3H), 3.23-3.07 (m, 1H), 2.70-2.59 (m, 1H). ^{13}C NMR (100 MHz, $CDCl_3$) δ 208.0, 192.4, 169.7, 141.5, 135.6, 133.6, 133.2, 132.7, 131.5, 130.8, 130.5, 129.2, 128.9, 128.5, 128.2, 127.7, 127.7, 127.3, 126.2, 125.7, 125.6, 124.8, 95.9, 90.2, 62.8, 52.4, 51.2, 32.6. HPLC: Chiralpak IC column, 254 nm, 30 °C, *n*-Hexane/*i*-PrOH = 94/6, flow = 1.0 mL/min, retention time 20.5 min (minor) and 22.8 min (major). HRMS Calculated for $C_{31}H_{28}NO_3S$ $[M+NH_4]^+$ 494.1784, found: 494.1777.

(-)-Methyl 4-oxo-2-phenyl-3-(4-(thiophen-3-yl)buta-2,3-dien-1-yl)thiochromane-3-carboxylate (3cl): The reaction was conducted at -20 °C for 120 h. 78.2 mg, 90% yield, pale yellow viscous liquid, new compound, R_f = 0.32 (hexanes/ethyl acetate 20/1), 32.3:1 dr, 98% ee (major diastereoisomer), $[\alpha]^{20}_D$ = -187.60 (*c* 0.92, $CHCl_3$). The major diastereoisomer:

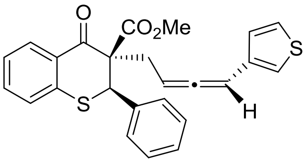

^1H NMR (400 MHz, $CDCl_3$) δ 8.22-8.13 (m, 1H), 7.42-7.32 (m, 6H), 7.25-7.18 (m, 3H), 6.99-6.91 (m, 2H), 6.31-5.97 (m, 1H), 5.52-5.41 (m, 1H), 4.97 (s, 1H), 3.64 (s, 3H), 3.15-3.05 (m, 1H), 2.63-2.54 (m, 1H). ^{13}C NMR (100 MHz, $CDCl_3$) δ 207.6, 192.4, 169.7, 141.5, 135.6, 135.1, 133.2, 130.8, 130.5, 129.2, 128.9, 128.5, 127.3, 126.3, 125.9, 125.5, 121.0, 90.1, 89.1, 62.7, 52.3, 51.1, 32.6. HPLC: Chiralcel OD-3 column, 254 nm, 30 °C, *n*-Hexane /*i*-PrOH = 93/7, flow = 0.8 mL/min, retention time 11.9 min (minor) and 25.4 min (major). HRMS Calculated for $C_{25}H_{24}NO_3S_2$ $[M+NH_4]^+$ 450.1192, found: 450.1182.

(-)-Methyl 4-oxo-3-(penta-2,3-dien-1-yl)-2-phenylthiochromane-3-carboxylate (3cm): The reaction was conducted at -20 °C for 120 h. 39.3 mg, 54% yield, pale yellow viscous liquid, new compound, R_f = 0.39 (hexanes/ethyl acetate 20/1), 7.3:1 dr, 97% ee (major diastereoisomer), $[\alpha]^{20}_D$ = -173.75 (*c* 0.85, $CHCl_3$). The major diastereoisomer:

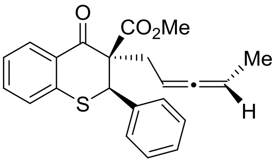

^1H NMR (400 MHz, $CDCl_3$) δ 8.29-8.02 (m, 1H), 7.44-7.31 (m, 6H), 7.28-7.22 (m, 2H), 5.08-4.92 (m, 3H), 3.66 (s, 3H), 3.06-2.95 (m, 1H), 2.52-2.40 (m, 1H), 1.55-1.48 (m, 3H). ^{13}C NMR (100 MHz, $CDCl_3$) δ 207.0, 192.6, 169.8, 141.4, 135.7, 133.1, 131.0, 130.4, 129.1, 128.8, 128.4, 127.3, 125.5, 86.8, 85.2, 63.0, 52.3, 50.7, 32.6, 14.1. HPLC: Chiralpak AD-3 column, 254 nm, 30 °C, *n*-Hexane/*i*-PrOH = 97/3, flow = 0.8 mL/min, retention time 18.8 min and 19.8 min (major). HRMS Calculated for $C_{22}H_{21}O_3S$ $[M+H]^+$ 365.1206, found: 365.1208.

(-)-Methyl 4-oxo-2-phenyl-3-(4-phenylpenta-2,3-dien-1-yl)thiochromane-3-carboxylate (3cn): The reaction was conducted at 30 °C for 120 h. 63.7 mg, 72% yield, pale yellow viscous liquid, new compound, R_f = 0.41 (hexanes/ethyl acetate 20/1), 6.7:1 dr, 90% ee (major diastereoisomer),

$[\alpha]^{20}_{D}$ = -109.42 (*c* 0.88, $CHCl_3$). The major diastereoisomer: ^1H NMR (400 MHz, $CDCl_3$) δ 8.17 (dd, *J* = 7.9, 1.1 Hz, 1H), 7.41-7.37 (m, 1H), 7.36-7.29 (m, 5H), 7.28-7.12 (m, 7H), 5.45-5.32 (m, 1H), 5.01 (s, 1H), 3.62 (s, 3H), 3.22-3.10 (m, 1H), 2.65-2.50 (m, 1H), 1.99 (d, *J* = 2.9 Hz, 3H). ^{13}C NMR (100 MHz, $CDCl_3$) δ 206.8, 192.3, 169.8, 141.4, 136.7, 135.9, 133.2, 130.7, 130.4, 128.9, 128.8, 128.5, 128.3, 127.3, 126.9, 125.8, 125.4, 101.9, 87.7, 62.7, 52.3, 50.4, 32.7, 16.9. HPLC: Chiralpak AD-H column, 254 nm, 30 ^oC, *n*-Hexane/*i*-Pr- OH = 96/4, flow = 1.0 mL/min, retention time 14.0 min and 17.1 min (major). HRMS Calculated for $C_{28}H_{24}NaO_3S$ $[M+Na]^+$ 463.1338, found: 463.1346.

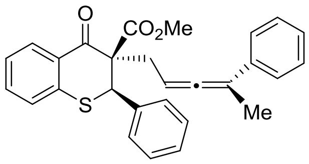

The Chemo- or Regioselective Isomers with the Different EWG

2-Benzylidene-1-(2-((1-phenylbuta-1,3-dien-2-yl)thio)phenyl)-3-(piperidin-1-yl)propane-1,3-dione (4'da): 46.1 mg, 48% yield, yellow viscous liquid, new compound, R_f = 0.18 (hexanes/ethyl acetate 5/1), 16.7/1 (*Z*/*E* or *E*/*Z*). The major diastereoisomer: ^1H NMR (400 MHz, $CDCl_3$ δ 7.56-7.42 (m, 4H), 7.41-7.26 (m, 10H), 7.09 (s, 1H), 7.00 (s, 1H), 6.83 (dd, *J* = 16.8, 10.5 Hz, 1H), 5.74 (d, *J* = 16.8 Hz, 1H), 5.28 (d, *J* = 10.6 Hz, 1H), 3.89-3.62 (m, 2H), 3.57-3.26 (m, 2H), 1.77-1.45 (m, 5H), 1.20-0.96 (m, 1H). ^{13}C NMR (100 MHz, $CDCl_3$) δ 194.7, 165.5, 143.8, 139.2, 137.2, 136.1, 134.4, 133.4, 133.2, 131.7, 131.2, 130.8, 130.6, 130.0, 129.4, 129.0, 128.9, 128.4, 127.9, 126.3, 120.1, 47.8, 42.4, 26.1, 25.3, 24.6. HRMS Calculated for $C_{31}H_{30}NO_2S$ $[M+H]^+$ 480.1992, found: 480.2001. (The Z/E configuration of carbon-carbon double bond is not further assigned)

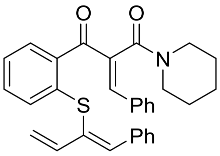

(-)-2-Nitro-3-phenyl-1-(2-((4-phenylbuta-2,3-dien-1-yl)thio)phenyl)prop-2-en-1-one (4aa): The reaction was conducted at 30 ^oC for 24 h with **L1**. 59.3 mg, 72% yield, pale yellow viscous liquid, new compound, R_f = 0.26 (hexanes/ethyl acetate 20/1), 84% ee, single isomer, $[\alpha]^{20}_{D}$ = -178.56 (*c* 0.63, $CHCl_3$). ^1H NMR (400 MHz, $CDCl_3$) δ 8.67-8.48 (m, 1H), 7.61-7.50 (m, 3H), 7.48-7.18 (m, 10H), 7.18-7.12 (m, 1H), 6.15-6.01 (m, 1H), 5.90-5.75 (m, 1H), 3.47-3.32 (m, 2H). ^{13}C NMR (100 MHz, $CDCl_3$) δ 205.8, 179.7, 149.9, 137.5, 136.3, 134.6, 132.8, 131.3, 130.5, 129.5, 129.2, 128.7, 128.6, 128.5, 127.4, 126.8, 126.7, 125.6, 95.6, 93.5, 28.6. HPLC: Chiralpak IA column, 254 nm, 30 ^oC, *n*-Hexane/*i*-PrOH = 94/6, flow = 1.0 mL/min, retention time 15.2 min and 16.2 min (major). HRMS Calculated for $C_{25}H_{19}KNO_3S$ $[M+K]^+$ 452.0717, found: 452.0731. (The absolute configuration of allene moiety is not further assigned)

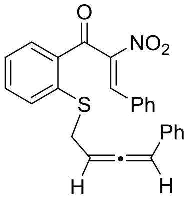

---

4. Scale-up Synthesis

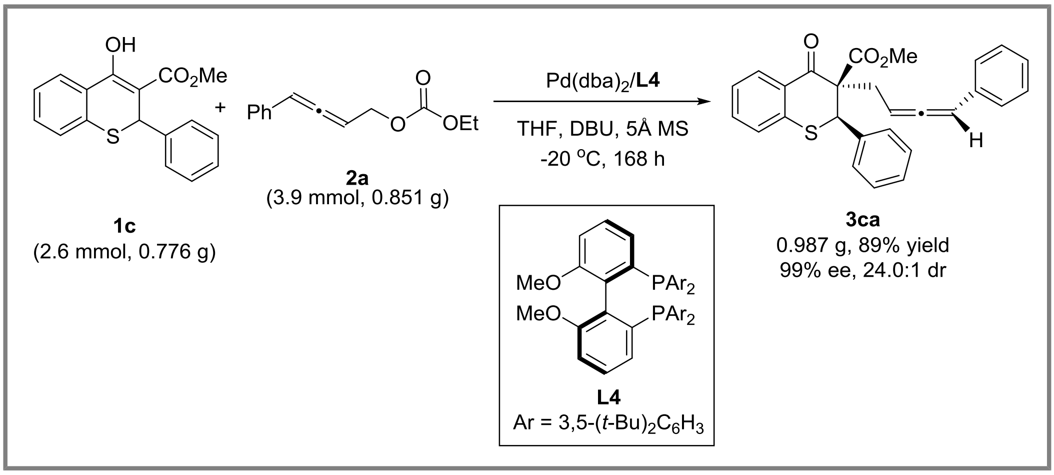

The metal precursor Pd(dba)$_2$ (0.26 mmol, 0.150 g), the chiral ligand (*R*)-DTB-BIPHEP (**L4**) (0.286 mmol, 0.295 g) and tetrahydrofuran (23 mL) were placed in a dried Schlenk tube under nitrogen atmosphere. The mixture was stirred at 30 ^{o}C for 30 min. Then, the mixture was cooled to -20 ^{o}C. Then, the substrate thiochromanone **1c** (2.6 mmol, 0.776 g), 5Å MS (0.650 g) and 1,8-diazabicyclo[5,4,0]undec-7-ene (DBU, 3.12 mmol, 0.47 mL) were added, and the reaction was stirred at -20 ^{o}C for 10 minutes. Sequentially, allenylic carbonate **2a** (3.9 mmol, 0.851 g) and tetrahydrofuran (3.0 mL) were added slowly. The mixture was stirred at -20 ^{o}C for 168 hours. After the completion of the reaction, the volatiles were directly removed under the reduced pressure. The crude residue was quickly purified by column chromatography on silica gel (hexanes/ethyl acetate /dichloromethane 100/1/2-100/3/6) to give the chiral allenylic alkylation product **3ca** 0.987 g, 89% isolated yield, 24.0:1 dr and 99% ee for the major diastereoisomer.

The racemate was prepared by running reaction with an achiral 1,3-bis(diphenylphosphino)-propane ligand at 30 ^{o}C. Notably, the product **3ca** was a little sensitive to water or alcohol, which would lead to slight decrease of dr value.

5. Product Elaborations

5.1. The Oxidation of Sulfide

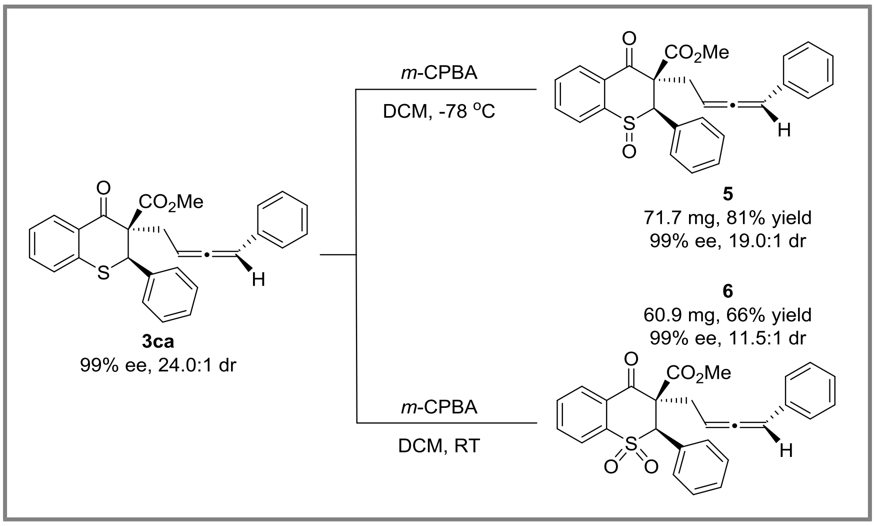

At -78 °C, to a solution of (-)-**3ca** (85.3 mg, 0.2 mmol, 99% ee) in dichloromethane (3.0 mL) was added 3-chloroperoxybenzoic acid (*m*-CPBA) (60.9 mg, 0.3 mmol, 85%) in dichloromethane (2.0 mL). The mixture was stirred at -78 °C for 48 hours. Then, to the reaction mixture was added a solution of *m*-CPBA (60.9 mg, 0.3 mmol, 85%) in dichloromethane (10 mL). The mixture was stirred at -78 °C for another 48 hours. After that, it was quenched with aqueous sodium bicarbonate and warmed to room temperature. The aqueous layer was extracted with dichloromethane. The combined organic layer was dried over anhydrous sodium sulfate, filtered and concentrated under the reduced pressure. The residue was purified by flash chromatography on silica gel using hexanes/ethyl acetate (5/1-3/1) as eluent to give the oxidative product sulfoxide **5**.

Methyl 4-oxo-2-phenyl-3-(4-phenylbuta-2,3-dien-1-yl)thiochromane-3-carboxylate 1-oxide (5): 71.7 mg, 81% yield, colorless viscous liquid, new compound, R_f = 0.23 (hexanes/ethyl acetate 3/1), 19.0:1 dr, 99% ee (major diastereoisomer), $[\alpha]^{20}_D$ = -357.57 (*c* 1.00, $CHCl_3$). The major diastereoisomer: ^1H NMR (400 MHz, $CDCl_3$) δ 8.10 (d, *J* = 7.6 Hz, 1H), 8.03 (d, *J* = 7.8 Hz, 1H), 7.81 (t, *J* = 7.3 Hz, 1H), 7.62 (t, *J* = 7.5 Hz, 1H), 7.45-7.34 (m, 5H), 7.25-7.06 (m, 5H), 6.17-6.01 (m, 1H), 5.38-5.26 (m, 1H), 4.82 (s, 1H), 3.76 (s, 3H), 3.44-3.29 (m, 1H), 2.51-2.37 (m, 1H). ^{13}C NMR (100 MHz, $CDCl_3$) δ 207.5, 190.8, 169.0, 147.6, 135.0, 133.3, 131.2, 129.9, 129.6, 129.6, 129.4, 129.1, 128.6, 127.3, 126.9, 96.1, 88.4, 70.5, 63.6, 53.1, 32.5. HPLC: Chiralpak AD-3 column, 254 nm, 30 °C, *n*-Hexane/*i*-PrOH = 70/30, flow = 0.7 mL/min, retention time 27.6 min (major) and 32.7 min. HRMS Calculated for $C_{27}H_{22}NaO_4S$ $[M+Na]^+$ 465.1131, found: 465.1139.

At room temperature, to a solution of (-)-**3ca** (85.3 mg, 0.2 mmol, 99% ee) in dichloromethane (3.0 mL) was added *m*-CPBA (121.8 mg, 0.6 mmol, 85%) in dichloromethane (2.0 mL). The mixture was stirred for 24 hours. Then, the reaction mixture was quenched with saturated sodium bicarbonate aqueous solution, and the aqueous layer was extracted three times with dichloromethane. The combined organic layer was dried over anhydrous sodium sulfate, filtered and concentrated under the reduced pressure. The residue was purified by flash chromatography on silica gel using hexanes/ethyl acetate (5/1) as eluent to give the oxidative product sulfone **6**.

(-)-Methyl 4-oxo-2-phenyl-3-(4-phenylbuta-2,3-dien-1-yl)thiochromane-3-carboxylate 1,1-dioxide (6): 60.9 mg, 66% yield, colorless viscous liquid, new compound, R_f = 0.51 (hexanes/ ethyl acetate 3/1), 11.5:1 dr, 99% ee (major diastereoisomer), $[\alpha]^{20}_D$ = -135.23 (*c* 1.05, $CHCl_3$). The major diastereoisomer: ^1H NMR (400 MHz, $CDCl_3$) δ 8.33-8.20 (m, 1H), 8.03-7.96 (m, 1H), 7.84-7.72 (m, 2H), 7.64-7.53 (m, 2H), 7.49-7.38 (m, 3H), 7.25-7.10 (m, 3H), 7.12-7.05 (m, 2H), 6.08-5.93 (m, 1H), 5.49-5.38 (m, 1H), 5.11 (s, 1H), 3.69 (s, 3H), 3.31-3.17 (m, 1H), 2.64-2.50 (m, 1H). ^{13}C NMR (100 MHz, $CDCl_3$) δ 207.4, 189.6, 169.0, 141.6, 134.7, 133.4, 133.2, 131.8, 130.7, 130.2, 129.4, 128.7, 128.7, 127.4, 126.8, 125.7, 123.9, 96.5, 88.8, 68.3, 63.1, 53.3, 33.9. HPLC: Chiralpak IC column, 254 nm, 30 °C, *n*-Hexane/*i*-PrOH = 65/35, flow = 0.8 mL/min, retention time 22.8 min and 40.9 min (major). HRMS Calculated for $C_{27}H_{22}NaO_5S$ $[M+Na]^+$ 481.1080, found: 481.1078.

5.2. The Hydrogenation of Allenyl Functional Group

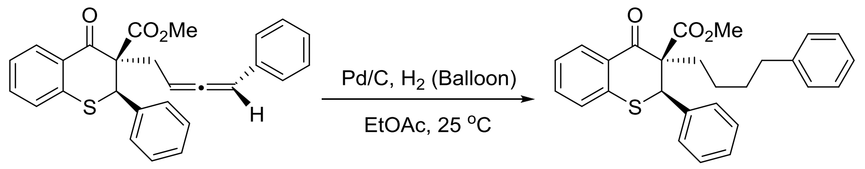

To a solution of the chiral allenylic alkylation product (-)-**3ca** (42.7 mg, 0.1 mmol, 99% ee) in ethyl acetate (1.0 mL) was added 10% Pd/C (5.3 mg, 0.005 mmol). The resulting mixture was degassed and stirred under hydrogen gas balloon pressure for about 13 hours at 25 °C. After the completion of hydrogenation, the volatiles were removed under the reduced pressure. The crude residue was purified by flash column chromatography on silica gel using hexanes/ethyl acetate (30/1-20/1) as eluent to give the desirable allene hydrogenation product (2*R*,3*S*)-(+)-**7**.

(2*R*,3*S*)-(+)-Methyl 4-oxo-2-phenyl-3-(4-phenylbutyl)thiochromane-3-carboxylate (7):
40.8 mg, 95% yield, colorless liquid, new compound, R_f = 0.28 (hexanes/ethyl acetate 20/1), 97% ee, $[\alpha]^{20}_D$ = +14.31 (*c* 1.02, $CHCl_3$). ^1H NMR (400 MHz, $CDCl_3$) δ 8.16 (dd, *J* = 8.2, 1.3 Hz, 1H), 7.43-7.37 (m, 1H), 7.33-7.22 (m, 9H), 7.18-7.09 (m, 3H), 4.68 (s, 1H), 3.61 (s, 3H), 2.62-2.44 (m, 2H), 2.17-2.05 (m, 1H), 1.93-1.80 (m, 1H), 1.63-1.48 (m, 3H), 1.27-1.17 (m, 1H). ^{13}C NMR (100 MHz, $CDCl_3$) δ 192.7, 170.4, 142.4, 141.3, 136.1, 133.1, 130.9, 130.4, 129.0, 128.7, 128.5, 128.4, 128.3, 127.2, 125.7, 125.5, 62.6, 52.2, 51.9, 35.5, 32.8, 31.8, 24.2. HPLC: Chiralpak AD-H column, 254 nm, 30 °C, *n*-Hexane/*i*-PrOH = 95/5, flow = 1.0 mL/min, retention time 11.7 min and 14.4 min (major). HRMS Calculated for $C_{27}H_{26}NaO_3S$ $[M+Na]^+$ 453.1495, found: 453.1503.

5.3. The Reduction of Carbonyl Functional Group

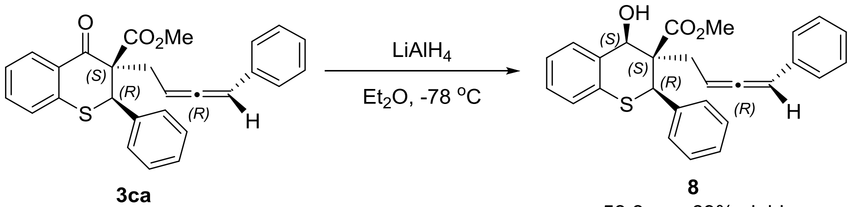

To a solution of lithium aluminium tetrahydride (15.2 mg, 0.4 mmol) in diethyl ether (7.0 mL) at -78 ^{o}C, the chiral compound (-)-**3ca** (85.3 mg, 0.2 mmol, 99% ee) and diethyl ether (1.0 mL) were added. The mixture was stirred at -78 ^{o}C for 5 hours. The reaction was quenched with 0.5 M potassium sodium tartrate aqueous solution (1.0 mL) and warmed to room temperature. After filtration through the celite, the combined organic layer was dried over sodium sulfate, filtered and concentrated under the reduced pressure. The residue was purified by column chromatography on silica gel using hexanes/ethyl acetate (20/1-10/1) as eluent to afford the desirable ketone carbonyl reductive product (-)-**8**.

The relative configuration of the hydroxyl group of the reductive product **8** was assigned as *S* by NOE.

(-)-Methyl 4-hydroxy-2-phenyl-3-(4-phenylbuta-2,3-dien-1-yl)thiochromane-3-carboxylate 8: 58.8 mg, 69% yield, colorless liquid, new compound, R_f = 0.48 (hexanes/ethyl acetate 5/1), 13.3:1 dr, 99% ee (major diastereoisomer), $[\alpha]^{20}_{D}$ = -33.73 (*c* 0.83, $CHCl_3$). The major diastereoisomer: ^{1}H NMR (700 MHz, $CDCl_3$) δ 7.52 (d, *J* = 7.5 Hz, 1H), 7.49-7.42 (m, 2H), 7.29-7.17 (m, 11H), 6.15-6.08 (m, 1H), 5.56-5.53 (m, 1H), 5.17 (d, *J* = 6.3 Hz, 1H), 4.58 (s, 1H), 3.55 (s, 3H), 3.43 (d, *J* = 6.4 Hz, 1H), 2.87-2.82 (m, 1H), 2.52-2.48 (m, 1H). ^{13}C NMR (175 MHz, $CDCl_3$) δ 207.0, 173.6, 138.6, 133.9, 133.9, 132.7, 130.4, 130.2, 128.6, 128.3, 127.9, 127.7, 127.1, 126.9, 125.8, 125.2, 94.8, 89.4, 72.3, 55.4, 52.0, 50.1, 36.4. HPLC: Chiralpak IA + AS-H column, 254 nm, 30 ^{o}C, *n*-Hexane/*i*-PrOH = 95/5, flow = 0.8 mL/min, retention time 62.0 min and 72.2 min (major). HRMS Calculated for $C_{27}H_{24}NaO_3S$ $[M+Na]^+$ 451.1338, found: 451.1335.

The NOE Result of Carbonyl Reduction (-)-8

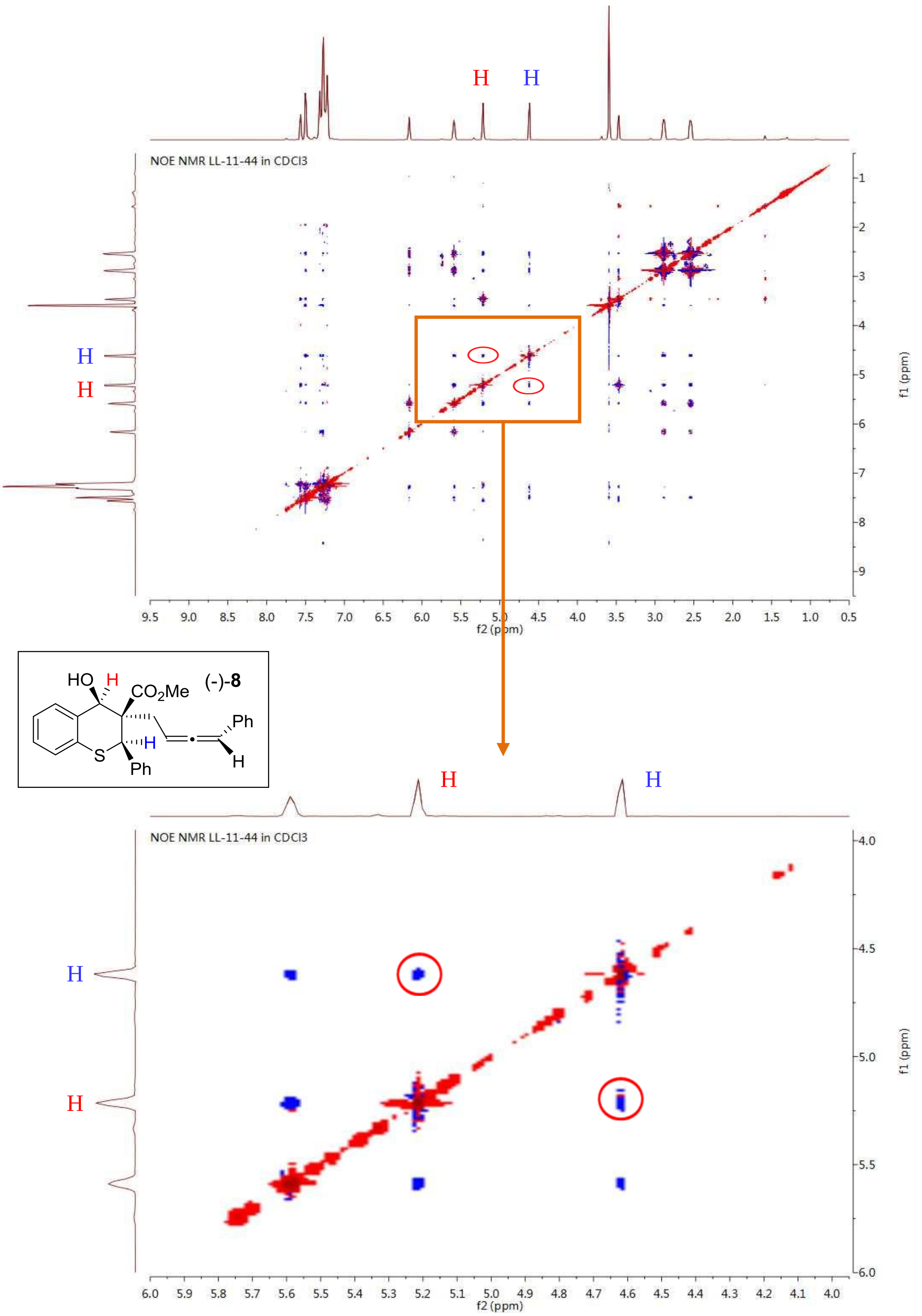

Figure S1. NOE spectrum of **(-)-8**

6. Determination of Absolute Configuration

To determine the absolute configuration of (-)-methyl 3-(4-(naphthalen-2-yl)buta-2,3 -dien-1-yl) -6-nitro-4-oxo-2-phenylthiochromane-3-carboxylate (**3mk**, 32.3:1 dr, >99% ee): (-)-**3mk** was completely dissolved in dichloromethane (2.0 mL), and *n*-hexane (2.0 mL) was added slowly at room temperature. The solvent diffused slowly, and the single crystal was obtained after about 36 hours. The structure in **Figure S2** showed the absolute configuration is (2*R*,3*S*,R_a). The CCDC number is 2214958. These details can be obtained free of charge *via* www.ccdc.com.ac.uk/ data_request/cif from the Cambridge Crystallographic Data Centre.

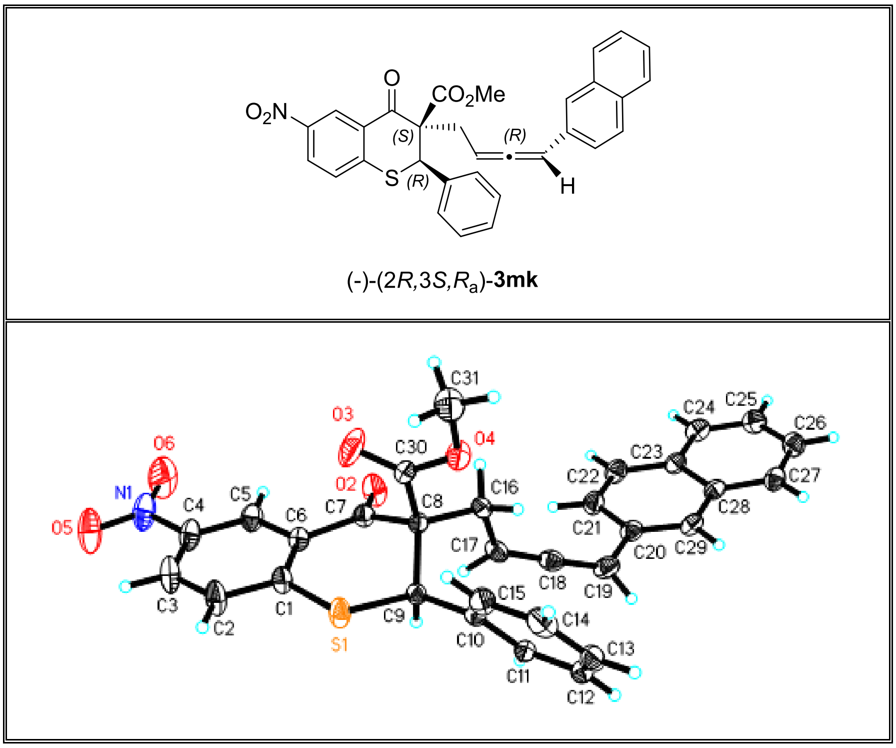

Figure S2. X-Ray Structure of (-)-(2*R*,3*S*,R_a)-**3mk**

Crystal Data and Structure Refinement for mo_d8v22443_0m for (-)-(2*R*,3*S*,*R*$_a$)-3mk

Identification code	mo_d8v22443_0m	
Empirical formula	$C_{31}H_{23}NO_5S$	
Formula weight	521.56	
Temperature	213(2) K	
Wavelength	0.71073 Å	
Crystal system	Orthorhombic	
Space group	P 21 21 21	
Unit cell dimensions	a = 6.8379(3) Å	α = 90 °
	b = 16.3952(5) Å	β = 90 °
	c = 22.7541(8) Å	γ = 90 °
Volume	2550.93(16) Å^3	
Z	4	
Density (calculated)	1.358 Mg/m^3	
Absorption coefficient	0.170 mm^{-1}	
F(000)	1088	
Crystal size	0.200 x 0.150 x 0.120 mm^3	
Theta range for data collection	2.641 to 25.997 °.	
Index ranges	-8<=h<=8, -20<=k<=18, -28<=l<=28	
Reflections collected	22409	
Independent reflections	5005 [R(int) = 0.0593]	
Completeness to theta = 25.242 °	99.4 %	
Absorption correction	Semi-empirical from equivalents	
Max. and min. transmission	0.7456 and 0.5174	
Refinement method	Full-matrix least-squares on F^2	
Data / restraints / parameters	5005 / 0 / 344	
Goodness-of-fit on F2	1.025	
Final R indices [I>2sigma(I)]	R1 = 0.0366, wR2 = 0.0887	
R indices (all data)	R1 = 0.0415, wR2 = 0.0925	
Absolute structure parameter	0.02(4)	
Extinction coefficient	n/a	
Largest diff. peak and hole	0.187 and -0.168 e.Å^{-3}	

7. References

1. (a) Choudhury, A. R.; Mukherjee, S. A Catalytic Michael/Horner-Wadsworth-Emmons Cascade Reaction for Enantioselective Synthesis of Thiochromenes. *Adv. Synth. Catal.* **2013**, *355*, 1989-1995. (b) Kirkeby, E. K.; Roberts, A. G. Design, Synthesis and Characterization of Structurally Dynamic Cyclic *N*,*S*-Acetals. *Chem. Commun.*, **2020**, *56*, 9118-9121. (c) Dong, X.-Q.; Fang, X.; Tao, H.-Y.; Zhou, X.; Wang, C.-J. Organocatalytic Asymmetric Domino sulfa-Michael-aldol Reactions of 2-Mercaptobenzaldehyde with *α*,*β*-Unsaturated *N*-Acylpyrazoles for the Construction of Thiochromane. *Chem. Commun.*, **2012**, *48*, 7238-7240. (d) Zhang, Y.-P.; You, Y.; Zhao, J.-Q.; Zhang, X.-M.; Xu, X.-Y.; Yuan, W.-C. Chiral Bifunctional Amine-Squaramide-Catalyzed Highly Diastereo- and Enantioselective Michael/Aldol Cascade Reaction of 2-Mercaptobenzaldehyde and *α*,*β*-Unsaturated 7-Azaindoline Amides. *J. Org. Chem.* **2019**, *84*, 7984-7994.
2. Meyer, C.; Piva, O.; Pete, J.-P. [2+2] Photocycloadditions and Photorearrangements of 2-Alkenylcarboxamido-2-cycloalken-1-ones. *Tetrahedron* **2000**, *56*, 4479-4489.
3. (a) Huang, X.; Cao, T.; Han, Y.; Jiang, X.; Lin, W.; Zhang, J.; Ma, S. General $CuBr_2$ -Catalyzed Highly Enantioselective Approach for Optically Active Allenols from Terminal Alkynols. *Chem. Commun.*, **2015**, *51*, 6956-6959. (b) Song, S.; Ma, S. Highly Selective Nucleophilic 4-Aryl-2,3-allenylation of Malonates. *Chin. J. Chem*. **2020**, *38*, 1233-1238.
4. Chen, H.; Jia, X.; Yu, Y.; Qian, Q.; Gong, H. Nickel-Catalyzed Reductive Allylation of Tertiary Alkyl Halides with Allylic Carbonates. *Angew. Chem. Int. Ed*. **2017**, *56*, 13103-13106.
5. Tang, X.; Woodward, S.; Krause, N. Synthesis of α-Hydroxyallenes by Copper-Catalyzed S_N2’ Substitution of Propargylic Dioxolanones. *Eur. J. Org. Chem.* **2009**, 2836-2844.

8. Copy of NMR and HPLC Spectra

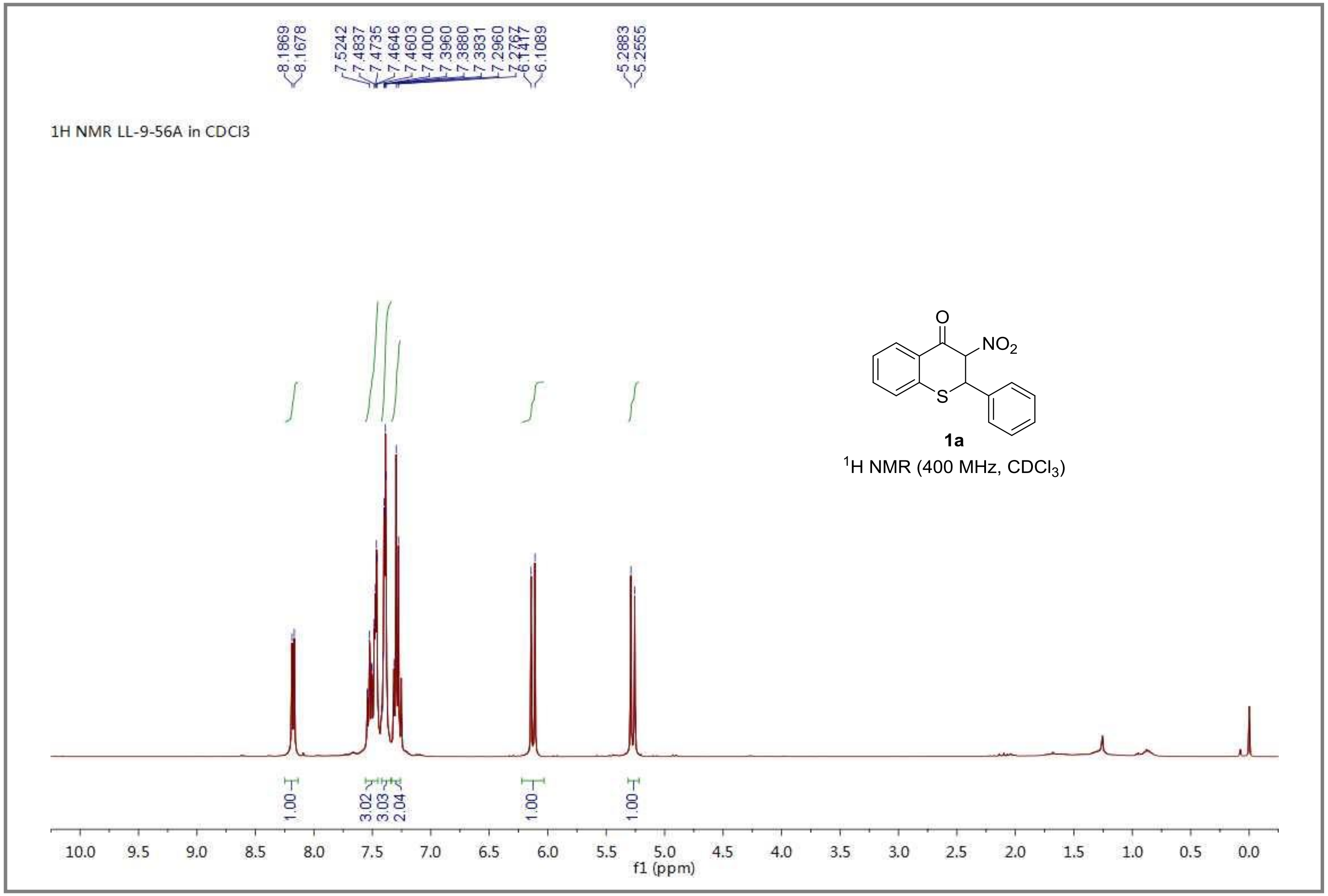

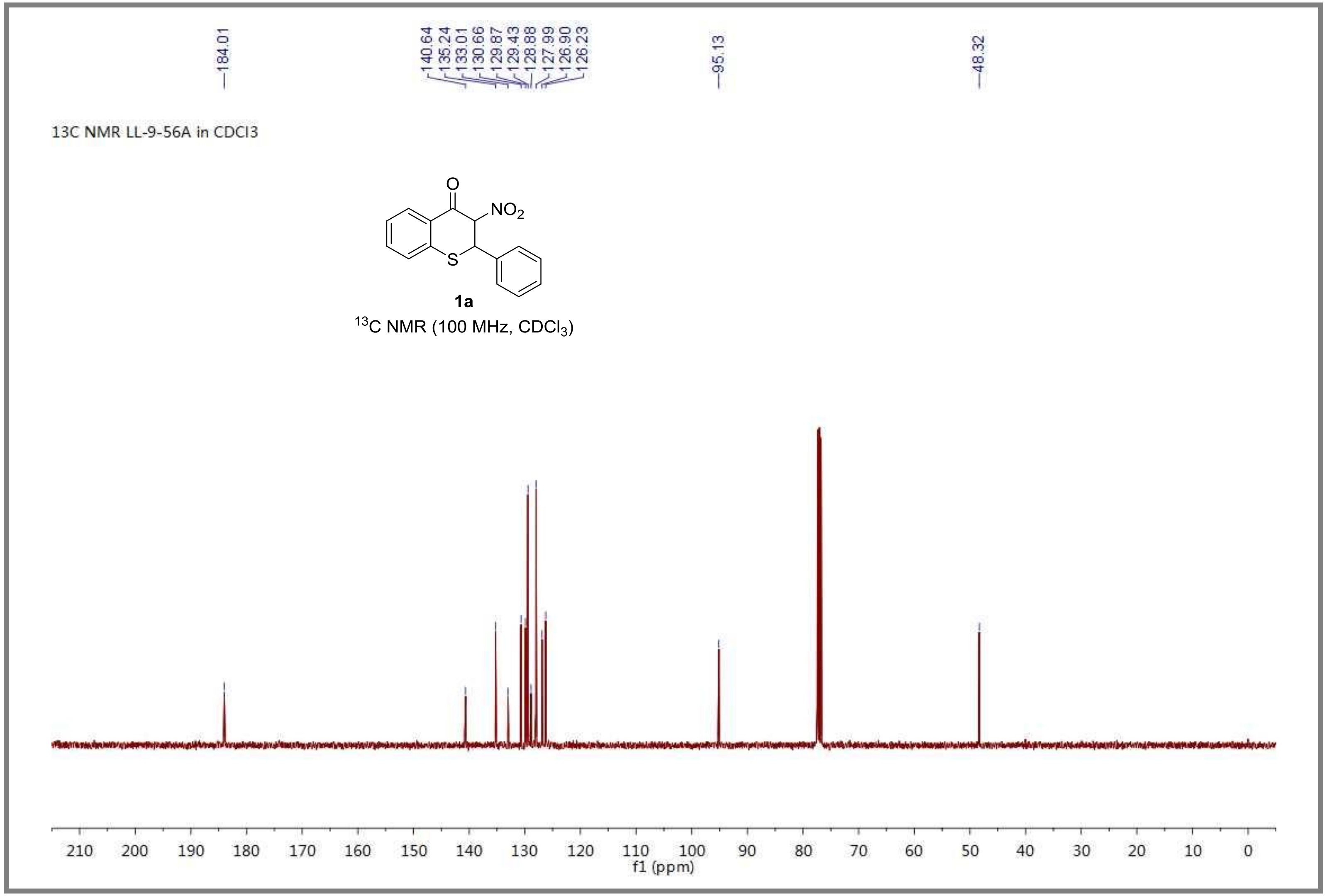
184.01
140.64
135.24
133.01
130.66
129.87
129.43
128.88
127.99
126.90
126.23
95.13
48.32
13C NMR LL-9-56A in CDCl3
O
NO2
S
1a
13C NMR (100 MHz, CDCl3)
210 200 190 180 170 160 150 140 130 120 110 100 90 80 70 60 50 40 30 20 10 0
f1 (ppm)

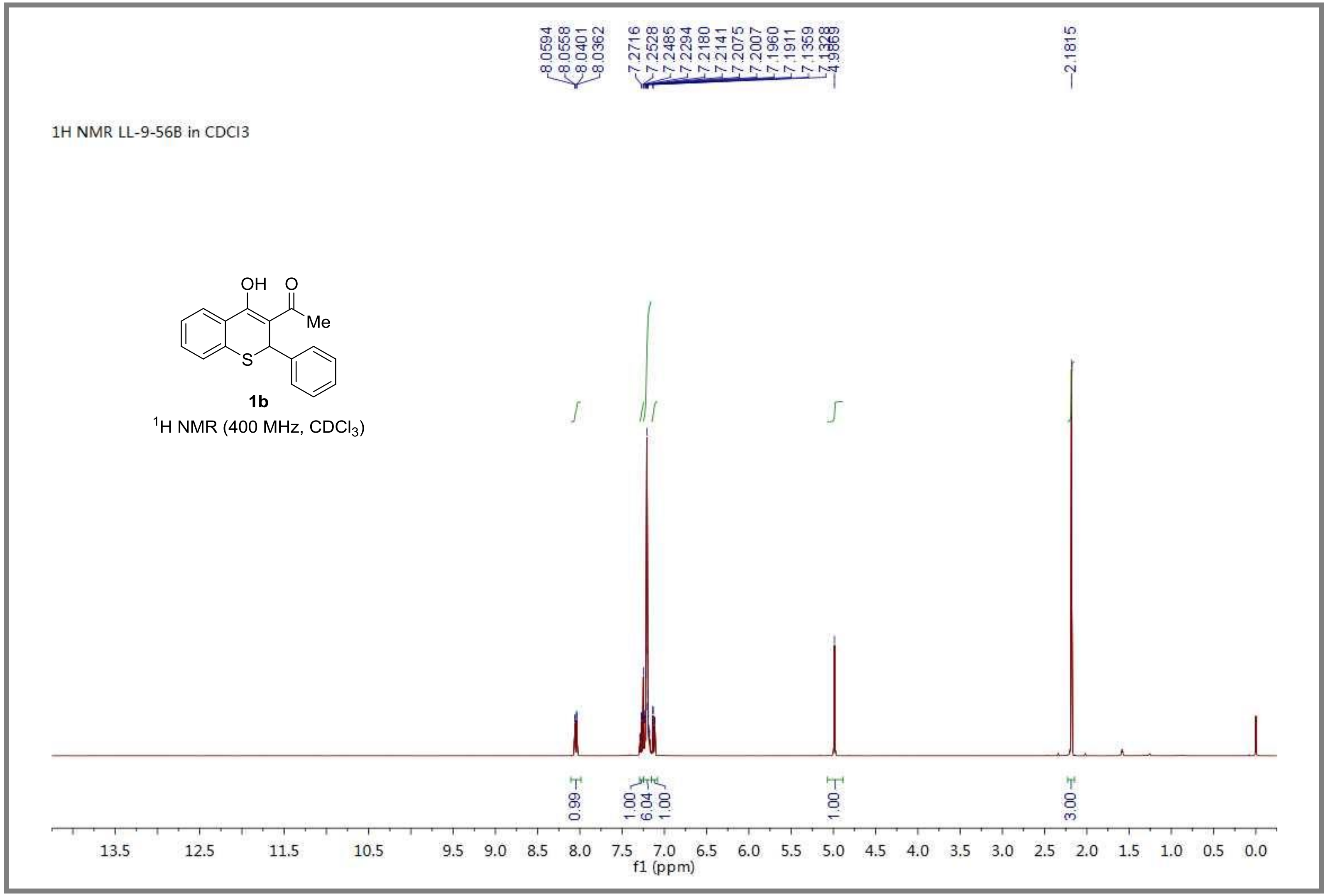
8.0594
8.0558
8.0401
8.0362
7.2716
7.2528
7.2485
7.2294
7.2180
7.2141
7.2075
7.2007
7.1960
7.1911
7.1359
7.1328
4.9869
2.1815
1H NMR LL-9-56B in CDCl3
OH
O
Me
S
1b
^{1}H NMR (400 MHz, $CDCl_3$)
0.99
1.00
6.04
1.00
1.00
3.00
13.5
12.5
11.5
10.5
9.5
9.0
8.5
8.0
7.5
7.0
6.5
6.0
5.5
5.0
4.5
4.0
3.5
3.0
2.5
2.0
1.5
1.0
0.5
0.0
f1 (ppm)

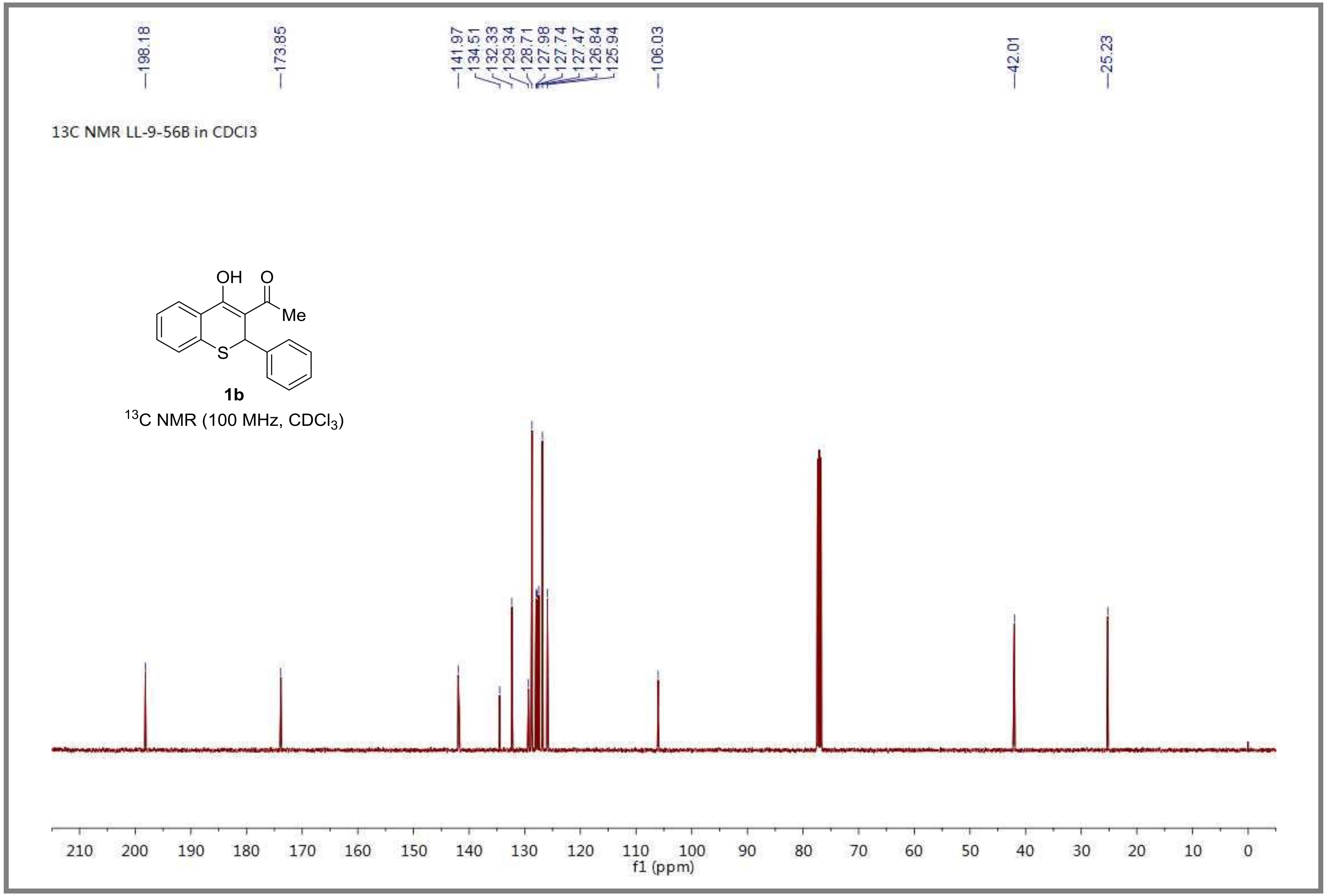

1b

^{13}C NMR (100 MHz, $CDCl_3$)

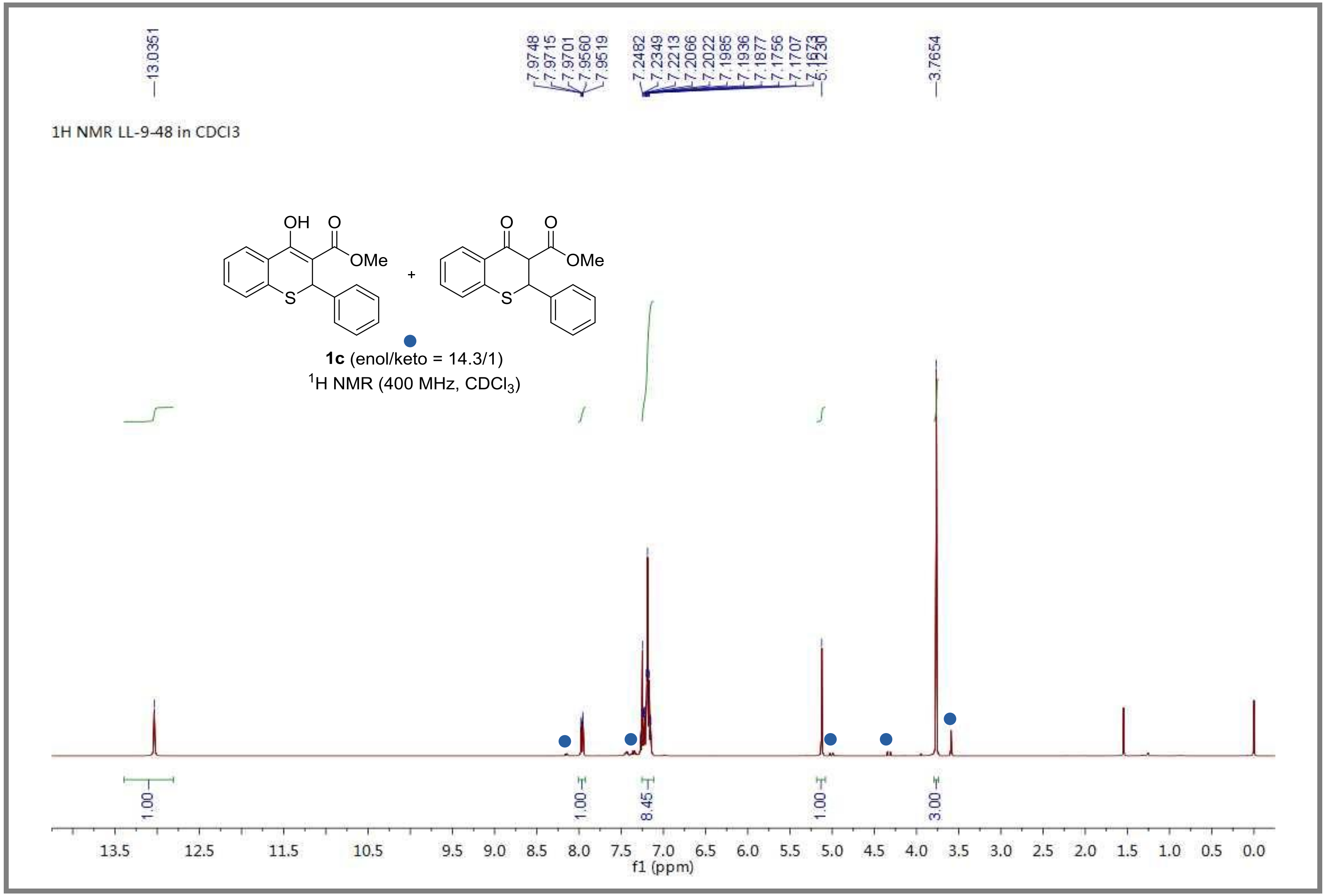
1H NMR LL-9-48 in CDCl3
OH
O
OMe
S
+
O
O
OMe
S
1c (enol/keto = 14.3/1)
1H NMR (400 MHz, CDCl3)
13.0351
7.9748
7.9715
7.9701
7.9560
7.9519
7.2482
7.2349
7.2213
7.2066
7.2022
7.1985
7.1936
7.1877
7.1756
7.1707
7.1673
5.1230
3.7654
1.00
1.00
8.45
1.00
3.00
f1 (ppm)

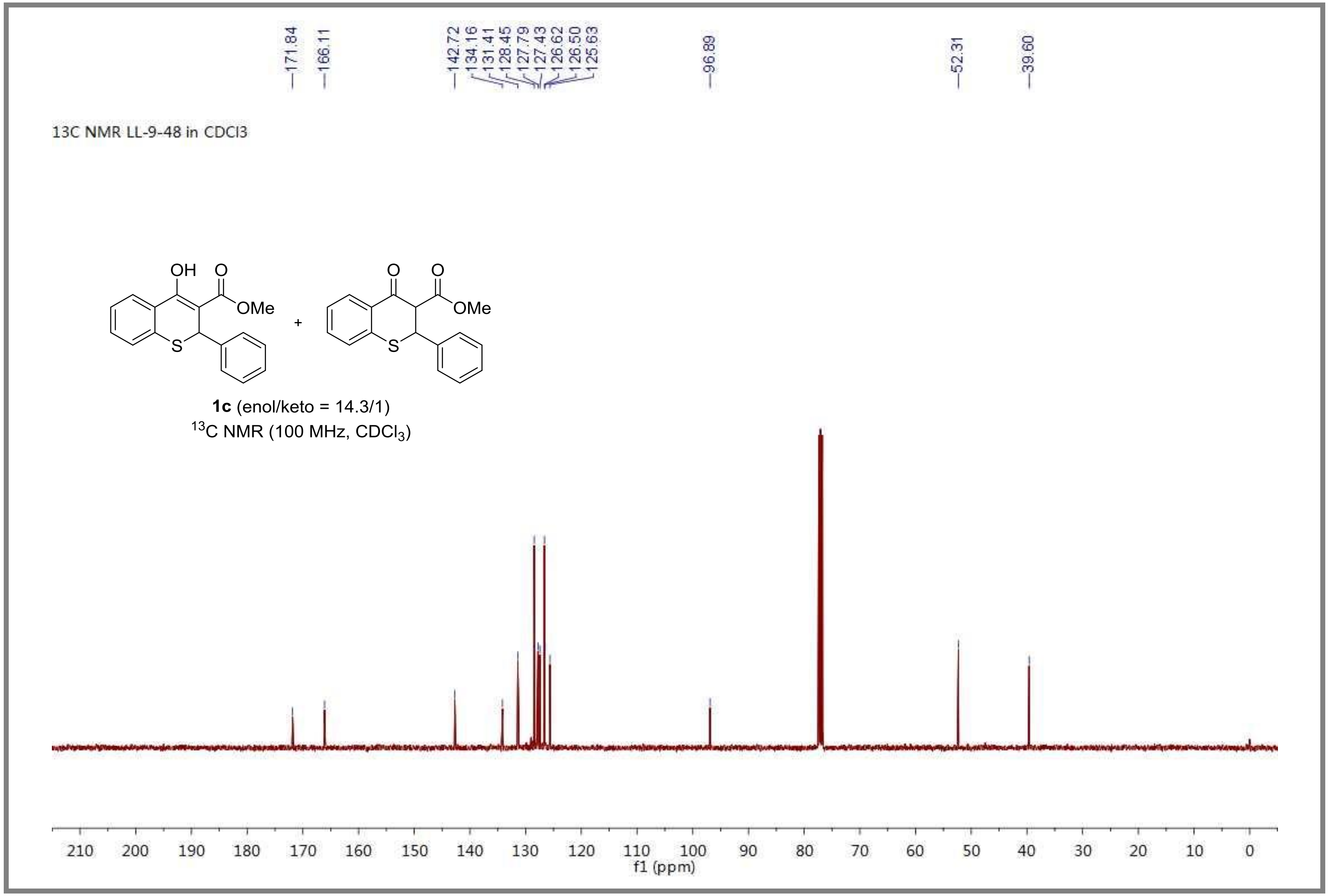
171.84
166.11
142.72
134.16
131.41
128.45
127.79
127.43
126.62
126.50
125.63
96.89
52.31
39.60
13C NMR LL-9-48 in CDCl3
OH
O
OMe
S
+
O
O
OMe
S
1c (enol/keto = 14.3/1)
13C NMR (100 MHz, CDCl3)
210 200 190 180 170 160 150 140 130 120 110 100 90 80 70 60 50 40 30 20 10 0
f1 (ppm)

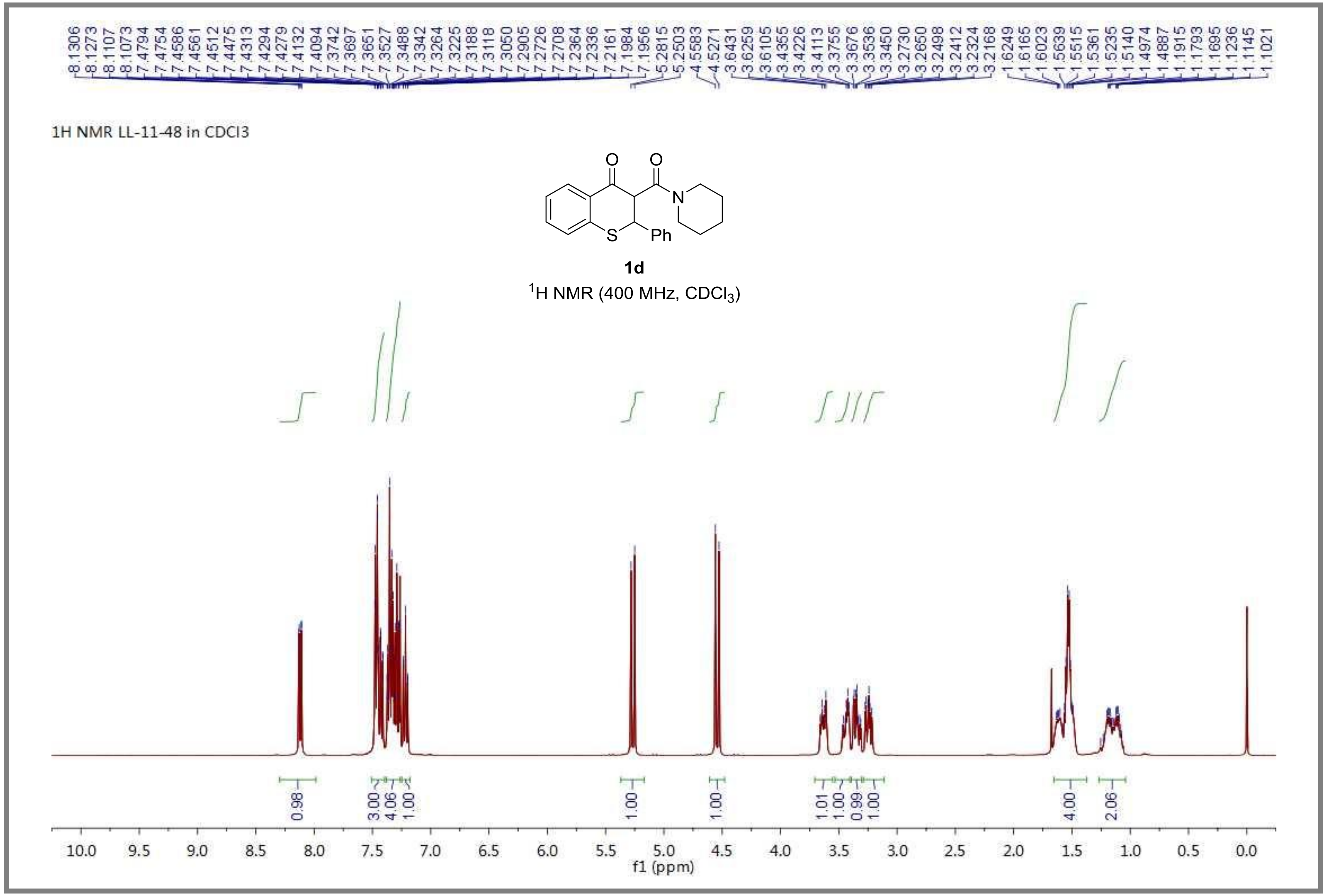
1H NMR LL-11-48 in CDCl3
1d
^{1}H NMR (400 MHz, CDCl$_3$)
f1 (ppm)

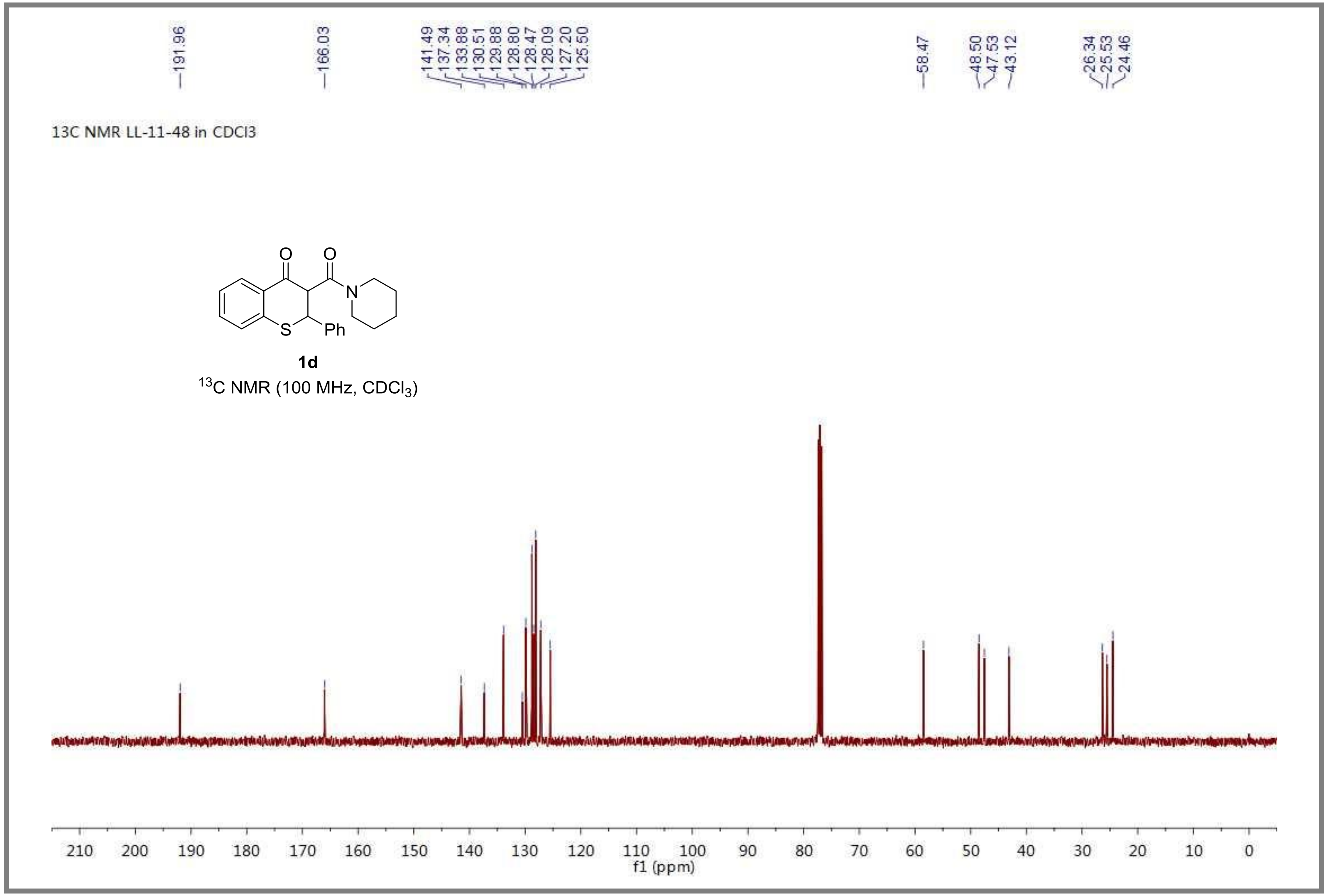

191.96
166.03
141.49
137.34
133.88
130.51
129.88
128.80
128.47
128.09
127.20
125.50
58.47
48.50
47.53
43.12
26.34
25.53
24.46
13C NMR LL-11-48 in CDCl3
O
O
N
S
Ph
1d
^{13}C NMR (100 MHz, $CDCl_3$)
210 200 190 180 170 160 150 140 130 120 110 100 90 80 70 60 50 40 30 20 10 0
f1 (ppm)

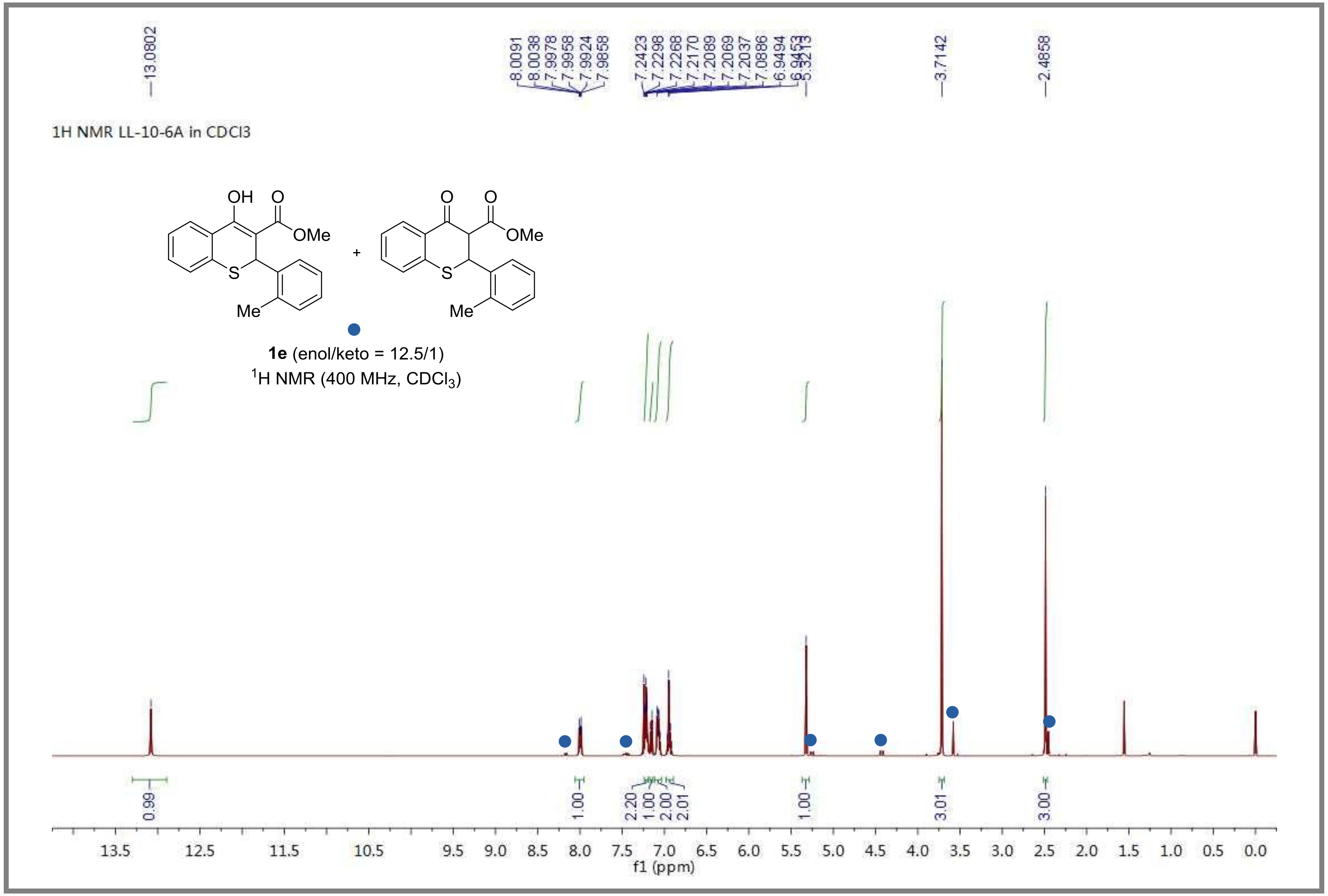
1H NMR LL-10-6A in CDCl3
1e (enol/keto = 12.5/1)
1H NMR (400 MHz, CDCl3)
f1 (ppm)

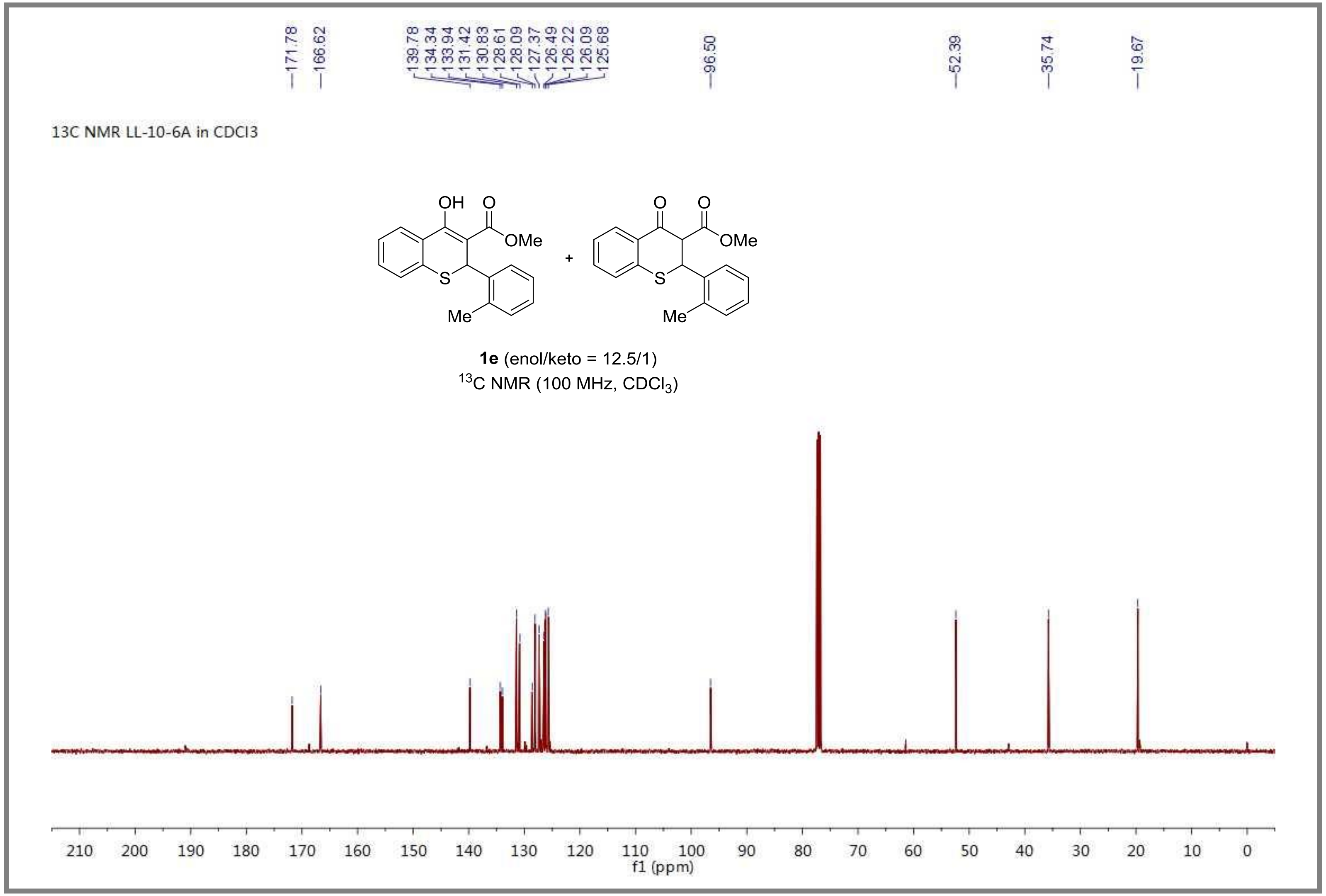
171.78
166.62
139.78
134.34
133.94
131.42
130.83
128.61
128.09
127.37
126.49
126.22
126.09
125.68
96.50
52.39
35.74
19.67
13C NMR LL-10-6A in CDCl3
OH
O
OMe
S
Me
+
O
O
OMe
S
Me
1e (enol/keto = 12.5/1)
13C NMR (100 MHz, CDCl3)
210 200 190 180 170 160 150 140 130 120 110 100 90 80 70 60 50 40 30 20 10 0
f1 (ppm)

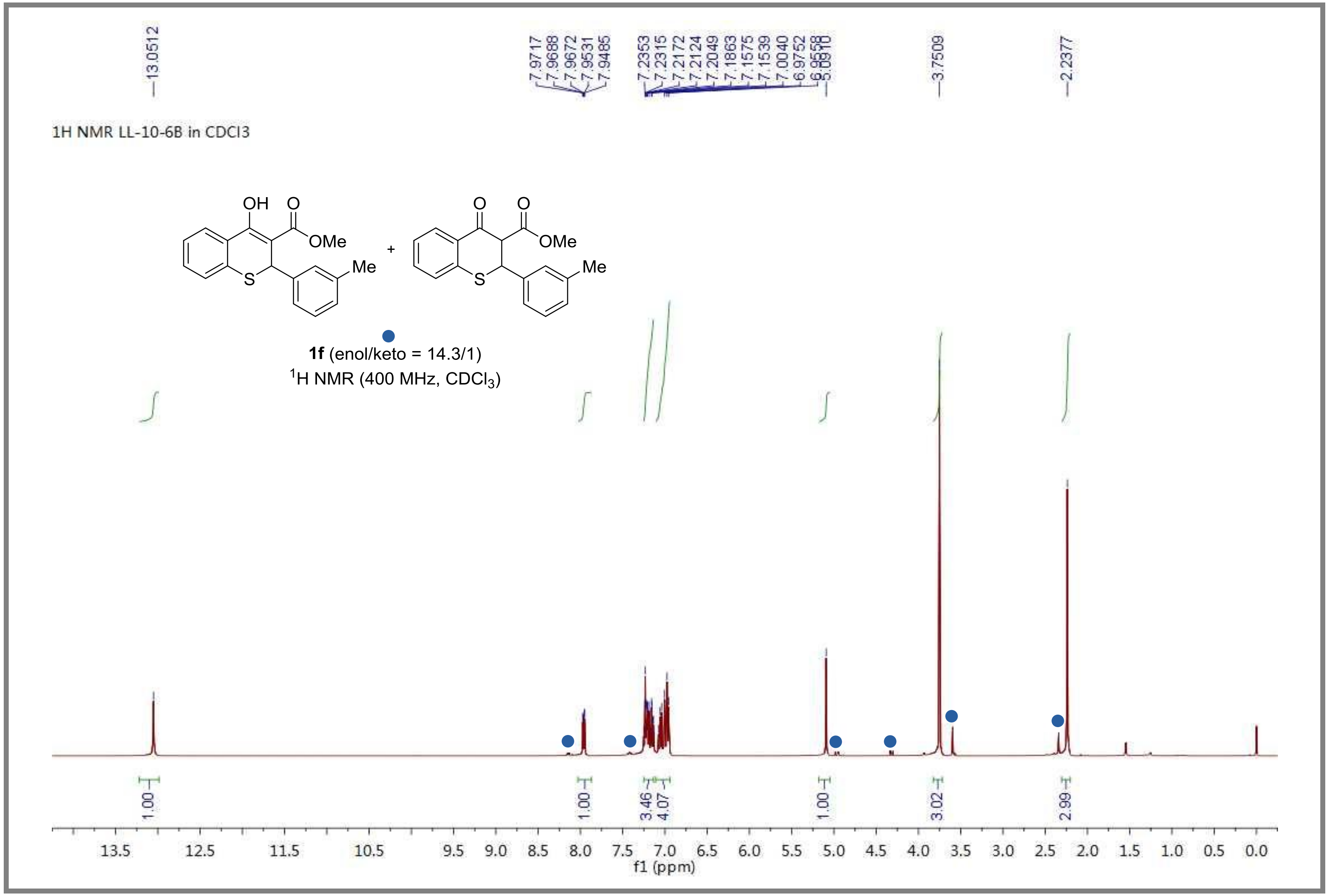

1H NMR LL-10-6B in CDCl3
13.0512
7.9717
7.9688
7.9672
7.9531
7.9485
7.2353
7.2315
7.2172
7.2124
7.2049
7.1863
7.1575
7.1539
7.0040
6.9752
3.7509
2.2377
OH
O
OMe
Me
S
+
1f (enol/keto = 14.3/1)
1H NMR (400 MHz, CDCl3)
1.00
1.00
3.46
4.07
1.00
3.02
2.99
f1 (ppm)

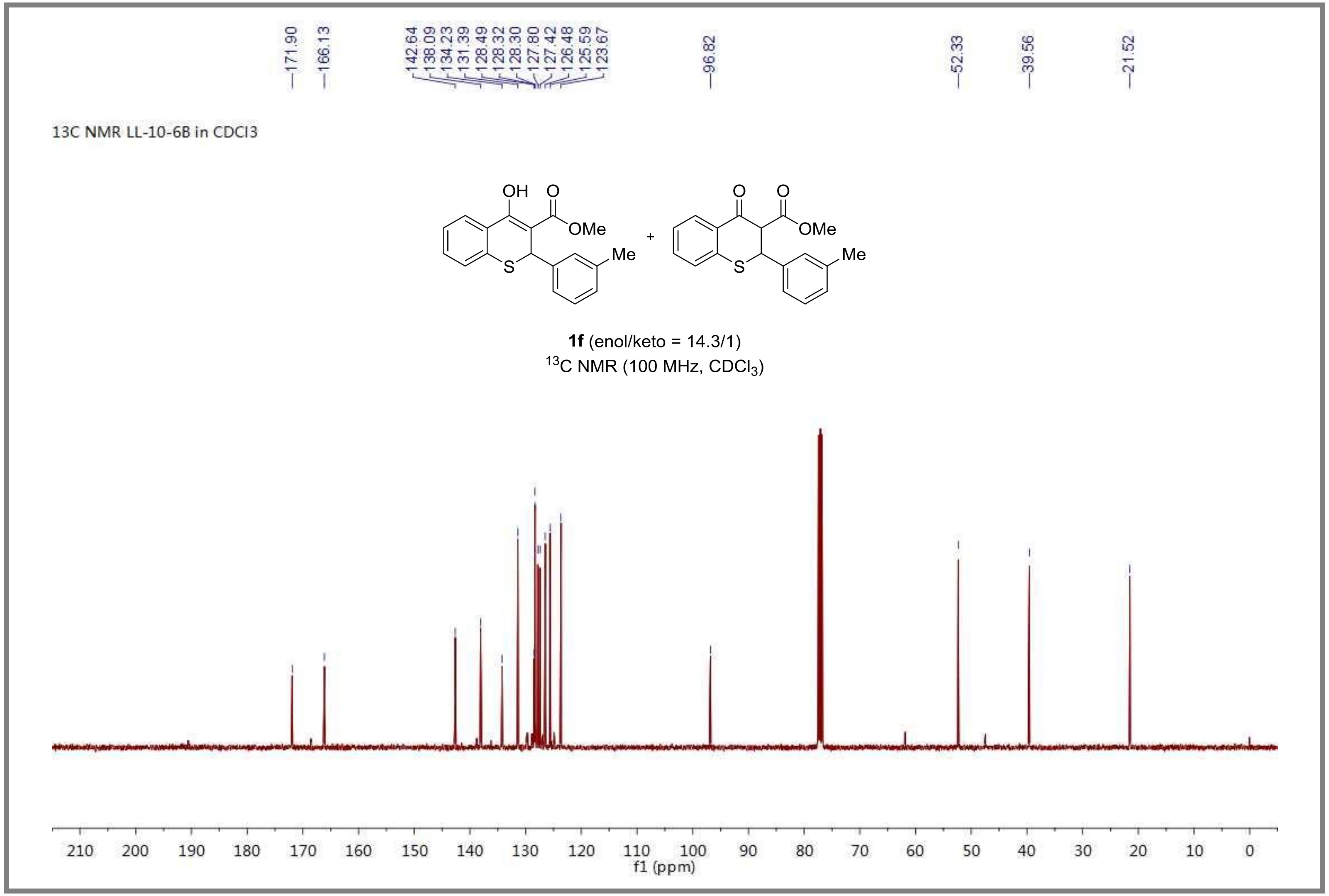

13C NMR LL-10-6B in CDCl3
OH
O
OMe
S
Me
+
O
O
OMe
S
Me
1f (enol/keto = 14.3/1)
13C NMR (100 MHz, CDCl3)
171.90
166.13
142.64
138.09
134.23
131.39
128.49
128.32
128.30
127.80
127.42
126.48
125.59
123.67
96.82
52.33
39.56
21.52
210
200
190
180
170
160
150
140
130
120
110
100
90
80
70
60
50
40
30
20
10
0
f1 (ppm)

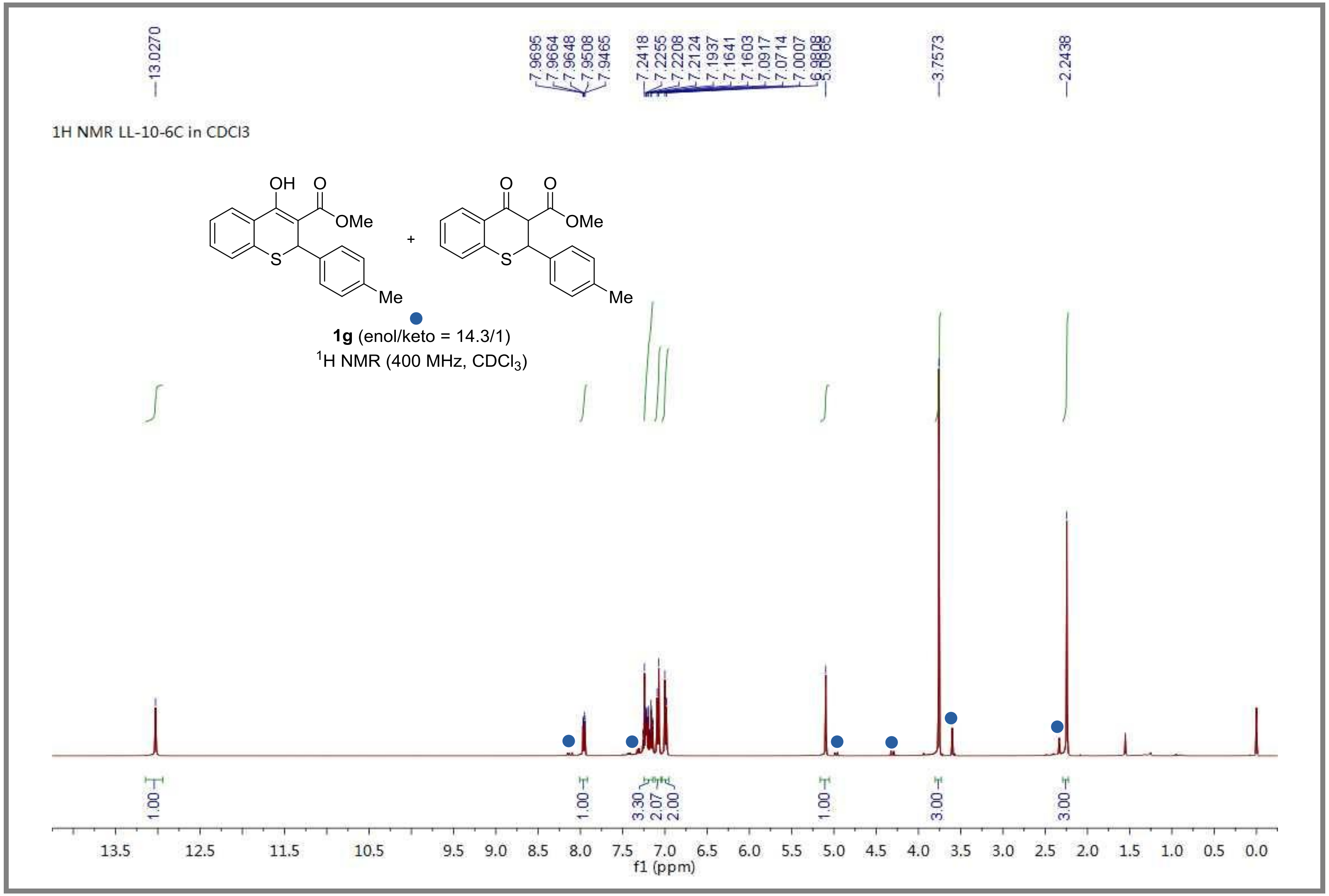

1H NMR LL-10-6C in CDCl3
OH O
OMe
S
Me
+
O O
OMe
S
Me
1g (enol/keto = 14.3/1)
^{1}H NMR (400 MHz, CDCl$_3$)
13.0270
7.9695
7.9664
7.9648
7.9508
7.9465
7.2418
7.2255
7.2208
7.2124
7.1937
7.1641
7.1603
7.0917
7.0714
7.0007
5.0965
3.7573
2.2438
1.00
1.00
3.30
2.07
2.00
1.00
3.00
3.00
f1 (ppm)

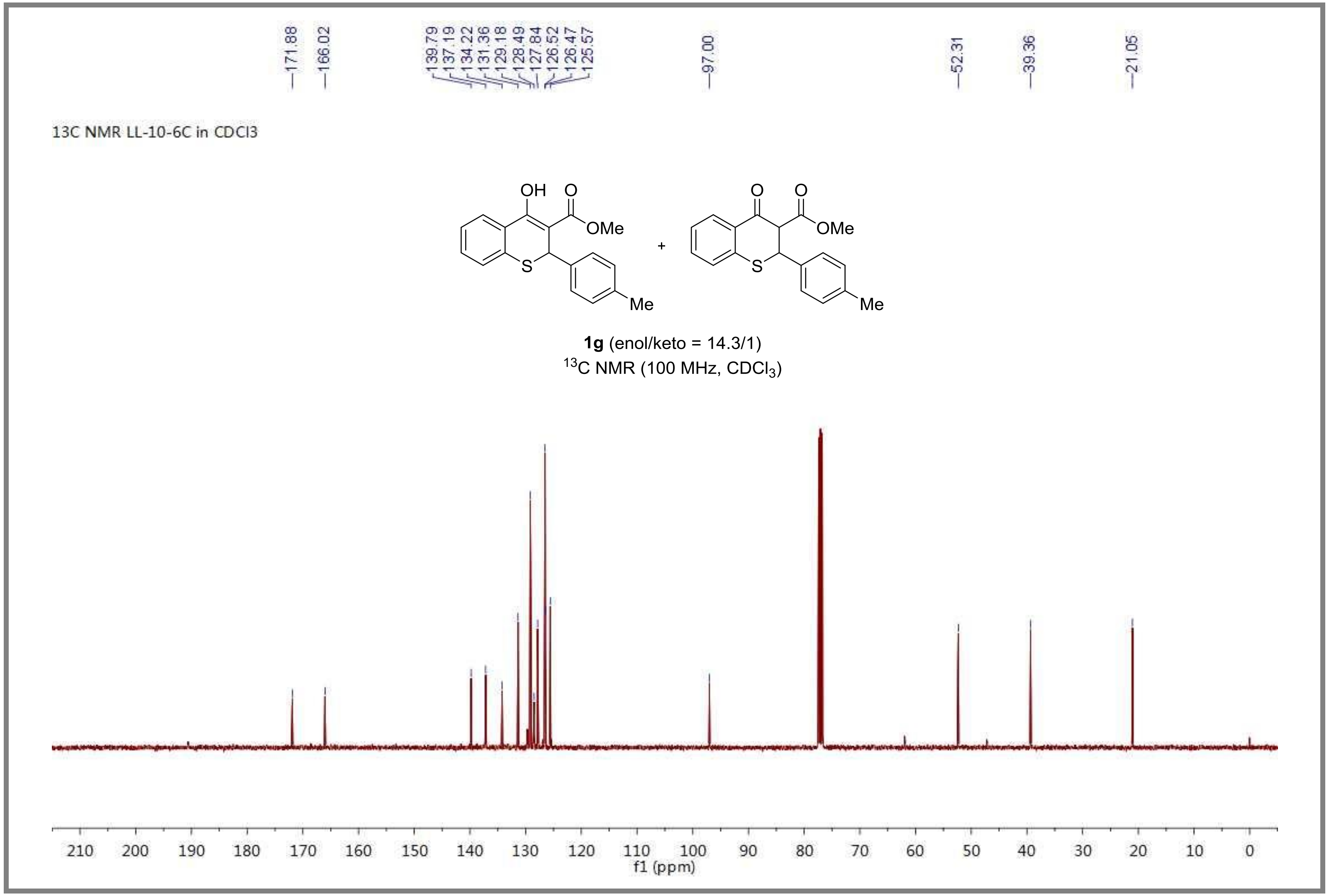

171.88
166.02
139.79
137.19
134.22
131.36
129.18
128.49
127.84
126.52
126.47
125.57
97.00
52.31
39.36
21.05
13C NMR LL-10-6C in CDCl3
OH
O
OMe
S
Me
+
O
O
OMe
S
Me
1g (enol/keto = 14.3/1)
13C NMR (100 MHz, CDCl3)
210 200 190 180 170 160 150 140 130 120 110 100 90 80 70 60 50 40 30 20 10 0
f1 (ppm)

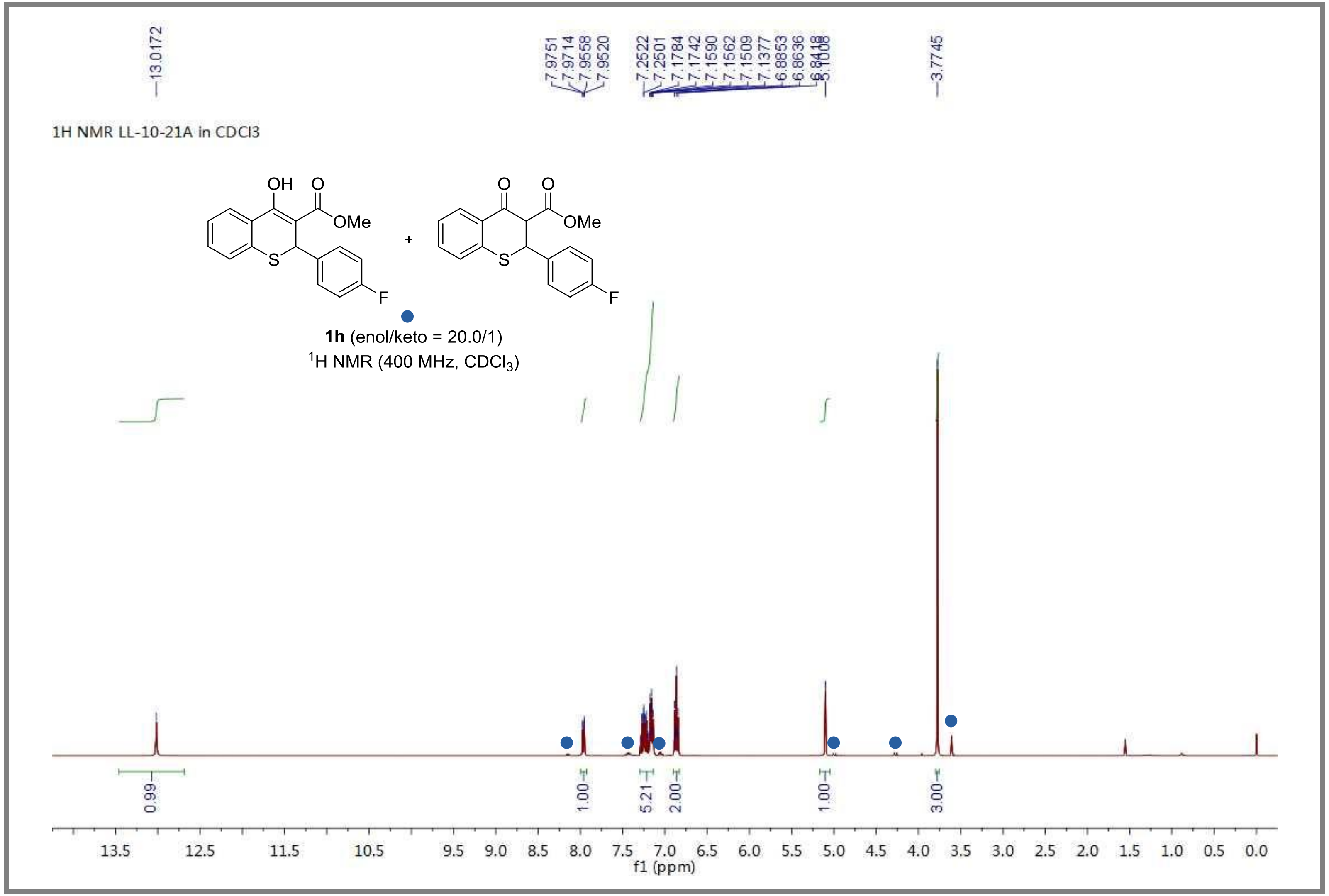
13.0172
7.9751
7.9714
7.9558
7.9520
7.2522
7.2501
7.1784
7.1742
7.1590
7.1562
7.1509
7.1377
6.8853
6.8636
3.7745
1H NMR LL-10-21A in CDCl3
OH
O
OMe
S
F
+
O
O
OMe
S
F
1h (enol/keto = 20.0/1)
1H NMR (400 MHz, CDCl3)
0.99
1.00
5.21
2.00
1.00
3.00
13.5
12.5
11.5
10.5
9.5
9.0
8.5
8.0
7.5
7.0
6.5
6.0
5.5
5.0
4.5
4.0
3.5
3.0
2.5
2.0
1.5
1.0
0.5
0.0
f1 (ppm)

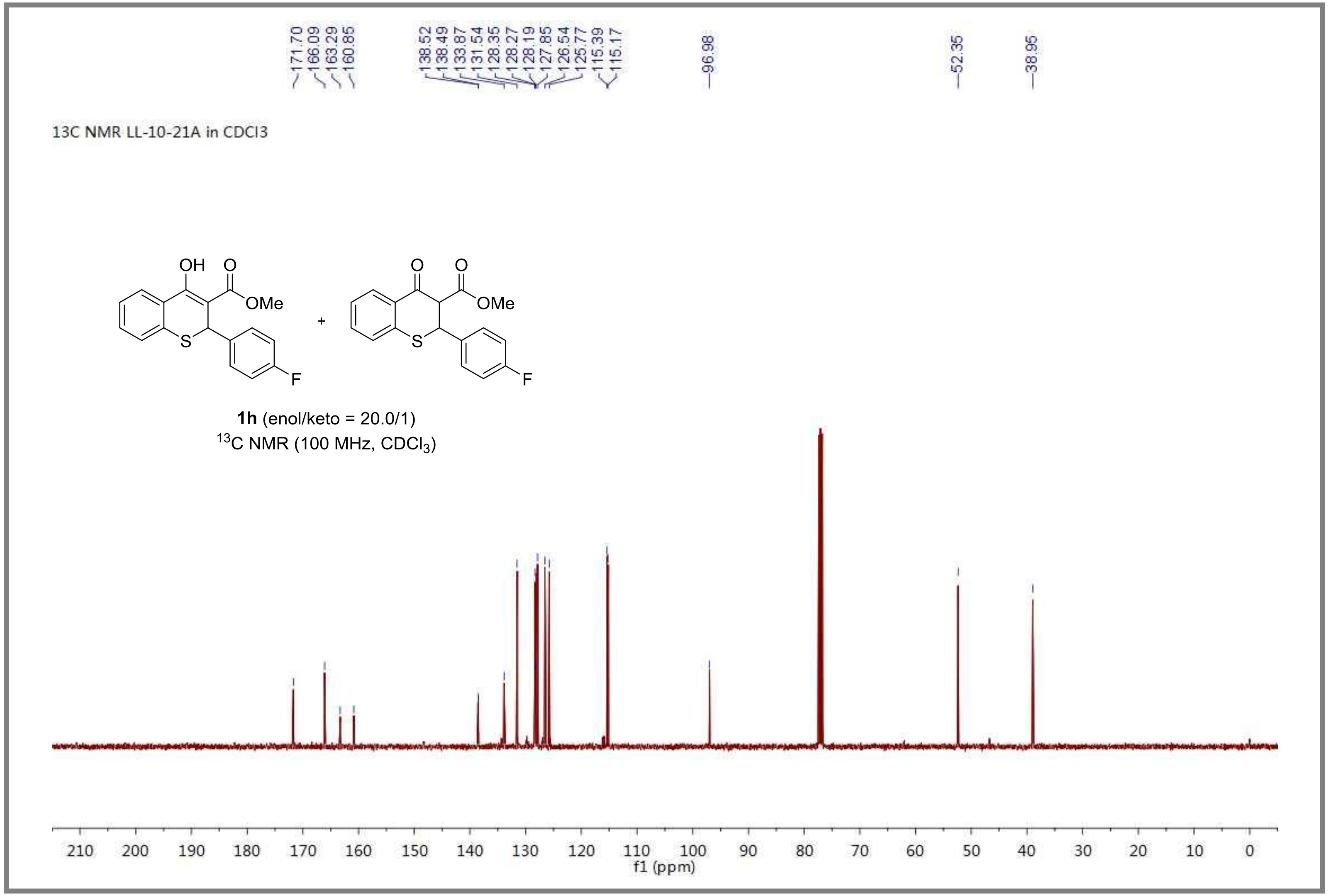

1h (enol/keto = 20.0/1)

^{13}C NMR (100 MHz, $CDCl_3$)

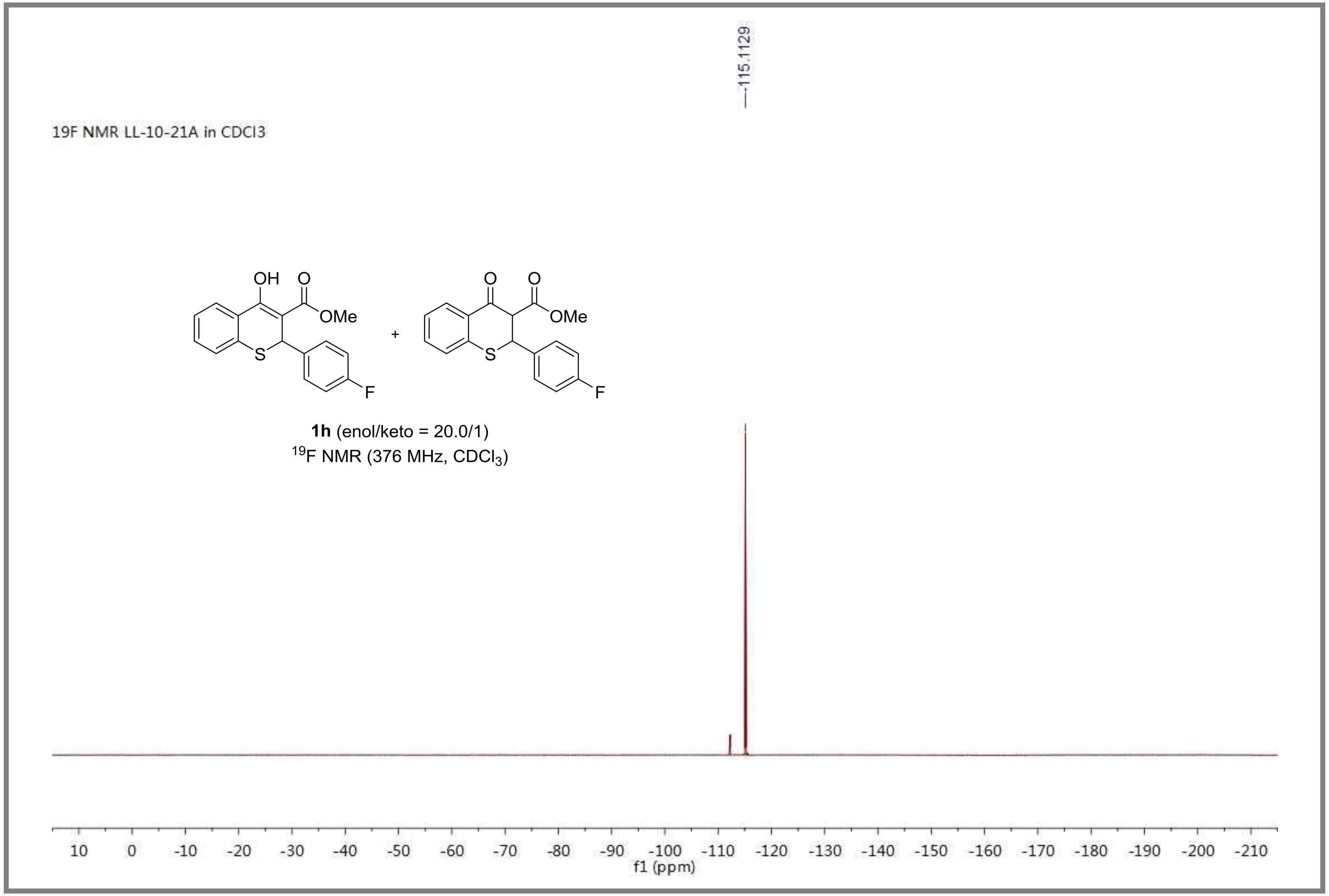

-115.1129
19F NMR LL-10-21A in CDCl3
OH
O
OMe
S
F
+
O
O
OMe
S
F
1h (enol/keto = 20.0/1)
19F NMR (376 MHz, CDCl3)
10
0
-10
-20
-30
-40
-50
-60
-70
-80
-90
-100
-110
-120
-130
-140
-150
-160
-170
-180
-190
-200
-210
f1 (ppm)

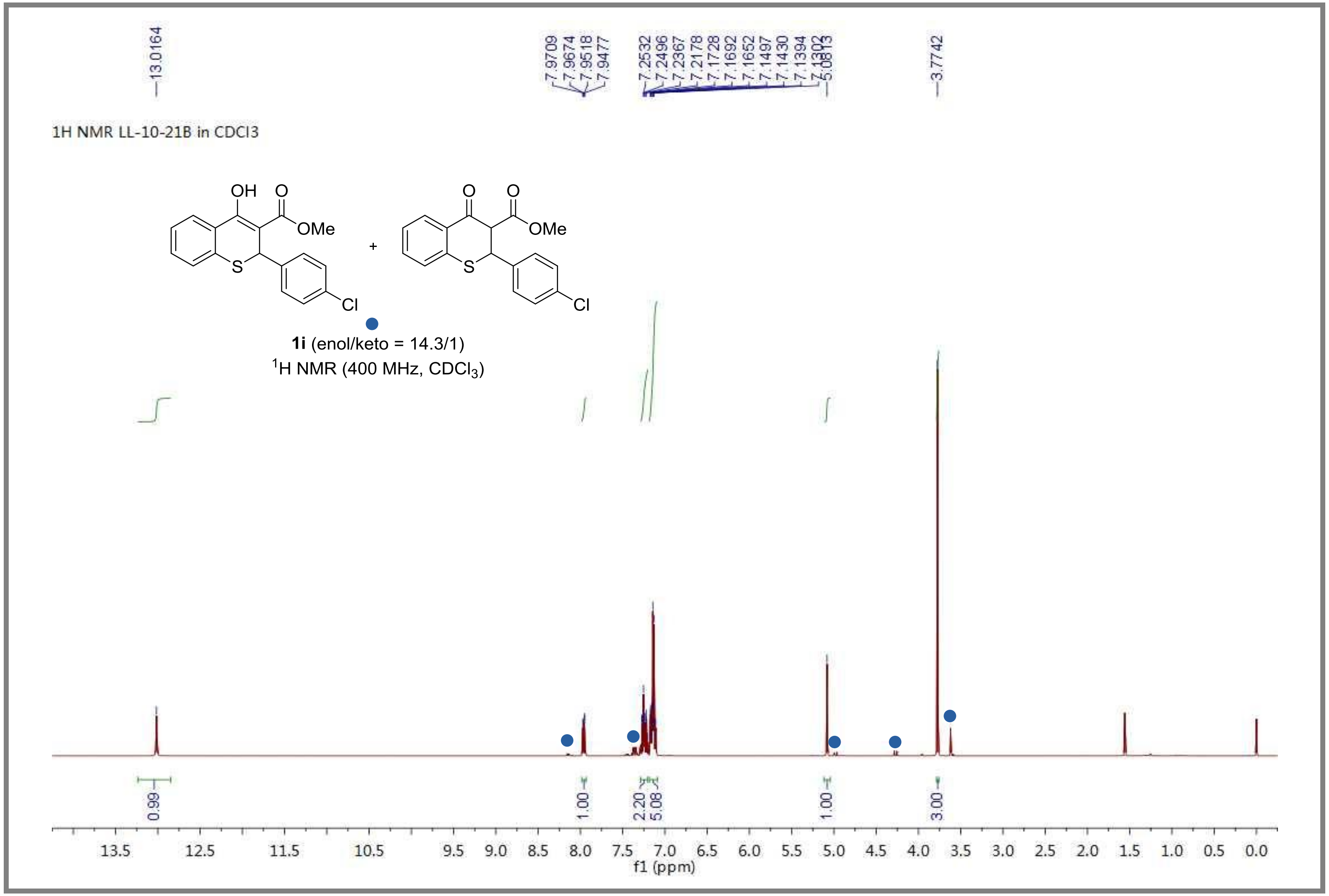

1H NMR LL-10-21B in CDCl3
OH
O
OMe
S
Cl
+
O
O
OMe
S
Cl
1i (enol/keto = 14.3/1)
1H NMR (400 MHz, CDCl3)
13.0164
7.9709
7.9674
7.9518
7.9477
7.2532
7.2496
7.2367
7.2178
7.1728
7.1692
7.1652
7.1497
7.1430
7.1394
7.1302
5.0813
3.7742
0.99
1.00
2.20
5.08
1.00
3.00
13.5
12.5
11.5
10.5
9.5
9.0
8.5
8.0
7.5
7.0
6.5
6.0
5.5
5.0
4.5
4.0
3.5
3.0
2.5
2.0
1.5
1.0
0.5
0.0
f1 (ppm)

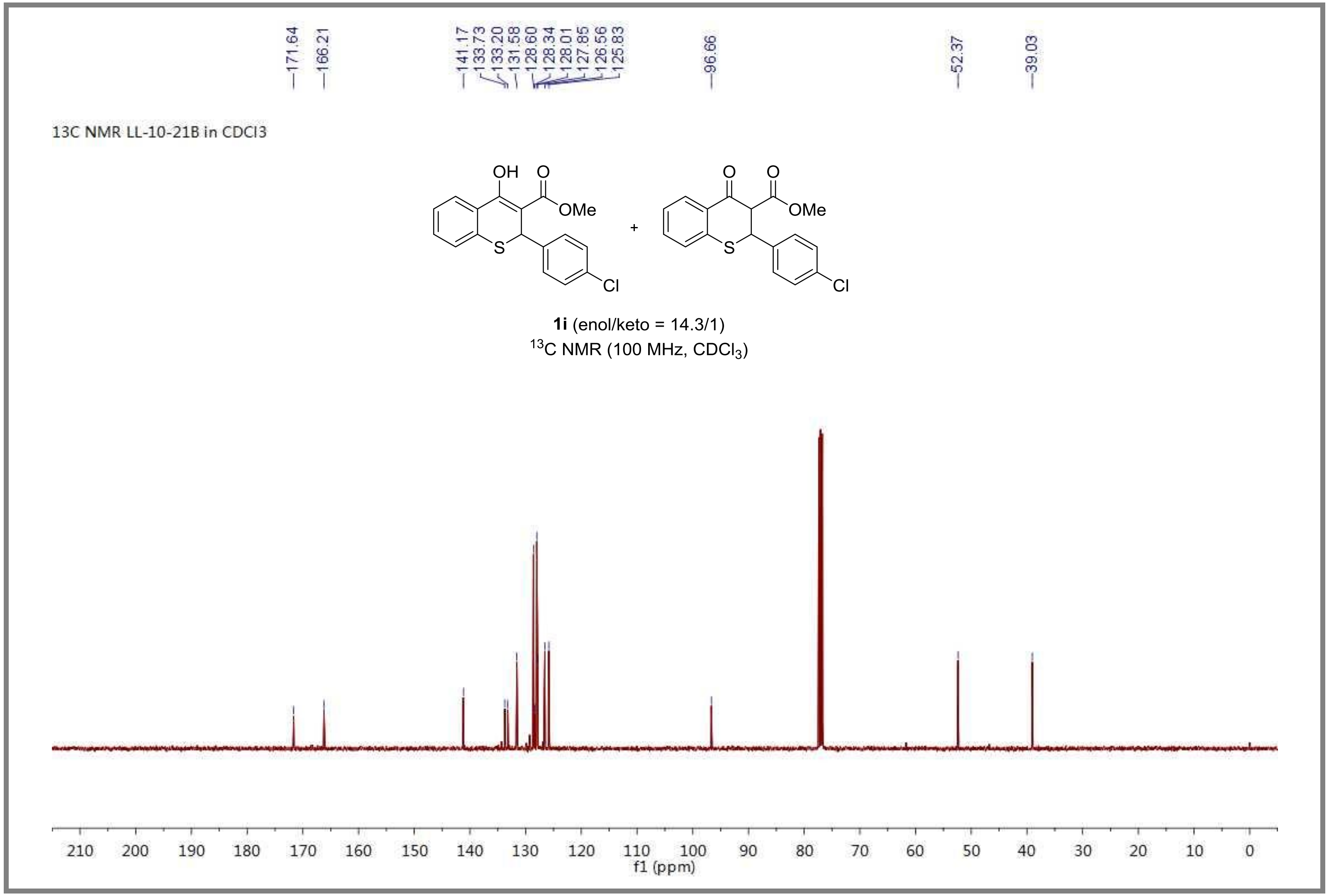

13C NMR LL-10-21B in CDCl3
171.64
166.21
141.17
133.73
133.20
131.58
128.60
128.34
128.01
127.85
126.56
125.83
96.66
52.37
39.03
OH
O
OMe
S
Cl
+
O
O
OMe
S
Cl
1i (enol/keto = 14.3/1)
^{13}C NMR (100 MHz, CDCl$_3$)
210
200
190
180
170
160
150
140
130
120
110
100
90
80
70
60
50
40
30
20
10
0
f1 (ppm)

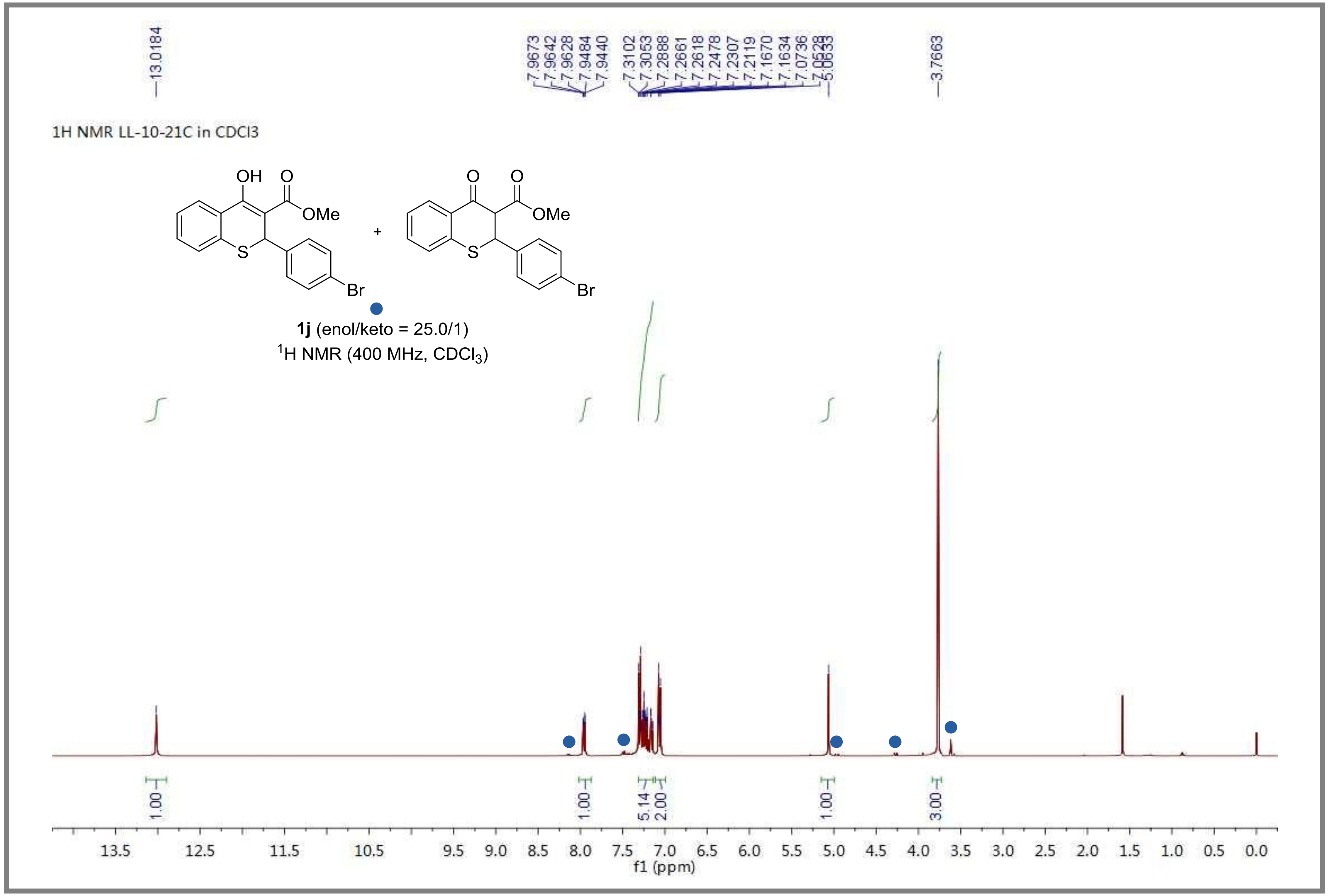
1H NMR LL-10-21C in CDCl3
OH
O
OMe
S
Br
+
O
O
OMe
S
Br
1j (enol/keto = 25.0/1)
1H NMR (400 MHz, CDCl3)
13.0184
7.9673
7.9642
7.9628
7.9484
7.9440
7.3102
7.3053
7.2888
7.2661
7.2618
7.2478
7.2307
7.2119
7.1670
7.1634
7.0736
3.7663
1.00
1.00
5.14
2.00
1.00
3.00
f1 (ppm)

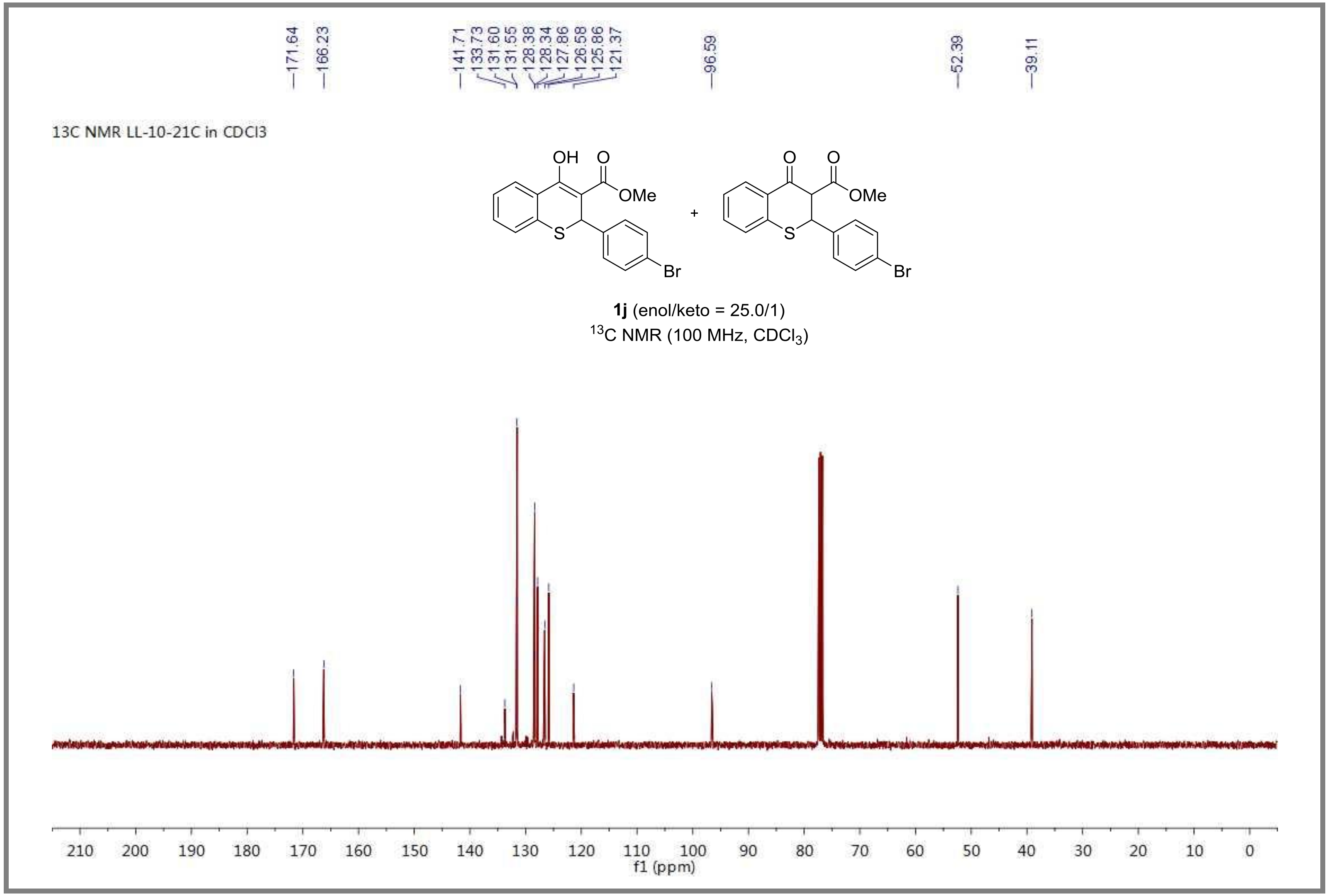
171.64
166.23
141.71
133.73
131.60
131.55
128.38
128.34
127.86
126.58
125.86
121.37
96.59
52.39
39.11
13C NMR LL-10-21C in CDCl3
OH
O
OMe
S
Br
+
O
O
OMe
S
Br
1j (enol/keto = 25.0/1)
13C NMR (100 MHz, CDCl3)
210 200 190 180 170 160 150 140 130 120 110 100 90 80 70 60 50 40 30 20 10 0
f1 (ppm)

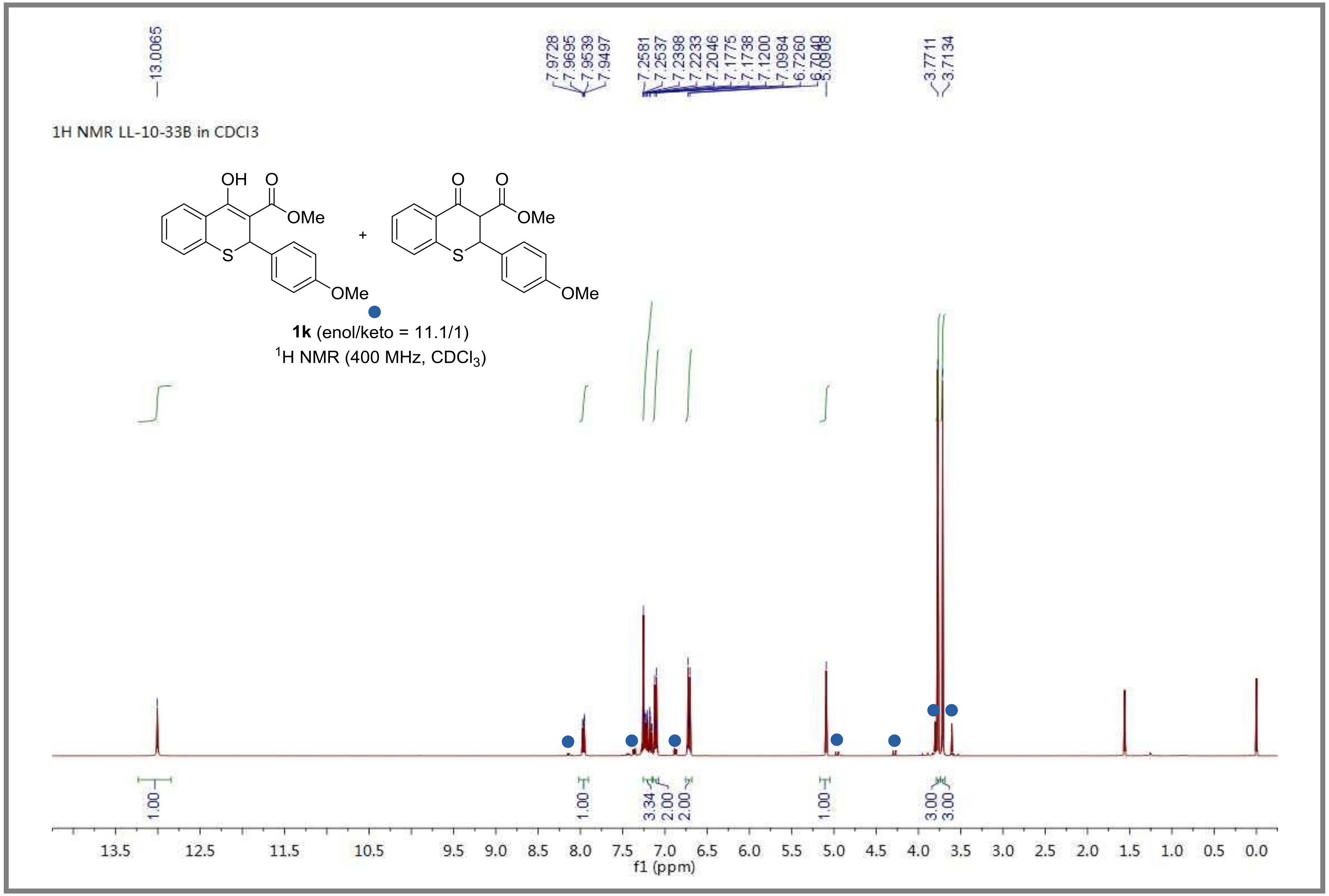
13.0065
7.9728
7.9695
7.9539
7.9497
7.2581
7.2537
7.2398
7.2233
7.2046
7.1775
7.1738
7.1200
7.0984
6.7260
5.0908
3.7711
3.7134
1H NMR LL-10-33B in CDCl3
OH
O
OMe
S
OMe
+
O
O
OMe
S
OMe
1k (enol/keto = 11.1/1)
^{1}H NMR (400 MHz, $CDCl_3$)
1.00
1.00
3.34
2.00
2.00
1.00
3.00
3.00
f1 (ppm)

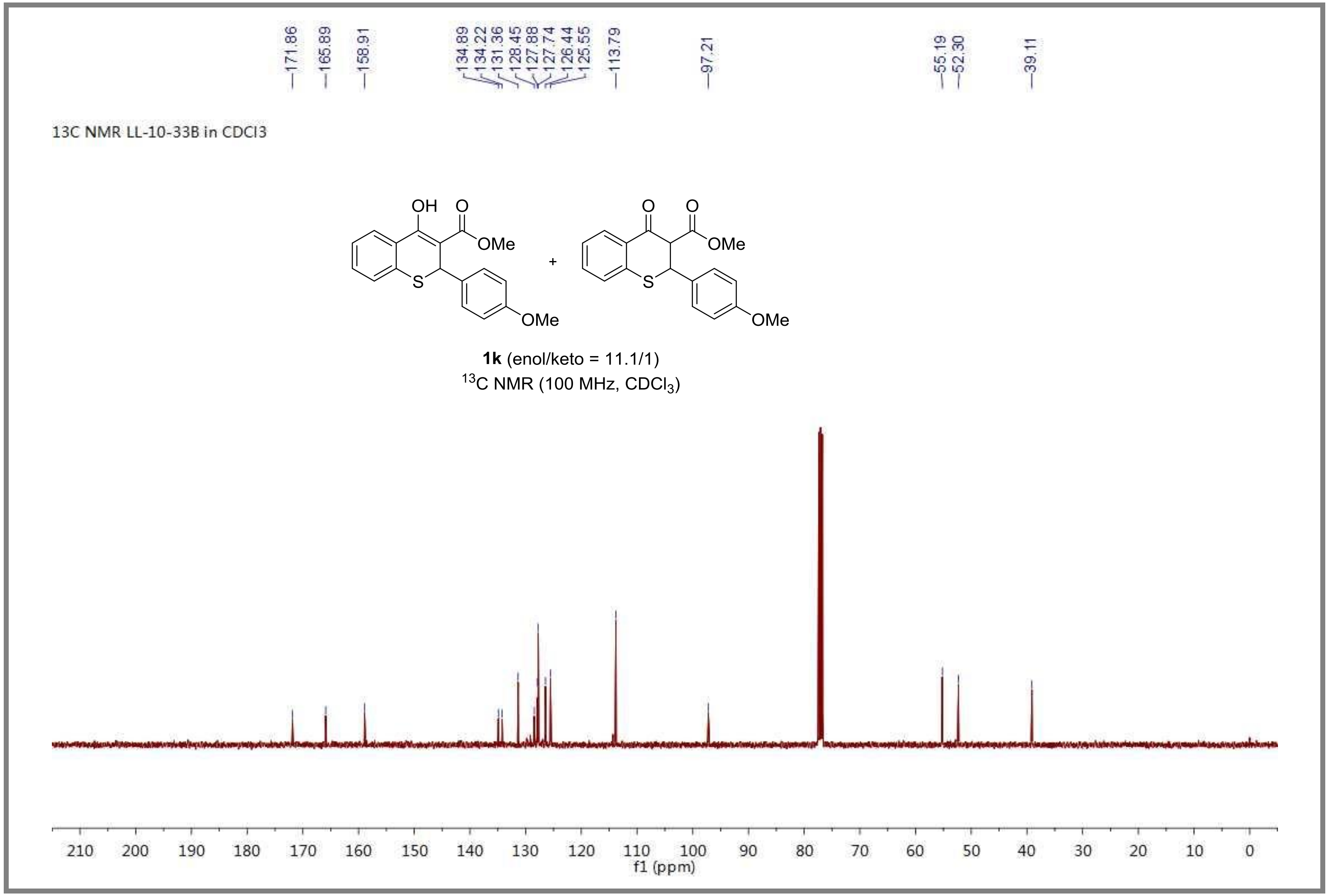

1k (enol/keto = 11.1/1)

^{13}C NMR (100 MHz, $CDCl_3$)

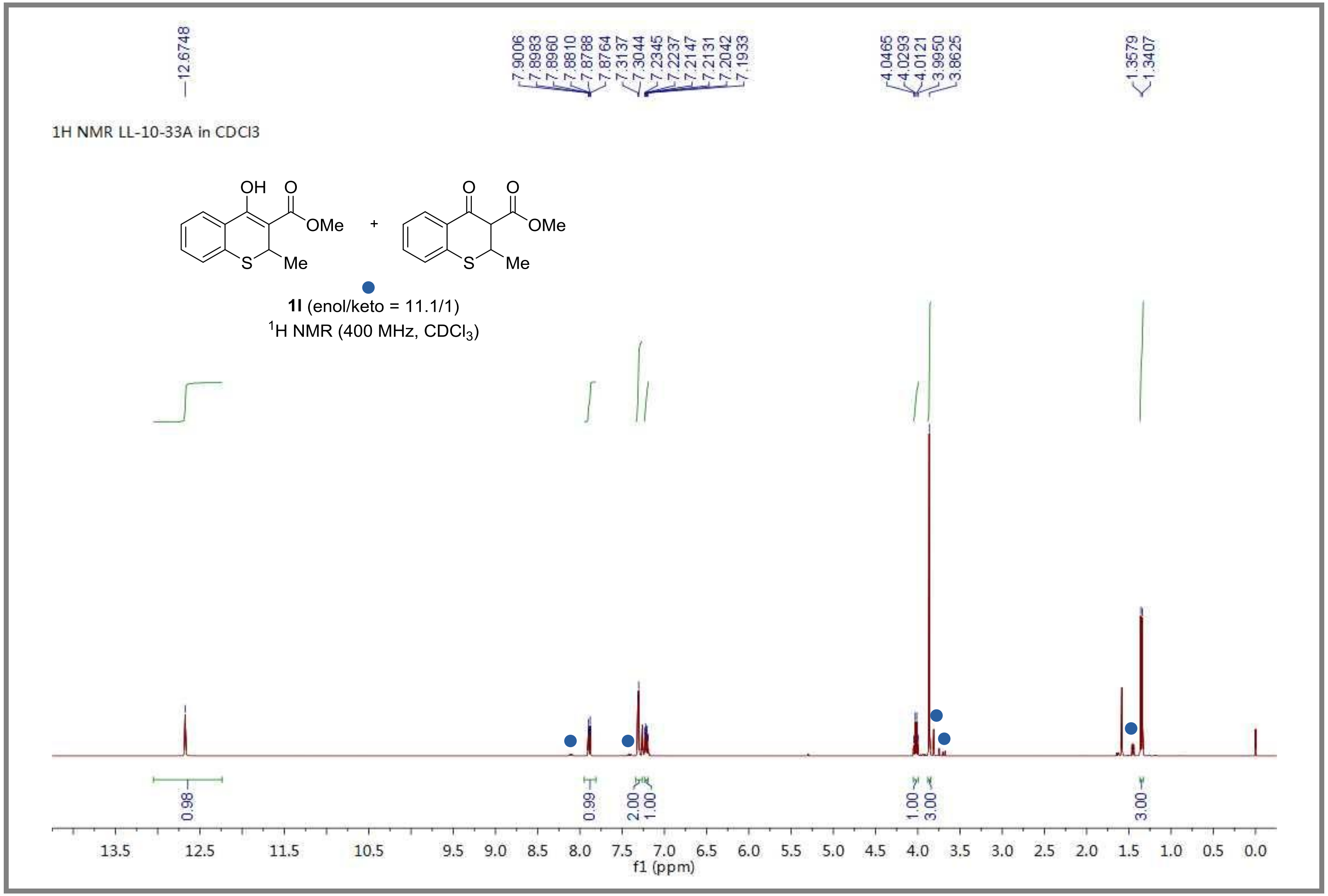

12.6748
7.9006
7.8983
7.8960
7.8810
7.8788
7.8764
7.3137
7.3044
7.2345
7.2237
7.2147
7.2131
7.2042
7.1933
4.0465
4.0293
4.0121
3.9950
3.8625
1.3579
1.3407
1H NMR LL-10-33A in CDCl3
OH
O
OMe
S
Me
+
O
O
OMe
S
Me
1l (enol/keto = 11.1/1)
1H NMR (400 MHz, CDCl3)
0.98
0.99
2.00
1.00
1.00
3.00
3.00
13.5
12.5
11.5
10.5
9.5
9.0
8.5
8.0
7.5
7.0
6.5
6.0
5.5
5.0
4.5
4.0
3.5
3.0
2.5
2.0
1.5
1.0
0.5
0.0
f1 (ppm)

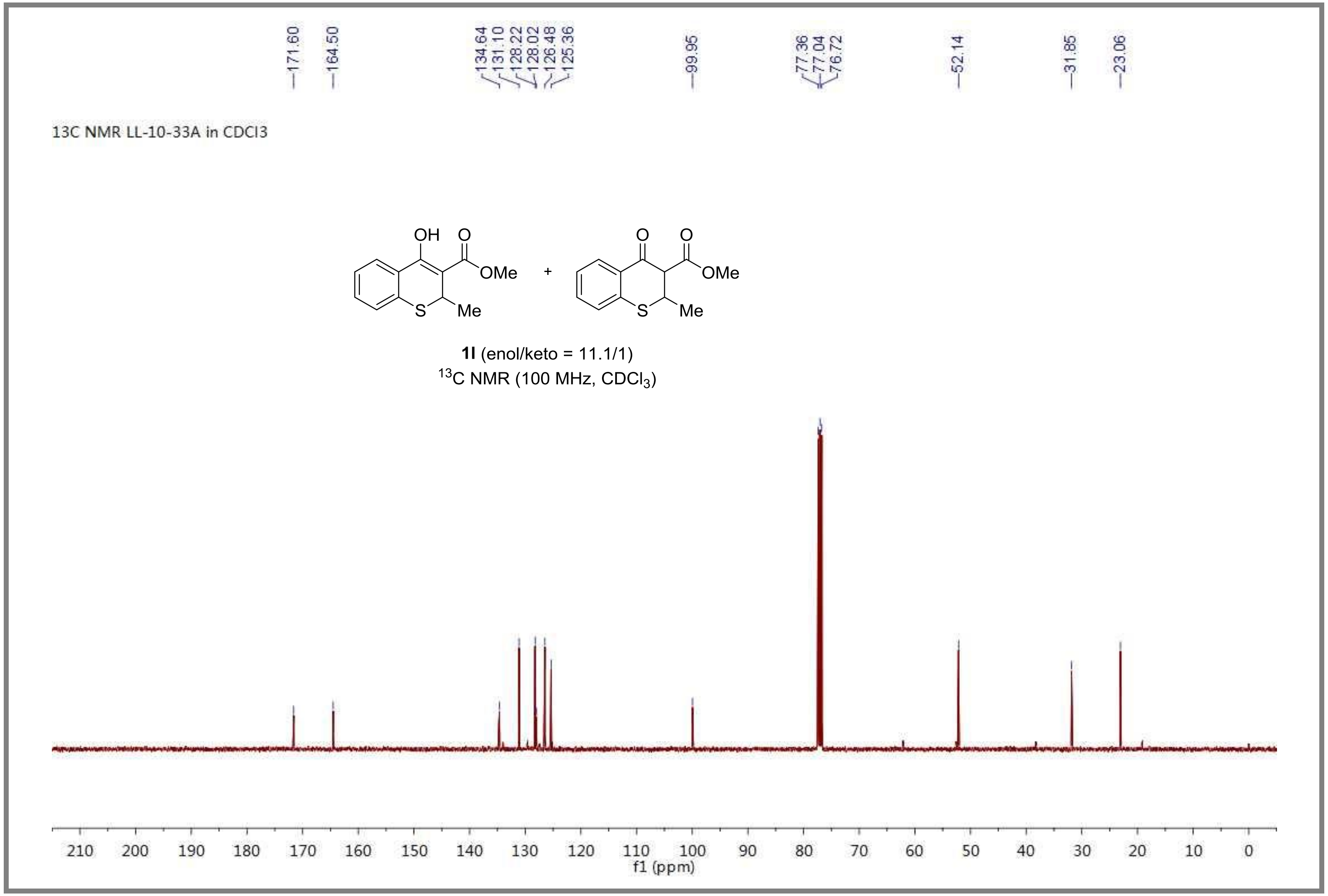
171.60
164.50
134.64
131.10
128.22
128.02
126.48
125.36
99.95
77.36
77.04
76.72
52.14
31.85
23.06
13C NMR LL-10-33A in CDCl3
OH
O
OMe
S
Me
+
O
O
OMe
S
Me
1l (enol/keto = 11.1/1)
13C NMR (100 MHz, CDCl3)
210 200 190 180 170 160 150 140 130 120 110 100 90 80 70 60 50 40 30 20 10 0
f1 (ppm)

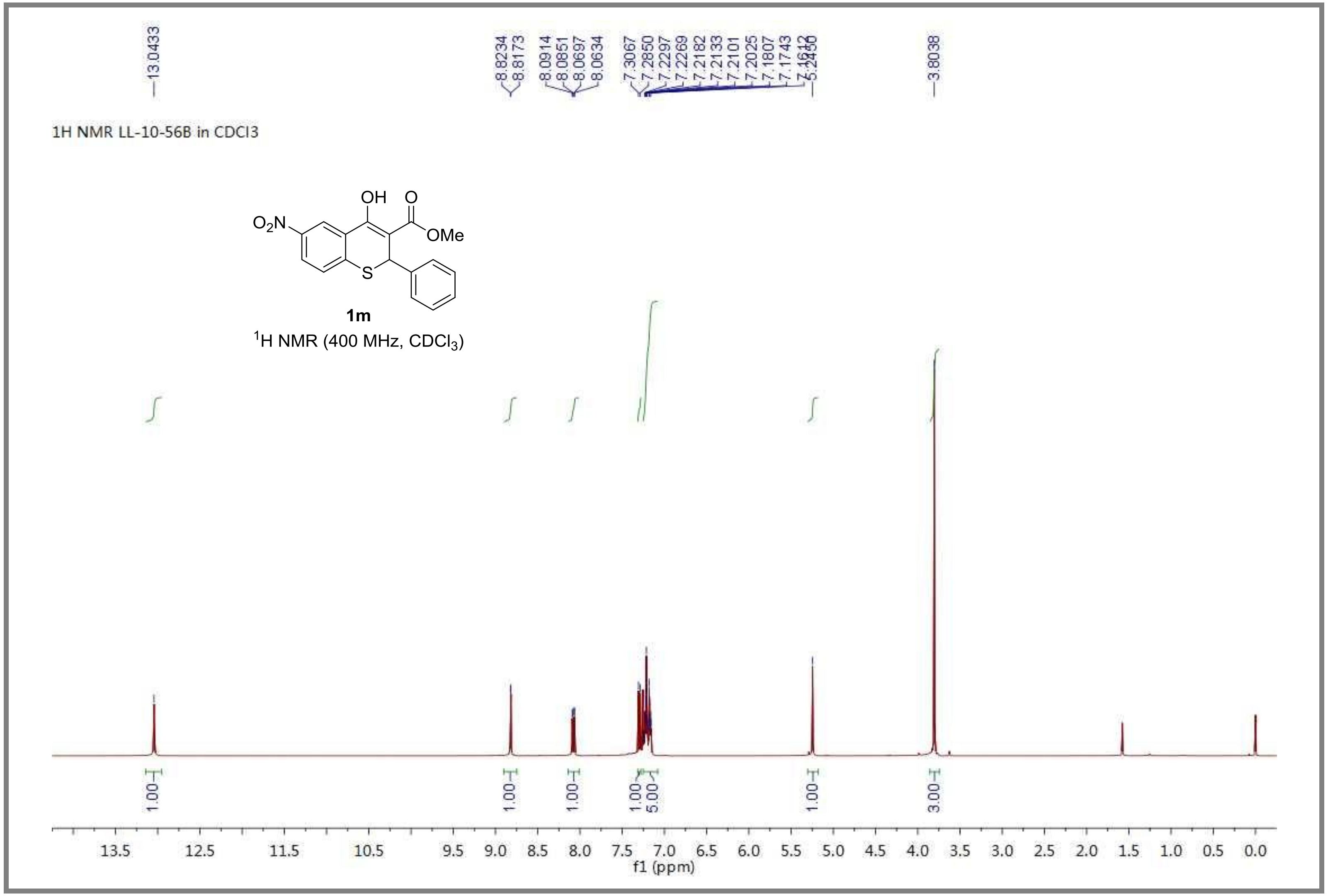
1H NMR LL-10-56B in CDCl3
1m
1H NMR (400 MHz, CDCl3)
f1 (ppm)

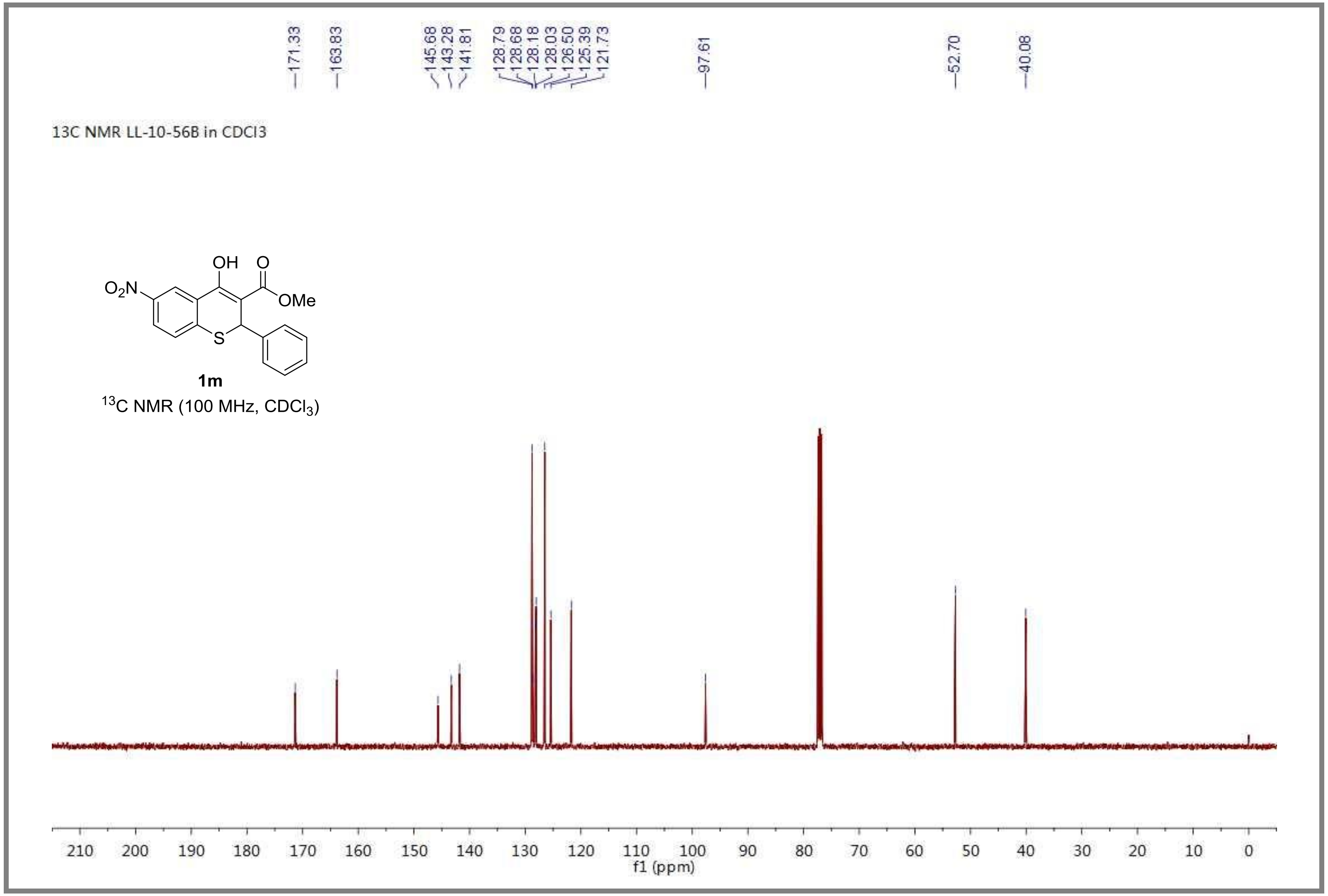

171.33
163.83
145.68
143.28
141.81
128.79
128.68
128.18
128.03
126.50
125.39
121.73
97.61
52.70
40.08
13C NMR LL-10-56B in CDCl3
OH
O
O_2N
OMe
S
1m
^{13}C NMR (100 MHz, $CDCl_3$)
210 200 190 180 170 160 150 140 130 120 110 100 90 80 70 60 50 40 30 20 10 0
f1 (ppm)

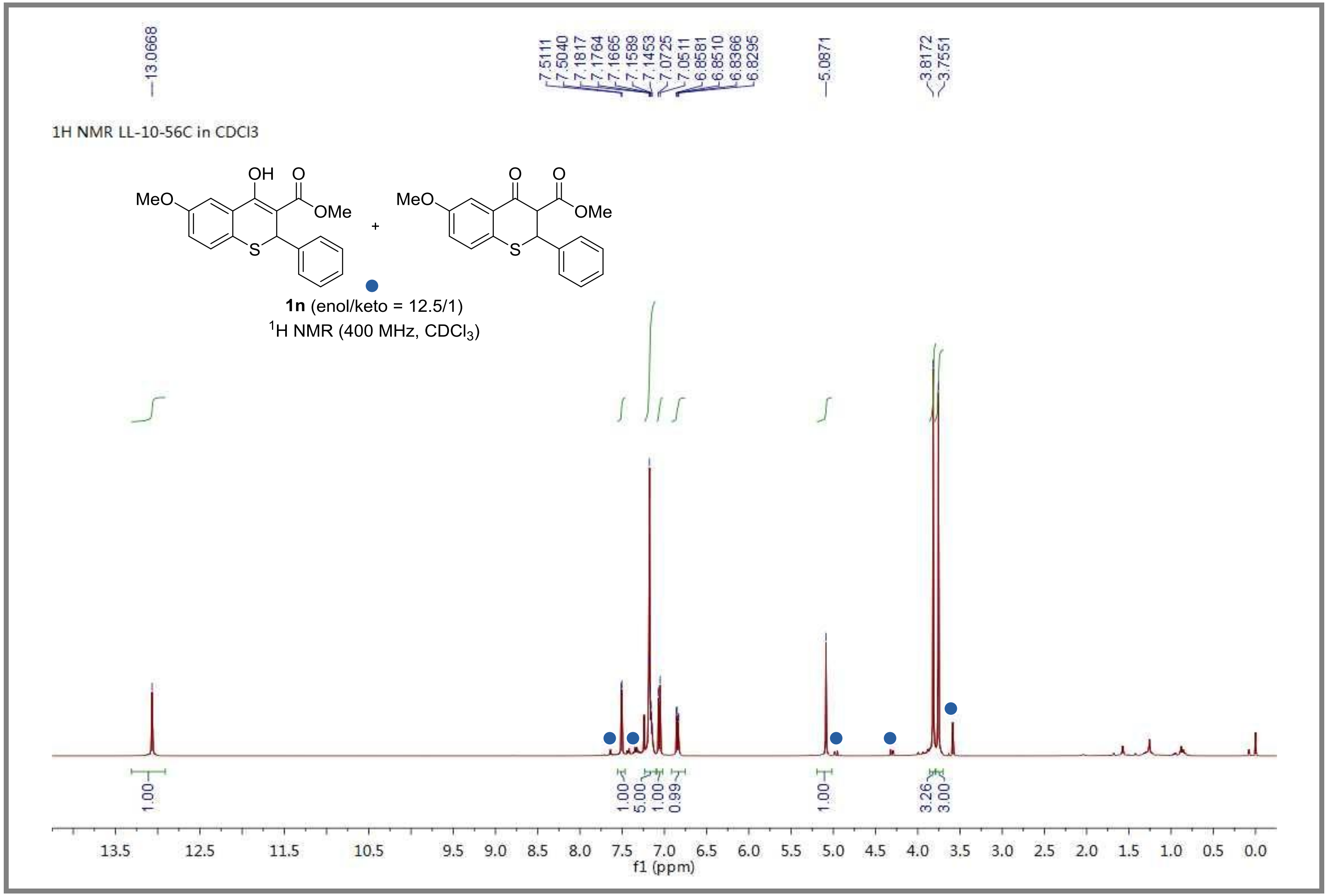

1H NMR LL-10-56C in CDCl3
1n (enol/keto = 12.5/1)
1H NMR (400 MHz, CDCl3)
13.0668
7.5111
7.5040
7.1817
7.1764
7.1665
7.1589
7.1453
7.0725
7.0511
6.8581
6.8510
6.8366
6.8295
5.0871
3.8172
3.7551
1.00
1.00
5.00
1.00
0.99
1.00
3.26
3.00
f1 (ppm)

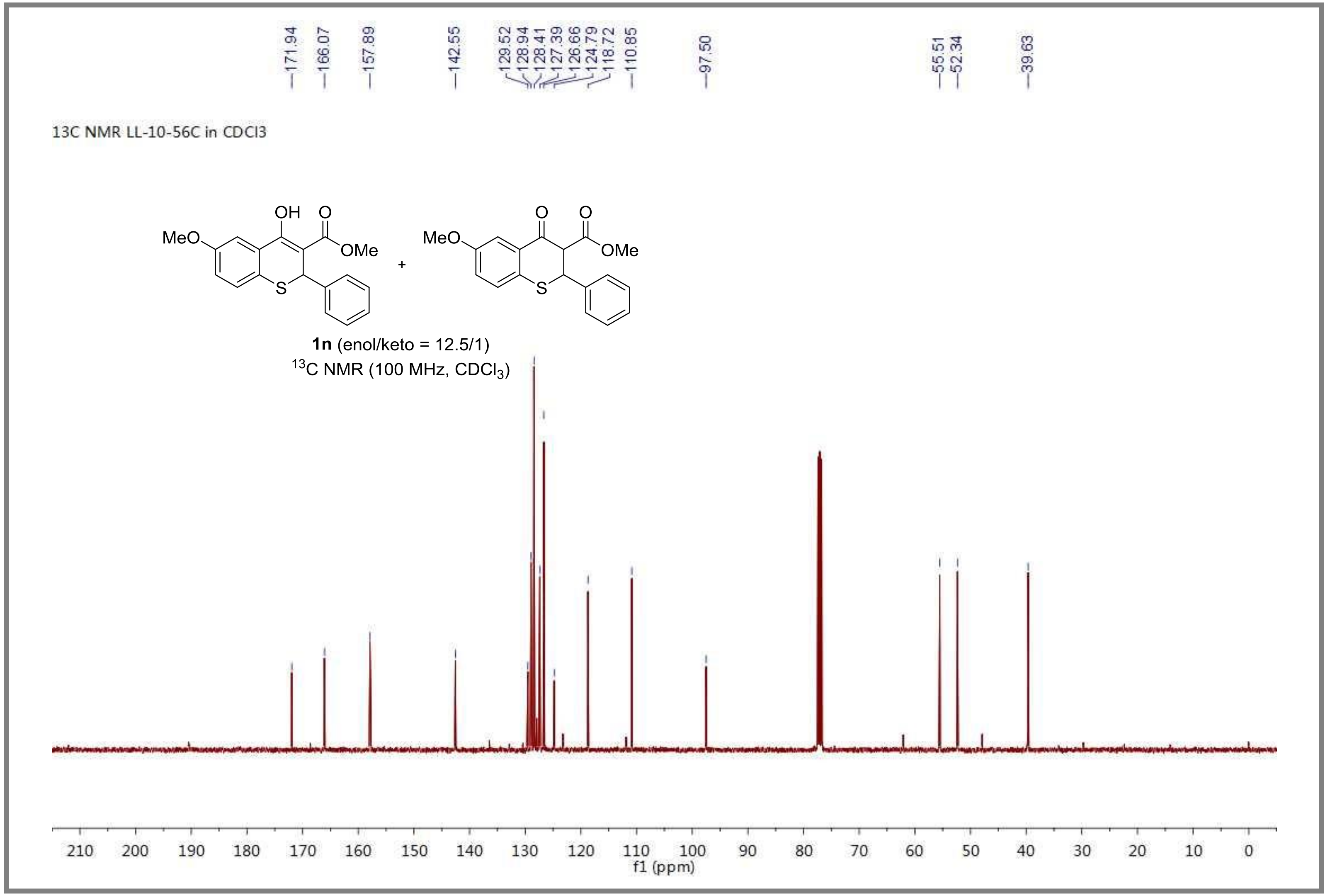
171.94
166.07
157.89
142.55
129.52
128.94
128.41
127.39
126.66
124.79
118.72
110.85
97.50
55.51
52.34
39.63
13C NMR LL-10-56C in CDCl3
OH
O
MeO
OMe
S
+
O
O
MeO
OMe
S
1n (enol/keto = 12.5/1)
13C NMR (100 MHz, CDCl3)
210 200 190 180 170 160 150 140 130 120 110 100 90 80 70 60 50 40 30 20 10 0
f1 (ppm)

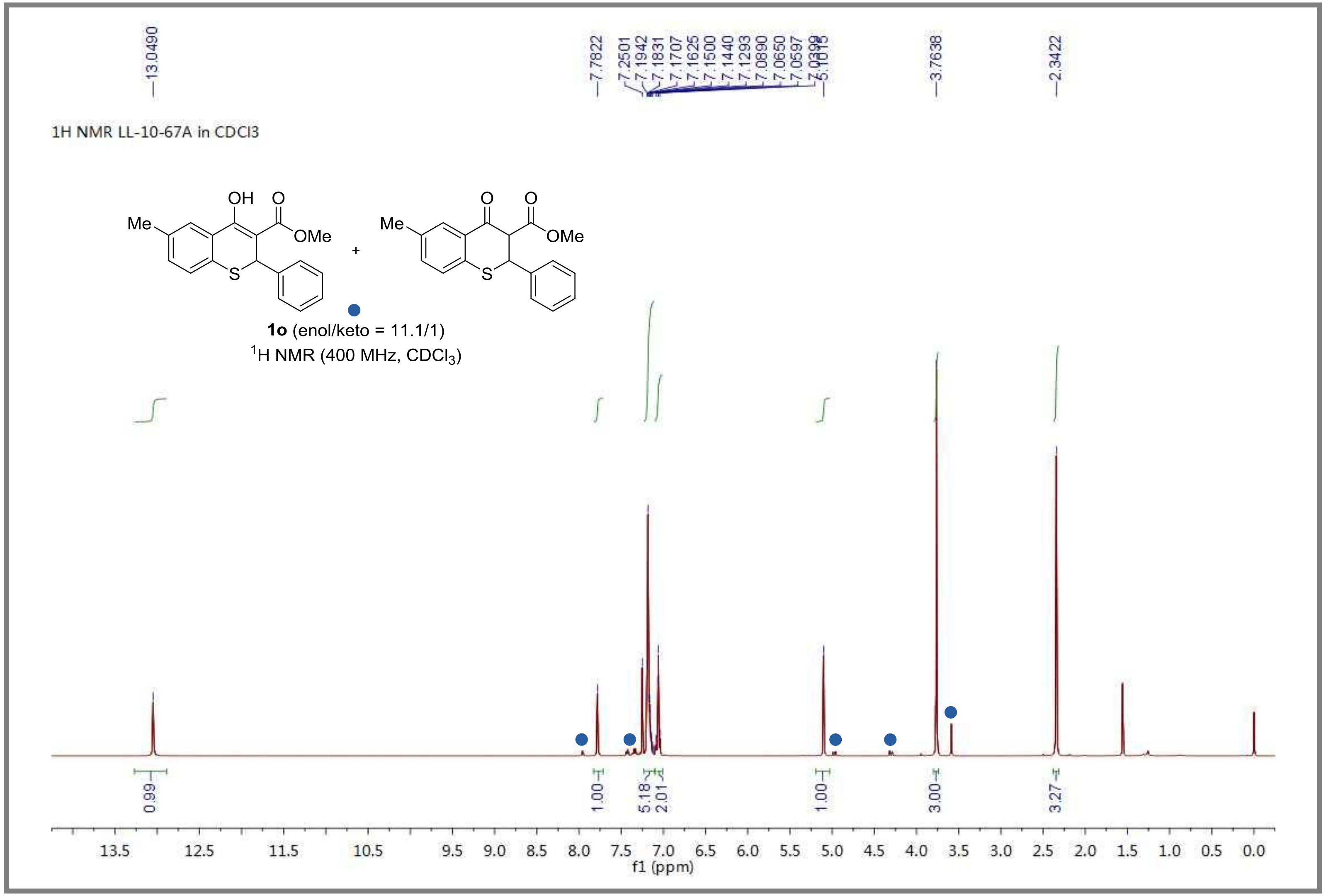
1H NMR LL-10-67A in CDCl3
13.0490
7.7822
7.2501
7.1942
7.1831
7.1707
7.1625
7.1500
7.1440
7.1293
7.0890
7.0650
7.0597
3.7638
2.3422
OH
O
OMe
Me
S
+
O
O
OMe
Me
S
1o (enol/keto = 11.1/1)
1H NMR (400 MHz, CDCl3)
0.99
1.00
5.18
2.01
1.00
3.00
3.27
13.5 12.5 11.5 10.5 9.5 9.0 8.5 8.0 7.5 7.0 6.5 6.0 5.5 5.0 4.5 4.0 3.5 3.0 2.5 2.0 1.5 1.0 0.5 0.0
f1 (ppm)

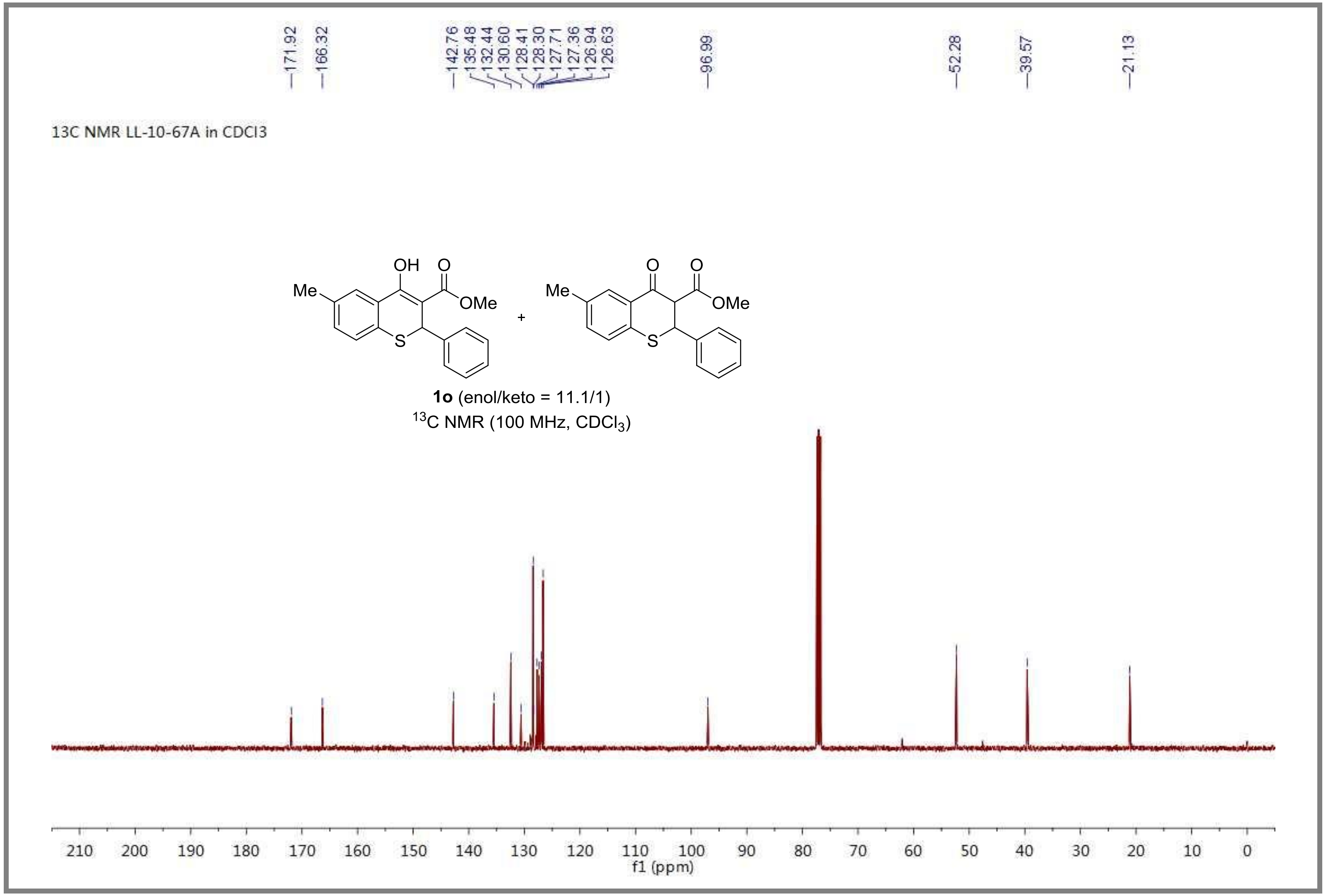
171.92
166.32
142.76
135.48
132.44
130.60
128.41
128.30
127.71
127.36
126.94
126.63
96.99
52.28
39.57
21.13
13C NMR LL-10-67A in CDCl3
OH
O
Me
OMe
S
+
O
O
Me
OMe
S
1o (enol/keto = 11.1/1)
13C NMR (100 MHz, CDCl3)
210 200 190 180 170 160 150 140 130 120 110 100 90 80 70 60 50 40 30 20 10 0
f1 (ppm)

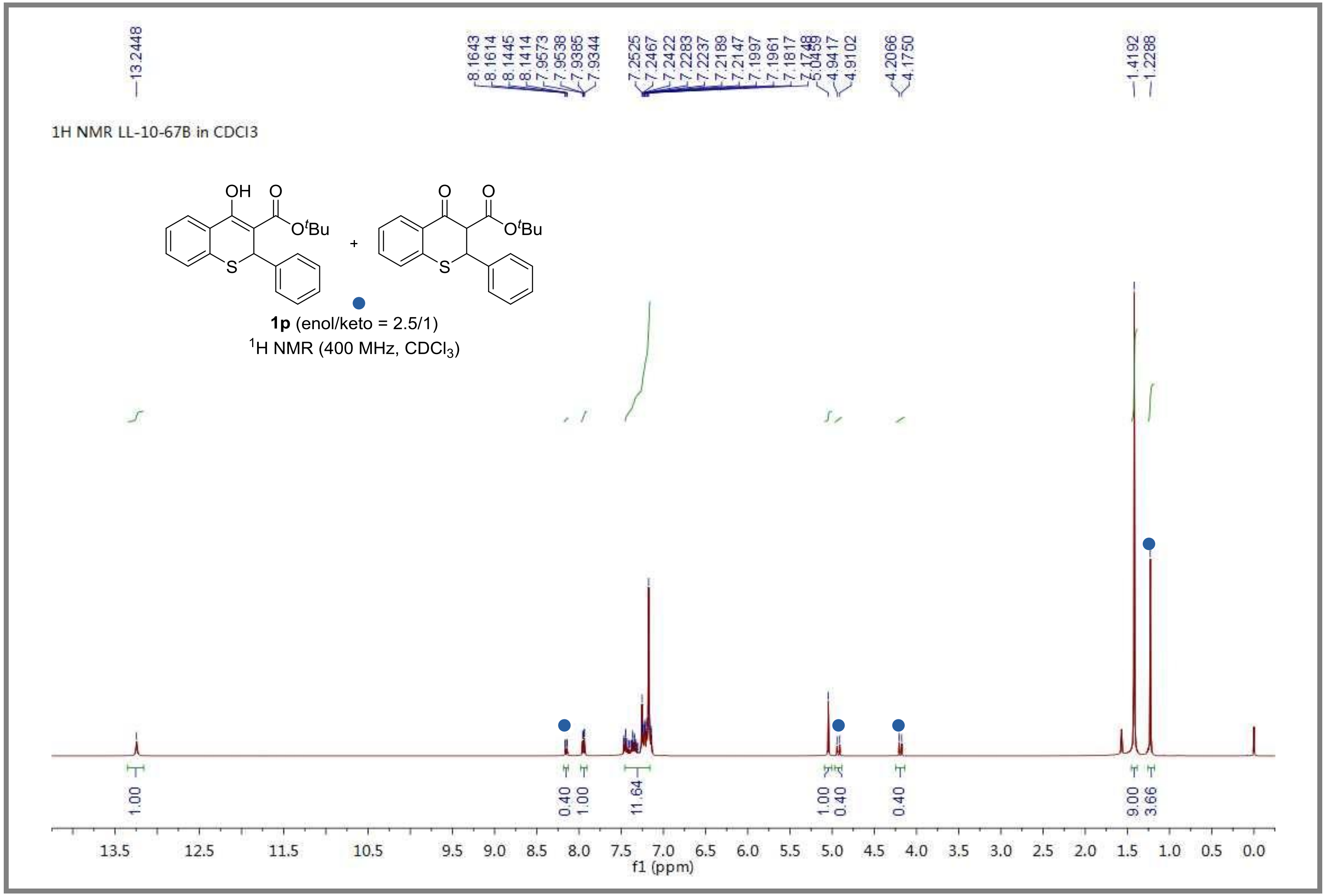
1H NMR LL-10-67B in CDCl3
1p (enol/keto = 2.5/1)
1H NMR (400 MHz, CDCl3)
f1 (ppm)

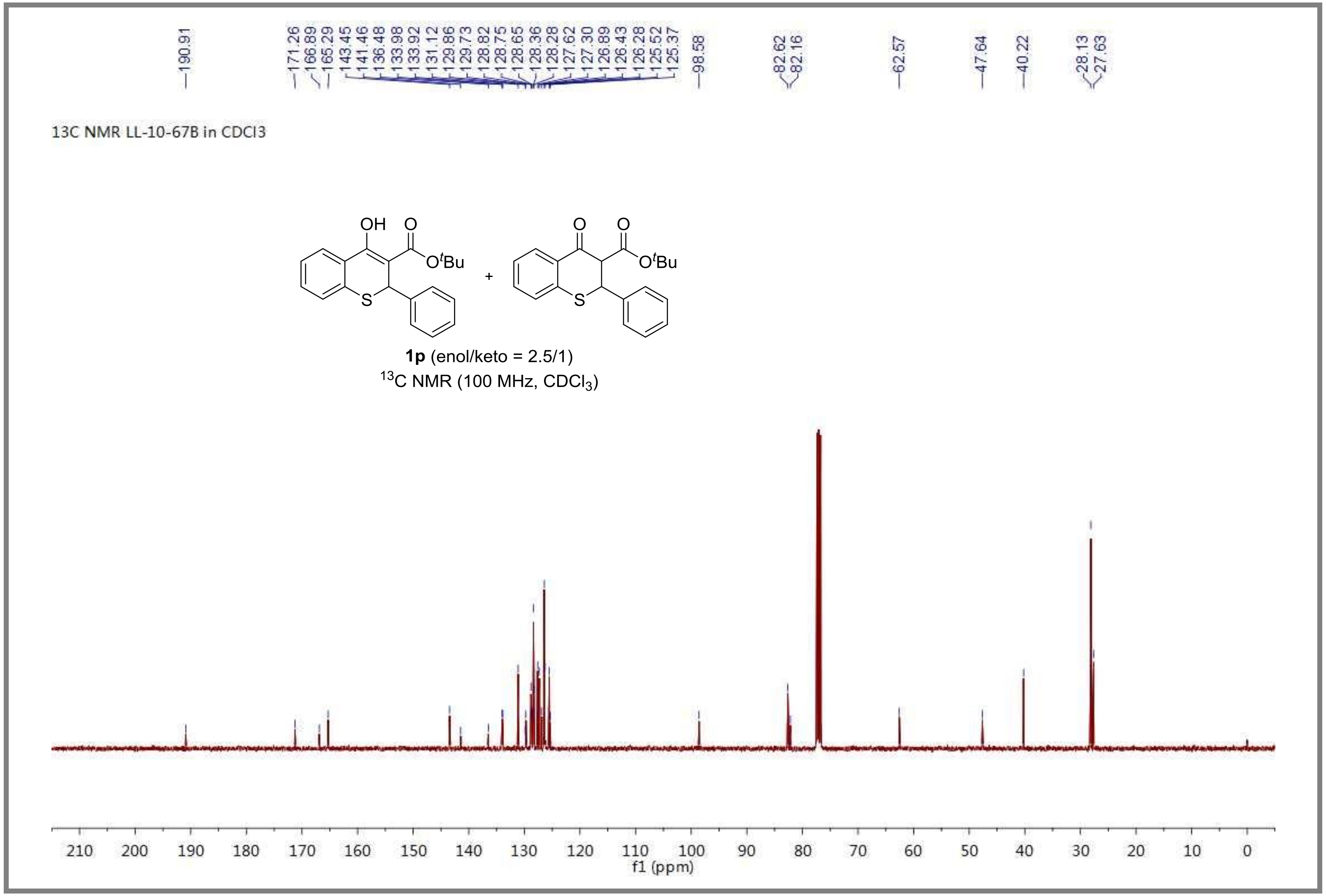
190.91
171.26
166.89
165.29
143.45
141.46
136.48
133.98
133.92
131.12
129.86
129.73
128.82
128.75
128.65
128.36
128.28
127.62
127.30
126.89
126.43
126.28
125.52
125.37
98.58
82.62
82.16
62.57
47.64
40.22
28.13
27.63
13C NMR LL-10-67B in CDCl3
OH
O
O^tBu
S
+
O
O
O^tBu
S
1p (enol/keto = 2.5/1)
13C NMR (100 MHz, CDCl3)
210 200 190 180 170 160 150 140 130 120 110 100 90 80 70 60 50 40 30 20 10 0
f1 (ppm)

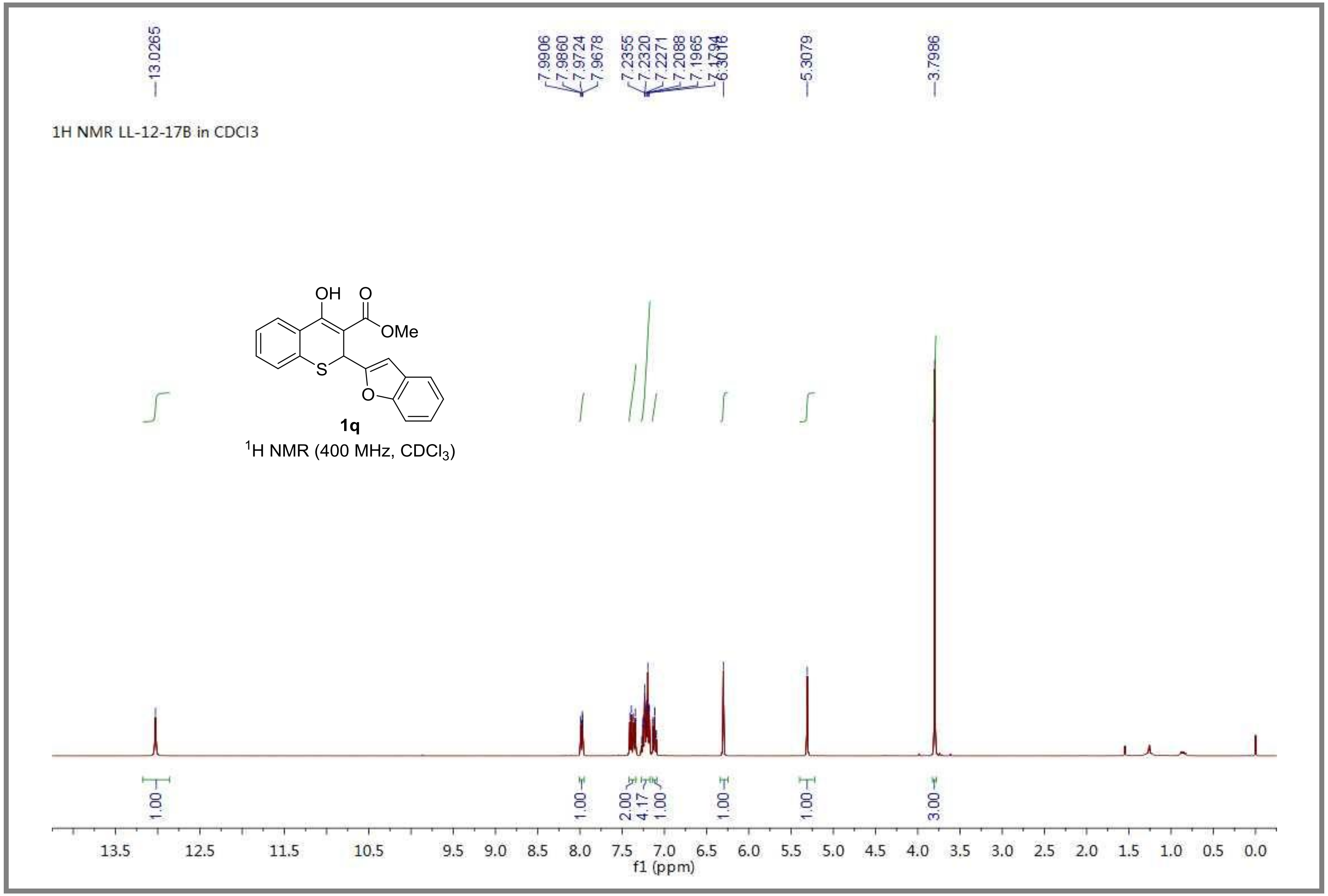
1H NMR LL-12-17B in CDCl3
13.0265
7.9906
7.9860
7.9724
7.9678
7.2355
7.2320
7.2271
7.2088
7.1965
5.3079
3.7986
OH
O
OMe
S
O
1q
^{1}H NMR (400 MHz, CDCl$_3$)
1.00
1.00
2.00
4.17
1.00
1.00
1.00
3.00
13.5
12.5
11.5
10.5
9.5
9.0
8.5
8.0
7.5
7.0
6.5
6.0
5.5
5.0
4.5
4.0
3.5
3.0
2.5
2.0
1.5
1.0
0.5
0.0
f1 (ppm)

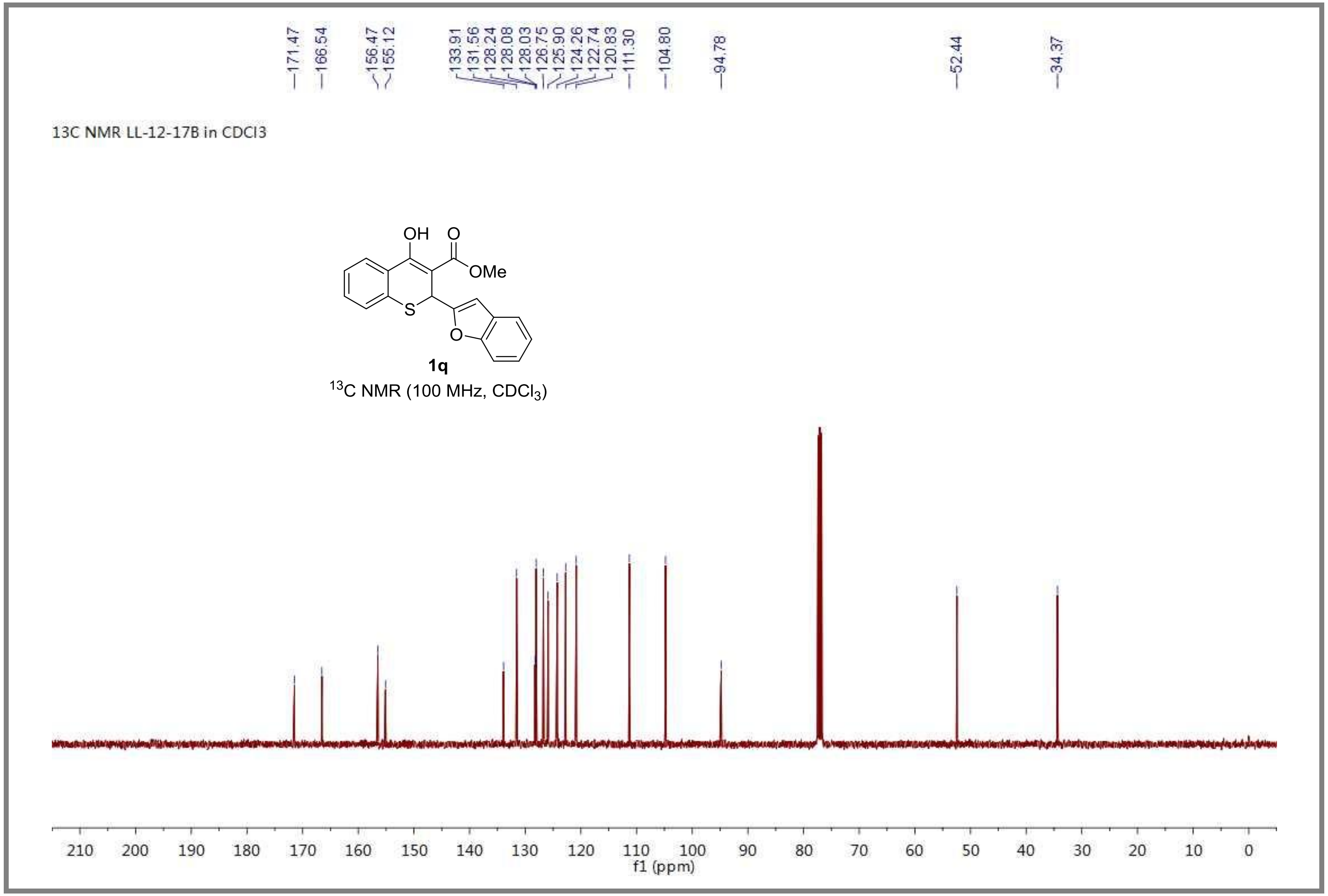

171.47
166.54
156.47
155.12
133.91
131.56
128.24
128.08
128.03
126.75
125.90
124.26
122.74
120.83
111.30
104.80
94.78
52.44
34.37
13C NMR LL-12-17B in CDCl3
OH
O
OMe
S
O
1q
13C NMR (100 MHz, CDCl3)
210
200
190
180
170
160
150
140
130
120
110
100
90
80
70
60
50
40
30
20
10
0
f1 (ppm)

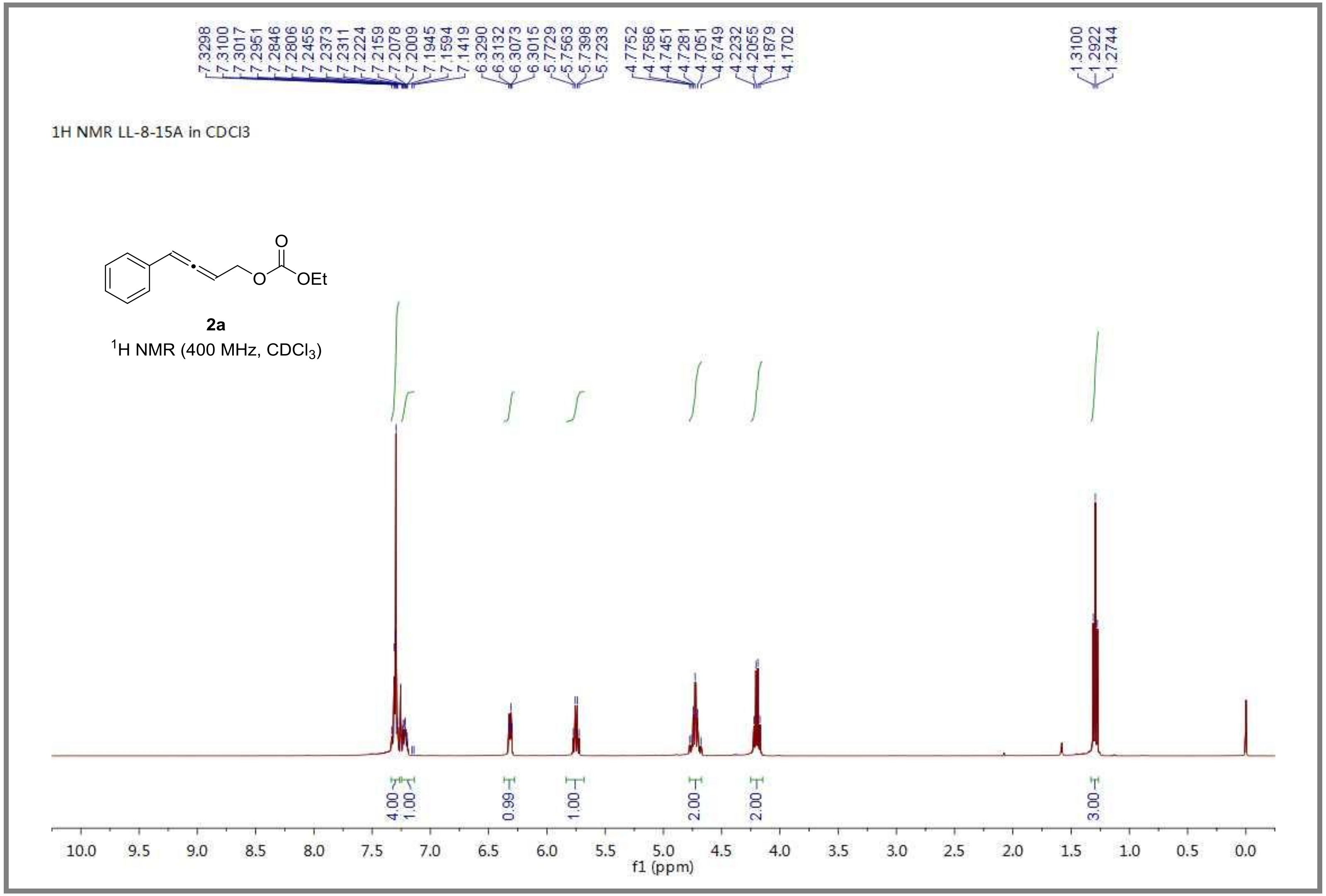

1H NMR LL-8-15A in CDCl3
2a
^{1}H NMR (400 MHz, CDCl$_3$)
OEt
O
f1 (ppm)

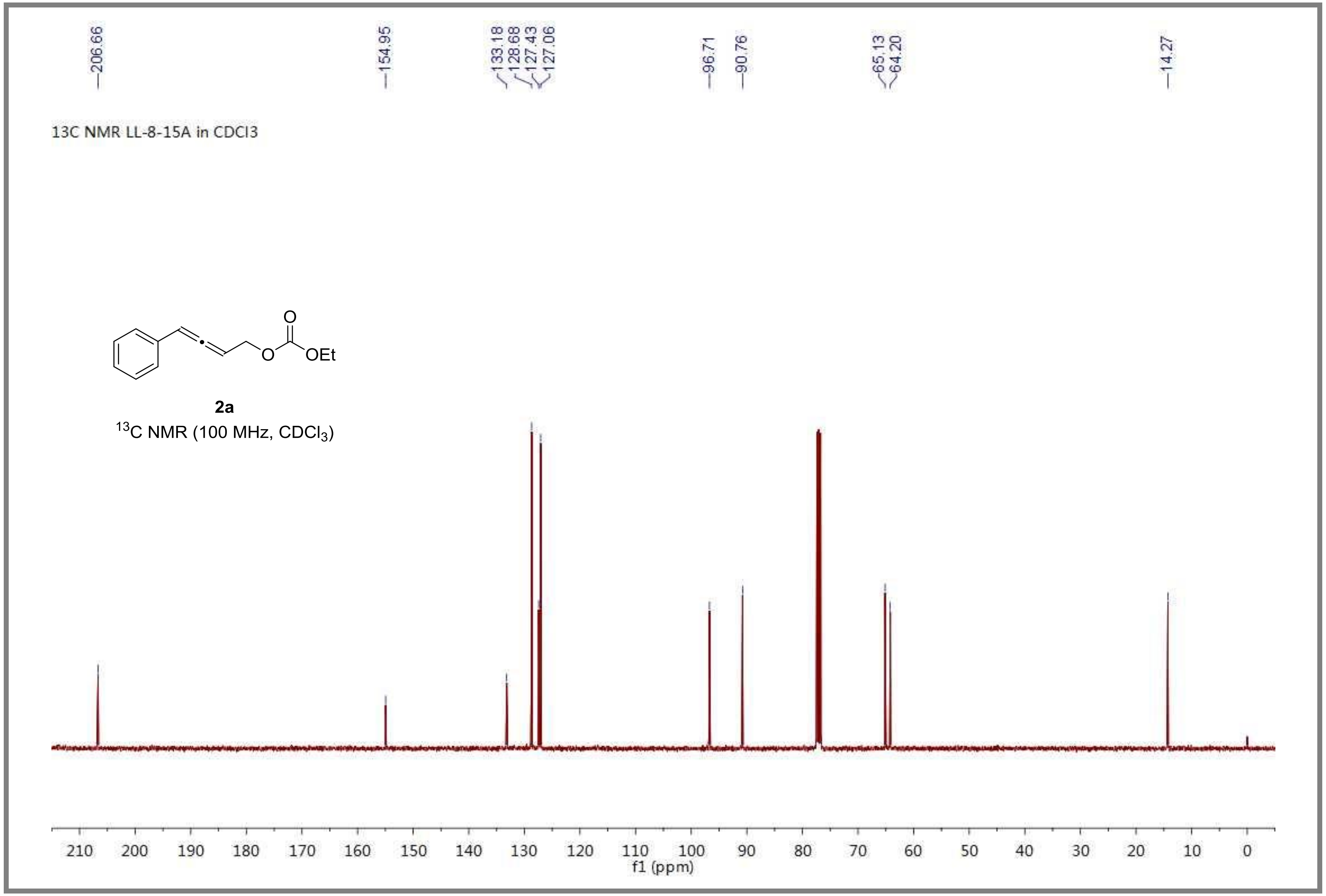

206.66
154.95
133.18
128.68
127.43
127.06
96.71
90.76
65.13
64.20
14.27
13C NMR LL-8-15A in CDCl3
O
O
OEt
2a
13C NMR (100 MHz, CDCl3)
210
200
190
180
170
160
150
140
130
120
110
100
90
80
70
60
50
40
30
20
10
0
f1 (ppm)

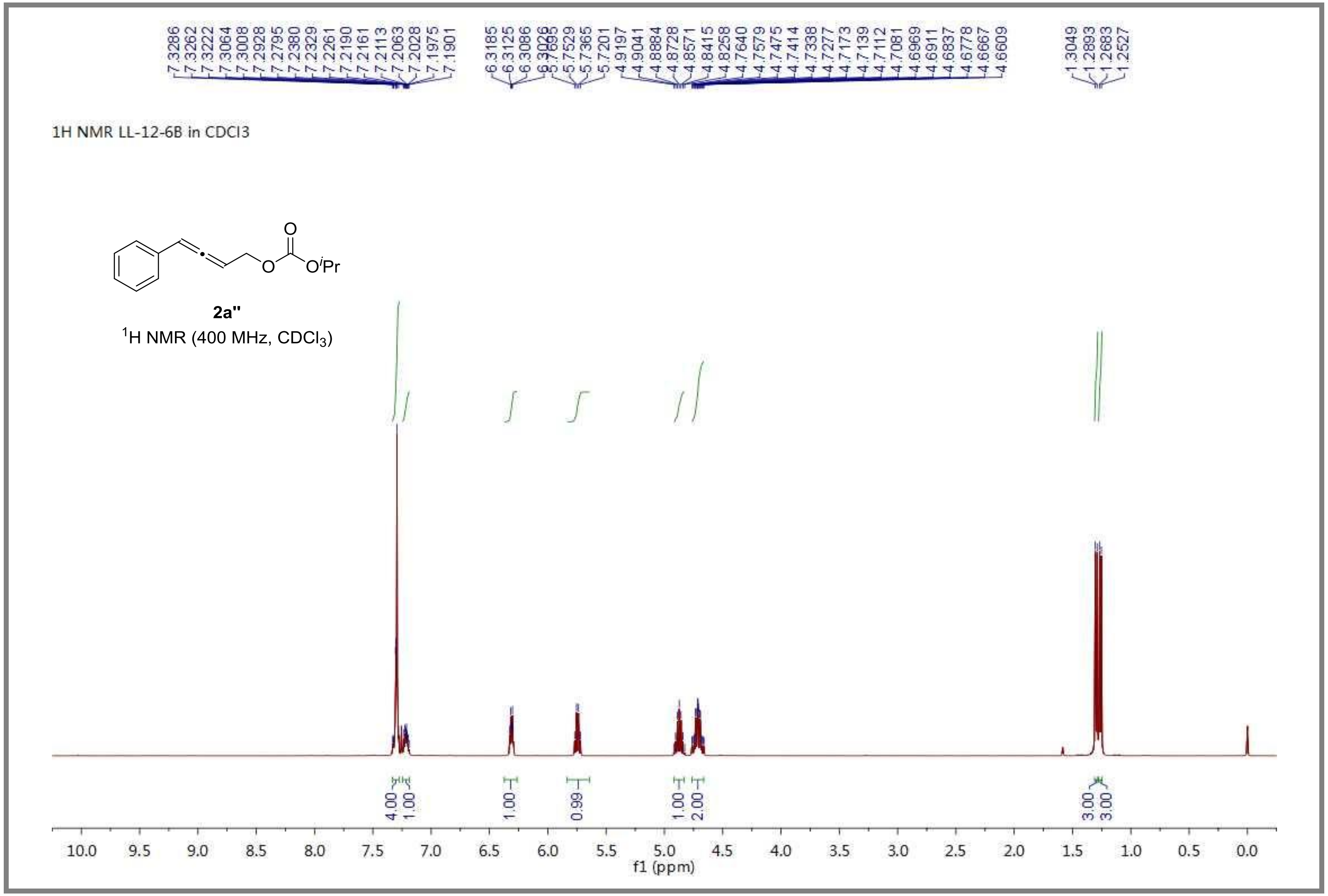

1H NMR LL-12-6B in CDCl3
2a''
^{1}H NMR (400 MHz, CDCl$_3$)
f1 (ppm)

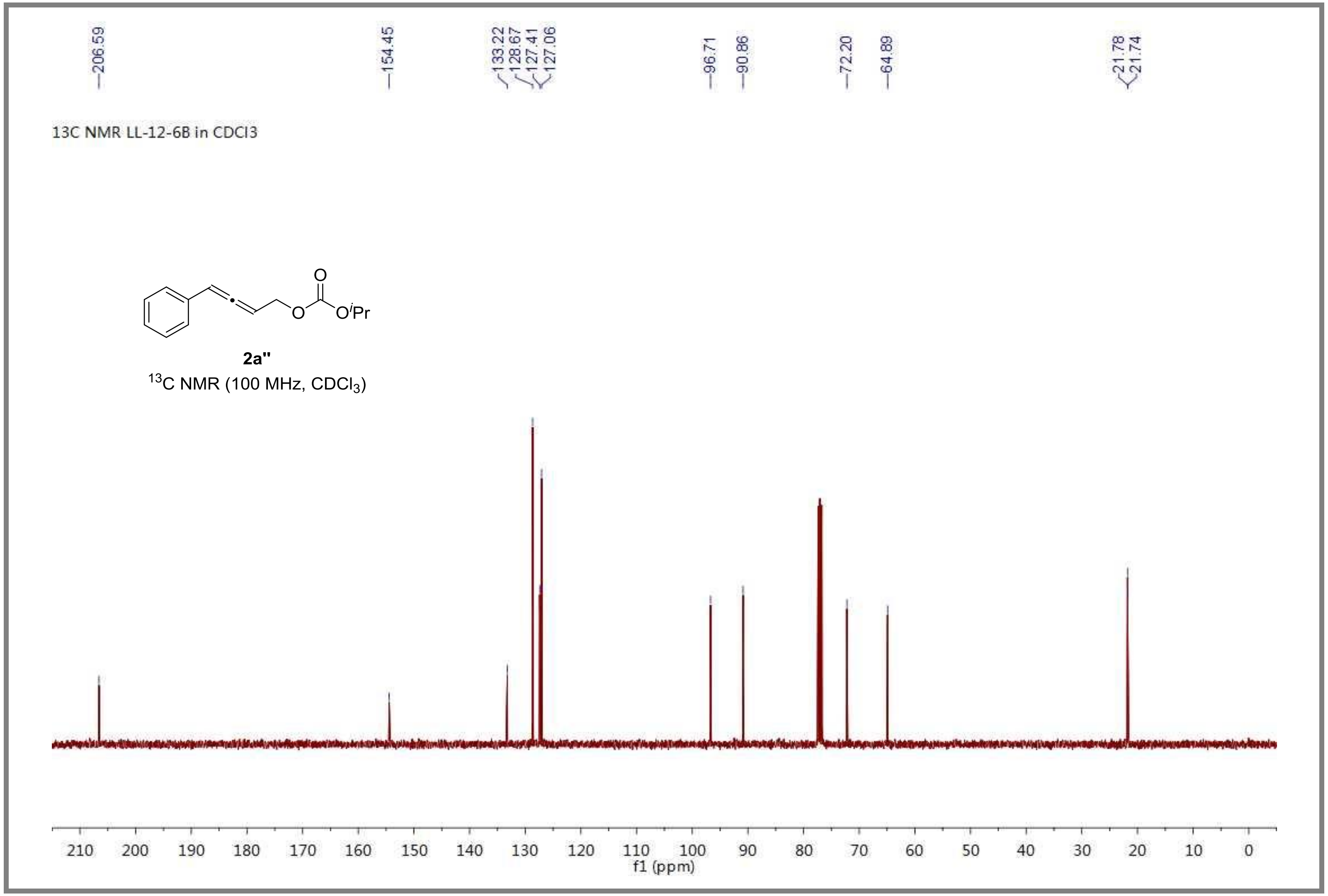

206.59
154.45
133.22
128.67
127.41
127.06
96.71
90.86
72.20
64.89
21.78
21.74
13C NMR LL-12-6B in CDCl3
O
O
O^{i}Pr
2a''
^{13}C NMR (100 MHz, $CDCl_3$)
210
200
190
180
170
160
150
140
130
120
110
100
90
80
70
60
50
40
30
20
10
0
f1 (ppm)

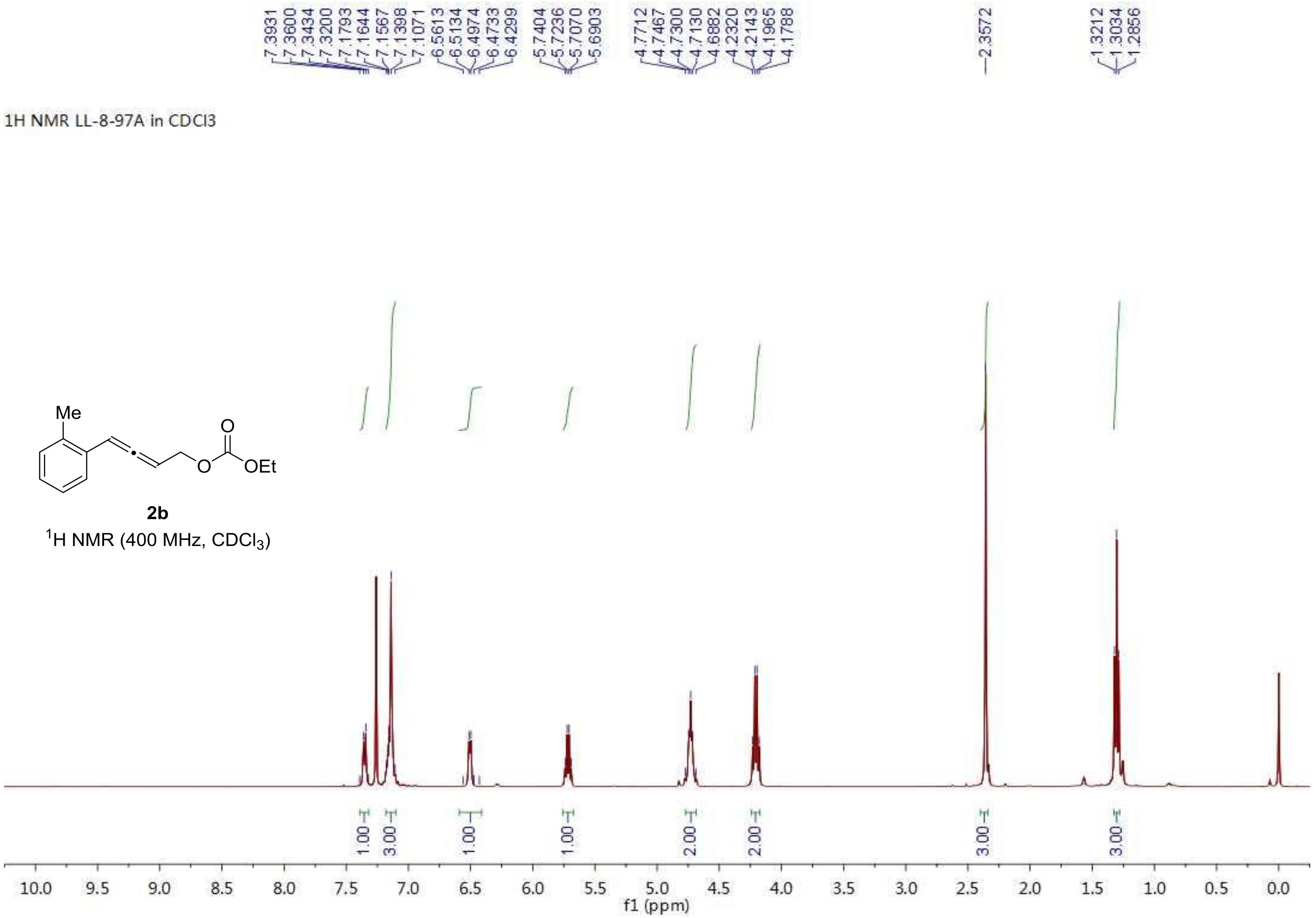

2b

^{1}H NMR (400 MHz, $CDCl_3$)

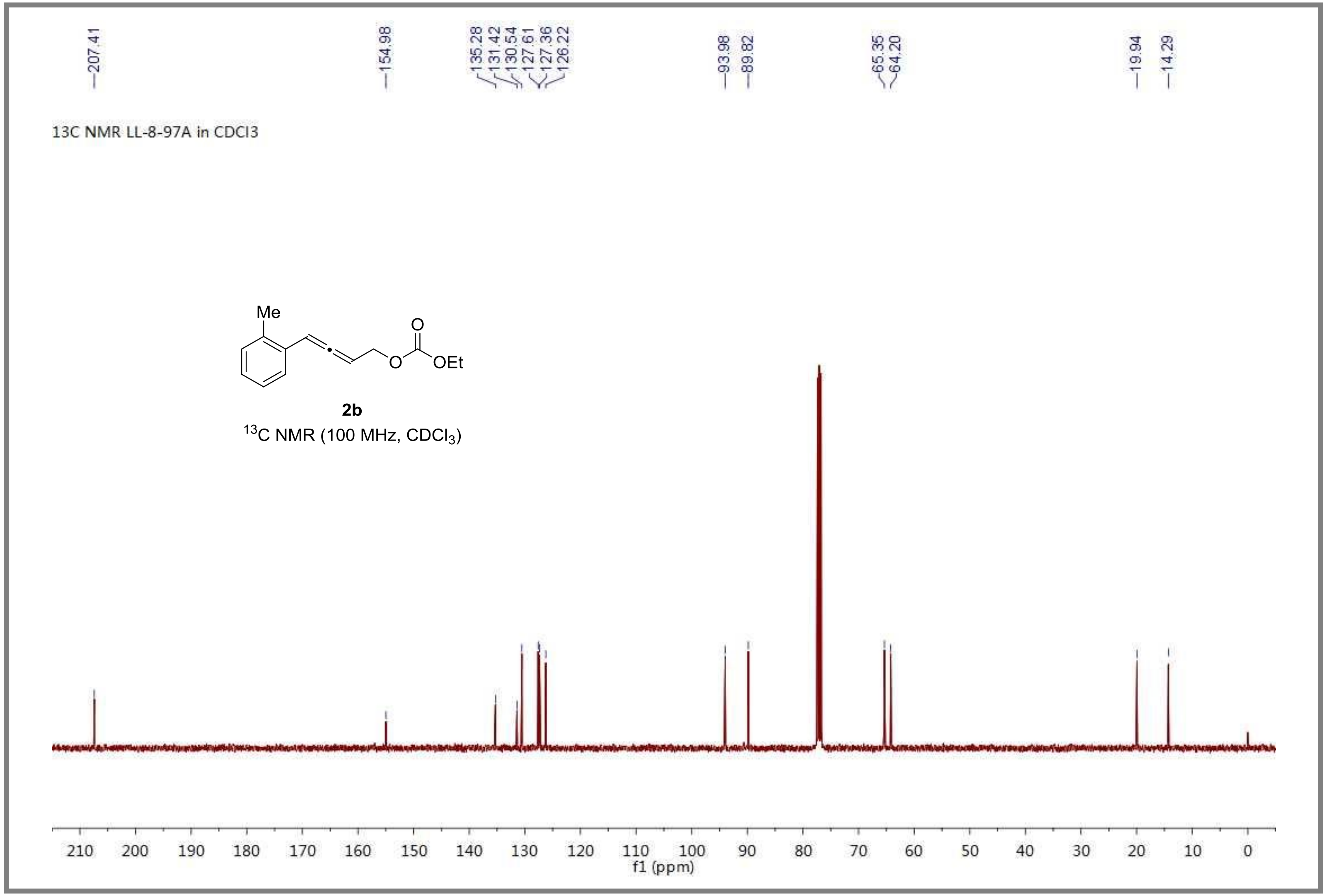

207.41
154.98
135.28
131.42
130.54
127.61
127.36
126.22
93.98
89.82
65.35
64.20
19.94
14.29
13C NMR LL-8-97A in CDCl3
Me
O
O
OEt
2b
^{13}C NMR (100 MHz, $CDCl_3$)
210 200 190 180 170 160 150 140 130 120 110 100 90 80 70 60 50 40 30 20 10 0
f1 (ppm)

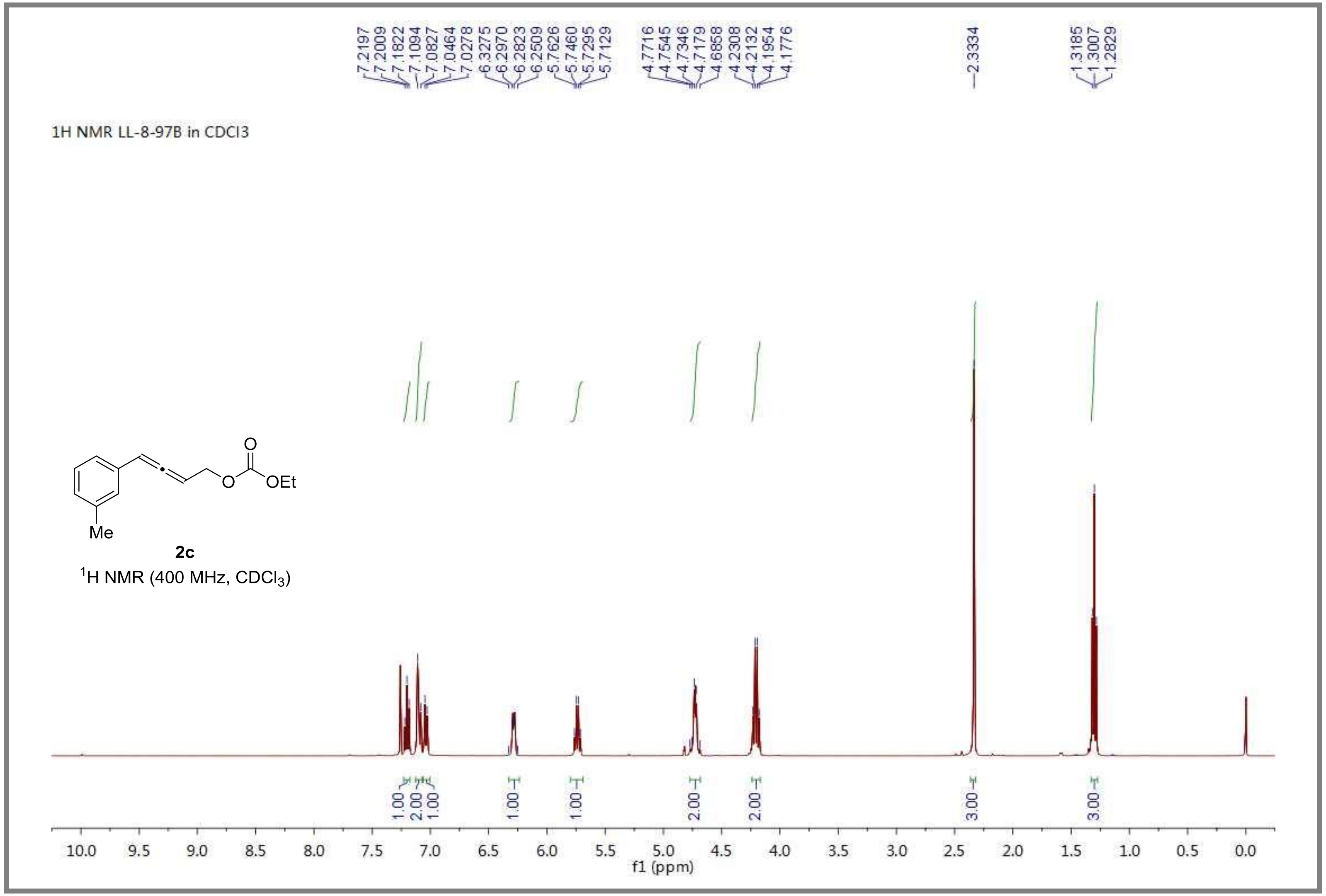
1H NMR LL-8-97B in CDCl3
7.2197
7.2009
7.1822
7.1094
7.0827
7.0464
7.0278
6.3275
6.2970
6.2823
6.2509
5.7626
5.7460
5.7295
5.7129
4.7716
4.7545
4.7346
4.7179
4.6858
4.2308
4.2132
4.1954
4.1776
2.3334
1.3185
1.3007
1.2829
O
O
OEt
Me
2c
^{1}H NMR (400 MHz, $CDCl_3$)
1.00
2.00
1.00
1.00
1.00
2.00
2.00
3.00
3.00
10.0
9.5
9.0
8.5
8.0
7.5
7.0
6.5
6.0
5.5
5.0
4.5
4.0
3.5
3.0
2.5
2.0
1.5
1.0
0.5
0.0
f1 (ppm)

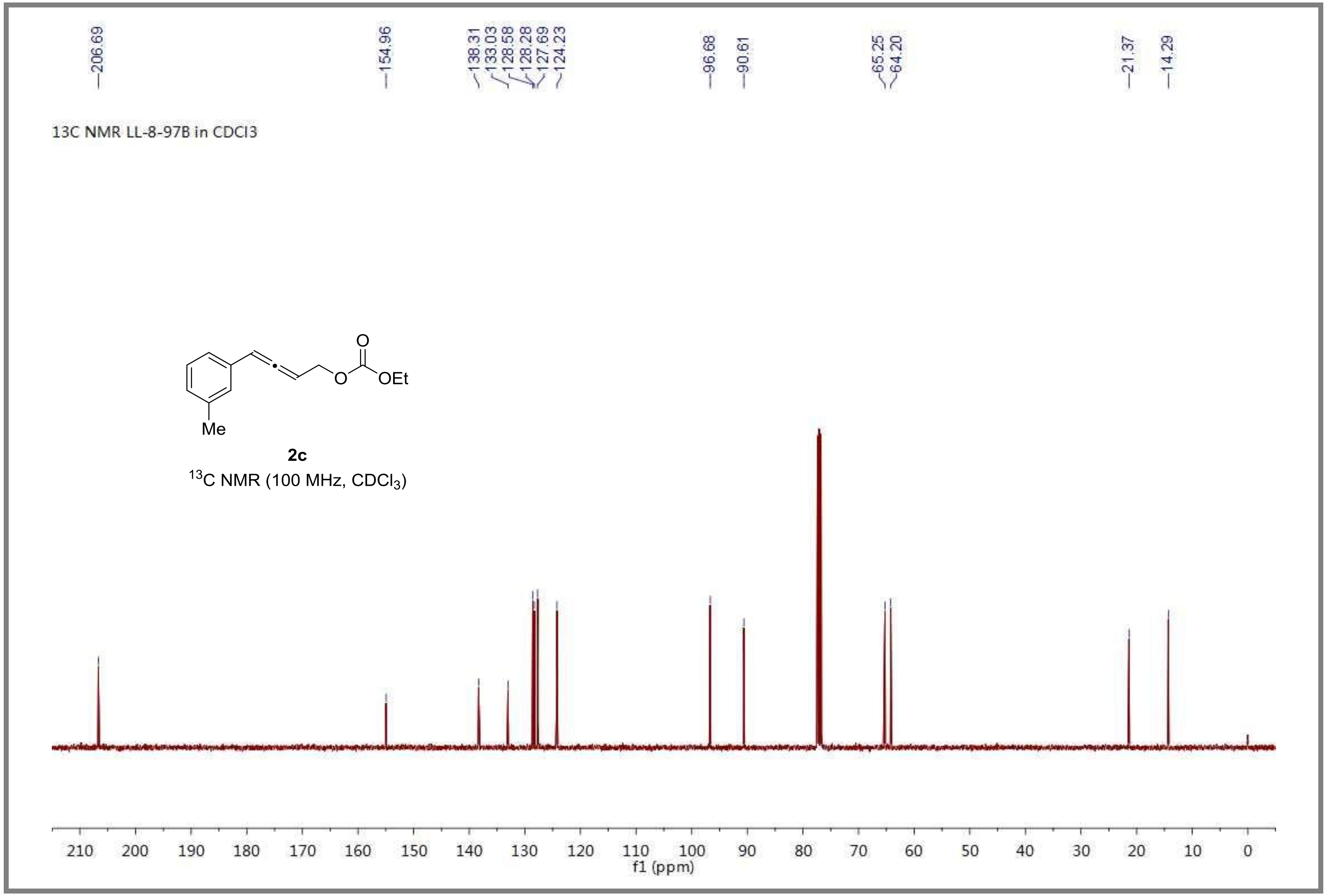
206.69
154.96
138.31
133.03
128.58
128.28
127.69
124.23
96.68
90.61
65.25
64.20
21.37
14.29
13C NMR LL-8-97B in CDCl3
O
OEt
O
Me
2c
13C NMR (100 MHz, CDCl3)
210 200 190 180 170 160 150 140 130 120 110 100 90 80 70 60 50 40 30 20 10 0
f1 (ppm)

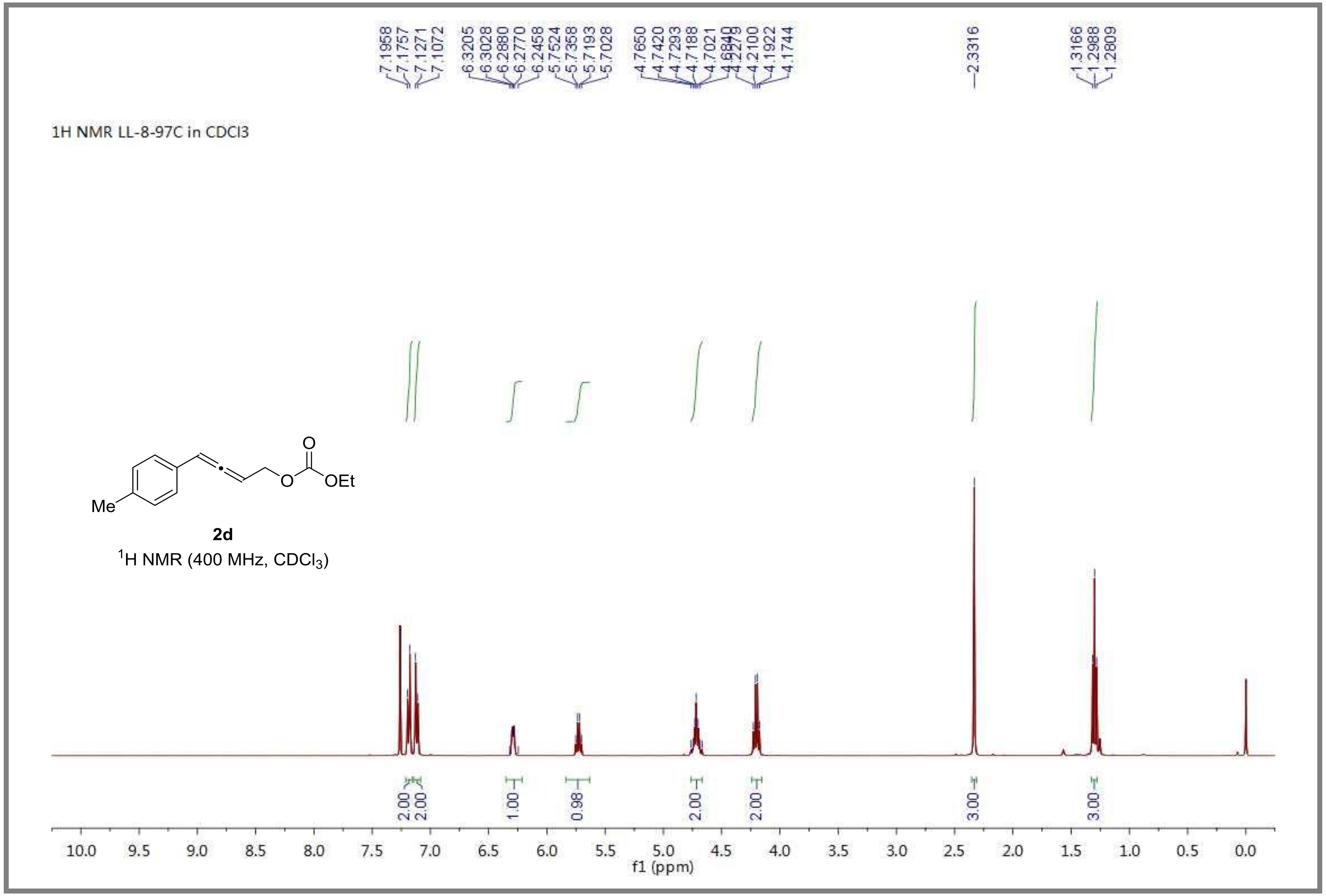

2d

^{1}H NMR (400 MHz, $CDCl_3$)

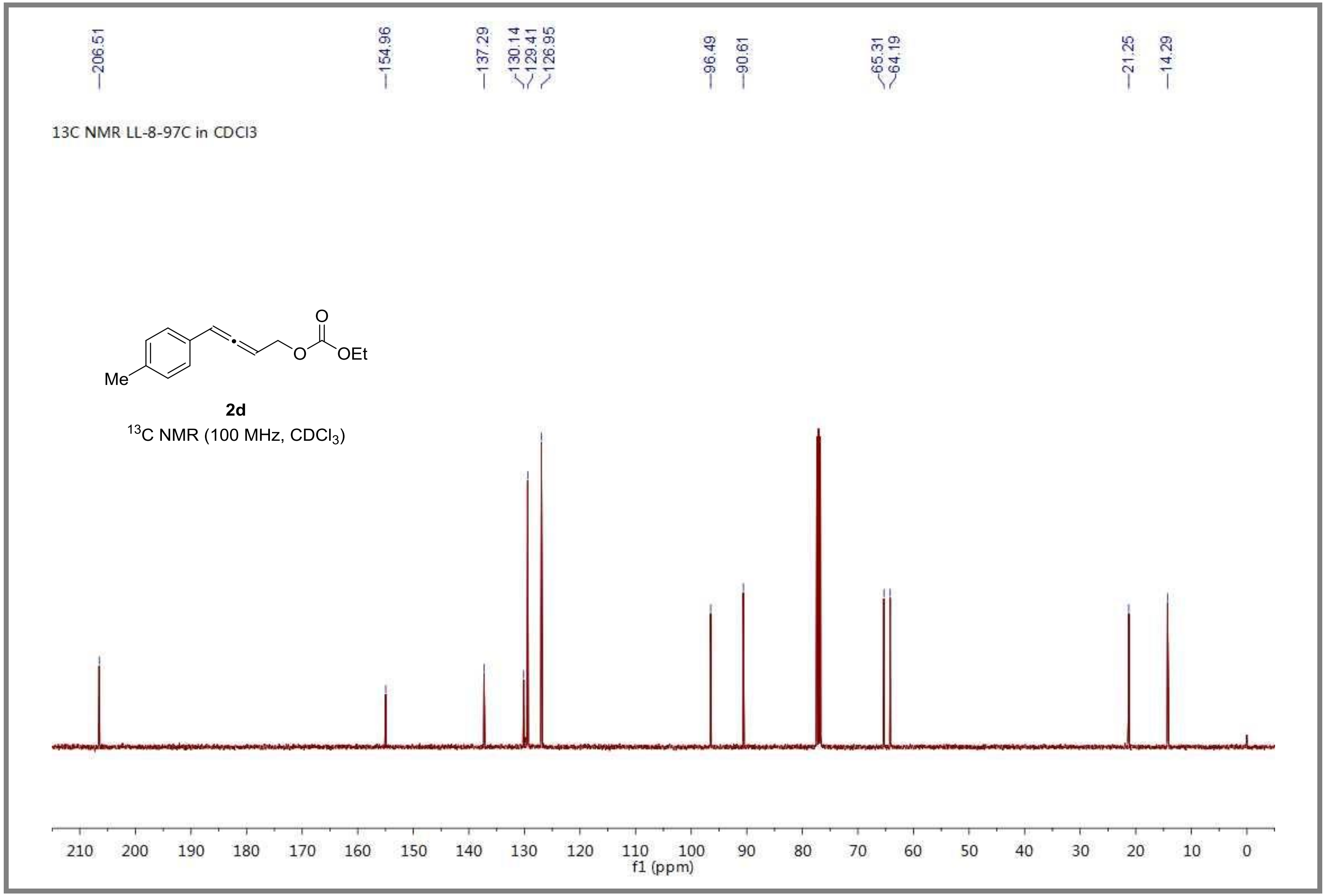
206.51
154.96
137.29
130.14
129.41
126.95
96.49
90.61
65.31
64.19
21.25
14.29
13C NMR LL-8-97C in CDCl3
O
OEt
Me
2d
13C NMR (100 MHz, CDCl3)
210 200 190 180 170 160 150 140 130 120 110 100 90 80 70 60 50 40 30 20 10 0
f1 (ppm)

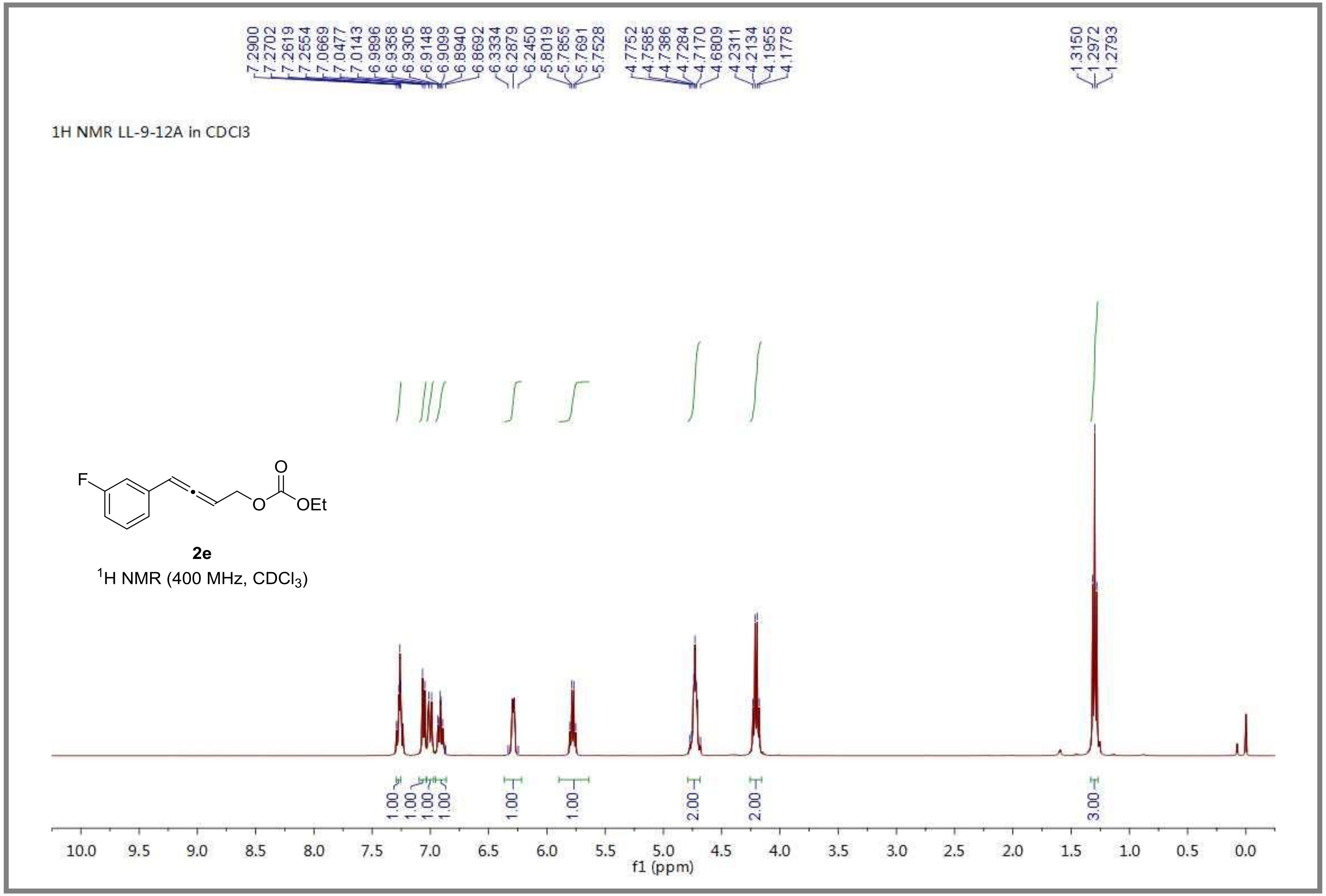
7.2900
7.2702
7.2619
7.2554
7.0669
7.0477
7.0143
6.9896
6.9358
6.9305
6.9148
6.9099
6.8940
6.8692
6.3334
6.2879
6.2450
5.8019
5.7855
5.7691
5.7528
4.7752
4.7585
4.7386
4.7284
4.7170
4.6809
4.2311
4.2134
4.1955
4.1778
1.3150
1.2972
1.2793
1H NMR LL-9-12A in CDCl3
F
O
O
OEt
2e
^{1}H NMR (400 MHz, $CDCl_3$)
1.00
1.00
1.00
1.00
1.00
1.00
2.00
2.00
3.00
10.0
9.5
9.0
8.5
8.0
7.5
7.0
6.5
6.0
5.5
5.0
4.5
4.0
3.5
3.0
2.5
2.0
1.5
1.0
0.5
0.0
f1 (ppm)

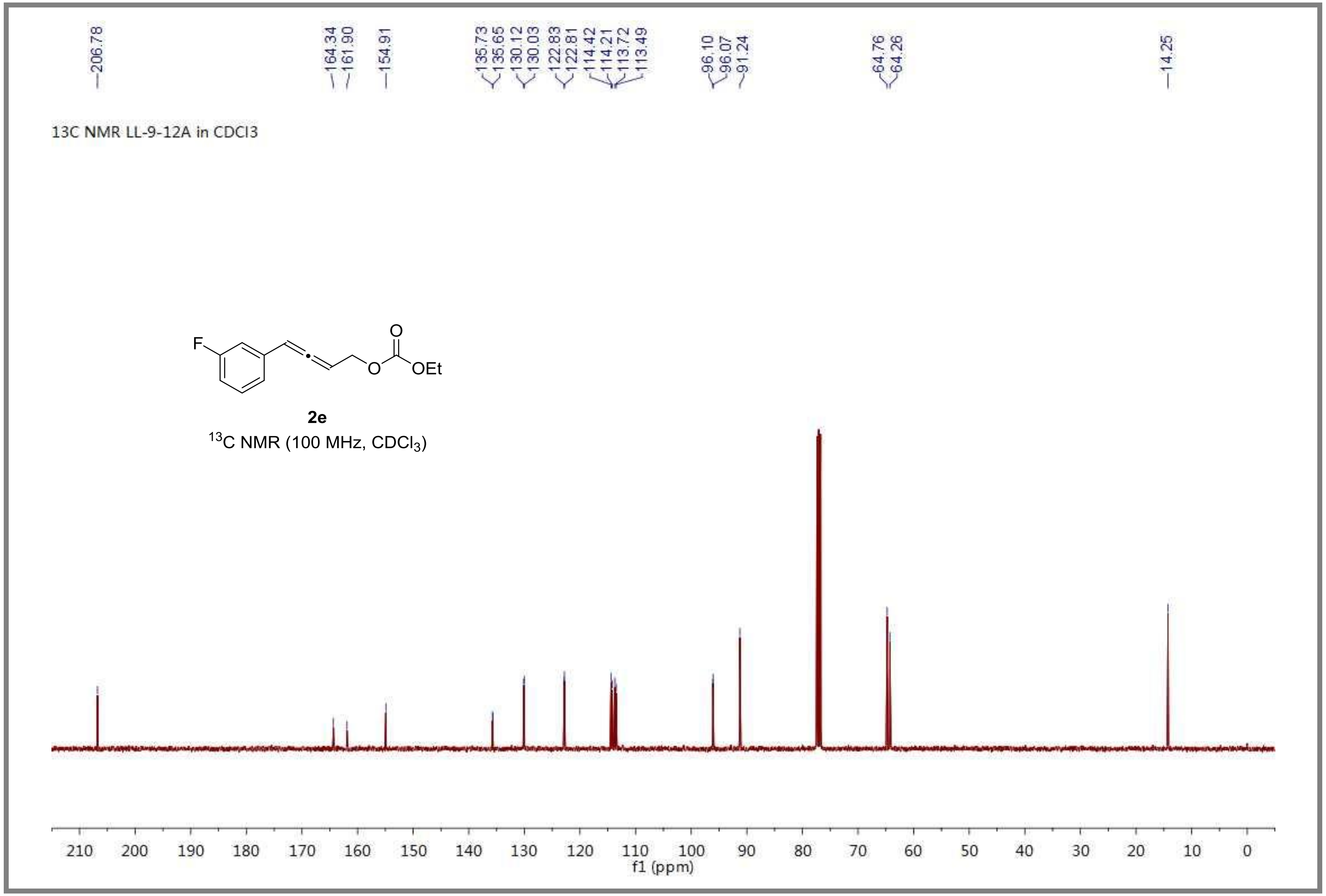
206.78
164.34
161.90
154.91
135.73
135.65
130.12
130.03
122.83
122.81
114.42
114.21
113.72
113.49
96.10
96.07
91.24
64.76
64.26
14.25
13C NMR LL-9-12A in CDCl3
F
O
O
OEt
2e
^{13}C NMR (100 MHz, $CDCl_3$)
210 200 190 180 170 160 150 140 130 120 110 100 90 80 70 60 50 40 30 20 10 0
f1 (ppm)

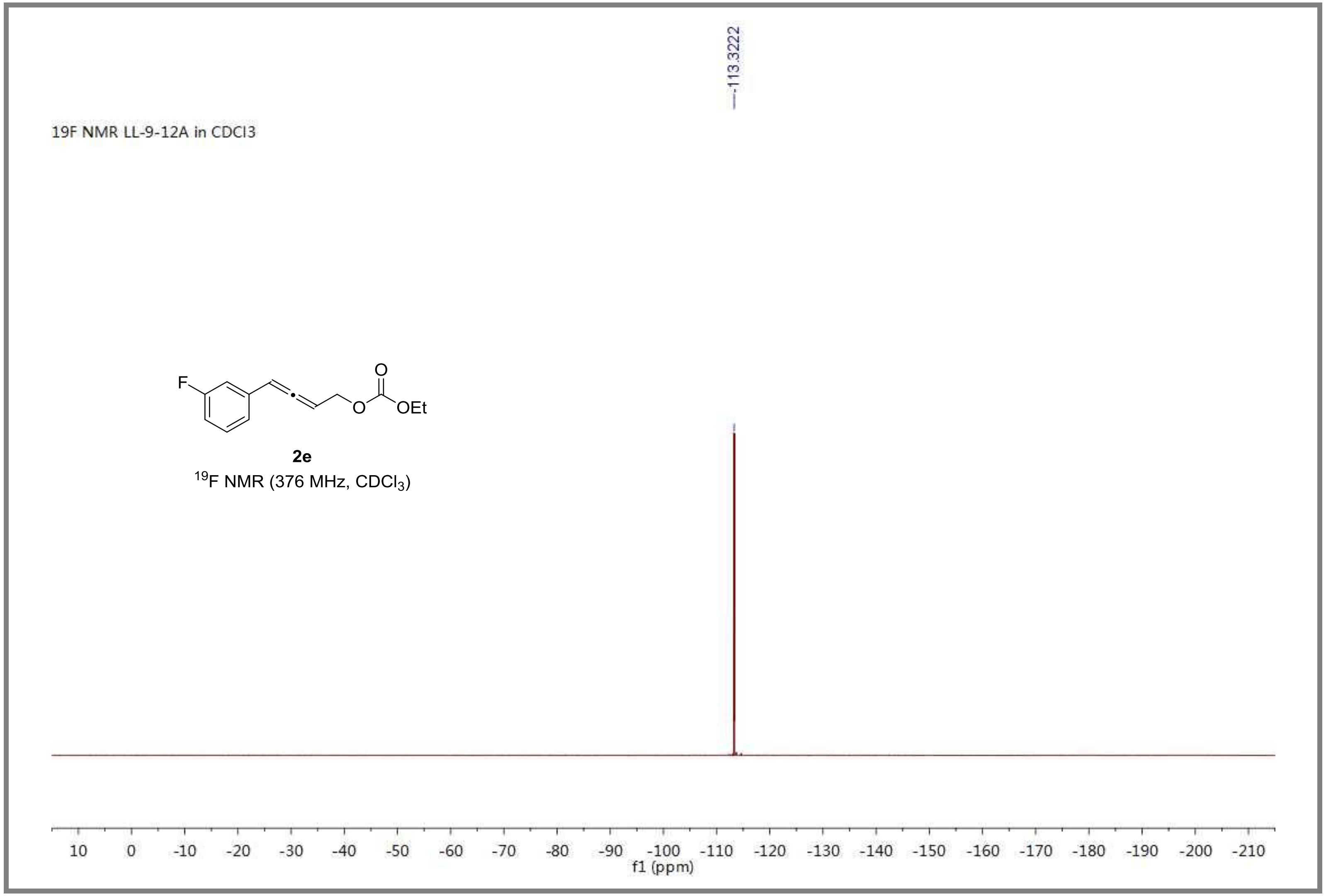
-113.3222
19F NMR LL-9-12A in CDCl3
F
O
O
OEt
2e
19F NMR (376 MHz, CDCl3)
10 0 -10 -20 -30 -40 -50 -60 -70 -80 -90 -100 -110 -120 -130 -140 -150 -160 -170 -180 -190 -200 -210
f1 (ppm)

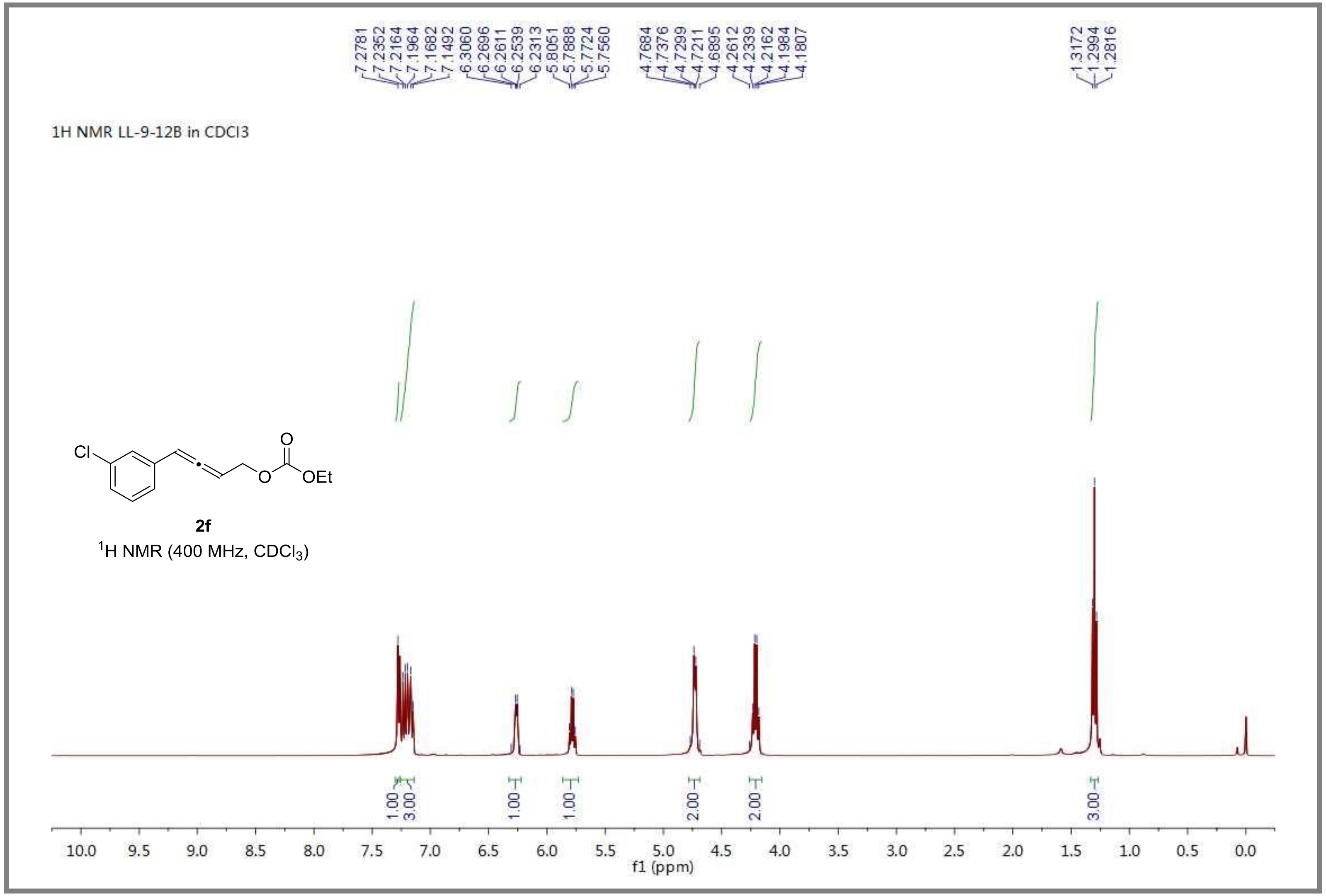

2f

^{1}H NMR (400 MHz, $CDCl_3$)

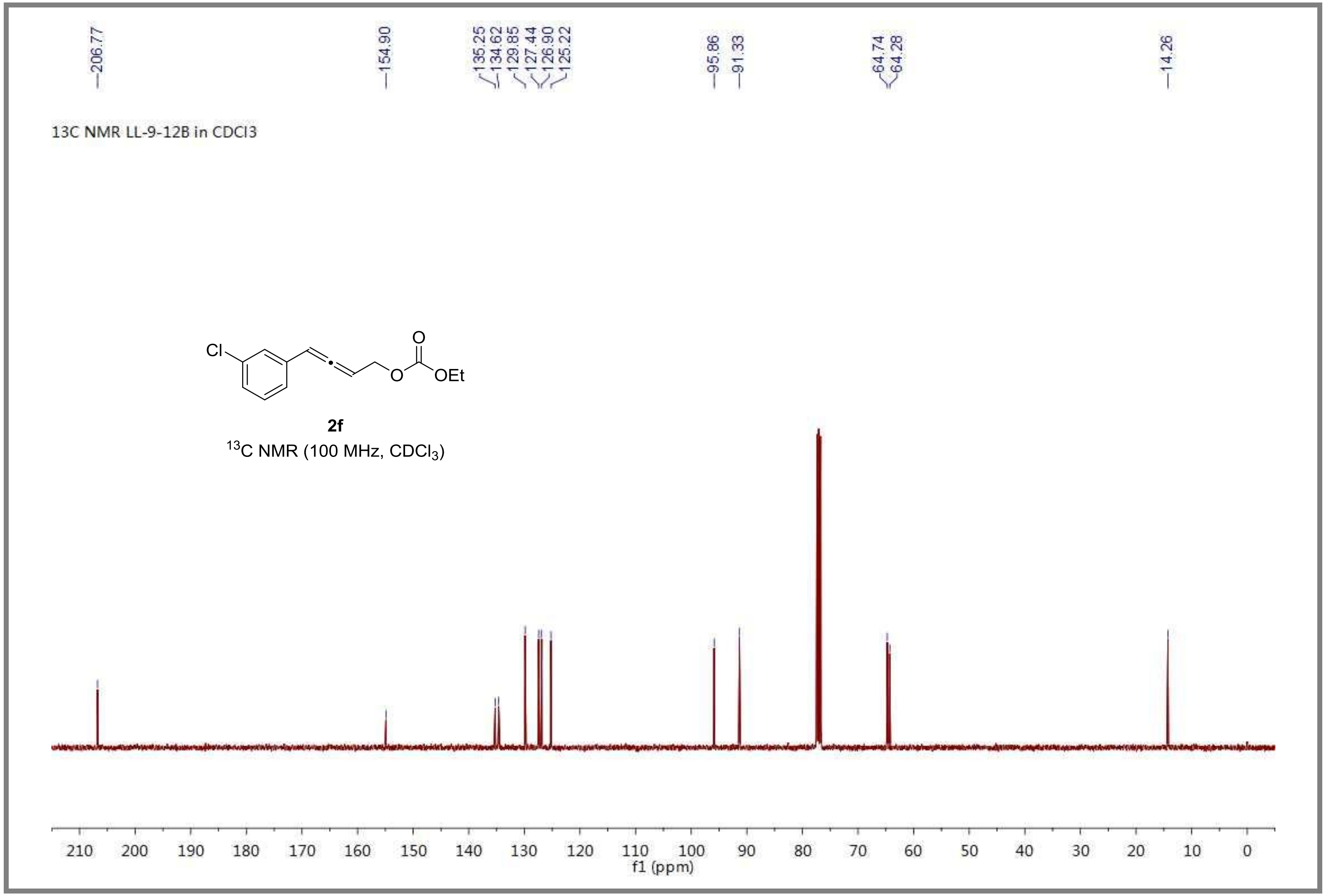
206.77
154.90
135.25
134.62
129.85
127.44
126.90
125.22
95.86
91.33
64.74
64.28
14.26
13C NMR LL-9-12B in CDCl3
Cl
O
O
OEt
2f
^{13}C NMR (100 MHz, $CDCl_3$)
210 200 190 180 170 160 150 140 130 120 110 100 90 80 70 60 50 40 30 20 10 0
f1 (ppm)

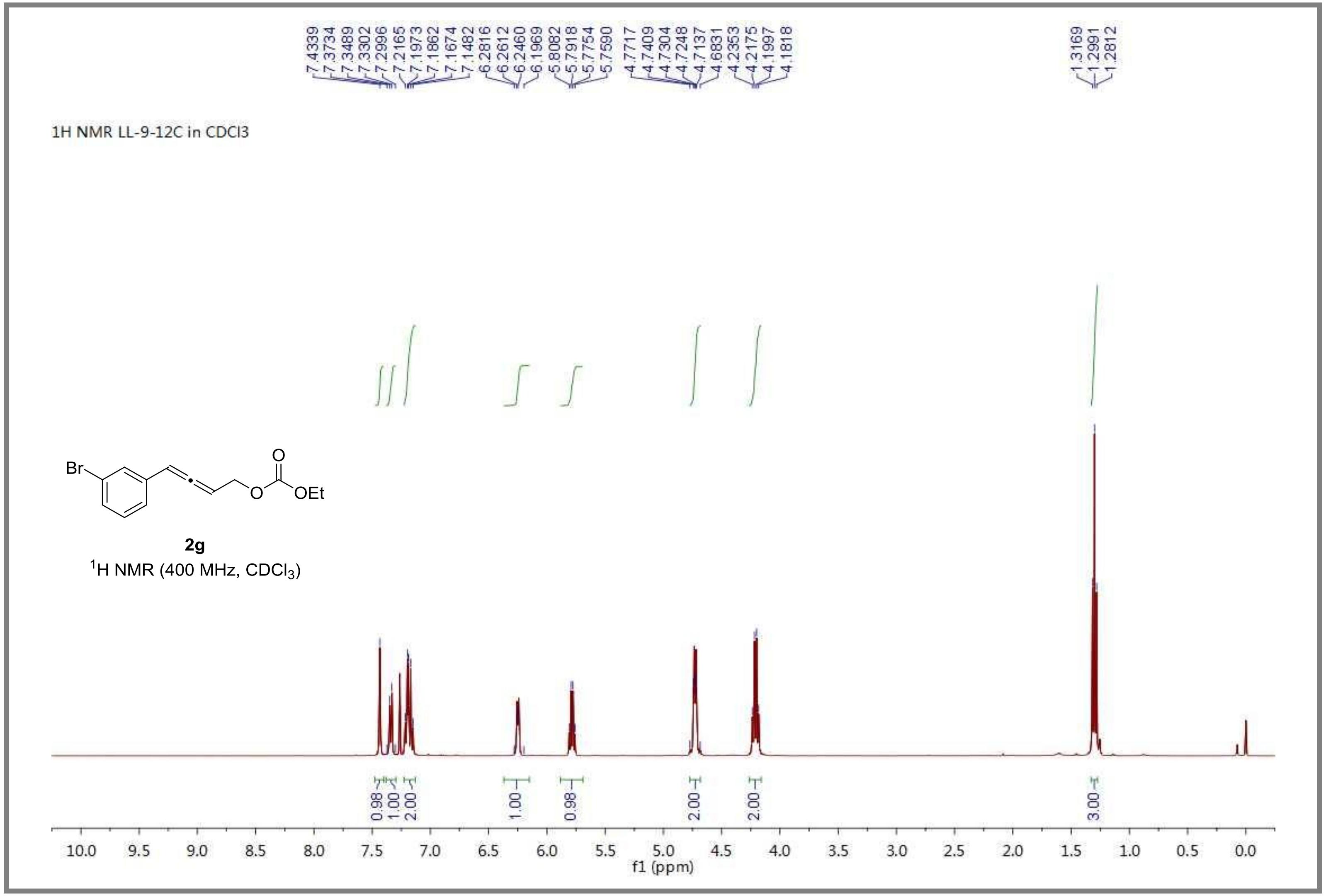

1H NMR LL-9-12C in CDCl3
7.4339
7.3734
7.3489
7.3302
7.2996
7.2165
7.1973
7.1862
7.1674
7.1482
6.2816
6.2612
6.2460
6.1969
5.8082
5.7918
5.7754
5.7590
4.7717
4.7409
4.7304
4.7248
4.7137
4.6831
4.2353
4.2175
4.1997
4.1818
1.3169
1.2991
1.2812
Br
O
O
OEt
2g
¹H NMR (400 MHz, CDCl₃)
0.98
1.00
2.00
1.00
0.98
2.00
2.00
3.00
10.0 9.5 9.0 8.5 8.0 7.5 7.0 6.5 6.0 5.5 5.0 4.5 4.0 3.5 3.0 2.5 2.0 1.5 1.0 0.5 0.0
f1 (ppm)

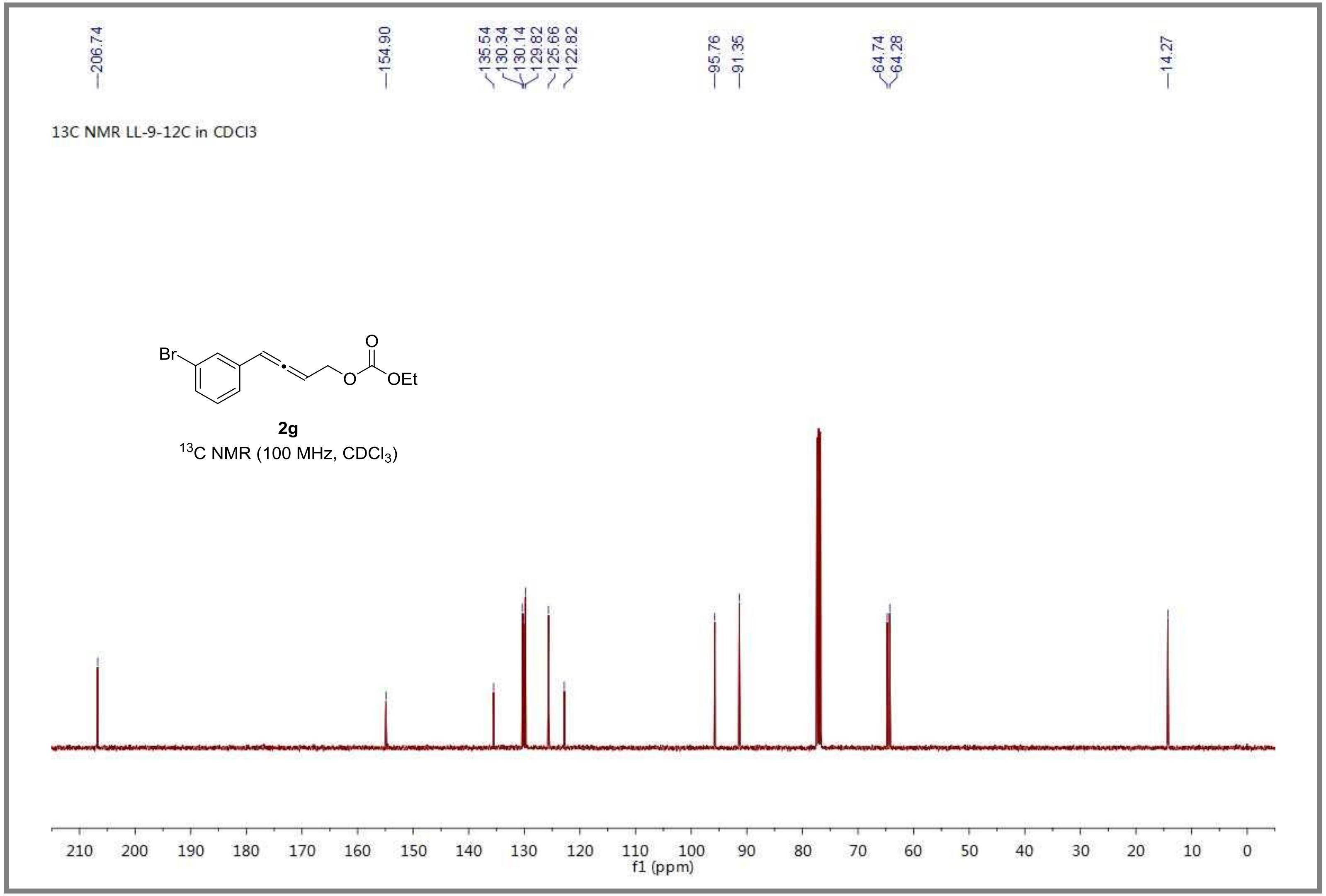

206.74
154.90
135.54
130.34
130.14
129.82
125.66
122.82
95.76
91.35
64.74
64.28
14.27
13C NMR LL-9-12C in CDCl3
Br
O
O
OEt
2g
^{13}C NMR (100 MHz, $CDCl_3$)
210 200 190 180 170 160 150 140 130 120 110 100 90 80 70 60 50 40 30 20 10 0
f1 (ppm)

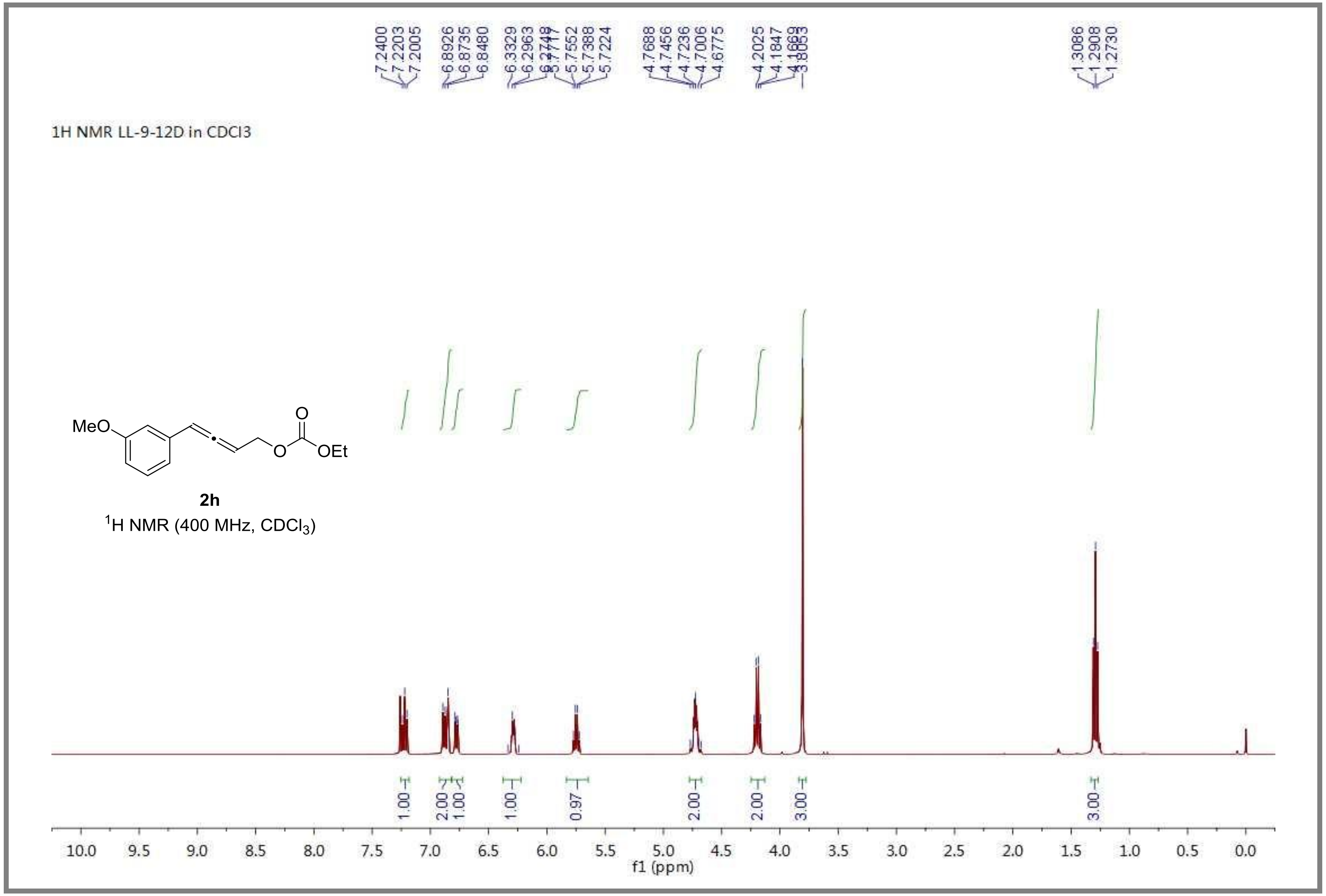

2h

^{1}H NMR (400 MHz, $CDCl_3$)

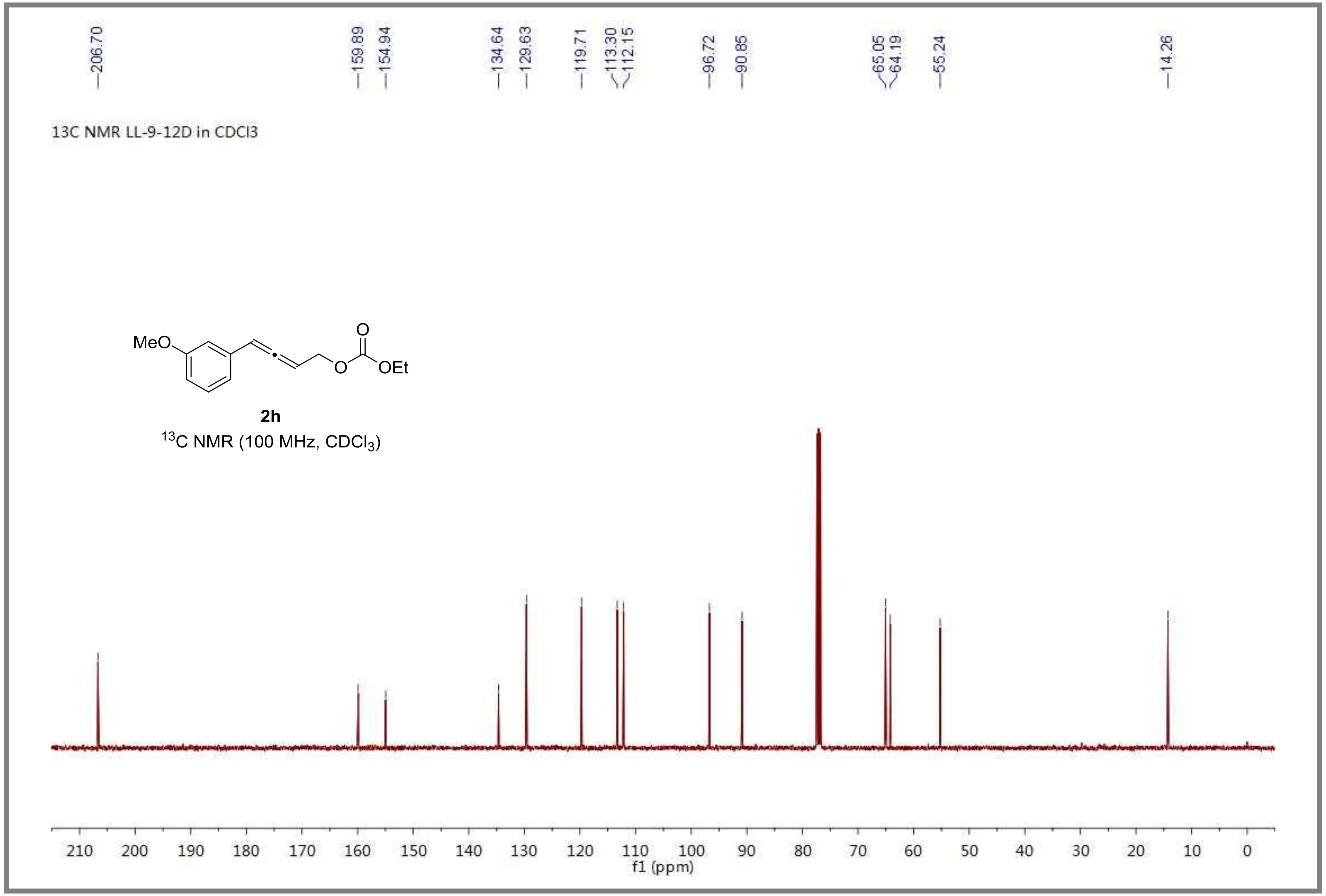

206.70
159.89
154.94
134.64
129.63
119.71
113.30
112.15
96.72
90.85
65.05
64.19
55.24
14.26
13C NMR LL-9-12D in CDCl3
MeO
O
O
OEt
2h
13C NMR (100 MHz, CDCl3)
210
200
190
180
170
160
150
140
130
120
110
100
90
80
70
60
50
40
30
20
10
0
f1 (ppm)

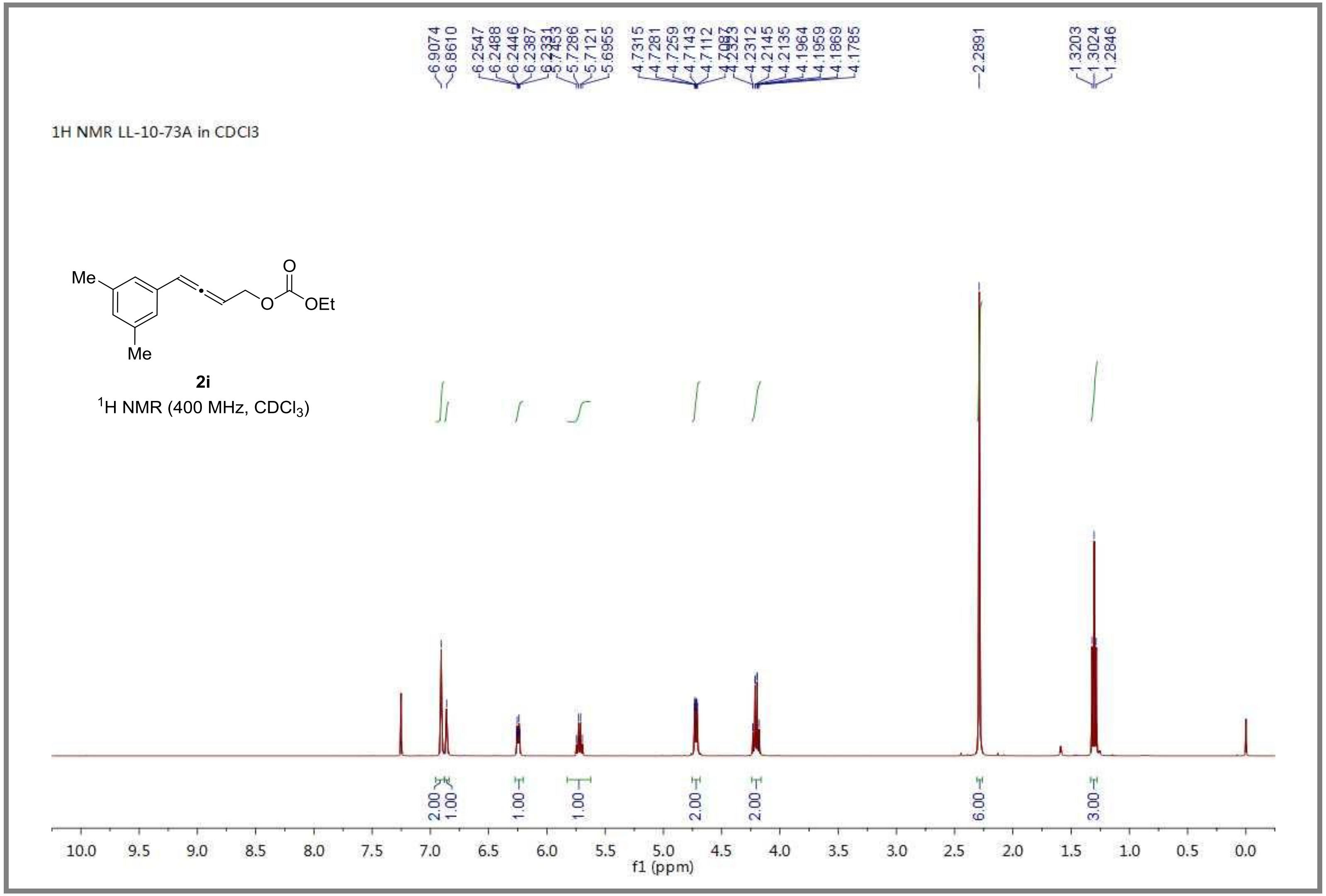
1H NMR LL-10-73A in CDCl3
Me
Me
O
O
OEt
2i
^{1}H NMR (400 MHz, $CDCl_3$)
6.9074
6.8610
6.2547
6.2488
6.2446
6.2387
6.2331
5.7453
5.7286
5.7121
5.6955
4.7315
4.7281
4.7259
4.7143
4.7112
4.7087
4.2323
4.2312
4.2145
4.2135
4.1964
4.1959
4.1869
4.1785
2.2891
1.3203
1.3024
1.2846
2.00
1.00
1.00
1.00
2.00
2.00
6.00
3.00
10.0 9.5 9.0 8.5 8.0 7.5 7.0 6.5 6.0 5.5 5.0 4.5 4.0 3.5 3.0 2.5 2.0 1.5 1.0 0.5 0.0
f1 (ppm)

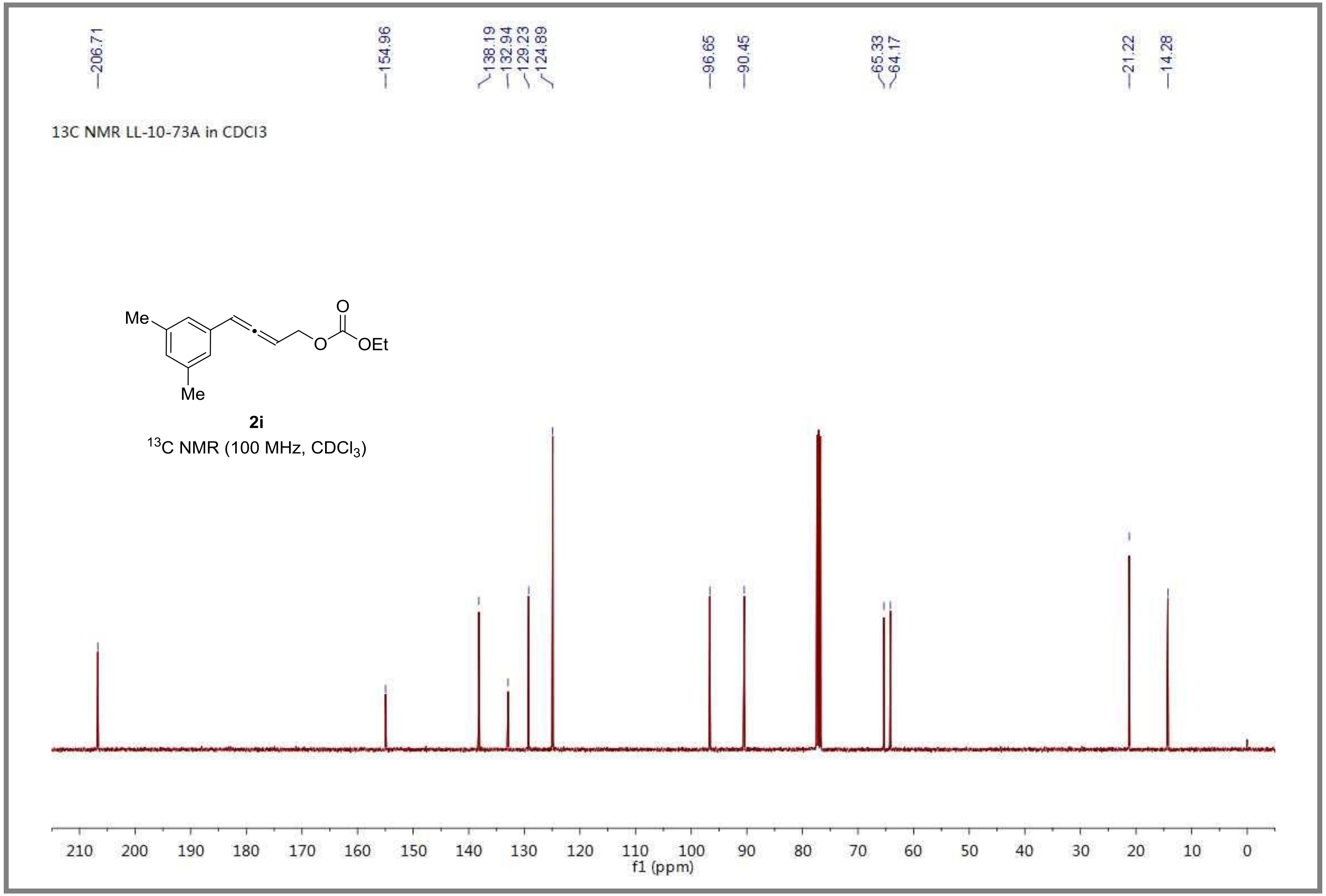

2i

^{13}C NMR (100 MHz, $CDCl_3$)

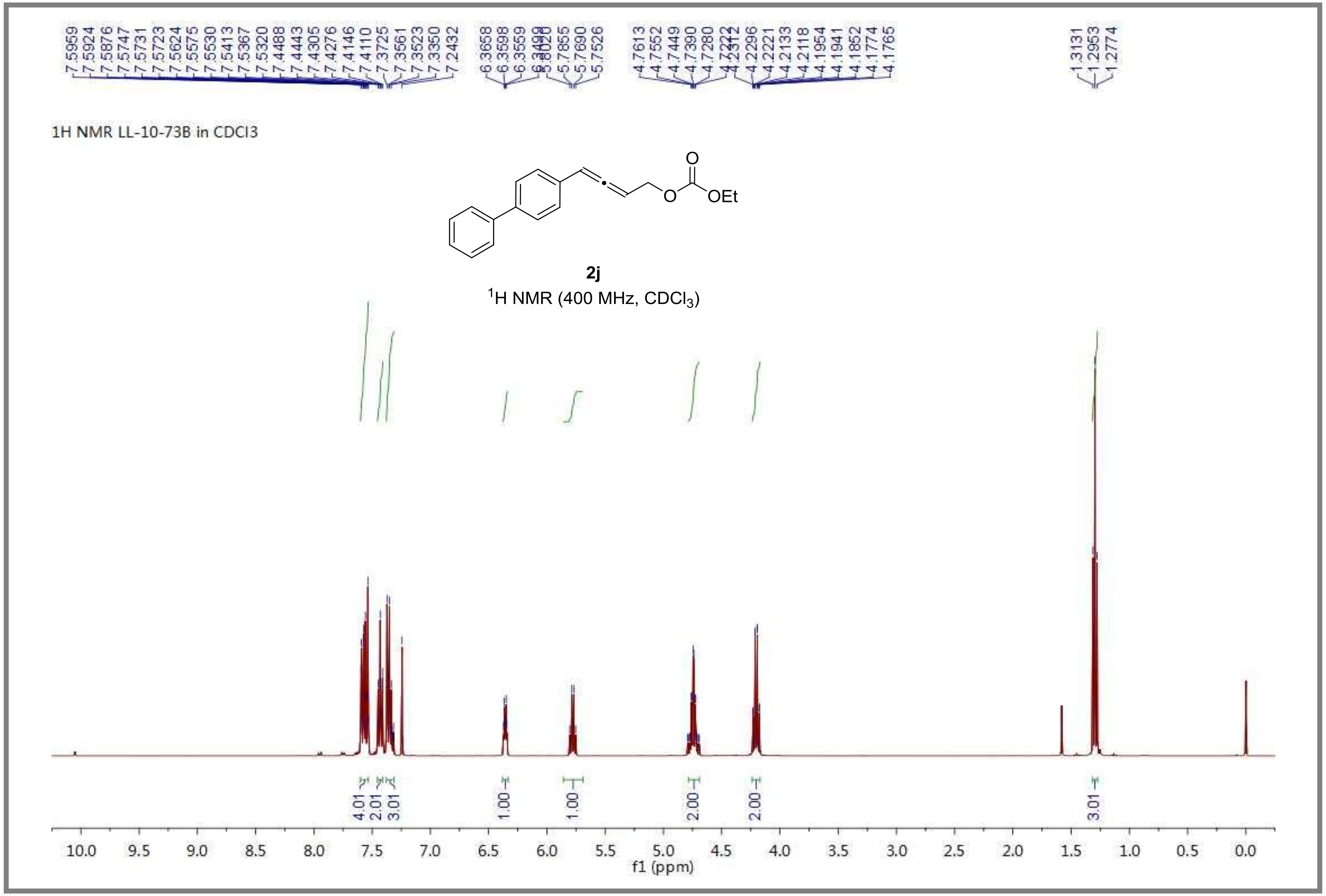

1H NMR LL-10-73B in CDCl3
OEt
O
2j
1H NMR (400 MHz, CDCl3)
f1 (ppm)

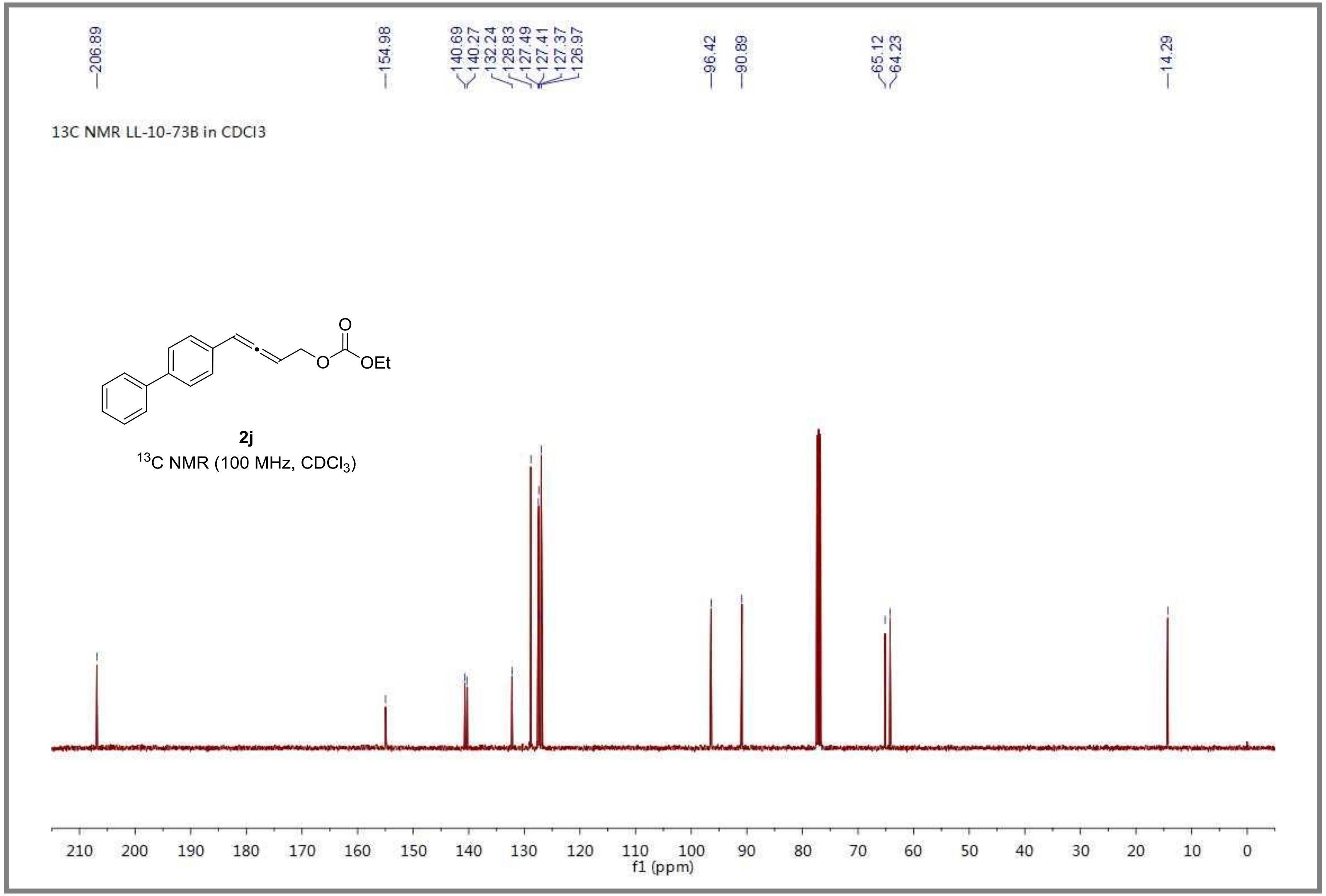
206.89
154.98
140.69
140.27
132.24
128.83
127.49
127.41
127.37
126.97
96.42
90.89
65.12
64.23
14.29
13C NMR LL-10-73B in CDCl3
O
O
OEt
2j
^{13}C NMR (100 MHz, CDCl$_3$)
210
200
190
180
170
160
150
140
130
120
110
100
90
80
70
60
50
40
30
20
10
0
f1 (ppm)

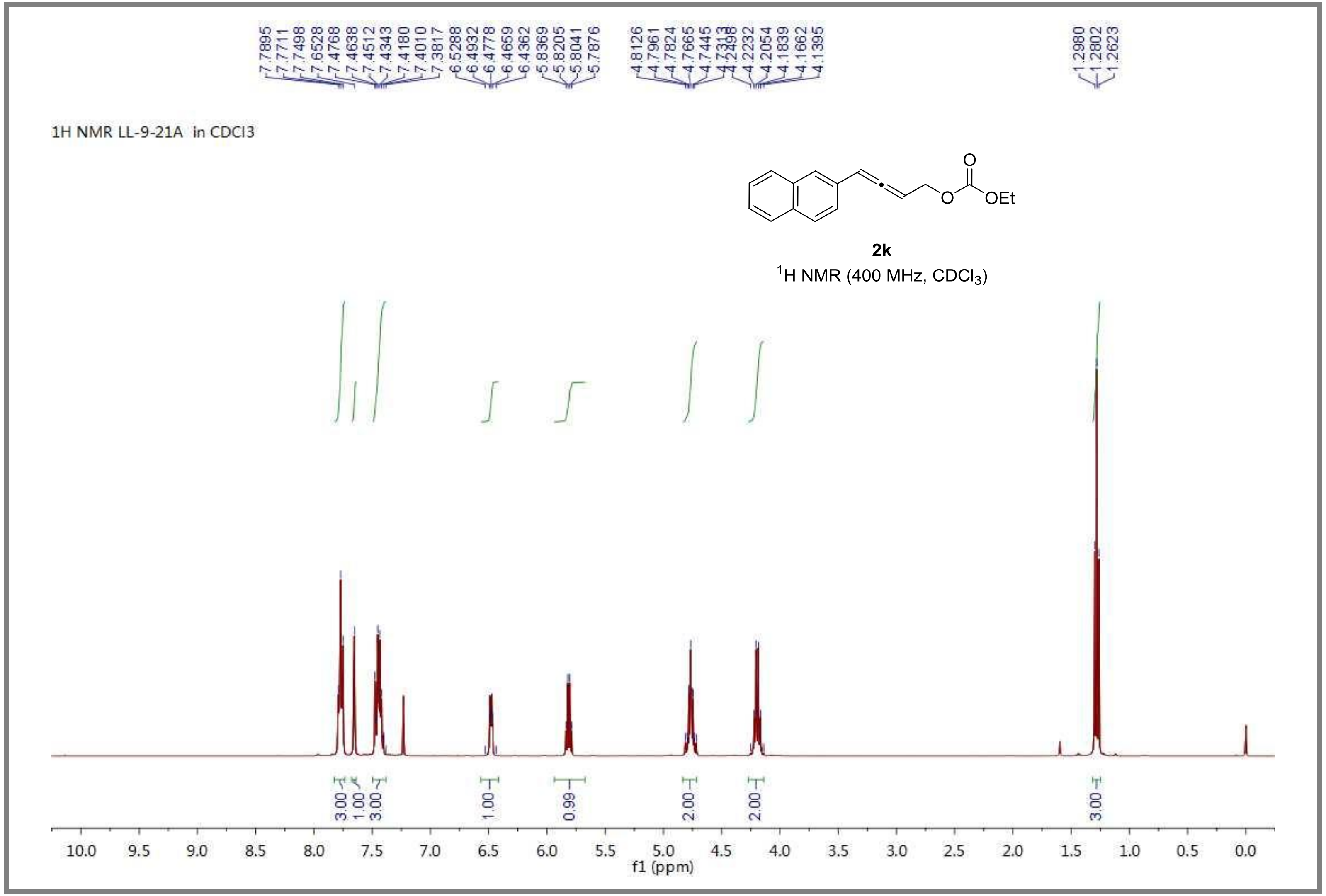
1H NMR LL-9-21A in CDCl3
2k
^{1}H NMR (400 MHz, CDCl$_3$)
OEt
O
f1 (ppm)

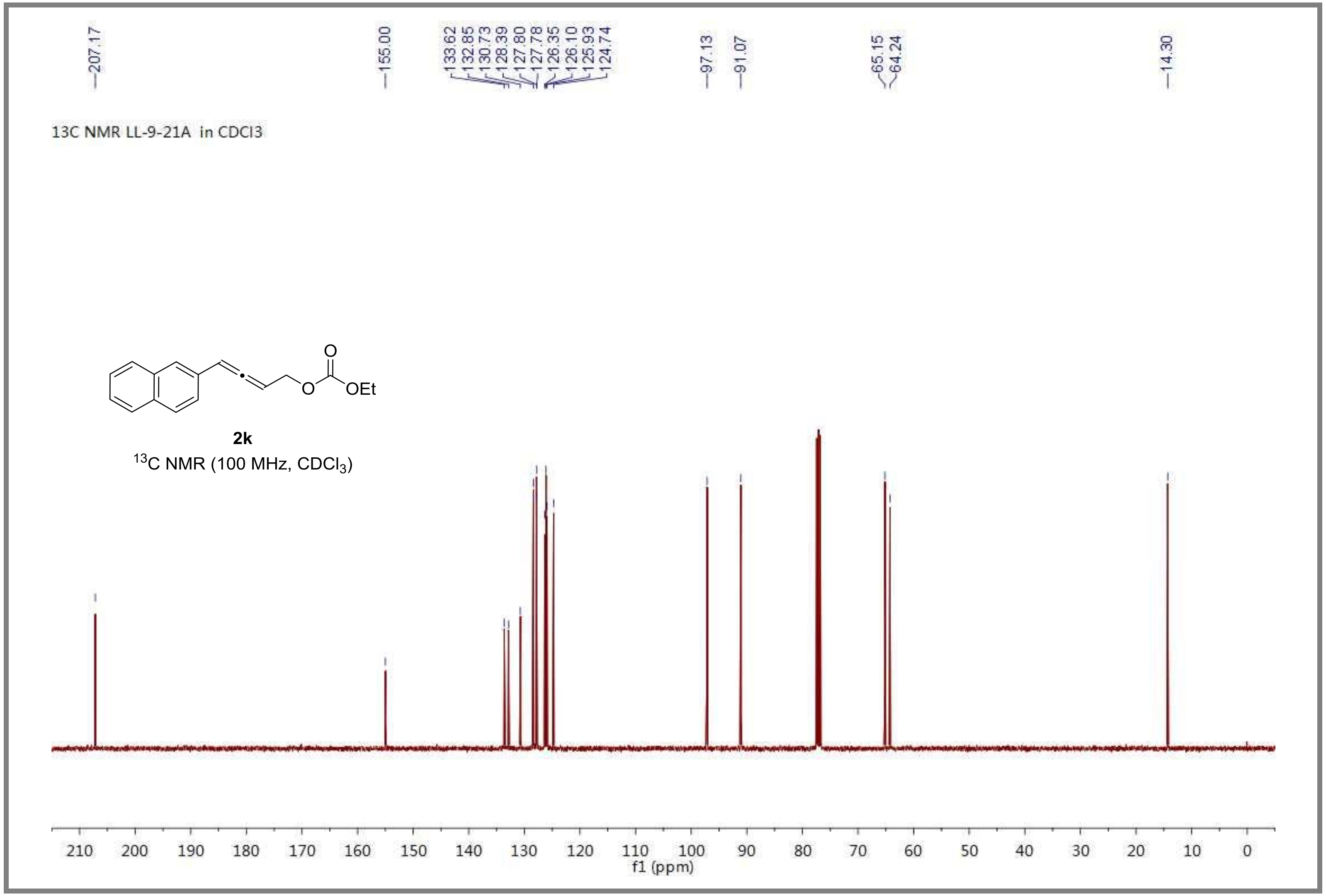
207.17
155.00
133.62
132.85
130.73
128.39
127.80
127.78
126.35
126.10
125.93
124.74
97.13
91.07
65.15
64.24
14.30
13C NMR LL-9-21A in CDCl3
OEt
O
O
2k
^{13}C NMR (100 MHz, $CDCl_3$)
210 200 190 180 170 160 150 140 130 120 110 100 90 80 70 60 50 40 30 20 10 0
f1 (ppm)

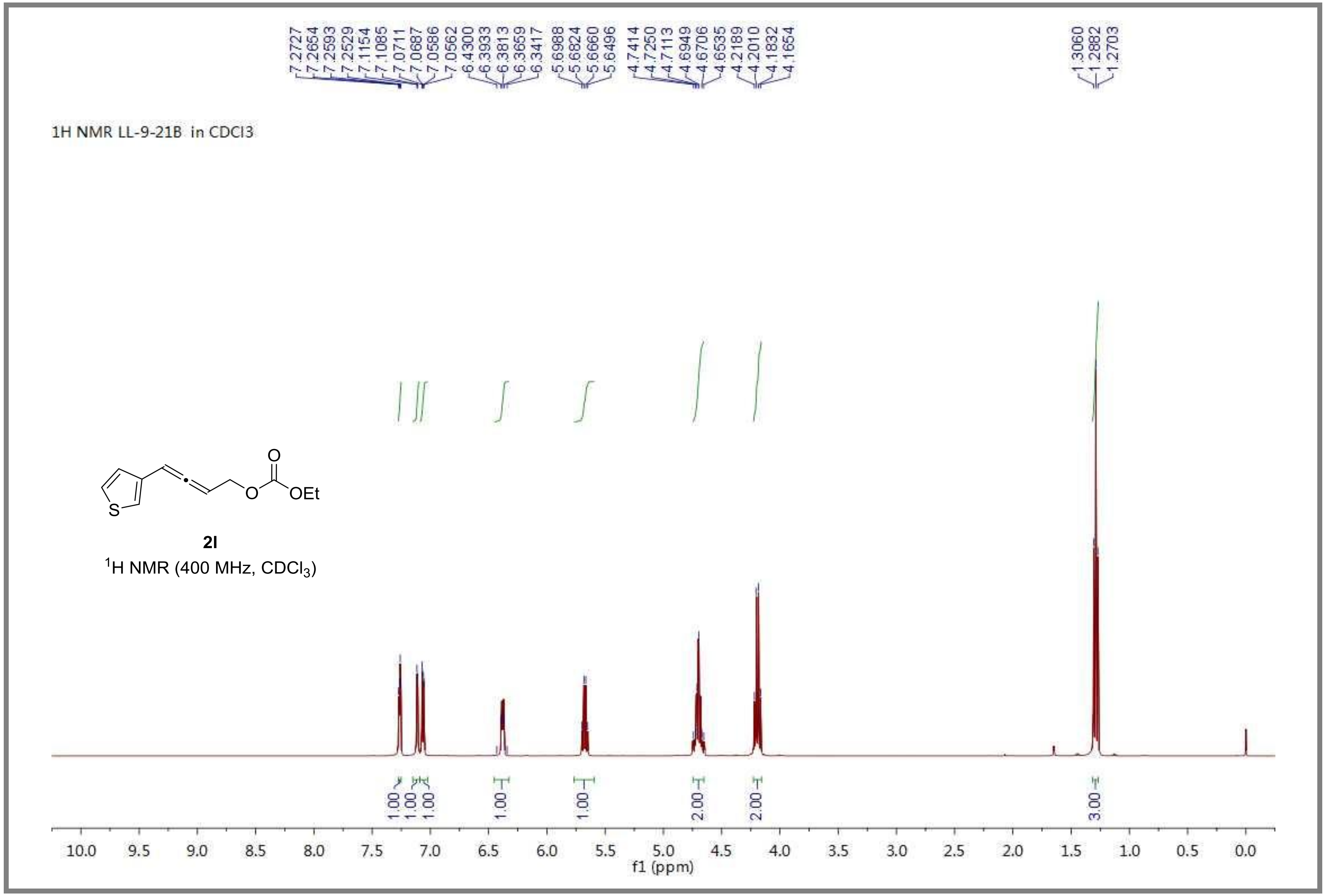
7.2727
7.2654
7.2593
7.2529
7.1154
7.1085
7.0711
7.0687
7.0586
7.0562
6.4300
6.3933
6.3813
6.3659
6.3417
5.6988
5.6824
5.6660
5.6496
4.7414
4.7250
4.7113
4.6949
4.6706
4.6535
4.2189
4.2010
4.1832
4.1654
1.3060
1.2882
1.2703
1H NMR LL-9-21B in CDCl3
O
S
O
OEt
2l
^{1}H NMR (400 MHz, CDCl$_3$)
1.00
1.00
1.00
1.00
1.00
2.00
2.00
3.00
10.0 9.5 9.0 8.5 8.0 7.5 7.0 6.5 6.0 5.5 5.0 4.5 4.0 3.5 3.0 2.5 2.0 1.5 1.0 0.5 0.0
f1 (ppm)

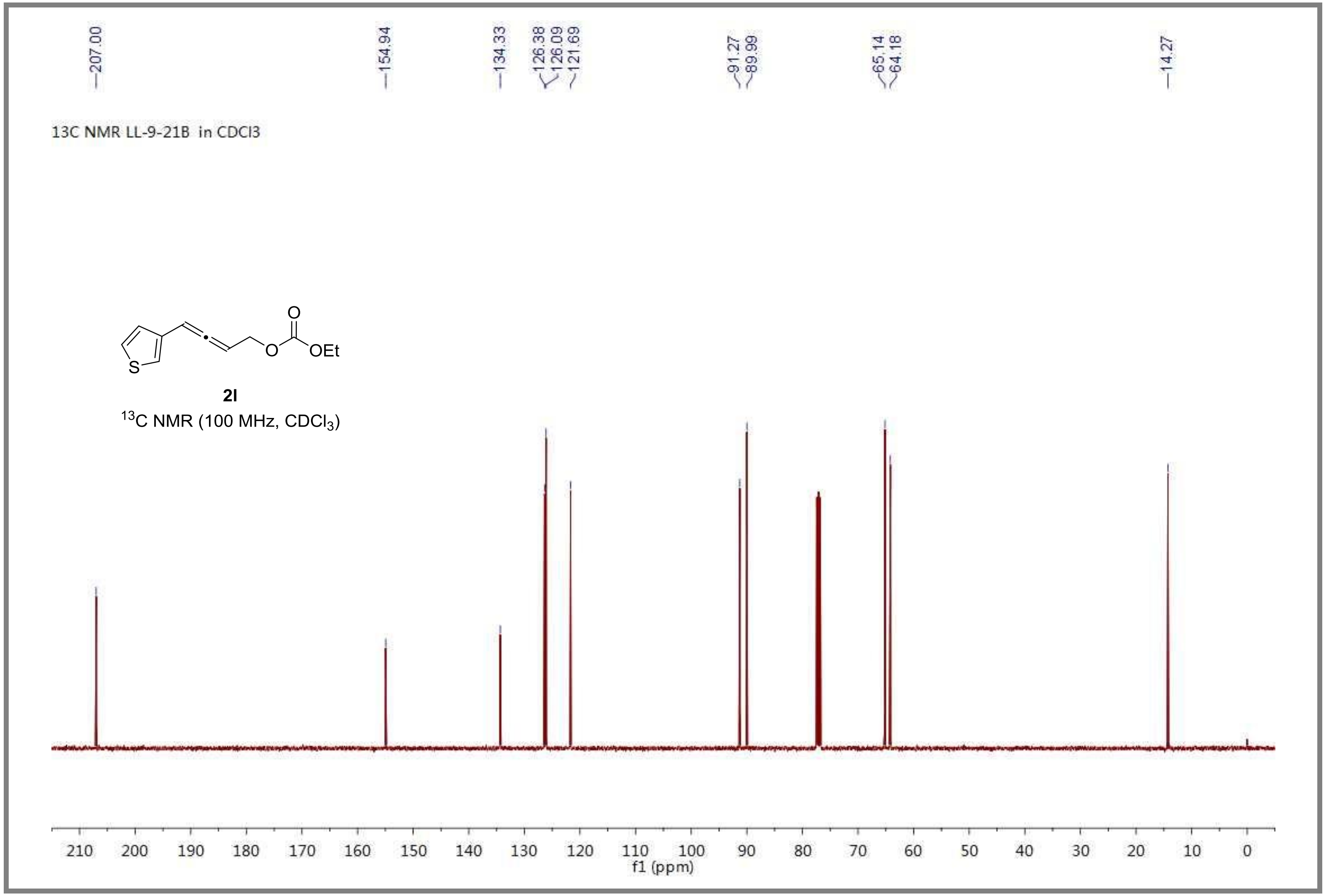

207.00
154.94
134.33
126.38
126.09
121.69
91.27
89.99
65.14
64.18
14.27
13C NMR LL-9-21B in CDCl3
O
OEt
O
S
2l
^{13}C NMR (100 MHz, $CDCl_3$)
210 200 190 180 170 160 150 140 130 120 110 100 90 80 70 60 50 40 30 20 10 0
f1 (ppm)

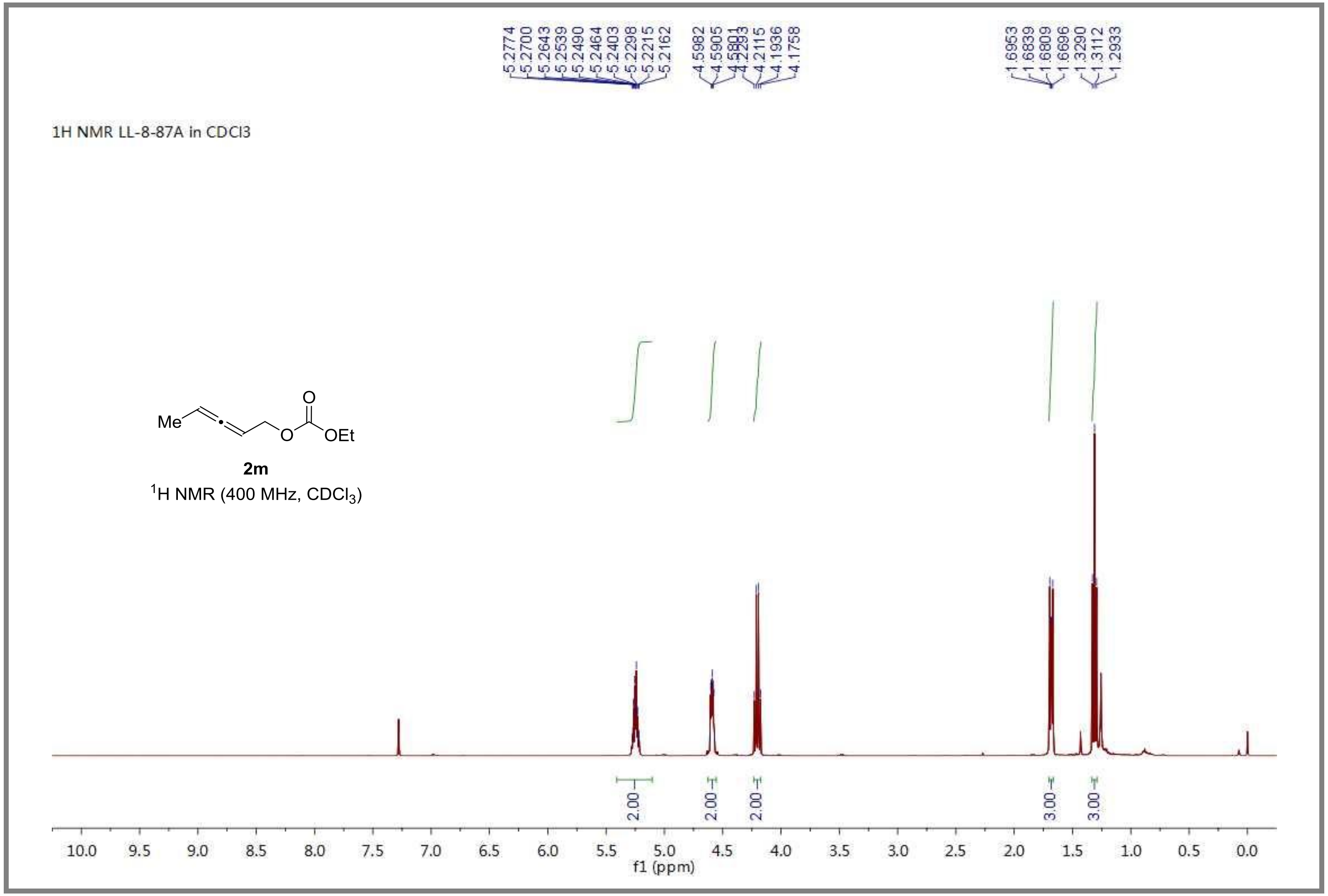

1H NMR LL-8-87A in CDCl3
5.2774
5.2700
5.2643
5.2539
5.2490
5.2464
5.2403
5.2298
5.2215
5.2162
4.5982
4.5905
4.2115
4.1936
4.1758
1.6953
1.6839
1.6809
1.6696
1.3290
1.3112
1.2933
Me
O
OEt
2m
^{1}H NMR (400 MHz, $CDCl_3$)
2.00
2.00
2.00
3.00
3.00
10.0 9.5 9.0 8.5 8.0 7.5 7.0 6.5 6.0 5.5 5.0 4.5 4.0 3.5 3.0 2.5 2.0 1.5 1.0 0.5 0.0
f1 (ppm)

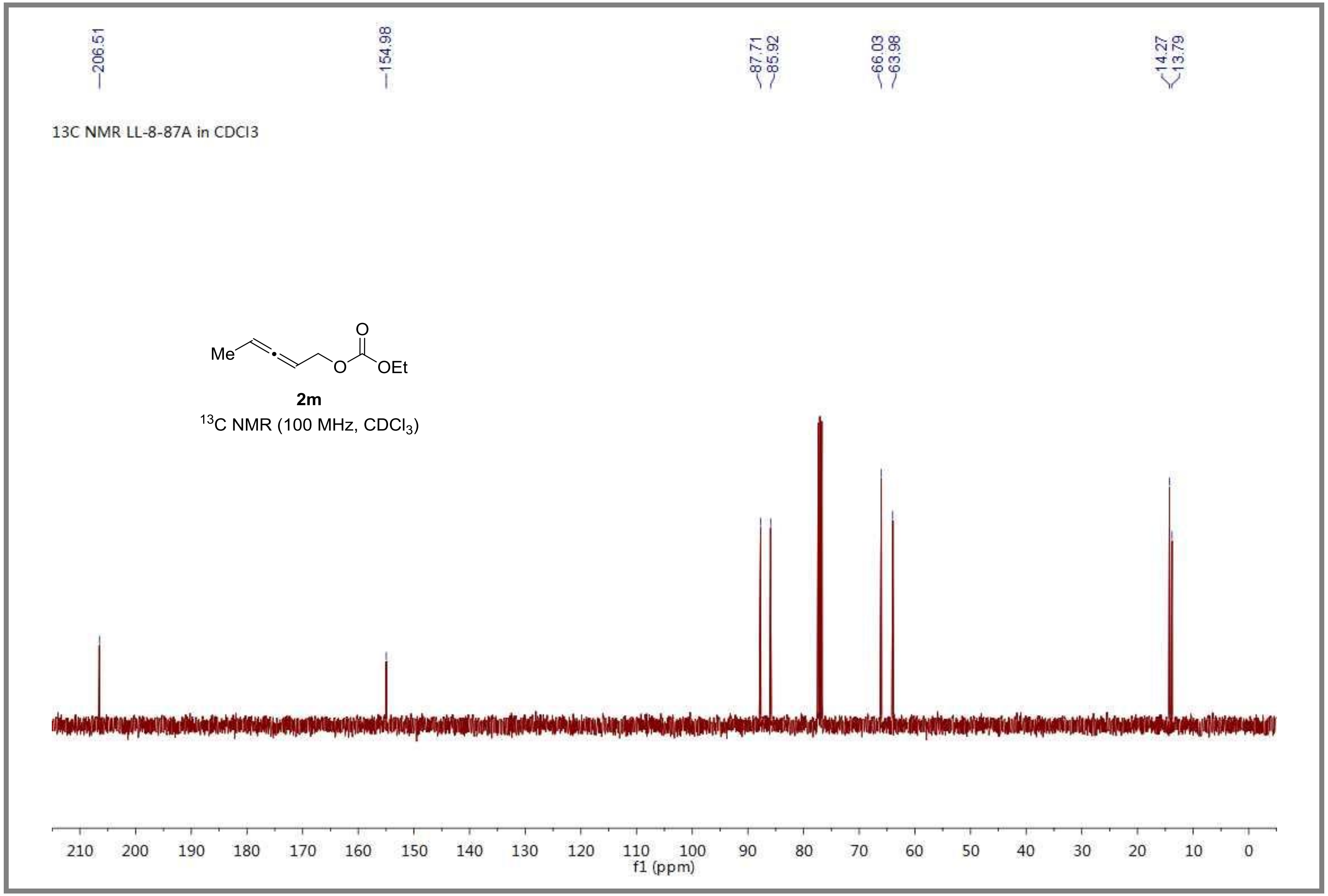

206.51
154.98
87.71
85.92
66.03
63.98
14.27
13.79
13C NMR LL-8-87A in CDCl3
Me
O
O
OEt
2m
^{13}C NMR (100 MHz, $CDCl_3$)
210 200 190 180 170 160 150 140 130 120 110 100 90 80 70 60 50 40 30 20 10 0
f1 (ppm)

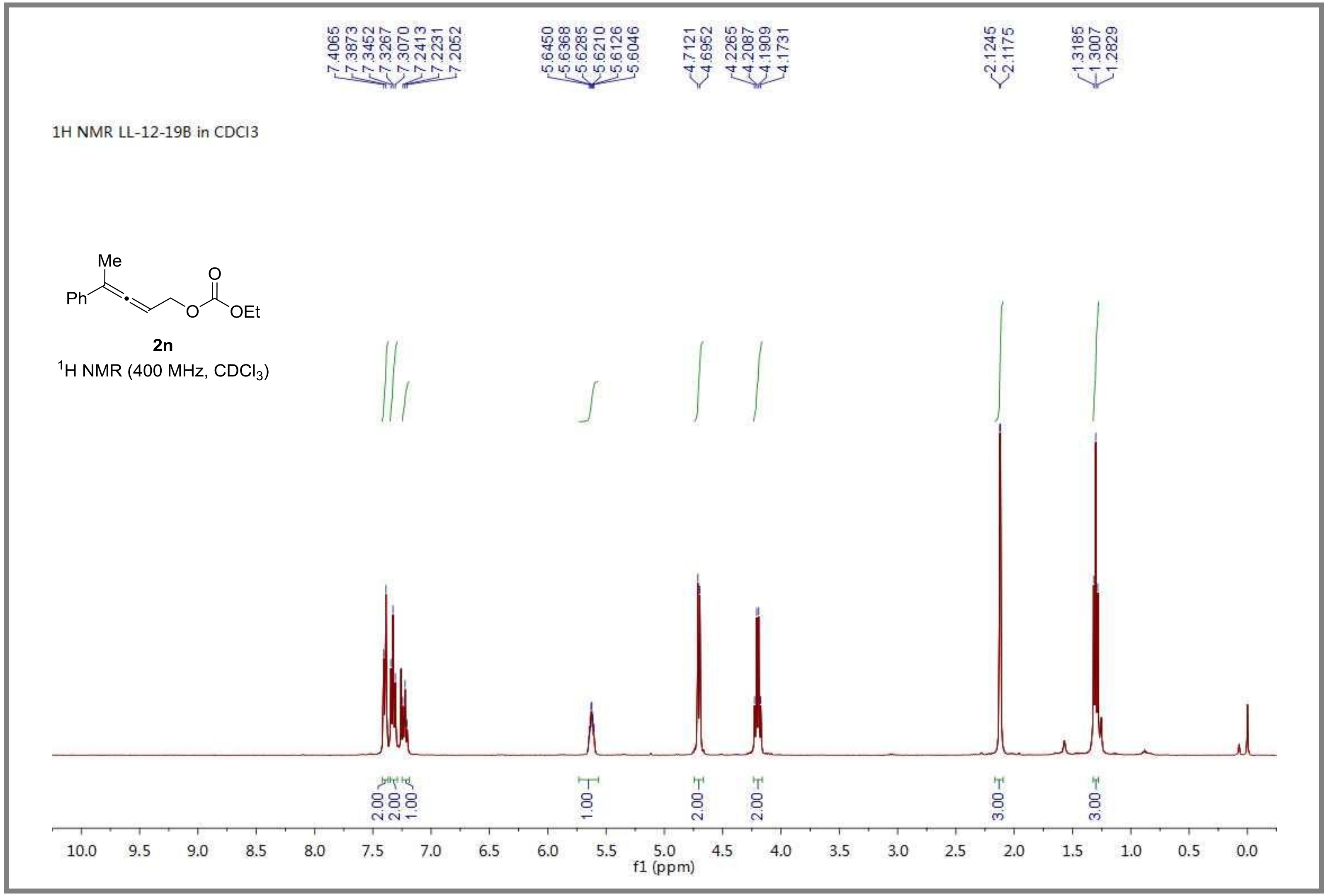

1H NMR LL-12-19B in CDCl3
Me
Ph
O
O
OEt
2n
^{1}H NMR (400 MHz, CDCl$_3$)
7.4065
7.3873
7.3452
7.3267
7.3070
7.2413
7.2231
7.2052
5.6450
5.6368
5.6285
5.6210
5.6126
5.6046
4.7121
4.6952
4.2265
4.2087
4.1909
4.1731
2.1245
2.1175
1.3185
1.3007
1.2829
2.00
2.00
1.00
1.00
2.00
2.00
3.00
3.00
f1 (ppm)

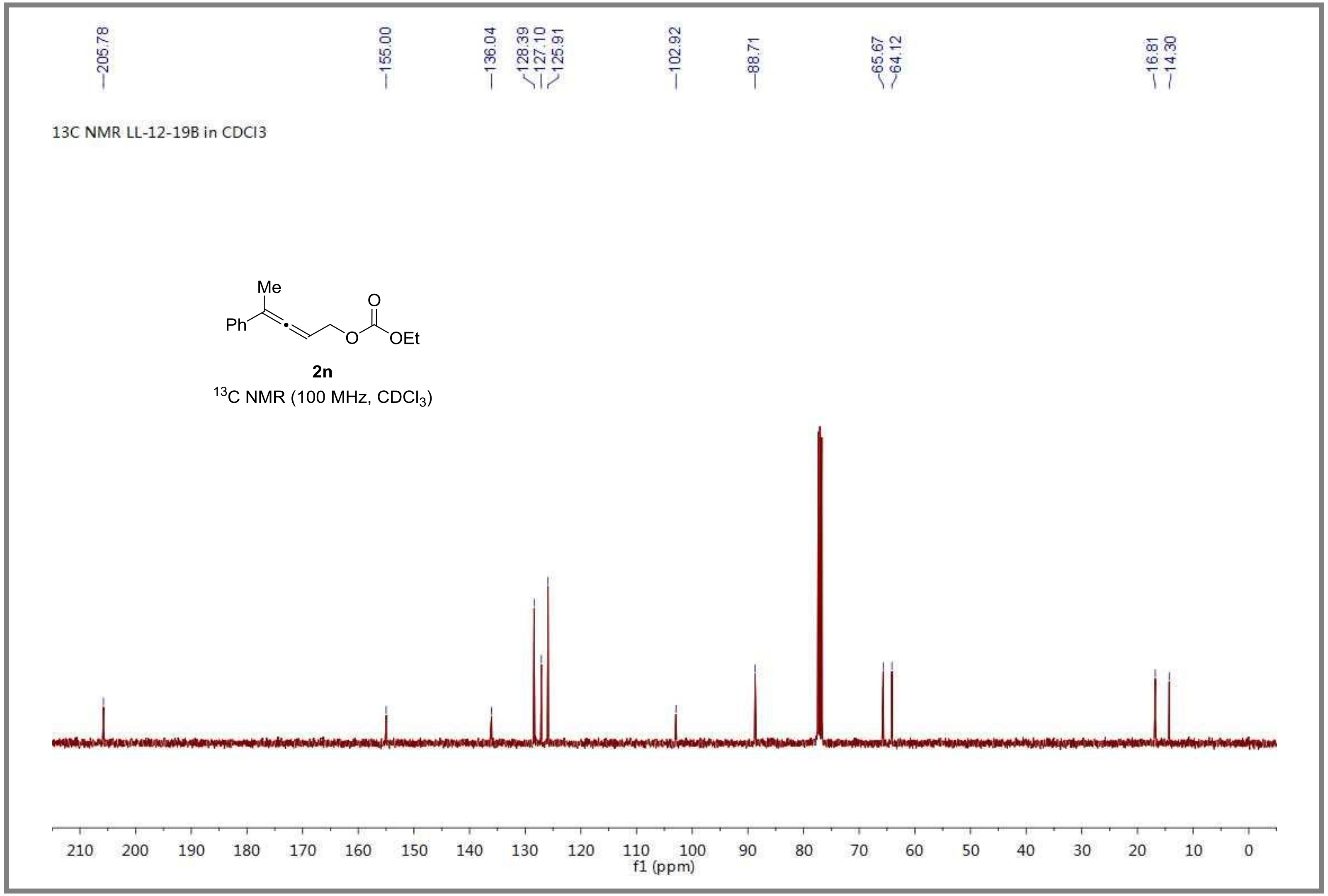
205.78
155.00
136.04
128.39
127.10
125.91
102.92
88.71
65.67
64.12
16.81
14.30
13C NMR LL-12-19B in CDCl3
Me
Ph
O
O
OEt
2n
13C NMR (100 MHz, CDCl3)
210 200 190 180 170 160 150 140 130 120 110 100 90 80 70 60 50 40 30 20 10 0
f1 (ppm)

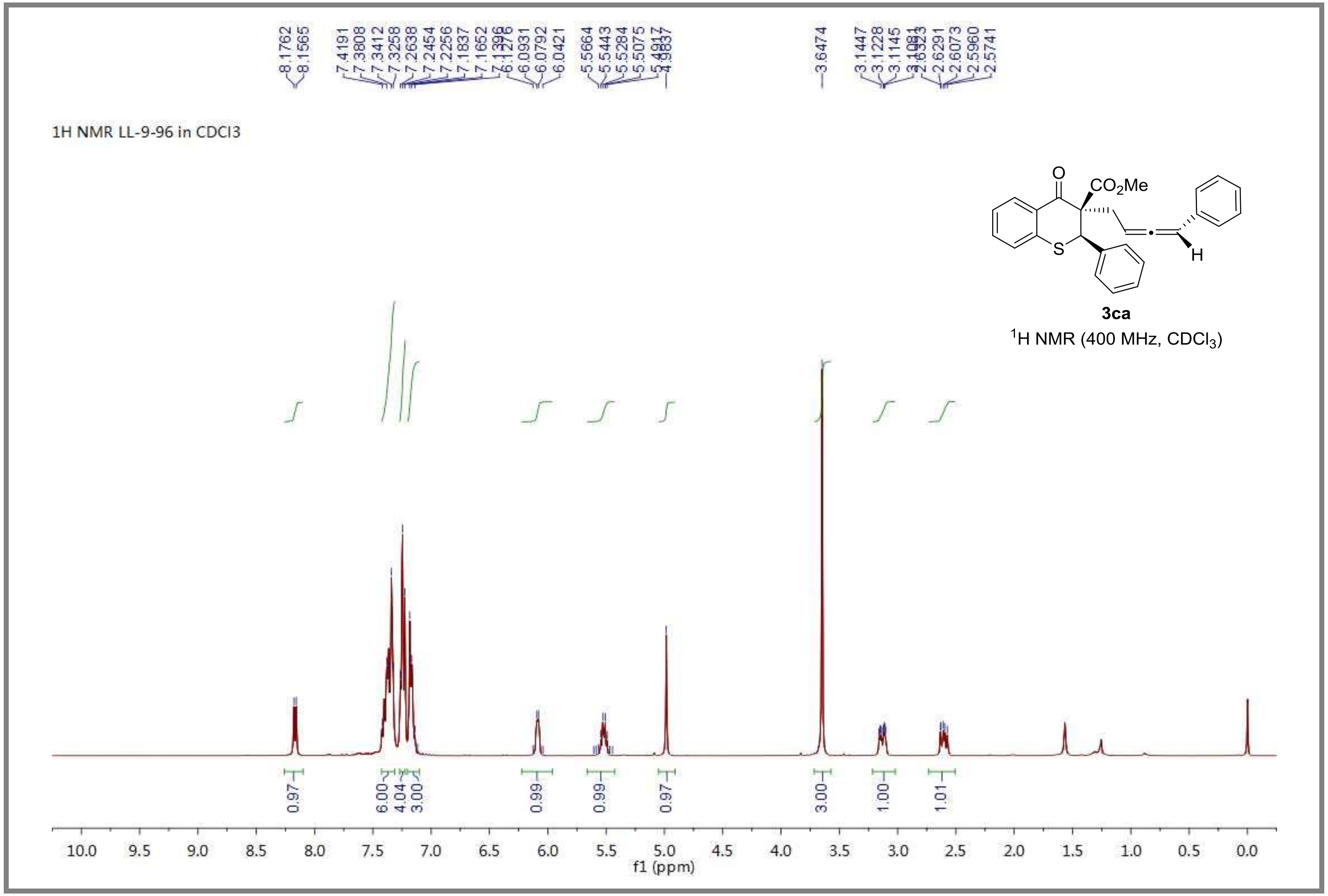
1H NMR LL-9-96 in CDCl3
8.1762
8.1565
7.4191
7.3808
7.3412
7.3258
7.2638
7.2454
7.2256
7.1837
7.1652
6.0931
6.0792
6.0421
5.5664
5.5443
5.5284
5.5075
4.9837
3.6474
3.1447
3.1228
3.1145
2.6291
2.6073
2.5960
2.5741
0.97
6.00
4.04
3.00
0.99
0.99
0.97
3.00
1.00
1.01
f1 (ppm)
CO2Me
O
S
H
3ca
1H NMR (400 MHz, CDCl3)

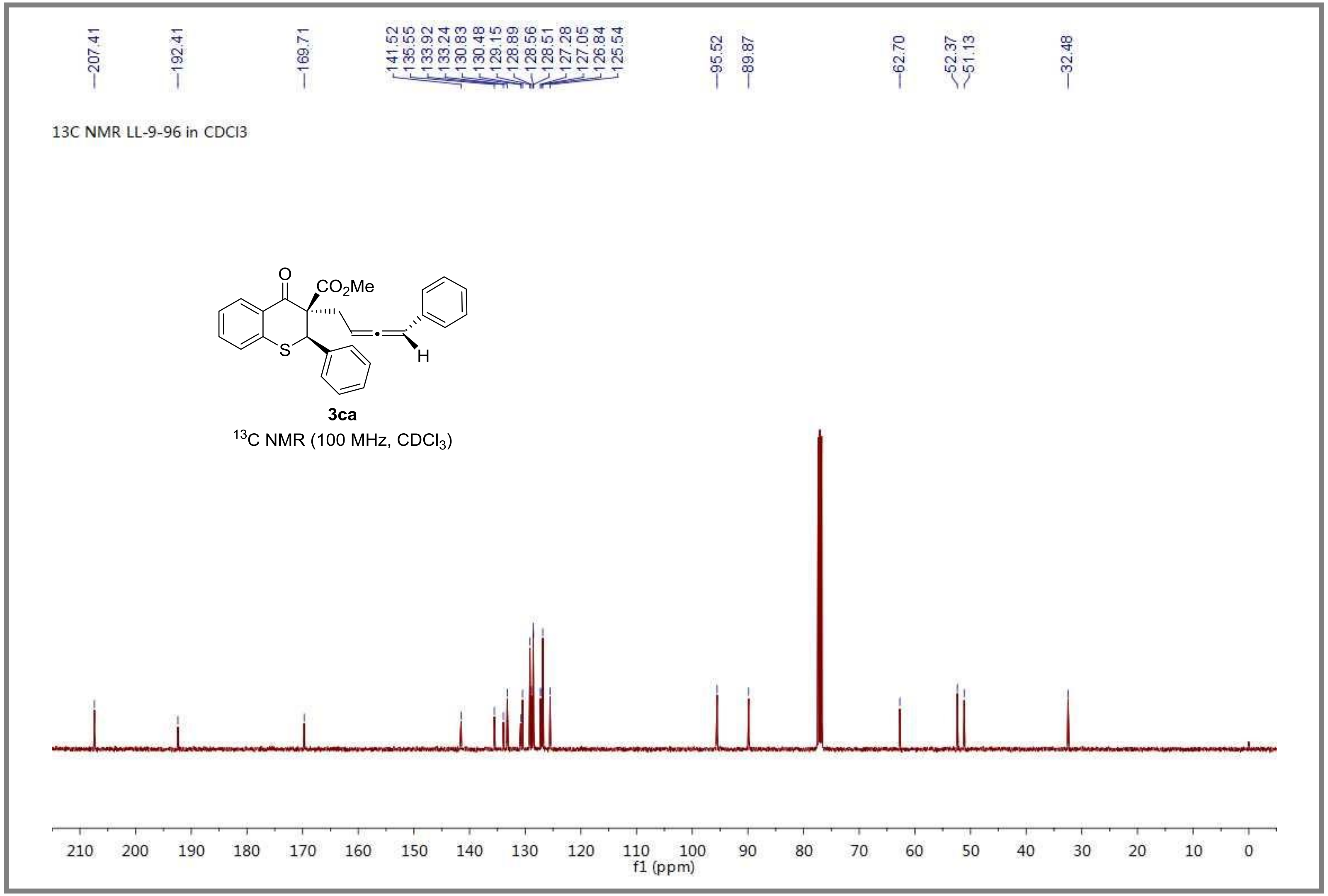
207.41
192.41
169.71
141.52
135.55
133.92
133.24
130.83
130.48
129.15
128.89
128.56
128.51
127.28
127.05
126.84
125.54
95.52
89.87
62.70
52.37
51.13
32.48
13C NMR LL-9-96 in CDCl3
CO_2Me
O
S
H
3ca
^{13}C NMR (100 MHz, $CDCl_3$)
210 200 190 180 170 160 150 140 130 120 110 100 90 80 70 60 50 40 30 20 10 0
f1 (ppm)

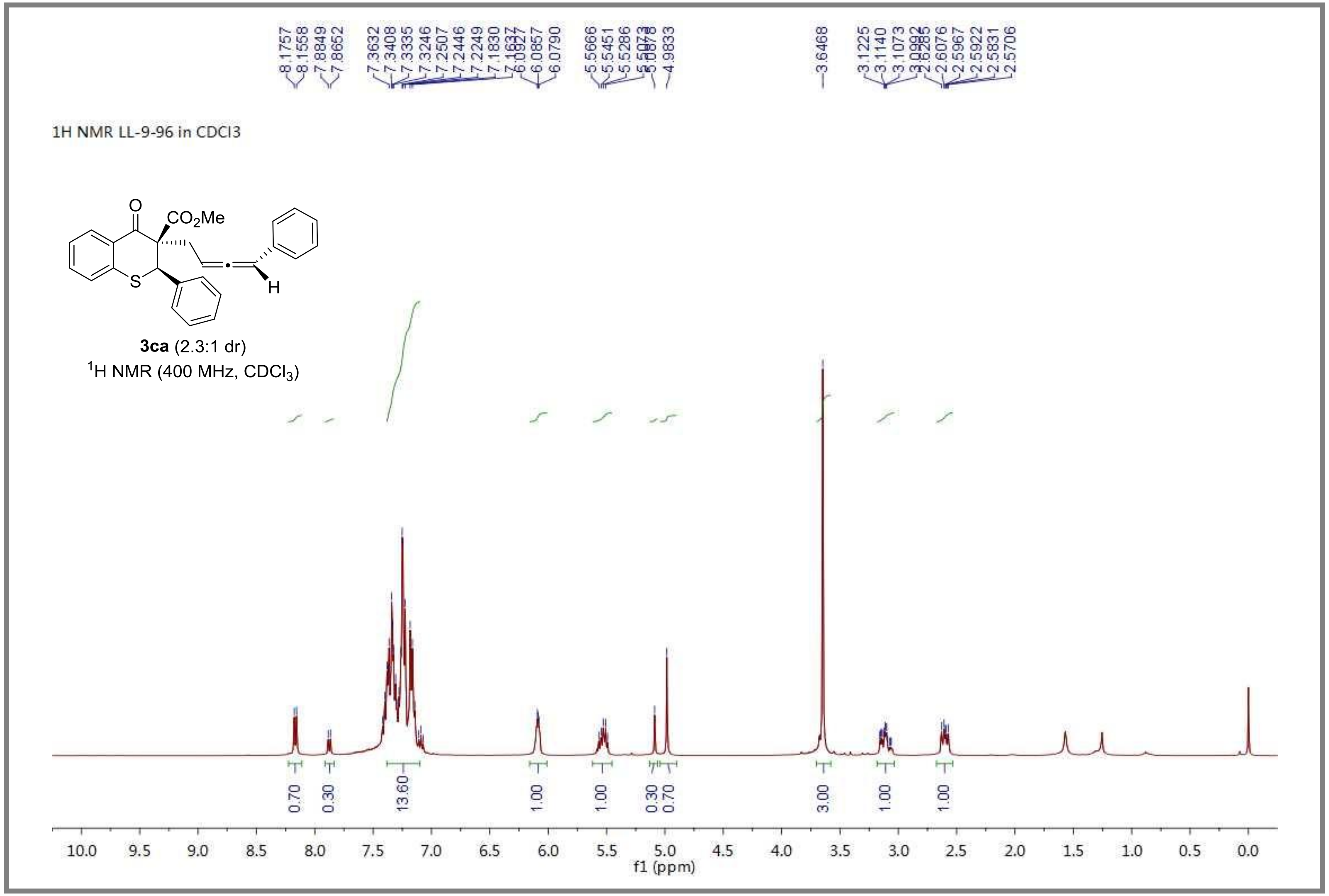
1H NMR LL-9-96 in CDCl3
3ca (2.3:1 dr)
1H NMR (400 MHz, CDCl3)
f1 (ppm)

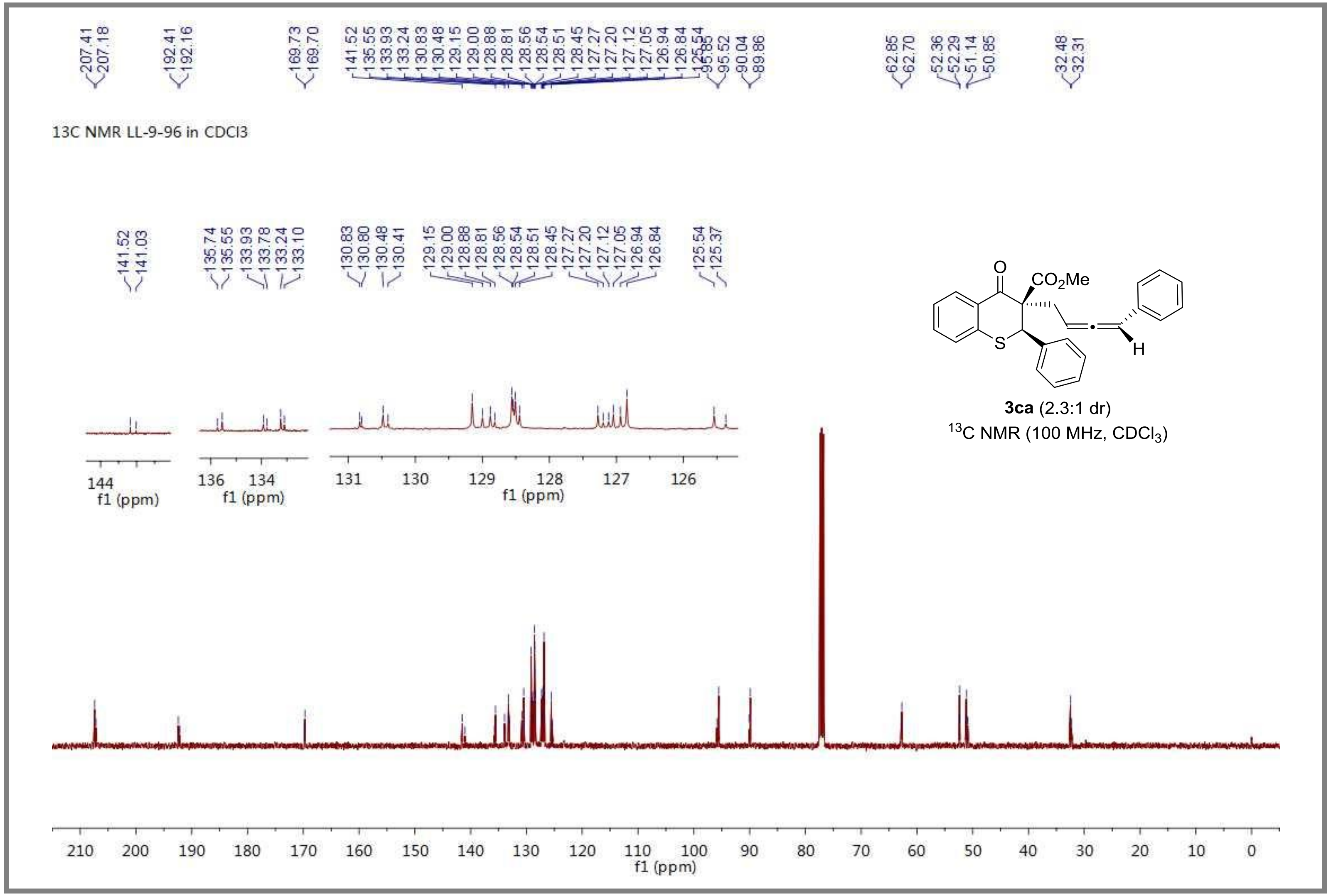
13C NMR LL-9-96 in CDCl3
3ca (2.3:1 dr)
13C NMR (100 MHz, CDCl3)
f1 (ppm)

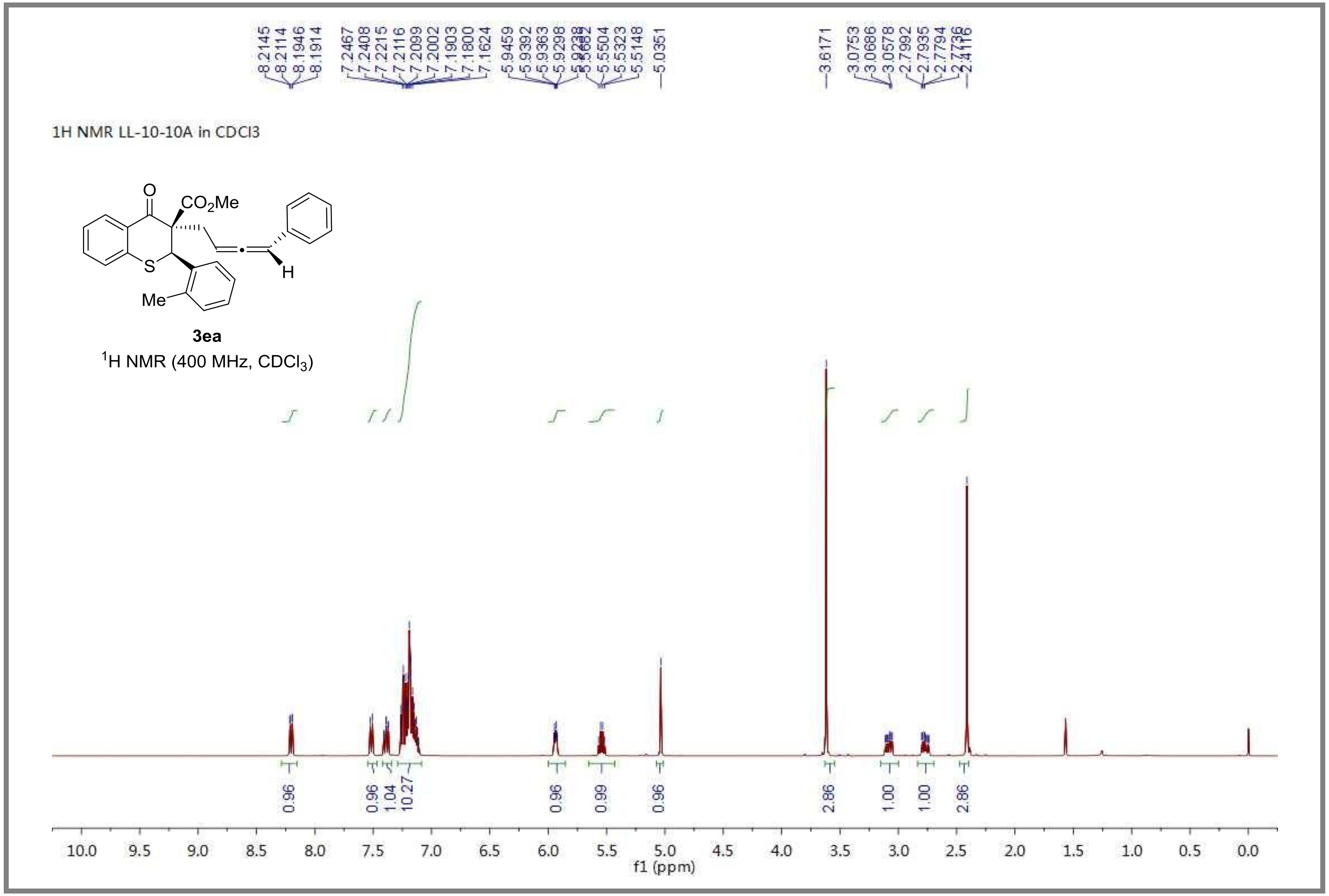
1H NMR LL-10-10A in CDCl3
CO2Me
O
S
Me
H
3ea
1H NMR (400 MHz, CDCl3)
8.2145
8.2114
8.1946
8.1914
7.2467
7.2408
7.2215
7.2116
7.2099
7.2002
7.1903
7.1800
7.1624
5.9459
5.9392
5.9363
5.9298
5.5504
5.5323
5.5148
5.0351
3.6171
3.0753
3.0686
3.0578
2.7992
2.7935
2.7794
2.4116
0.96
0.96
1.04
10.27
0.96
0.99
0.96
2.86
1.00
1.00
2.86
10.0
9.5
9.0
8.5
8.0
7.5
7.0
6.5
6.0
5.5
5.0
4.5
4.0
3.5
3.0
2.5
2.0
1.5
1.0
0.5
0.0
f1 (ppm)

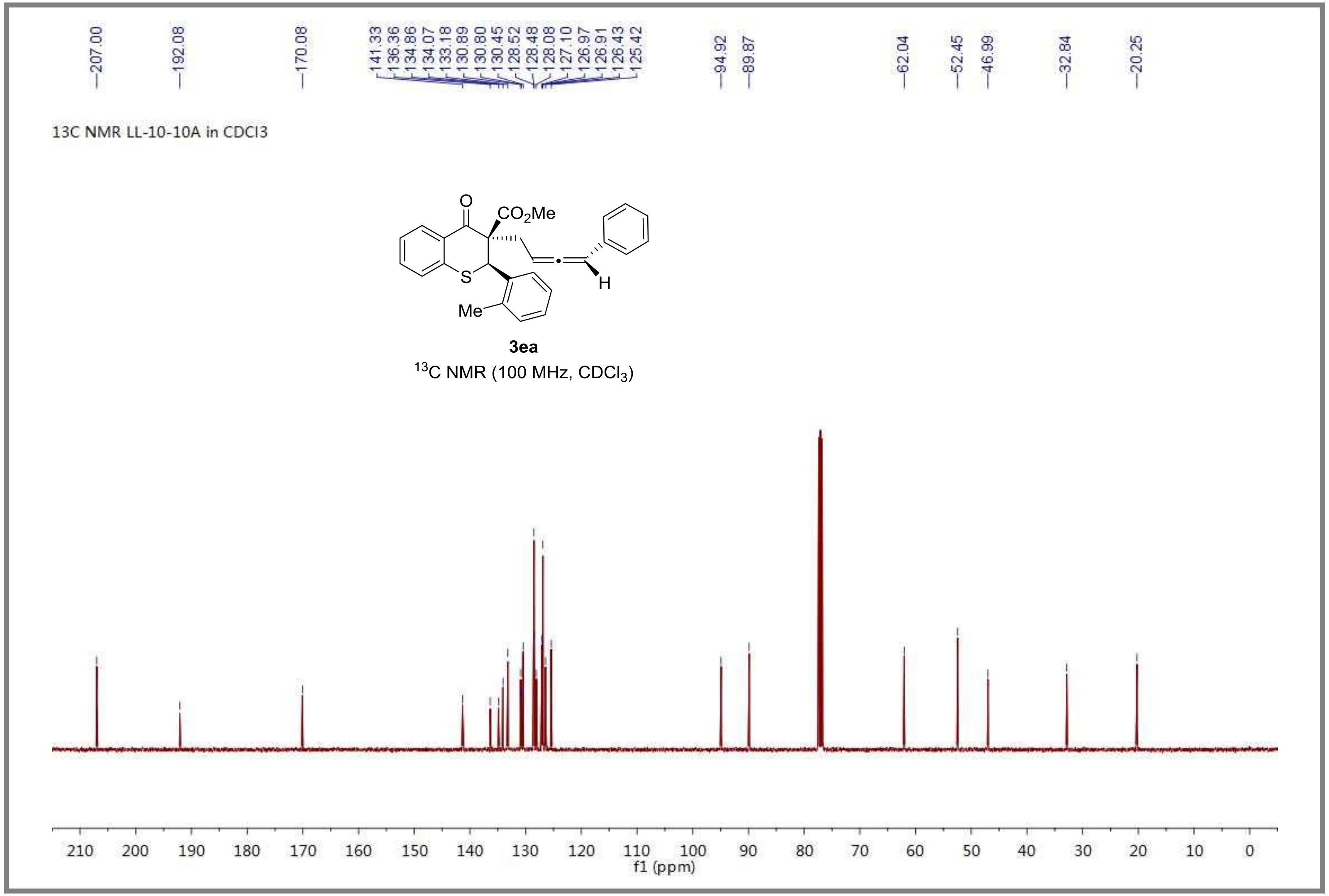

207.00
192.08
170.08
141.33
136.36
134.86
134.07
133.18
130.89
130.80
130.45
128.52
128.48
128.08
127.10
126.97
126.91
126.43
125.42
94.92
89.87
62.04
52.45
46.99
32.84
20.25
13C NMR LL-10-10A in CDCl3
CO2Me
S
Me
H
3ea
13C NMR (100 MHz, CDCl3)
210 200 190 180 170 160 150 140 130 120 110 100 90 80 70 60 50 40 30 20 10 0
f1 (ppm)

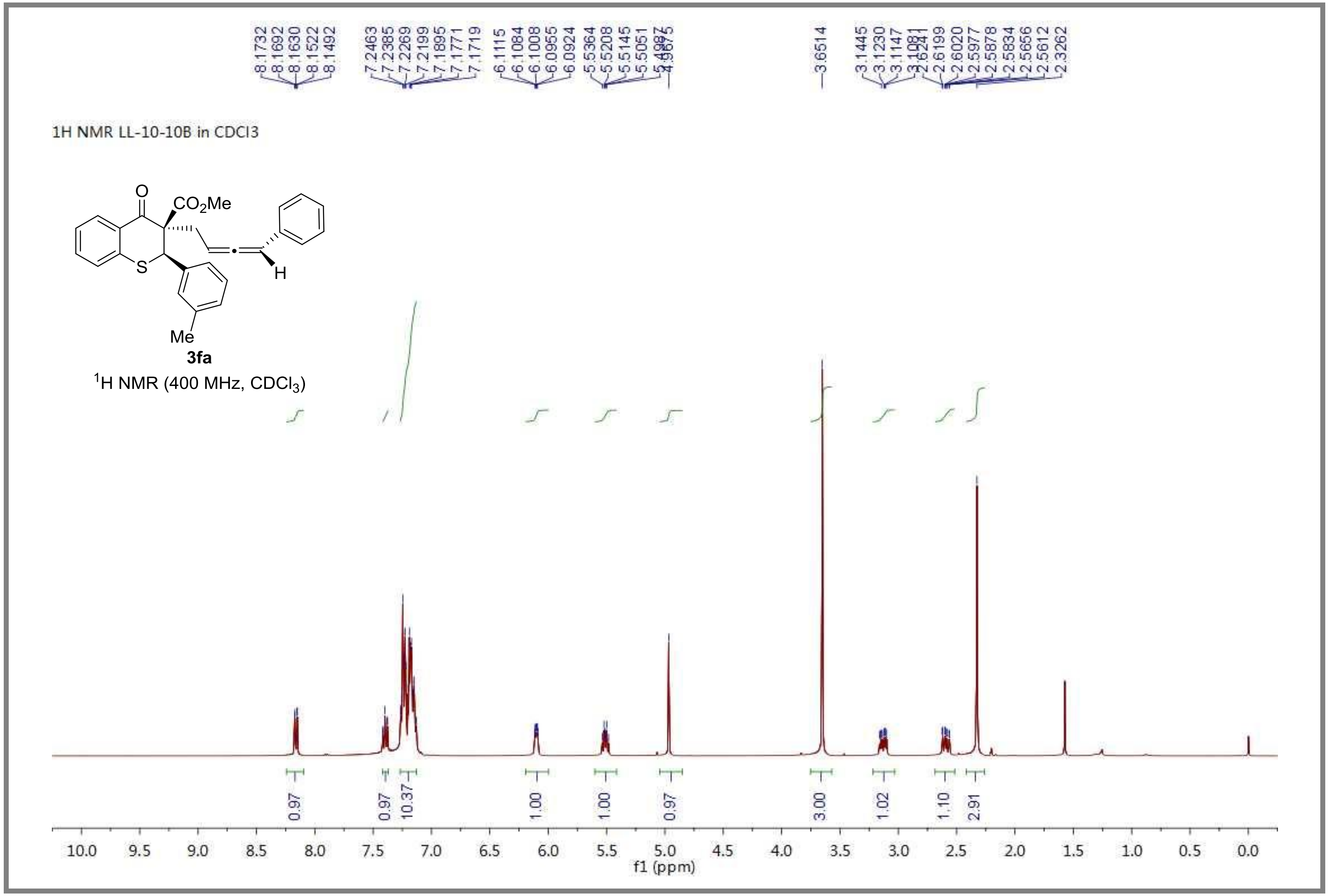
1H NMR LL-10-10B in CDCl3
CO2Me
O
S
H
Me
3fa
1H NMR (400 MHz, CDCl3)
8.1732
8.1692
8.1630
8.1522
8.1492
7.2463
7.2385
7.2269
7.2199
7.1895
7.1771
7.1719
6.1115
6.1084
6.1008
6.0955
6.0924
5.5364
5.5208
5.5145
5.5051
3.6514
3.1445
3.1230
3.1147
2.6199
2.6020
2.5977
2.5878
2.5834
2.5656
2.5612
2.3262
0.97
0.97
10.37
1.00
1.00
0.97
3.00
1.02
1.10
2.91
f1 (ppm)

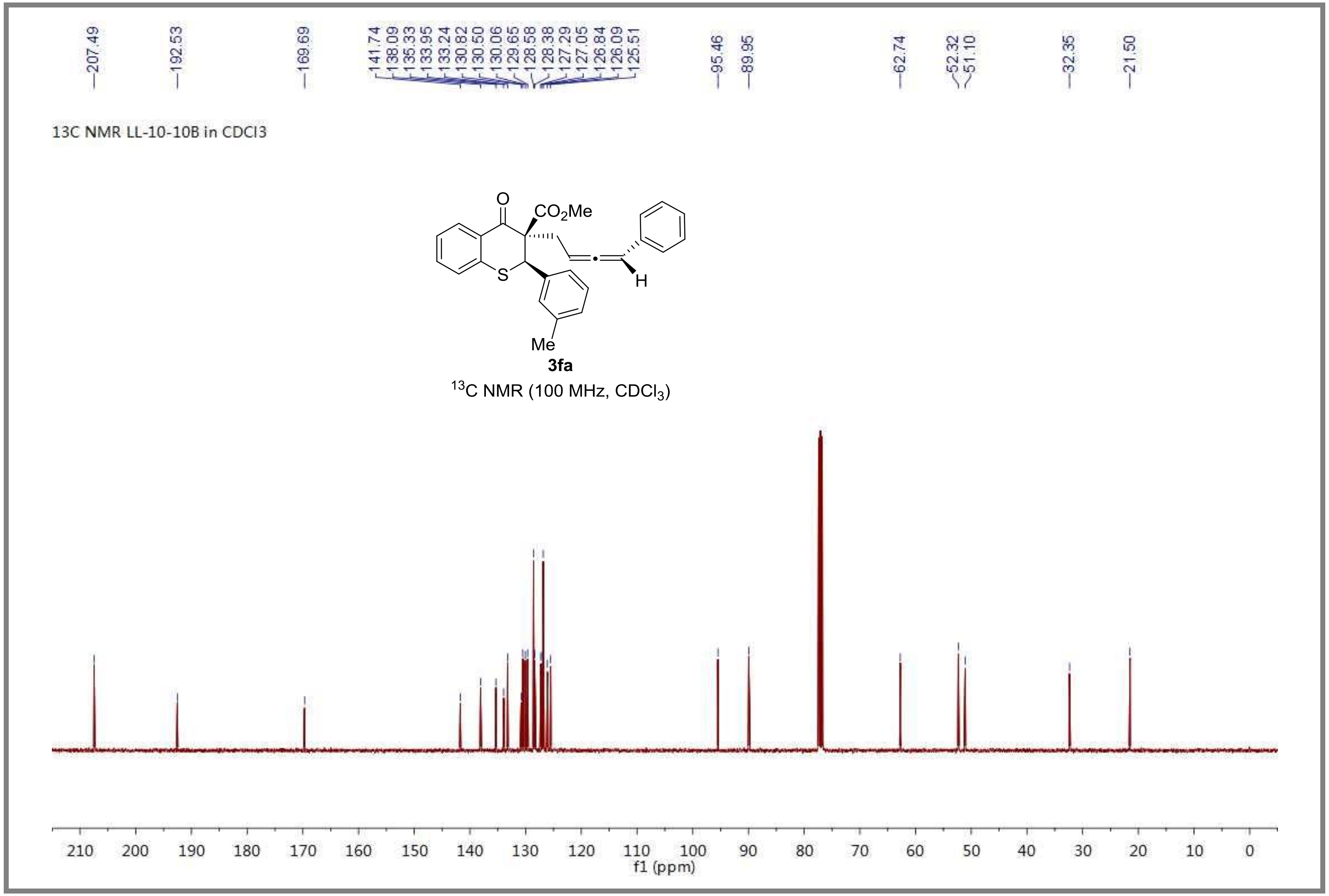
207.49
192.53
169.69
141.74
138.09
135.33
133.95
133.24
130.82
130.50
130.06
129.65
128.58
128.38
127.29
127.05
126.84
126.09
125.51
95.46
89.95
62.74
52.32
51.10
32.35
21.50
13C NMR LL-10-10B in CDCl3
O
CO2Me
S
H
Me
3fa
13C NMR (100 MHz, CDCl3)
210 200 190 180 170 160 150 140 130 120 110 100 90 80 70 60 50 40 30 20 10 0
f1 (ppm)

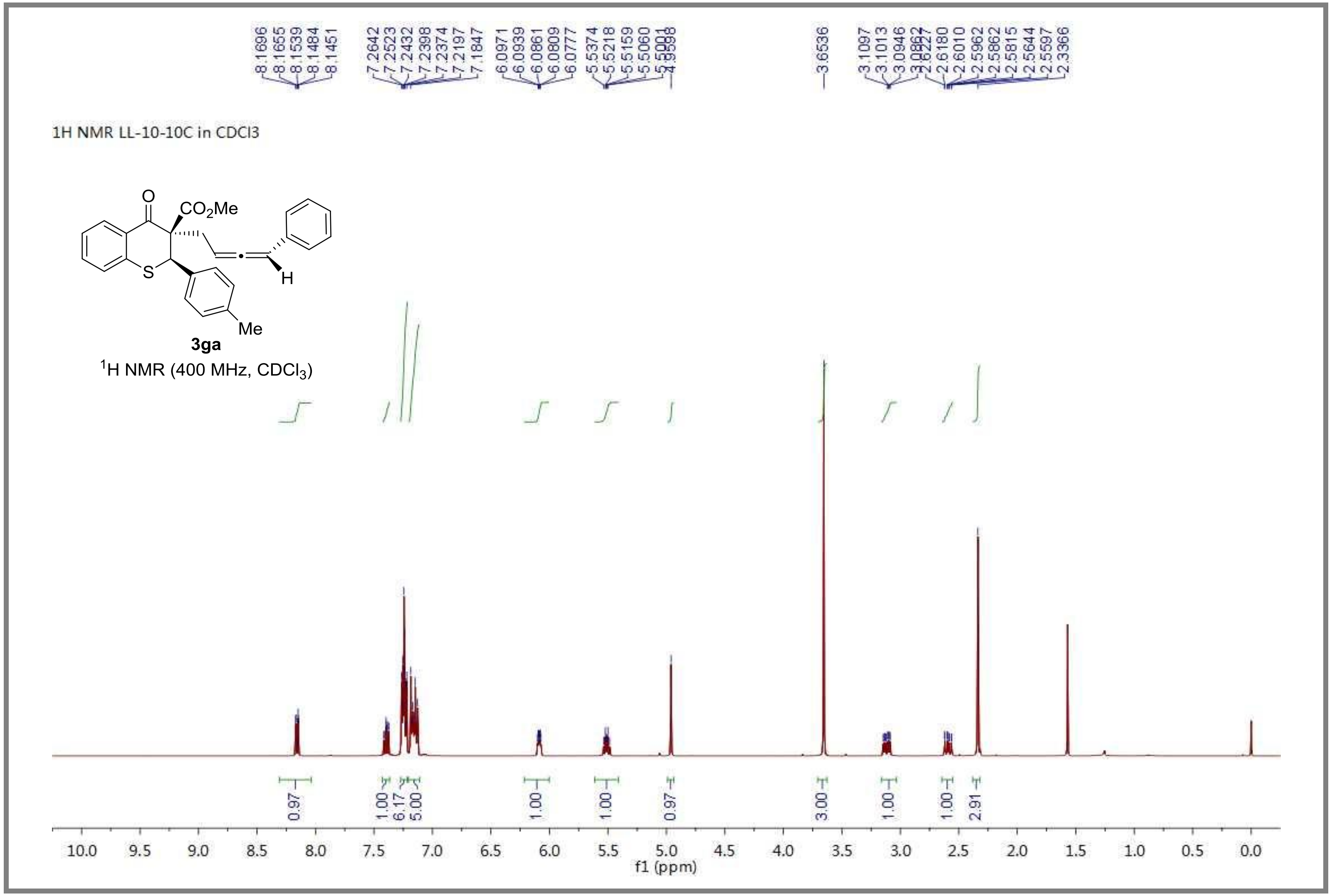

1H NMR LL-10-10C in CDCl3
3ga
^{1}H NMR (400 MHz, CDCl$_3$)
f1 (ppm)

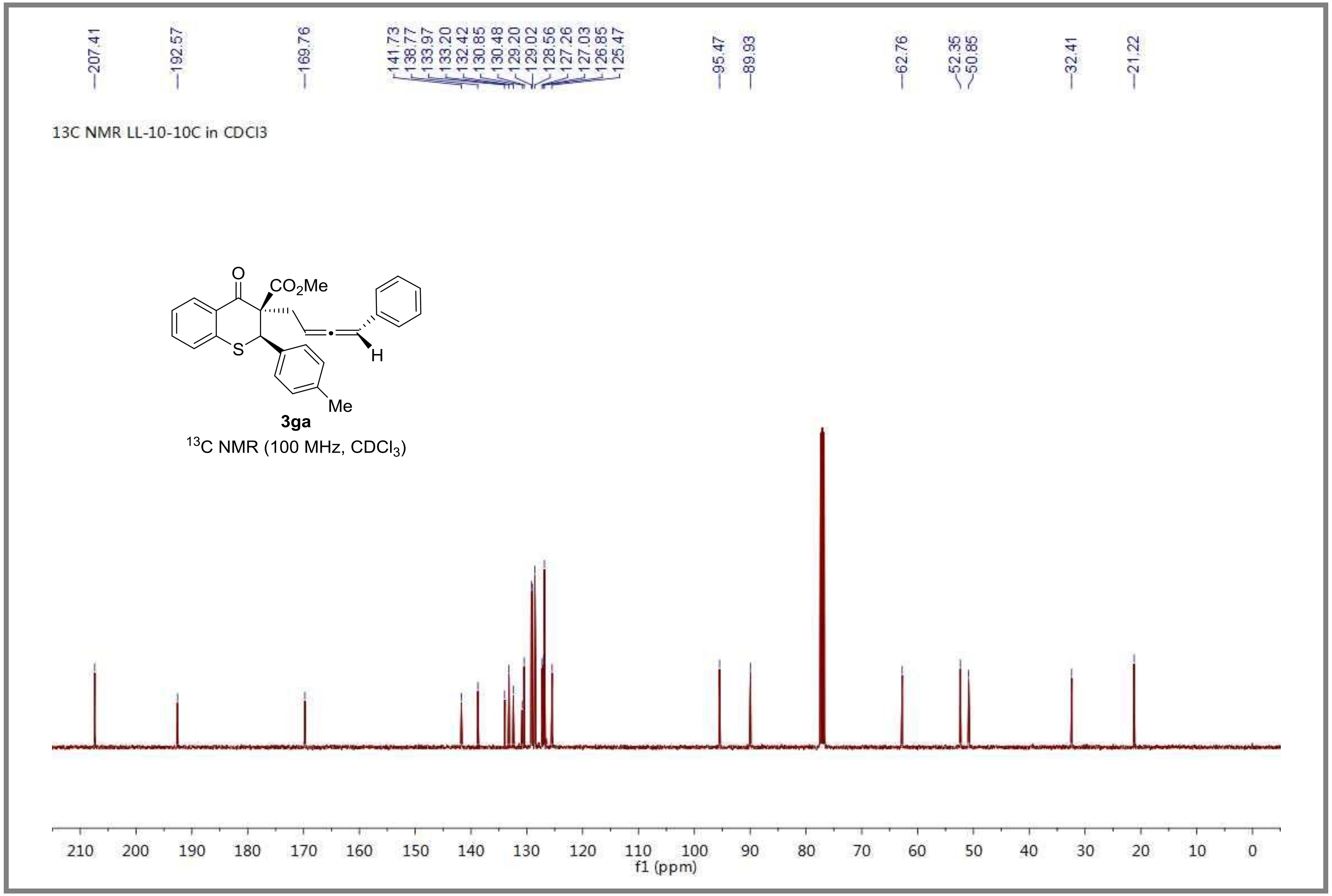
207.41
192.57
169.76
141.73
138.77
133.97
133.20
132.42
130.85
130.48
129.20
129.02
128.56
127.26
127.03
126.85
125.47
95.47
89.93
62.76
52.35
50.85
32.41
21.22
13C NMR LL-10-10C in CDCl3
O
CO2Me
S
H
Me
3ga
13C NMR (100 MHz, CDCl3)
210 200 190 180 170 160 150 140 130 120 110 100 90 80 70 60 50 40 30 20 10 0
f1 (ppm)

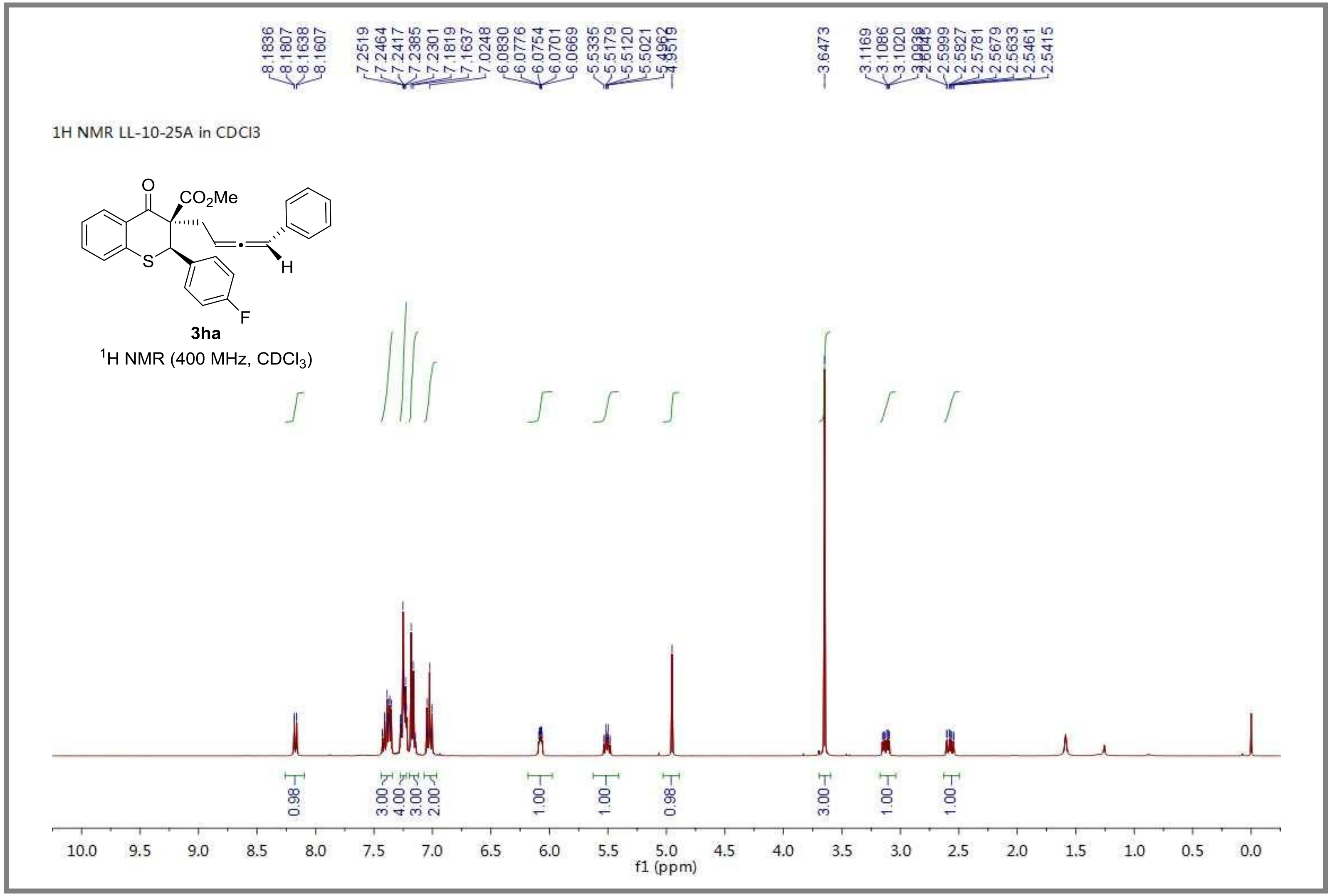
8.1836
8.1807
8.1638
8.1607
7.2519
7.2464
7.2417
7.2385
7.2301
7.1819
7.1637
7.0248
6.0830
6.0776
6.0754
6.0701
6.0669
5.5335
5.5179
5.5120
5.5021
5.4962
4.9519
3.6473
3.1169
3.1086
3.1020
2.5999
2.5827
2.5781
2.5679
2.5633
2.5461
2.5415
1H NMR LL-10-25A in CDCl3
O
CO2Me
S
H
F
3ha
1H NMR (400 MHz, CDCl3)
0.98
3.00
4.00
3.00
2.00
1.00
1.00
0.98
3.00
1.00
1.00
10.0
9.5
9.0
8.5
8.0
7.5
7.0
6.5
6.0
5.5
5.0
4.5
4.0
3.5
3.0
2.5
2.0
1.5
1.0
0.5
0.0
f1 (ppm)

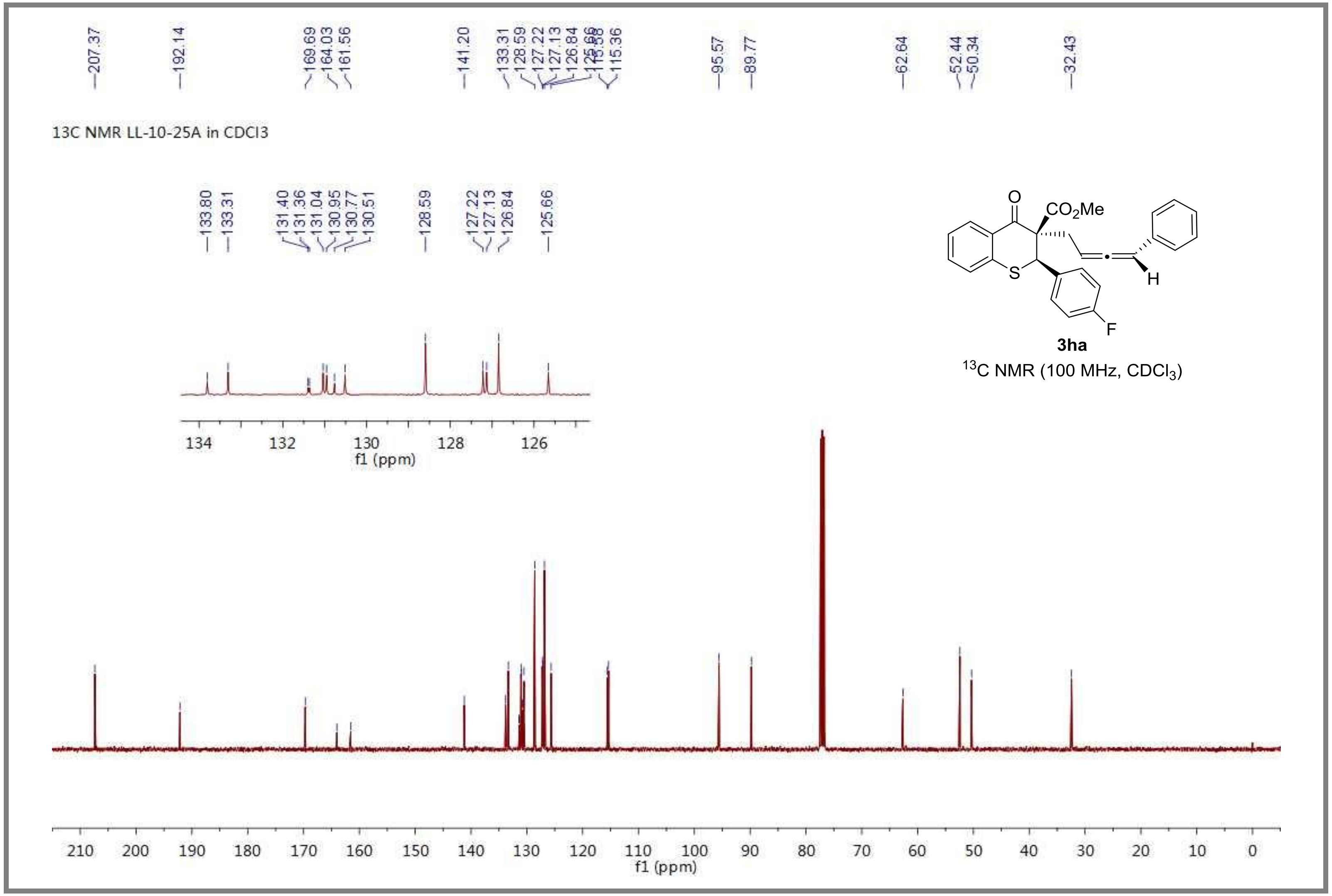

207.37
192.14
169.69
164.03
161.56
141.20
133.31
128.59
127.22
127.13
126.84
115.36
95.57
89.77
62.64
52.44
50.34
32.43
13C NMR LL-10-25A in CDCl3
133.80
133.31
131.40
131.36
131.04
130.95
130.77
130.51
128.59
127.22
127.13
126.84
125.66
134
132
130
128
126
f1 (ppm)
O
CO2Me
S
H
F
3ha
13C NMR (100 MHz, CDCl3)
210
200
190
180
170
160
150
140
130
120
110
100
90
80
70
60
50
40
30
20
10
0
f1 (ppm)

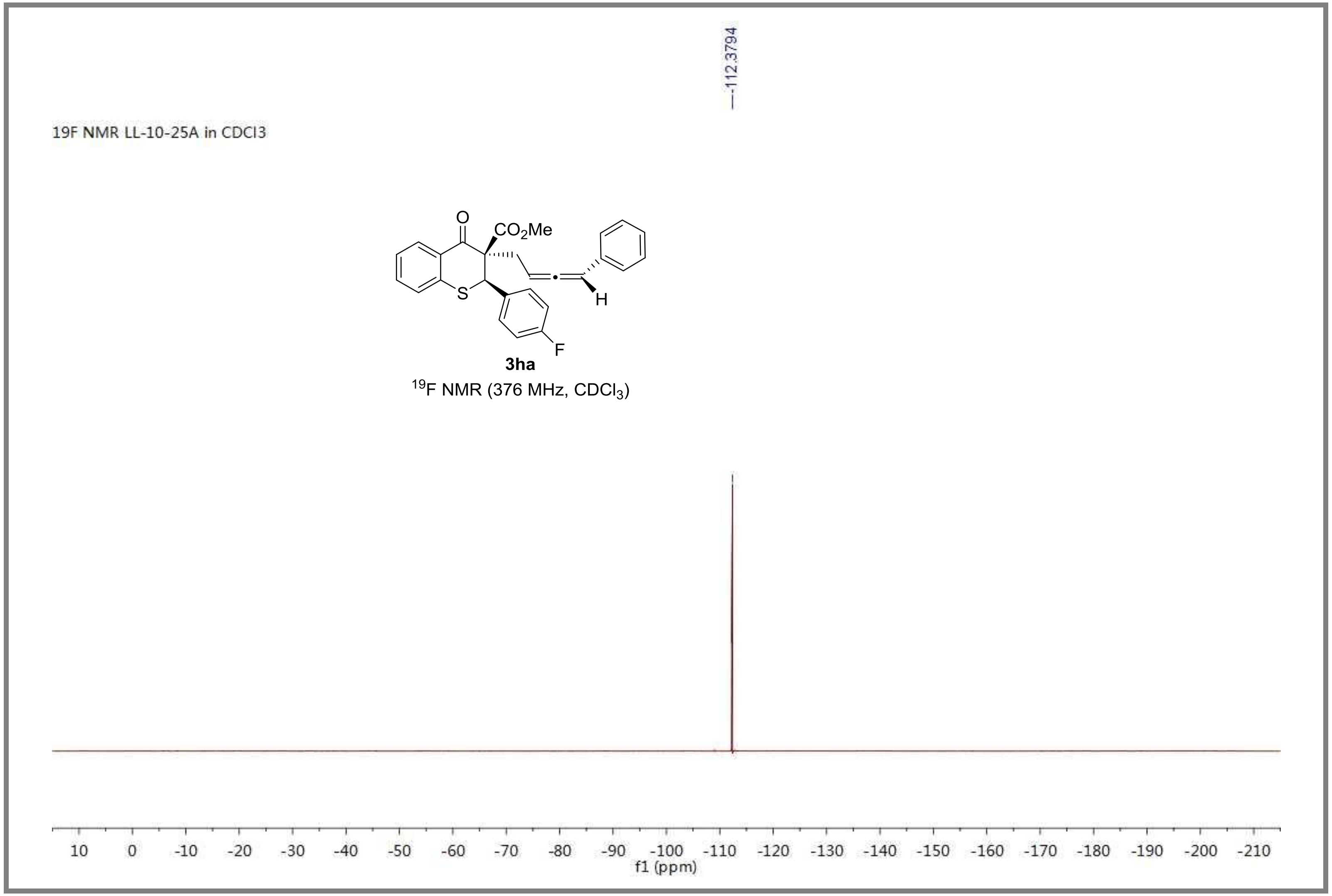

-112.3794
19F NMR LL-10-25A in CDCl3
O
CO2Me
S
H
F
3ha
19F NMR (376 MHz, CDCl3)
10 0 -10 -20 -30 -40 -50 -60 -70 -80 -90 -100 -110 -120 -130 -140 -150 -160 -170 -180 -190 -200 -210
f1 (ppm)

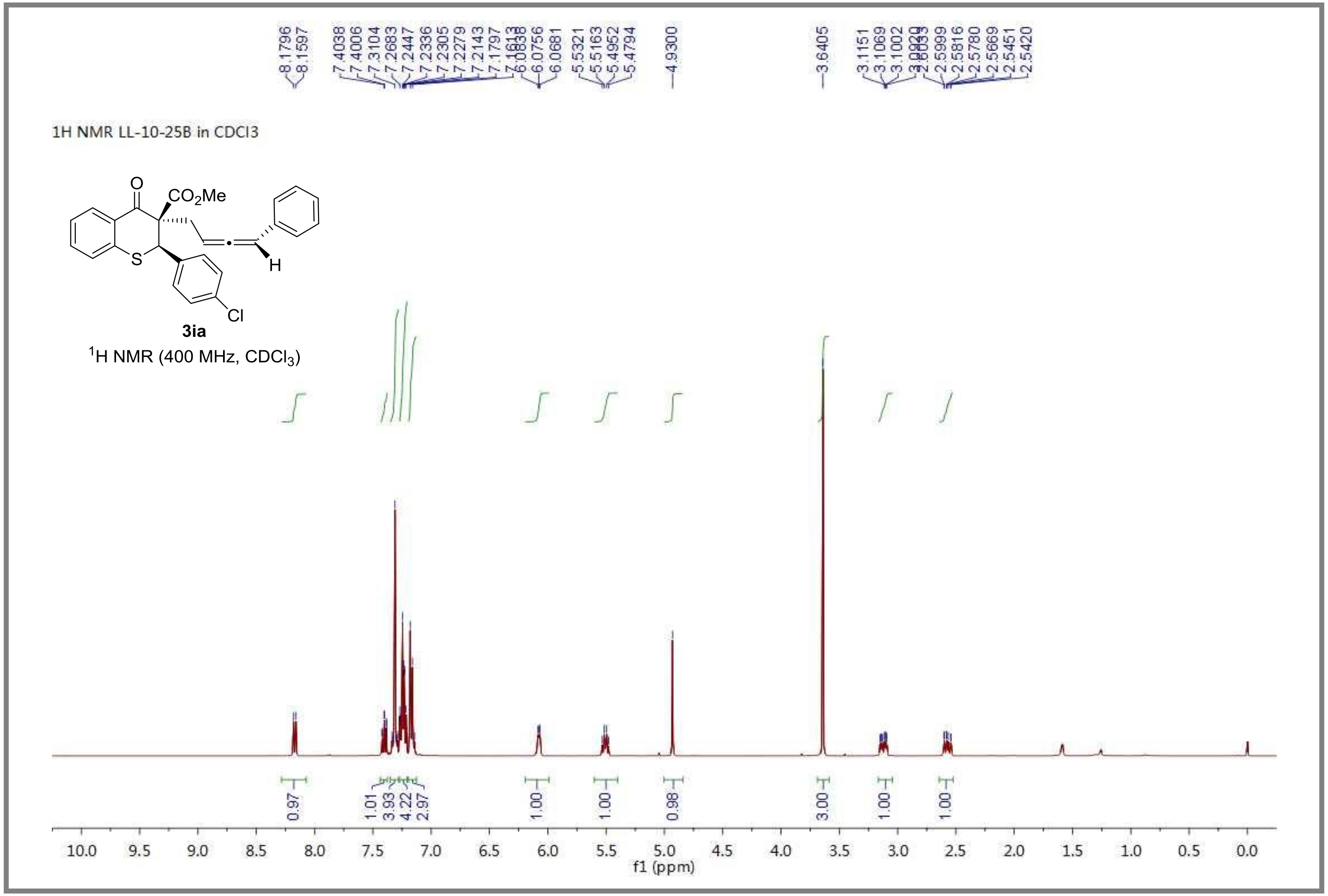
1H NMR LL-10-25B in CDCl3
3ia
^{1}H NMR (400 MHz, $CDCl_3$)
f1 (ppm)

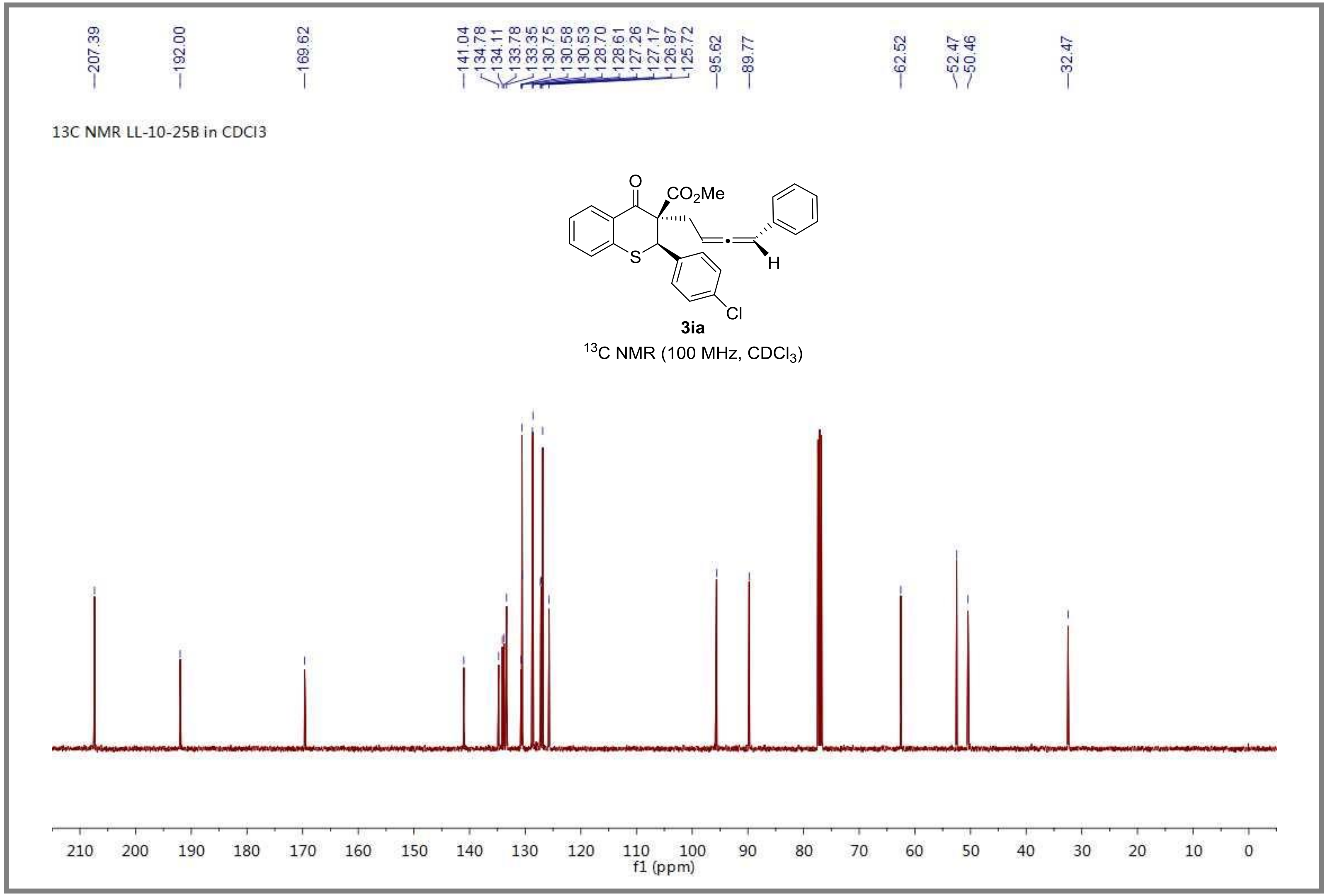

13C NMR LL-10-25B in CDCl3
207.39
192.00
169.62
141.04
134.78
134.11
133.78
133.35
130.75
130.58
130.53
128.70
128.61
127.26
127.17
126.87
125.72
95.62
89.77
62.52
52.47
50.46
32.47
O
CO2Me
S
H
Cl
3ia
13C NMR (100 MHz, CDCl3)
210 200 190 180 170 160 150 140 130 120 110 100 90 80 70 60 50 40 30 20 10 0
f1 (ppm)

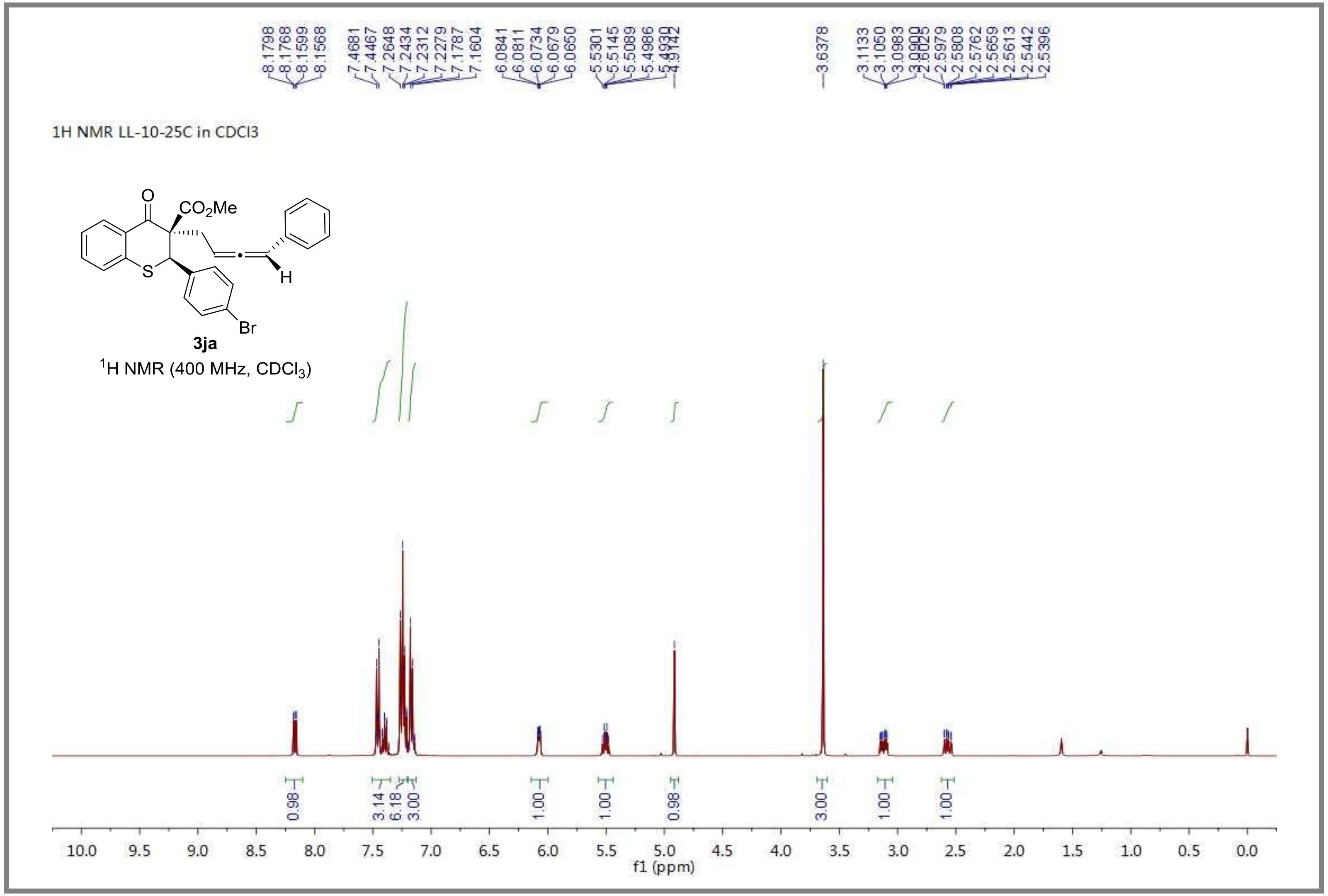

8.1798
8.1768
8.1599
8.1568
7.4681
7.4467
7.2648
7.2434
7.2312
7.2279
7.1787
7.1604
6.0841
6.0811
6.0734
6.0679
6.0650
5.5301
5.5145
5.5089
5.4986
3.6378
3.1133
3.1050
3.0983
2.5979
2.5808
2.5762
2.5659
2.5613
2.5442
2.5396
1H NMR LL-10-25C in CDCl3
O
CO2Me
S
H
Br
3ja
1H NMR (400 MHz, CDCl3)
0.98
3.14
6.18
3.00
1.00
1.00
0.98
3.00
1.00
1.00
10.0
9.5
9.0
8.5
8.0
7.5
7.0
6.5
6.0
5.5
5.0
4.5
4.0
3.5
3.0
2.5
2.0
1.5
1.0
0.5
0.0
f1 (ppm)

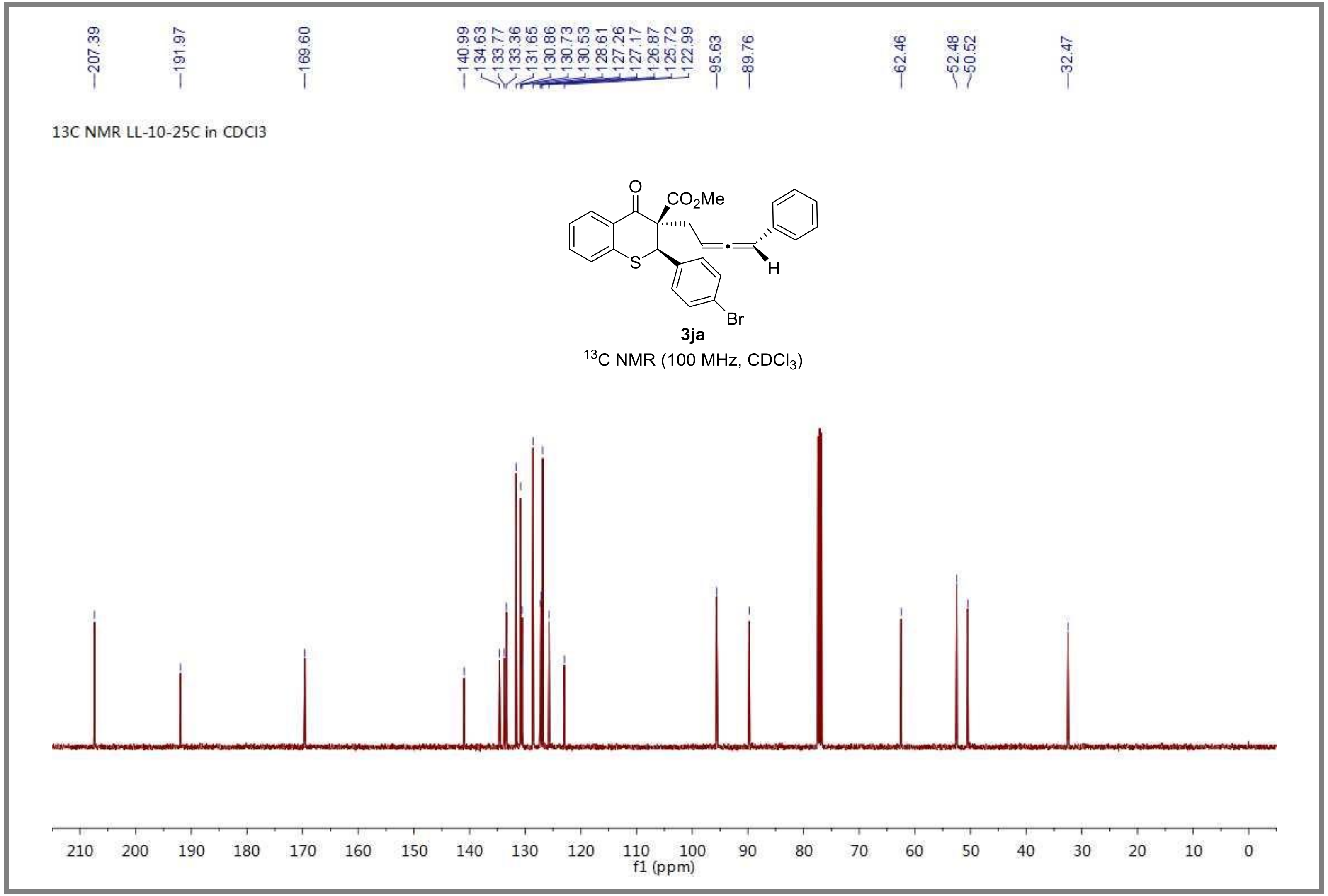

207.39
191.97
169.60
140.99
134.63
133.77
133.36
131.65
130.86
130.73
130.53
128.61
127.26
127.17
126.87
125.72
122.99
95.63
89.76
62.46
52.48
50.52
32.47
13C NMR LL-10-25C in CDCl3
O
CO_2Me
S
H
Br
3ja
^{13}C NMR (100 MHz, $CDCl_3$)
210
200
190
180
170
160
150
140
130
120
110
100
90
80
70
60
50
40
30
20
10
0
f1 (ppm)

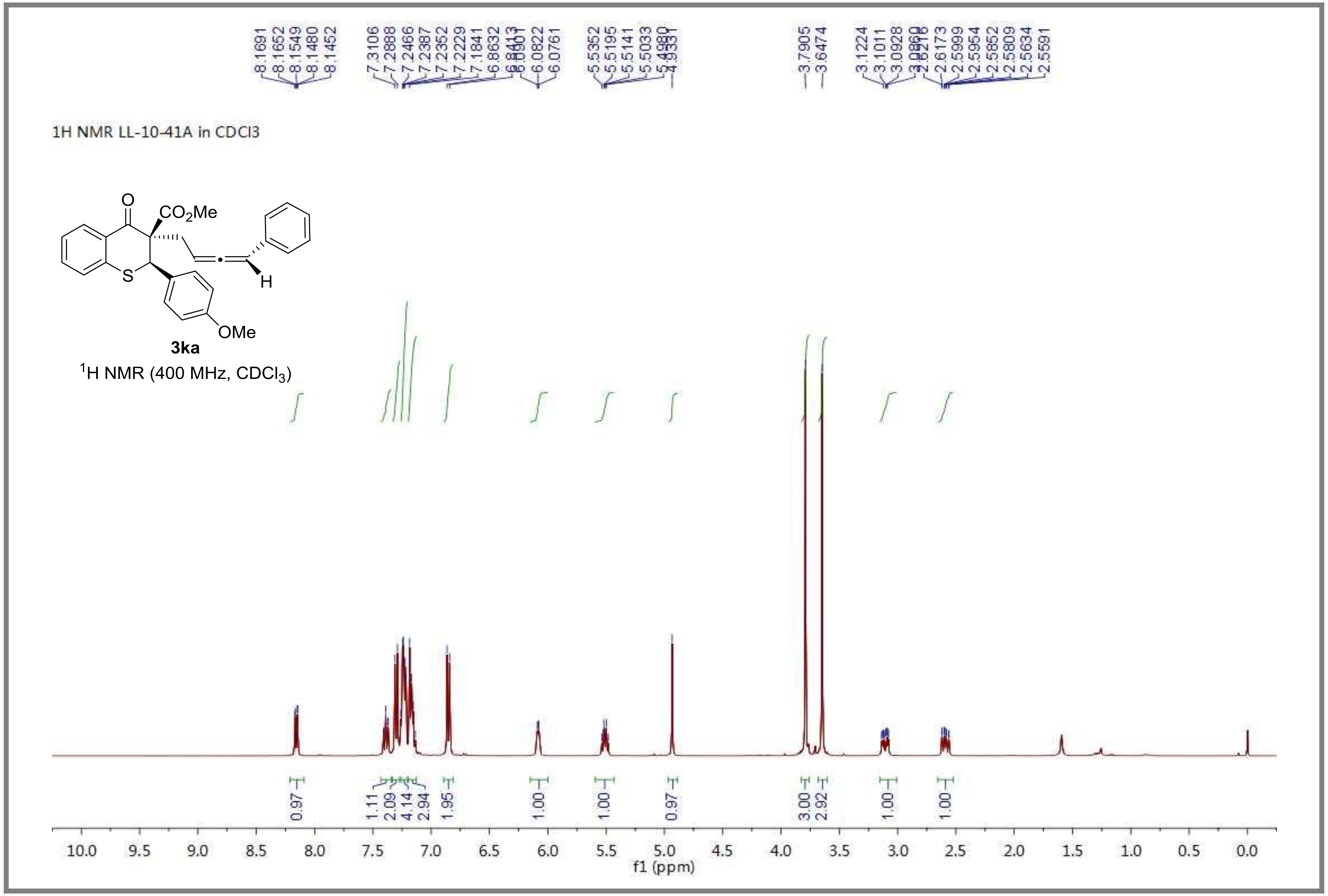
1H NMR LL-10-41A in CDCl3
CO2Me
OMe
H
S
O
3ka
1H NMR (400 MHz, CDCl3)
f1 (ppm)

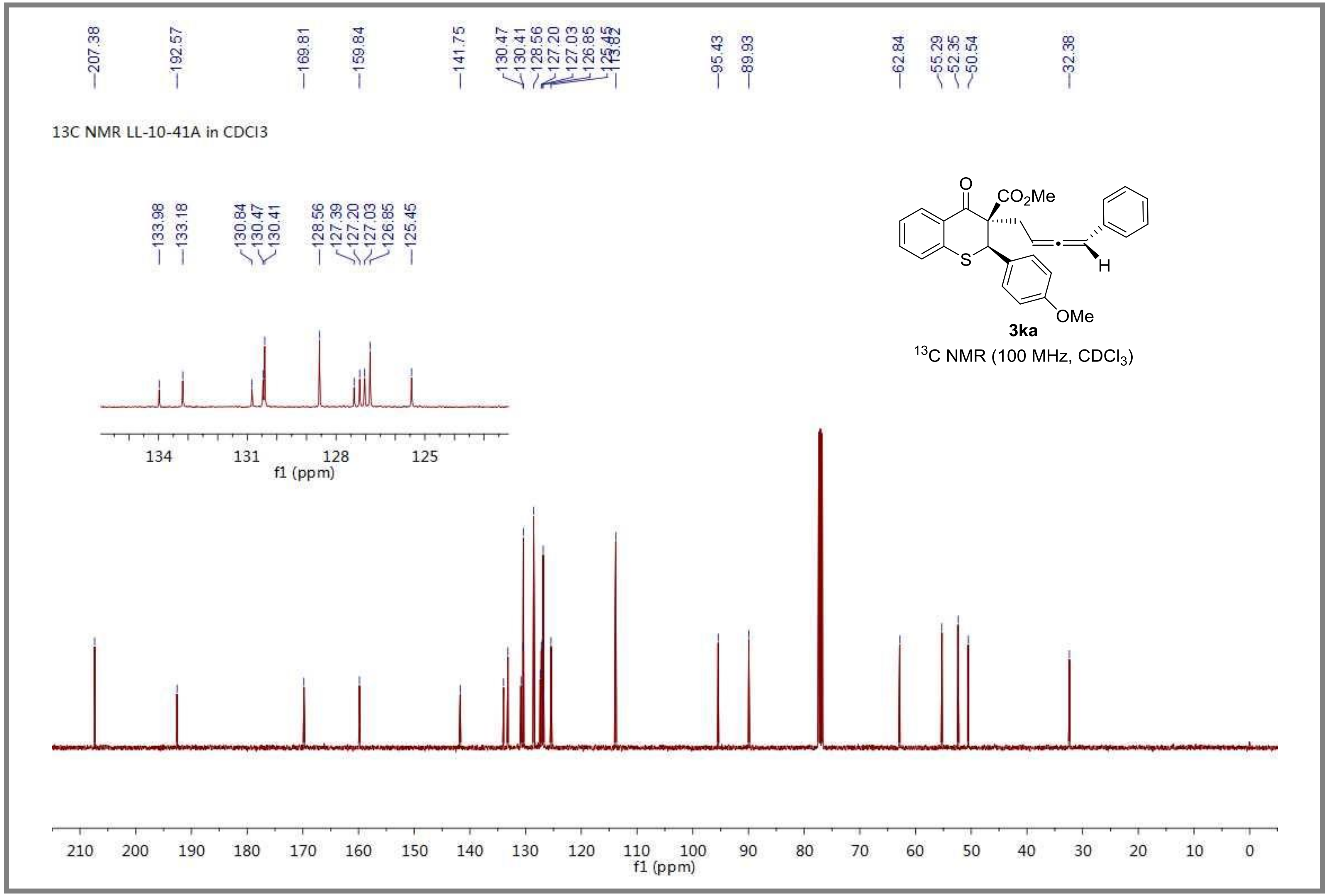
207.38
192.57
169.81
159.84
141.75
130.47
130.41
128.56
127.20
127.03
126.85
125.45
113.82
95.43
89.93
62.84
55.29
52.35
50.54
32.38
13C NMR LL-10-41A in CDCl3
133.98
133.18
130.84
130.47
130.41
128.56
127.39
127.20
127.03
126.85
125.45
134
131
128
125
f1 (ppm)
O
CO2Me
S
H
OMe
3ka
13C NMR (100 MHz, CDCl3)
210
200
190
180
170
160
150
140
130
120
110
100
90
80
70
60
50
40
30
20
10
0
f1 (ppm)

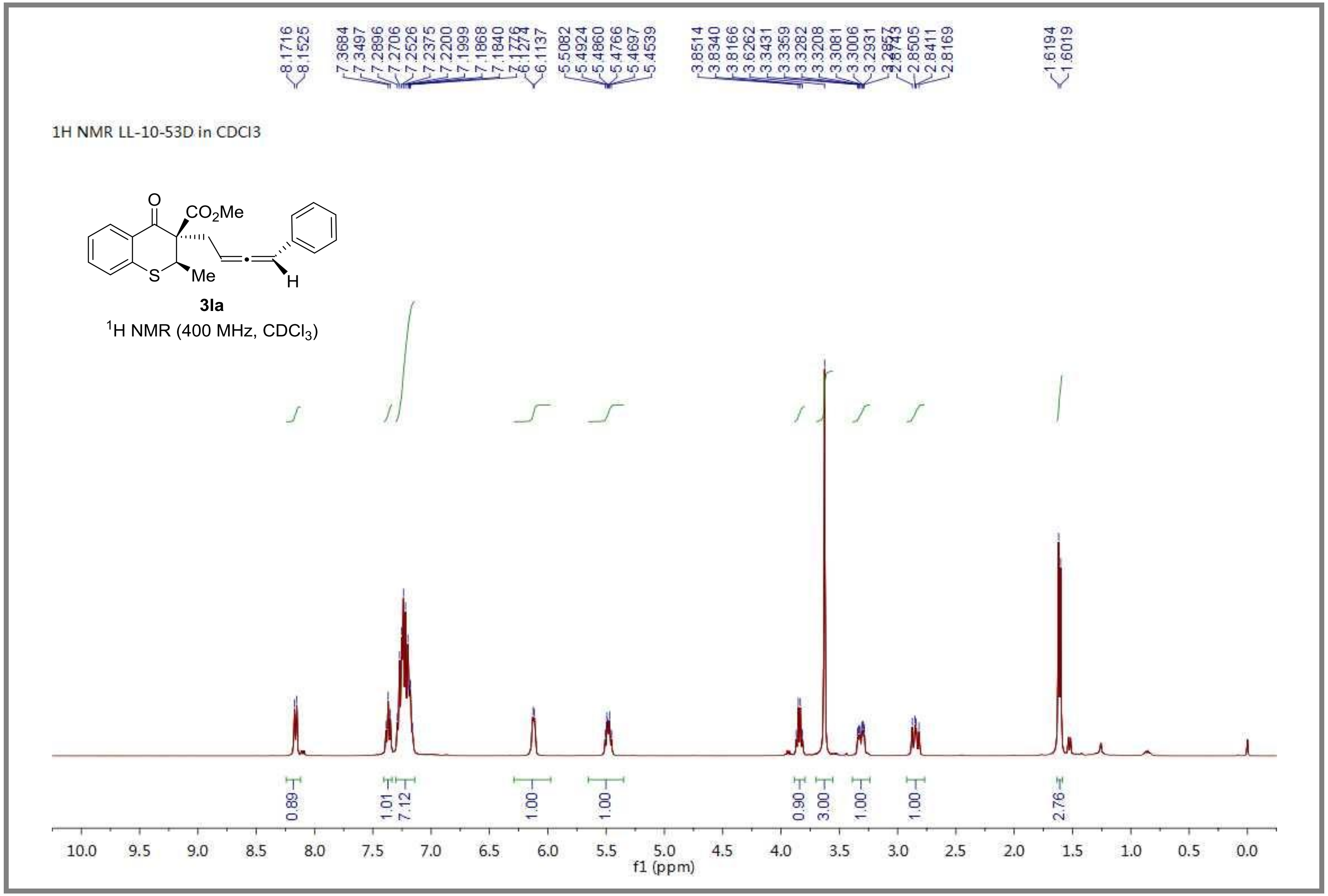

1H NMR LL-10-53D in CDCl3
3la
¹H NMR (400 MHz, CDCl₃)
f1 (ppm)

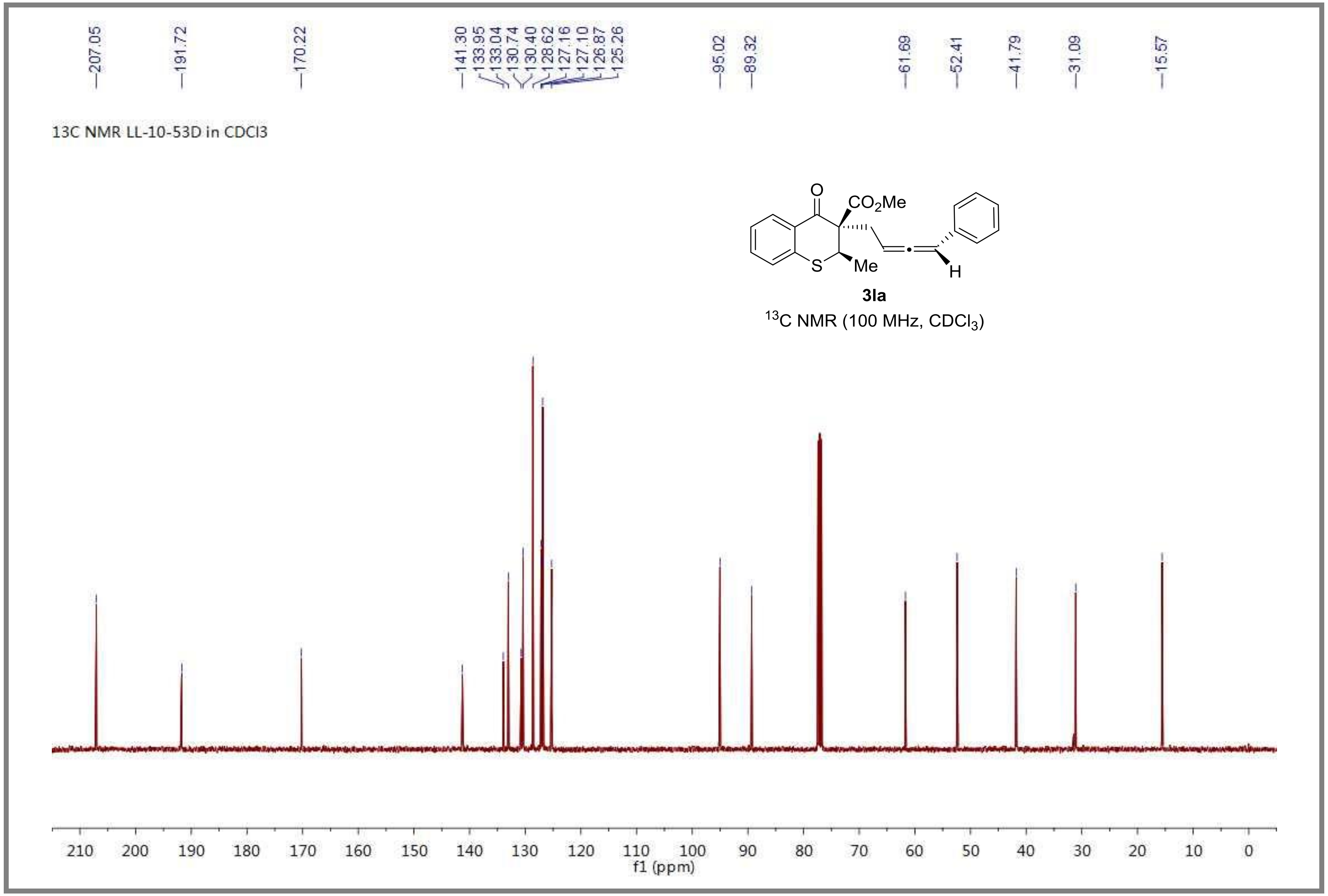

207.05
191.72
170.22
141.30
133.95
133.04
130.74
130.40
128.62
127.16
127.10
126.87
125.26
95.02
89.32
61.69
52.41
41.79
31.09
15.57
13C NMR LL-10-53D in CDCl3
O
CO2Me
S
Me
H
3la
13C NMR (100 MHz, CDCl3)
210 200 190 180 170 160 150 140 130 120 110 100 90 80 70 60 50 40 30 20 10 0
f1 (ppm)

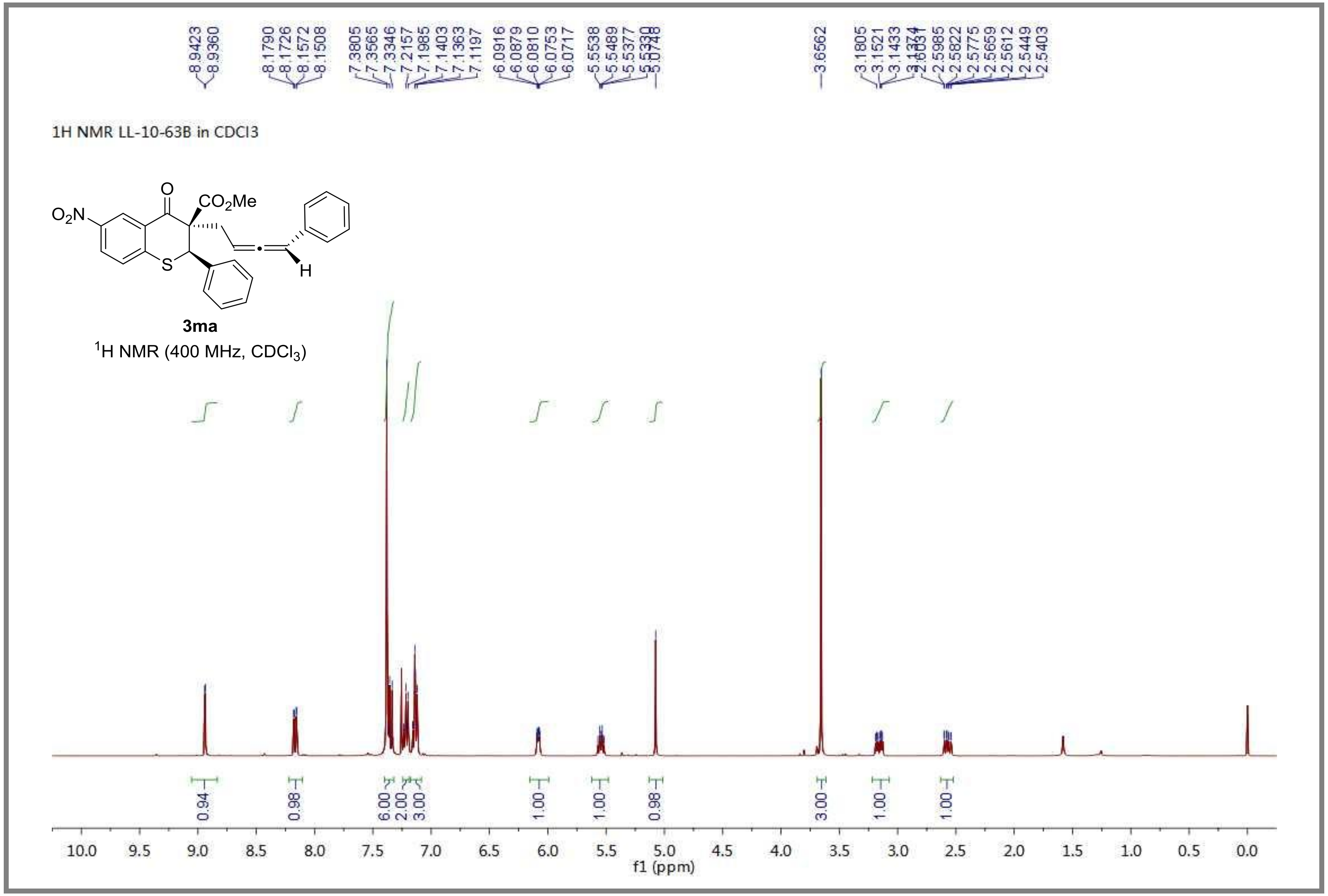
8.9423
8.9360
8.1790
8.1726
8.1572
8.1508
7.3805
7.3565
7.3346
7.2157
7.1985
7.1403
7.1363
7.1197
6.0916
6.0879
6.0810
6.0753
6.0717
5.5538
5.5489
5.5377
5.0748
3.6562
3.1805
3.1521
3.1433
2.5985
2.5822
2.5775
2.5659
2.5612
2.5449
2.5403
1H NMR LL-10-63B in CDCl3
O
O_2N
CO_2Me
S
H
3ma
^1H NMR (400 MHz, $CDCl_3$)
0.94
0.98
6.00
2.00
3.00
1.00
1.00
0.98
3.00
1.00
1.00
10.0 9.5 9.0 8.5 8.0 7.5 7.0 6.5 6.0 5.5 5.0 4.5 4.0 3.5 3.0 2.5 2.0 1.5 1.0 0.5 0.0
f1 (ppm)

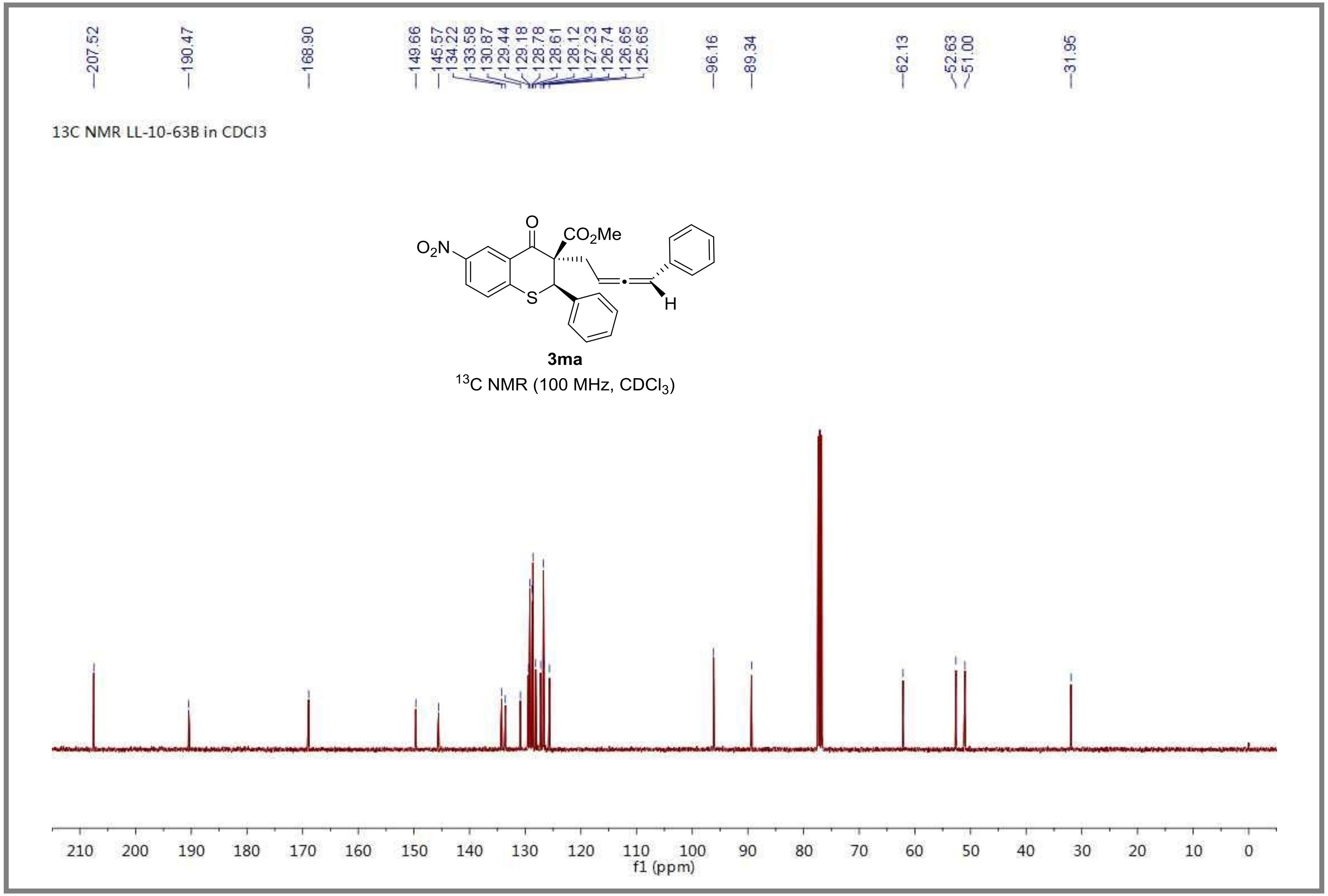

207.52
190.47
168.90
149.66
145.57
134.22
133.58
130.87
129.44
129.18
128.78
128.61
128.12
127.23
126.74
126.65
125.65
96.16
89.34
62.13
52.63
51.00
31.95
13C NMR LL-10-63B in CDCl3
O
CO2Me
O2N
S
H
3ma
13C NMR (100 MHz, CDCl3)
210 200 190 180 170 160 150 140 130 120 110 100 90 80 70 60 50 40 30 20 10 0
f1 (ppm)

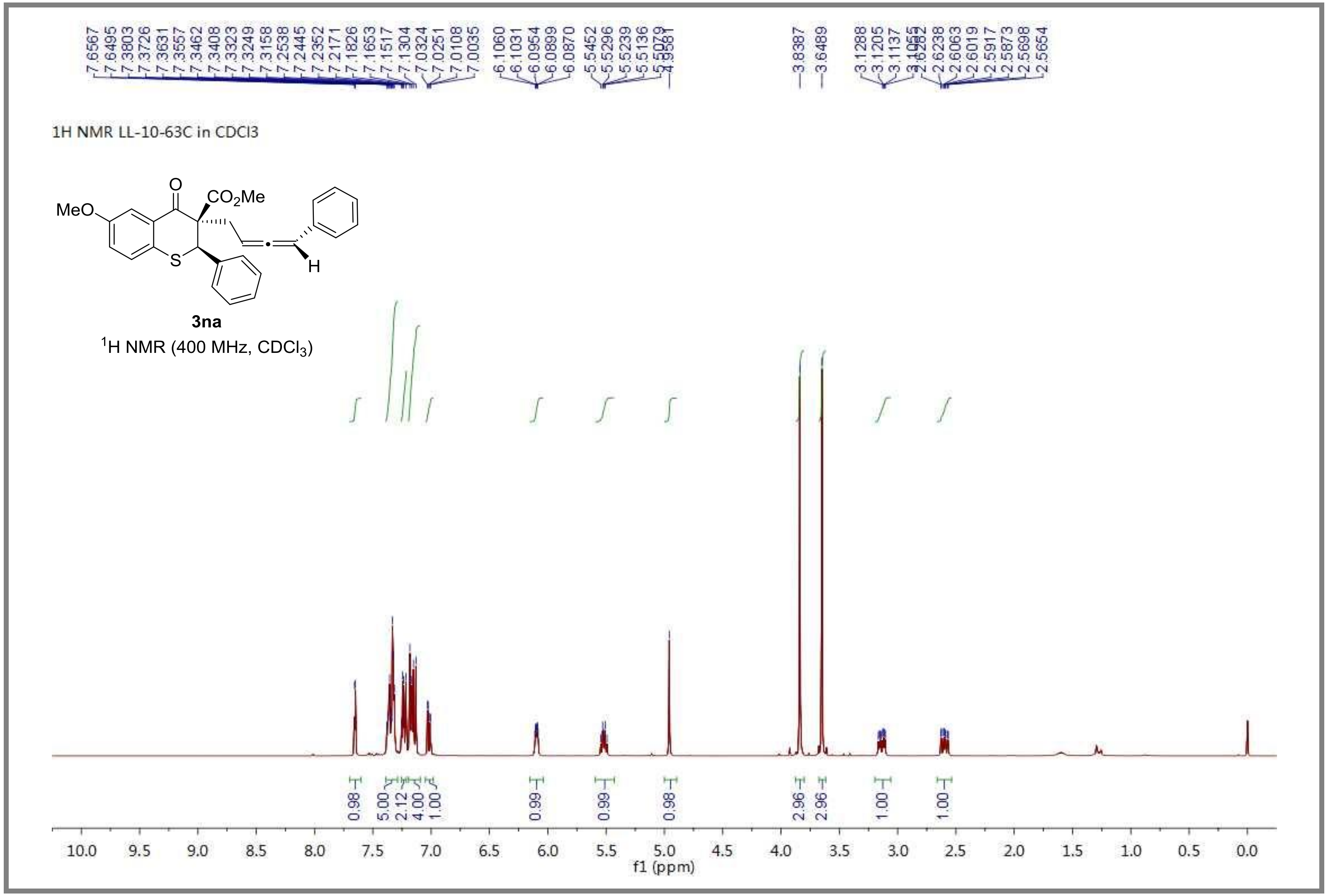

1H NMR LL-10-63C in CDCl3
MeO
O
CO2Me
S
H
3na
1H NMR (400 MHz, CDCl3)
f1 (ppm)

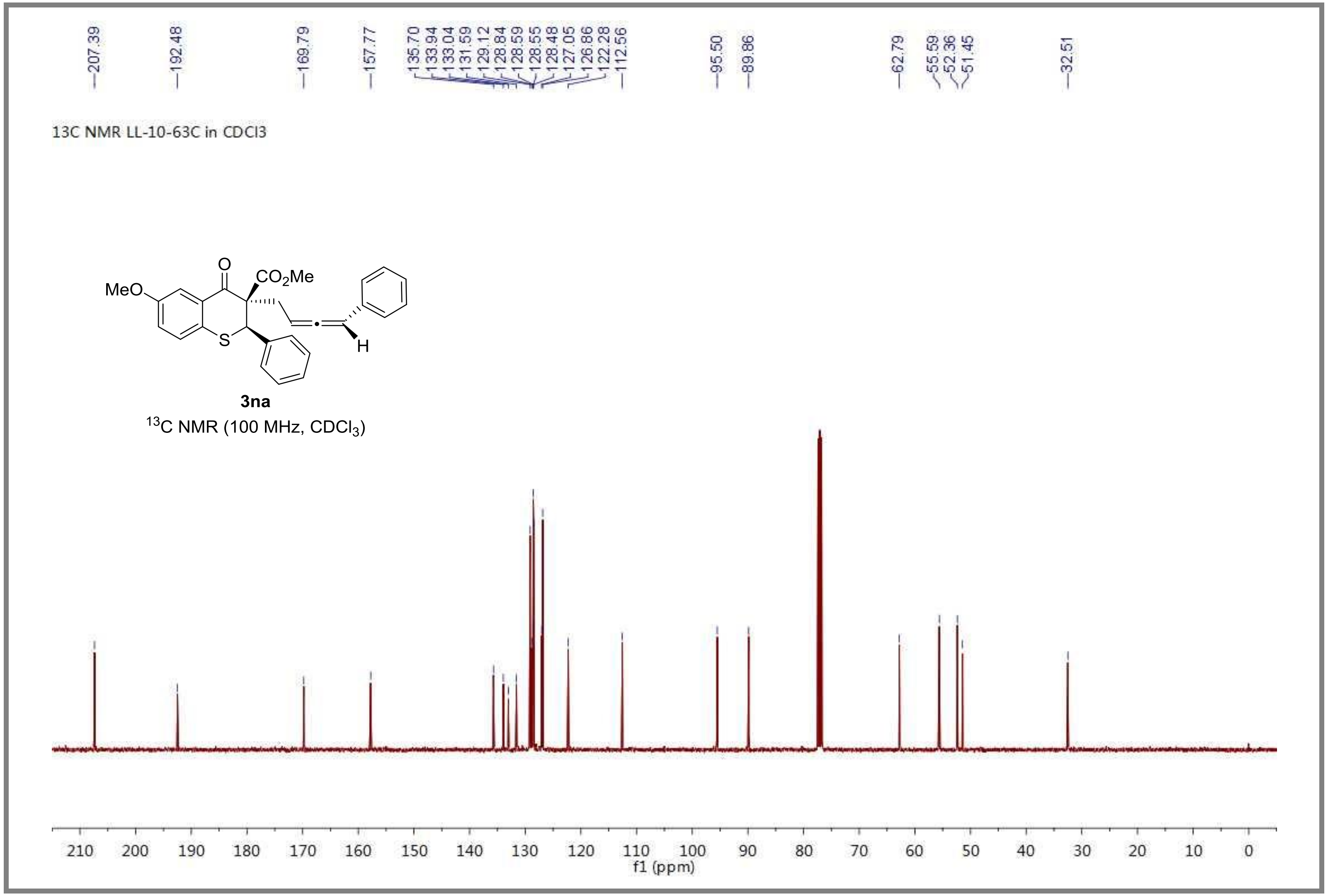

3na

^{13}C NMR (100 MHz, $CDCl_3$)

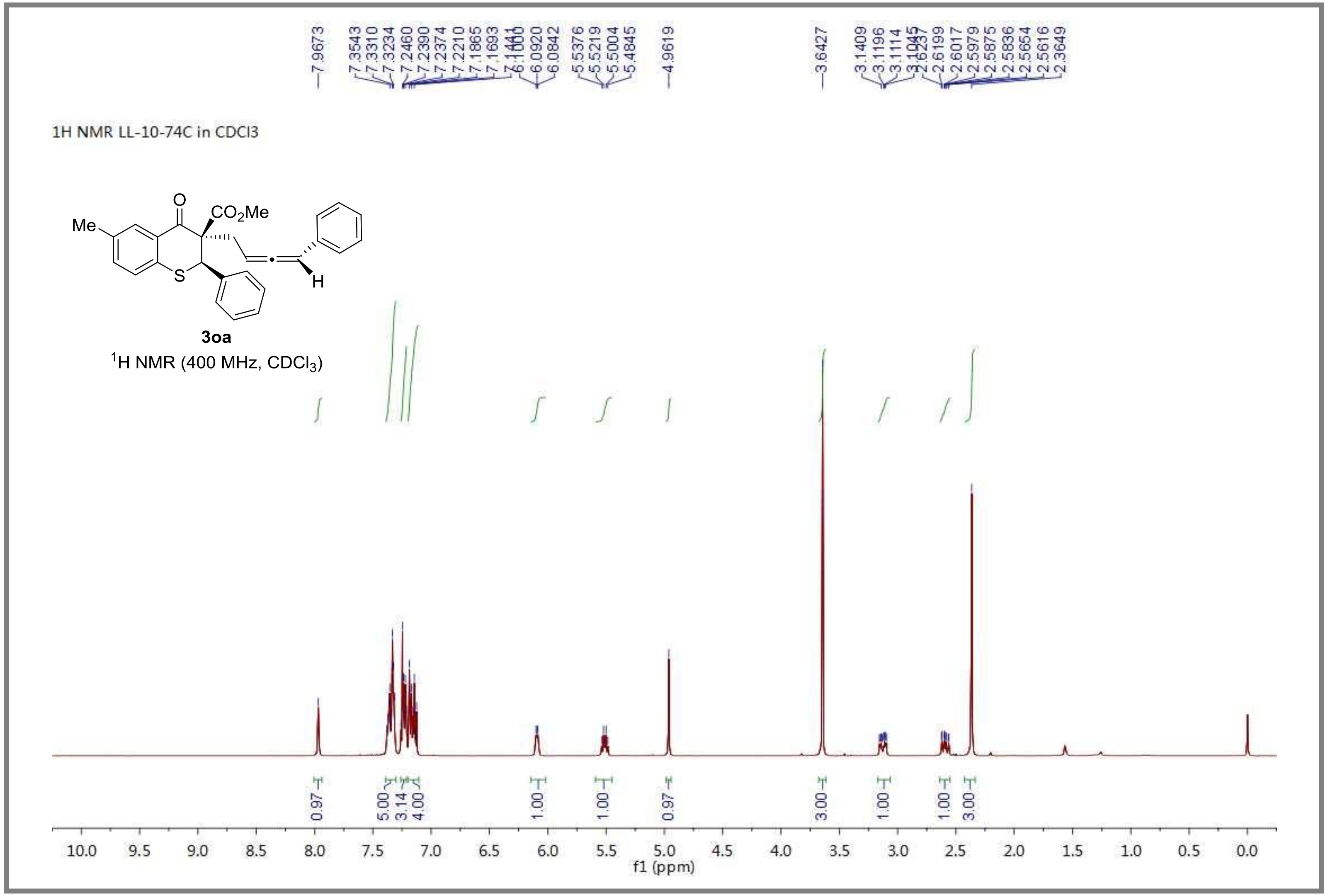
1H NMR LL-10-74C in CDCl3
3oa
^{1}H NMR (400 MHz, $CDCl_3$)
f1 (ppm)

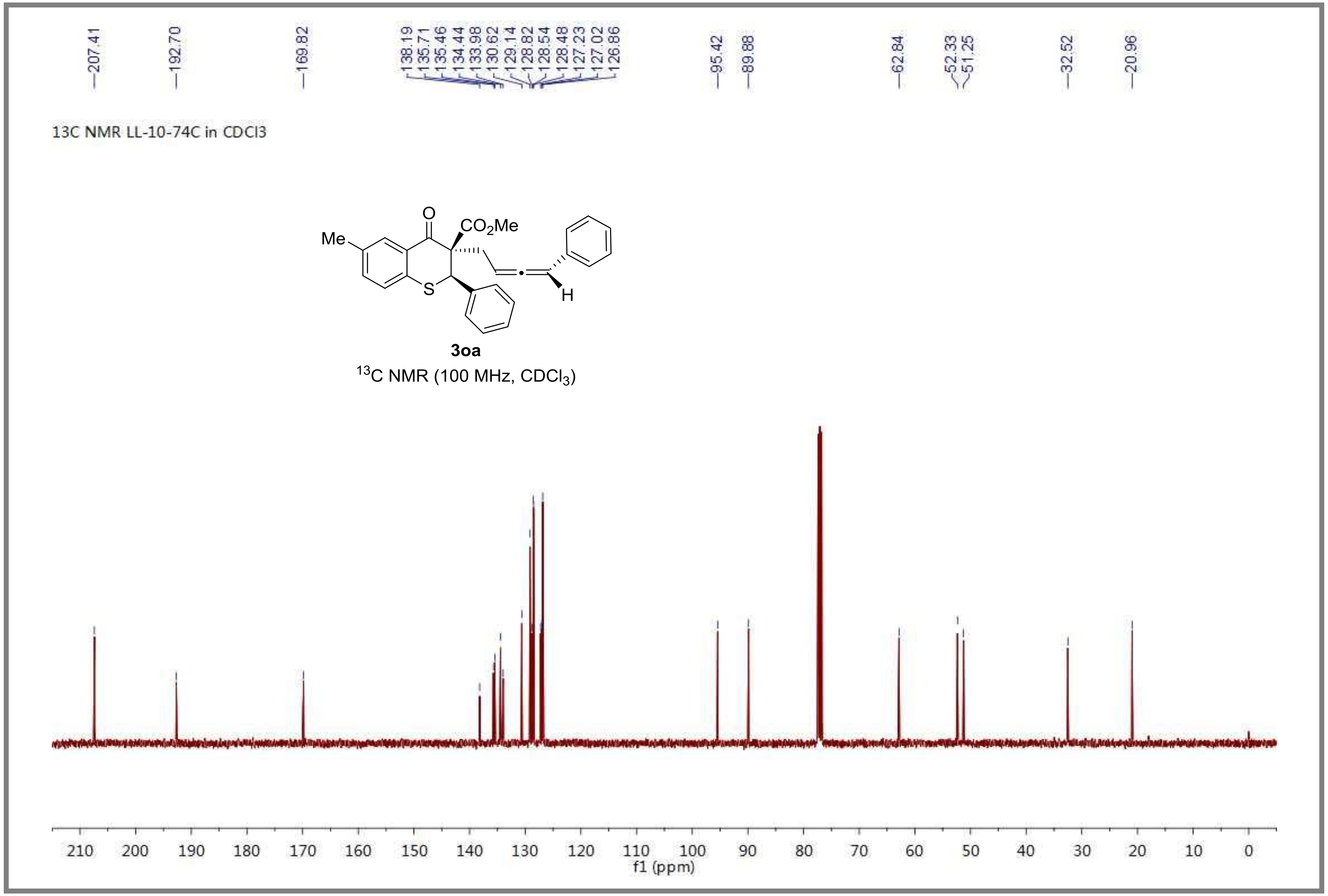

207.41
192.70
169.82
138.19
135.71
135.46
134.44
133.98
130.62
129.14
128.82
128.54
128.48
127.23
127.02
126.86
95.42
89.88
62.84
52.33
51.25
32.52
20.96
13C NMR LL-10-74C in CDCl3
Me
O
CO2Me
S
H
3oa
13C NMR (100 MHz, CDCl3)
210 200 190 180 170 160 150 140 130 120 110 100 90 80 70 60 50 40 30 20 10 0
f1 (ppm)

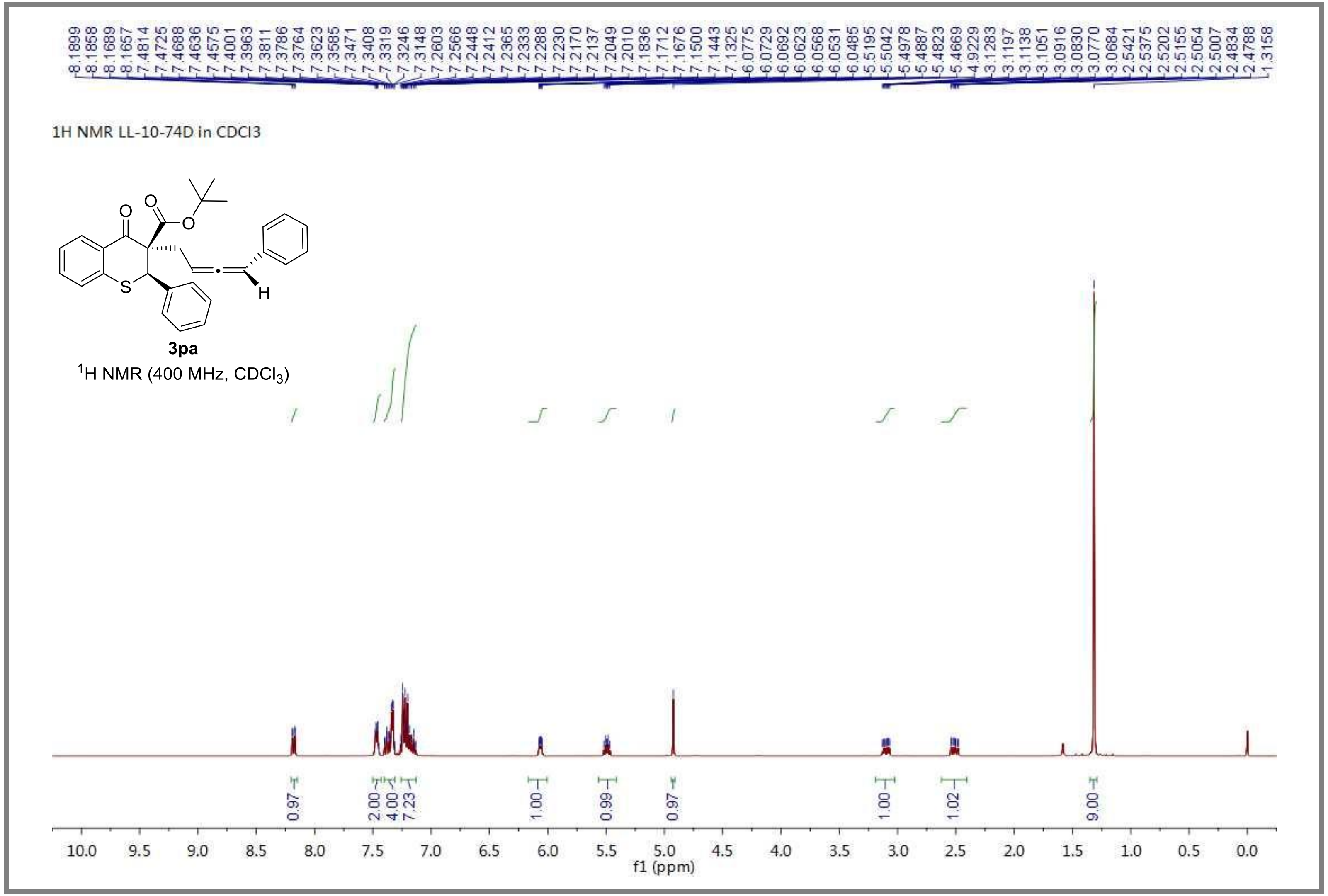
1H NMR LL-10-74D in CDCl3
3pa
1H NMR (400 MHz, CDCl3)
f1 (ppm)

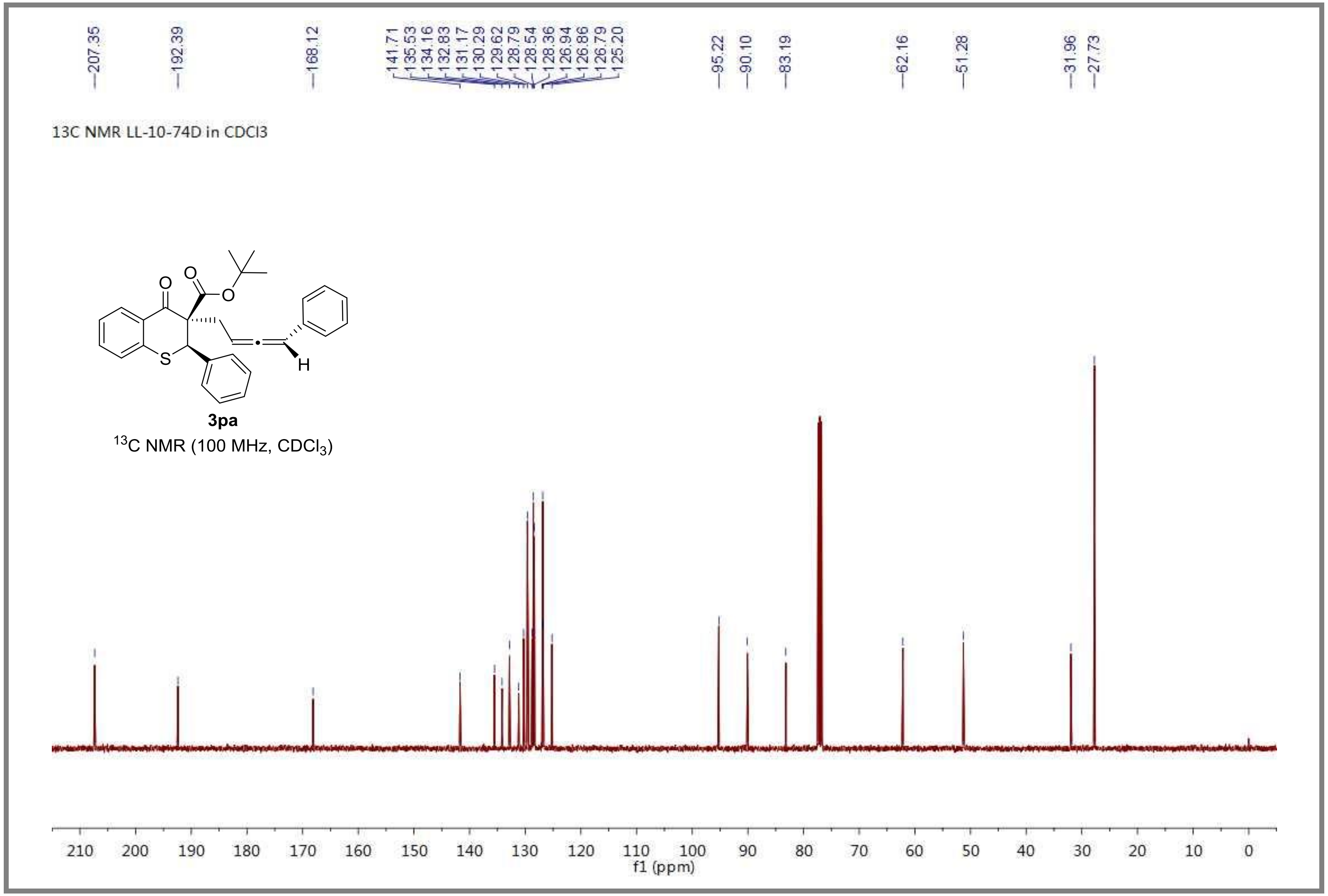
207.35
192.39
168.12
141.71
135.53
134.16
132.83
131.17
130.29
129.62
128.79
128.54
128.36
126.94
126.86
126.79
125.20
95.22
90.10
83.19
62.16
51.28
31.96
27.73
13C NMR LL-10-74D in CDCl3
O
O
O
S
H
3pa
^{13}C NMR (100 MHz, $CDCl_3$)
210 200 190 180 170 160 150 140 130 120 110 100 90 80 70 60 50 40 30 20 10 0
f1 (ppm)

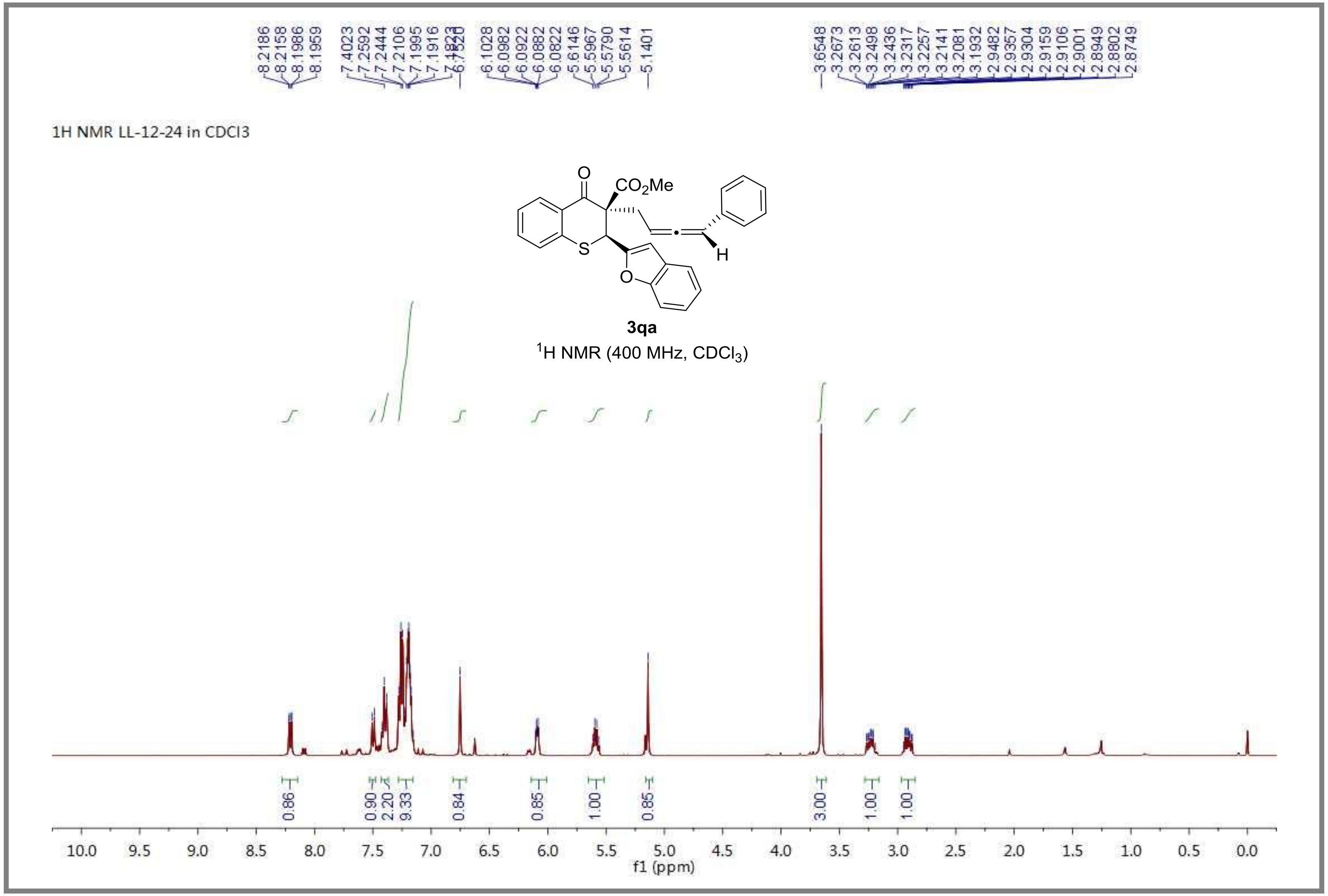
1H NMR LL-12-24 in CDCl3
3qa
^{1}H NMR (400 MHz, $CDCl_3$)
f1 (ppm)

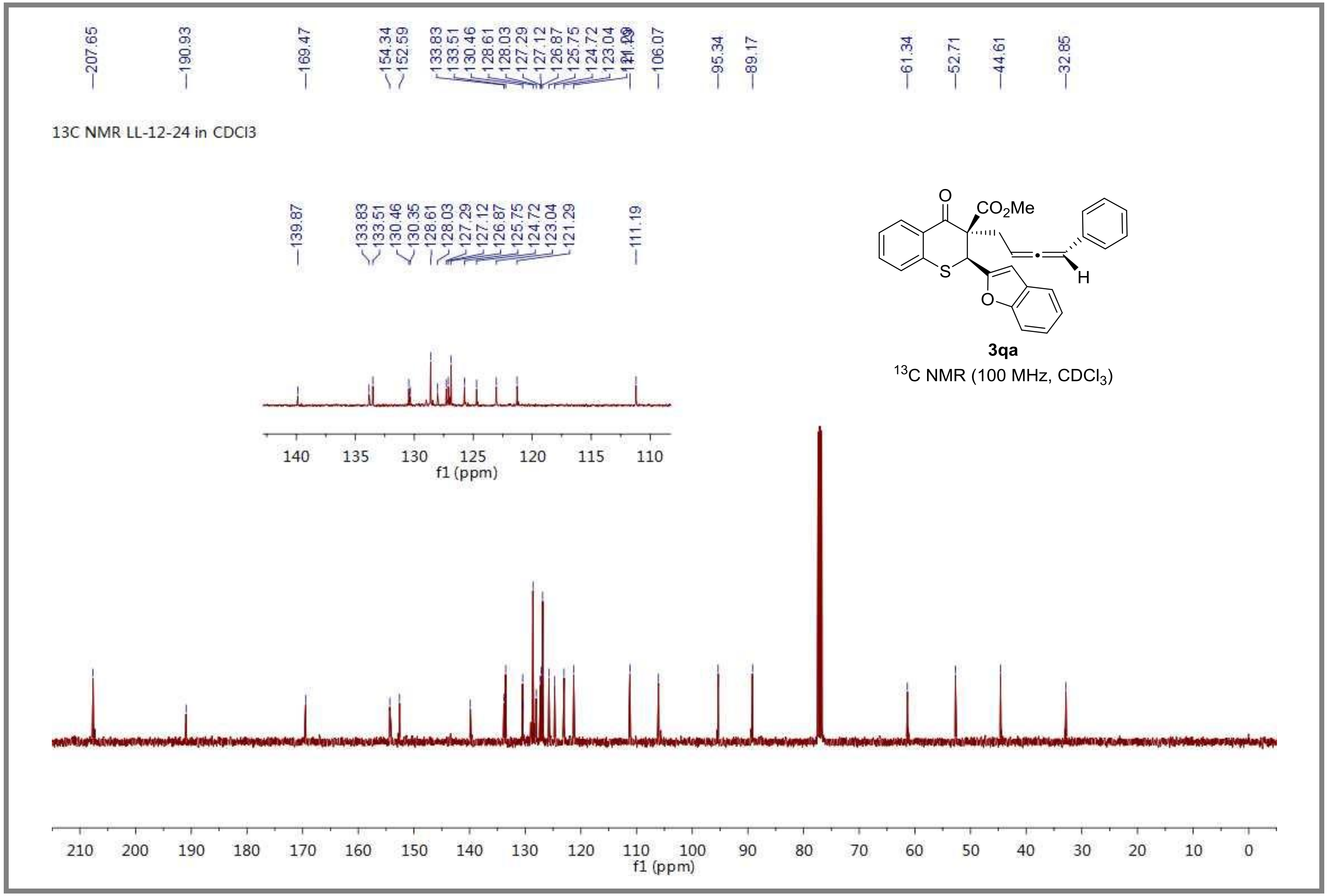
13C NMR LL-12-24 in CDCl3
207.65
190.93
169.47
154.34
152.59
133.83
133.51
130.46
128.61
128.03
127.29
127.12
126.87
125.75
124.72
123.04
106.07
95.34
89.17
61.34
52.71
44.61
32.85
139.87
130.35
121.29
111.19
f1 (ppm)
CO2Me
S
O
H
3qa
13C NMR (100 MHz, CDCl3)

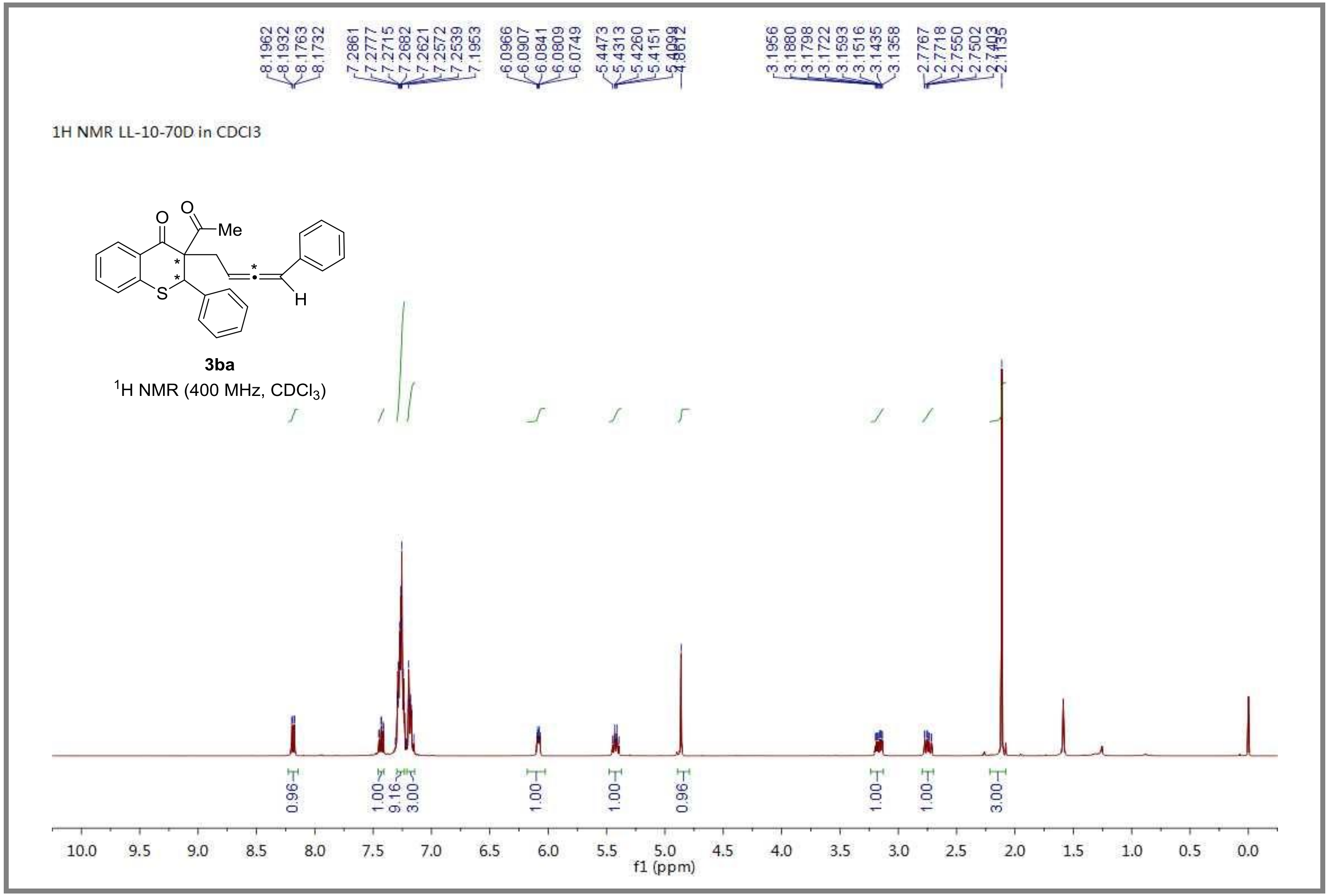
8.1962
8.1932
8.1763
8.1732
7.2861
7.2777
7.2715
7.2682
7.2621
7.2572
7.2539
7.1953
6.0966
6.0907
6.0841
6.0809
6.0749
5.4473
5.4313
5.4260
5.4151
3.1956
3.1880
3.1798
3.1722
3.1593
3.1516
3.1435
3.1358
2.7767
2.7718
2.7550
2.7502
2.1135
1H NMR LL-10-70D in CDCl3
O
O
Me
S
H
3ba
1H NMR (400 MHz, CDCl3)
0.96
1.00
9.16
3.00
1.00
1.00
0.96
1.00
1.00
3.00
10.0 9.5 9.0 8.5 8.0 7.5 7.0 6.5 6.0 5.5 5.0 4.5 4.0 3.5 3.0 2.5 2.0 1.5 1.0 0.5 0.0
f1 (ppm)

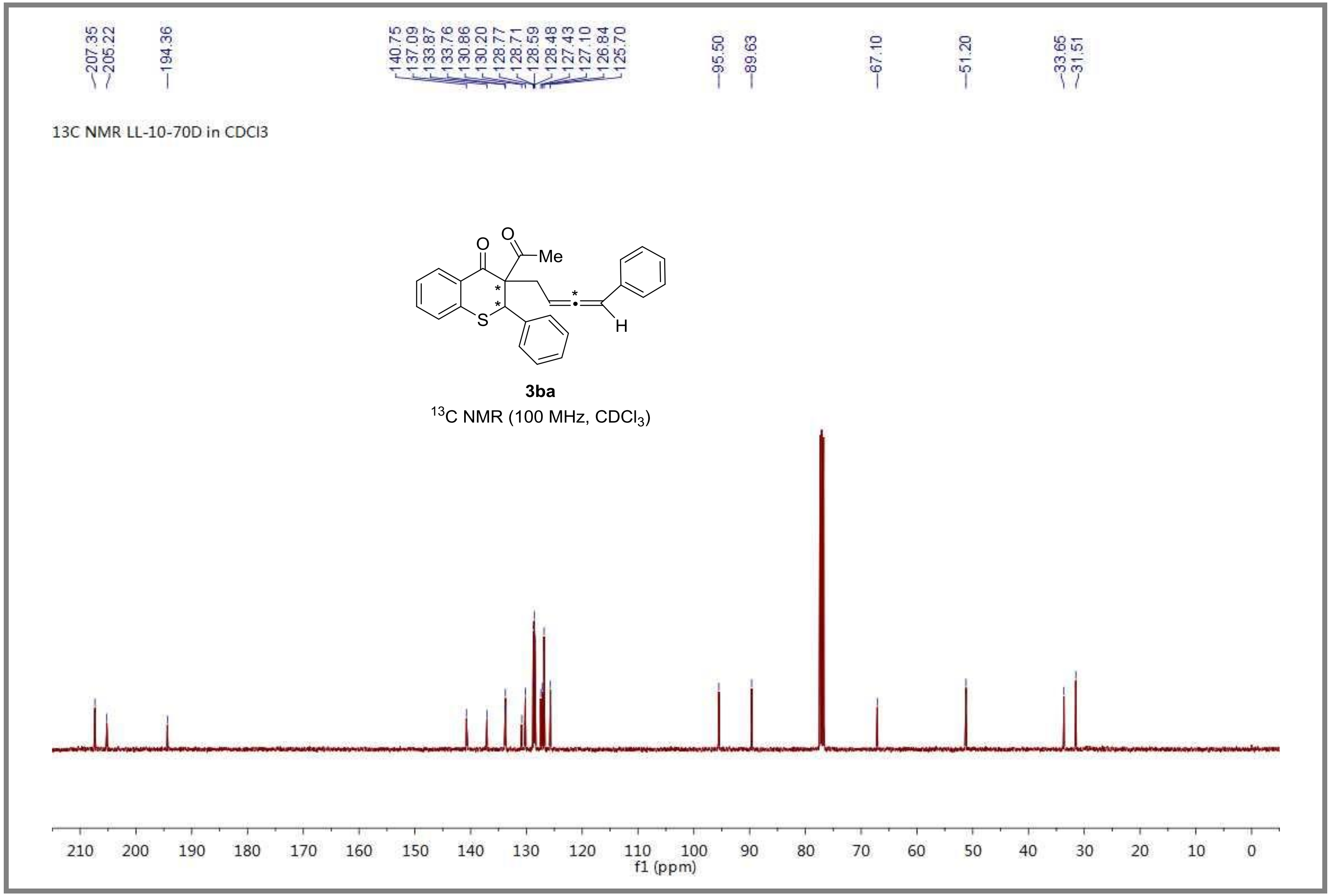

207.35
205.22
194.36
140.75
137.09
133.87
133.76
130.86
130.20
128.77
128.71
128.59
128.48
127.43
127.10
126.84
125.70
95.50
89.63
67.10
51.20
33.65
31.51
13C NMR LL-10-70D in CDCl3
O
O
Me
S
H
3ba
13C NMR (100 MHz, CDCl3)
210 200 190 180 170 160 150 140 130 120 110 100 90 80 70 60 50 40 30 20 10 0
f1 (ppm)

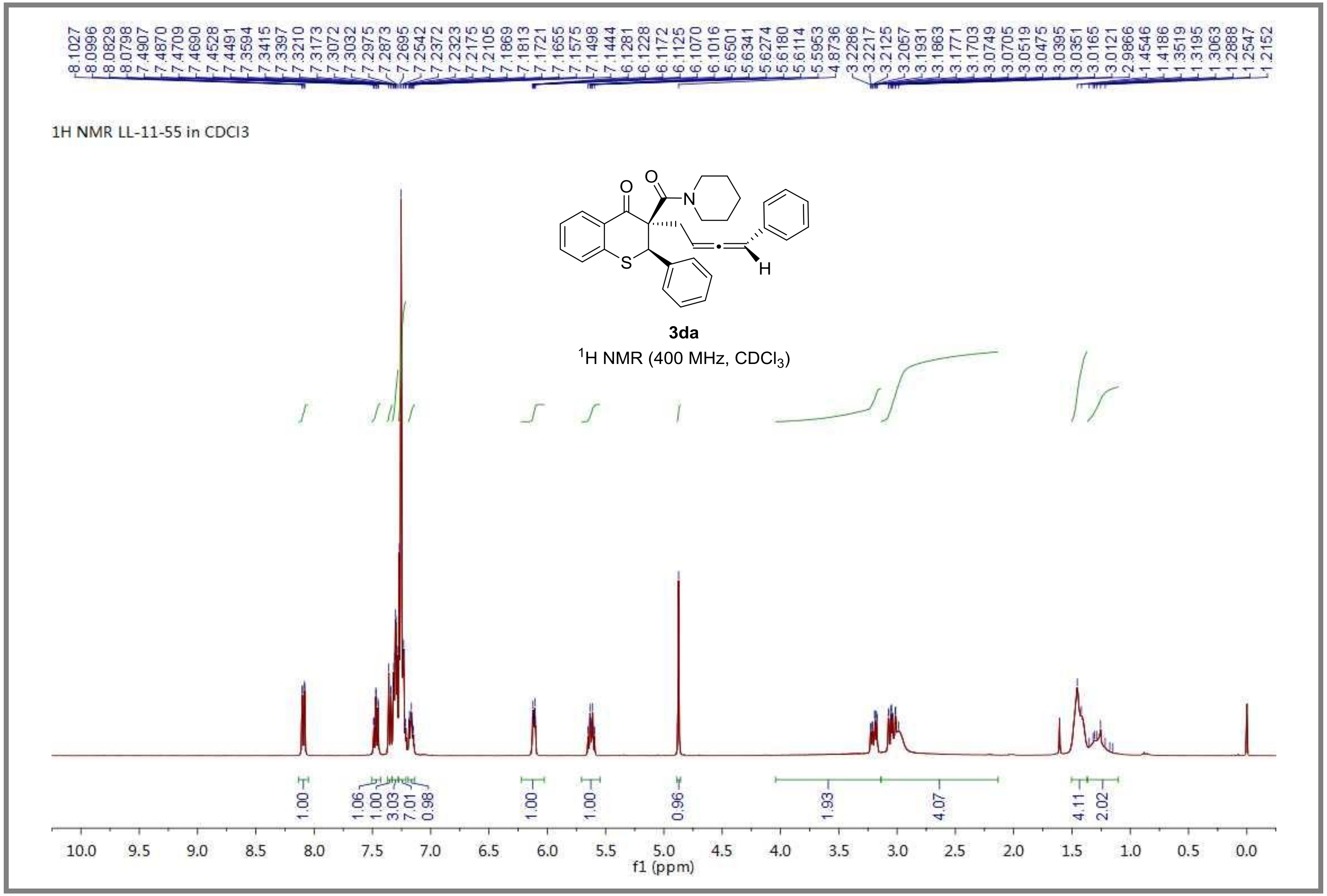
1H NMR LL-11-55 in CDCl3
3da
¹H NMR (400 MHz, CDCl₃)
f1 (ppm)

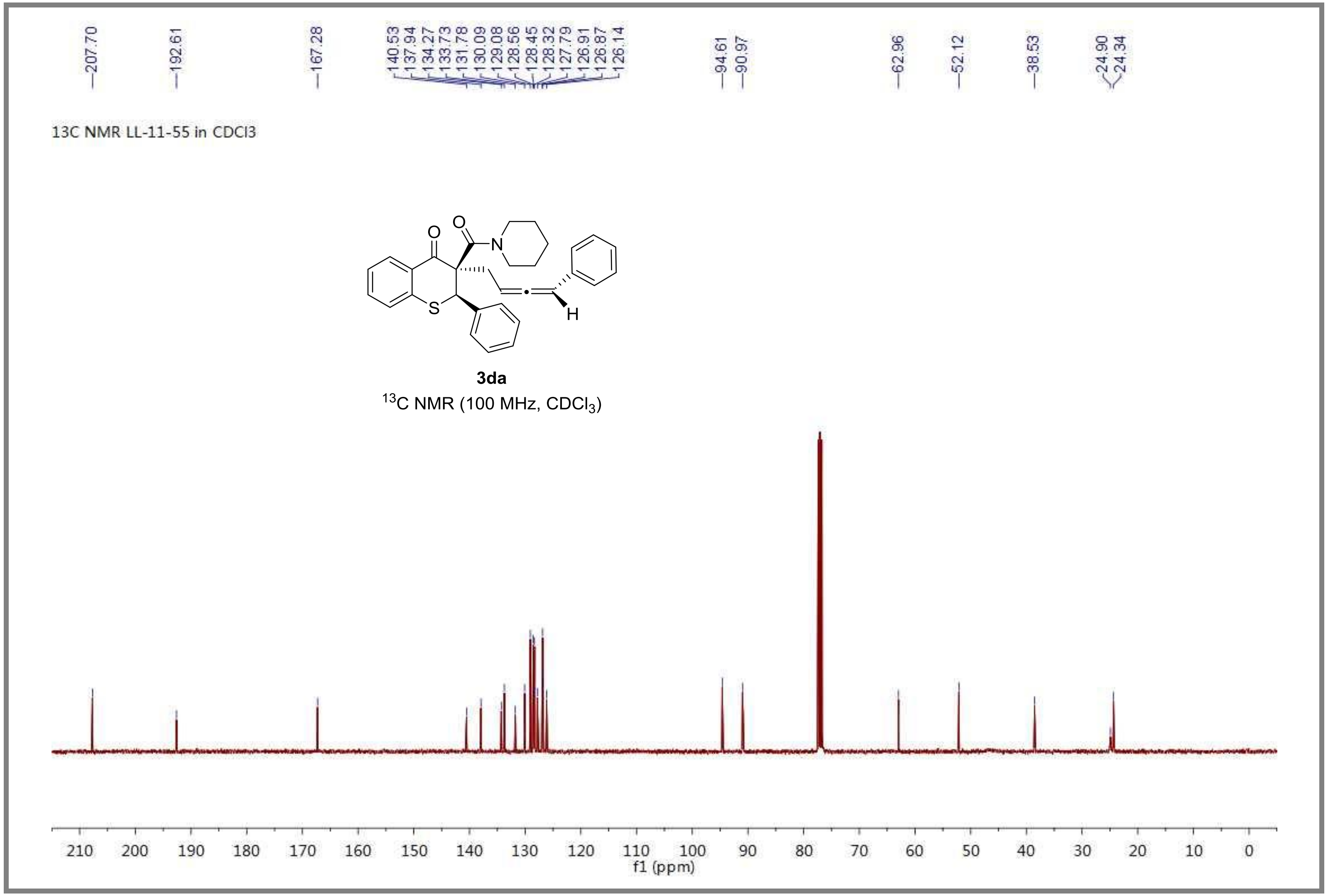
207.70
192.61
167.28
140.53
137.94
134.27
133.73
131.78
130.09
129.08
128.56
128.45
128.32
127.79
126.91
126.87
126.14
94.61
90.97
62.96
52.12
38.53
24.90
24.34
13C NMR LL-11-55 in CDCl3
O
O
N
S
H
3da
^{13}C NMR (100 MHz, $CDCl_3$)
210 200 190 180 170 160 150 140 130 120 110 100 90 80 70 60 50 40 30 20 10 0
f1 (ppm)

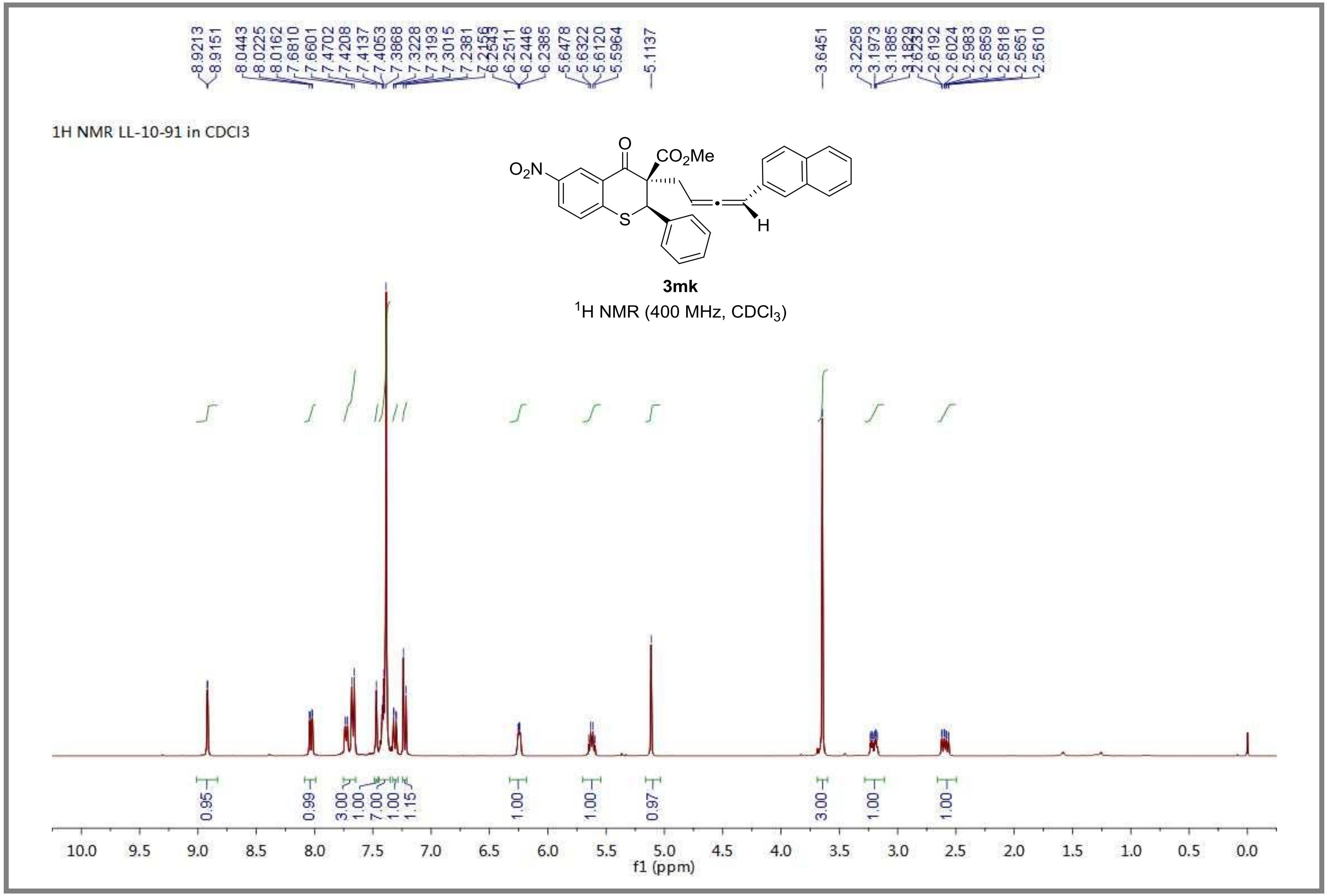
1H NMR LL-10-91 in CDCl3
O
CO2Me
O2N
S
H
3mk
1H NMR (400 MHz, CDCl3)
f1 (ppm)

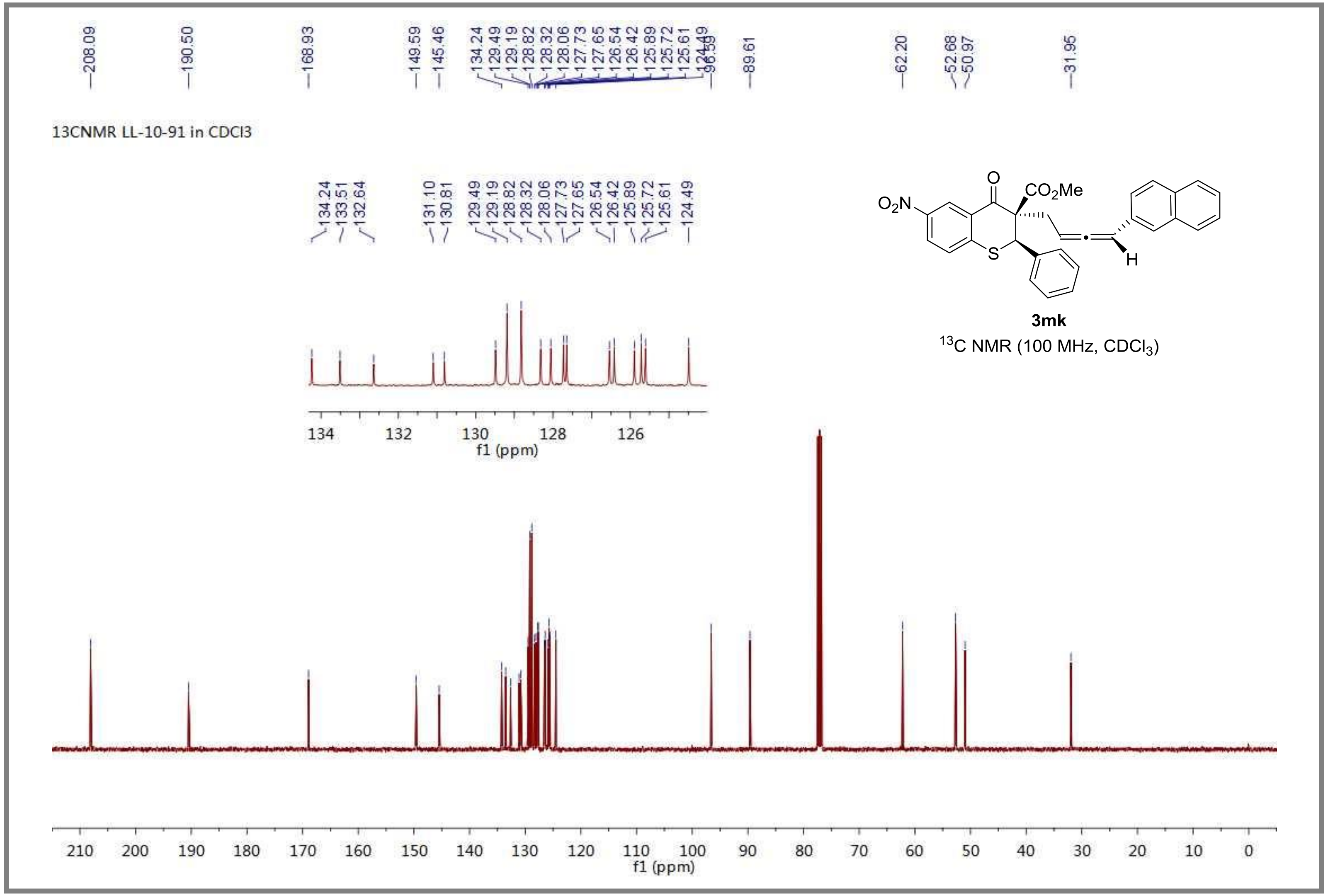
13CNMR LL-10-91 in CDCl3
208.09
190.50
168.93
149.59
145.46
134.24
129.49
129.19
128.82
128.32
128.06
127.73
127.65
126.54
126.42
125.89
125.72
125.61
124.49
96.59
89.61
62.20
52.68
50.97
31.95
134.24
133.51
132.64
131.10
130.81
129.49
129.19
128.82
128.32
128.06
127.73
127.65
126.54
126.42
125.89
125.72
125.61
124.49
O2N
O
CO2Me
S
H
3mk
13C NMR (100 MHz, CDCl3)
f1 (ppm)

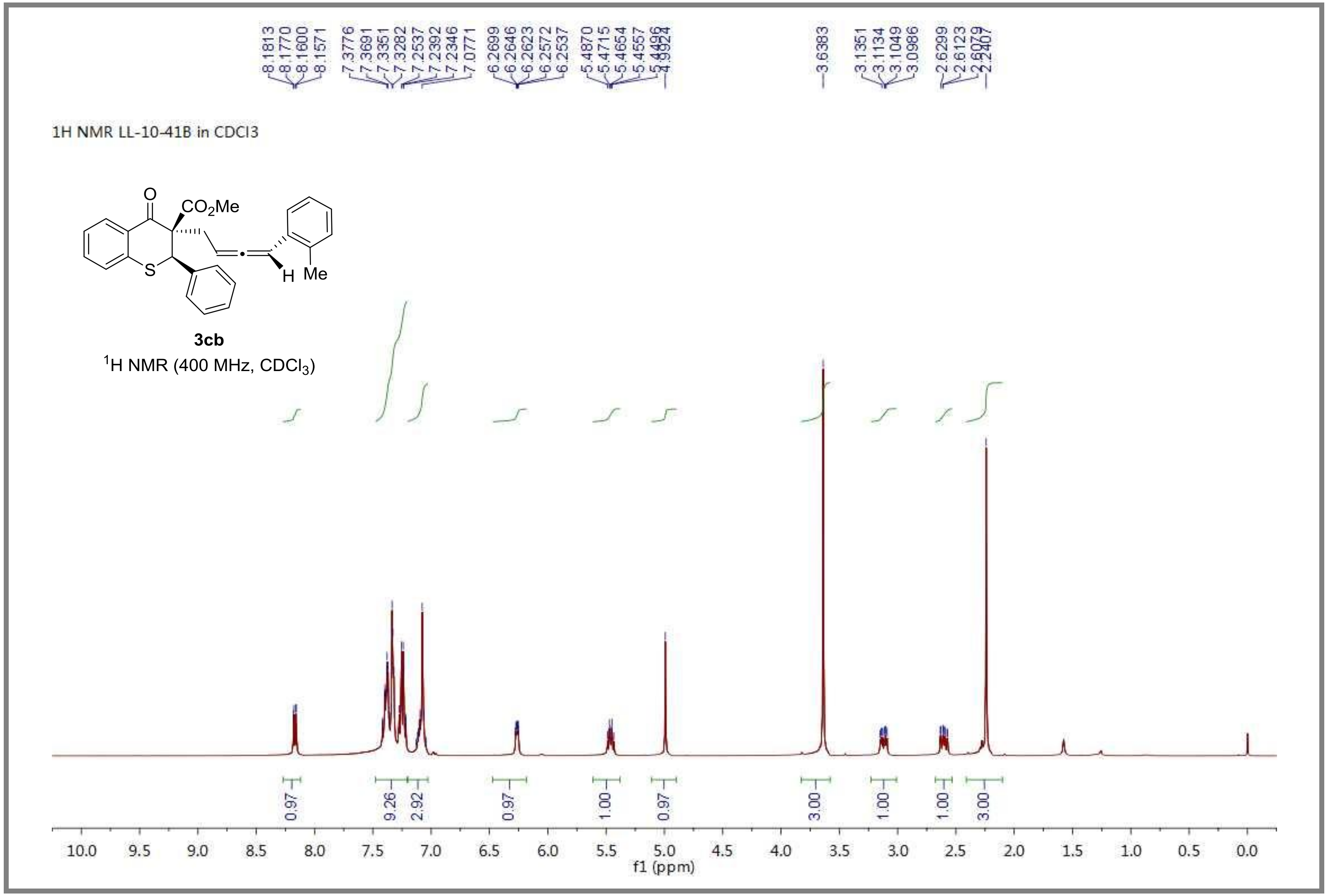

1H NMR LL-10-41B in CDCl3
3cb
¹H NMR (400 MHz, CDCl3)
8.1813
8.1770
8.1600
8.1571
7.3776
7.3691
7.3351
7.3282
7.2537
7.2392
7.2346
7.0771
6.2699
6.2646
6.2623
6.2572
6.2537
5.4870
5.4715
5.4654
5.4557
5.4496
4.9924
3.6383
3.1351
3.1134
3.1049
3.0986
2.6299
2.6123
2.6079
2.2407
0.97
9.26
2.92
0.97
1.00
0.97
3.00
1.00
1.00
3.00
f1 (ppm)

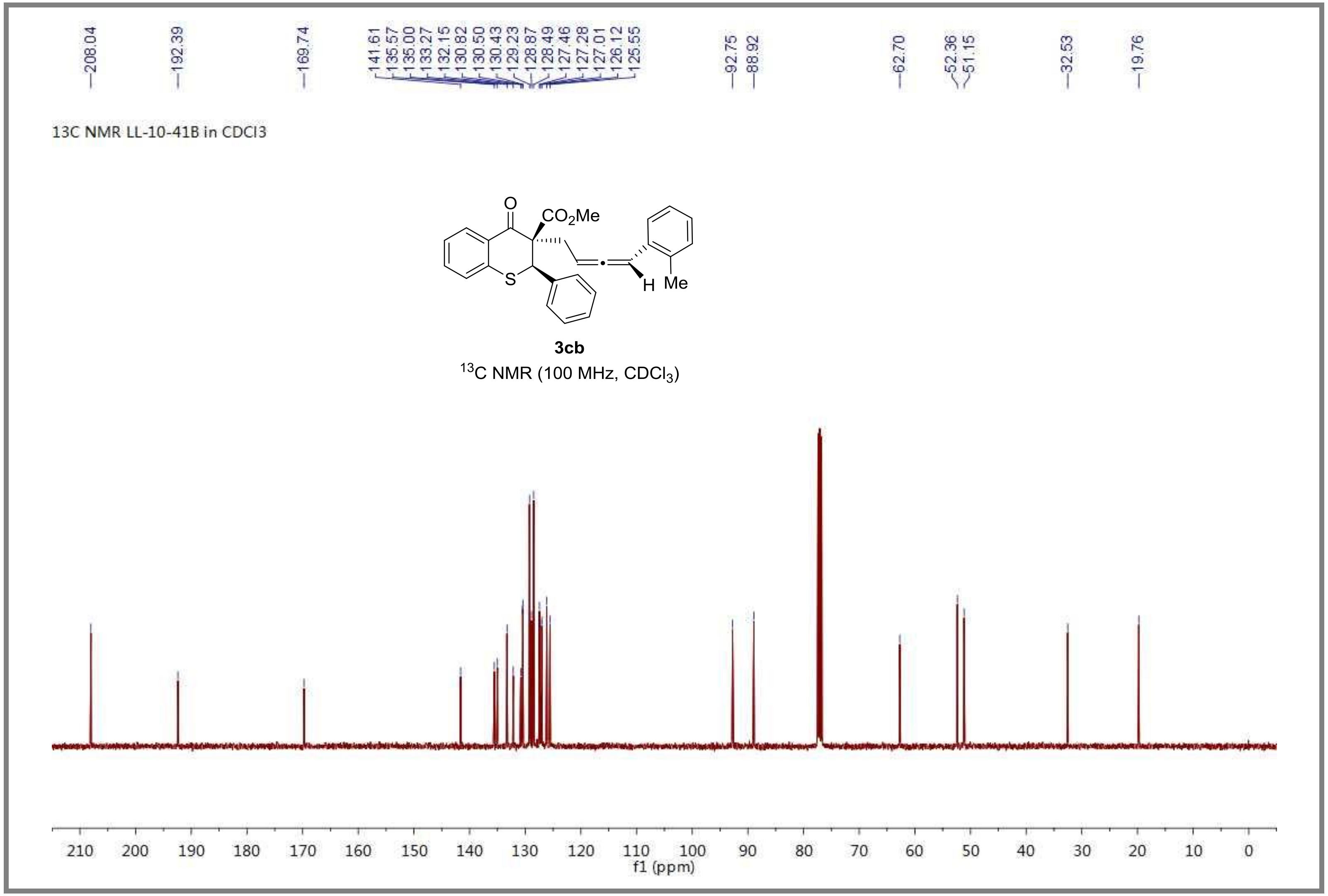
208.04
192.39
169.74
141.61
135.57
135.00
133.27
132.15
130.82
130.50
130.43
129.23
128.87
128.49
127.46
127.28
127.01
126.12
125.55
92.75
88.92
62.70
52.36
51.15
32.53
19.76
13C NMR LL-10-41B in CDCl3
O
CO2Me
S
H
Me
3cb
13C NMR (100 MHz, CDCl3)
210
200
190
180
170
160
150
140
130
120
110
100
90
80
70
60
50
40
30
20
10
0
f1 (ppm)

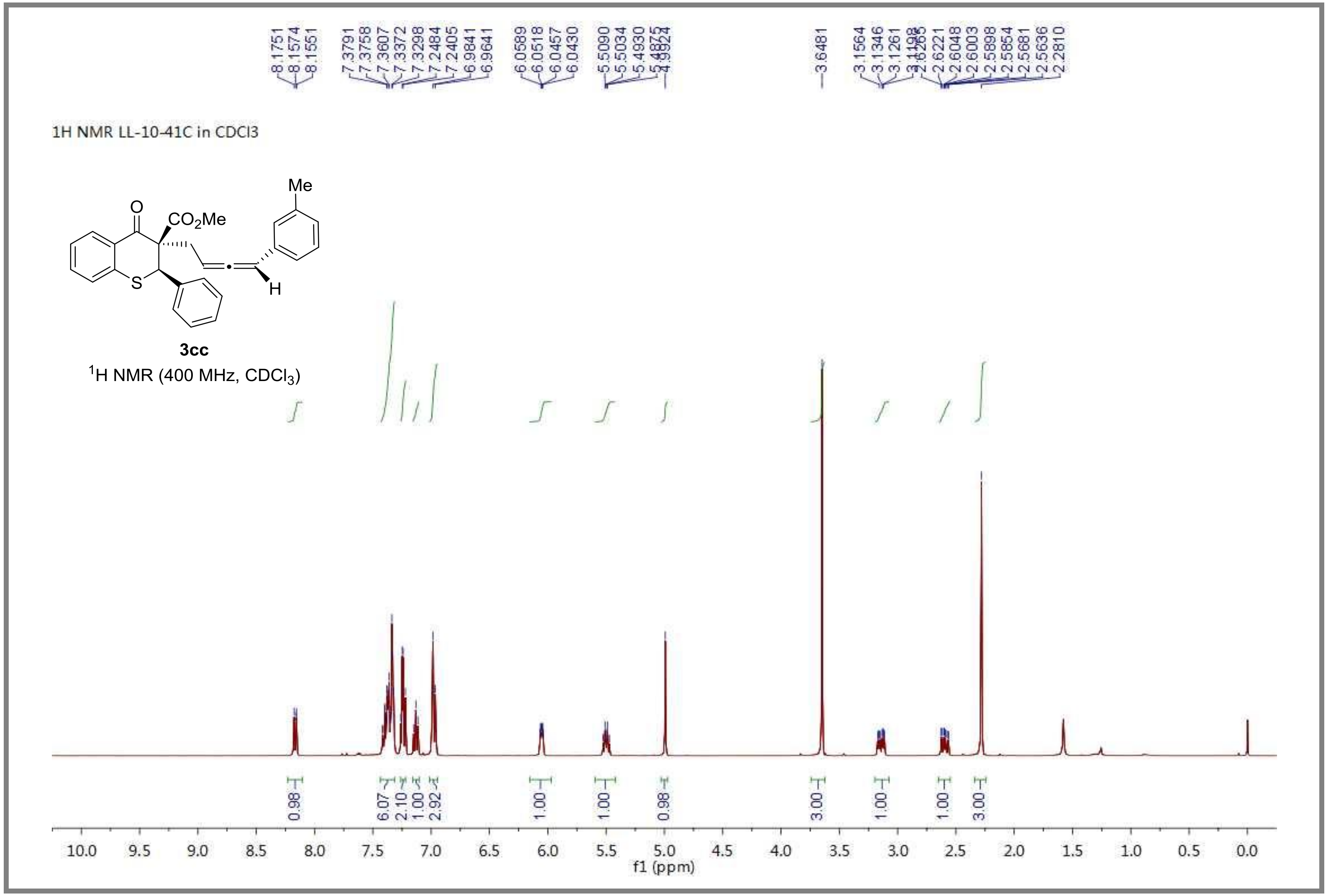
1H NMR LL-10-41C in CDCl3
Me
O
CO2Me
S
H
3cc
1H NMR (400 MHz, CDCl3)
8.1751
8.1574
8.1551
7.3791
7.3758
7.3607
7.3372
7.3298
7.2484
7.2405
6.9841
6.9641
6.0589
6.0518
6.0457
6.0430
5.5090
5.5034
5.4930
3.6481
3.1564
3.1346
3.1261
2.6221
2.6048
2.6003
2.5898
2.5854
2.5681
2.5636
2.2810
0.98
6.07
2.10
1.00
2.92
1.00
1.00
0.98
3.00
1.00
1.00
3.00
10.0
9.5
9.0
8.5
8.0
7.5
7.0
6.5
6.0
5.5
5.0
4.5
4.0
3.5
3.0
2.5
2.0
1.5
1.0
0.5
0.0
f1 (ppm)

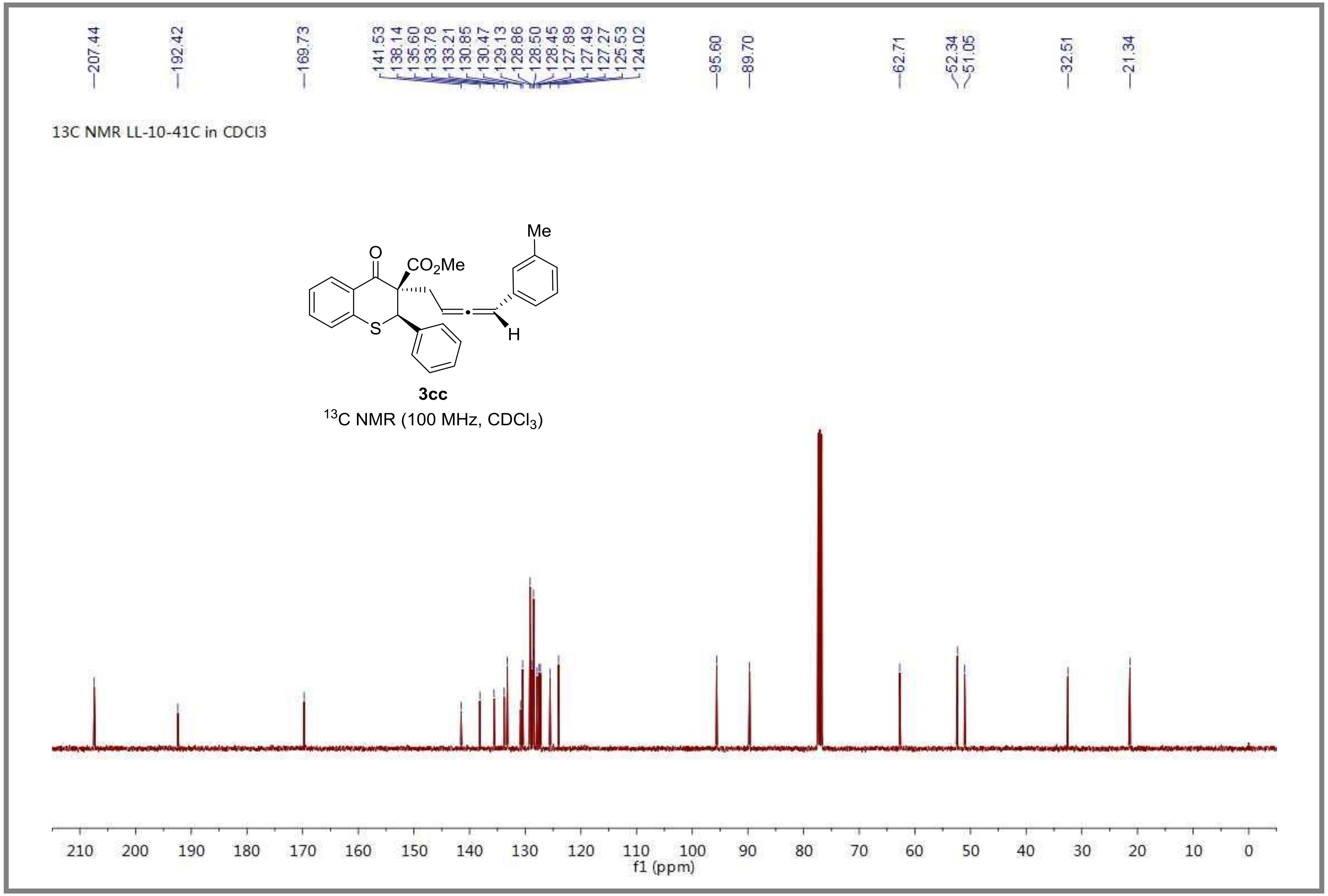
207.44
192.42
169.73
141.53
138.14
135.60
133.78
133.21
130.85
130.47
129.13
128.86
128.50
128.45
127.89
127.49
127.27
125.53
124.02
95.60
89.70
62.71
52.34
51.05
32.51
21.34
13C NMR LL-10-41C in CDCl3
Me
O
CO_2Me
S
H
3cc
^{13}C NMR (100 MHz, $CDCl_3$)
210 200 190 180 170 160 150 140 130 120 110 100 90 80 70 60 50 40 30 20 10 0
f1 (ppm)

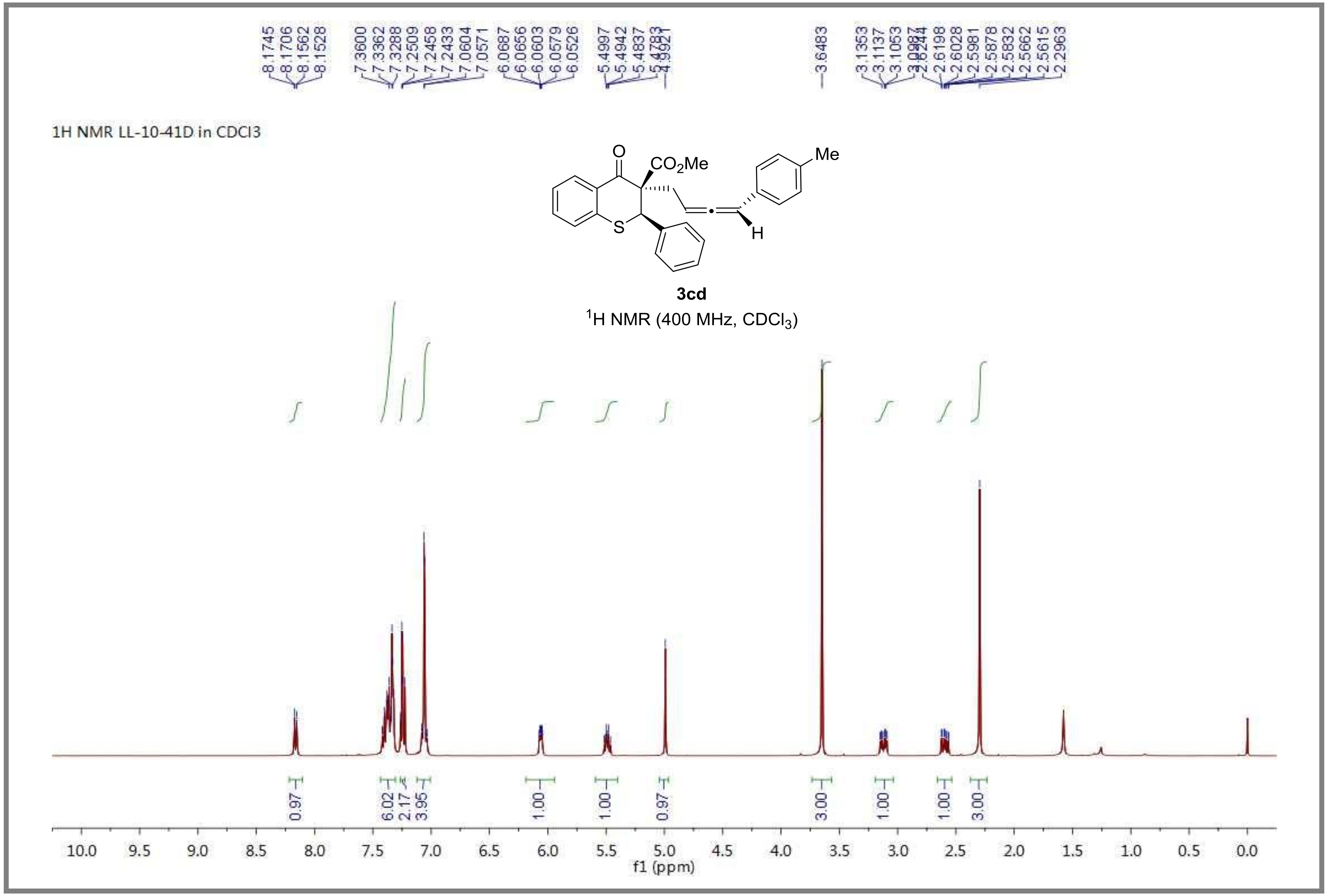
1H NMR LL-10-41D in CDCl3
CO2Me
Me
O
S
H
3cd
1H NMR (400 MHz, CDCl3)
f1 (ppm)

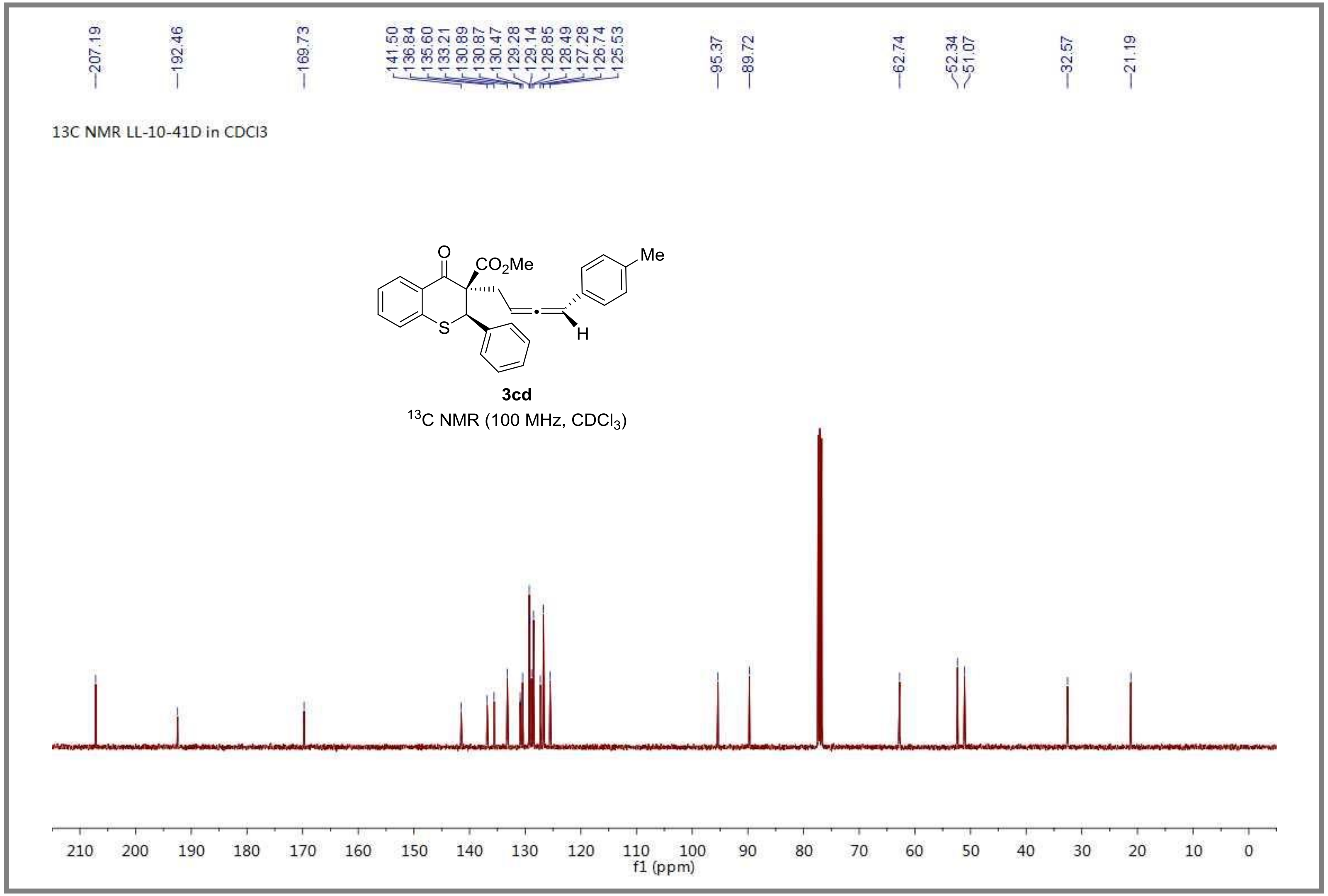

207.19
192.46
169.73
141.50
136.84
135.60
133.21
130.89
130.87
130.47
129.28
129.14
128.85
128.49
127.28
126.74
125.53
95.37
89.72
62.74
52.34
51.07
32.57
21.19
13C NMR LL-10-41D in CDCl3
O
CO_2Me
Me
S
H
3cd
^{13}C NMR (100 MHz, $CDCl_3$)
210
200
190
180
170
160
150
140
130
120
110
100
90
80
70
60
50
40
30
20
10
0
f1 (ppm)

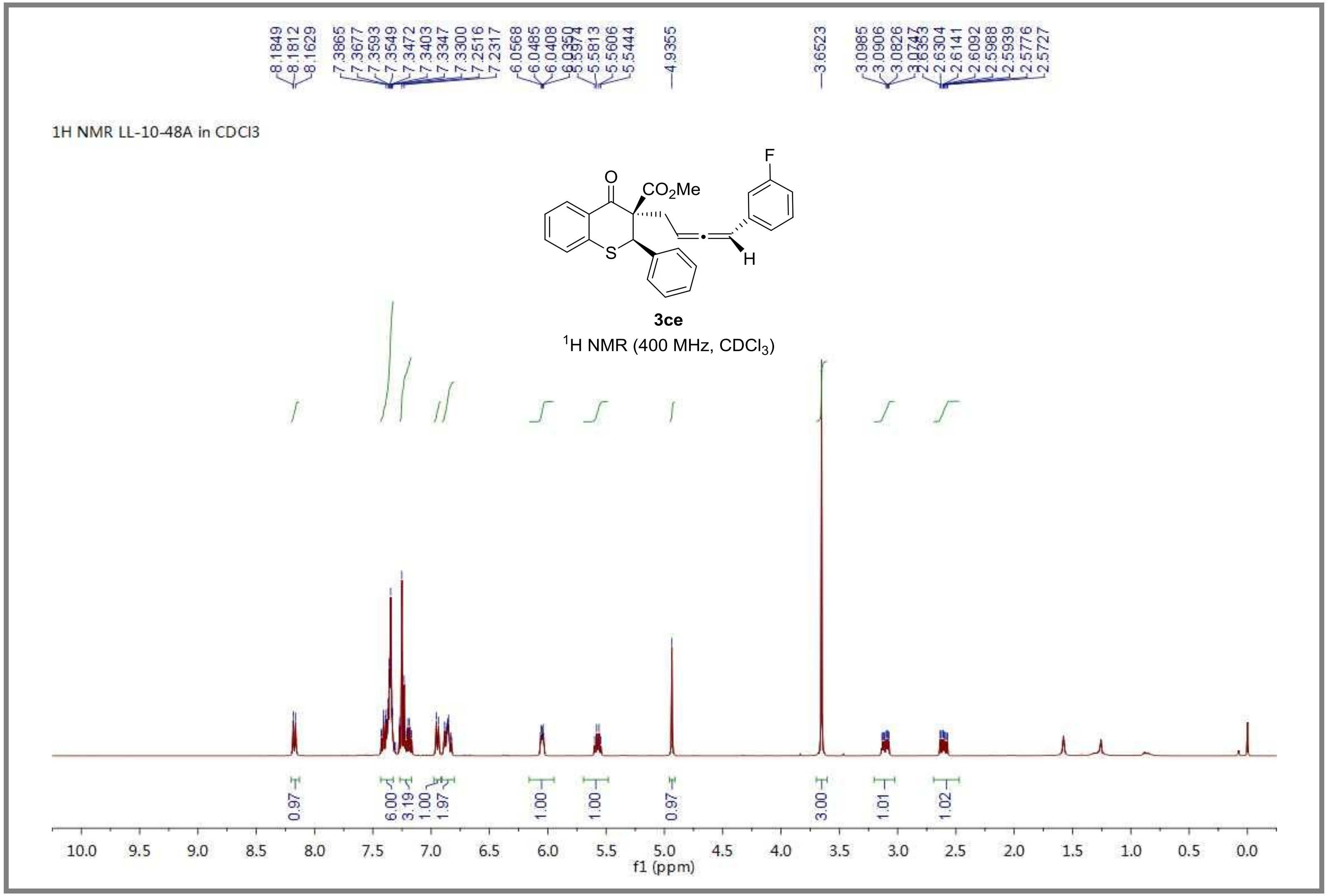
8.1849
8.1812
8.1629
7.3865
7.3677
7.3593
7.3549
7.3472
7.3403
7.3347
7.3300
7.2516
7.2317
6.0568
6.0485
6.0408
5.5974
5.5813
5.5606
5.5444
4.9355
3.6523
3.0985
3.0906
3.0826
2.6304
2.6141
2.6092
2.5988
2.5939
2.5776
2.5727
1H NMR LL-10-48A in CDCl3
O
CO2Me
F
S
H
3ce
1H NMR (400 MHz, CDCl3)
0.97
6.00
3.19
1.00
1.97
1.00
1.00
0.97
3.00
1.01
1.02
10.0
9.5
9.0
8.5
8.0
7.5
7.0
6.5
6.0
5.5
5.0
4.5
4.0
3.5
3.0
2.5
2.0
1.5
1.0
0.5
0.0
f1 (ppm)

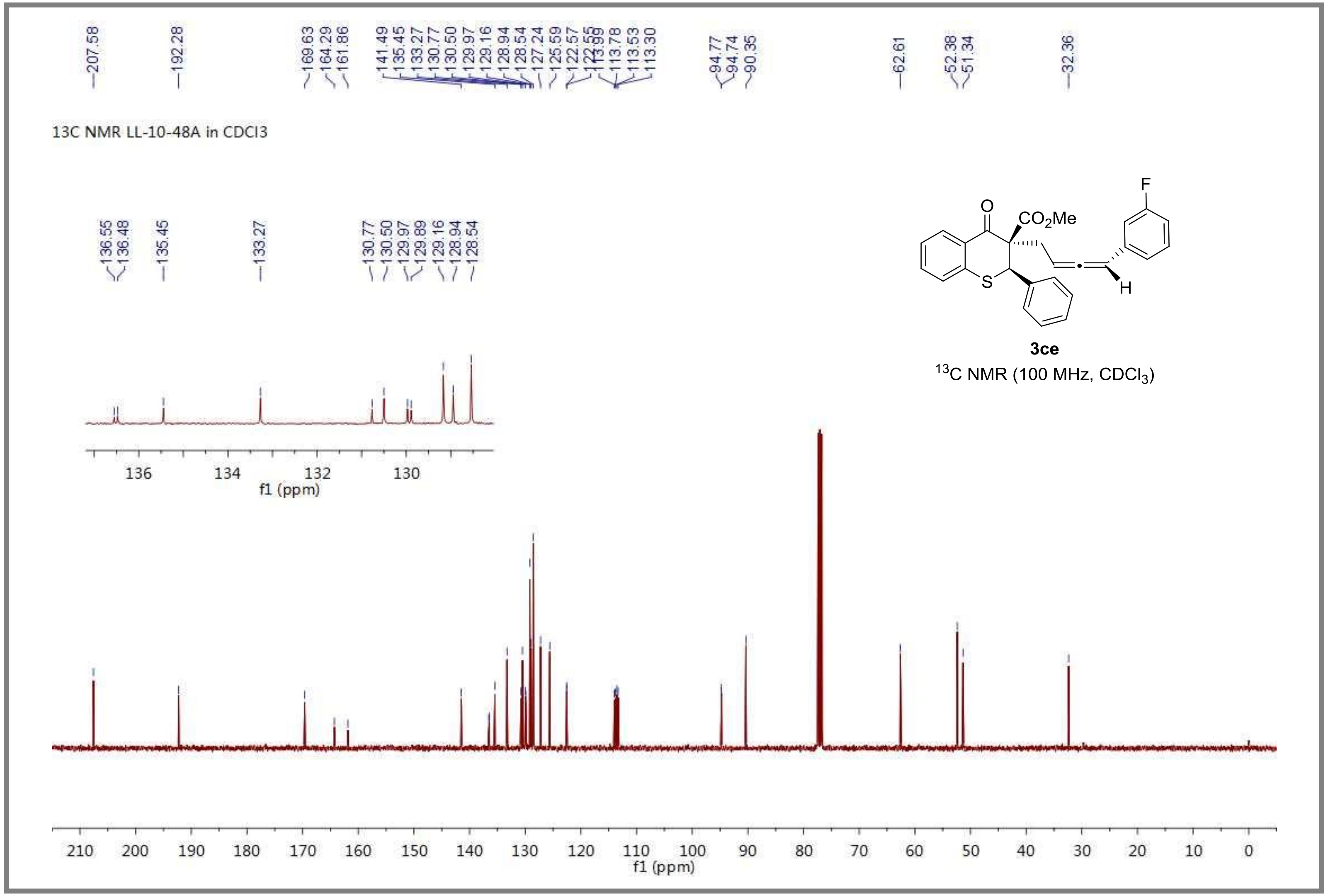

3ce

^{13}C NMR (100 MHz, $CDCl_3$)

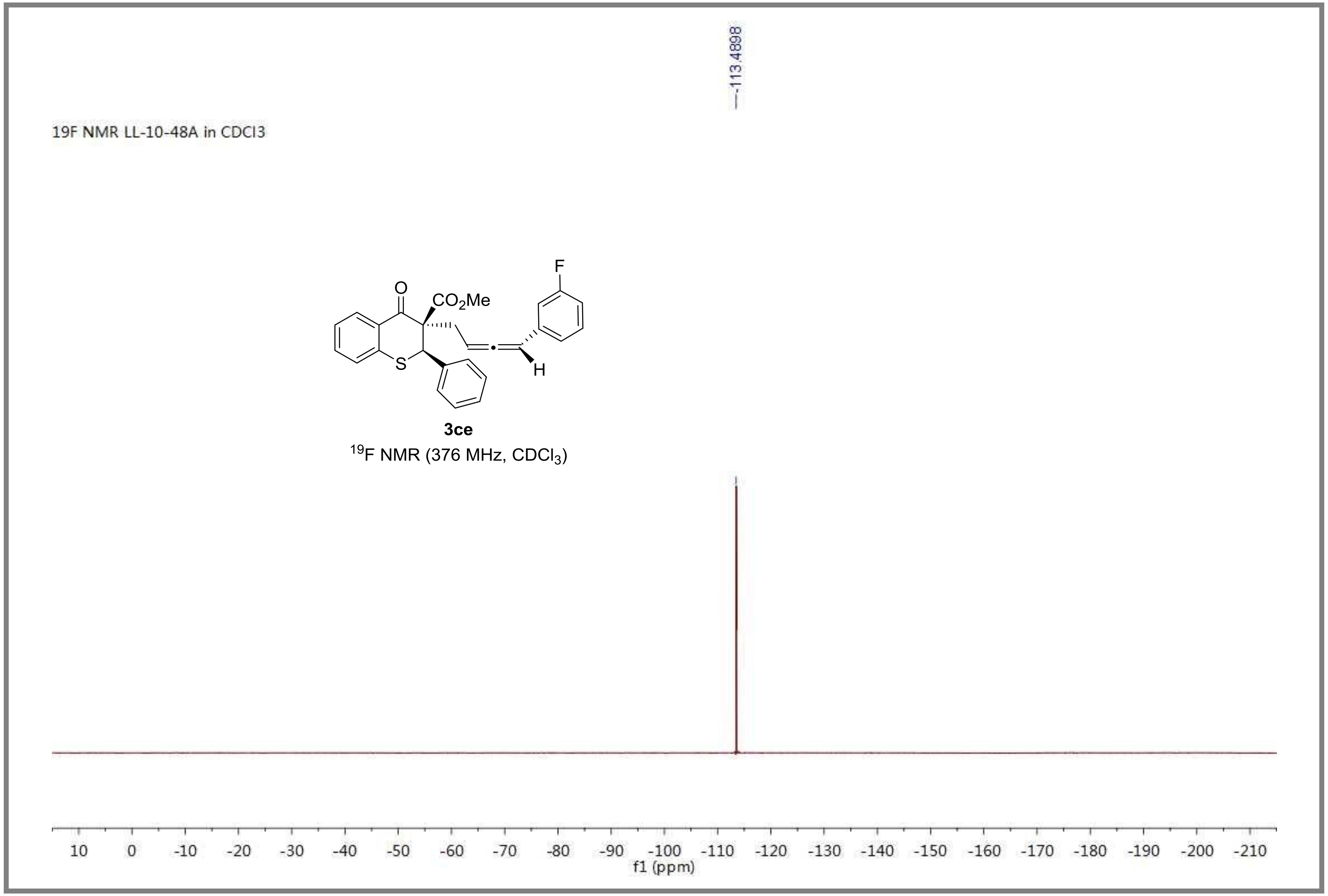
-113.4898
19F NMR LL-10-48A in CDCl3
O
CO2Me
S
H
F
3ce
19F NMR (376 MHz, CDCl3)
10 0 -10 -20 -30 -40 -50 -60 -70 -80 -90 -100 -110 -120 -130 -140 -150 -160 -170 -180 -190 -200 -210
f1 (ppm)

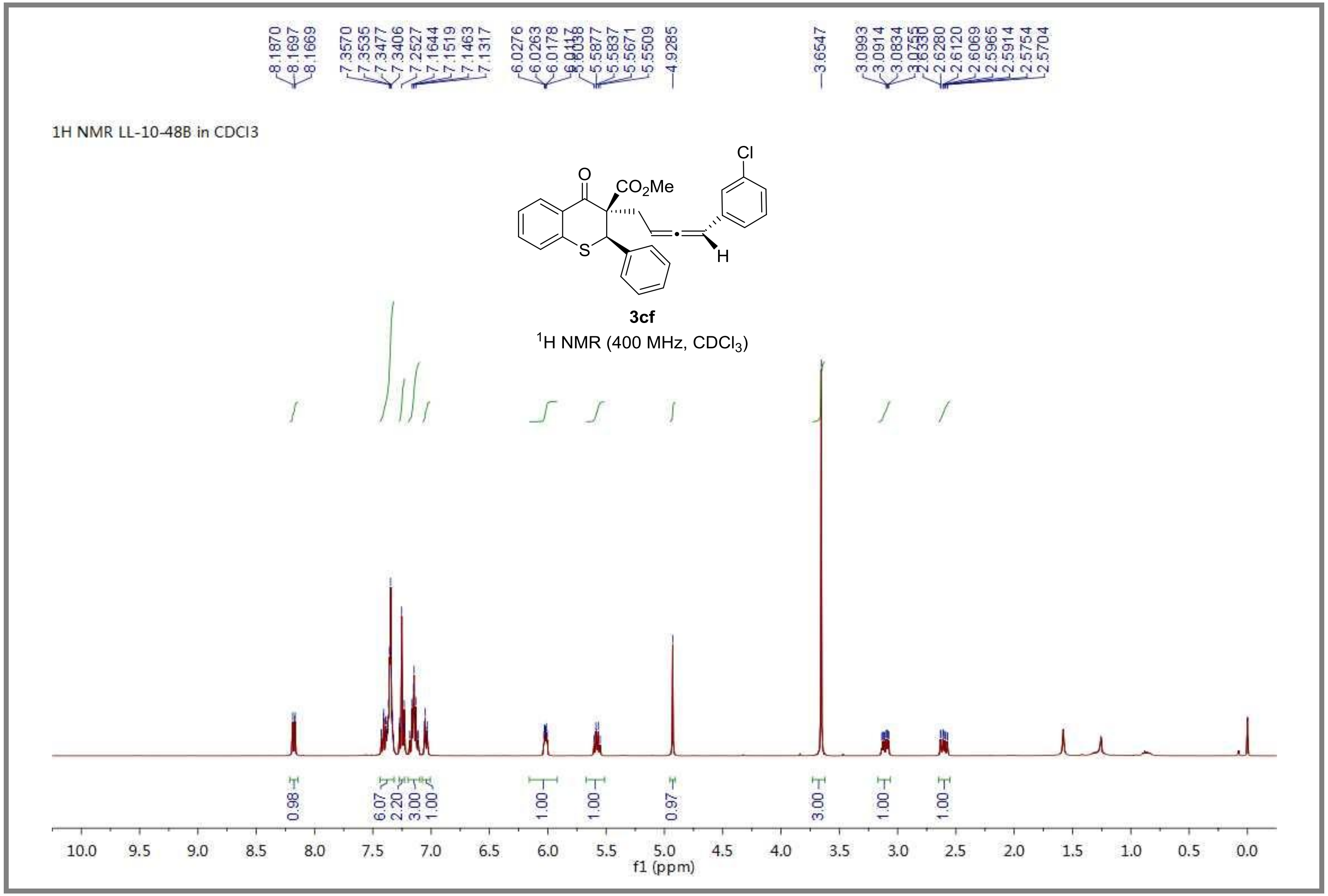

1H NMR LL-10-48B in CDCl3
8.1870
8.1697
8.1669
7.3570
7.3535
7.3477
7.3406
7.2527
7.1644
7.1519
7.1463
7.1317
6.0276
6.0263
6.0178
5.5877
5.5837
5.5671
5.5509
4.9285
3.6547
3.0993
3.0914
3.0834
2.6280
2.6120
2.6069
2.5965
2.5914
2.5754
2.5704
Cl
O
CO2Me
S
H
3cf
1H NMR (400 MHz, CDCl3)
0.98
6.07
2.20
3.00
1.00
1.00
1.00
0.97
3.00
1.00
1.00
10.0 9.5 9.0 8.5 8.0 7.5 7.0 6.5 6.0 5.5 5.0 4.5 4.0 3.5 3.0 2.5 2.0 1.5 1.0 0.5 0.0
f1 (ppm)

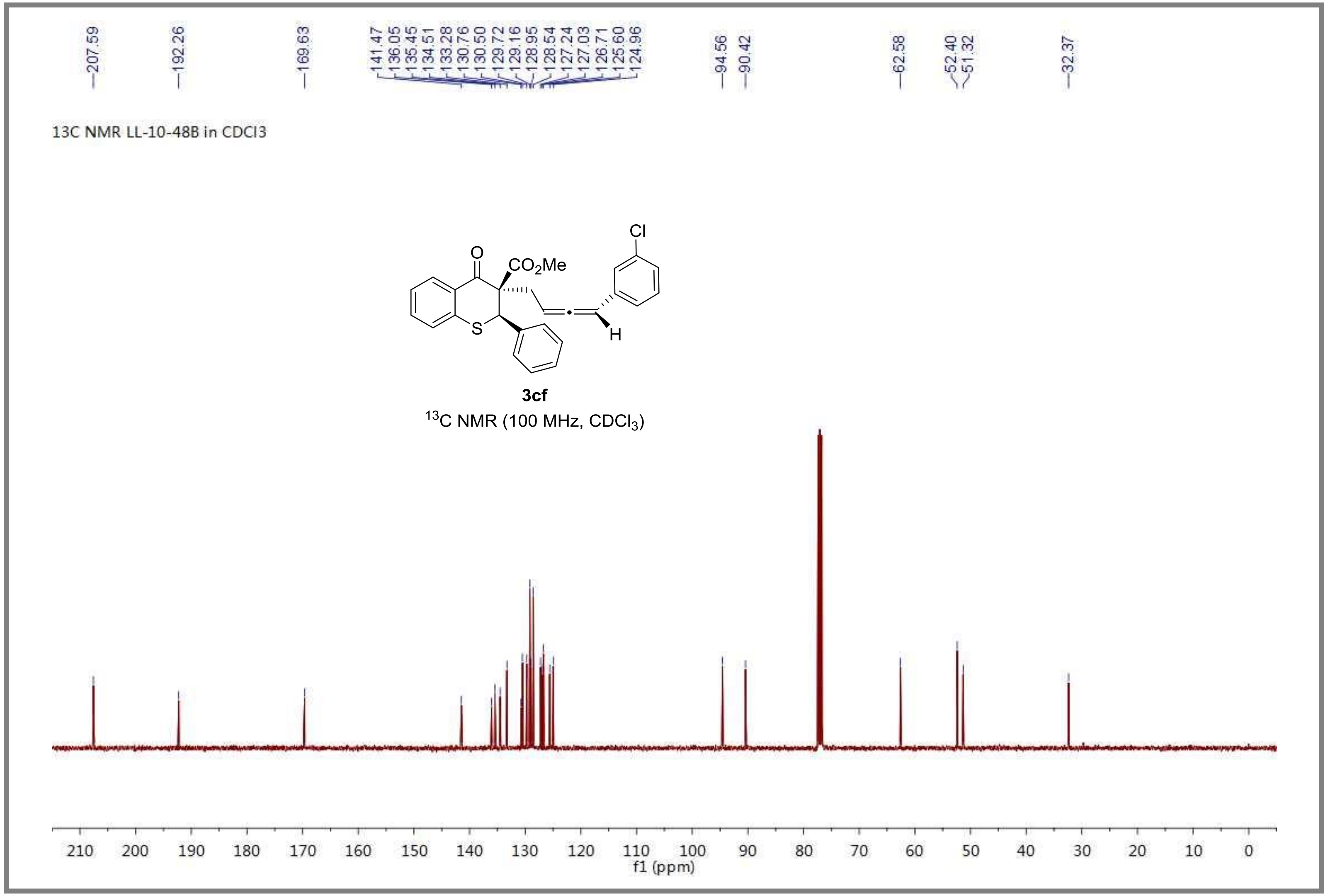

3cf

^{13}C NMR (100 MHz, $CDCl_3$)

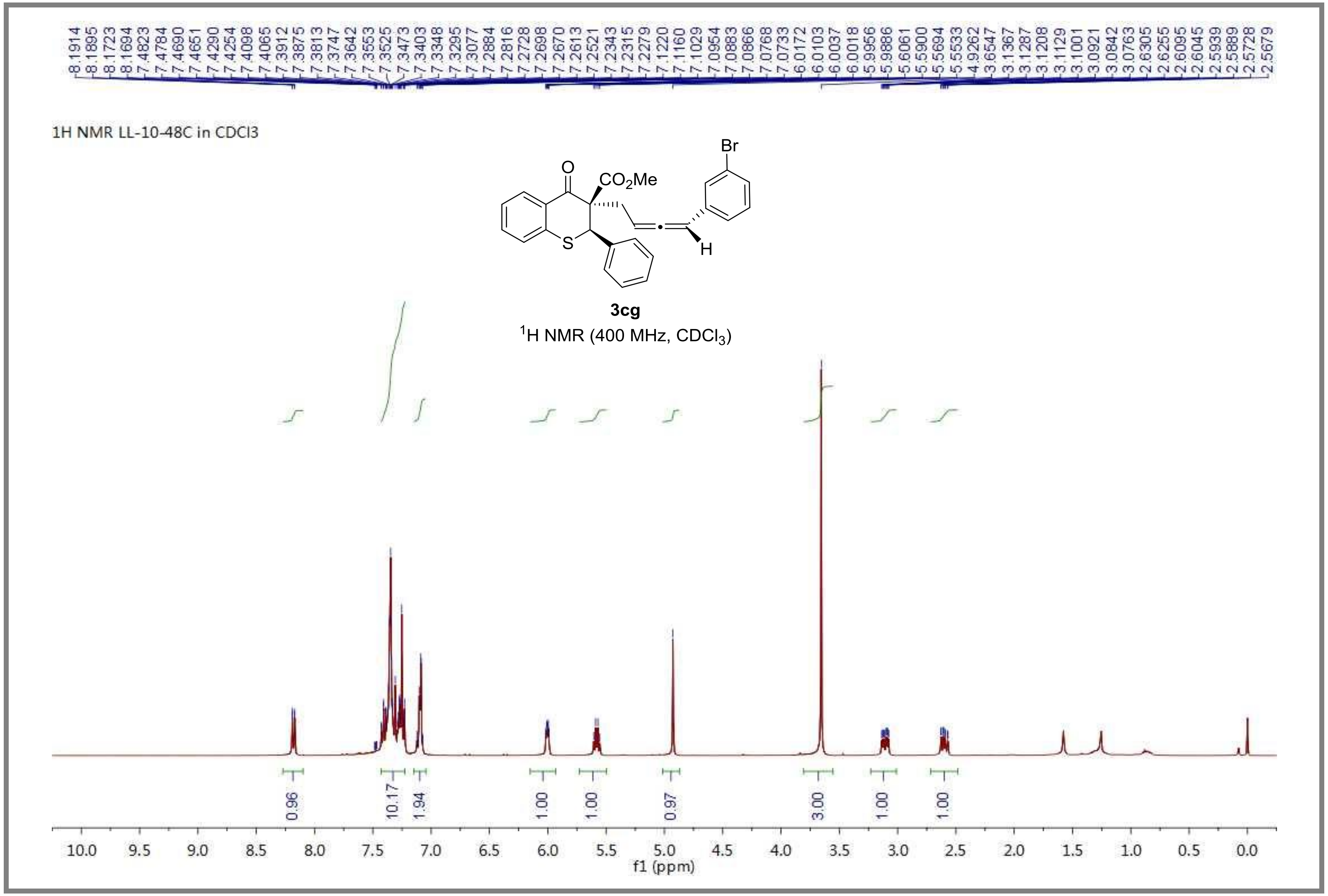
1H NMR LL-10-48C in CDCl3
Br
CO2Me
O
S
H
3cg
1H NMR (400 MHz, CDCl3)
f1 (ppm)

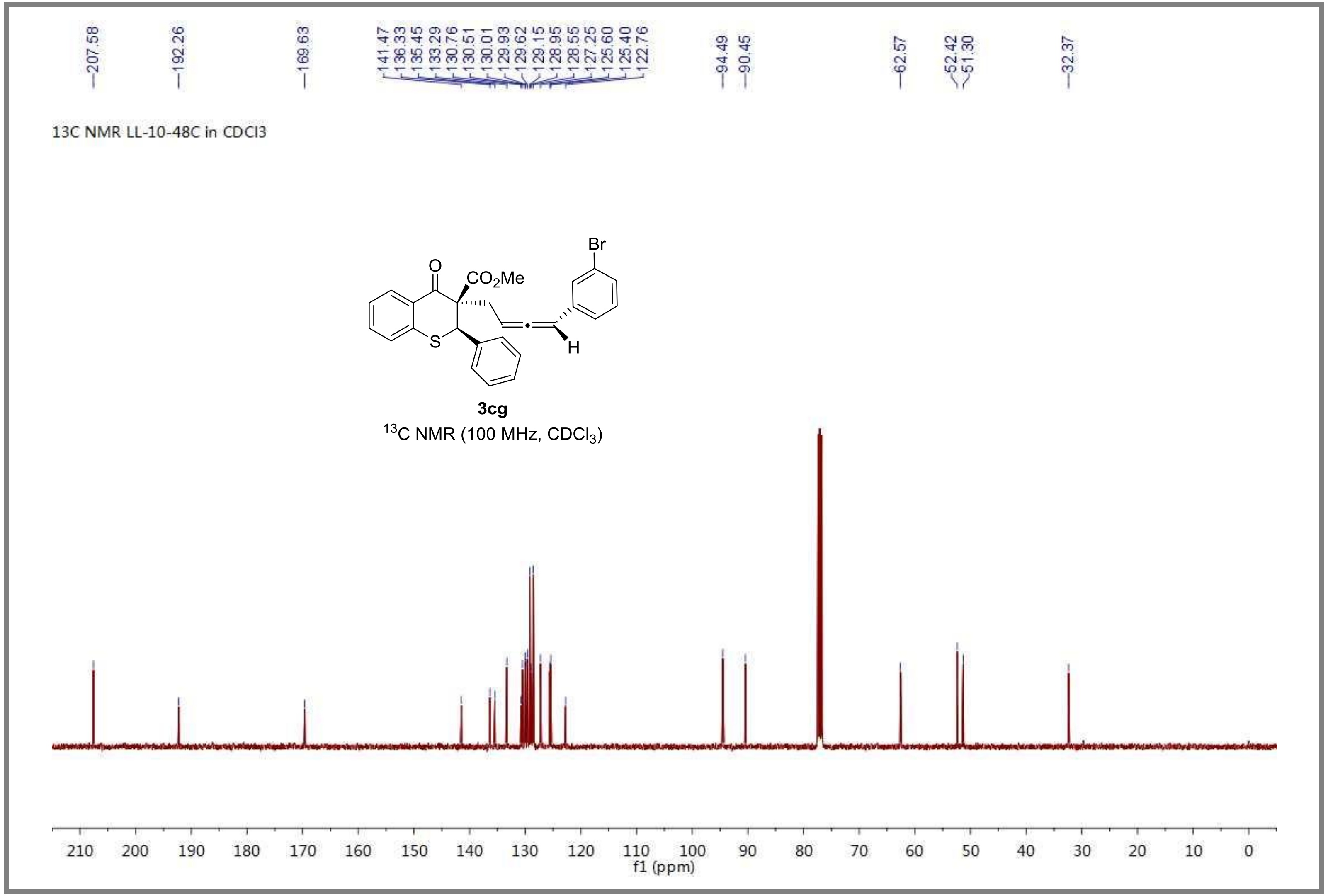

207.58
192.26
169.63
141.47
136.33
135.45
133.29
130.76
130.51
130.01
129.93
129.62
129.15
128.95
128.55
127.25
125.60
125.40
122.76
94.49
90.45
62.57
52.42
51.30
32.37
13C NMR LL-10-48C in CDCl3
Br
O
CO2Me
S
H
3cg
13C NMR (100 MHz, CDCl3)
210 200 190 180 170 160 150 140 130 120 110 100 90 80 70 60 50 40 30 20 10 0
f1 (ppm)

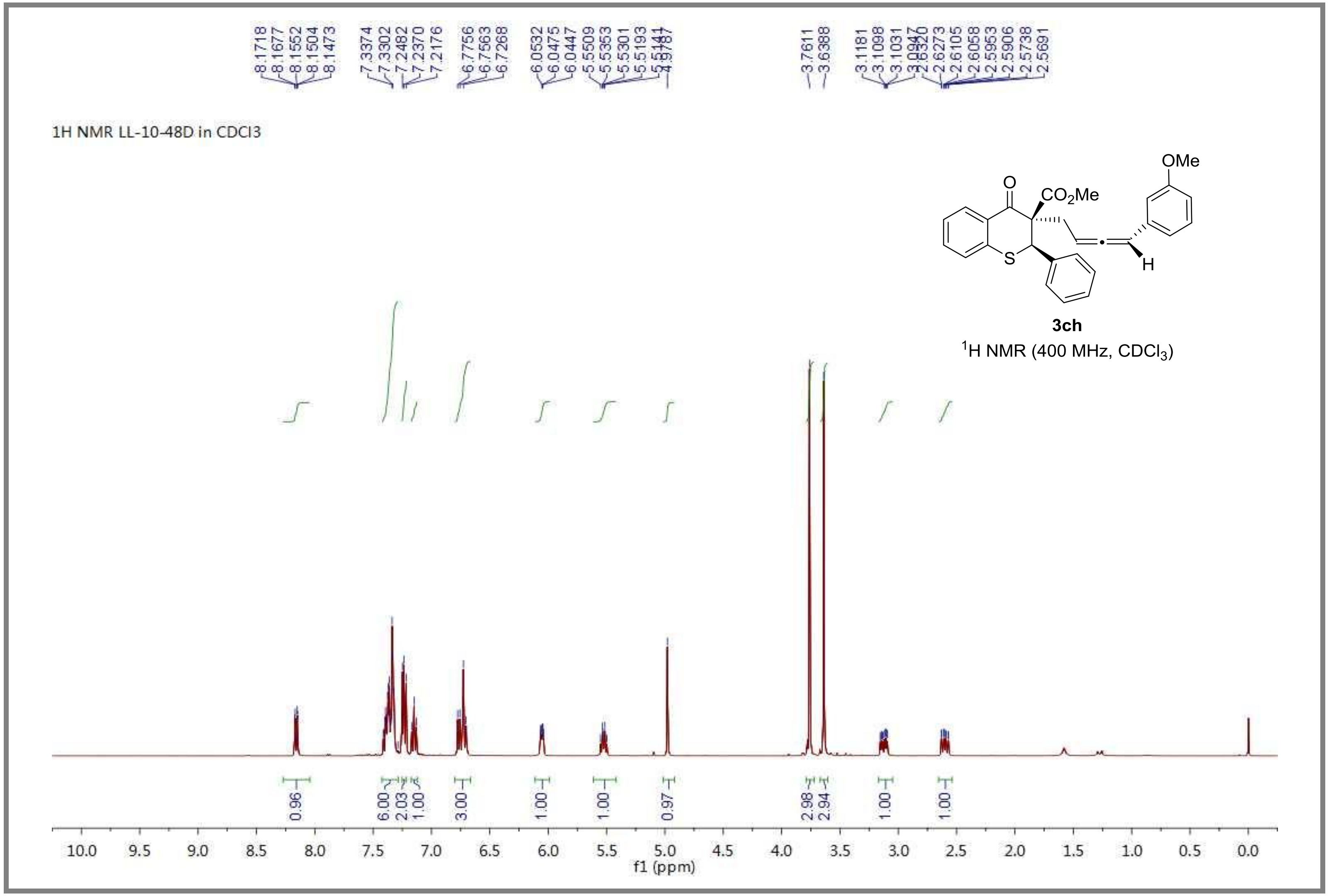

3ch

^{1}H NMR (400 MHz, $CDCl_3$)

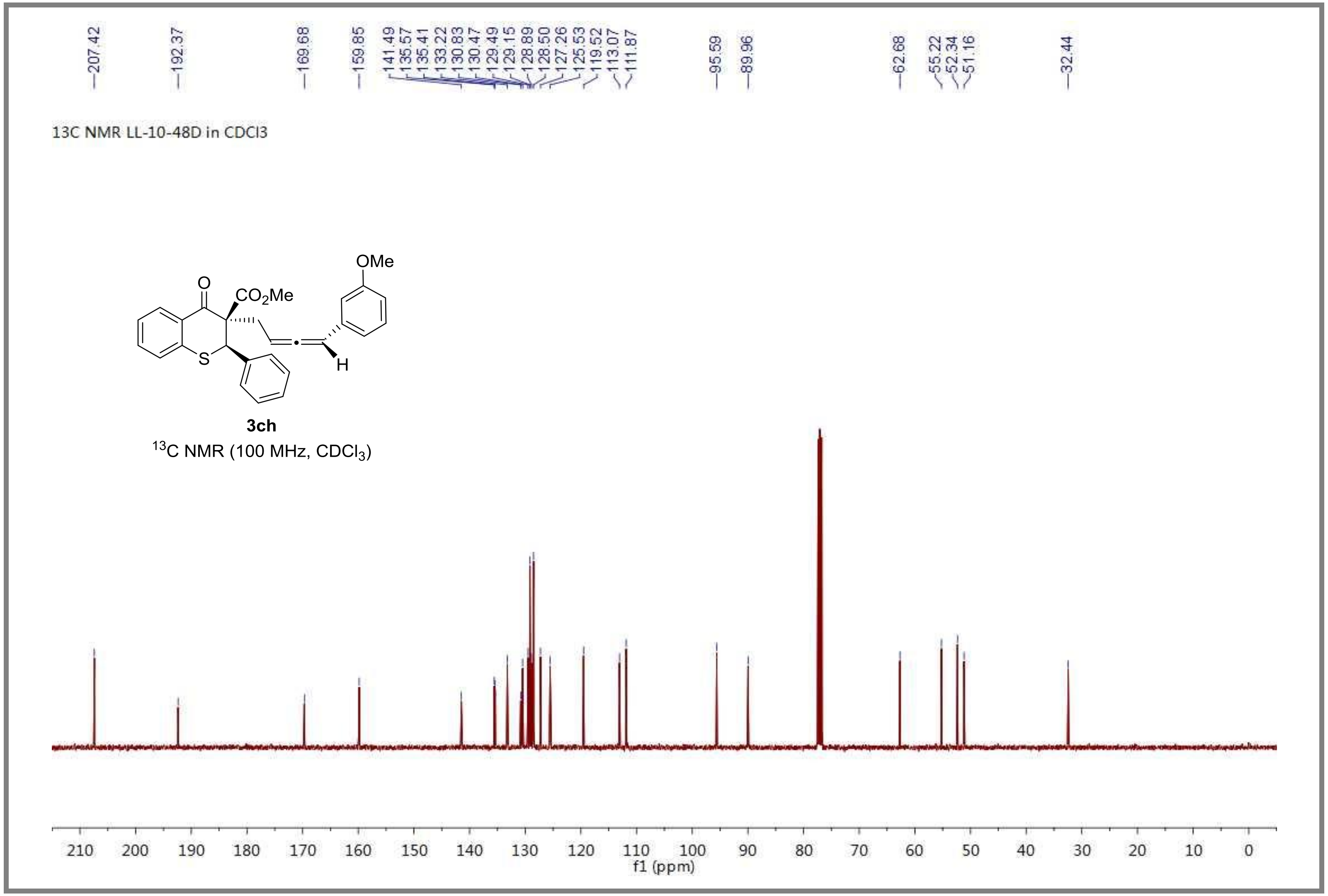
207.42
192.37
169.68
159.85
141.49
135.57
135.41
133.22
130.83
130.47
129.49
129.15
128.89
128.50
127.26
125.53
119.52
113.07
111.87
95.59
89.96
62.68
55.22
52.34
51.16
32.44
13C NMR LL-10-48D in CDCl3
OMe
O
CO2Me
S
H
3ch
13C NMR (100 MHz, CDCl3)
210 200 190 180 170 160 150 140 130 120 110 100 90 80 70 60 50 40 30 20 10 0
f1 (ppm)

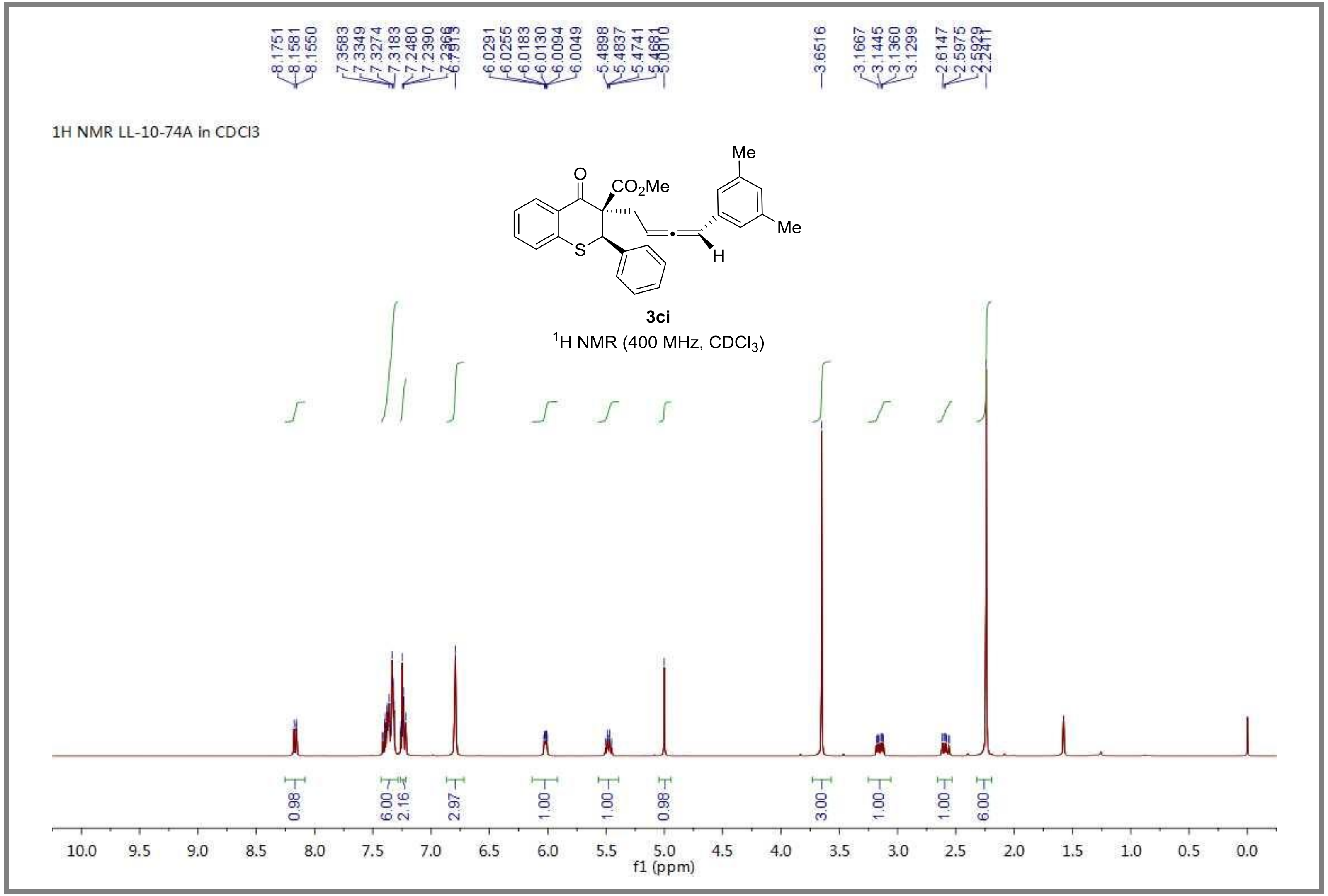
1H NMR LL-10-74A in CDCl3
3ci
^{1}H NMR (400 MHz, $CDCl_3$)
O
CO_2Me
Me
Me
S
H
8.1751
8.1581
8.1550
7.3583
7.3349
7.3274
7.3183
7.2480
7.2390
7.2366
6.7913
6.0291
6.0255
6.0183
6.0130
6.0094
6.0049
5.4898
5.4837
5.4741
5.4681
5.0010
3.6516
3.1667
3.1445
3.1360
3.1299
2.6147
2.5975
2.5929
2.2411
0.98
6.00
2.16
2.97
1.00
1.00
0.98
3.00
1.00
1.00
6.00
f1 (ppm)

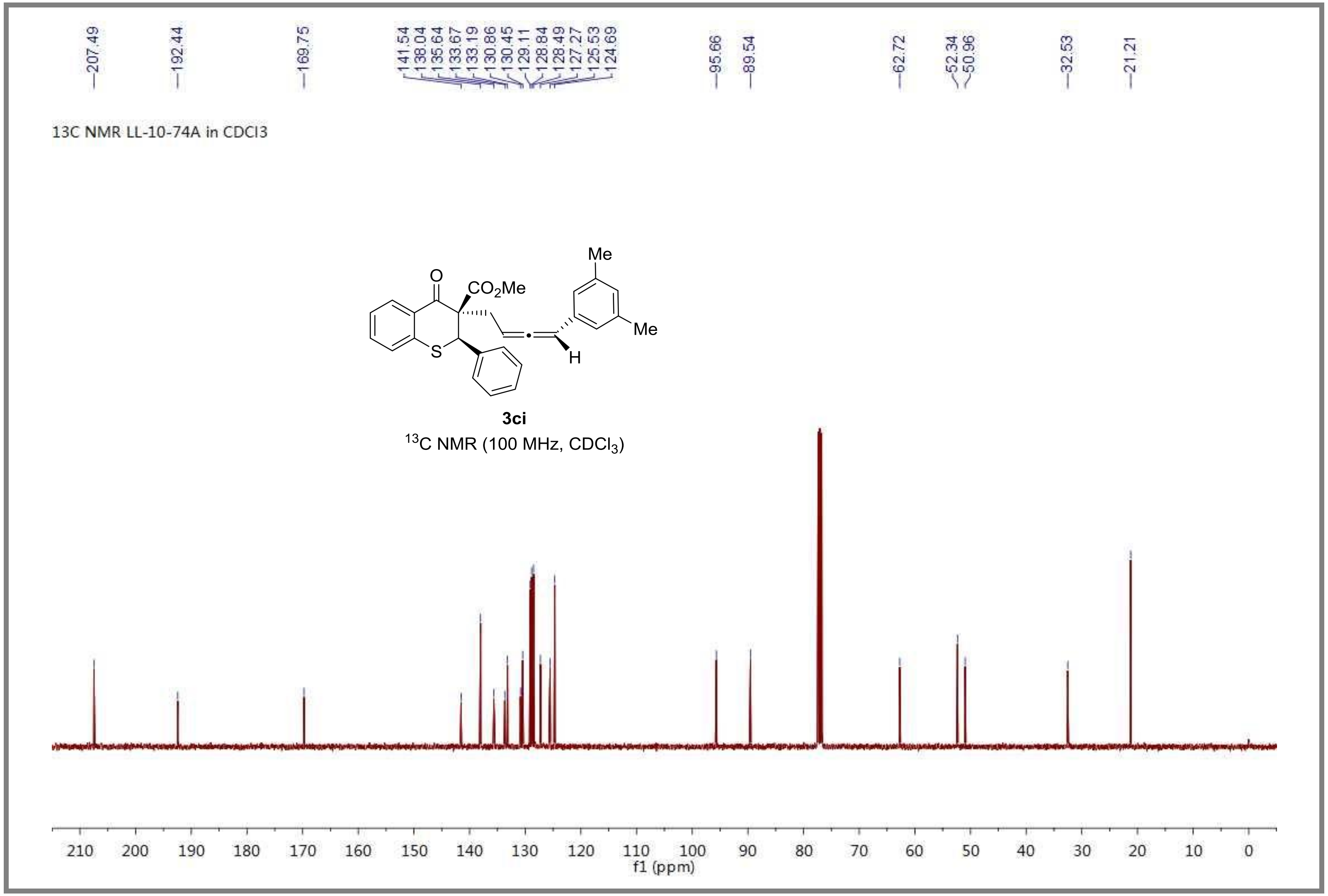
207.49
192.44
169.75
141.54
138.04
135.64
133.67
133.19
130.86
130.45
129.11
128.84
128.49
127.27
125.53
124.69
95.66
89.54
62.72
52.34
50.96
32.53
21.21
13C NMR LL-10-74A in CDCl3
O
CO_2Me
Me
Me
S
H
3ci
^{13}C NMR (100 MHz, $CDCl_3$)
210 200 190 180 170 160 150 140 130 120 110 100 90 80 70 60 50 40 30 20 10 0
f1 (ppm)

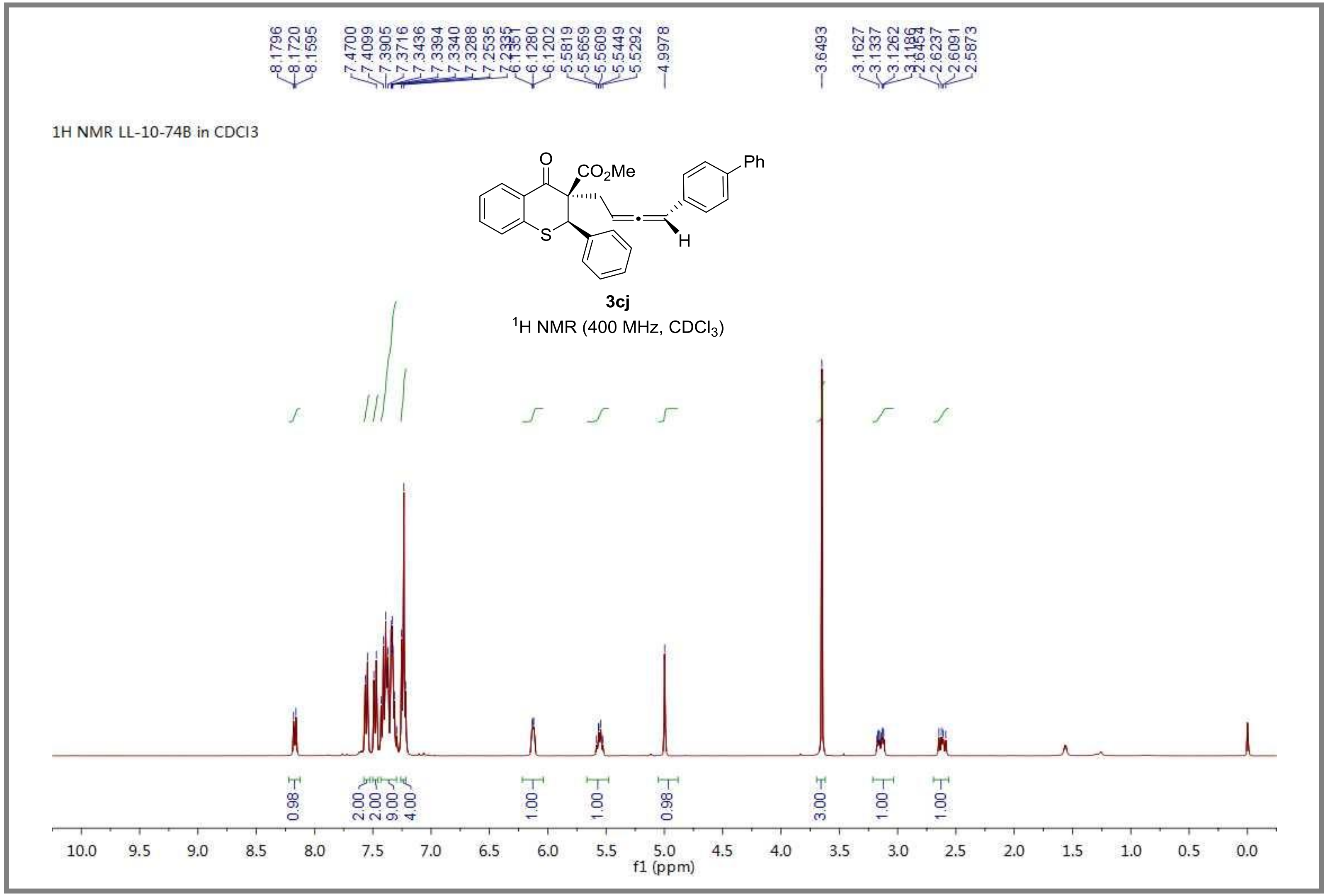
1H NMR LL-10-74B in CDCl3
3cj
^{1}H NMR (400 MHz, CDCl$_3$)
f1 (ppm)

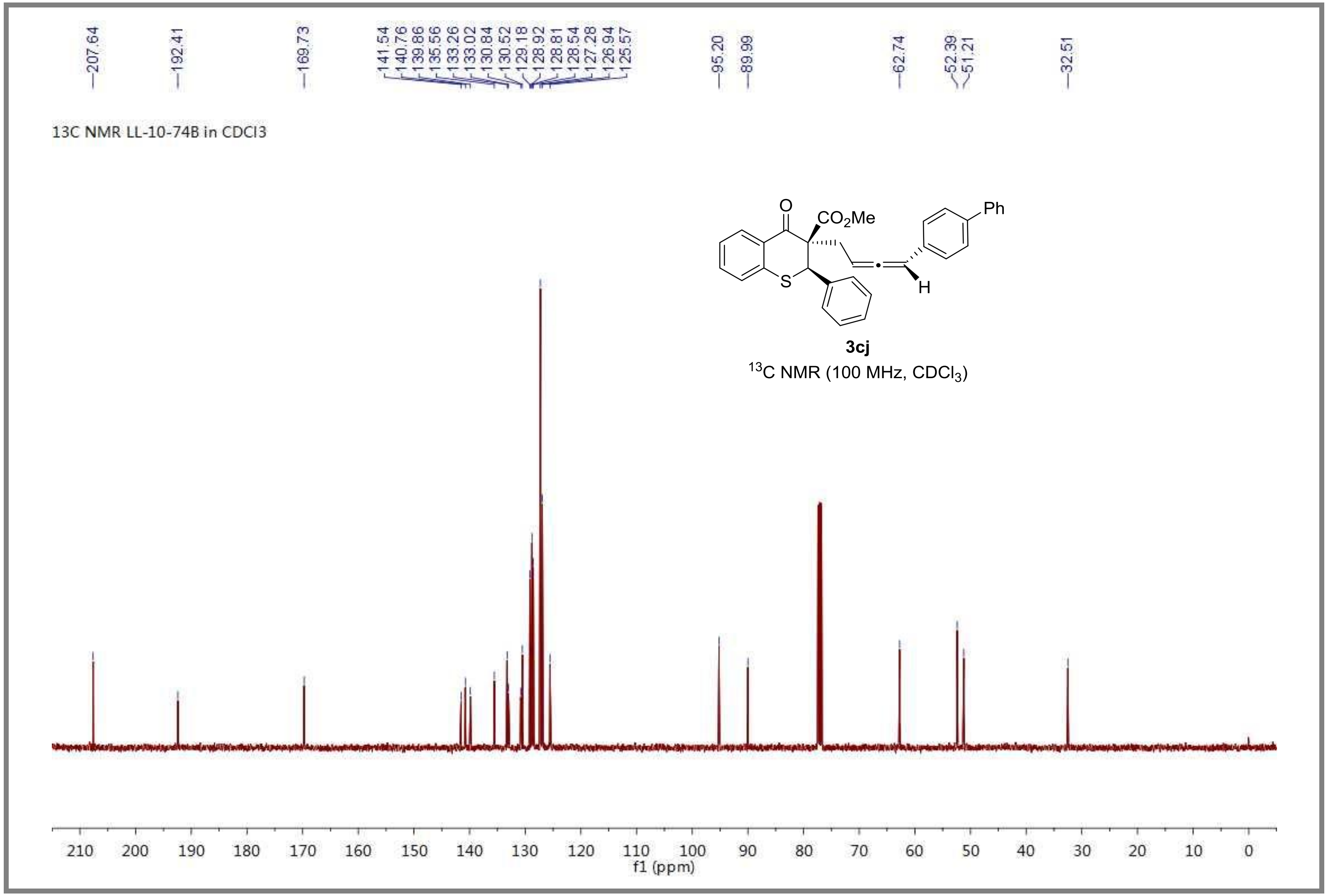
207.64
192.41
169.73
141.54
140.76
139.86
135.56
133.26
133.02
130.84
130.52
129.18
128.92
128.81
128.54
127.28
126.94
125.57
95.20
89.99
62.74
52.39
51.21
32.51
13C NMR LL-10-74B in CDCl3
O
CO2Me
Ph
S
H
3cj
13C NMR (100 MHz, CDCl3)
210 200 190 180 170 160 150 140 130 120 110 100 90 80 70 60 50 40 30 20 10 0
f1 (ppm)

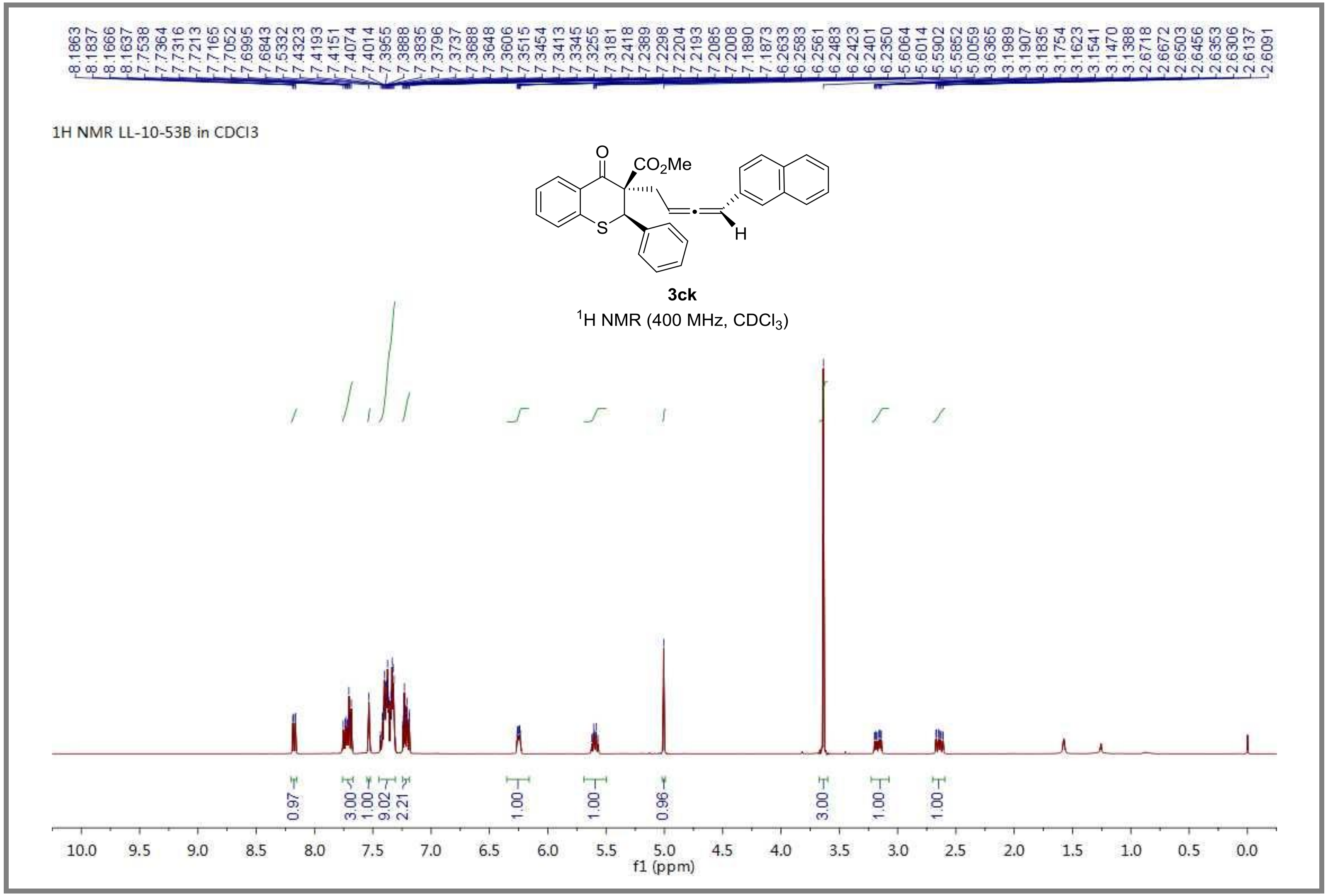
8.1863
8.1837
8.1666
8.1637
7.7538
7.7364
7.7316
7.7213
7.7165
7.7052
7.6995
7.6843
7.5332
7.4323
7.4193
7.4151
7.4074
7.4014
7.3955
7.3888
7.3835
7.3796
7.3737
7.3688
7.3648
7.3606
7.3515
7.3454
7.3413
7.3345
7.3255
7.3181
7.2418
7.2389
7.2298
7.2204
7.2193
7.2085
7.2008
7.1890
7.1873
6.2633
6.2583
6.2561
6.2483
6.2423
6.2401
6.2350
5.6064
5.6014
5.5902
5.5852
5.0059
3.6365
3.1989
3.1907
3.1835
3.1754
3.1623
3.1541
3.1470
3.1388
2.6718
2.6672
2.6503
2.6456
2.6353
2.6306
2.6137
2.6091
1H NMR LL-10-53B in CDCl3
O
CO2Me
S
H
3ck
1H NMR (400 MHz, CDCl3)
0.97
3.00
1.00
9.02
2.21
1.00
1.00
0.96
3.00
1.00
1.00
10.0
9.5
9.0
8.5
8.0
7.5
7.0
6.5
6.0
5.5
5.0
4.5
4.0
3.5
3.0
2.5
2.0
1.5
1.0
0.5
0.0
f1 (ppm)

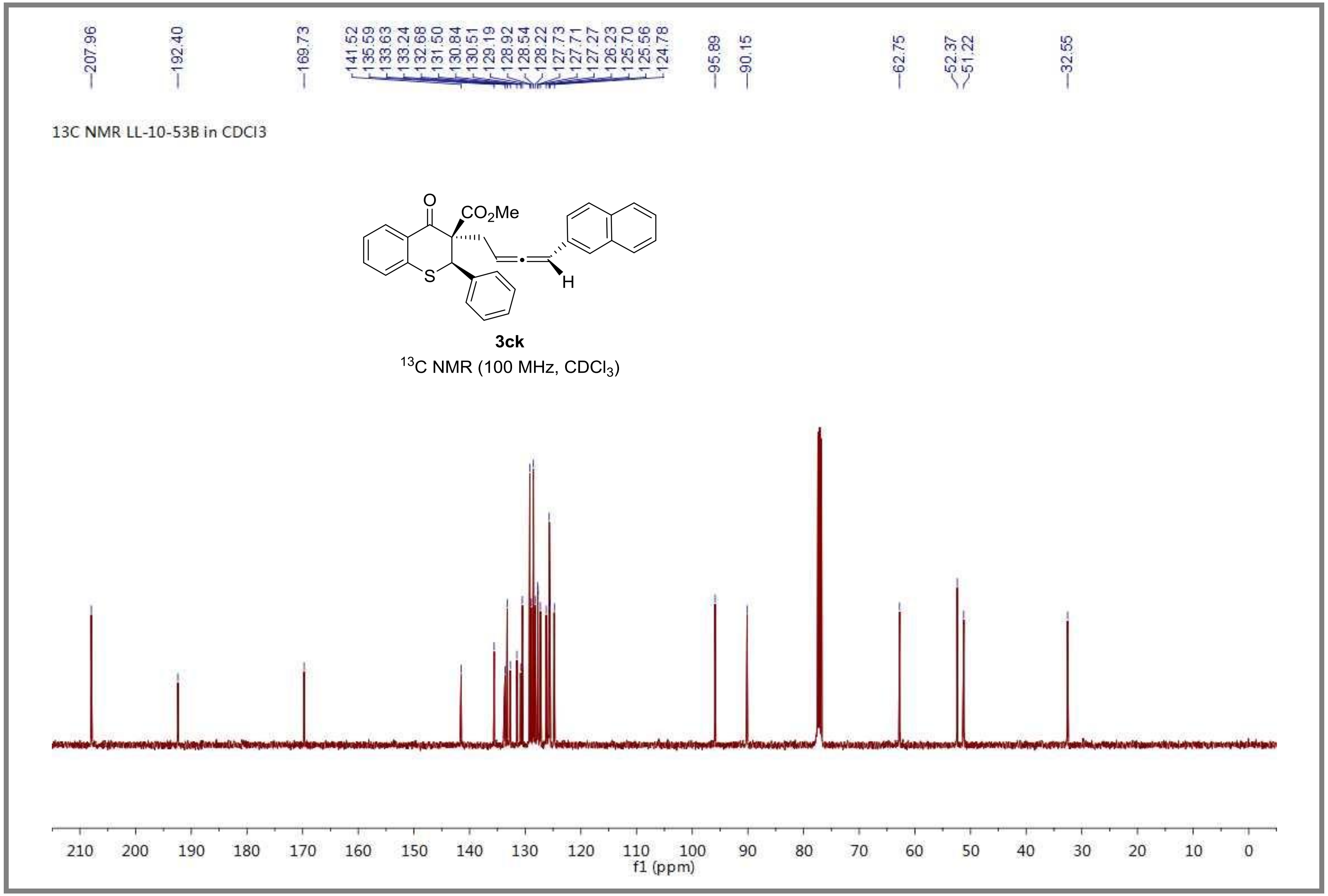
207.96
192.40
169.73
141.52
135.59
133.63
133.24
132.68
131.50
130.84
130.51
129.19
128.92
128.54
128.22
127.73
127.71
127.27
126.23
125.70
125.56
124.78
95.89
90.15
62.75
52.37
51.22
32.55
13C NMR LL-10-53B in CDCl3
O
CO2Me
S
H
3ck
13C NMR (100 MHz, CDCl3)
210 200 190 180 170 160 150 140 130 120 110 100 90 80 70 60 50 40 30 20 10 0
f1 (ppm)

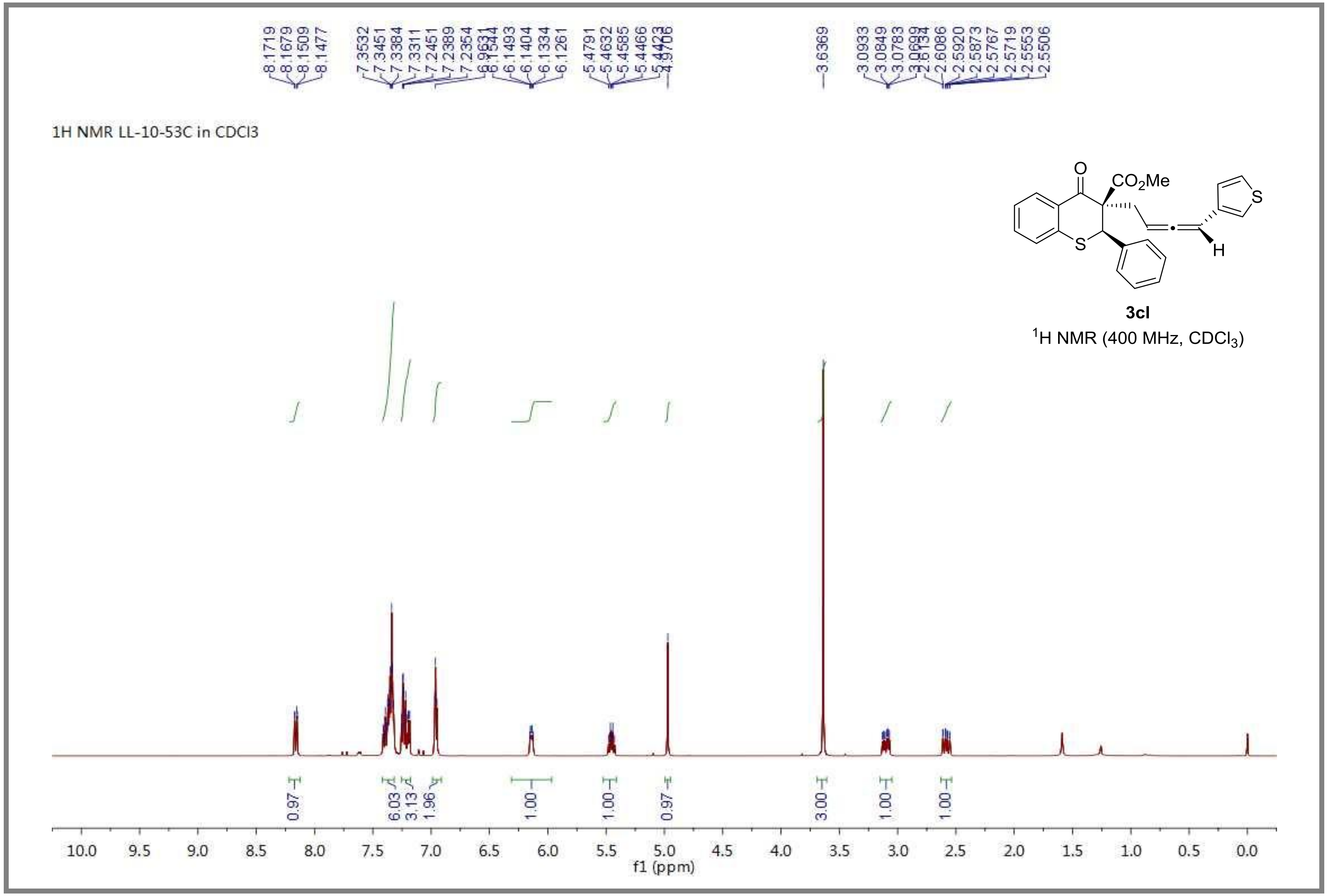
1H NMR LL-10-53C in CDCl3
3cl
1H NMR (400 MHz, CDCl3)
CO2Me
S
H
O
f1 (ppm)

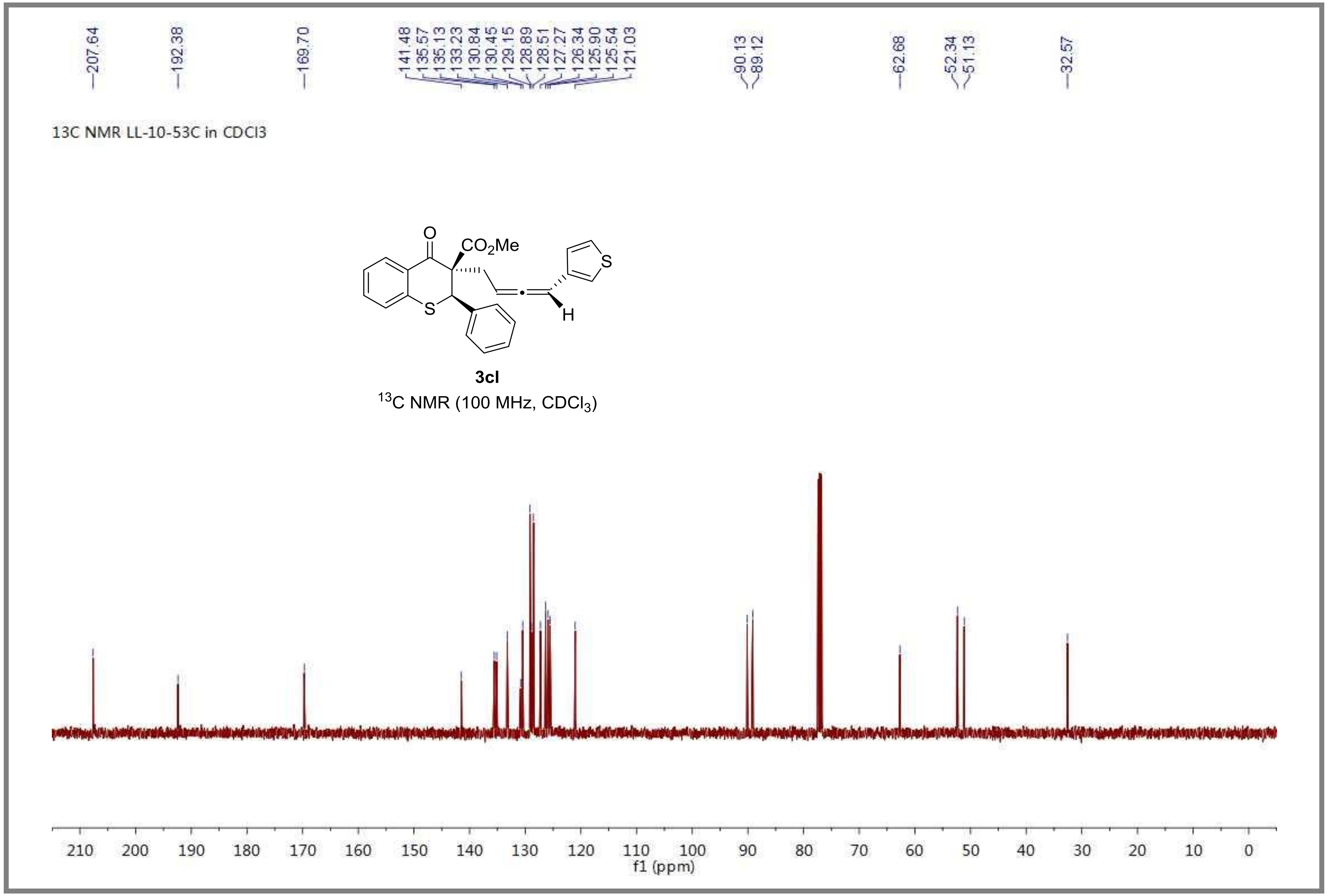
207.64
192.38
169.70
141.48
135.57
135.13
133.23
130.84
130.45
129.15
128.89
128.51
127.27
126.34
125.90
125.54
121.03
90.13
89.12
62.68
52.34
51.13
32.57
13C NMR LL-10-53C in CDCl3
O
CO2Me
S
S
H
3cl
13C NMR (100 MHz, CDCl3)
210 200 190 180 170 160 150 140 130 120 110 100 90 80 70 60 50 40 30 20 10 0
f1 (ppm)

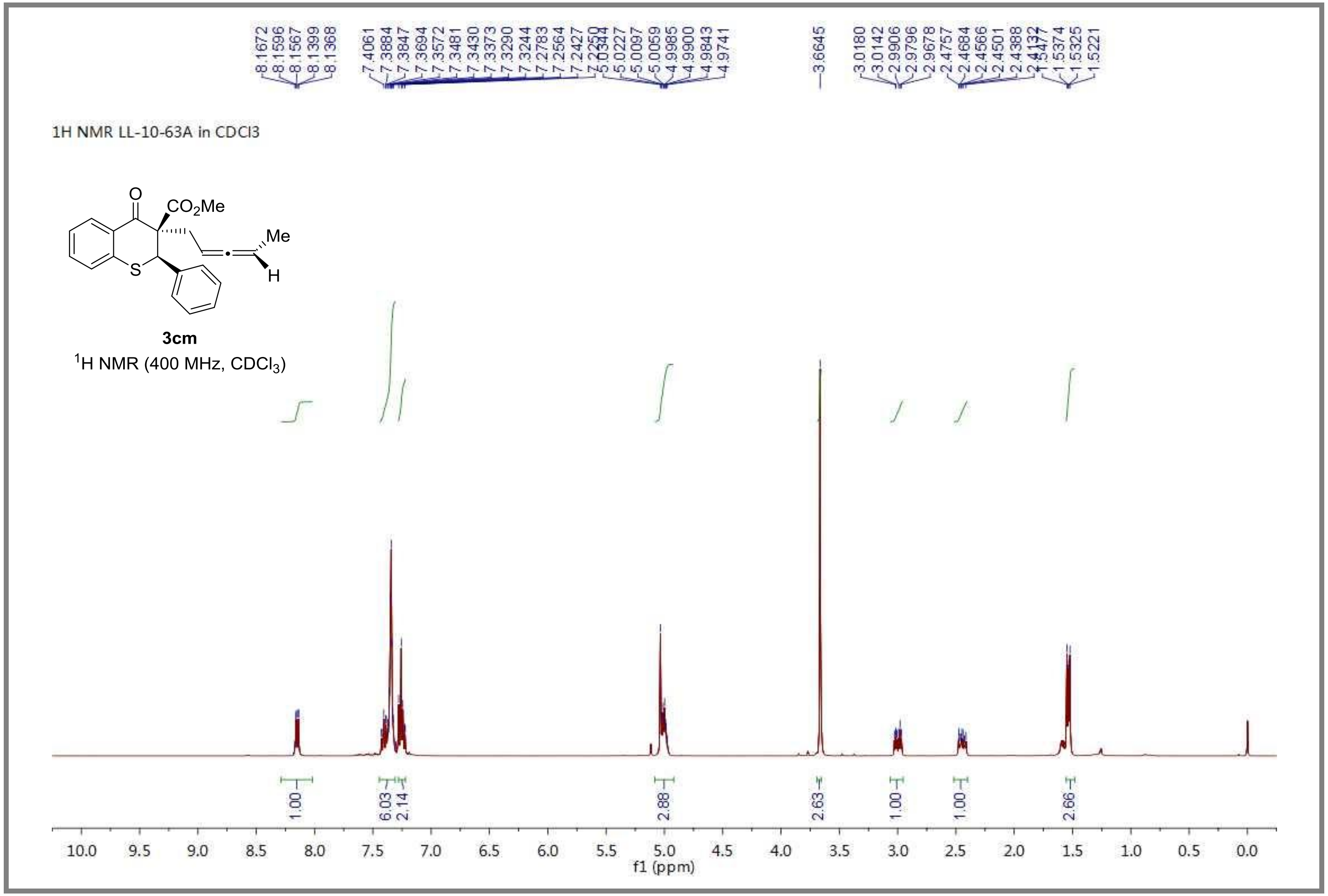
1H NMR LL-10-63A in CDCl3
CO2Me
Me
S
H
O
3cm
1H NMR (400 MHz, CDCl3)
f1 (ppm)

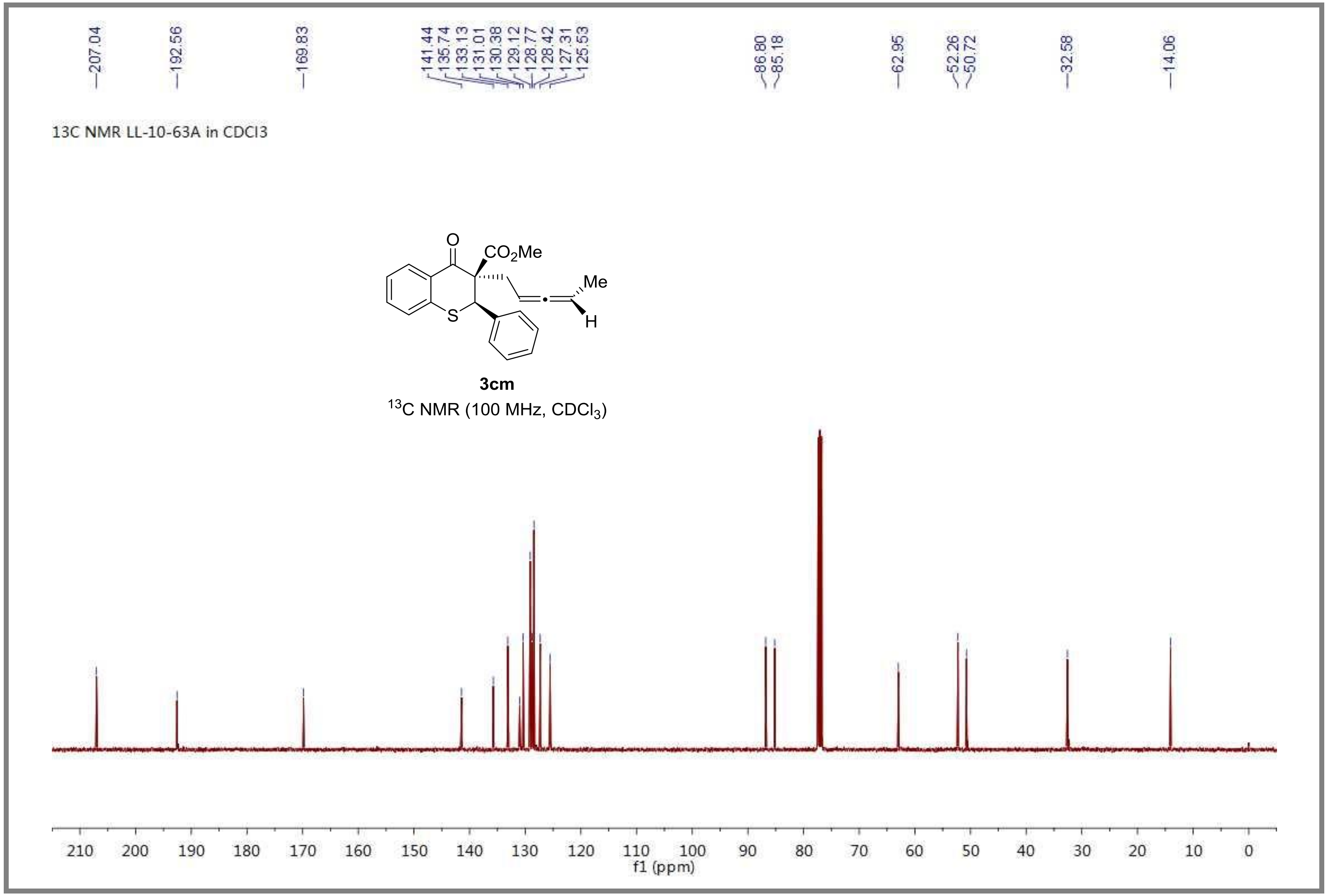
207.04
192.56
169.83
141.44
135.74
133.13
131.01
130.38
129.12
128.77
128.42
127.31
125.53
86.80
85.18
62.95
52.26
50.72
32.58
14.06
13C NMR LL-10-63A in CDCl3
O
CO_2Me
Me
S
H
3cm
^{13}C NMR (100 MHz, $CDCl_3$)
210
200
190
180
170
160
150
140
130
120
110
100
90
80
70
60
50
40
30
20
10
0
f1 (ppm)

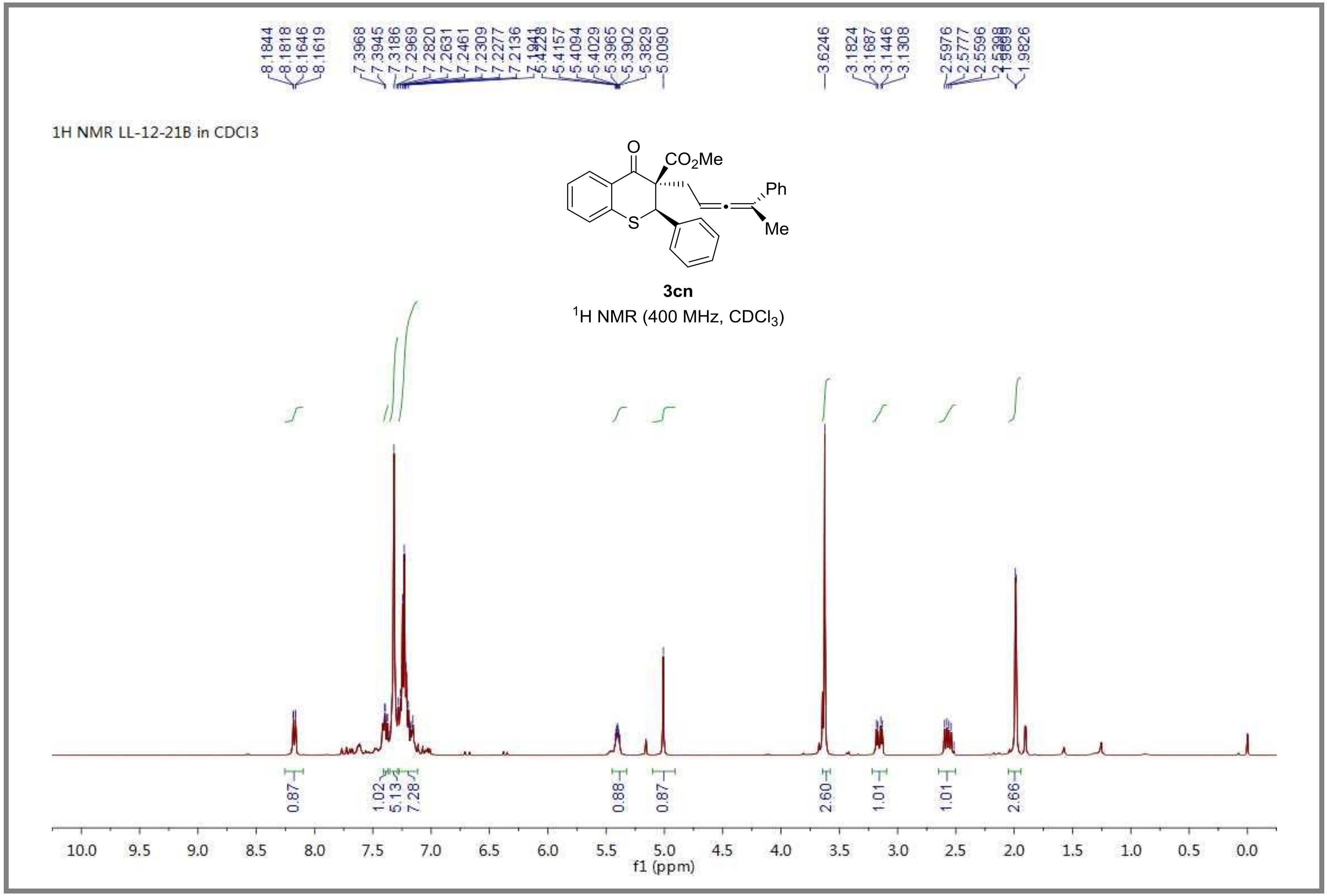
1H NMR LL-12-21B in CDCl3
3cn
1H NMR (400 MHz, CDCl3)
f1 (ppm)

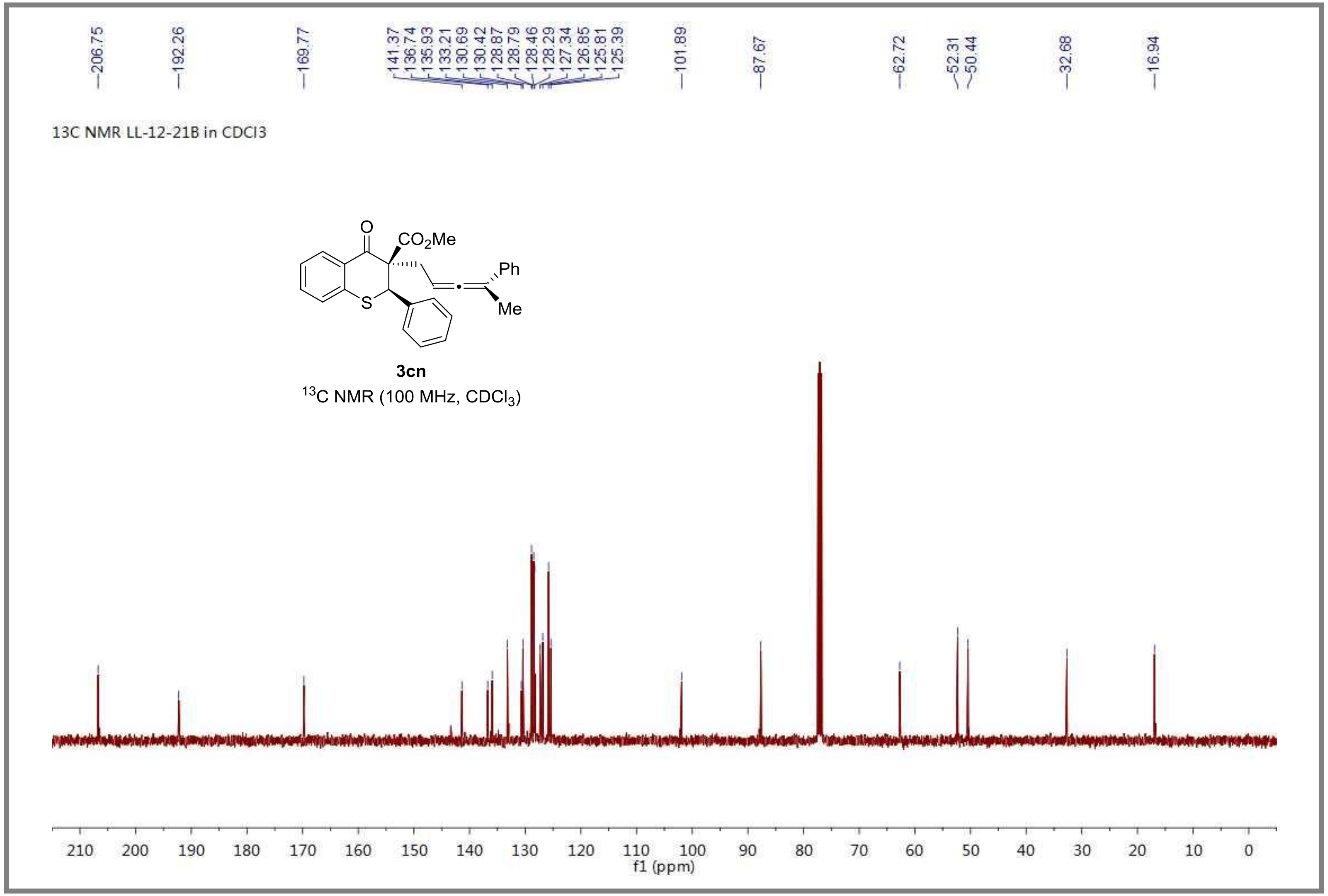

206.75
192.26
169.77
141.37
136.74
135.93
133.21
130.69
130.42
128.87
128.79
128.46
128.29
127.34
126.85
125.81
125.39
101.89
87.67
62.72
52.31
50.44
32.68
16.94
13C NMR LL-12-21B in CDCl3
O
CO_2Me
Ph
S
Me
3cn
^{13}C NMR (100 MHz, $CDCl_3$)
210 200 190 180 170 160 150 140 130 120 110 100 90 80 70 60 50 40 30 20 10 0
f1 (ppm)

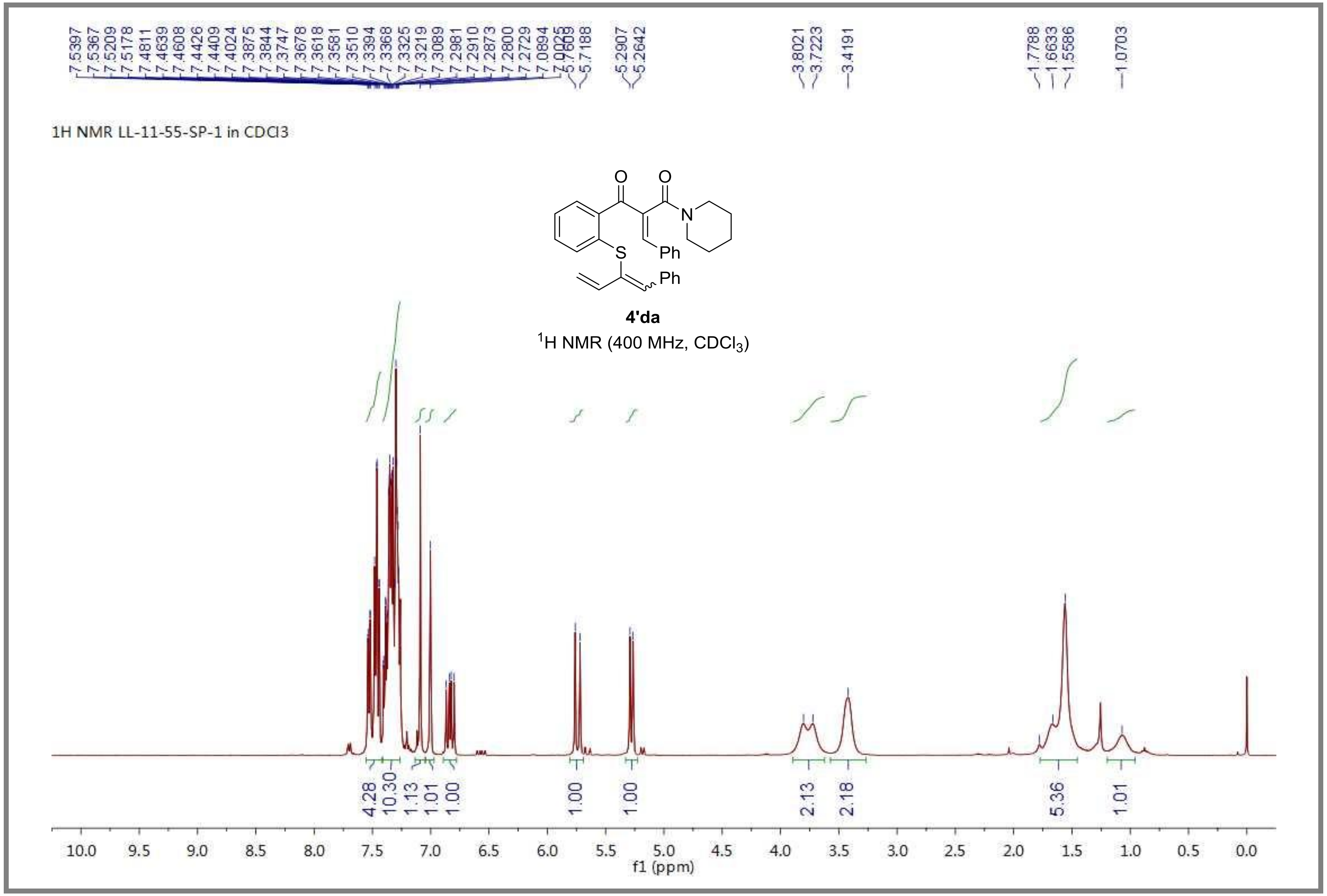
1H NMR LL-11-55-SP-1 in CDCl3
4'da
^{1}H NMR (400 MHz, $CDCl_3$)
7.5397 7.5367 7.5209 7.5178 7.4811 7.4639 7.4608 7.4426 7.4409 7.4024 7.3875 7.3844 7.3747 7.3678 7.3618 7.3581 7.3510 7.3394 7.3368 7.3325 7.3219 7.3089 7.2981 7.2910 7.2873 7.2800 7.2729 7.0894 7.0025 5.7609 5.7188 5.2907 5.2642 3.8021 3.7223 3.4191 1.7788 1.6633 1.5586 1.0703
4.28 10.30 1.13 1.01 1.00 1.00 1.00 2.13 2.18 5.36 1.01
10.0 9.5 9.0 8.5 8.0 7.5 7.0 6.5 6.0 5.5 5.0 4.5 4.0 3.5 3.0 2.5 2.0 1.5 1.0 0.5 0.0
f1 (ppm)

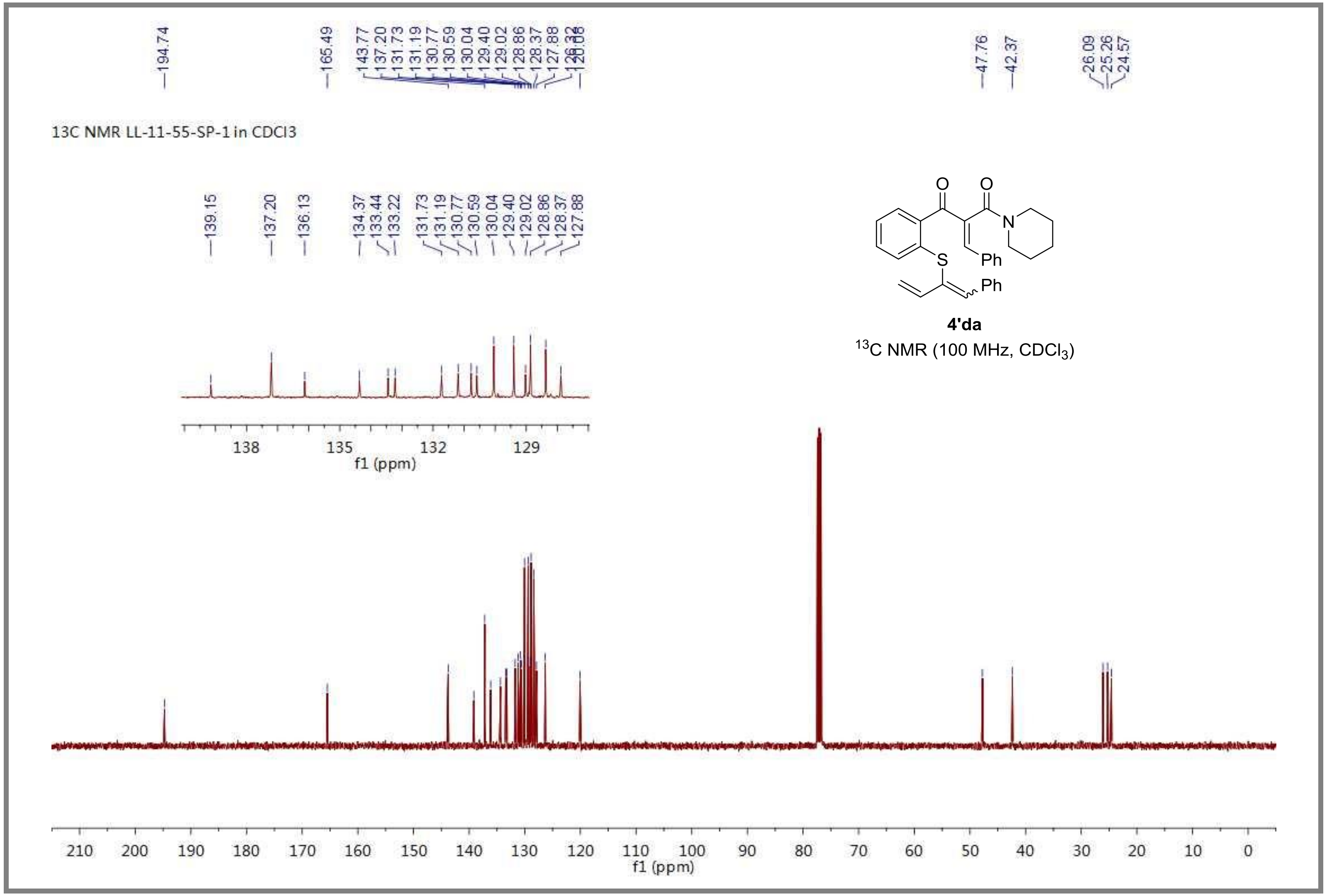
13C NMR LL-11-55-SP-1 in CDCl3
4'da
^{13}C NMR (100 MHz, CDCl$_3$)
194.74
165.49
143.77
137.20
131.73
131.19
130.77
130.59
130.04
129.40
129.02
128.86
128.37
127.88
47.76
42.37
26.09
25.26
24.57
139.15
137.20
136.13
134.37
133.44
133.22
f1 (ppm)

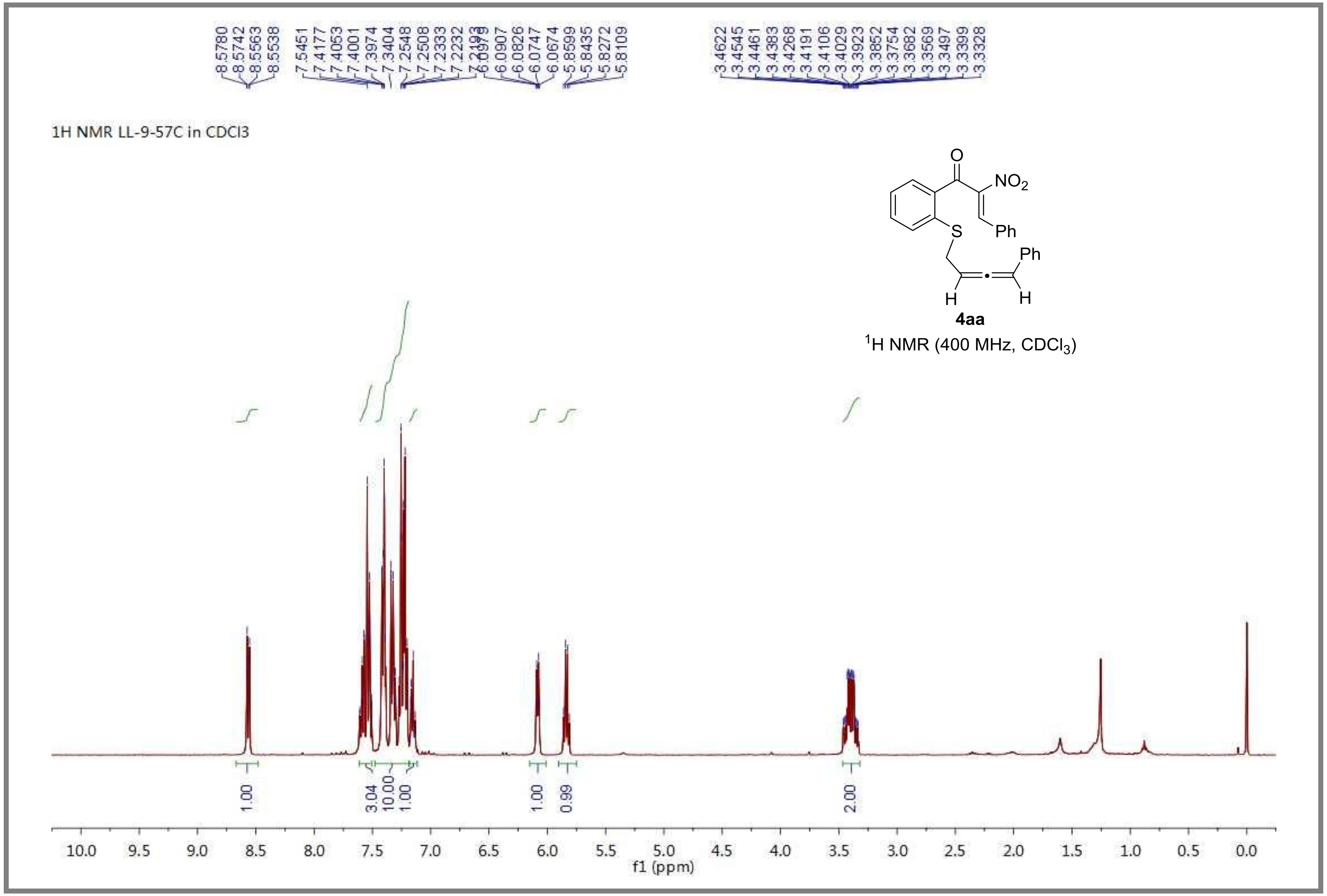
1H NMR LL-9-57C in CDCl3
4aa
^{1}H NMR (400 MHz, $CDCl_3$)
f1 (ppm)

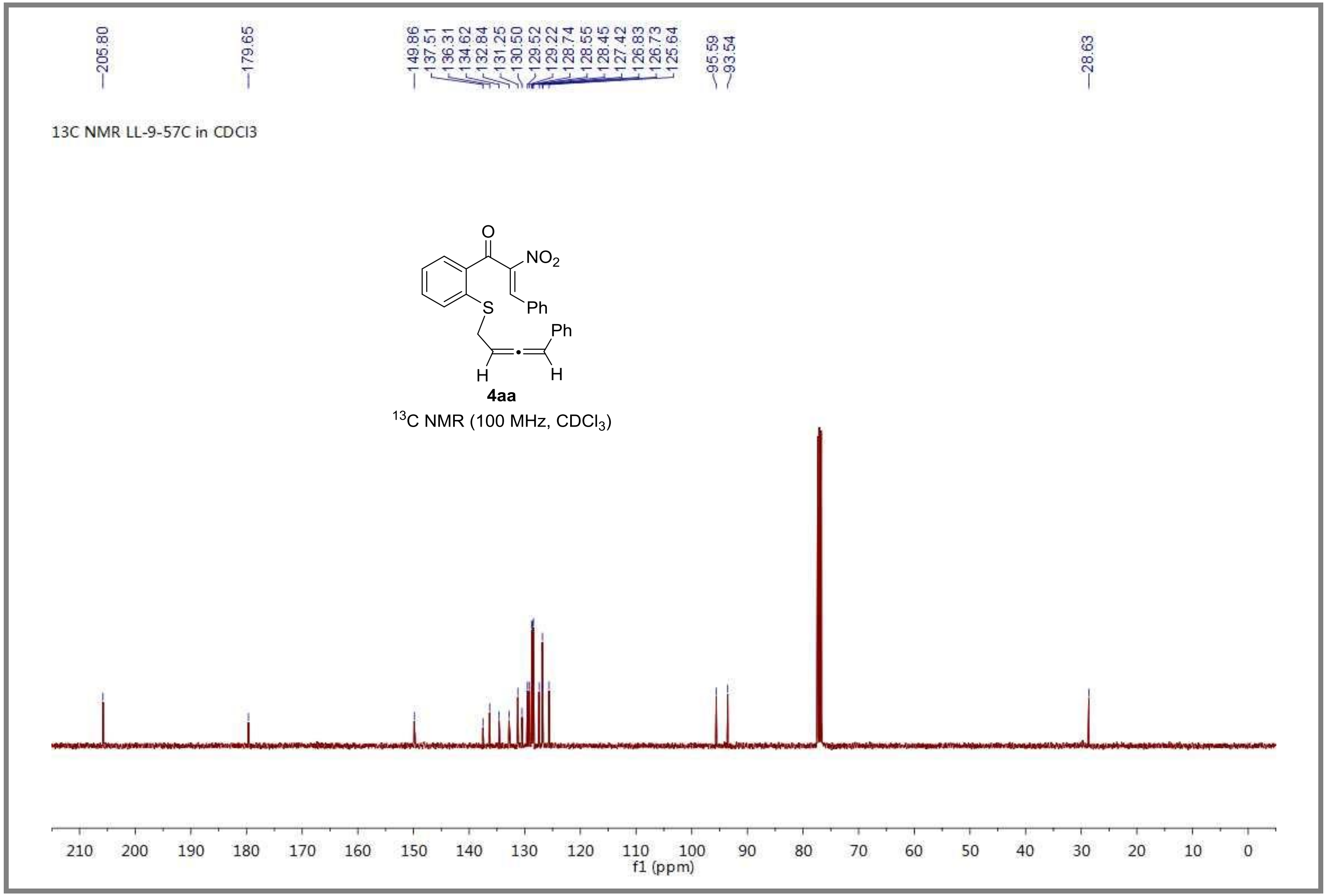

205.80
179.65
149.86
137.51
136.31
134.62
132.84
131.25
130.50
129.52
129.22
128.74
128.55
128.45
127.42
126.83
126.73
125.64
95.59
93.54
28.63
13C NMR LL-9-57C in CDCl3
O
NO_2
Ph
S
Ph
H
H
4aa
^{13}C NMR (100 MHz, $CDCl_3$)
210 200 190 180 170 160 150 140 130 120 110 100 90 80 70 60 50 40 30 20 10 0
f1 (ppm)

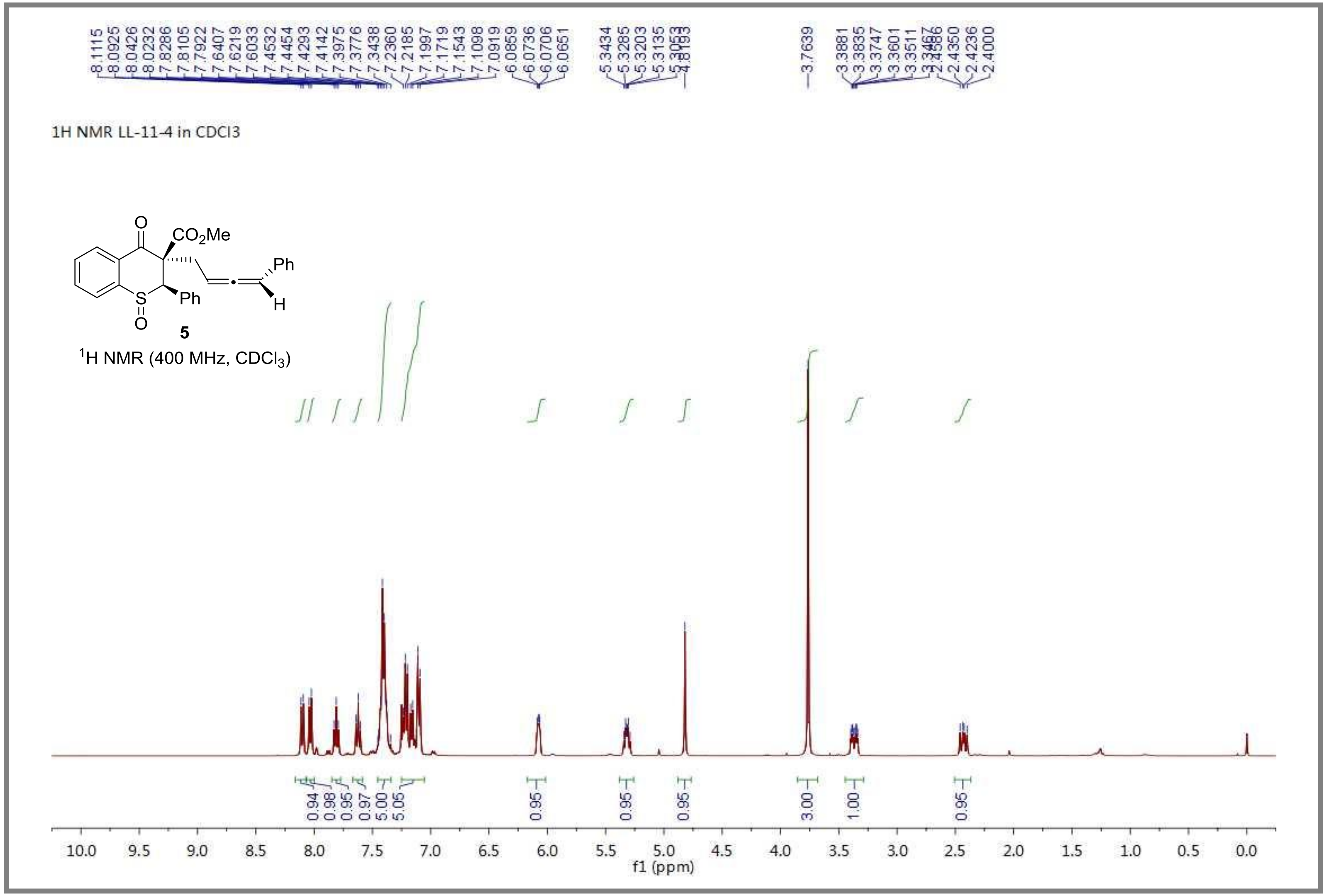
1H NMR LL-11-4 in CDCl3
CO2Me
Ph
Ph
H
S
O
O
5
1H NMR (400 MHz, CDCl3)
f1 (ppm)

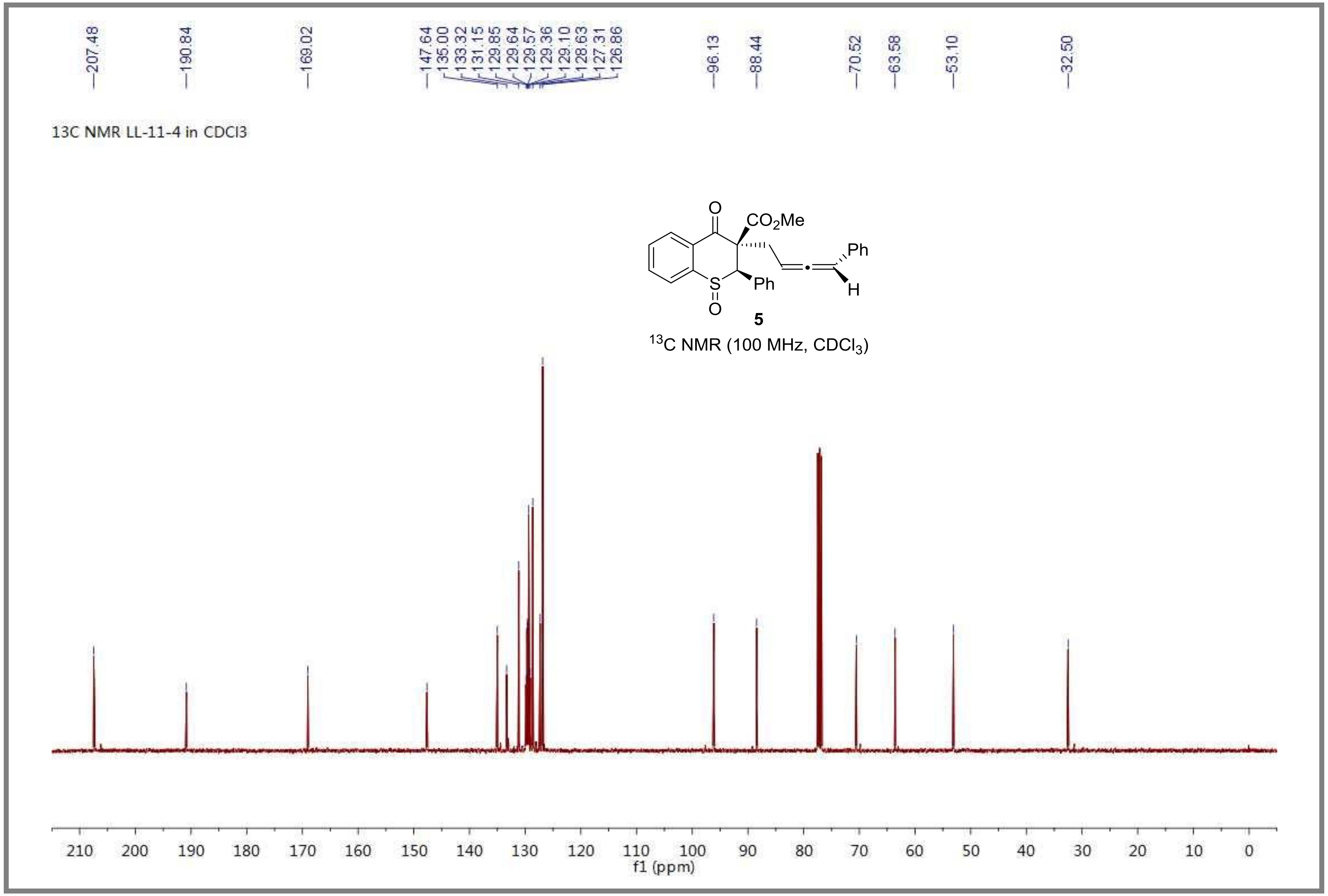
207.48
190.84
169.02
147.64
135.00
133.32
131.15
129.85
129.64
129.57
129.36
129.10
128.63
127.31
126.86
96.13
88.44
70.52
63.58
53.10
32.50
13C NMR LL-11-4 in CDCl3
CO2Me
Ph
Ph
H
O
S
O
5
13C NMR (100 MHz, CDCl3)
210 200 190 180 170 160 150 140 130 120 110 100 90 80 70 60 50 40 30 20 10 0
f1 (ppm)

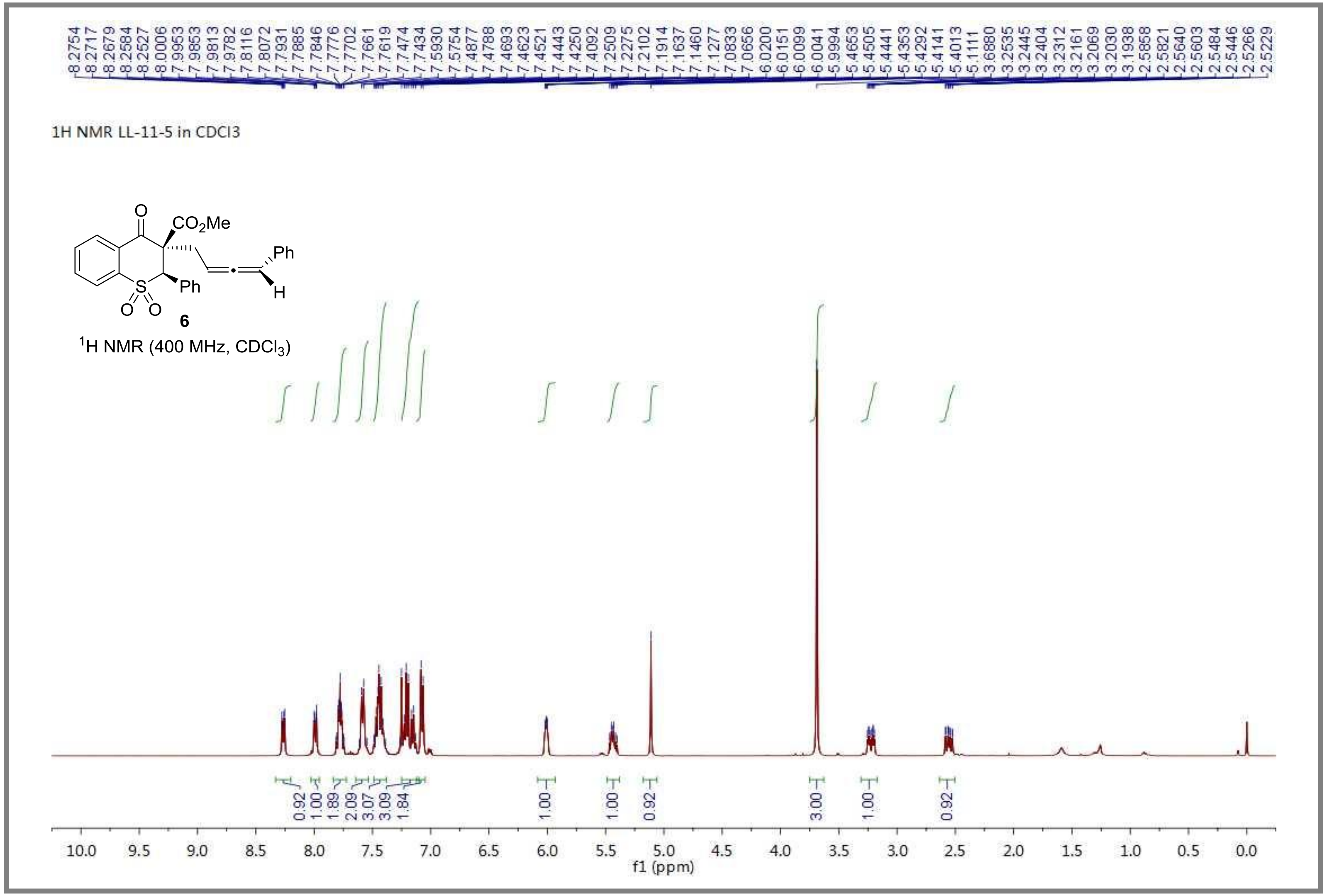
1H NMR LL-11-5 in CDCl3
CO2Me
Ph
Ph
H
6
1H NMR (400 MHz, CDCl3)
f1 (ppm)

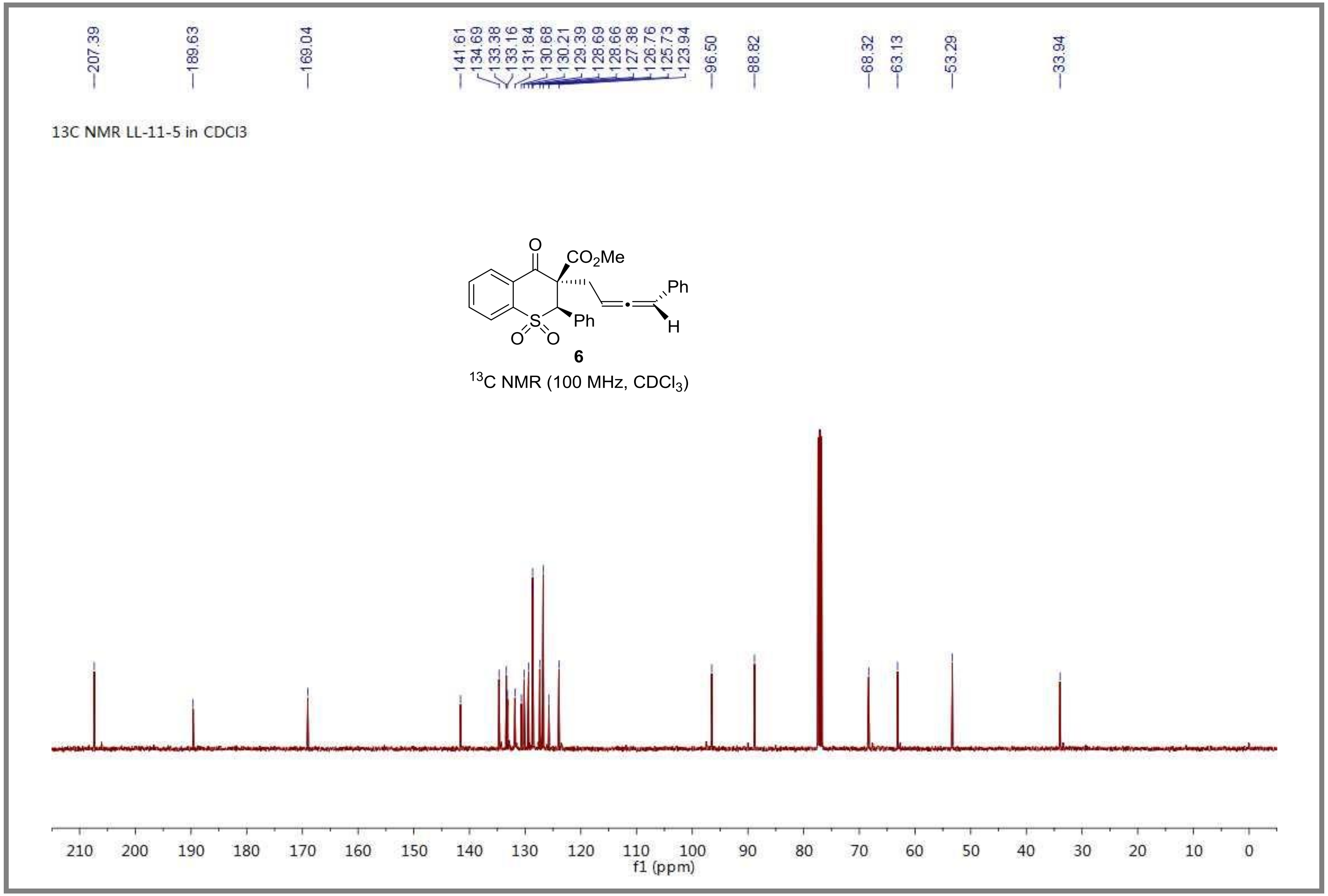
207.39
189.63
169.04
141.61
134.69
133.38
133.16
131.84
130.68
130.21
129.39
128.69
128.66
127.38
126.76
125.73
123.94
96.50
88.82
68.32
63.13
53.29
33.94
13C NMR LL-11-5 in CDCl3
CO2Me
Ph
Ph
H
6
13C NMR (100 MHz, CDCl3)
210 200 190 180 170 160 150 140 130 120 110 100 90 80 70 60 50 40 30 20 10 0
f1 (ppm)

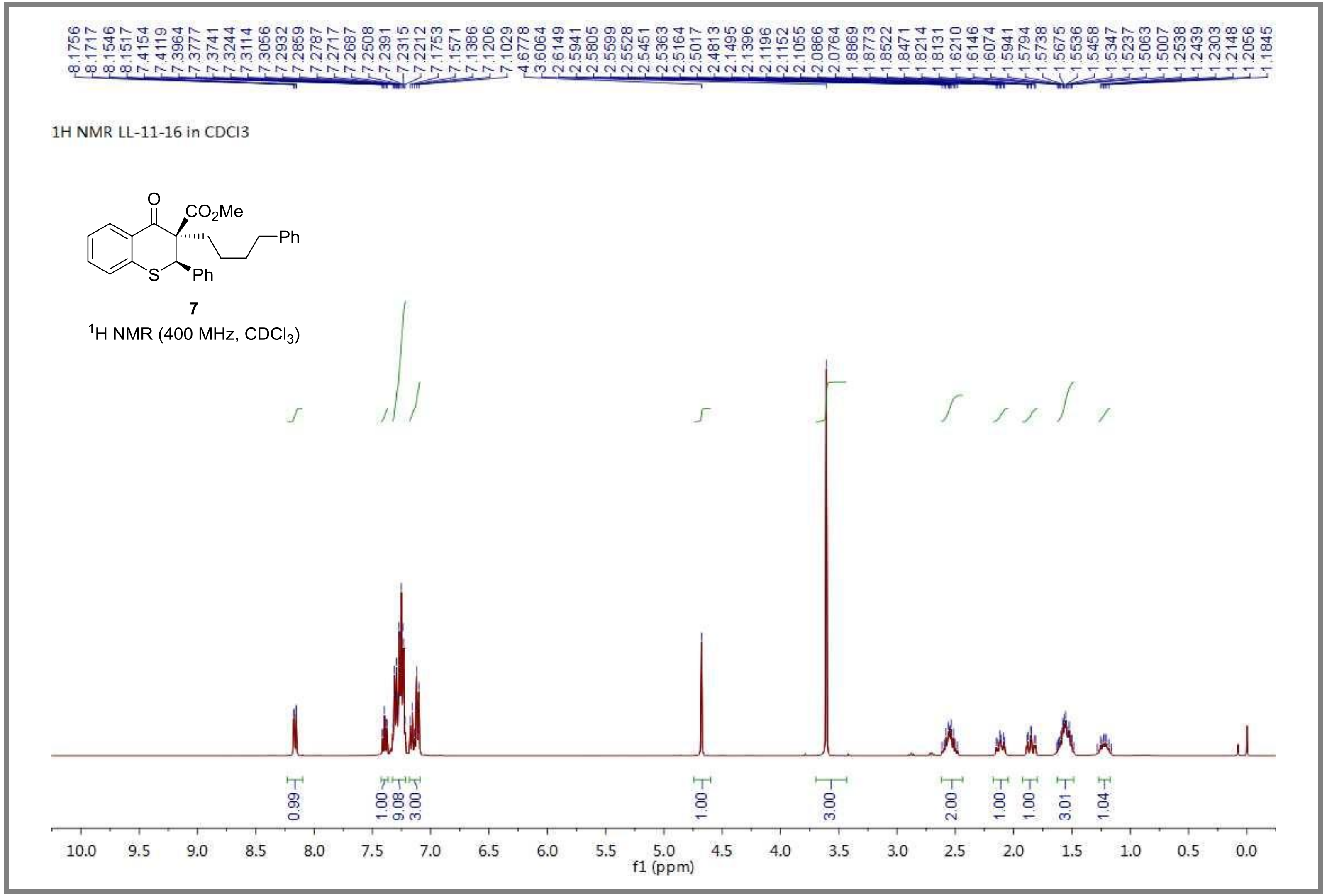
1H NMR LL-11-16 in CDCl3
CO2Me
Ph
S
Ph
O
7
1H NMR (400 MHz, CDCl3)
f1 (ppm)

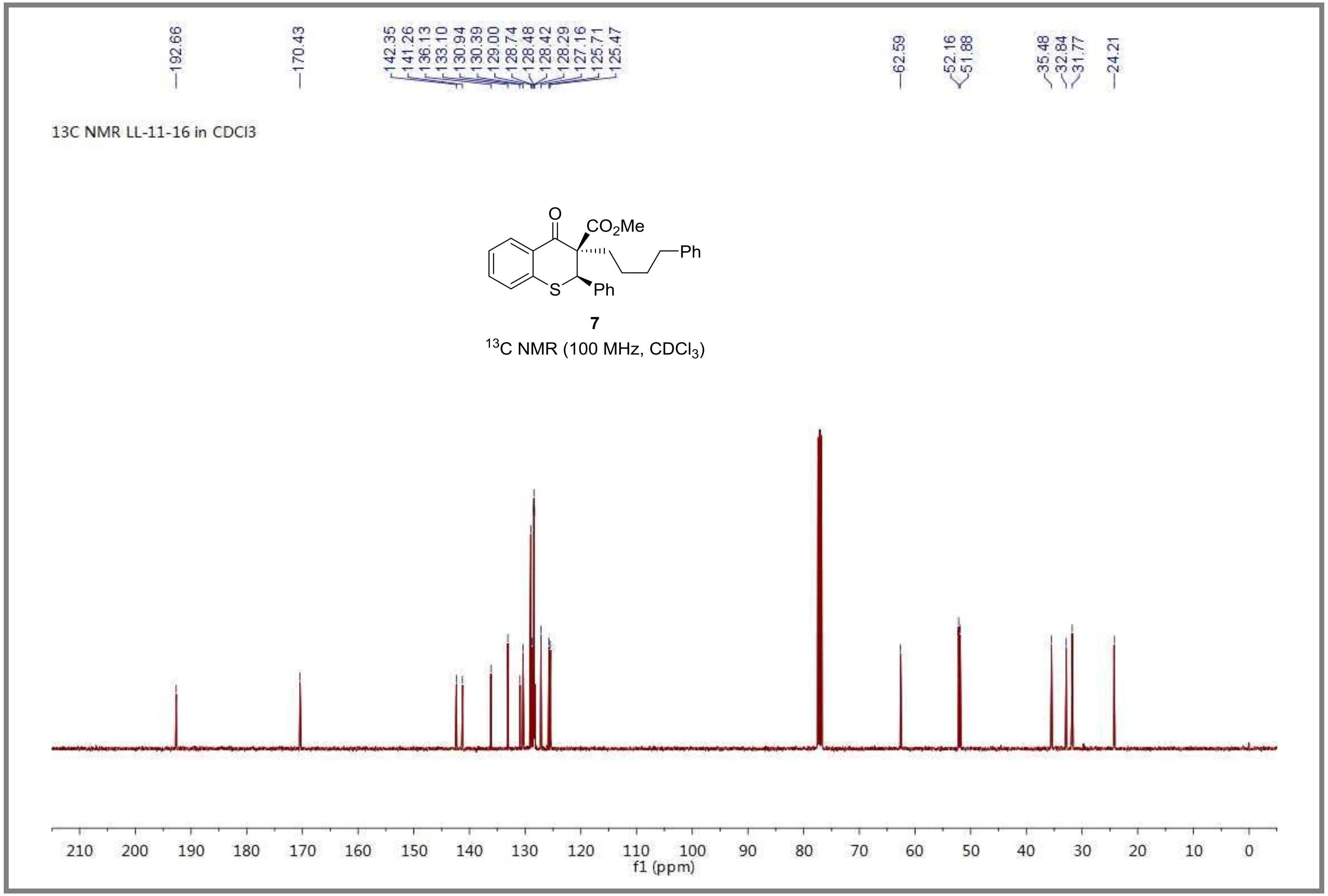

192.66
170.43
142.35
141.26
136.13
133.10
130.94
130.39
129.00
128.74
128.48
128.42
128.29
127.16
125.71
125.47
62.59
52.16
51.88
35.48
32.84
31.77
24.21
13C NMR LL-11-16 in CDCl3
O
CO2Me
Ph
S
Ph
7
13C NMR (100 MHz, CDCl3)
210 200 190 180 170 160 150 140 130 120 110 100 90 80 70 60 50 40 30 20 10 0
f1 (ppm)

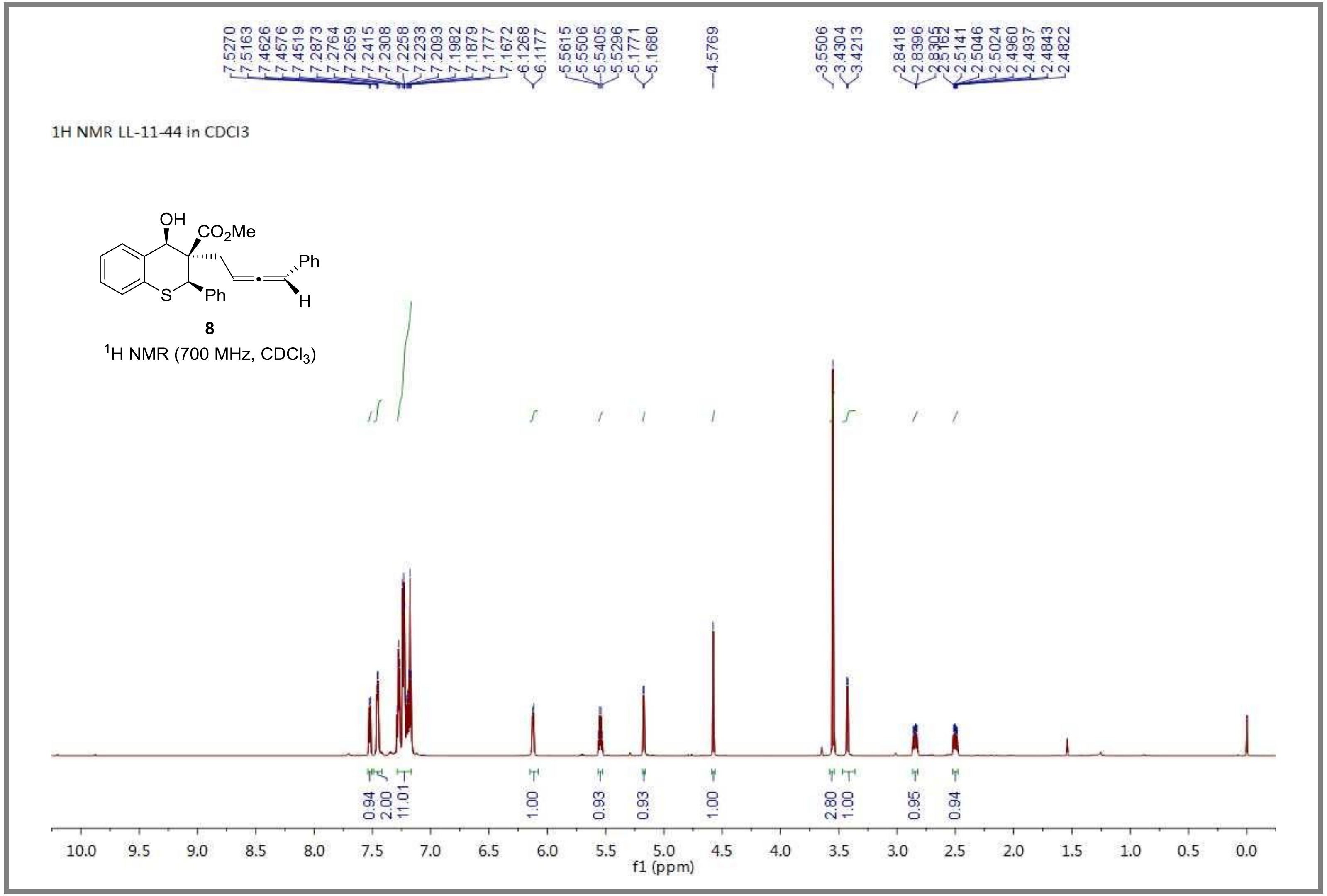
1H NMR LL-11-44 in CDCl3
OH
CO_2Me
Ph
H
S
Ph
8
^1H NMR (700 MHz, $CDCl_3$)
f1 (ppm)

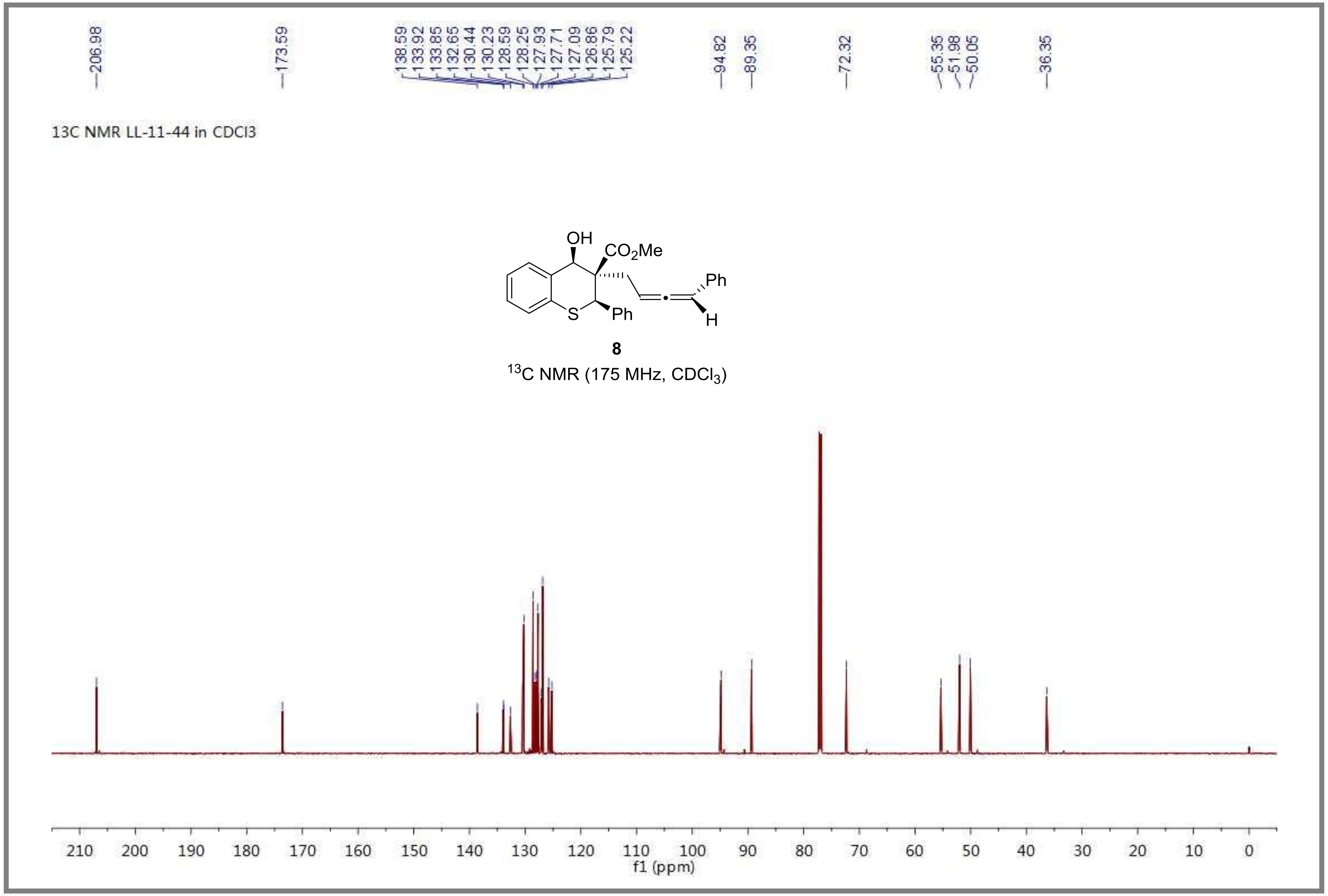

206.98
173.59
138.59
133.92
133.85
132.65
130.44
130.23
128.59
128.25
127.93
127.71
127.09
126.86
125.79
125.22
94.82
89.35
72.32
55.35
51.98
50.05
36.35
13C NMR LL-11-44 in CDCl3
OH
CO2Me
Ph
S
Ph
H
8
13C NMR (175 MHz, CDCl3)
210 200 190 180 170 160 150 140 130 120 110 100 90 80 70 60 50 40 30 20 10 0
f1 (ppm)

Data File C:\CHEM32\1\DATA\ZHOU-22\YZN021306.D
Sample Name: LL-9-96 +/-

Acq. Operator :
Acq. Instrument : Instrument 1 Location : -
Injection Date : 5/31/2022 10:04:59 PM
Acq. Method : C:\CHEM32\1\METHODS\DEF_LC.M
Last changed : 5/31/2022 10:03:56 PM
(modified after loading)
Analysis Method : C:\CHEM32\1\METHODS\DEF_LC.M
Last changed : 6/2/2022 9:05:02 AM
(modified after loading)
Sample Info : IC, Hexane/i-PrOH = 95/5, 1.0 mL/min, 30 oC, 254 nm

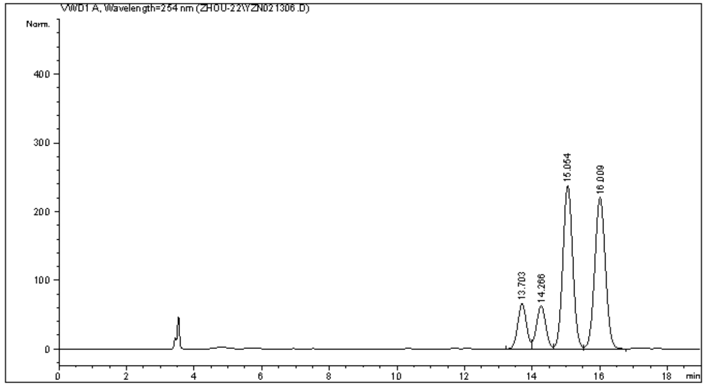

Area Percent Report

Sorted By : Signal
Multiplier: : 1.0000
Dilution: : 1.0000
Use Multiplier & Dilution Factor with ISTDs

Signal 1: VWD1 A, Wavelength=254 nm

Peak #	RetTime [min]	Type	Width [min]	Area mAU *s	Height [mAU]	Area %
1	13.703	BV	0.2796	1181.40186	65.72604	9.8469
2	14.266	VV	0.2934	1184.59485	62.63682	9.8735
3	15.054	VV	0.3161	4809.14453	237.68280	40.0840
4	16.009	VB	0.3413	4822.53174	220.28629	40.1956
Totals :				1.19977e4	586.33195	

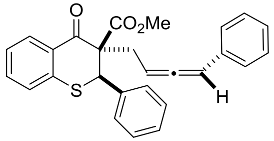

(+/-)-3ca

*** End of Report ***

Instrument 1 6/2/2022 9:05:10 AM Page 1 of 1

Data File C:\CHEM32\1\DATA\ZHOU-22\YZN021305.D
Sample Name: LL-9-96

Acq. Operator :
Acq. Instrument : Instrument 1 Location : -
Injection Date : 5/31/2022 9:44:46 PM
Acq. Method : C:\CHEM32\1\METHODS\DEF_LC.M
Last changed : 5/31/2022 9:30:21 PM
(modified after loading)
Analysis Method : C:\CHEM32\1\METHODS\DEF_LC.M
Last changed : 6/2/2022 9:05:56 AM
(modified after loading)
Sample Info : IC, Hexane/i-PrOH = 95/5, 1.0 mL/min, 30 oC, 254 nm

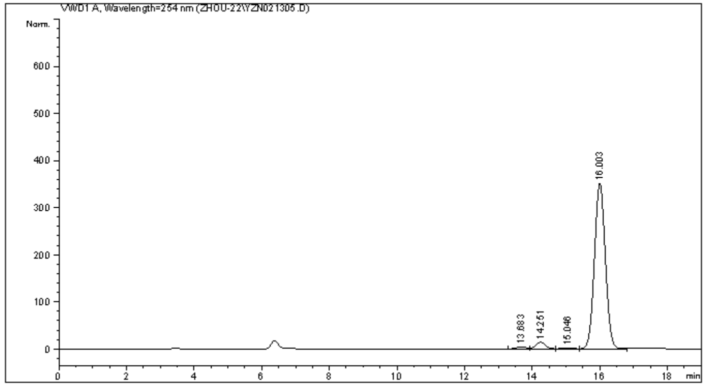

Area Percent Report

Sorted By : Signal
Multiplier: : 1.0000
Dilution: : 1.0000
Use Multiplier & Dilution Factor with ISTDs

Signal 1: VWD1 A, Wavelength=254 nm

Peak #	RetTime [min]	Type	Width [min]	Area mAU *s	Height [mAU]	Area %
1	13.683	BV	0.2830	84.60534	4.63237	1.0251
2	14.251	VV	0.2991	264.45209	13.81320	3.2040
3	15.046	VV	0.2989	37.25450	1.90967	0.4514
4	16.003	VB	0.3487	7867.43896	351.24875	95.3196
Totals :				8253.75089	371.60400	

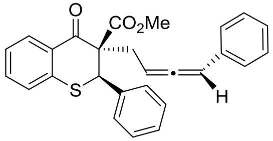

(-)-3ca

*** End of Report ***

Instrument 1 6/2/2022 9:07:21 AM Page 1 of 1

Data File C:\CHEM32\1\DATA\ZHOU-22\YZN021397.D
Sample Name: LL-10-10A +/-

===

Acq. Operator :
Acq. Instrument : Instrument 1 Location : -
Injection Date : 6/16/2022 9:58:20 PM
Acq. Method : C:\CHEM32\1\METHODS\DEF_LC.M
Last changed : 6/16/2022 9:50:17 PM
(modified after loading)
Analysis Method : C:\CHEM32\1\METHODS\DEF_LC.M
Last changed : 6/17/2022 9:57:36 AM
(modified after loading)
Sample Info : ID, Hexane/i-PrOH = 97/3, 1.0 mL/min, 30 oC, 254 nm

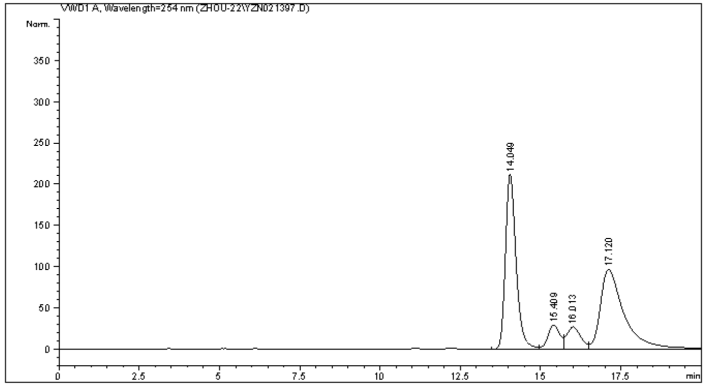

===

Area Percent Report

===

Sorted By : Signal
Multiplier: : 1.0000
Dilution: : 1.0000
Use Multiplier & Dilution Factor with ISTDs

Signal 1: VWD1 A, Wavelength=254 nm

Peak #	RetTime [min]	Type	Width [min]	Area mAU *s	Height [mAU]	Area %
1	14.049	VV	0.3526	4865.26904	211.67204	42.6194
2	15.409	VV	0.3884	746.58508	29.21757	6.5400
3	16.013	VV	0.4594	821.14044	26.80166	7.1931
4	17.120	VBA	0.7535	4982.61914	96.54684	43.6474

Totals : 1.14156e4 364.23812

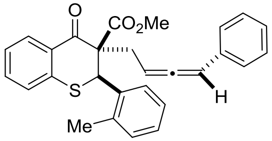

(+/-)-3ea

===

*** End of Report ***

Instrument 1 6/17/2022 9:57:44 AM Page 1 of 1

Data File C:\CHEM32\1\DATA\ZHOU-22\YZN021396.D
Sample Name: LL-10-10A

===

Acq. Operator :
Acq. Instrument : Instrument 1 Location : -
Injection Date : 6/16/2022 9:28:37 PM
Acq. Method : C:\CHEM32\1\METHODS\DEF_LC.M
Last changed : 6/16/2022 9:13:30 PM
(modified after loading)
Analysis Method : C:\CHEM32\1\METHODS\DEF_LC.M
Last changed : 6/17/2022 9:56:51 AM
(modified after loading)
Sample Info : ID, Hexane/i-PrOH = 97/3, 1.0 mL/min, 30 oC, 254 nm

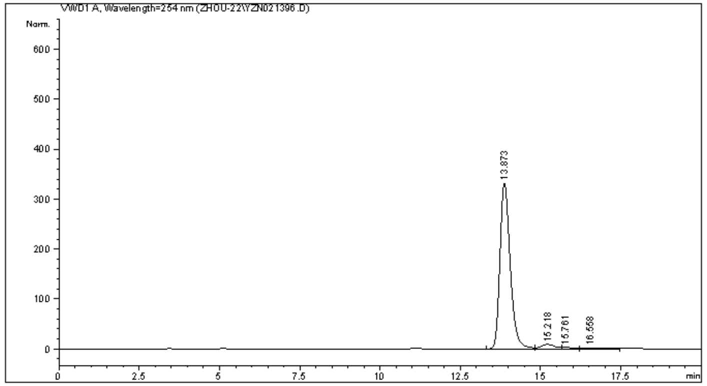

===

Area Percent Report

===

Sorted By : Signal
Multiplier: : 1.0000
Dilution: : 1.0000
Use Multiplier & Dilution Factor with ISTDs

Signal 1: VWD1 A, Wavelength=254 nm

Peak #	RetTime [min]	Type	Width [min]	Area mAU *s	Height [mAU]	Area %
1	13.873	VV	0.3377	7385.88330	332.60446	94.6470
2	15.218	VV	0.4480	263.34531	8.87867	3.3747
3	15.761	VV	0.3517	69.32341	2.84078	0.8884
4	16.558	VV	0.7494	85.05629	1.57499	1.0900

Totals : 7803.60831 345.89890

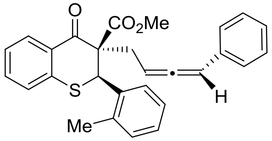

(-)-3ea

===

*** End of Report ***

Instrument 1 6/17/2022 9:57:00 AM Page 1 of 1

Data File C:\CHEM32\1\DATA\ZHOU-22\YZN021399.D
Sample Name: LL-10-10B +/-

===

Acq. Operator :
Acq. Instrument : Instrument 1 Location : -
Injection Date : 6/16/2022 11:12:53 PM
Acq. Method : C:\CHEM32\1\METHODS\DEF_LC.M
Last changed : 6/16/2022 11:11:23 PM
(modified after loading)
Analysis Method : C:\CHEM32\1\METHODS\DEF_LC.M
Last changed : 6/17/2022 10:03:43 AM
(modified after loading)
Sample Info : OD-3, Hexane/i-PrOH = 95/5, 0.8 mL/min, 30 oC, 254 nm

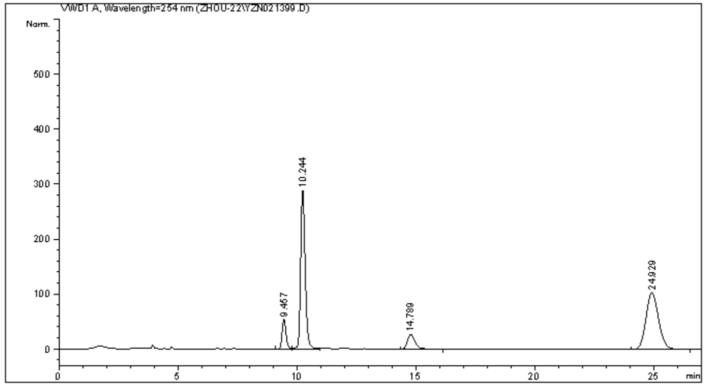

===

Area Percent Report

===

Sorted By : Signal
Multiplier: : 1.0000
Dilution: : 1.0000
Use Multiplier & Dilution Factor with ISTDs

Signal 1: VWD1 A, Wavelength=254 nm

Peak #	RetTime [min]	Type	Width [min]	Area mAU *s	Height [mAU]	Area %
1	9.457	VV	0.1777	618.70984	53.87775	6.9623
2	10.244	VV	0.2054	3865.48975	289.33228	43.4980
3	14.789	VB	0.3386	599.61310	26.90870	6.7474
4	24.929	BB	0.5717	3802.77173	103.42006	42.7923

Totals : 8886.58441 473.53879

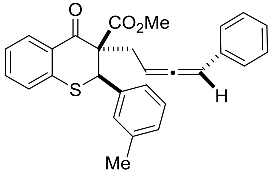

(+/-)-3fa

===

*** End of Report ***

Instrument 1 6/17/2022 10:03:47 AM Page 1 of 1

Data File C:\CHEM32\1\DATA\ZHOU-22\YZN021398.D
Sample Name: LL-10-10B

===

Acq. Operator :
Acq. Instrument : Instrument 1 Location : -
Injection Date : 6/16/2022 10:37:23 PM
Acq. Method : C:\CHEM32\1\METHODS\DEF_LC.M
Last changed : 6/16/2022 10:20:50 PM
(modified after loading)
Analysis Method : C:\CHEM32\1\METHODS\DEF_LC.M
Last changed : 6/17/2022 10:03:08 AM
(modified after loading)
Sample Info : OD-3, Hexane/i-PrOH = 95/5, 0.8 mL/min, 30 oC, 254 nm

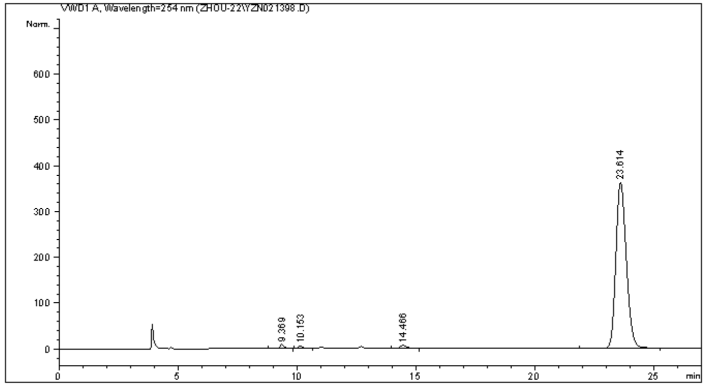

===

Area Percent Report

===

Sorted By : Signal
Multiplier: : 1.0000
Dilution: : 1.0000
Use Multiplier & Dilution Factor with ISTDs

Signal 1: VWD1 A, Wavelength=254 nm

Peak #	RetTime [min]	Type	Width [min]	Area mAU *s	Height [mAU]	Area %
1	9.369	VB	0.1970	126.27138	9.42724	1.0969
2	10.153	BB	0.2182	80.58767	5.57237	0.7000
3	14.466	VB	0.3299	141.23883	6.48234	1.2269
4	23.614	VV	0.4786	1.11640e4	362.64539	96.9763

Totals : 1.15121e4 384.12733

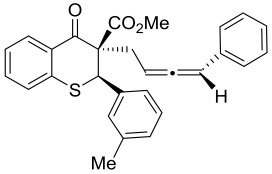

(-)-3fa

===

*** End of Report ***

Instrument 1 6/17/2022 10:03:14 AM Page 1 of 1

Data File C:\CHEM32\1\DATA\ZHOU-22\YZN021401.D
Sample Name: LL-10-10C +/-

Acq. Operator :
Acq. Instrument : Instrument 1 Location : -
Injection Date : 6/17/2022 12:22:05 AM
Acq. Method : C:\CHEM32\1\METHODS\DEF_LC.M
Last changed : 6/17/2022 12:20:56 AM
(modified after loading)
Analysis Method : C:\CHEM32\1\METHODS\DEF_LC.M
Last changed : 6/18/2022 1:56:11 AM
(modified after loading)
Sample Info : IA, Hexane/i-PrOH = 95/5, 1.0 mL/min, 30 oC, 254 nm

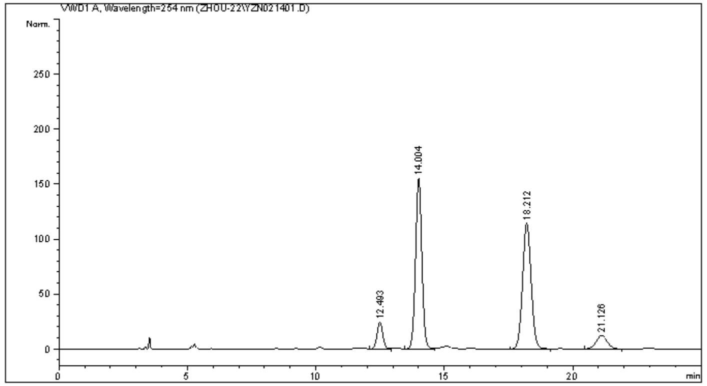

Area Percent Report

Sorted By : Signal
Multiplier: : 1.0000
Dilution: : 1.0000
Use Multiplier & Dilution Factor with ISTDs

Signal 1: VWD1 A, Wavelength=254 nm

Peak #	RetTime [min]	Type	Width [min]	Area mAU *s	Height [mAU]	Area %
1	12.493	VB	0.2410	379.17752	24.40358	6.2095
2	14.004	VB	0.2654	2677.81982	155.15761	43.8525
3	18.212	BB	0.3598	2676.45337	114.61832	43.8301
4	21.126	BB	0.4633	372.97498	12.39067	6.1079

Totals : 6106.42569 306.57018

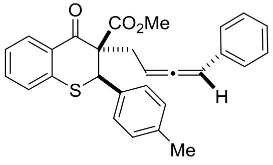

(+/-)-3ga

*** End of Report ***

Instrument 1 6/18/2022 1:56:15 AM Page 1 of 1

Data File C:\CHEM32\1\DATA\ZHOU-22\YZN021407.D
Sample Name: LL-10-10C

Acq. Operator :
Acq. Instrument : Instrument 1 Location : -
Injection Date : 6/17/2022 8:25:19 PM
Acq. Method : C:\CHEM32\1\METHODS\DEF_LC.M
Last changed : 6/17/2022 8:01:10 PM
(modified after loading)
Analysis Method : C:\CHEM32\1\METHODS\DEF_LC.M
Last changed : 6/18/2022 1:55:37 AM
(modified after loading)
Sample Info : IA, Hexane/i-PrOH = 95/5, 1.0 mL/min, 30 oC, 254 nm

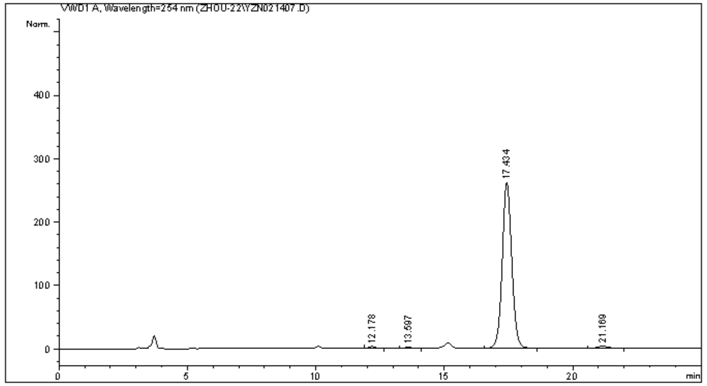

Area Percent Report

Sorted By : Signal
Multiplier: : 1.0000
Dilution: : 1.0000
Use Multiplier & Dilution Factor with ISTDs

Signal 1: VWD1 A, Wavelength=254 nm

Peak #	RetTime [min]	Type	Width [min]	Area mAU *s	Height [mAU]	Area %
1	12.178	VB	0.2871	63.09805	3.25846	0.9013
2	13.597	VB	0.3102	46.35885	2.30663	0.6622
3	17.434	BB	0.3974	6776.02734	262.32910	96.7889
4	21.169	BB	0.4919	115.34654	3.64128	1.6476

Totals : 7000.83079 271.53548

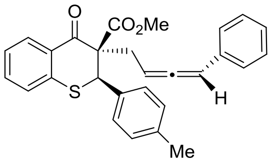

(-)-3ga

*** End of Report ***

Instrument 1 6/18/2022 1:55:48 AM Page 1 of 1

Data File C:\CHEM32\1\DATA\ZHOU-22\YZN021461.D
Sample Name: LL-10-25A +/-

===

Acq. Operator :
Acq. Instrument : Instrument 1 Location : -
Injection Date : 6/30/2022 9:14:24 PM
Acq. Method : C:\CHEM32\1\METHODS\DEF_LC.M
Last changed : 6/30/2022 9:11:25 PM
(modified after loading)
Analysis Method : C:\CHEM32\1\METHODS\DEF_LC.M
Last changed : 7/1/2022 4:41:13 AM
(modified after loading)
Sample Info : OD-H, Hexane/i-PrOH = 95/5, 1.0 mL/min, 30 oC, 254 nm

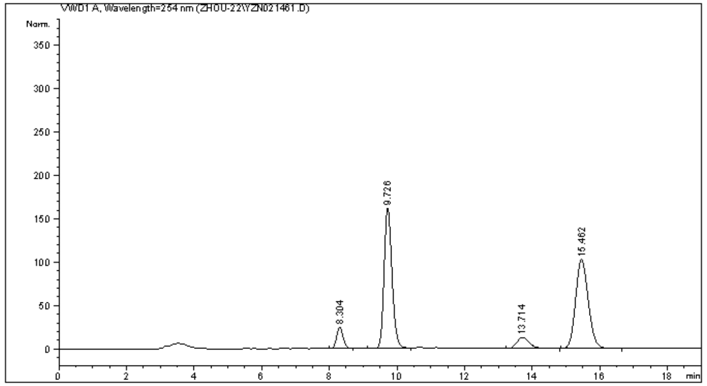

===
Area Percent Report
===

Sorted By : Signal
Multiplier: : 1.0000
Dilution: : 1.0000
Use Multiplier & Dilution Factor with ISTDs

Signal 1: VWD1 A, Wavelength=254 nm

Peak #	RetTime [min]	Type	Width [min]	Area mAU *s	Height [mAU]	Area %
1	8.304	BV	0.2048	332.16351	24.95156	5.5432
2	9.726	VV	0.2535	2674.45972	162.17827	44.6315
3	13.714	BB	0.3957	333.42480	12.85673	5.5642
4	15.462	BB	0.4010	2652.26978	102.46486	44.2612

Totals : 5992.31781 302.45142

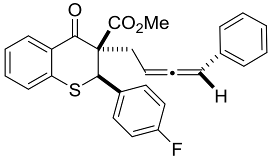

(+/-)-3ha

===
*** End of Report ***

Instrument 1 7/1/2022 4:41:54 AM Page 1 of 1

Data File C:\CHEM32\1\DATA\ZHOU-22\YZN021460.D
Sample Name: LL-10-25A

===

Acq. Operator :
Acq. Instrument : Instrument 1 Location : -
Injection Date : 6/30/2022 8:49:54 PM
Acq. Method : C:\CHEM32\1\METHODS\DEF_LC.M
Last changed : 6/30/2022 7:50:37 PM
(modified after loading)
Analysis Method : C:\CHEM32\1\METHODS\DEF_LC.M
Last changed : 7/1/2022 4:41:13 AM
(modified after loading)
Sample Info : OD-H, Hexane/i-PrOH = 95/5, 1.0 mL/min, 30 oC, 254 nm

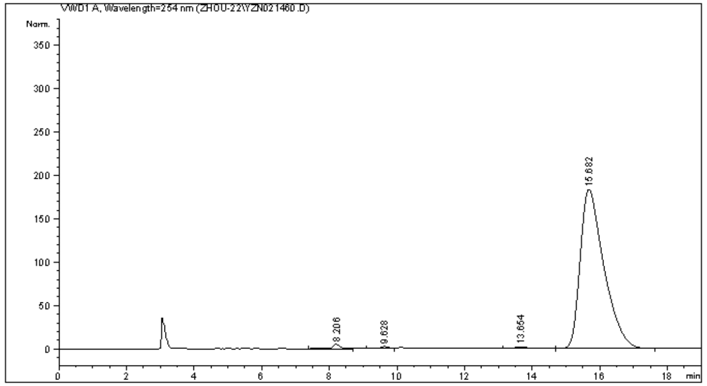

===
Area Percent Report
===

Sorted By : Signal
Multiplier: : 1.0000
Dilution: : 1.0000
Use Multiplier & Dilution Factor with ISTDs

Signal 1: VWD1 A, Wavelength=254 nm

Peak #	RetTime [min]	Type	Width [min]	Area mAU *s	Height [mAU]	Area %
1	8.206	BB	0.2224	74.65000	5.03600	0.8207
2	9.628	BV	0.2763	42.77714	2.35195	0.4703
3	13.654	BV	0.4673	52.19173	1.65324	0.5738
4	15.682	VB	0.7245	8926.16309	183.59103	98.1352

Totals : 9095.78195 192.63221

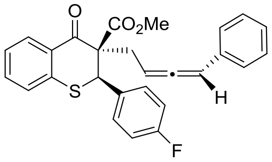

(-)-3ha

===
*** End of Report ***

Instrument 1 7/1/2022 4:41:24 AM Page 1 of 1

Data File C:\CHEM32\1\DATA\ZHOU-22\YZN021463.D
Sample Name: LL-10-25B +/-

===

Acq. Operator :
Acq. Instrument : Instrument 1 Location : -
Injection Date : 6/30/2022 10:00:41 PM
Acq. Method : C:\CHEM32\1\METHODS\DEF_LC.M
Last changed : 6/30/2022 9:58:43 PM
(modified after loading)
Analysis Method : C:\CHEM32\1\METHODS\DEF_LC.M
Last changed : 7/1/2022 4:46:27 AM
(modified after loading)
Sample Info : OD-H, Hexane/i-PrOH = 95/5, 1.0 mL/min, 30 oC, 254 nm

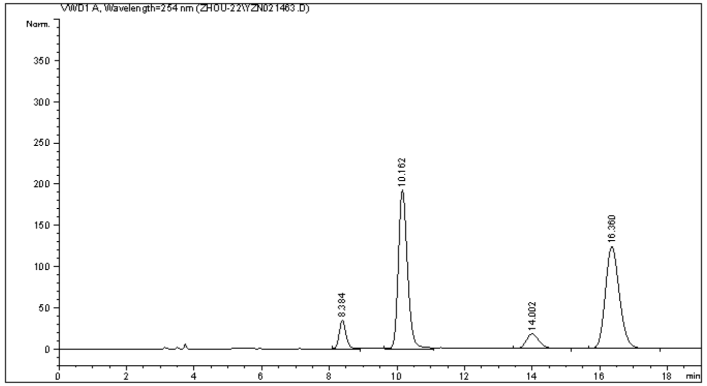

===
Area Percent Report
===

Sorted By : Signal
Multiplier: : 1.0000
Dilution: : 1.0000
Use Multiplier & Dilution Factor with ISTDs

Signal 1: VWD1 A, Wavelength=254 nm

Peak #	RetTime [min]	Type	Width [min]	Area mAU *s	Height [mAU]	Area %
1	8.384	BV	0.2152	482.19040	34.56851	5.9241
2	10.162	VV	0.2885	3604.76440	192.39569	44.2875
3	14.002	BB	0.4158	484.23416	17.99613	5.9492
4	16.360	BB	0.4444	3568.27002	123.64312	43.8392

Totals : 8139.45898 368.60345

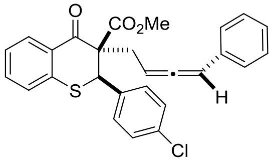

(+/-)-3ia

===
*** End of Report ***

Instrument 1 7/1/2022 4:46:30 AM Page 1 of 1

Data File C:\CHEM32\1\DATA\ZHOU-22\YZN021462.D
Sample Name: LL-10-25B

===

Acq. Operator :
Acq. Instrument : Instrument 1 Location : -
Injection Date : 6/30/2022 9:38:03 PM
Acq. Method : C:\CHEM32\1\METHODS\DEF_LC.M
Last changed : 6/30/2022 9:33:48 PM
(modified after loading)
Analysis Method : C:\CHEM32\1\METHODS\DEF_LC.M
Last changed : 7/1/2022 4:46:02 AM
(modified after loading)
Sample Info : OD-H, Hexane/i-PrOH = 95/5, 1.0 mL/min, 30 oC, 254 nm

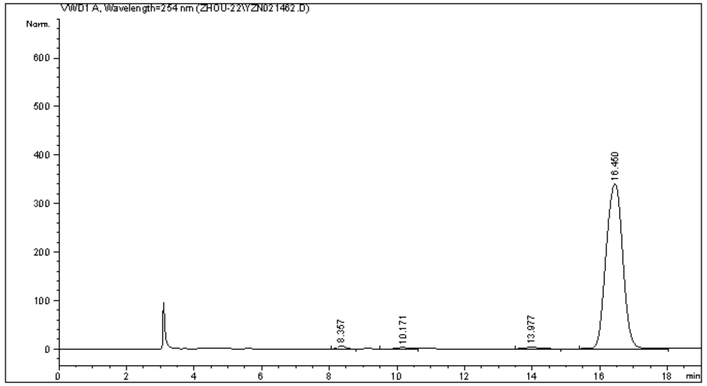

===
Area Percent Report
===

Sorted By : Signal
Multiplier: : 1.0000
Dilution: : 1.0000
Use Multiplier & Dilution Factor with ISTDs

Signal 1: VWD1 A, Wavelength=254 nm

Peak #	RetTime [min]	Type	Width [min]	Area mAU *s	Height [mAU]	Area %
1	8.357	BV	0.2143	91.02782	6.55875	0.7722
2	10.171	VV	0.3167	58.77496	2.77823	0.4986
3	13.977	BB	0.4041	86.74761	3.28612	0.7359
4	16.450	BB	0.5535	1.15518e4	340.12857	97.9934

Totals : 1.17884e4 352.75168

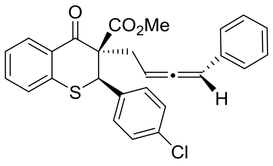

(-)-3ia

===
*** End of Report ***

Instrument 1 7/1/2022 4:46:06 AM Page 1 of 1

Data File C:\CHEM32\1\DATA\ZHOU-22\YZN021465.D
Sample Name: LL-10-25C +/-

===

Acq. Operator :
Acq. Instrument : Instrument 1 Location : -
Injection Date : 6/30/2022 10:53:03 PM
Acq. Method : C:\CHEM32\1\METHODS\DEF_LC.M
Last changed : 6/30/2022 10:51:05 PM
(modified after loading)
Analysis Method : C:\CHEM32\1\METHODS\DEF_LC.M
Last changed : 7/1/2022 4:50:40 AM
(modified after loading)
Sample Info : OD-H, Hexane/i-PrOH = 95/5, 1.0 mL/min, 30 oC, 254 nm

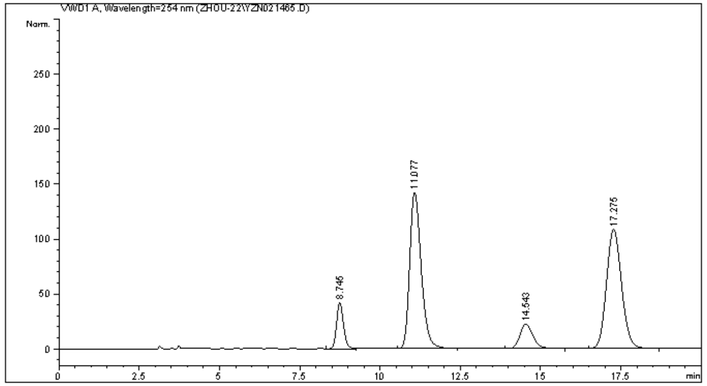

===
Area Percent Report
===

Sorted By : Signal
Multiplier: : 1.0000
Dilution: : 1.0000
Use Multiplier & Dilution Factor with ISTDs

Signal 1: VWD1 A, Wavelength=254 nm

Peak #	RetTime [min]	Type	Width [min]	Area mAU *s	Height [mAU]	Area %
1	8.745	VV	0.2287	625.18262	42.06327	7.5051
2	11.077	VB	0.3851	3562.41309	142.35739	42.7654
3	14.543	VB	0.4303	623.19031	22.33843	7.4812
4	17.275	BB	0.5038	3519.34717	108.44839	42.2484

Totals : 8330.13318 315.20749

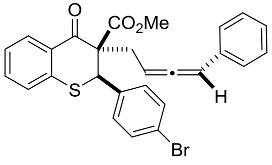

(+/-)-3ja

===
*** End of Report ***

Instrument 1 7/1/2022 4:51:05 AM Page 1 of 1

Data File C:\CHEM32\1\DATA\ZHOU-22\YZN021464.D
Sample Name: LL-10-25C

===

Acq. Operator :
Acq. Instrument : Instrument 1 Location : -
Injection Date : 6/30/2022 10:29:22 PM
Acq. Method : C:\CHEM32\1\METHODS\DEF_LC.M
Last changed : 6/30/2022 10:23:14 PM
(modified after loading)
Analysis Method : C:\CHEM32\1\METHODS\DEF_LC.M
Last changed : 7/1/2022 4:50:40 AM
(modified after loading)
Sample Info : OD-H, Hexane/i-PrOH = 95/5, 1.0 mL/min, 30 oC, 254 nm

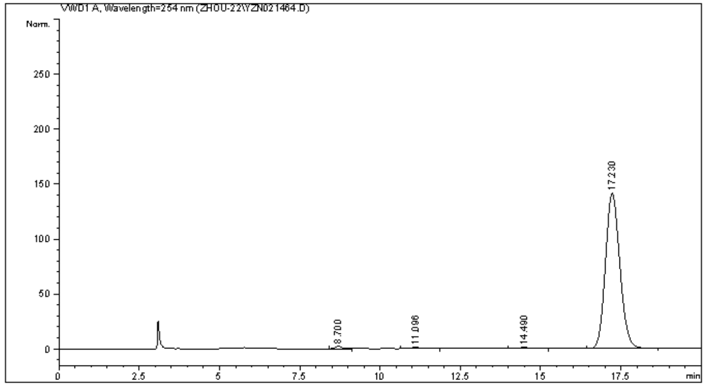

===
Area Percent Report
===

Sorted By : Signal
Multiplier: : 1.0000
Dilution: : 1.0000
Use Multiplier & Dilution Factor with ISTDs

Signal 1: VWD1 A, Wavelength=254 nm

Peak #	RetTime [min]	Type	Width [min]	Area mAU *s	Height [mAU]	Area %
1	8.700	BB	0.2285	32.55314	2.19361	0.7245
2	11.096	BB	0.3843	27.47681	1.09014	0.6115
3	14.490	BB	0.4159	29.13189	1.04390	0.6483
4	17.230	BB	0.4828	4404.18799	141.39259	98.0157

Totals : 4493.34983 145.72023

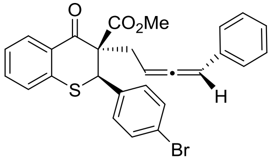

(-)-3ja

===
*** End of Report ***

Instrument 1 7/1/2022 4:50:46 AM Page 1 of 1

Data File C:\CHEM32\1\DATA\ZHOU-22\YZN021508.D
Sample Name: LL-10-41A +/-

===

Acq. Operator :
Acq. Instrument : Instrument 1 Location : -
Injection Date : 7/12/2022 9:34:30 PM
Acq. Method : C:\CHEM32\1\METHODS\DEF_LC.M
Last changed : 7/12/2022 9:33:12 PM
(modified after loading)
Analysis Method : C:\CHEM32\1\METHODS\DEF_LC.M
Last changed : 7/13/2022 9:54:45 AM
(modified after loading)
Sample Info : OD-H, Hexane/i-PrOH = 94/6, 1.0 mL/min, 30 oC, 254 nm

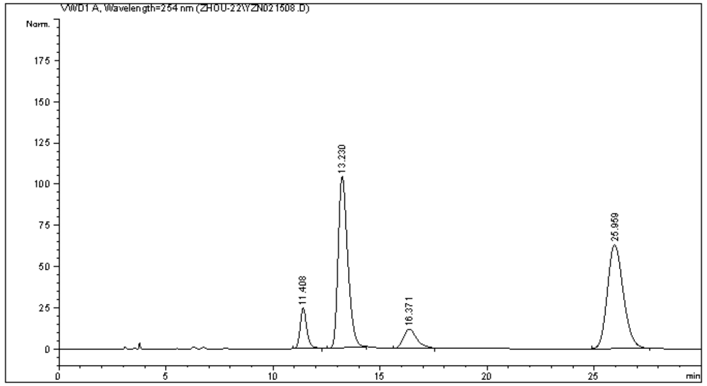

===
Area Percent Report
===

Sorted By : Signal
Multiplier: : 1.0000
Dilution: : 1.0000
Use Multiplier & Dilution Factor with ISTDs

Signal 1: VWD1 A, Wavelength=254 nm

Peak #	RetTime [min]	Type	Width [min]	Area mAU *s	Height [mAU]	Area %
1	11.408	BB	0.3305	528.32330	24.47416	7.0115
2	13.230	BB	0.4760	3226.41968	103.85638	42.8184
3	16.371	BB	0.6728	510.24002	11.64561	6.7715
4	25.959	BB	0.8072	3270.14917	63.07744	43.3987

Totals : 7535.13217 203.05359

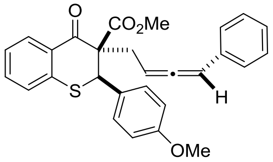

(+/-)-3ka

===
*** End of Report ***

Instrument 1 7/13/2022 9:54:47 AM Page 1 of 1

Data File C:\CHEM32\1\DATA\ZHOU-22\YZN021507.D
Sample Name: LL-10-41A

===

Acq. Operator :
Acq. Instrument : Instrument 1 Location : -
Injection Date : 7/12/2022 9:00:46 PM
Acq. Method : C:\CHEM32\1\METHODS\DEF_LC.M
Last changed : 7/12/2022 8:48:05 PM
(modified after loading)
Analysis Method : C:\CHEM32\1\METHODS\DEF_LC.M
Last changed : 7/12/2022 10:01:46 PM
(modified after loading)
Sample Info : OD-H, Hexane/i-PrOH = 94/6, 1.0 mL/min, 30 oC, 254 nm

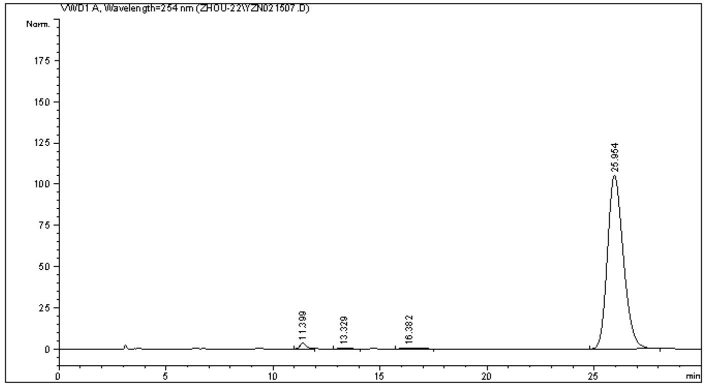

===
Area Percent Report
===

Sorted By : Signal
Multiplier: : 1.0000
Dilution: : 1.0000
Use Multiplier & Dilution Factor with ISTDs

Signal 1: VWD1 A, Wavelength=254 nm

Peak #	RetTime [min]	Type	Width [min]	Area mAU *s	Height [mAU]	Area %
1	11.399	BB	0.3295	71.07248	3.38412	1.2782
2	13.329	BB	0.4364	16.56297	5.33641e-1	0.2979
3	16.382	BB	0.6242	31.66577	6.64726e-1	0.5695
4	25.954	BB	0.8004	5441.19580	105.13624	97.8545

Totals : 5560.49702 109.71872

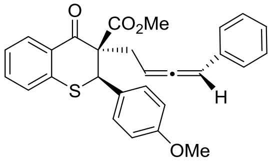

(-)-3ka

===
*** End of Report ***

Instrument 1 7/12/2022 10:01:49 PM Page 1 of 1

Data File C:\CHEM32\1\DATA\ZHOU-22\YZN021580.D
Sample Name: LL-10-53D +/-

===

Acq. Operator :
Acq. Instrument : Instrument 1   Location : -
Injection Date : 7/27/2022 8:40:40 AM
Acq. Method : C:\CHEM32\1\METHODS\DEF_LC.M
Last changed : 7/27/2022 8:37:30 AM
(modified after loading)
Analysis Method : C:\CHEM32\1\METHODS\DEF_LC.M
Last changed : 7/27/2022 9:14:13 AM
(modified after loading)
Sample Info : IC, Hexane/i-PrOH = 99/1, 1.0 mL/min, 30 oC, 254 nm

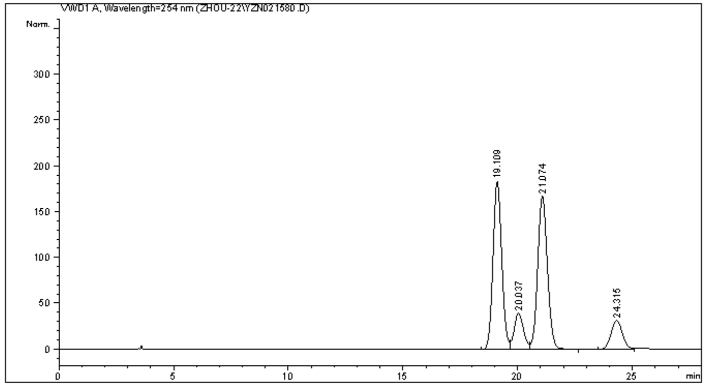

===
Area Percent Report
===

Sorted By : Signal
Multiplier: : 1.0000
Dilution: : 1.0000
Use Multiplier & Dilution Factor with ISTDs

Signal 1: VWD1 A, Wavelength=254 nm

Peak #	RetTime [min]	Type	Width [min]	Area mAU *s	Height [mAU]	Area %
1	19.109	BV	0.4115	4856.06885	183.02740	40.6086
2	20.037	VV	0.4314	1103.95667	39.26189	9.2318
3	21.074	VB	0.4557	4915.48437	166.89432	41.1054
4	24.315	BV	0.5321	1082.72046	31.49474	9.0542

Totals : 1.19582e4 420.67836

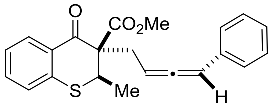

(+/-)-3la

===
*** End of Report ***

Instrument 1 7/27/2022 9:14:58 AM   Page 1 of 1

Data File C:\CHEM32\1\DATA\ZHOU-22\YZN021576.D
Sample Name: LL-10-53D

===

Acq. Operator :
Acq. Instrument : Instrument 1   Location : -
Injection Date : 7/26/2022 9:00:16 PM
Acq. Method : C:\CHEM32\1\METHODS\DEF_LC.M
Last changed : 7/26/2022 8:19:57 PM
(modified after loading)
Analysis Method : C:\CHEM32\1\METHODS\DEF_LC.M
Last changed : 7/27/2022 9:09:48 AM
(modified after loading)
Sample Info : IC, Hexane/i-PrOH = 99/1, 1.0 mL/min, 30 oC, 254 nm

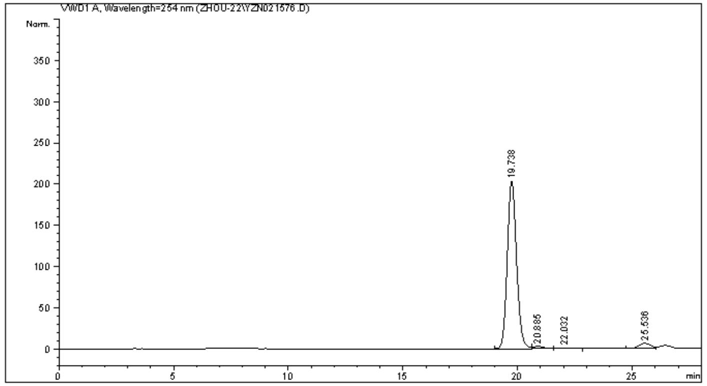

===
Area Percent Report
===

Sorted By : Signal
Multiplier: : 1.0000
Dilution: : 1.0000
Use Multiplier & Dilution Factor with ISTDs

Signal 1: VWD1 A, Wavelength=254 nm

Peak #	RetTime [min]	Type	Width [min]	Area mAU *s	Height [mAU]	Area %
1	19.738	BV	0.4350	5717.60791	202.95280	93.9247
2	20.885	VV	0.4792	92.30535	2.80027	1.5163
3	22.032	VB	0.4718	29.17776	8.37778e-1	0.4793
4	25.536	BV	0.5708	248.34492	6.65473	4.0796

Totals : 6087.43595 213.24558

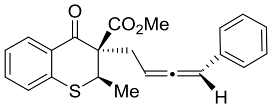

(-)-3la

===
*** End of Report ***

Instrument 1 7/27/2022 9:09:58 AM   Page 1 of 1

Data File C:\CHEM32\1\DATA\ZHOU-22\YZN021627.D
Sample Name: LL-10-63B +/-

===

Acq. Operator :
Acq. Instrument : Instrument 1 Location : -
Injection Date : 8/25/2022 9:23:11 PM
Acq. Method : C:\CHEM32\1\METHODS\DEF_LC.M
Last changed : 8/25/2022 9:21:55 PM
(modified after loading)
Analysis Method : C:\CHEM32\1\METHODS\DEF_LC.M
Last changed : 8/26/2022 9:08:17 PM
(modified after loading)
Sample Info : IB, Hexane/i-PrOH = 85/15, 1.0 mL/min, 30 oC, 254 nm

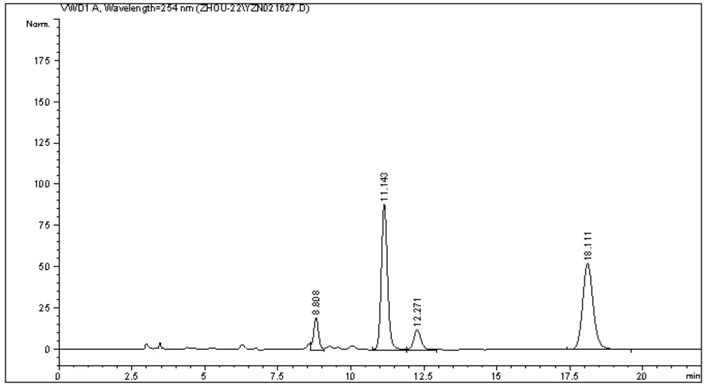

===
Area Percent Report
===

Sorted By : Signal
Multiplier: : 1.0000
Dilution: : 1.0000
Use Multiplier & Dilution Factor with ISTDs

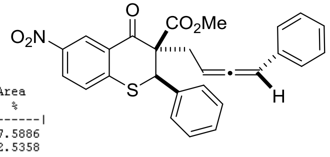

(+/-)-3ma

Signal 1: VWD1 A, Wavelength=254 nm

Peak #	RetTime [min]	Type	Width [min]	Area mAU *s	Height [mAU]	Area %
1	8.808	VV	0.1801	232.57033	19.67810	7.5886
2	11.143	BV	0.2279	1303.60742	88.15347	42.5358
3	12.271	VB	0.2674	212.96240	12.21845	6.9488
4	18.111	VB	0.3880	1315.58972	52.06441	42.9268

Totals : 3064.72987 172.11444

===
*** End of Report ***

Instrument 1 8/26/2022 9:08:21 PM Page 1 of 1

Data File C:\CHEM32\1\DATA\ZHOU-22\YZN021628.D
Sample Name: LL-10-63B

===

Acq. Operator :
Acq. Instrument : Instrument 1 Location : -
Injection Date : 8/25/2022 9:48:11 PM
Acq. Method : C:\CHEM32\1\METHODS\DEF_LC.M
Last changed : 8/25/2022 9:46:45 PM
(modified after loading)
Analysis Method : C:\CHEM32\1\METHODS\DEF_LC.M
Last changed : 8/26/2022 8:57:54 PM
(modified after loading)
Sample Info : IB, Hexane/i-PrOH = 85/15, 1.0 mL/min, 30 oC, 254 nm

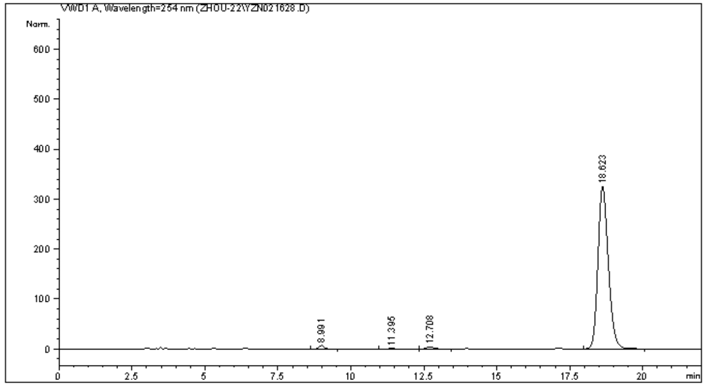

===
Area Percent Report
===

Sorted By : Signal
Multiplier: : 1.0000
Dilution: : 1.0000
Use Multiplier & Dilution Factor with ISTDs

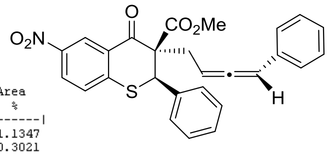

(-)-3ma

Signal 1: VWD1 A, Wavelength=254 nm

Peak #	RetTime [min]	Type	Width [min]	Area mAU *s	Height [mAU]	Area %
1	8.991	BV	0.1997	93.60039	6.99972	1.1347
2	11.395	VB	0.3694	24.92332	9.25774e-1	0.3021
3	12.708	BB	0.2929	83.31068	4.24815	1.0100
4	18.623	BB	0.3821	8047.06543	324.92776	97.5532

Totals : 8248.89981 337.10141

===
*** End of Report ***

Instrument 1 8/26/2022 8:58:03 PM Page 1 of 1

Data File C:\CHEM32\1\DATA\ZHOU-22\YZN021616.D
Sample Name: LL-10-63C +/-

===

Acq. Operator   :
Acq. Instrument : Instrument 1   Location :   -
Injection Date : 8/23/2022 2:03:58 AM
Acq. Method   : C:\CHEM32\1\METHODS\DEF_LC.M
Last changed   : 8/23/2022 1:49:44 AM
                  (modified after loading)
Analysis Method : C:\CHEM32\1\METHODS\DEF_LC.M
Last changed   : 8/26/2022 9:41:43 PM
                  (modified after loading)
Sample Info   : AD-3, Hexane/i-PrOH = 90/10, 0.8 mL/min, 30 oC, 254 nm

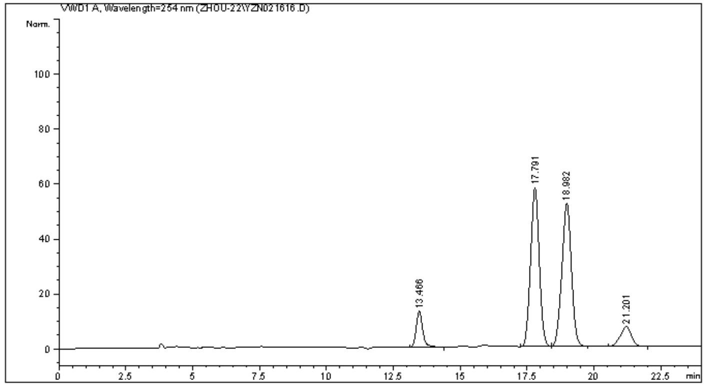

===
Area Percent Report
===

Sorted By   :   Signal
Multiplier: :   1.0000
Dilution: :   1.0000
Use Multiplier & Dilution Factor with ISTDs

Signal 1: VWD1 A, Wavelength=254 nm

Peak #	RetTime [min]	Type	Width [min]	Area mAU *s	Height [mAU]	Area %
1	13.466	BB	0.2546	218.44931	13.07639	7.2224
2	17.791	BV	0.3474	1303.31104	57.82098	43.0903
3	18.982	VB	0.3782	1296.90649	52.01914	42.8785
4	21.201	BB	0.4330	205.93846	7.19370	6.8088

Totals :   3024.60530   130.11022

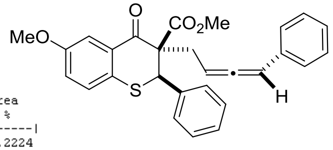

(+/-)-3na

===
*** End of Report ***

Instrument 1 8/26/2022 9:41:47 PM   Page 1 of 1

Data File C:\CHEM32\1\DATA\ZHOU-22\YZN021631.D
Sample Name: LL-10-63C

===

Acq. Operator   :
Acq. Instrument : Instrument 1   Location :   -
Injection Date : 8/25/2022 11:33:14 PM
Acq. Method   : C:\CHEM32\1\METHODS\DEF_LC.M
Last changed   : 8/25/2022 11:26:21 PM
                  (modified after loading)
Analysis Method : C:\CHEM32\1\METHODS\DEF_LC.M
Last changed   : 8/26/2022 9:38:23 PM
                  (modified after loading)
Sample Info   : AD-3, Hexane/i-PrOH = 90/10, 0.8 mL/min, 30 oC, 254 nm

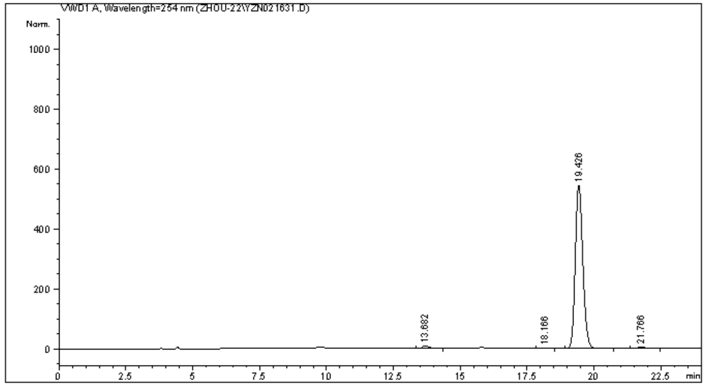

===
Area Percent Report
===

Sorted By   :   Signal
Multiplier: :   1.0000
Dilution: :   1.0000
Use Multiplier & Dilution Factor with ISTDs

Signal 1: VWD1 A, Wavelength=254 nm

Peak #	RetTime [min]	Type	Width [min]	Area mAU *s	Height [mAU]	Area %
1	13.682	VB	0.2632	175.35739	9.63931	1.5972
2	18.166	VV	0.2730	39.70545	2.24793	0.3616
3	19.426	VB	0.3034	1.06550e4	545.97540	97.0484
4	21.766	VB	0.3436	108.99452	4.90750	0.9928

Totals :   1.09790e4   562.77014

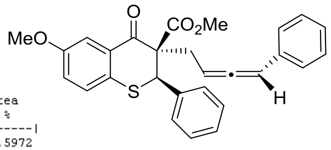

(-)-3na

===
*** End of Report ***

Instrument 1 8/26/2022 9:39:49 PM   Page 1 of 1

Data File C:\CHEM32\1\DATA\ZHOU-22\YZN021731.D
Sample Name: LL-10-74C +/-

===

Acq. Operator :
Acq. Instrument : Instrument 1 Location : -
Injection Date : 9/29/2022 10:31:50 PM
Acq. Method : C:\CHEM32\1\METHODS\DEF_LC.M
Last changed : 9/29/2022 10:28:25 PM
(modified after loading)
Analysis Method : C:\CHEM32\1\METHODS\DEF_LC.M
Last changed : 9/29/2022 11:26:15 PM
(modified after loading)
Sample Info : AD-H, Hexane/i-PrOH = 96/4, 1.0 mL/min, 30 oC, 254 nm

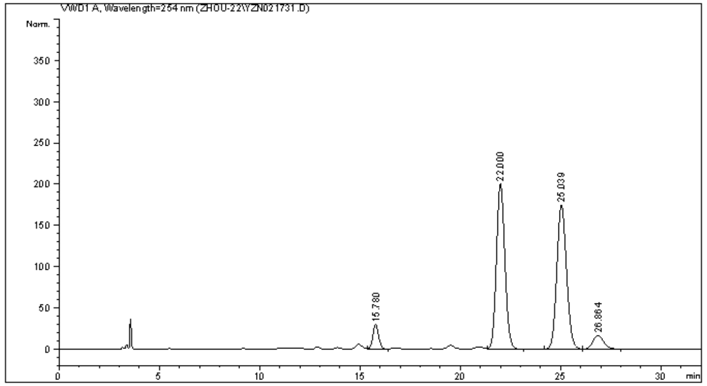

===

Area Percent Report

===

Sorted By : Signal
Multiplier: : 1.0000
Dilution: : 1.0000
Use Multiplier & Dilution Factor with ISTDs

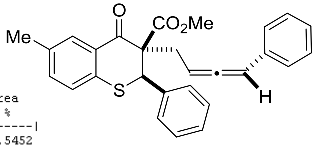

(+/-)-3oa

Signal 1: VWD1 A, Wavelength=254 nm

Peak #	RetTime [min]	Type	Width [min]	Area mAU *s	Height [mAU]	Area %
1	15.780	VV	0.3096	589.07922	29.38743	4.5452
2	22.000	VB	0.4587	5877.99072	200.36493	45.3529
3	25.039	VV	0.5261	5875.54785	174.82314	45.3341
4	26.864	VB	0.5741	617.93982	16.59704	4.7678

Totals : 1.29606e4 421.17253

===

*** End of Report ***

Instrument 1 9/29/2022 11:26:17 PM Page 1 of 1

Data File C:\CHEM32\1\DATA\ZHOU-22\YZN021730.D
Sample Name: LL-10-74C

===

Acq. Operator :
Acq. Instrument : Instrument 1 Location : -
Injection Date : 9/29/2022 9:50:45 PM
Acq. Method : C:\CHEM32\1\METHODS\DEF_LC.M
Last changed : 9/29/2022 9:29:36 PM
(modified after loading)
Analysis Method : C:\CHEM32\1\METHODS\DEF_LC.M
Last changed : 9/29/2022 11:26:47 PM
(modified after loading)
Sample Info : AD-H, Hexane/i-PrOH = 96/4, 1.0 mL/min, 30 oC, 254 nm

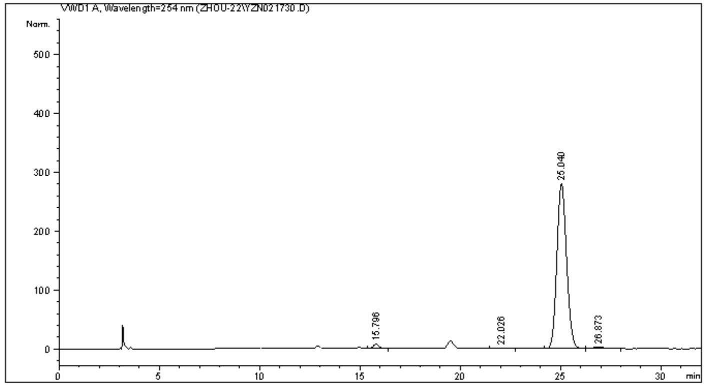

===

Area Percent Report

===

Sorted By : Signal
Multiplier: : 1.0000
Dilution: : 1.0000
Use Multiplier & Dilution Factor with ISTDs

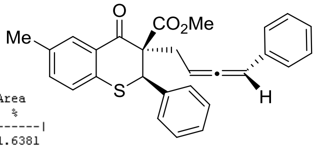

(-)-3oa

Signal 1: VWD1 A, Wavelength=254 nm

Peak #	RetTime [min]	Type	Width [min]	Area mAU *s	Height [mAU]	Area %
1	15.796	VB	0.3152	159.12651	7.84813	1.6381
2	22.026	BB	0.4457	26.53791	9.16074e-1	0.2732
3	25.040	BV	0.5227	9408.33594	280.25143	96.8517
4	26.873	VB	0.5841	120.17010	3.15510	1.2371

Totals : 9714.17046 292.17074

===

*** End of Report ***

Instrument 1 9/29/2022 11:28:52 PM Page 1 of 1

Data File C:\CHEM32\1\DATA\ZHOU-22\YZN021737.D
Sample Name: LL-10-74D +/-

===

Acq. Operator :
Acq. Instrument : Instrument 1 Location : -
Injection Date : 9/30/2022 4:09:33 AM
Acq. Method : C:\CHEM32\1\METHODS\DEF_LC.M
Last changed : 9/30/2022 3:22:41 AM
(modified after loading)
Analysis Method : C:\CHEM32\1\METHODS\DEF_LC.M
Last changed : 9/30/2022 10:30:07 AM
(modified after loading)
Sample Info : AD-3, Hexane/i-PrOH = 96/4, 0.8 mL/min, 30 oC, 254 nm

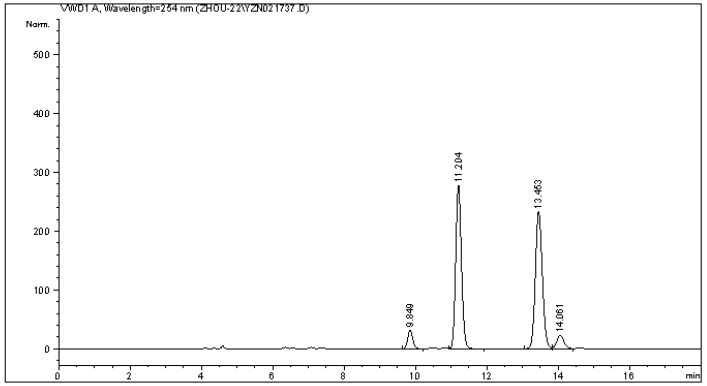

===
Area Percent Report
===

Sorted By : Signal
Multiplier: : 1.0000
Dilution: : 1.0000
Use Multiplier & Dilution Factor with ISTDs

Signal 1: VWD1 A, Wavelength=254 nm

Peak #	RetTime [min]	Type	Width [min]	Area mAU *s	Height [mAU]	Area %
1	9.849	BV	0.1524	313.24661	31.90422	4.5283
2	11.204	VB	0.1747	3124.62256	278.28778	45.1701
3	13.453	BV	0.2107	3161.98291	233.03334	45.7102
4	14.061	VV	0.2167	317.61075	22.55185	4.5914

Totals : 6917.46283 565.77719

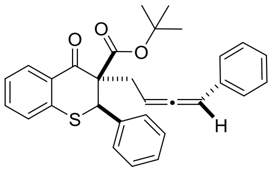

(+/-)-3pa

===
*** End of Report ***

Instrument 1 9/30/2022 10:30:10 AM Page 1 of 1

Data File C:\CHEM32\1\DATA\ZHOU-22\YZN021738.D
Sample Name: LL-10-74D

===

Acq. Operator :
Acq. Instrument : Instrument 1 Location : -
Injection Date : 9/30/2022 4:36:43 AM
Acq. Method : C:\CHEM32\1\METHODS\DEF_LC.M
Last changed : 9/30/2022 4:35:06 AM
(modified after loading)
Analysis Method : C:\CHEM32\1\METHODS\DEF_LC.M
Last changed : 9/30/2022 10:31:58 AM
(modified after loading)
Sample Info : AD-3, Hexane/i-PrOH = 96/4, 0.8 mL/min, 30 oC, 254 nm

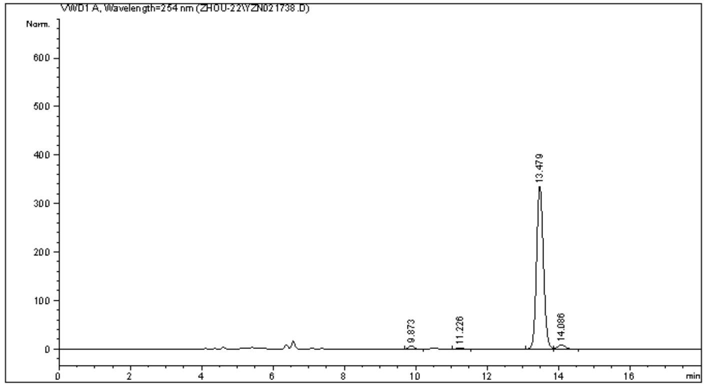

===
Area Percent Report
===

Sorted By : Signal
Multiplier: : 1.0000
Dilution: : 1.0000
Use Multiplier & Dilution Factor with ISTDs

Signal 1: VWD1 A, Wavelength=254 nm

Peak #	RetTime [min]	Type	Width [min]	Area mAU *s	Height [mAU]	Area %
1	9.873	BB	0.1520	64.97693	6.64180	1.3830
2	11.226	BB	0.1724	23.62246	2.14162	0.5028
3	13.479	BV	0.2086	4488.53320	335.39087	95.5353
4	14.086	VB	0.2211	121.16512	8.52858	2.5789

Totals : 4698.29771 352.70287

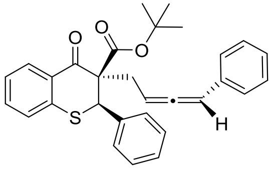

(-)-3pa

===
*** End of Report ***

Instrument 1 9/30/2022 10:32:01 AM Page 1 of 1

Data File C:\CHEM32\1\DATA\ZHOU-22\YZN022844.D
Sample Name: LL-12-24 +/-

===

Acq. Operator :
Acq. Instrument : Instrument 1 Location : -
Injection Date : 4/7/2023 7:29:20 AM
Acq. Method : C:\CHEM32\1\METHODS\DEF_LC.M
Last changed : 4/7/2023 7:13:23 AM
(modified after loading)
Analysis Method : C:\CHEM32\1\METHODS\DEF_LC.M
Last changed : 4/7/2023 8:39:19 AM
(modified after loading)
Sample Info : AD-H, Hexane/i-PrOH = 93/7, 1.0 mL/min, 30 oC, 254 nm

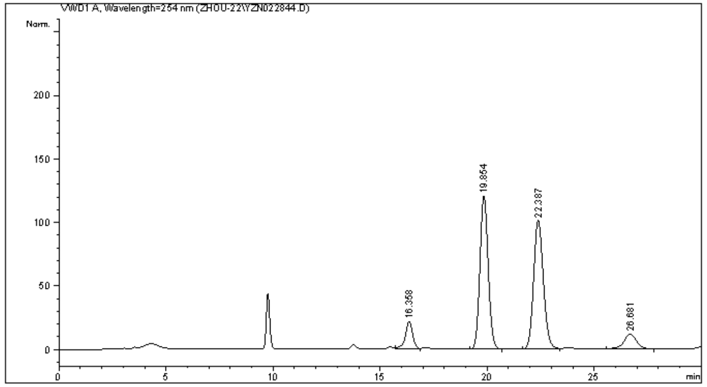

===
Area Percent Report
===

Sorted By : Signal
Multiplier: : 1.0000
Dilution: : 1.0000
Use Multiplier & Dilution Factor with ISTDs

Signal 1: VWD1 A, Wavelength=254 nm

Peak #	RetTime [min]	Type	Width [min]	Area mAU *s	Height [mAU]	Area %
1	16.358	VV	0.3672	524.67554	21.54059	7.0267
2	19.854	BB	0.4169	3234.56738	120.34997	43.3188
3	22.387	BB	0.4919	3219.40454	101.22676	43.1157
4	26.681	BB	0.6375	488.24710	11.68464	6.5388

Totals : 7466.89456 254.80195

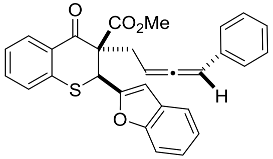

(+/-)-3qa

===
*** End of Report ***

Instrument 1 4/7/2023 8:39:24 AM Page 1 of 1

Data File C:\CHEM32\1\DATA\ZHOU-22\YZN022845.D
Sample Name: LL-12-24

===

Acq. Operator :
Acq. Instrument : Instrument 1 Location : -
Injection Date : 4/7/2023 8:07:17 AM
Acq. Method : C:\CHEM32\1\METHODS\DEF_LC.M
Last changed : 4/7/2023 8:05:23 AM
(modified after loading)
Analysis Method : C:\CHEM32\1\METHODS\DEF_LC.M
Last changed : 4/7/2023 8:41:35 AM
(modified after loading)
Sample Info : AD-H, Hexane/i-PrOH = 93/7, 1.0 mL/min, 30 oC, 254 nm

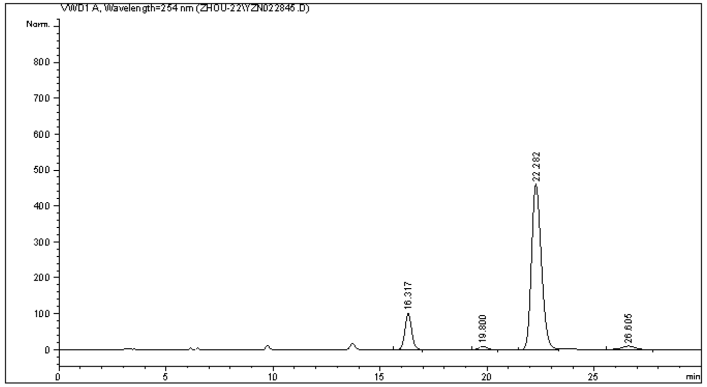

===
Area Percent Report
===

Sorted By : Signal
Multiplier: : 1.0000
Dilution: : 1.0000
Use Multiplier & Dilution Factor with ISTDs

Signal 1: VWD1 A, Wavelength=254 nm

Peak #	RetTime [min]	Type	Width [min]	Area mAU *s	Height [mAU]	Area %
1	16.317	VB	0.3393	2188.46094	100.18697	12.4988
2	19.800	BB	0.4179	235.59383	8.78028	1.3455
3	22.282	VV	0.4926	1.46637e4	460.16486	83.7479
4	26.605	BB	0.6436	421.57547	9.79131	2.4077

Totals : 1.75094e4 578.92341

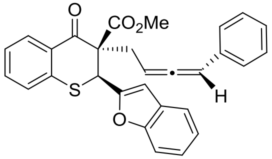

(+)-3qa

===
*** End of Report ***

Instrument 1 4/7/2023 8:41:43 AM Page 1 of 1

```
Data File D:\USERS\CHEMSTATION\1\DATA\ZHOU2022\YZ3005246  20-34-08.D
Sample Name: LL-10-70 D +/-

    =====================================================================
    Acq. Operator   : SYSTEM
    Sample Operator : SYSTEM
    Acq. Instrument : 1260II                        Location :     -
    Injection Date  : 9/29/2022 8:34:08 PM                 Inj :   1
                                                    Inj Volume : No inj
    Acq. Method     : C:\Users\Public\Documents\ChemStation\1\Methods\def_LC.M
    Last changed    : 9/29/2022 6:41:47 PM by SYSTEM
                      (modified after loading)
    Analysis Method : C:\Users\Public\Documents\ChemStation\1\Methods\def_LC.M
    Last changed    : 9/29/2022 10:32:32 PM by SYSTEM
                      (modified after loading)
    Sample Info     : OD-H, n-Hexane/i-PrOH = 95/5, 1.0 mL/min, 30 oC, 254 nm
```

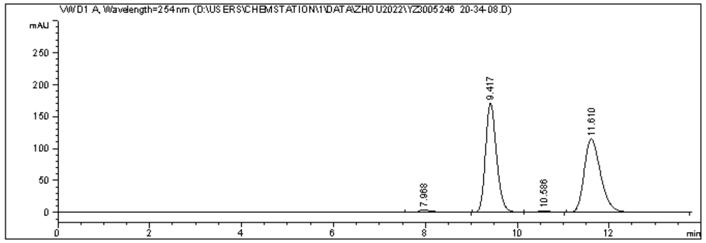


```
    =====================================================================
                            Area Percent Report
    =====================================================================

    Sorted By             :      Signal
    Multiplier            :      1.0000
    Dilution              :      1.0000
    Do not use Multiplier & Dilution Factor with ISTDs


    Signal 1: VWD1 A, Wavelength=254 nm
```

Peak #	RetTime [min]	Type	Width [min]	Area [mAU*s]	Height [mAU]	Area %
1	7.968	BB	0.3140	95.62804	4.22667	1.6388
2	9.417	BB	0.2554	2837.91357	171.22769	48.6328
3	10.586	BB	0.2802	42.20076	2.32035	0.7232
4	11.610	BB	0.3819	2859.64502	115.07438	49.0052

Totals : 5835.38739 292.84909

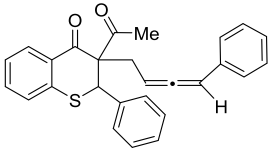

(+/-)-3ba

```
    =====================================================================
                          *** End of Report ***
```

1260II 9/29/2022 10:32:34 PM SYSTEM   Page 1 of 1

```
Data File D:\USERS\CHEMSTATION\1\DATA\ZHOU2022\YZ3005247  20-49-55.D
Sample Name: LL-10-70 D

    =====================================================================
    Acq. Operator   : SYSTEM
    Sample Operator : SYSTEM
    Acq. Instrument : 1260II                        Location :     -
    Injection Date  : 9/29/2022 8:49:55 PM                 Inj :   1
                                                    Inj Volume : No inj
    Acq. Method     : C:\Users\Public\Documents\ChemStation\1\Methods\def_LC.M
    Last changed    : 9/29/2022 6:41:47 PM by SYSTEM
                      (modified after loading)
    Analysis Method : C:\Users\Public\Documents\ChemStation\1\Methods\def_LC.M
    Last changed    : 9/29/2022 10:34:30 PM by SYSTEM
                      (modified after loading)
    Sample Info     : OD-H, n-Hexane/i-PrOH = 95/5, 1.0 mL/min, 30 oC, 254 nm

    Additional Info : Peak(s) manually integrated
```

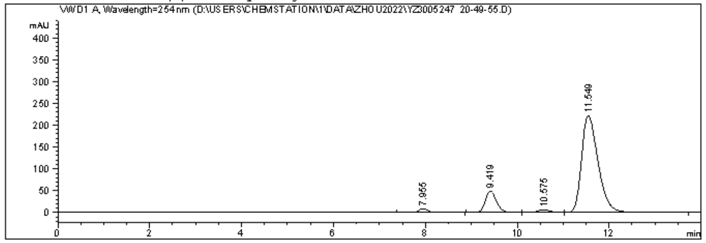


```
    =====================================================================
                            Area Percent Report
    =====================================================================

    Sorted By             :      Signal
    Multiplier            :      1.0000
    Dilution              :      1.0000
    Do not use Multiplier & Dilution Factor with ISTDs


    Signal 1: VWD1 A, Wavelength=254 nm
```

Peak #	RetTime [min]	Type	Width [min]	Area [mAU*s]	Height [mAU]	Area %
1	7.955	BB	0.2151	128.63974	9.09024	1.9534
2	9.419	BB	0.2575	820.85236	49.23763	12.4649
3	10.575	BV	0.2909	110.45456	5.83479	1.6773
4	11.549	VB	0.3810	5525.35205	222.30194	83.9043

Totals : 6585.29871 286.46461

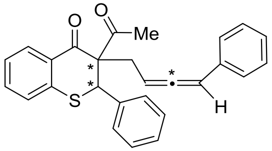

(+)-3ba

```
    =====================================================================
                          *** End of Report ***
```

1260II 9/29/2022 10:34:32 PM SYSTEM   Page 1 of 1

Data File C:\CHEM32\1\DATA\ZHOU-22\YZN022246.D
Sample Name: LL-11-55 +/-

===

Acq. Operator :
Acq. Instrument : Instrument 1 Location : -
Injection Date : 12/28/2022 3:07:07 AM
Acq. Method : C:\CHEM32\1\METHODS\DEF_LC.M
Last changed : 12/28/2022 2:38:29 AM
(modified after loading)
Analysis Method : C:\CHEM32\1\METHODS\DEF_LC.M
Last changed : 12/28/2022 7:07:08 AM
(modified after loading)
Sample Info : IB, Hexane/i-PrOH = 94/6, 1.0 mL/min, 30 oC, 254 nm

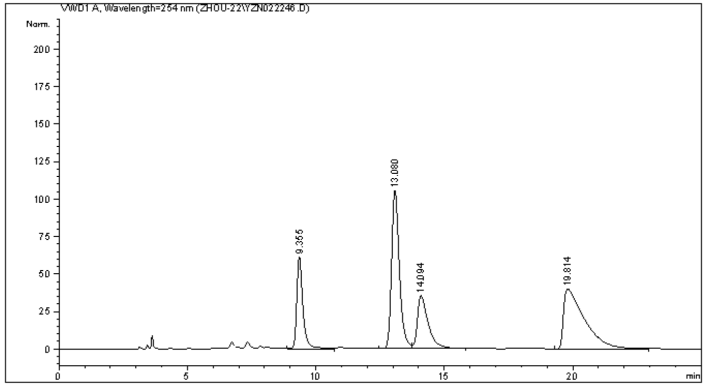

===
Area Percent Report
===

Sorted By : Signal
Multiplier: : 1.0000
Dilution: : 1.0000
Use Multiplier & Dilution Factor with ISTDs

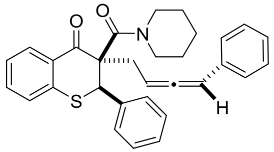

(+/-)-3da

Signal 1: VWD1 A, Wavelength=254 nm

Peak #	RetTime [min]	Type	Width [min]	Area mAU *s	Height [mAU]	Area %
1	9.355	VV	0.2485	1026.85571	61.56679	15.6667
2	13.080	BV	0.3230	2235.13110	105.44186	34.1014
3	14.094	VB	0.4324	1035.48242	35.31476	15.7983
4	19.814	VB	0.8030	2256.90283	39.87349	34.4335

Totals : 6554.37207 242.19690

===
*** End of Report ***

Instrument 1 12/28/2022 7:07:16 AM Page 1 of 1

Data File C:\CHEM32\1\DATA\ZHOU-22\YZN022247.D
Sample Name: LL-11-55

===

Acq. Operator :
Acq. Instrument : Instrument 1 Location : -
Injection Date : 12/28/2022 3:40:27 AM
Acq. Method : C:\CHEM32\1\METHODS\DEF_LC.M
Last changed : 12/28/2022 3:38:28 AM
(modified after loading)
Analysis Method : C:\CHEM32\1\METHODS\DEF_LC.M
Last changed : 12/28/2022 7:11:09 AM
(modified after loading)
Sample Info : IB, Hexane/i-PrOH = 94/6, 1.0 mL/min, 30 oC, 254 nm

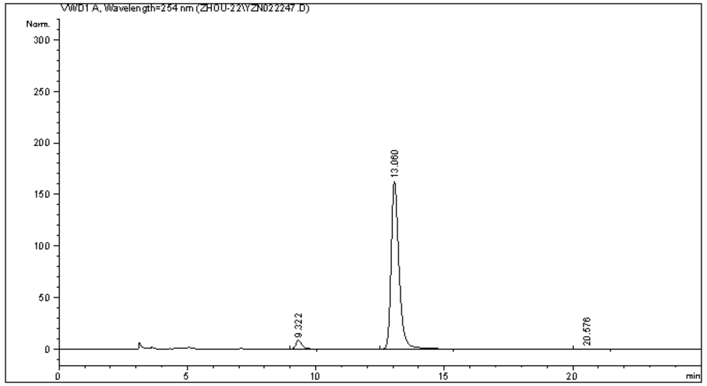

===
Area Percent Report
===

Sorted By : Signal
Multiplier: : 1.0000
Dilution: : 1.0000
Use Multiplier & Dilution Factor with ISTDs

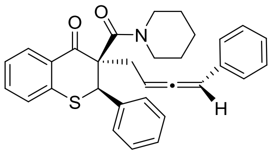

(-)-3da

Signal 1: VWD1 A, Wavelength=254 nm

Peak #	RetTime [min]	Type	Width [min]	Area mAU *s	Height [mAU]	Area %
1	9.322	BB	0.2406	133.84752	8.42971	3.7125
2	13.060	BB	0.3217	3467.33984	162.54366	96.1728
3	20.576	BB	0.5043	4.13658	9.73773e-2	0.1147

Totals : 3605.32394 171.07074

===
*** End of Report ***

Instrument 1 12/28/2022 7:11:14 AM Page 1 of 1

Data File C:\CHEM32\1\DATA\ZHOU-22\YZN021807.D
Sample Name: LL-10-91 +/-

===

Acq. Operator   :
Acq. Instrument : Instrument 1   Location :   -
Injection Date : 10/19/2022 9:35:00 PM
Acq. Method   : C:\CHEM32\1\METHODS\DEF_LC.M
Last changed   : 10/19/2022 9:33:18 PM
                  (modified after loading)
Analysis Method : C:\CHEM32\1\METHODS\DEF_LC.M
Last changed   : 10/20/2022 9:17:15 AM
                  (modified after loading)
Sample Info   : IB, Hexane/i-PrOH = 80/20, 1.0 mL/min, 30 oC, 254 nm

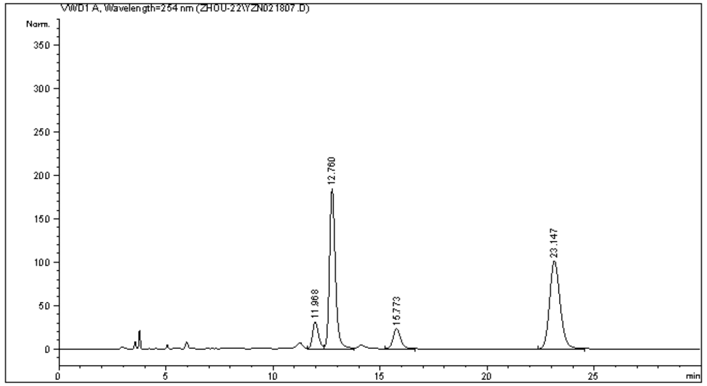

===
Area Percent Report
===

Sorted By   :   Signal
Multiplier: :   1.0000
Dilution: :   1.0000
Use Multiplier & Dilution Factor with ISTDs

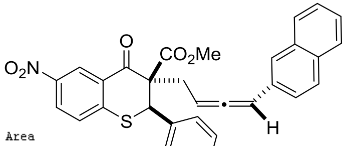

(+/-)-3mk

Signal 1: VWD1 A, Wavelength=254 nm

Peak #	RetTime [min]	Type	Width [min]	Area mAU *s	Height [mAU]	Area %
1	11.968	VV	0.2969	599.92419	31.03337	7.3543
2	12.760	VV	0.2895	3518.40283	184.44260	43.1311
3	15.773	BB	0.3779	568.60596	23.06100	6.9704
4	23.147	BB	0.5238	3470.52051	101.58165	42.5442
Totals :				8157.45349	340.11861	

===
*** End of Report ***

Instrument 1 10/20/2022 9:17:21 AM   Page 1 of 1

Data File C:\CHEM32\1\DATA\ZHOU-22\YZN021806.D
Sample Name: LL-10-91

===

Acq. Operator   :
Acq. Instrument : Instrument 1   Location :   -
Injection Date : 10/19/2022 8:20:31 PM
Acq. Method   : C:\CHEM32\1\METHODS\DEF_LC.M
Last changed   : 10/19/2022 7:51:35 PM
                  (modified after loading)
Analysis Method : C:\CHEM32\1\METHODS\DEF_LC.M
Last changed   : 10/20/2022 9:19:44 AM
                  (modified after loading)
Sample Info   : IB, Hexane/i-PrOH = 80/20, 1.0 mL/min, 30 oC, 254 nm

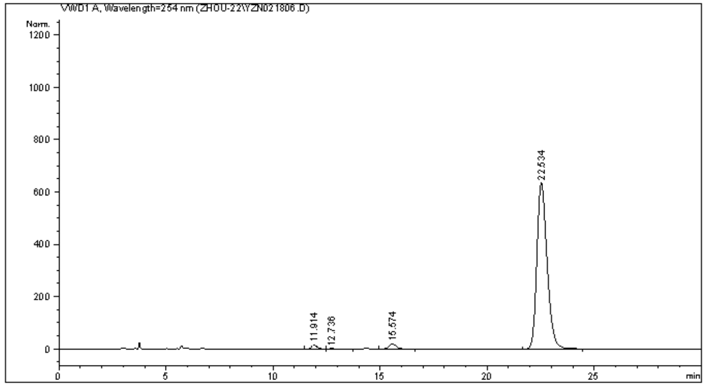

===
Area Percent Report
===

Sorted By   :   Signal
Multiplier: :   1.0000
Dilution: :   1.0000
Use Multiplier & Dilution Factor with ISTDs

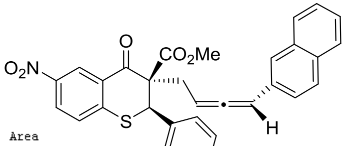

(-)-3mk

Signal 1: VWD1 A, Wavelength=254 nm

Peak #	RetTime [min]	Type	Width [min]	Area mAU *s	Height [mAU]	Area %
1	11.914	VV	0.3045	288.89047	15.81055	1.3195
2	12.736	VB	0.3716	34.89754	1.56533	0.1594
3	15.574	VB	0.4055	486.77145	20.00565	2.2233
4	22.534	BB	0.5537	2.10831e4	634.59418	96.2977
Totals :				2.18936e4	671.97571	

===
*** End of Report ***

Instrument 1 10/20/2022 9:19:49 AM   Page 1 of 1

Data File C:\CHEM32\1\DATA\ZHOU-22\YZN021511.D
Sample Name: LL-10-41B +/-

===

Acq. Operator :
Acq. Instrument : Instrument 1 Location : -
Injection Date : 7/12/2022 11:43:50 PM
Acq. Method : C:\CHEM32\1\METHODS\DEF_LC.M
Last changed : 7/12/2022 11:42:35 PM
(modified after loading)
Analysis Method : C:\CHEM32\1\METHODS\DEF_LC.M
Last changed : 7/13/2022 9:48:22 AM
(modified after loading)
Sample Info : OD-H, Hexane/i-PrOH = 95/5, 1.0 mL/min, 30 oC, 254 nm

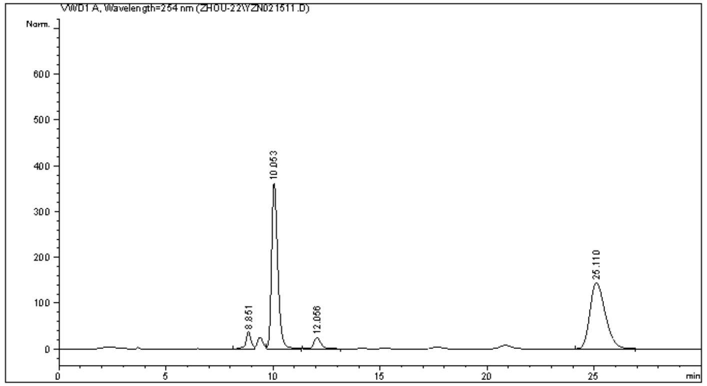

===
Area Percent Report
===

Sorted By : Signal
Multiplier: : 1.0000
Dilution: : 1.0000
Use Multiplier & Dilution Factor with ISTDs

Signal 1: VWD1 A, Wavelength=254 nm

Peak #	RetTime [min]	Type	Width [min]	Area mAU *s	Height [mAU]	Area %
1	8.851	BV	0.2473	609.97455	37.34377	3.8930
2	10.053	VB	0.3069	7261.25586	361.86163	46.3432
3	12.056	BB	0.3781	604.73486	24.14730	3.8596
4	25.110	BB	0.7700	7192.47559	144.86612	45.9042

Totals : 1.56684e4 568.21882

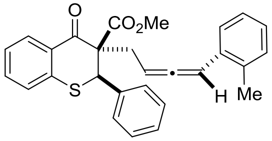

(+/-)-3cb

===
*** End of Report ***

Instrument 1 7/13/2022 9:51:09 AM Page 1 of 1

Data File C:\CHEM32\1\DATA\ZHOU-22\YZN021510.D
Sample Name: LL-10-41B

===

Acq. Operator :
Acq. Instrument : Instrument 1 Location : -
Injection Date : 7/12/2022 11:11:48 PM
Acq. Method : C:\CHEM32\1\METHODS\DEF_LC.M
Last changed : 7/12/2022 10:52:16 PM
(modified after loading)
Analysis Method : C:\CHEM32\1\METHODS\DEF_LC.M
Last changed : 7/13/2022 9:52:38 AM
(modified after loading)
Sample Info : OD-H, Hexane/i-PrOH = 95/5, 1.0 mL/min, 30 oC, 254 nm

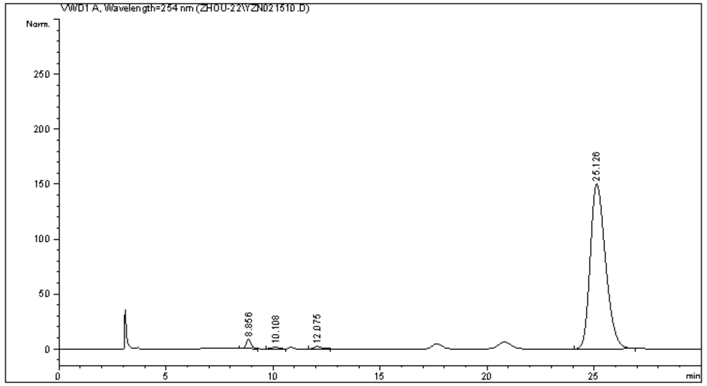

===
Area Percent Report
===

Sorted By : Signal
Multiplier: : 1.0000
Dilution: : 1.0000
Use Multiplier & Dilution Factor with ISTDs

Signal 1: VWD1 A, Wavelength=254 nm

Peak #	RetTime [min]	Type	Width [min]	Area mAU *s	Height [mAU]	Area %
1	8.856	BB	0.2383	131.87021	8.54310	1.7209
2	10.108	BV	0.3297	36.70646	1.70549	0.4790
3	12.075	BB	0.3454	49.80859	2.15486	0.6500
4	25.126	BB	0.7652	7444.52246	150.05699	97.1501

Totals : 7662.90771 162.46044

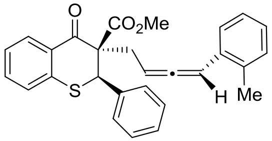

(-)-3cb

===
*** End of Report ***

Instrument 1 7/13/2022 9:52:40 AM Page 1 of 1

Data File C:\CHEM32\1\DATA\ZHOU-22\YZN021513.D
Sample Name: LL-10-41C +/-

===

Acq. Operator   :
Acq. Instrument : Instrument 1   Location :   -
Injection Date : 7/13/2022 2:40:13 AM
Acq. Method   : C:\CHEM32\1\METHODS\DEF_LC.M
Last changed   : 7/13/2022 2:36:21 AM
                  (modified after loading)
Analysis Method : C:\CHEM32\1\METHODS\DEF_LC.M
Last changed   : 7/13/2022 9:57:16 AM
                  (modified after loading)
Sample Info   : OD-H, Hexane/i-PrOH = 95/5, 1.0 mL/min, 30 oC, 254 nm

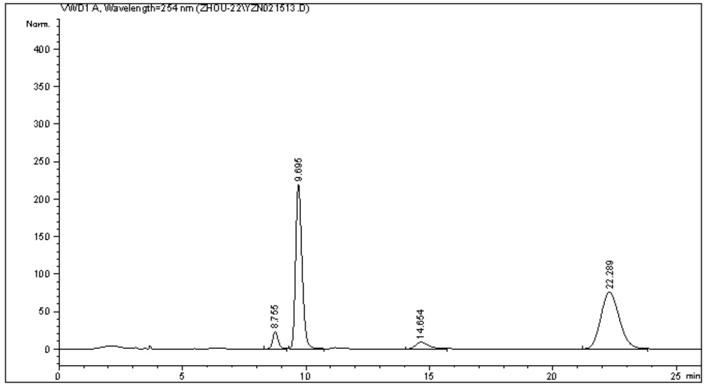

===
Area Percent Report
===

Sorted By : Signal
Multiplier: : 1.0000
Dilution: : 1.0000
Use Multiplier & Dilution Factor with ISTDs

Signal 1: VWD1 A, Wavelength=254 nm

Peak #	RetTime [min]	Type	Width [min]	Area mAU *s	Height [mAU]	Area %
1	8.755	BB	0.2370	355.48538	23.01476	4.1674
2	9.695	BB	0.2763	3939.74927	219.56552	46.1862
3	14.654	BB	0.5372	319.35623	8.91707	3.7439
4	22.289	BB	0.8076	3915.56177	75.65646	45.9026

Totals : 8530.15265 327.15382

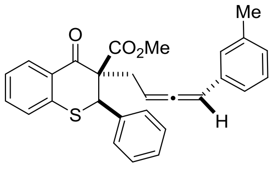

(+/-)-3cc

===
*** End of Report ***

Instrument 1 7/13/2022 9:57:19 AM   Page 1 of 1

Data File C:\CHEM32\1\DATA\ZHOU-22\YZN021512.D
Sample Name: LL-10-41C

===

Acq. Operator   :
Acq. Instrument : Instrument 1   Location :   -
Injection Date : 7/13/2022 2:03:52 AM
Acq. Method   : C:\CHEM32\1\METHODS\DEF_LC.M
Last changed   : 7/13/2022 1:18:45 AM
                  (modified after loading)
Analysis Method : C:\CHEM32\1\METHODS\DEF_LC.M
Last changed   : 7/13/2022 9:59:51 AM
                  (modified after loading)
Sample Info   : OD-H, Hexane/i-PrOH = 95/5, 1.0 mL/min, 30 oC, 254 nm

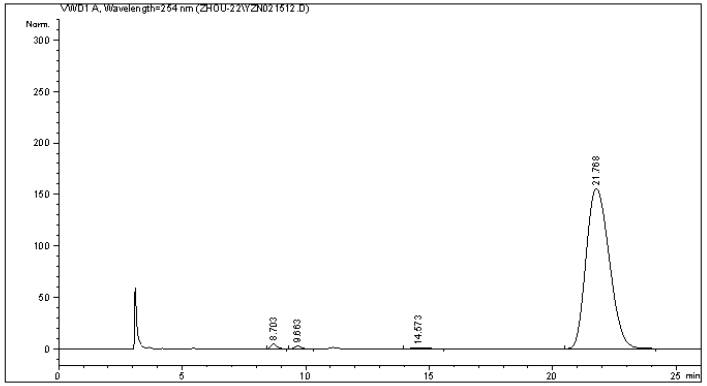

===
Area Percent Report
===

Sorted By : Signal
Multiplier: : 1.0000
Dilution: : 1.0000
Use Multiplier & Dilution Factor with ISTDs

Signal 1: VWD1 A, Wavelength=254 nm

Peak #	RetTime [min]	Type	Width [min]	Area mAU *s	Height [mAU]	Area %
1	8.703	BB	0.2401	75.96688	4.87366	0.7375
2	9.663	BB	0.2808	48.24833	2.63208	0.4684
3	14.573	BB	0.5498	41.64367	1.12108	0.4043
4	21.768	BB	1.0323	1.01341e4	155.85570	98.3897

Totals : 1.03000e4 164.48252

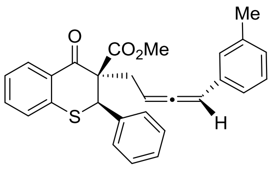

(-)-3cc

===
*** End of Report ***

Instrument 1 7/13/2022 9:59:54 AM   Page 1 of 1

Data File C:\CHEM32\1\DATA\ZHOU-22\YZN021515.D
Sample Name: LL-10-41D +/-

===

Acq. Operator :
Acq. Instrument : Instrument 1 Location : -
Injection Date : 7/13/2022 3:44:48 AM
Acq. Method : C:\CHEM32\1\METHODS\DEF_LC.M
Last changed : 7/13/2022 3:43:33 AM
(modified after loading)
Analysis Method : C:\CHEM32\1\METHODS\DEF_LC.M
Last changed : 7/13/2022 10:04:33 AM
(modified after loading)
Sample Info : OD-H, Hexane/i-PrOH = 95/5, 1.0 mL/min, 30 oC, 254 nm

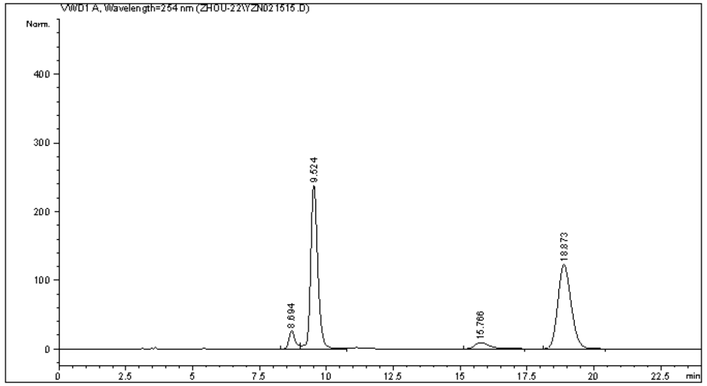

===
Area Percent Report
===

Sorted By : Signal
Multiplier: : 1.0000
Dilution: : 1.0000
Use Multiplier & Dilution Factor with ISTDs

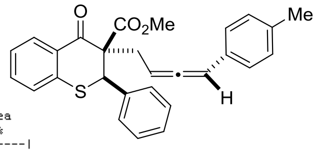

(+/-)-3cd

Signal 1: VWD1 A, Wavelength=254 nm

Peak #	RetTime [min]	Type	Width [min]	Area mAU *s	Height [mAU]	Area %
1	8.694	VV	0.2425	427.73480	26.45411	4.5443
2	9.524	VV	0.2810	4403.81104	238.46748	46.7862
3	15.766	BB	0.5838	353.51495	8.99354	3.7557
4	18.873	BB	0.5337	4227.57227	122.49112	44.9138

Totals : 9412.63306 396.40625

===
*** End of Report ***

Instrument 1 7/13/2022 10:04:37 AM Page 1 of 1

Data File C:\CHEM32\1\DATA\ZHOU-22\YZN021514.D
Sample Name: LL-10-41D

===

Acq. Operator :
Acq. Instrument : Instrument 1 Location : -
Injection Date : 7/13/2022 3:15:37 AM
Acq. Method : C:\CHEM32\1\METHODS\DEF_LC.M
Last changed : 7/13/2022 3:07:28 AM
(modified after loading)
Analysis Method : C:\CHEM32\1\METHODS\DEF_LC.M
Last changed : 7/13/2022 10:06:41 AM
(modified after loading)
Sample Info : OD-H, Hexane/i-PrOH = 95/5, 1.0 mL/min, 30 oC, 254 nm

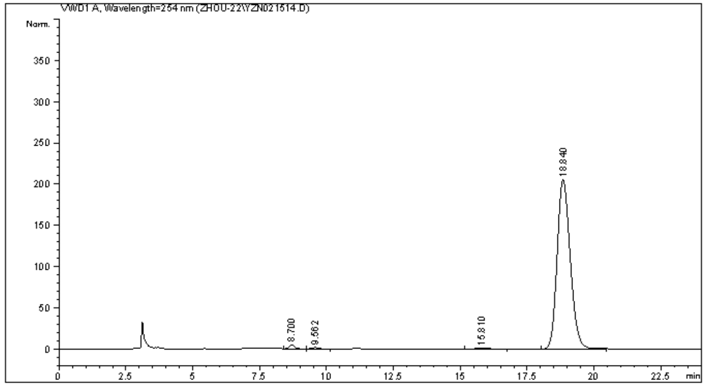

===
Area Percent Report
===

Sorted By : Signal
Multiplier: : 1.0000
Dilution: : 1.0000
Use Multiplier & Dilution Factor with ISTDs

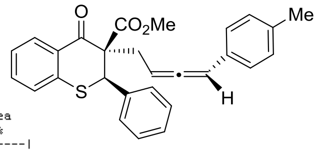

(-)-3cd

Signal 1: VWD1 A, Wavelength=254 nm

Peak #	RetTime [min]	Type	Width [min]	Area mAU *s	Height [mAU]	Area %
1	8.700	VV	0.2391	76.16090	4.83464	1.0714
2	9.562	VB	0.2840	31.32374	1.66183	0.4406
3	15.810	BB	0.4785	22.21332	5.92009e-1	0.3125
4	18.840	BB	0.5308	6978.90723	205.18338	98.1755

Totals : 7108.60519 212.27186

===
*** End of Report ***

Instrument 1 7/13/2022 10:06:43 AM Page 1 of 1

Data File C:\CHEM32\1\DATA\ZHOU-22\YZN021544.D
Sample Name: LL-10-48A +/-

Acq. Operator :
Acq. Instrument : Instrument 1 Location : -
Injection Date : 7/21/2022 9:23:56 PM
Acq. Method : C:\CHEM32\1\METHODS\DEF_LC.M
Last changed : 7/21/2022 9:23:44 PM
(modified after loading)
Analysis Method : C:\CHEM32\1\METHODS\DEF_LC.M
Last changed : 7/21/2022 11:39:52 PM
(modified after loading)
Sample Info : OD-3, Hexane/i-PrOH = 95/5, 0.8 mL/min, 30 oC, 254 nm

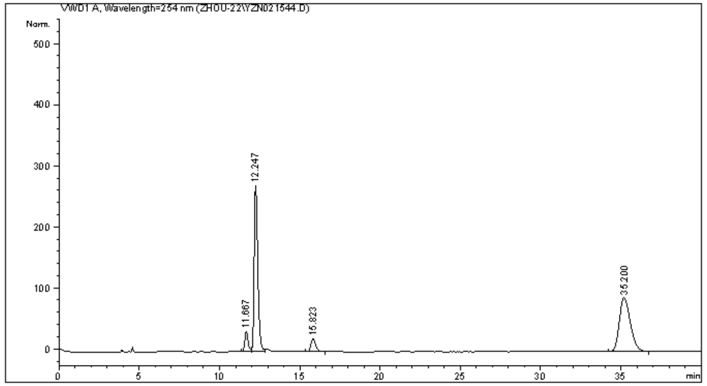

Area Percent Report

Sorted By : Signal
Multiplier: : 1.0000
Dilution: : 1.0000
Use Multiplier & Dilution Factor with ISTDs

Signal 1: VWD1 A, Wavelength=254 nm

Peak #	RetTime [min]	Type	Width [min]	Area mAU *s	Height [mAU]	Area %
1	11.667	VV	0.2227	487.38809	33.38966	5.1570
2	12.247	VV	0.2406	4294.65479	272.57999	45.4412
3	15.823	VB	0.3153	437.54269	21.18040	4.6296
4	35.200	BB	0.7468	4231.42529	88.31273	44.7722

Totals : 9451.01086 415.46278

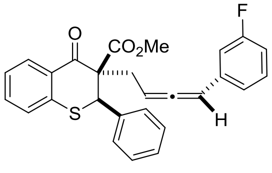

(+/-)-3ce

*** End of Report ***

Instrument 1 7/21/2022 11:41:35 PM Page 1 of 1

Data File C:\CHEM32\1\DATA\ZHOU-22\YZN021543.D
Sample Name: LL-10-48A

Acq. Operator :
Acq. Instrument : Instrument 1 Location : -
Injection Date : 7/21/2022 8:43:11 PM
Acq. Method : C:\CHEM32\1\METHODS\DEF_LC.M
Last changed : 7/21/2022 7:55:42 PM
(modified after loading)
Analysis Method : C:\CHEM32\1\METHODS\DEF_LC.M
Last changed : 7/21/2022 11:39:52 PM
(modified after loading)
Sample Info : OD-3, Hexane/i-PrOH = 95/5, 0.8 mL/min, 30 oC, 254 nm

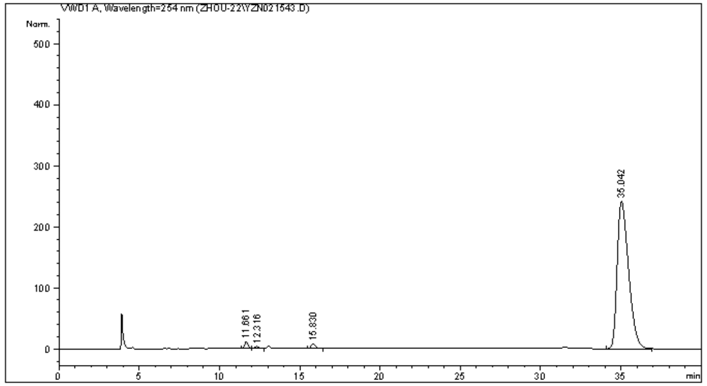

Area Percent Report

Sorted By : Signal
Multiplier: : 1.0000
Dilution: : 1.0000
Use Multiplier & Dilution Factor with ISTDs

Signal 1: VWD1 A, Wavelength=254 nm

Peak #	RetTime [min]	Type	Width [min]	Area mAU *s	Height [mAU]	Area %
1	11.661	BV	0.2154	163.26605	11.68452	1.3286
2	12.316	VB	0.2666	64.76287	3.67800	0.5270
3	15.830	BB	0.3145	155.72932	7.60719	1.2673
4	35.042	BB	0.7585	1.19044e4	241.51691	96.8770

Totals : 1.22881e4 264.48662

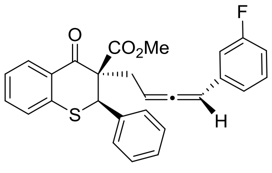

(-)-3ce

*** End of Report ***

Instrument 1 7/21/2022 11:39:56 PM Page 1 of 1

Data File C:\CHEM32\1\DATA\ZHOU-22\YZN021546.D
Sample Name: LL-10-48B +/-

===

Acq. Operator :
Acq. Instrument : Instrument 1 Location : -
Injection Date : 7/21/2022 10:48:39 PM
Acq. Method : C:\CHEM32\1\METHODS\DEF_LC.M
Last changed : 7/21/2022 10:48:12 PM
(modified after loading)
Analysis Method : C:\CHEM32\1\METHODS\DEF_LC.M
Last changed : 7/22/2022 10:06:38 AM
(modified after loading)
Sample Info : OD-3, Hexane/i-PrOH = 95/5, 0.8 mL/min, 30 oC, 254 nm

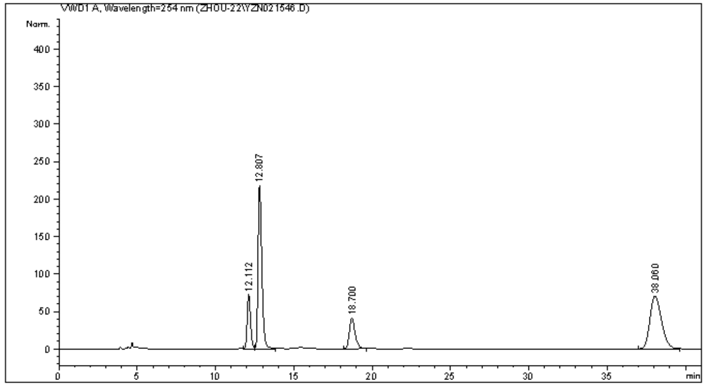

===
Area Percent Report
===

Sorted By : Signal
Multiplier: : 1.0000
Dilution: : 1.0000
Use Multiplier & Dilution Factor with ISTDs

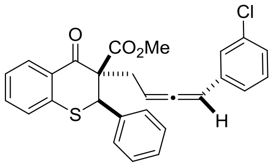

(+/-)-3cf

Signal 1: VWD1 A, Wavelength=254 nm

Peak #	RetTime [min]	Type	Width [min]	Area mAU *s	Height [mAU]	Area %
1	12.112	VV	0.2295	1082.02344	72.47380	11.4343
2	12.807	VB	0.2594	3699.03784	217.69196	39.0898
3	18.700	BB	0.3912	1055.68799	41.12384	11.1560
4	38.060	BB	0.7947	3626.18237	70.56090	38.3199

Totals : 9462.93164 401.85050

===
*** End of Report ***

Instrument 1 7/22/2022 10:07:05 AM Page 1 of 1

Data File C:\CHEM32\1\DATA\ZHOU-22\YZN021545.D
Sample Name: LL-10-48B

===

Acq. Operator :
Acq. Instrument : Instrument 1 Location : -
Injection Date : 7/21/2022 10:05:32 PM
Acq. Method : C:\CHEM32\1\METHODS\DEF_LC.M
Last changed : 7/21/2022 10:04:30 PM
(modified after loading)
Analysis Method : C:\CHEM32\1\METHODS\DEF_LC.M
Last changed : 7/22/2022 10:04:30 AM
(modified after loading)
Sample Info : OD-3, Hexane/i-PrOH = 95/5, 0.8 mL/min, 30 oC, 254 nm

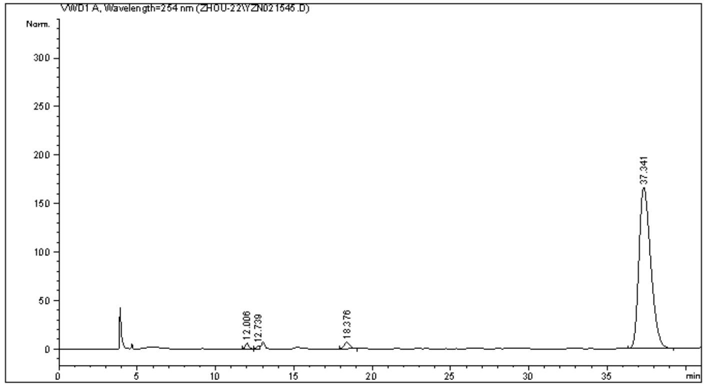

===
Area Percent Report
===

Sorted By : Signal
Multiplier: : 1.0000
Dilution: : 1.0000
Use Multiplier & Dilution Factor with ISTDs

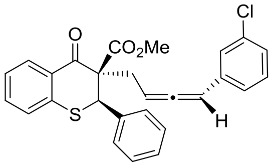

(-)-3cf

Signal 1: VWD1 A, Wavelength=254 nm

Peak #	RetTime [min]	Type	Width [min]	Area mAU *s	Height [mAU]	Area %
1	12.006	BB	0.2282	85.89397	5.79628	0.9573
2	12.739	BV	0.2121	43.48048	3.11931	0.4846
3	18.376	BB	0.3801	162.03986	6.58871	1.8060
4	37.341	BB	0.8133	8680.69629	166.17635	96.7520

Totals : 8972.11060 181.68064

===
*** End of Report ***

Instrument 1 7/22/2022 10:04:39 AM Page 1 of 1

Data File C:\CHEM32\1\DATA\ZHOU-22\YZN021548.D
Sample Name: LL-10-48C +/-

===

Acq. Operator :
Acq. Instrument : Instrument 1 Location : -
Injection Date : 7/22/2022 12:16:30 AM
Acq. Method : C:\CHEM32\1\METHODS\DEF_LC.M
Last changed : 7/22/2022 12:15:50 AM
(modified after loading)
Analysis Method : C:\CHEM32\1\METHODS\DEF_LC.M
Last changed : 7/22/2022 10:14:58 AM
(modified after loading)
Sample Info : OD-3, Hexane/i-PrOH = 95/5, 0.8 mL/min, 30 oC, 254 nm

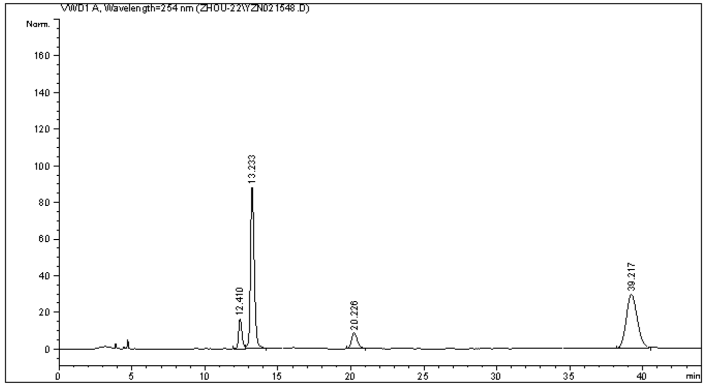

===

Area Percent Report

===

Sorted By : Signal
Multiplier: : 1.0000
Dilution: : 1.0000
Use Multiplier & Dilution Factor with ISTDs

Signal 1: VWD1 A, Wavelength=254 nm

Peak #	RetTime [min]	Type	Width [min]	Area mAU *s	Height [mAU]	Area %
1	12.410	BV	0.2463	260.25967	16.14247	7.2047
2	13.233	VB	0.2767	1596.99915	88.23946	44.2091
3	20.226	BB	0.4272	237.74484	8.64189	6.5814
4	39.217	BB	0.8056	1517.37439	29.34702	42.0049

Totals : 3612.37805 142.37085

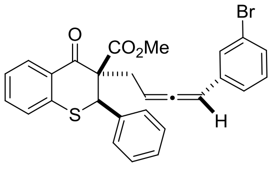

(+/-)-3cg

===

*** End of Report ***

Instrument 1 7/22/2022 10:15:03 AM Page 1 of 1

Data File C:\CHEM32\1\DATA\ZHOU-22\YZN021547.D
Sample Name: LL-10-48C

===

Acq. Operator :
Acq. Instrument : Instrument 1 Location : -
Injection Date : 7/21/2022 11:31:27 PM
Acq. Method : C:\CHEM32\1\METHODS\DEF_LC.M
Last changed : 7/21/2022 11:31:04 PM
(modified after loading)
Analysis Method : C:\CHEM32\1\METHODS\DEF_LC.M
Last changed : 7/22/2022 10:13:11 AM
(modified after loading)
Sample Info : OD-3, Hexane/i-PrOH = 95/5, 0.8 mL/min, 30 oC, 254 nm

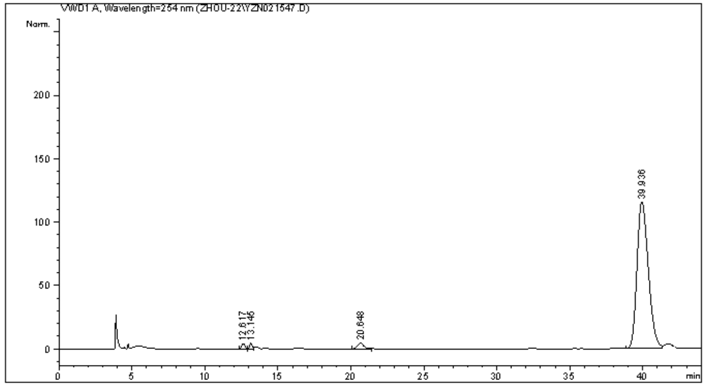

===

Area Percent Report

===

Sorted By : Signal
Multiplier: : 1.0000
Dilution: : 1.0000
Use Multiplier & Dilution Factor with ISTDs

Signal 1: VWD1 A, Wavelength=254 nm

Peak #	RetTime [min]	Type	Width [min]	Area mAU *s	Height [mAU]	Area %
1	12.617	BV	0.2411	66.40042	4.23675	1.0209
2	13.145	VV	0.1952	58.21764	4.57029	0.8951
3	20.648	BB	0.4388	126.19016	4.50699	1.9402
4	39.936	BV	0.8360	6253.27637	115.93504	96.1438

Totals : 6504.08458 129.24908

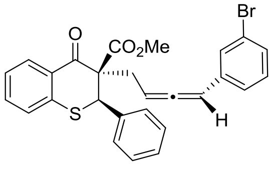

(-)-3cg

===

*** End of Report ***

Instrument 1 7/22/2022 10:13:15 AM Page 1 of 1

Data File C:\CHEM32\1\DATA\ZHOU-22\YZN021529.D
Sample Name: LL-10-48D +/-

===

Acq. Operator   :
Acq. Instrument : Instrument 1   Location :   -
Injection Date : 7/19/2022 9:39:10 PM
Acq. Method   : C:\CHEM32\1\METHODS\DEF_LC.M
Last changed   : 7/19/2022 9:37:20 PM
                  (modified after loading)
Analysis Method : C:\CHEM32\1\METHODS\DEF_LC.M
Last changed   : 7/20/2022 3:16:36 AM
                  (modified after loading)
Sample Info   : OD-3, Hexane/i-PrOH = 94/6, 0.8 mL/min, 30 oC, 254 nm

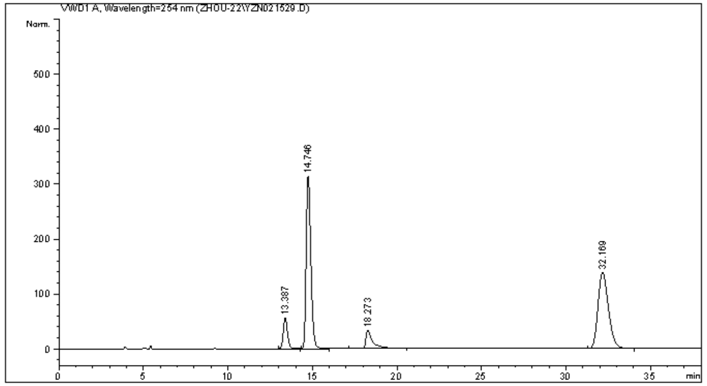

===
Area Percent Report
===

Sorted By   :   Signal
Multiplier: :   1.0000
Dilution: :   1.0000
Use Multiplier & Dilution Factor with ISTDs

Signal 1: VWD1 A, Wavelength=254 nm

Peak #	RetTime [min]	Type	Width [min]	Area mAU *s	Height [mAU]	Area %
1	13.387	BB	0.2705	982.42444	55.91048	7.0429
2	14.746	BB	0.2928	5998.19287	313.98233	43.0003
3	18.273	VB	0.4124	990.43689	33.74031	7.1003
4	32.169	BB	0.6657	5978.12354	138.77162	42.8565

Totals :   1.39492e4   542.40474

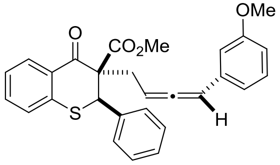

(+/-)-3ch

===
*** End of Report ***

Instrument 1 7/20/2022 3:16:46 AM   Page 1 of 1

Data File C:\CHEM32\1\DATA\ZHOU-22\YZN021528.D
Sample Name: LL-10-48D

===

Acq. Operator   :
Acq. Instrument : Instrument 1   Location :   -
Injection Date : 7/19/2022 8:54:08 PM
Acq. Method   : C:\CHEM32\1\METHODS\DEF_LC.M
Last changed   : 7/19/2022 8:07:12 PM
                  (modified after loading)
Analysis Method : C:\CHEM32\1\METHODS\DEF_LC.M
Last changed   : 7/20/2022 3:17:36 AM
                  (modified after loading)
Sample Info   : OD-3, Hexane/i-PrOH = 94/6, 0.8 mL/min, 30 oC, 254 nm

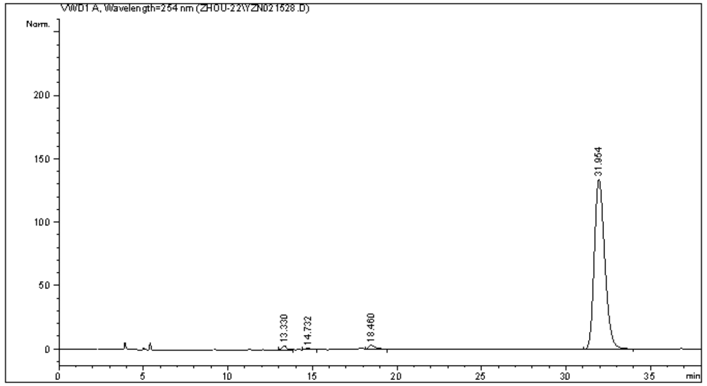

===
Area Percent Report
===

Sorted By   :   Signal
Multiplier: :   1.0000
Dilution: :   1.0000
Use Multiplier & Dilution Factor with ISTDs

Signal 1: VWD1 A, Wavelength=254 nm

Peak #	RetTime [min]	Type	Width [min]	Area mAU *s	Height [mAU]	Area %
1	13.330	BB	0.2765	56.67506	3.20063	0.9536
2	14.732	BB	0.2897	20.42396	1.08425	0.3436
3	18.460	VB	0.4683	104.38224	3.24607	1.7563
4	31.954	BB	0.6648	5761.81055	133.98936	96.9465

Totals :   5943.29180   141.52031

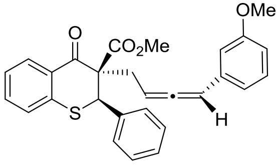

(-)-3ch

===
*** End of Report ***

Instrument 1 7/20/2022 3:17:46 AM   Page 1 of 1

```
Data File C:\CHEM32\1\DATA\ZHOU-22\YZN021729.D
Sample Name: LL-10-74A +/-

=====================================================================
Acq. Operator   :
Acq. Instrument : Instrument 1                    Location :   -
Injection Date  : 9/29/2022 9:03:32 PM
Acq. Method     : C:\CHEM32\1\METHODS\DEF_LC.M
Last changed    : 9/29/2022 8:58:52 PM
                  (modified after loading)
Analysis Method : C:\CHEM32\1\METHODS\DEF_LC.M
Last changed    : 9/29/2022 11:14:30 PM
                  (modified after loading)
Sample Info     : OD-H, Hexane/i-PrOH = 96/4, 1.0 mL/min, 30 oC, 254 nm
```

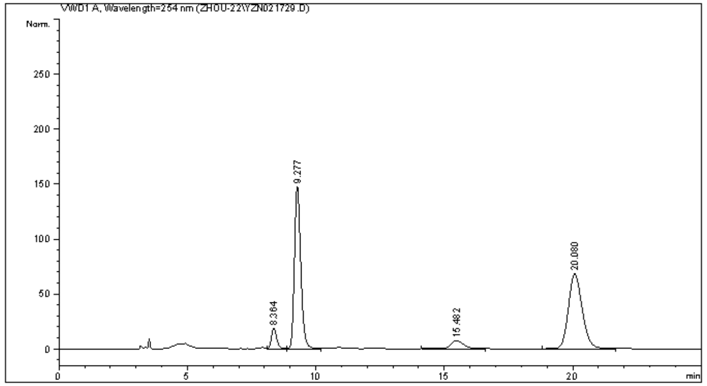


```
=====================================================================
                     Area Percent Report
=====================================================================

Sorted By             :      Signal
Multiplier:                  :      1.0000
Dilution:                    :      1.0000
Use Multiplier & Dilution Factor with ISTDs


Signal 1: VWD1 A, Wavelength=254 nm

Peak RetTime Type  Width     Area      Height     Area
  #   [min]        [min]  mAU   *s   [mAU   ]      %
----|-------|----|-------|----------|----------|--------|
   1   8.364 VV    0.2272  277.44485   18.67989   4.9168
   2   9.277 VB    0.2654 2550.08911  147.77696  45.1921
   3  15.482 BB    0.5470  275.74170    7.57428   4.8866
   4  20.080 BB    0.5712 2539.49609   68.20557  45.0044

Totals :                  5642.77176  242.23669

=====================================================================
                     *** End of Report ***
```

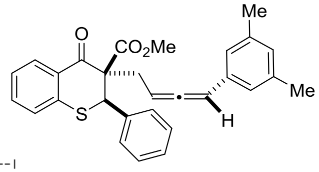

(+/-)-3ci

Instrument 1 9/29/2022 11:14:34 PM   Page 1 of 1

```
Data File C:\CHEM32\1\DATA\ZHOU-22\YZN021728.D
Sample Name: LL-10-74A

=====================================================================
Acq. Operator   :
Acq. Instrument : Instrument 1                    Location :   -
Injection Date  : 9/29/2022 8:33:03 PM
Acq. Method     : C:\CHEM32\1\METHODS\DEF_LC.M
Last changed    : 9/29/2022 7:49:40 PM
                  (modified after loading)
Analysis Method : C:\CHEM32\1\METHODS\DEF_LC.M
Last changed    : 9/29/2022 11:15:41 PM
                  (modified after loading)
Sample Info     : OD-H, Hexane/i-PrOH = 96/4, 1.0 mL/min, 30 oC, 254 nm
```

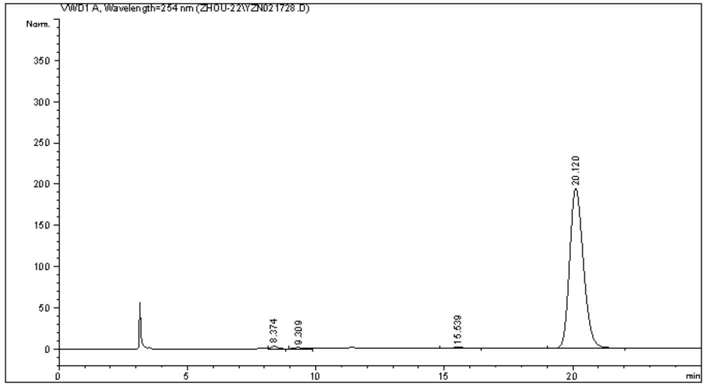


```
=====================================================================
                     Area Percent Report
=====================================================================

Sorted By             :      Signal
Multiplier:                  :      1.0000
Dilution:                    :      1.0000
Use Multiplier & Dilution Factor with ISTDs


Signal 1: VWD1 A, Wavelength=254 nm

Peak RetTime Type  Width     Area      Height     Area
  #   [min]        [min]  mAU   *s   [mAU   ]      %
----|-------|----|-------|----------|----------|--------|
   1   8.374 VB    0.2196   46.83584    3.29737   0.6383
   2   9.309 BB    0.2758   27.89707    1.59160   0.3802
   3  15.539 BB    0.4939   47.86770    1.43521   0.6524
   4  20.120 BB    0.5762 7214.99951  194.17969  98.3291

Totals :                  7337.60013  200.50386

=====================================================================
                     *** End of Report ***
```

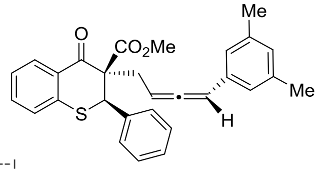

(-)-3ci

Instrument 1 9/29/2022 11:15:45 PM   Page 1 of 1

Data File C:\CHEM32\1\DATA\ZHOU-22\YZN021715.D
Sample Name: LL-10-74B +/-

===

Acq. Operator :
Acq. Instrument : Instrument 1 Location : -
Injection Date : 9/28/2022 9:40:16 PM
Acq. Method : C:\CHEM32\1\METHODS\DEF_LC.M
Last changed : 9/28/2022 8:27:29 PM
(modified after loading)
Analysis Method : C:\CHEM32\1\METHODS\DEF_LC.M
Last changed : 9/30/2022 10:27:10 AM
(modified after loading)
Sample Info : ID+IC, Hexane/i-PrOH = 94/6, 1.0 mL/min, 30 oC, 254 nm

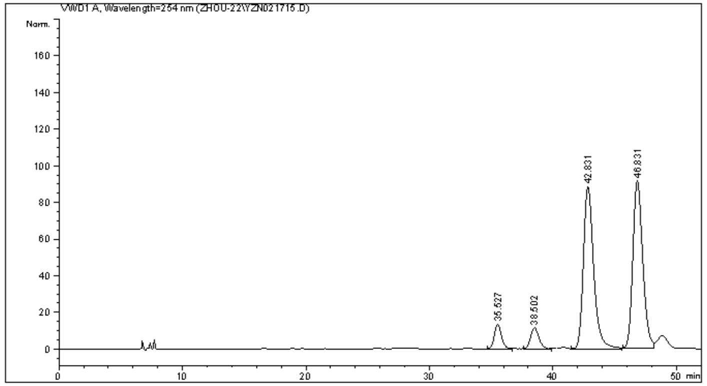

===
Area Percent Report
===

Sorted By : Signal
Multiplier: : 1.0000
Dilution: : 1.0000
Use Multiplier & Dilution Factor with ISTDs

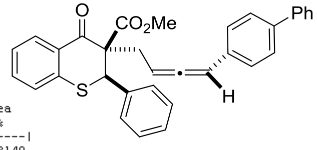

(+/-)-3cj

Signal 1: VWD1 A, Wavelength=254 nm

Peak #	RetTime [min]	Type	Width [min]	Area mAU *s	Height [mAU]	Area %
1	35.527	BB	0.6267	544.41461	13.44718	4.8149
2	38.502	BB	0.7234	538.08063	11.44043	4.7589
3	42.831	VB	0.8775	5104.59961	88.40113	45.1458
4	46.831	BV	0.8589	5119.81348	91.58820	45.2804

Totals : 1.13069e4 204.87693

===
*** End of Report ***

Instrument 1 9/30/2022 10:27:12 AM Page 1 of 1

Data File C:\CHEM32\1\DATA\ZHOU-22\YZN021734.D
Sample Name: LL-10-74B

===

Acq. Operator :
Acq. Instrument : Instrument 1 Location : -
Injection Date : 9/30/2022 12:25:01 AM
Acq. Method : C:\CHEM32\1\METHODS\DEF_LC.M
Last changed : 9/29/2022 11:54:09 PM
(modified after loading)
Analysis Method : C:\CHEM32\1\METHODS\DEF_LC.M
Last changed : 9/30/2022 10:23:42 AM
(modified after loading)
Sample Info : ID+IC, Hexane/i-PrOH = 94/6, 1.0 mL/min, 30 oC, 254 nm

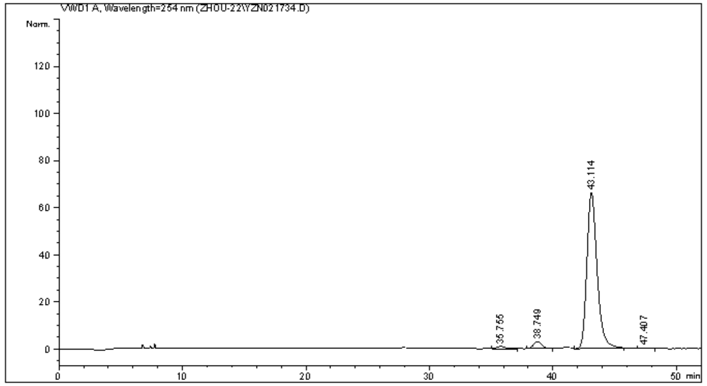

===
Area Percent Report
===

Sorted By : Signal
Multiplier: : 1.0000
Dilution: : 1.0000
Use Multiplier & Dilution Factor with ISTDs

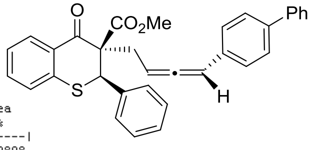

(-)-3cj

Signal 1: VWD1 A, Wavelength=254 nm

Peak #	RetTime [min]	Type	Width [min]	Area mAU *s	Height [mAU]	Area %
1	35.755	BB	0.6426	43.35707	9.66871e-1	1.0808
2	38.749	BB	0.7249	145.61395	3.07175	3.6298
3	43.114	VB	0.8817	3812.06421	66.17386	95.0265
4	47.407	BB	0.5337	10.54632	2.52585e-1	0.2629

Totals : 4011.58155 70.46507

===
*** End of Report ***

Instrument 1 9/30/2022 10:23:55 AM Page 1 of 1

Data File C:\CHEM32\1\DATA\ZHOU-22\YZN021587.D
Sample Name: LL-10-53B +/-

===

Acq. Operator :
Acq. Instrument : Instrument 1 Location : -
Injection Date : 7/29/2022 2:53:24 AM
Acq. Method : C:\CHEM32\1\METHODS\DEF_LC.M
Last changed : 7/29/2022 2:52:48 AM
(modified after loading)
Analysis Method : C:\CHEM32\1\METHODS\DEF_LC.M
Last changed : 7/29/2022 4:35:49 AM
(modified after loading)
Sample Info : IC, Hexane/i-PrOH = 94/6, 1.0 mL/min, 30 oC, 254 nm

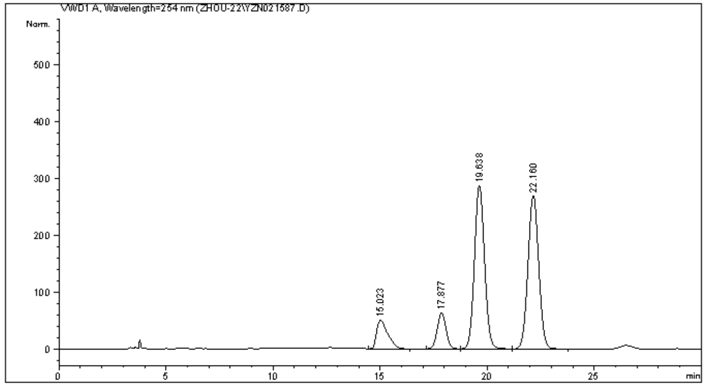

===
Area Percent Report
===

Sorted By : Signal
Multiplier: : 1.0000
Dilution: : 1.0000
Use Multiplier & Dilution Factor with ISTDs

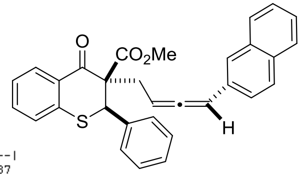

(+/-)-3ck

Signal 1: VWD1 A, Wavelength=254 nm

Peak #	RetTime [min]	Type	Width [min]	Area mAU *s	Height [mAU]	Area %
1	15.023	BB	0.5436	1811.80005	49.99577	8.0487
2	17.877	BV	0.4428	1806.43420	63.45760	8.0248
3	19.638	VV	0.5112	9447.49902	287.71786	41.9691
4	22.160	VB	0.5446	9444.86133	270.23135	41.9574

Totals : 2.25106e4 671.40259

===
*** End of Report ***

Instrument 1 7/29/2022 4:35:55 AM Page 1 of 1

Data File C:\CHEM32\1\DATA\ZHOU-22\YZN021588.D
Sample Name: LL-10-53B

===

Acq. Operator :
Acq. Instrument : Instrument 1 Location : -
Injection Date : 7/29/2022 3:28:15 AM
Acq. Method : C:\CHEM32\1\METHODS\DEF_LC.M
Last changed : 7/29/2022 3:26:18 AM
(modified after loading)
Analysis Method : C:\CHEM32\1\METHODS\DEF_LC.M
Last changed : 7/29/2022 4:34:04 AM
(modified after loading)
Sample Info : IC, Hexane/i-PrOH = 94/6, 1.0 mL/min, 30 oC, 254 nm

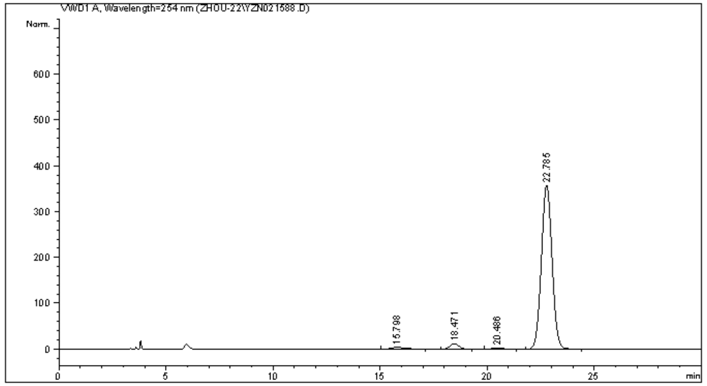

===
Area Percent Report
===

Sorted By : Signal
Multiplier: : 1.0000
Dilution: : 1.0000
Use Multiplier & Dilution Factor with ISTDs

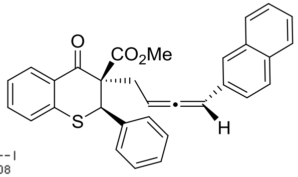

(-)-3ck

Signal 1: VWD1 A, Wavelength=254 nm

Peak #	RetTime [min]	Type	Width [min]	Area mAU *s	Height [mAU]	Area %
1	15.798	VB	0.6727	173.94283	3.61031	1.3408
2	18.471	BB	0.4384	339.25488	12.02127	2.6150
3	20.486	BB	0.5023	89.74234	2.76555	0.6917
4	22.785	BB	0.5372	1.23705e4	357.86490	95.3525

Totals : 1.29734e4 376.26203

===
*** End of Report ***

Instrument 1 7/29/2022 4:34:08 AM Page 1 of 1

Data File C:\CHEM32\1\DATA\ZHOU-22\YZN021585.D
Sample Name: LL-10-53C +/-

===

Acq. Operator :
Acq. Instrument : Instrument 1   Location : -
Injection Date : 7/28/2022 11:13:46 PM
Acq. Method : C:\CHEM32\1\METHODS\DEF_LC.M
Last changed : 7/28/2022 11:11:35 PM
(modified after loading)
Analysis Method : C:\CHEM32\1\METHODS\DEF_LC.M
Last changed : 7/29/2022 4:48:25 AM
(modified after loading)
Sample Info : OD-3, Hexane/i-PrOH = 93/7, 0.8 mL/min, 30 oC, 254 nm

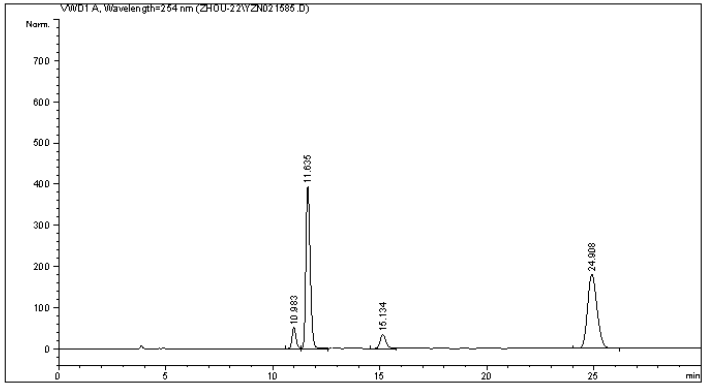

===
Area Percent Report
===

Sorted By : Signal
Multiplier: : 1.0000
Dilution: : 1.0000
Use Multiplier & Dilution Factor with ISTDs

Signal 1: VWD1 A, Wavelength=254 nm

Peak #	RetTime [min]	Type	Width [min]	Area mAU *s	Height [mAU]	Area %
1	10.983	VV	0.1991	658.74353	50.87207	5.3293
2	11.635	VB	0.2163	5523.64844	393.20840	44.6868
3	15.134	VV	0.2871	649.02606	34.62311	5.2507
4	24.908	VB	0.4777	5529.38184	180.06413	44.7332

Totals : 1.23608e4 658.76772

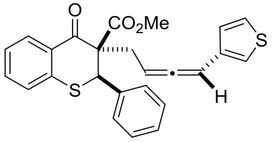

(+/-)-3cl

===
*** End of Report ***

Instrument 1 7/29/2022 4:48:29 AM   Page 1 of 1

Data File C:\CHEM32\1\DATA\ZHOU-22\YZN021584.D
Sample Name: LL-10-53C

===

Acq. Operator :
Acq. Instrument : Instrument 1   Location : -
Injection Date : 7/28/2022 10:38:21 PM
Acq. Method : C:\CHEM32\1\METHODS\DEF_LC.M
Last changed : 7/28/2022 10:37:54 PM
(modified after loading)
Analysis Method : C:\CHEM32\1\METHODS\DEF_LC.M
Last changed : 7/29/2022 4:46:30 AM
(modified after loading)
Sample Info : OD-3, Hexane/i-PrOH = 93/7, 0.8 mL/min, 30 oC, 254 nm

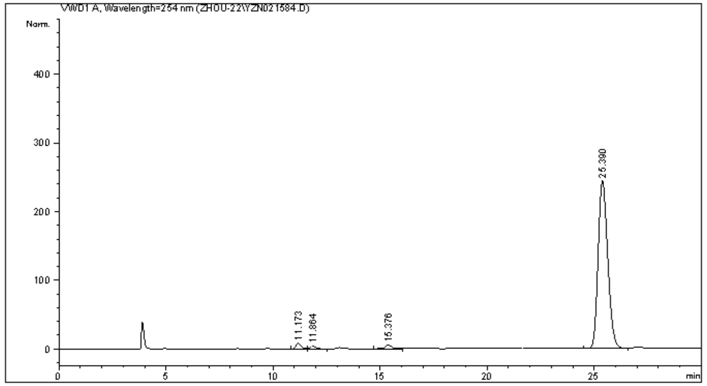

===
Area Percent Report
===

Sorted By : Signal
Multiplier: : 1.0000
Dilution: : 1.0000
Use Multiplier & Dilution Factor with ISTDs

Signal 1: VWD1 A, Wavelength=254 nm

Peak #	RetTime [min]	Type	Width [min]	Area mAU *s	Height [mAU]	Area %
1	11.173	BV	0.2393	129.58018	8.21898	1.6320
2	11.864	VB	0.2325	64.47478	4.24672	0.8120
3	15.376	BV	0.3116	113.67928	5.55369	1.4317
4	25.390	VV	0.4868	7632.28809	244.31071	96.1243

Totals : 7940.02232 262.33011

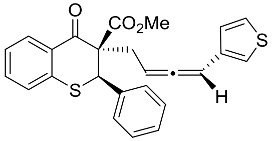

(-)-3cl

===
*** End of Report ***

Instrument 1 7/29/2022 4:46:33 AM   Page 1 of 1

Data File C:\CHEM32\1\DATA\ZHOU-22\YZN021630.D
Sample Name: LL-10-63A +/-

===

```
Acq. Operator   :
Acq. Instrument : Instrument 1                    Location :   -
Injection Date  : 8/25/2022 11:00:22 PM
Acq. Method     : C:\CHEM32\1\METHODS\DEF_LC.M
Last changed    : 8/25/2022 10:59:11 PM
                  (modified after loading)
Analysis Method : C:\CHEM32\1\METHODS\DEF_LC.M
Last changed    : 8/26/2022 9:11:16 PM
                  (modified after loading)
Sample Info     : AD-3, Hexane/i-PrOH = 97/3, 0.8 mL/min, 30 oC, 254 nm
```

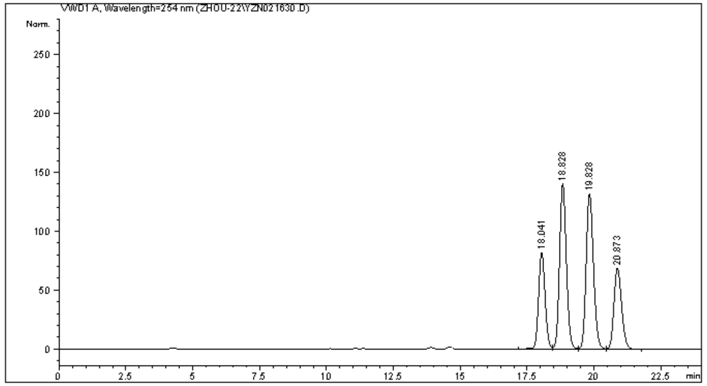

===
Area Percent Report
===

```
Sorted By             :      Signal
Multiplier:                  :      1.0000
Dilution:                    :      1.0000
Use Multiplier & Dilution Factor with ISTDs
```

Signal 1: VWD1 A, Wavelength=254 nm

Peak #	RetTime [min]	Type	Width [min]	Area mAU *s	Height [mAU]	Area %
1	18.041	VV	0.2632	1408.25598	82.50092	18.0563
2	18.828	VV	0.2746	2500.16602	140.51218	32.0565
3	19.828	VV	0.2947	2508.90479	131.92908	32.1685
4	20.873	VB	0.3121	1381.92310	69.06464	17.7187

Totals : 7799.24988 424.00681

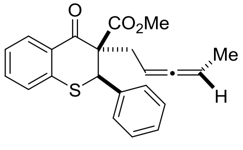

(+/-)-3cm

===
*** End of Report ***

Instrument 1 8/26/2022 9:11:27 PM Page 1 of 1

Data File C:\CHEM32\1\DATA\ZHOU-22\YZN021629.D
Sample Name: LL-10-63A

===

```
Acq. Operator   :
Acq. Instrument : Instrument 1                    Location :   -
Injection Date  : 8/25/2022 10:31:46 PM
Acq. Method     : C:\CHEM32\1\METHODS\DEF_LC.M
Last changed    : 8/25/2022 10:13:46 PM
                  (modified after loading)
Analysis Method : C:\CHEM32\1\METHODS\DEF_LC.M
Last changed    : 8/26/2022 9:29:54 PM
                  (modified after loading)
Sample Info     : AD-3, Hexane/i-PrOH = 97/3, 0.8 mL/min, 30 oC, 254 nm
```

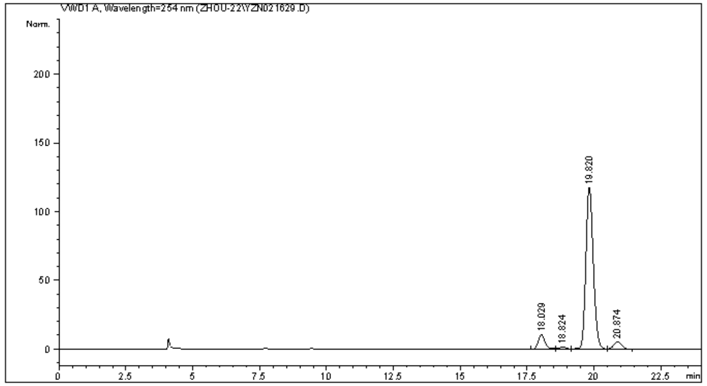

===
Area Percent Report
===

```
Sorted By             :      Signal
Multiplier:                  :      1.0000
Dilution:                    :      1.0000
Use Multiplier & Dilution Factor with ISTDs
```

Signal 1: VWD1 A, Wavelength=254 nm

Peak #	RetTime [min]	Type	Width [min]	Area mAU *s	Height [mAU]	Area %
1	18.029	VV	0.2715	188.61549	10.68129	7.2438
2	18.824	VV	0.2767	30.36503	1.68906	1.1662
3	19.820	VV	0.2983	2274.95874	117.72143	87.3704
4	20.874	VB	0.3135	109.87061	5.39045	4.2196

Totals : 2603.80988 135.48223

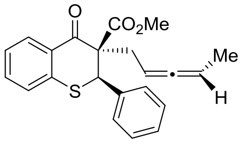

(-)-3cm

===
*** End of Report ***

Instrument 1 8/26/2022 9:31:10 PM Page 1 of 1

Data File C:\CHEM32\1\DATA\ZHOU-22\YZN022804.D
Sample Name: LL-12-21B +/-

===

Acq. Operator :
Acq. Instrument : Instrument 1 Location : -
Injection Date : 4/2/2023 4:16:47 AM
Acq. Method : C:\CHEM32\1\METHODS\DEF_LC.M
Last changed : 4/2/2023 3:58:06 AM
(modified after loading)
Analysis Method : C:\CHEM32\1\METHODS\DEF_LC.M
Last changed : 4/7/2023 8:20:59 AM
(modified after loading)
Sample Info : AD-H, Hexane/i-PrOH = 96/4, 1.0 mL/min, 30 oC, 254 nm

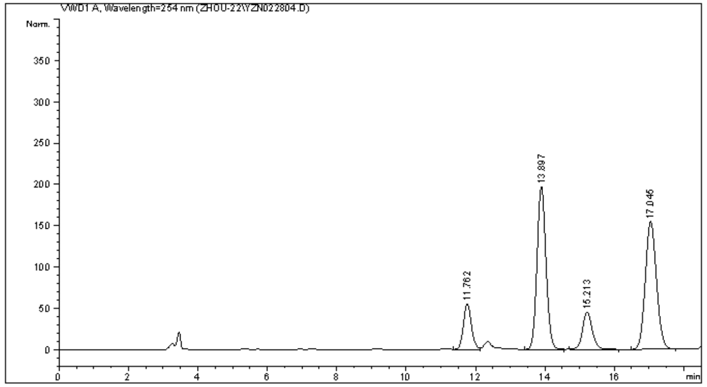

===

Area Percent Report

===

Sorted By : Signal
Multiplier: : 1.0000
Dilution: : 1.0000
Use Multiplier & Dilution Factor with ISTDs

Signal 1: VWD1 A, Wavelength=254 nm

Peak #	RetTime [min]	Type	Width [min]	Area mAU *s	Height [mAU]	Area %
1	11.762	VV	0.2461	884.03217	55.33675	10.0786
2	13.897	BB	0.2779	3541.40186	197.28014	40.3747
3	15.213	BB	0.2984	885.45581	45.20324	10.0949
4	17.045	BB	0.3474	3460.45874	154.38235	39.4518

Totals : 8771.34857 452.20248

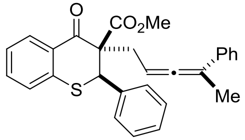

(+/-)-3cn

===

*** End of Report ***

Instrument 1 4/7/2023 8:21:08 AM Page 1 of 1

Data File C:\CHEM32\1\DATA\ZHOU-22\YZN022838.D
Sample Name: LL-12-21B

===

Acq. Operator :
Acq. Instrument : Instrument 1 Location : -
Injection Date : 4/7/2023 2:08:13 AM
Acq. Method : C:\CHEM32\1\METHODS\DEF_LC.M
Last changed : 4/7/2023 1:43:00 AM
(modified after loading)
Analysis Method : C:\CHEM32\1\METHODS\DEF_LC.M
Last changed : 4/7/2023 8:22:14 AM
(modified after loading)
Sample Info : AD-H, Hexane/i-PrOH = 96/4, 1.0 mL/min, 30 oC, 254 nm

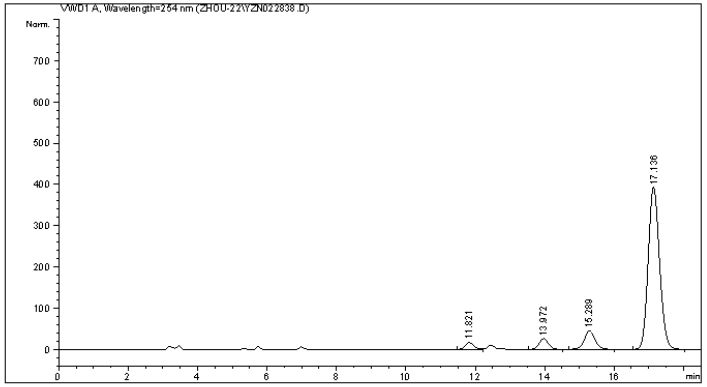

===

Area Percent Report

===

Sorted By : Signal
Multiplier: : 1.0000
Dilution: : 1.0000
Use Multiplier & Dilution Factor with ISTDs

Signal 1: VWD1 A, Wavelength=254 nm

Peak #	RetTime [min]	Type	Width [min]	Area mAU *s	Height [mAU]	Area %
1	11.821	BV	0.2516	282.20267	16.90334	2.6509
2	13.972	BB	0.2823	473.63223	26.01060	4.4492
3	15.289	BB	0.3257	954.33020	44.54163	8.9647
4	17.136	BB	0.3544	8935.21973	392.48340	83.9352

Totals : 1.06454e4 479.93896

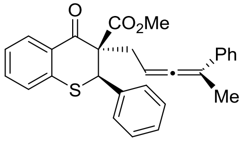

(-)-3cn

===

*** End of Report ***

Instrument 1 4/7/2023 8:23:10 AM Page 1 of 1

```
Data File C:\CHEM32\1\DATA\ZHOU-22\YZN021015.D
Sample Name: LL-9-57C +/-

=====================================================================
Acq. Operator   :
Acq. Instrument : Instrument 1                    Location :   -
Injection Date  : 4/21/2022 9:12:58 AM
Acq. Method     : C:\CHEM32\1\METHODS\DEF_LC.M
Last changed    : 4/21/2022 9:06:36 AM
                  (modified after loading)
Analysis Method : C:\CHEM32\1\METHODS\DEF_LC.M
Last changed    : 1/6/2023 8:34:42 PM
                  (modified after loading)
Sample Info     : IA, Hexane/i-PrOH = 94/6, 1.0 mL/min, 30 oC, 254 nm
```

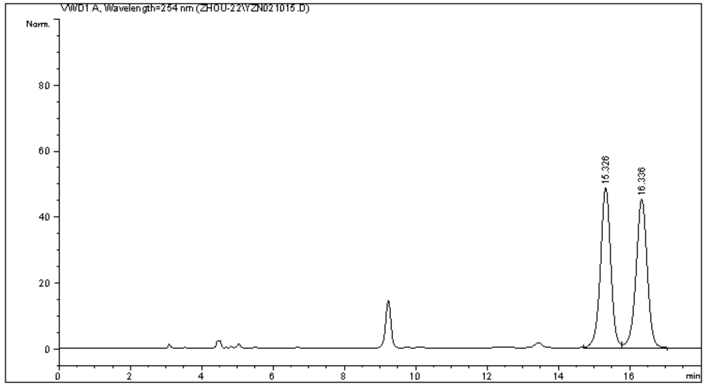


```
=====================================================================
                        Area Percent Report
=====================================================================

Sorted By             :      Signal
Multiplier:                  :      1.0000
Dilution:                    :      1.0000
Use Multiplier & Dilution Factor with ISTDs


Signal 1: VWD1 A, Wavelength=254 nm

Peak RetTime Type  Width     Area      Height     Area
  #   [min]        [min]  mAU   *s   [mAU   ]      %
----|-------|----|-------|----------|----------|--------|
   1  15.326 BV    0.3082  973.04175   48.52695  49.8425
   2  16.336 VB    0.3318  979.19135   45.12582  50.1575

Totals :                  1952.23309   93.65277
```

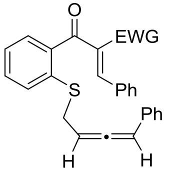

(+/-)-4aa

```
=====================================================================
                         *** End of Report ***
```

Instrument 1 1/6/2023 8:34:47 PM   Page 1 of 1

```
Data File C:\CHEM32\1\DATA\ZHOU-22\YZN021016.D
Sample Name: LL-9-57C

=====================================================================
Acq. Operator   :
Acq. Instrument : Instrument 1                    Location :   -
Injection Date  : 4/21/2022 9:32:34 AM
Acq. Method     : C:\CHEM32\1\METHODS\DEF_LC.M
Last changed    : 4/21/2022 9:31:05 AM
                  (modified after loading)
Analysis Method : C:\CHEM32\1\METHODS\DEF_LC.M
Last changed    : 1/6/2023 8:35:12 PM
                  (modified after loading)
Sample Info     : IA, Hexane/i-PrOH = 94/6, 1.0 mL/min, 30 oC, 254 nm
```

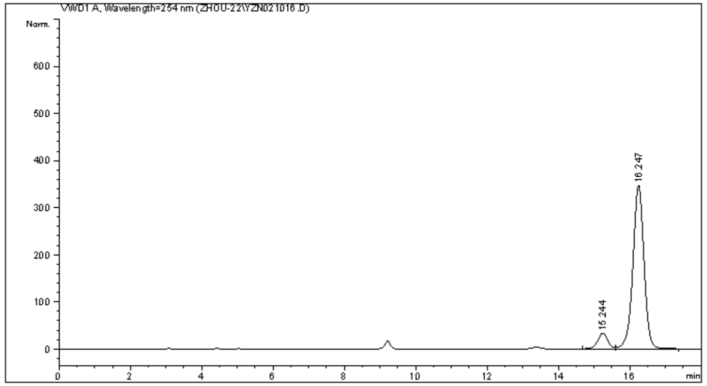


```
=====================================================================
                        Area Percent Report
=====================================================================

Sorted By             :      Signal
Multiplier:                  :      1.0000
Dilution:                    :      1.0000
Use Multiplier & Dilution Factor with ISTDs


Signal 1: VWD1 A, Wavelength=254 nm

Peak RetTime Type  Width     Area      Height     Area
  #   [min]        [min]  mAU   *s   [mAU   ]      %
----|-------|----|-------|----------|----------|--------|
   1  15.244 BV    0.3005  657.08240   33.45493   8.0540
   2  16.247 VB    0.3304 7501.42480  347.58792  91.9460

Totals :                  8158.50720  381.04285
```

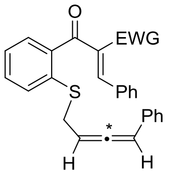

(-)-4aa

```
=====================================================================
                         *** End of Report ***
```

Instrument 1 1/6/2023 8:35:19 PM   Page 1 of 1

Data File C:\CHEM32\1\DATA\ZHOU-22\YZN021872.D
Sample Name: LL-11-4 +/-

===

Acq. Operator :
Acq. Instrument : Instrument 1 Location : -
Injection Date : 10/24/2022 10:30:45 PM
Acq. Method : C:\CHEM32\1\METHODS\DEF_LC.M
Last changed : 10/24/2022 10:29:24 PM
(modified after loading)
Analysis Method : C:\CHEM32\1\METHODS\DEF_LC.M
Last changed : 10/24/2022 11:53:01 PM
(modified after loading)
Sample Info : AD-3, Hexane/i-PrOH = 70/30, 0.7 mL/min, 30 oC, 254 nm

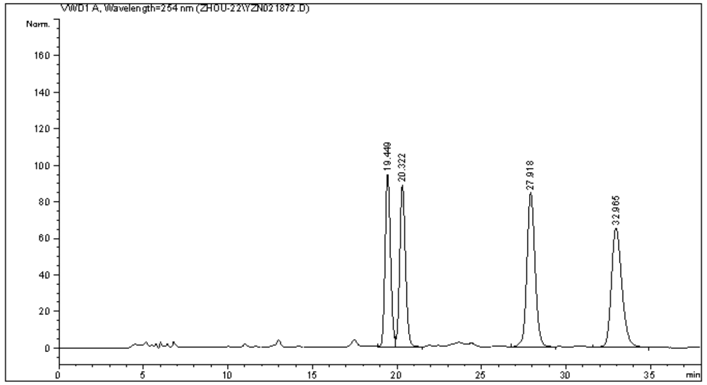

===
Area Percent Report
===

Sorted By : Signal
Multiplier: : 1.0000
Dilution: : 1.0000
Use Multiplier & Dilution Factor with ISTDs

Signal 1: VWD1 A, Wavelength=254 nm

Peak #	RetTime [min]	Type	Width [min]	Area mAU *s	Height [mAU]	Area %
1	19.449	VV	0.3459	2128.38428	94.98555	21.3251
2	20.322	VV	0.3698	2130.66040	88.91367	21.3479
3	27.918	VB	0.5186	2875.06445	84.64687	28.8064
4	32.965	VB	0.6695	2846.55029	65.57854	28.5207

Totals : 9980.65942 334.12463

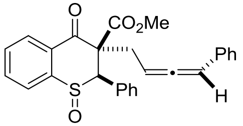

(+/-)-5

===
*** End of Report ***

Instrument 1 10/24/2022 11:53:05 PM Page 1 of 1

Data File C:\CHEM32\1\DATA\ZHOU-22\YZN021871.D
Sample Name: LL-11-4

===

Acq. Operator :
Acq. Instrument : Instrument 1 Location : -
Injection Date : 10/24/2022 9:50:34 PM
Acq. Method : C:\CHEM32\1\METHODS\DEF_LC.M
Last changed : 10/24/2022 9:29:28 PM
(modified after loading)
Analysis Method : C:\CHEM32\1\METHODS\DEF_LC.M
Last changed : 10/24/2022 11:54:40 PM
(modified after loading)
Sample Info : AD-3, Hexane/i-PrOH = 70/30, 0.7 mL/min, 30 oC, 254 nm

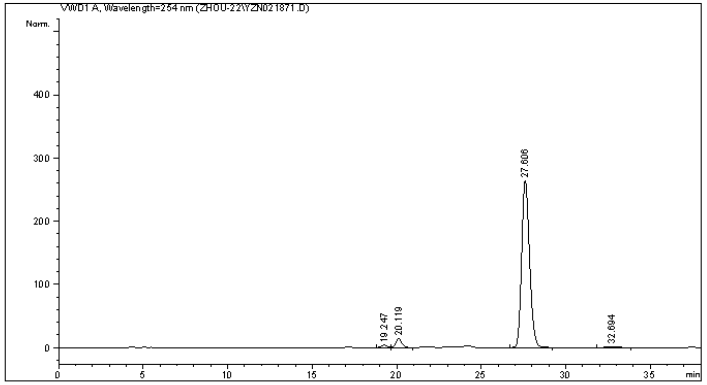

===
Area Percent Report
===

Sorted By : Signal
Multiplier: : 1.0000
Dilution: : 1.0000
Use Multiplier & Dilution Factor with ISTDs

Signal 1: VWD1 A, Wavelength=254 nm

Peak #	RetTime [min]	Type	Width [min]	Area mAU *s	Height [mAU]	Area %
1	19.247	BV	0.3477	84.40138	3.78293	0.9343
2	20.119	VB	0.3672	331.54840	14.11645	3.6701
3	27.606	BB	0.5067	8566.81055	264.04214	94.8319
4	32.694	BB	0.7352	50.92006	1.03840	0.5637

Totals : 9033.68039 282.97992

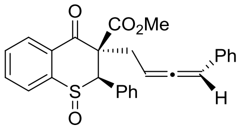

(-)-5

===
*** End of Report ***

Instrument 1 10/24/2022 11:56:26 PM Page 1 of 1

Data File C:\CHEM32\1\DATA\ZHOU-22\YZN021841.D
Sample Name: LL-11-5 +/-

===

Acq. Operator   :
Acq. Instrument : Instrument 1   Location :   -
Injection Date : 10/22/2022 3:08:51 AM
Acq. Method   : C:\CHEM32\1\METHODS\DEF_LC.M
Last changed   : 10/22/2022 3:04:06 AM
                  (modified after loading)
Analysis Method : C:\CHEM32\1\METHODS\DEF_LC.M
Last changed   : 10/22/2022 10:51:46 PM
                  (modified after loading)
Sample Info   : IC, Hexane/i-PrOH = 65/35, 0.8 mL/min, 30 oC, 254 nm

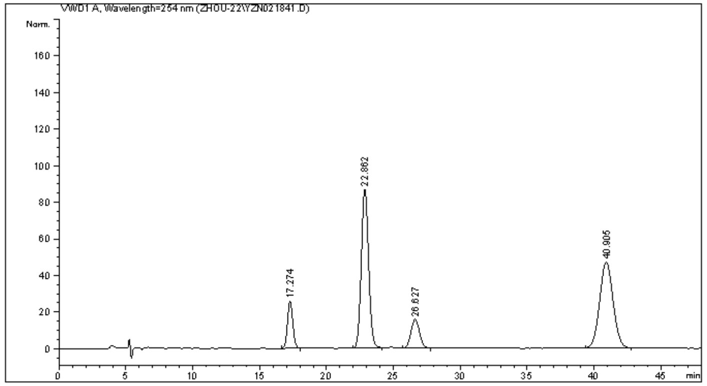

===
Area Percent Report
===

Sorted By   :   Signal
Multiplier: :   1.0000
Dilution: :   1.0000
Use Multiplier & Dilution Factor with ISTDs

Signal 1: VWD1 A, Wavelength=254 nm

Peak #	RetTime [min]	Type	Width [min]	Area mAU *s	Height [mAU]	Area %
1	17.274	BB	0.4320	715.35370	25.74029	8.9740
2	22.862	BB	0.5889	3294.61182	86.98376	41.3303
3	26.627	BB	0.6896	700.55835	15.74153	8.7884
4	40.905	BB	1.0814	3260.90479	46.85238	40.9074
Totals :				7971.42865	175.31795	

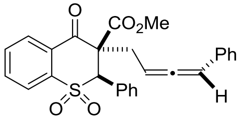

(+/-)-6

===
*** End of Report ***

Instrument 1 10/22/2022 10:51:52 PM   Page 1 of 1

Data File C:\CHEM32\1\DATA\ZHOU-22\YZN021840.D
Sample Name: LL-11-5

===

Acq. Operator   :
Acq. Instrument : Instrument 1   Location :   -
Injection Date : 10/22/2022 2:15:24 AM
Acq. Method   : C:\CHEM32\1\METHODS\DEF_LC.M
Last changed   : 10/22/2022 1:41:04 AM
                  (modified after loading)
Analysis Method : C:\CHEM32\1\METHODS\DEF_LC.M
Last changed   : 10/22/2022 10:53:50 PM
                  (modified after loading)
Sample Info   : IC, Hexane/i-PrOH = 65/35, 0.8 mL/min, 30 oC, 254 nm

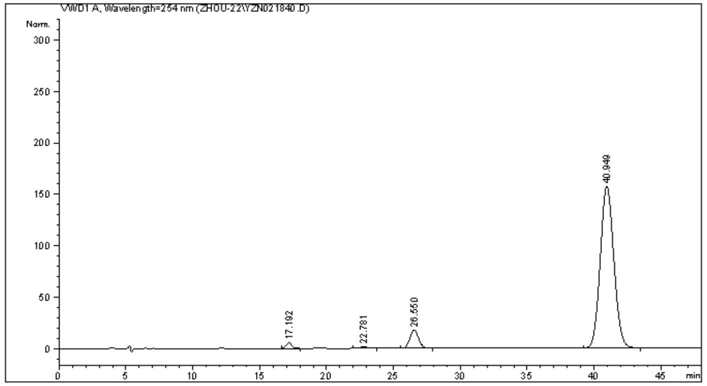

===
Area Percent Report
===

Sorted By   :   Signal
Multiplier: :   1.0000
Dilution: :   1.0000
Use Multiplier & Dilution Factor with ISTDs

Signal 1: VWD1 A, Wavelength=254 nm

Peak #	RetTime [min]	Type	Width [min]	Area mAU *s	Height [mAU]	Area %
1	17.192	BB	0.4332	159.04973	5.70220	1.3105
2	22.781	BB	0.6010	60.96534	1.57199	0.5023
3	26.550	BB	0.7082	808.95935	17.88644	6.6653
4	40.949	BB	1.1047	1.11079e4	157.05879	91.5219
Totals :				1.21369e4	182.21943	

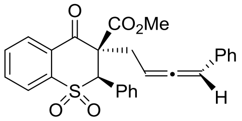

(-)-6

===
*** End of Report ***

Instrument 1 10/22/2022 10:55:27 PM   Page 1 of 1

Data File C:\CHEM32\1\DATA\ZHOU-22\YZN021917.D
Sample Name: LL-11-16 +/-

Acq. Operator :
Acq. Instrument : Instrument 1 Location : -
Injection Date : 10/30/2022 3:52:27 AM
Acq. Method : C:\CHEM32\1\METHODS\DEF_LC.M
Last changed : 10/30/2022 3:50:56 AM
(modified after loading)
Analysis Method : C:\CHEM32\1\METHODS\DEF_LC.M
Last changed : 10/30/2022 4:16:07 AM
(modified after loading)
Sample Info : AD-H, Hexane/i-PrOH = 95/5, 1.0 mL/min, 30 oC, 254 nm

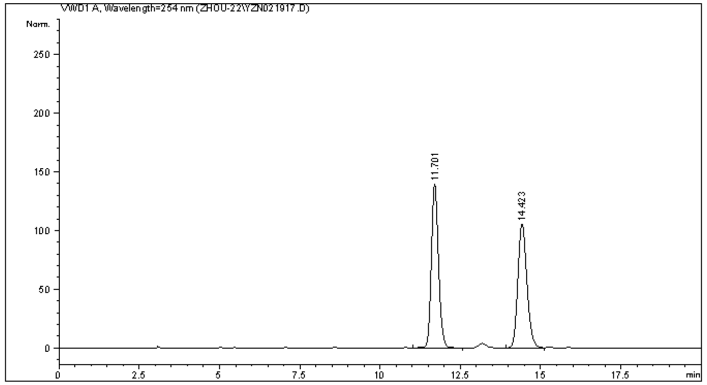

Area Percent Report

Sorted By : Signal
Multiplier: : 1.0000
Dilution: : 1.0000
Use Multiplier & Dilution Factor with ISTDs

Signal 1: VWD1 A, Wavelength=254 nm

Peak #	RetTime [min]	Type	Width [min]	Area mAU *s	Height [mAU]	Area %
1	11.701	VB	0.2371	2156.65771	139.52293	50.8865
2	14.423	VV	0.3026	2081.51245	105.66568	49.1135
Totals :				4238.17017	245.18861	

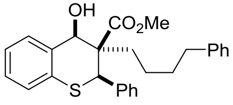

(+/-)-7

*** End of Report ***

Instrument 1 10/30/2022 4:16:15 AM Page 1 of 1

Data File C:\CHEM32\1\DATA\ZHOU-22\YZN021916.D
Sample Name: LL-11-16

Acq. Operator :
Acq. Instrument : Instrument 1 Location : -
Injection Date : 10/30/2022 3:28:32 AM
Acq. Method : C:\CHEM32\1\METHODS\DEF_LC.M
Last changed : 10/30/2022 3:04:05 AM
(modified after loading)
Analysis Method : C:\CHEM32\1\METHODS\DEF_LC.M
Last changed : 10/30/2022 4:17:32 AM
(modified after loading)
Sample Info : AD-H, Hexane/i-PrOH = 95/5, 1.0 mL/min, 30 oC, 254 nm

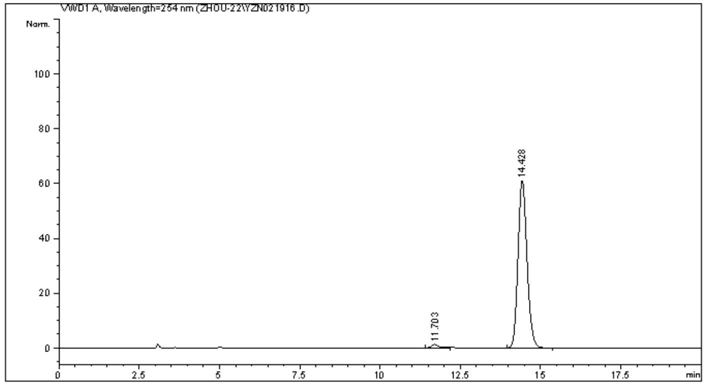

Area Percent Report

Sorted By : Signal
Multiplier: : 1.0000
Dilution: : 1.0000
Use Multiplier & Dilution Factor with ISTDs

Signal 1: VWD1 A, Wavelength=254 nm

Peak #	RetTime [min]	Type	Width [min]	Area mAU *s	Height [mAU]	Area %
1	11.703	VB	0.2459	19.75402	1.21869	1.6311
2	14.428	BB	0.3005	1191.35352	61.03853	98.3689
Totals :				1211.10754	62.25722	

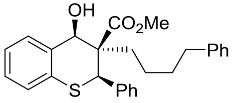

(+)-7

*** End of Report ***

Instrument 1 10/30/2022 4:17:48 AM Page 1 of 1

Data File C:\CHEM32\1\DATA\ZHOU-22\YZN022147.D
Sample Name: LL-11-44 +/-

Acq. Operator	:
Acq. Instrument	: Instrument 1 Location : -
Injection Date	: 11/30/2022 11:30:47 PM
Acq. Method	: C:\CHEM32\1\METHODS\DEF_LC.M
Last changed	: 11/30/2022 11:29:14 PM (modified after loading)
Analysis Method	: C:\CHEM32\1\METHODS\DEF_LC.M
Last changed	: 12/1/2022 2:31:03 AM (modified after loading)
Sample Info	: IA+AS-H, Hexane/i-PrOH = 95/5, 0.8 mL/min, 30 oC, 254 nm

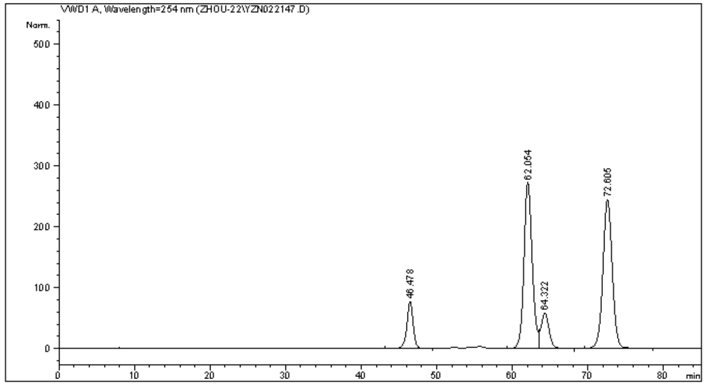

Area Percent Report

Sorted By : Signal
Multiplier: : 1.0000
Dilution: : 1.0000
Use Multiplier & Dilution Factor with ISTDs

Signal 1: VWD1 A, Wavelength=254 nm

Peak #	RetTime [min]	Type	Width [min]	Area mAU *s	Height [mAU]	Area %
1	46.478	BB	0.8766	4460.64746	77.35817	8.8316
2	62.054	BV	1.1644	2.06928e4	272.83978	40.9695
3	64.322	VB	1.1556	4356.79199	58.02239	8.6260
4	72.605	BB	1.3270	2.09975e4	244.75700	41.5729
Totals :				5.05077e4	652.97735	

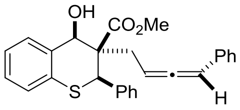

(+/-)-8

*** End of Report ***

Instrument 1 12/1/2022 2:31:11 AM Page 1 of 1

Data File C:\CHEM32\1\DATA\ZHOU-22\YZN022146.D
Sample Name: LL-11-44

Acq. Operator	:
Acq. Instrument	: Instrument 1 Location : -
Injection Date	: 11/30/2022 9:53:50 PM
Acq. Method	: C:\CHEM32\1\METHODS\DEF_LC.M
Last changed	: 11/30/2022 9:51:30 PM (modified after loading)
Analysis Method	: C:\CHEM32\1\METHODS\DEF_LC.M
Last changed	: 12/1/2022 2:28:53 AM (modified after loading)
Sample Info	: IA+AS-H, Hexane/i-PrOH = 95/5, 0.8 mL/min, 30 oC, 254 nm

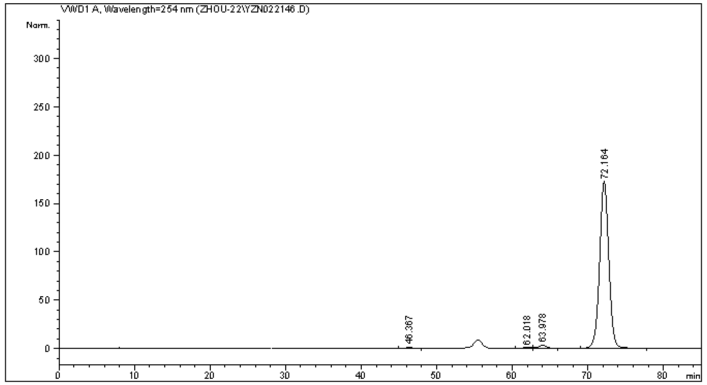

Area Percent Report

Sorted By : Signal
Multiplier: : 1.0000
Dilution: : 1.0000
Use Multiplier & Dilution Factor with ISTDs

Signal 1: VWD1 A, Wavelength=254 nm

Peak #	RetTime [min]	Type	Width [min]	Area mAU *s	Height [mAU]	Area %
1	46.367	BB	0.8741	62.76188	1.09244	0.4206
2	62.018	BV	1.1131	68.22928	9.22701e-1	0.4573
3	63.978	VB	1.1444	235.30170	3.15277	1.5770
4	72.164	BB	1.2935	1.45549e4	172.42085	97.5451
Totals :				1.49212e4	177.58876	

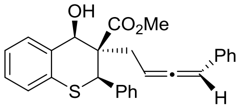

(-)-8

*** End of Report ***

Instrument 1 12/1/2022 2:28:56 AM Page 1 of 1
